# Pd(II)-Catalyzed Enantioselective C(sp^3^)–H Arylation of Cyclopropanes and Cyclobutanes Guided by Tertiary Alkylamines

**DOI:** 10.1021/jacs.1c11921

**Published:** 2022-02-25

**Authors:** Jesus Rodrigalvarez, Luke A. Reeve, Javier Miró, Matthew J. Gaunt

**Affiliations:** Yusuf Hamied Department of Chemistry, University of Cambridge, Lensfield Road, Cambridge CB2 1EW, United Kingdom

## Abstract

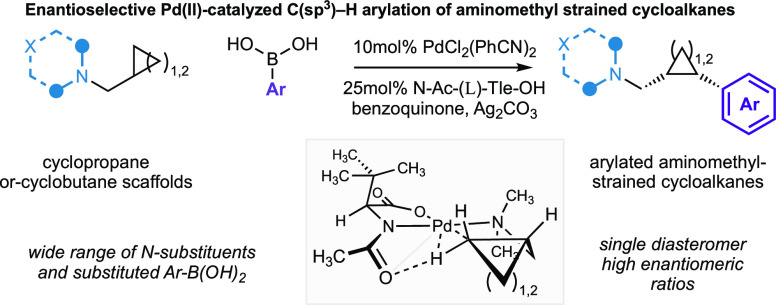

Strained aminomethyl-cycloalkanes are a recurrent scaffold in medicinal chemistry due to their unique structural features that give rise to a range of biological properties. Here, we report a palladium-catalyzed enantioselective C(sp^3^)–H arylation of aminomethyl-cyclopropanes and -cyclobutanes with aryl boronic acids. A range of native tertiary alkylamine groups are able to direct C–H cleavage and forge carbon-aryl bonds on the strained cycloalkanes framework as single diastereomers and with excellent enantiomeric ratios. Central to the success of this strategy is the use of a simple *N*-acetyl amino acid ligand, which not only controls the enantioselectivity but also promotes γ-C–H activation of over other pathways. Computational analysis of the cyclopalladation step provides an understanding of how enantioselective C–H cleavage occurs and revealed distinct transition structures to our previous work on enantioselective desymmetrization of *N*-isobutyl tertiary alkylamines. This straightforward and operationally simple method simplifies the construction of functionalized aminomethyl-strained cycloalkanes, which we believe will find widespread use in academic and industrial settings relating to the synthesis of biologically active small molecules.

## Introduction

**Figure 1:**
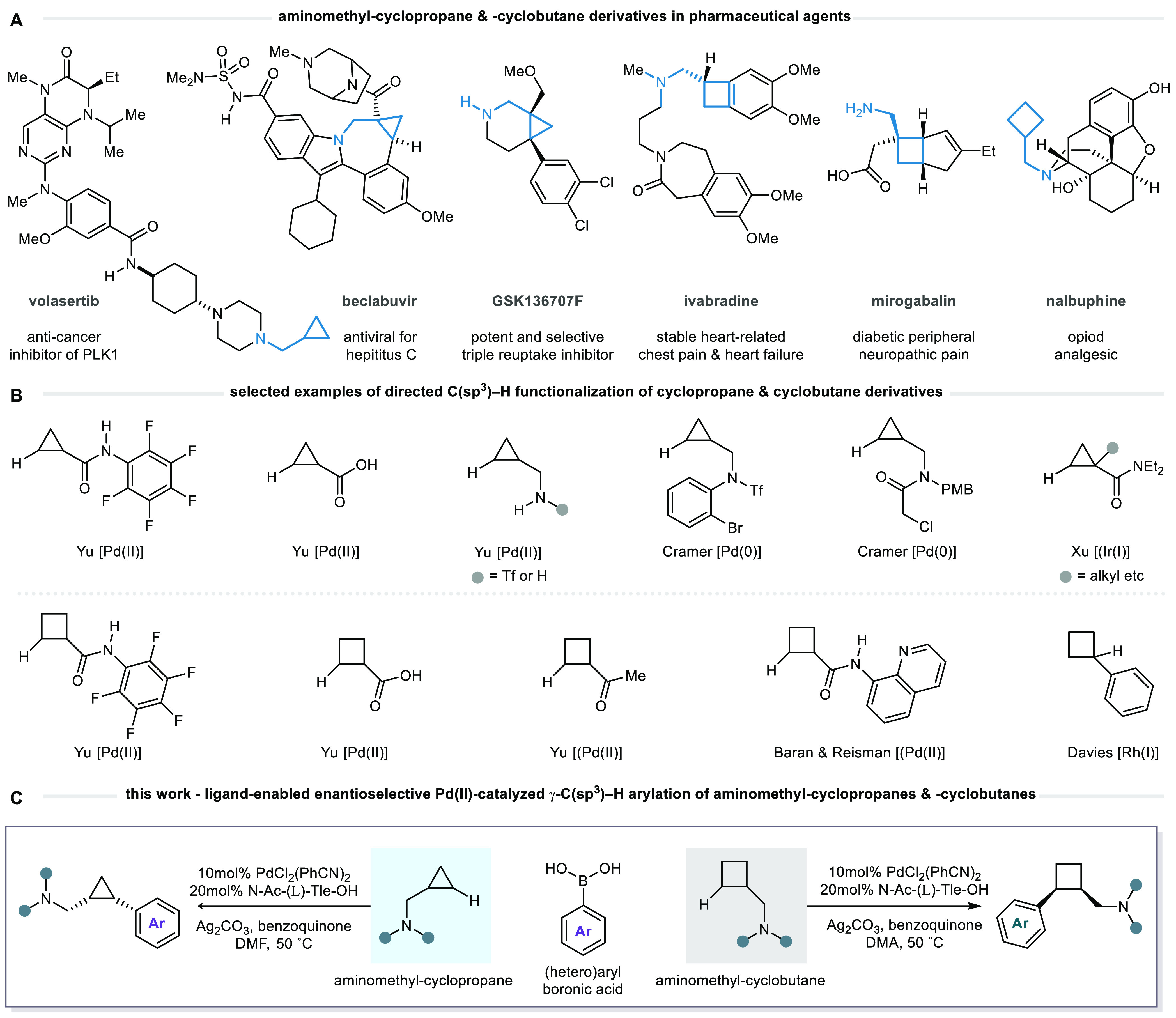
(A) Selected pharmaceuticals containing cyclobutanes and cyclopropanes. (B) Selected C–H activation reactions on cyclobutanes and cyclopropanes. (C) Pd(II)-catalyzed enantioselective C(sp^3^)–H arylation of aminomethyl-cyclopropanes and -cyclobutanes directed by unbiased tertiary alkylamine.

Strained cycloalkanes displaying an aminomethyl-substituent are common features in pharmaceutical candidates and approved drugs as well as agrochemicals. These small polar scaffolds frequently convey important physical features that lead to enhanced biological properties, when compared with linear *N*-alkyl congeners (Figure 1A).^1^ In particular, cyclopropane and cyclobutane derivatives can boost metabolic stability and reduce lipophilicity when used as bioisosteres of *gem-*dimethyl, isopropyl, or phenyl groups, which results from a combination of high coplanarity of the ring-carbon atoms, relatively shorter C–C bonds, enhanced π-character, and shorter and stronger C–H bonds. Furthermore, the well-defined exit vectors of these rigid cycloalkanes make them ideal as scaffold candidates through which to probe distinct spatial environments, particularly through their deployment as single enantiomers.^2^ As a result of these properties, the preparation of functionally diverse nonracemic aminomethyl-cyclopropanes (AMCPs) and aminomethyl-cyclobutanes (AMCBs) represents an important challenge for chemical synthesis. While the synthesis of simple unfunctionalized variants of aminomethyl-strained cycloalkanes can be achieved via *N*-alkylation, reductive amination, or amide reduction with readily available strained cycloalkane-containing starting materials, the synthesis of more complex, densely functionalized variants frequently requires multiple steps as a result of the problematic amine functionality that precludes the effective use of many of the well-established ring formation protocols.^3^

Metal-catalyzed C(sp^3^)–H functionalization of simple monofunctionalized strained cycloalkane frameworks has emerged as a powerful alternative strategy (to *de novo* methods)^3^ for the synthesis of higher order variants, in particular, on cyclopropane scaffolds (Figure 1B). Yu and co-workers have reported a series of Pd(II)-C(sp^3^)–H functionalization reactions on cyclopropane derivatives directed by *N*-arylcarboxamides,^4a,4b^*N*-triflamides,^4c^ carboxylic acids,^4d^ and primary amines,^4e^ many of which can be rendered enantioselective. Cramer and co-workers exploited oxidative addition to a pendant bromoarene motif to direct intramolecular Pd(0)-catalyzed C(sp^3^)–H arylation onto triflimide-protected *N*-aryl-aminomethyl-cyclopropanes.^5a^ This approach was also extended to a number of other tethering units to formulate an approach to the synthesis of bicyclic systems containing a substituted cyclopropane unit and, in many cases, could be carried out enantioselectively.^5c,5d^ Xu and co-workers reported an Ir-catalyzed C(sp^3^)–H borylation directed by a carboxamide motif.^6^

In contrast, the deployment of Pd(II)-catalyzed C(sp^3^)–H functionalization strategies on cyclobutane scaffolds is less common (Figure 1B). Yu and co-workers were able to extend their seminal carboxamide-directed C(sp^3^)–H arylation of cyclopropanes to the corresponding cyclobutane frameworks.^7a−7c^ Subsequent advances enabled the deployment of native carboxylic acids,^7d^ ketones (via transiently generated imines),^7e^ and oximes^7f^ as directing groups for a selection of C(sp^3^)–H functionalization reactions, many of which could, again, be rendered enantioselective using a range of ligand-controlled strategies. Baran and Reisman have shown, independently, that reactivity augmenting auxiliary-directed C–H arylation can be leveraged for the synthesis of di- and trisubstituted cyclobutane derivatives.^8^ Finally, Davies and co-workers reported a nondirected C–H arylation of aryl-cyclobutanes through the reaction of catalytically generated Rh-carbenoids.^9^ Considering the demonstrated importance of aminomethyl-cyclopropanes and -cyclobutanes, harnessing the native tertiary amine functionality to direct C–H transformations on the ring framework would provide a powerful tool for the streamlined synthesis of complex variants of these substituted strained cycloalkanes.

Here, we report the development of a Pd(II)-catalyzed process capable of affecting enantioselective desymmetrizing arylation of methylene-C(sp^3^)–H bonds in aminomethyl-cyclopropanes and -cyclobutanes (Figure 1C). The reaction platform exploits the versatile coordination capacity of native, unbiased tertiary alkylamines, which are replete of reactivity-augmenting auxiliary groups. A broad scope is presented across a series of strained cycloalkanes and transferring aryl groups, leading to nonracemic *cis*-substituted cyclic products with high enantiomeric ratios. The multifaceted role of a commercial *N*-acetyl-amino acid ligand not only enables the cycloalkane desymmetrization process but it can also be applied in a kinetic resolution-type mode to form trisubstituted aminomethyl-cyclopropanes, which together with the basic transformation will be of interest to practitioners of synthetic chemistry tasked with preparing biologically active small molecules.^1^

## Results and Discussion

**Figure 2:**
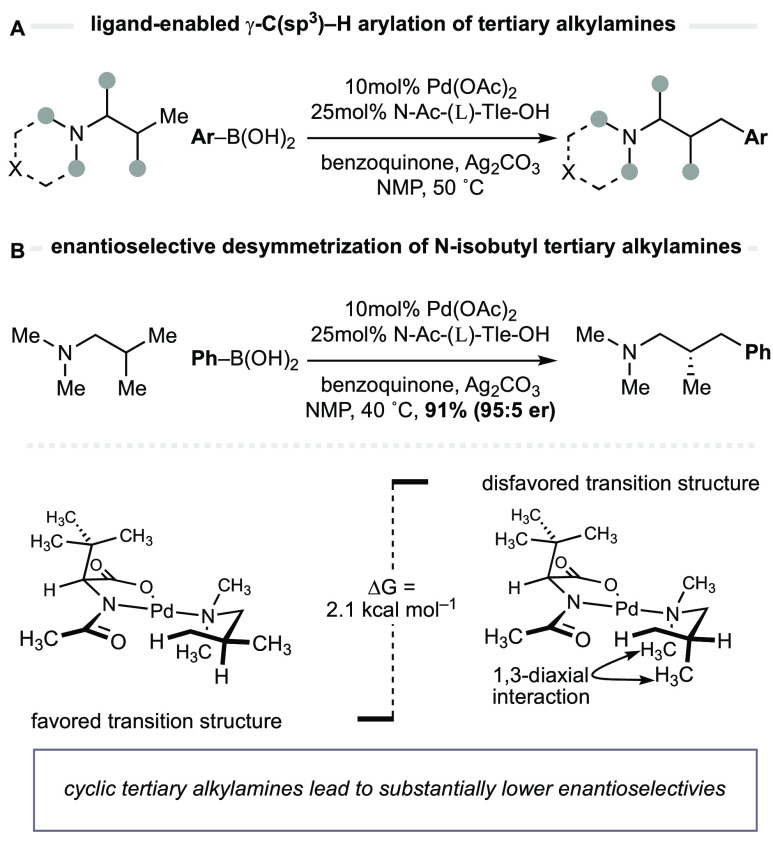
Previous work on Pd(II)-catalyzed γ-C(sp^3^)–H arylation of tertiary alkylamines.

Over the last 7 years, our group has established the use of unprotected free(NH)-alkylamines in Pd(II)-catalyzed C(sp^3^)–H functionalization.^10^ The use of amines in their native form significantly advances their synthetic utility by precluding the need for additional multistep procedures to add and remove auxiliary directing functionalities. Central to the success of many of these transformations was the exploitation of an intramolecular hydrogen bond between the carbonyl oxygen atom of the Pd(II)-bound carboxylate and the NH motif of the ligated amine, which oriented the substrate such that the C–H bond aligned with the requisite carboxylate ligand for C–H bond cleavage.^11^ However, this platform cannot be extended to tertiary alkylamine-directed C(sp^3^)–H activation because there is no NH feature in these substrates. In addressing this, we discovered that a ligand-directed strategy, wherein an *N*-acyl amino acid ligand^12^ was able to promote a C(sp^3^)–H activation event over competitive β-hydride elimination pathways, which had presumably precluded the use of tertiary alkylamines in C–H activation reactions prior to our work (Figure 2A). Crucial to the success of this activation platform was a relay effect originating from the α-substituent on the amino acid ligand which oriented the acetamide group in perfect alignment for γ-C–H bond cleavage in preference to the corresponding β-hydride elimination pathway. Accordingly, a general γ-C(sp^3^)–H arylation platform was developed which coupled γ-methyl groups in a wide range of tertiary alkylamines with aryl-boronic acids.^13^ Furthermore, the chiral nature of the *N*-acetyl-*t*-leucine ligand was exploited through an enantioselective desymmetrization method for *N*-isobutyl-derived tertiary alkylamines (Figure 2B). The origin of the enantioselectivity is thought to arise from minimization of 1,3-diaxial interactions between the nonreacting *N*-substituent and the nonreacting methyl group on the reacting alkyl chain of the substrate within the two lowest-energy conformations of chair-like six-membered ring transition structures. However, asymmetric induction was highly dependent on the structure of the nonreacting amine substituents: Acyclic tertiary alkylamines delivered products in good yield and with high enantioselectivity, whereas substrates directed through a *N*-heterocycle motif performed modestly across a range of examples and ultimately limited the wider efficacy of the transformation. In these cases, we believe that interactions between the catalyst and saturated heterocycle framework—not present with smaller acyclic substituents—disturb the ideal conformation of the transition structures and lead to poorer enantioselectivity.

As part of the evolution of the tertiary alkylamine-directed platform, we questioned whether enantioselective γ-methylene C(sp^3^)–H arylation could be achieved on the strained ring framework of aminomethyl-cyclopropanes and cyclobutanes. If the reaction was able to accommodate an unbiased range of *N*-substituents on the tertiary alkylamine function, then the products of such a transformation could have widespread utility in the construction of nonracemic complex strained cycloalkane scaffolds that are prevalent in biologically relevant small molecules.

Investigations toward the development of a γ-methylene C(sp^3^)–H arylation on AMCP scaffolds began by reacting amine **1a** with phenyl boronic acid **2a** under conditions related to our previous studies (Table 1, entry 1).^13^ With 3 equiv of amine **1a**, a reaction using 10 mol % of Pd(OAc)_2_, 20 mol % of N-Ac-(l)-Tle-OH, 2.5 equiv of Ag_2_CO_3_, and 2.0 equiv of 1,4-benzoquinone at 50 °C delivered an 94% assay yield (determined by ^1^H NMR) of a single *cis*-substituted γ-arylated cyclopropane (**3a**), with a 99:1 enantiomeric ratio (e.r.). However, we were surprised to find that a reaction without the ligand delivered a 12% assay yield of racemic **3a** (entry 2), which is in contrast to the corresponding γ-methyl C(sp^3^)–H arylation on linear *N*-propyl tertiary alkylamines where no background reaction was observed.^13^ Given that the acetate anion of the Pd(OAc)_2_ appears capable of affecting the γ-methylene C(sp^3^)–H activation on AMCPs, albeit at low conversion, we were concerned that in less reactive systems, this deleterious pathway might become more dominant and thereby erode enantioselectivity. We reasoned that a palladium catalyst without the acetate counteranion might obviate the background reaction. We were pleased to find that when using 10 mol % of Pd(PhCN)_2_Cl_2_, the reaction still had excellent assay yield and enantioselectivity, but importantly afforded no background reaction in the absence of the *N*-acetyl amino acid ligand (entries 3 and 4). Further tuning of the reaction parameters delivered an optimized protocol that involved stirring a DMF solution of phenyl boronic acid, amine **1a** (1.5 equiv), benzoquinone (1.0 equiv), Pd(PhCN)_2_Cl_2_ (10 mol %), and *N*-acetyl *tert*-(l)-leucine (20 mol %) at 40 °C for 15 h, to afford 82% yield of product **3a**, after chromatographic purification, with an e.r. of >99:1 (entry 5). It is interesting to note that a reaction using amine **1a** as the limiting reagent (with 2 equiv of PhB(OH)_2_**2a**) gave a 58% assay yield of **3a**. We believe it is possible that a modest excess of amine is required to compete with a product inhibition through ligation to the palladium catalyst.

**Table 1:**
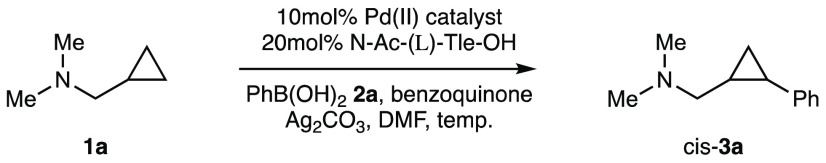
Selected Optimization for γ-C–H Arylation of Cyclopropane Tertiary Amines

	Pd cat.	*T* (°C)	**1a** (equiv )	Ag_2_CO_3_ (equiv )	BQ (equiv )	yield^a^**3a** (%)	e.r. (%)
1	Pd(OAc)_2_	50	3.0	2.5	2.0	94	99:1
2^a^	Pd(OAc)_2_*No ligand*	50	3.0	2.5	2.0	12	0
3	Pd(PhCN)_2_Cl_2_	50	3.0	2.5	2.0	93	>99:1
4^a^	Pd(PhCN)_2_Cl_2_*No ligand*	50	3.0	2.5	2.0	0	–
5	Pd(PhCN)_2_Cl_2_	40	1.5	1.5	1.0	88 (82^b^)	>99:1

a Yields were determined by ^1^H NMR using 1,1,2,2-tetrachloroethane as an internal standard.

b Yield of isolated product after purification by silica gel chromatography.

**Figure 3:**
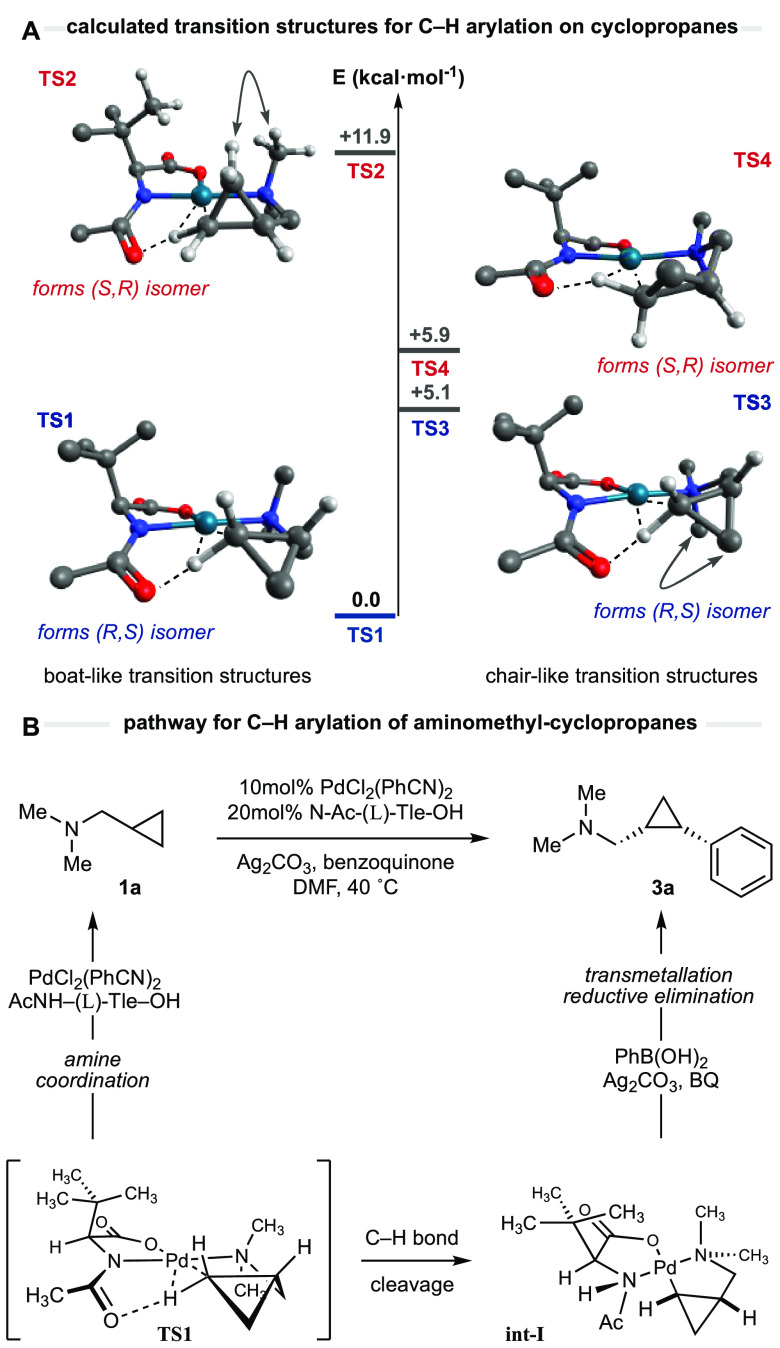
(A) Computational analysis of the enantiodetermining C–H cleavage on aminomethyl-cyclopropanes. Basis set B3LyP-D3(BJ)/[6-311+G(2d,p)/ SDD(Pd)]. (B) Proposed pathway for of aminomethyl-cyclopropanes via Pd(II)-catalyzed enantioselective γ-methylene-C(sp^3^)–H arylation.

In lieu of a crystalline sample of product **3a**, we initially predicted that the model for γ-methyl C(sp^3^)–H arylation of *N*-isobutyl tertiary alkylamine would provide an accurate rationale for the stereochemical outcome on the cyclopropane system; minimization of the 1,3-diaxial interactions between nonreacting groups on the nitrogen atom and the cyclopropane ring in the reacting chain would be the dominating feature determining the lowest energy pathway (Figure 2B). However, the rigid cyclopropane framework would likely instill geometric restrictions into the chair-like transition structures based on the *N*-isobutyl tertiary alkylamine model. Accordingly, we calculated new transition structures for the γ-methylene C(sp^3^)–H activation on the aminomethyl-cyclopropane scaffold (Figure 3A) and found that amine **1a** generated boat-like **TS1** as the lowest-energy form. **TS1** displays the empirically required conformation for C(sp^3^)–H cleavage, where the amido-palladium (O=C–N–Pd) dihedral angle of 11.5° serves to arrange the cyclopropane ring so that its steric interactions with the nonreacting *N*-substituents are minimized.^14^**TS2**, an alternative boat-like transition structure, is substantially higher in energy and displays interactions between the cyclopropane ring and the nonreacting *N*-substituent. A chair-like transition structure (**TS3**), similar to that found for the reaction of *N*-isobutyl tertiary alkylamines, appears to be destabilized by pseudo 1,3-diaxial interaction between one of the nonreacting *N*-substituents (axial) and a CH_2_ unit of the cyclopropane, increasing the energy by 5.1 kcal·mol^–1^. A final transition state that is worthy of comment is **TS4**, which was found to be 5.9 kcal·mol^–1^ higher than **TS1** and appears to be destabilized by torsional interactions. Therefore, a pathway through **TS1** would deliver palladacyclic intermediate **int-I**, and benzoquinone-assisted reductive elimination would be expected to form the (1*R*,2*S*)-aryl-substituted cyclopropane **3a** (Figure 3B).

**Chart 1:**
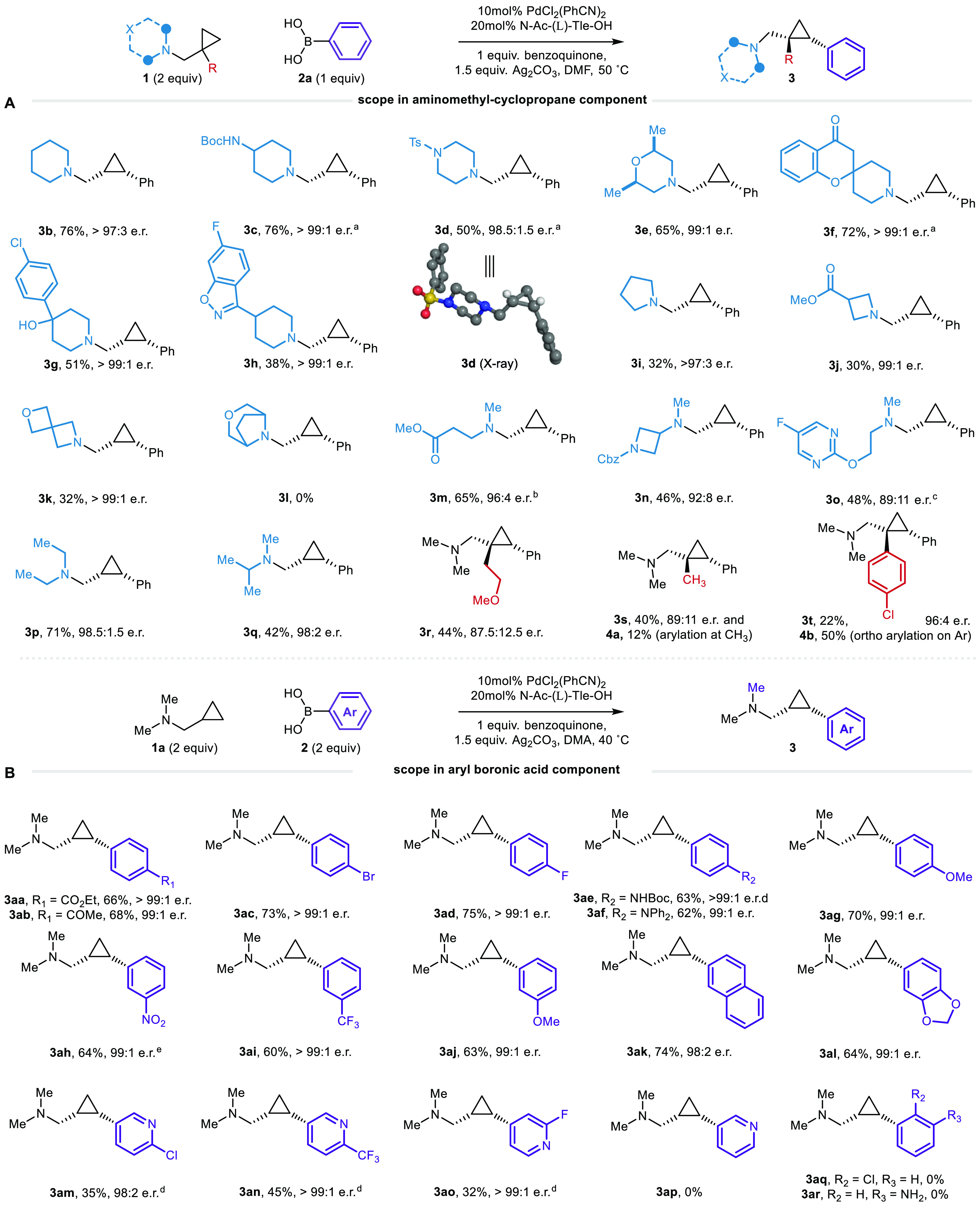
Scope of Enantioselective γ-Methylene C(sp^3^)–H Arylation of Aminomethyl-cyclopropanes

With a set of optimized conditions for a γ-methylene C(sp^3^)–H arylation on AMCPs and a basic understanding of the factors controlling the stereoinduction, we set about exploring the scope of this new enantioselective transformation (Chart 1). An important part of these studies was determining the range of nonreacting amine substituents that were accommodated in the reaction. Our previous studies on a γ-methyl C(sp^3^)–H arylation on *N*-isobutyl tertiary alkylamines had shown a clear limitation in the scope of the amine heterocycles amenable to this transformation; the e.r. of the products was substantially elevated only when acyclic substituents were displayed part of the amine. Therefore, we were pleased to find that a piperidine-derived AMCP also reacted well under the standard conditions and produced the arylated product **3b** with >96:4 e.r. (Chart 1A). A selection of other nitrogen-containing six-membered ring heterocycle-derived AMCPs (**3c**–**h**), displaying a variety of functional motifs and features common to pharmaceutical agents, also performed well, giving products with >99:1 e.r. For example, piperazine (**3d**) and morpholine (**3e**)-derived substrates produced reasonable yields of the corresponding arylated cyclopropanes, again, with excellent e.r’s. *N*-Tosyl-piperazine **3d** was isolated as a crystalline product, which determined the absolute configuration to be the (1*R*,2*S*) enantiomer, after analysis of the X-ray diffraction pattern of a single crystal. Lower yields were obtained in the presence of competing Pd(II)-coordinating functionality (isoxazoline in **3h**). The configuration of the product confirmed our calculations for the boat-type transition structure and validated our model for asymmetric induction. Our previous work on γ-methyl C(sp^3^)–H arylation on pyrrolidine-derived substrates failed to generate any of the desired arylated products because the competitive β-hydride elimination pathways dominated the reaction, leading to decomposition of the substrate. However, we were pleased to find that, despite competitive β-hydride elimination, the reaction of a pyrrolidine-derived AMCP gave **3i** with an e.r. > 96:4 in a modest, yet synthetically usable, yield. Similarly, azetidine- and spirocyclic-derived substrates also produced their arylated products (**3j**,**k**) with excellent e.r’s and represent attractive small-molecule fragments of interest in the design of biologically active molecules. A bicyclic amine substrate failed to generate its corresponding product (**3l**), likely due to the hindered nature of the nitrogen lone pair, which prevents an efficient coordination with the Pd(II)-center.

a Reaction at 40 °C. b Reaction at 60 °C. c Reaction with NMP as solvent. d Reaction at 50 °C. e Reaction at 30 °C.

While we did not extensively explore the scope of aminomethyl-cyclopropanes with acyclic nonreacting substituents (**3a**,**m**–**o**), we did find that ester and *N*-carbamyl-azetidine functionality did not adversely affect the reaction and gave products **3m** and **3n** in high e.r. The reaction was able to accommodate Lewis-basic heteroarene functionality, but the product (**3o**) was formed with lower yield and enantio-induction, possibly as a result of competitive coordination which affects the stability of the required transition structure. Substrates containing more hindered tertiary alkylamine motifs were also tolerated by the reaction and produced the corresponding arylated-cyclopropanes with high enantiomeric ratios (**3p** and **3q**). Interestingly, we found that further substitution on the cyclopropane at the same position as the aminomethyl-group gave substrates amenable to the γ-methylene C(sp^3^)–H arylation, although the yield and e.r. of the products **3r**–**t** were lower than their lesser-substituted congeners. While we are not certain of the origins of this reduced enantioselectivity, it seems likely that the addition geminal substituent on the cyclopropane ring would lead to a syn-pentane-like interaction in the corresponding **TS1**, thereby raising its energy such that other transition structures may come into play. This further substitution did, however, allow us to assess several selectivity factors in substrates containing more than one suitably proximal C–H bond.

We prepared a substrate that presented a competing γ-methyl C–H bond in addition to the γ-methylene C–H bond of the cyclopropane. Reaction under the standard conditions produced an approximately 3.5:1 mixture of products in favor of C–H arylation on the cyclopropane ring (**3s**). In spite of the enhanced reactivity of cyclopropane C–H bonds, the selectivity observed over the classically more reactive γ-methyl C–H bonds is surprising. When the reaction was challenged with a substrate displaying a proximal aryl group and the γ-methylene C(sp^3^)–H bond of the cyclopropane, we observed an approximately 2:1 ratio in favor of arylation on the arene (to **4b**); the arylated cyclopropane was produced with an e.r. of 96:4, which provides a modest but usable yield of the highly substituted enantioenriched aminomethyl-cyclopropane (**3t**). Neither primary or secondary aminomethyl cyclopropanes were productive substrates in this reaction.

Following the assessment of the amine motif, the focus shifted toward assessing the scope of the boronic acid component (Chart 1B). It was initially found that arylboronic acids substituted with electron-withdrawing groups delivered lower reactions yields, due to the significant formation of the homocoupled biaryl (see Supporting Information for details). However, better conversion to the desired γ-methylene C(sp^3^)–H bond arylation product was achieved when carrying the reaction at 40 °C for longer reactions times and with *N*,*N*-dimethylacetamide (DMA) as solvent. With this subtle change to the reaction conditions, a variety of aryl groups with substituents at the *meta*- or *para*-positions underwent transfer in good yields: aryl groups containing carbonyls (**3aa**–**ab**), halogens (**3ac**–**ad**), *N*-substituted arenes (**3ae**–**af**), alkoxy ethers (**3ag**, **3aj**), nitro groups (**3ah**), trifluoromethyl (**3ai**), extended aromatic systems (**3ak**), and dioxalane groups (**3al**). A selection of pyridyl-boronic acids were also compatible with the reaction and transferred the Lewis basic heterocycles to the cyclopropane scaffold with excellent e.r’s, albeit in lower yield compared to benzene derivatives (**3am**–**ao**); 3-pyridyl boronic acid, chosen as a representative unsubstituted Lewis basic heteroarene, was unsuccessful in the reaction with homocoupled heteroarene observed as the major product. Unfortunately, arylboronic acids displaying *ortho*-substituents or free amino groups failed to deliver the desired product under these reaction conditions (**3an–ao**). All arylated aminomethyl-cyclopropanes displayed excellent levels of enantioselectivity, suggesting that the boronic acid component is not involved in the enantiodetermining step.

**Scheme 1:**
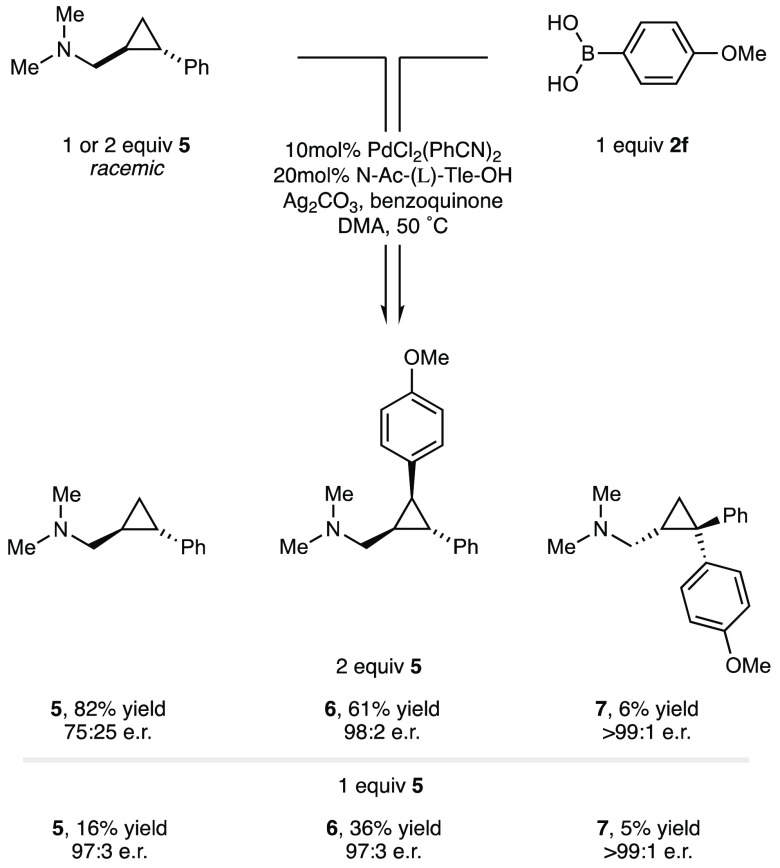
Reaction of Racemic Disubstituted Aminomethyl-cyclopropanes to Form Enantioenriched Trisubstituted Products

With the γ-methylene C(sp^3^)–H bond arylation of AMCPs displaying a broad substrate scope in both components and a good understanding of the transition structures governing the enantioselective C–H cleavage, we questioned whether this transformation would be amenable to kinetic resolution of racemic substituted cyclopropanes.^15^ We chose *trans*-substituted cyclopropane **5** with which to test this potentially useful transformation, as the presence of a substituent on the opposite face to the reacting C–H bond should not affect the amine conformations depicted in **TS1**. Accordingly, reaction of 2.0 equiv of disubstituted cyclopropane **5**, under our standard conditions, delivered a 61% yield of *trans*-diaryl amine **6** with a e.r. of 98:2 (Scheme 1). The formation of **6** was accompanied by a small amount of an isomeric trisubstituted aminomethyl-cyclopropane **7** arising from γ-methine arylation of the (*R,R*)-isomer of aminomethyl-cyclopropane **5** at the benzylic position on the strained ring in >99:1 e.r. The remaining starting aminomethyl-cyclopropane starting material, **5**, was recovered with an e.r. of 75:25. A similar reaction with only 1.0 equiv of amine **5** produced modest yields of the trisubstituted aminomethyl cyclopropane **6** with a 93:7 e.r. and 16% of the starting material (**5**) recovered with an e.r. of 97:3. Unfortunately, the conversion of amine **5** to two different arylated products made the calculation of the selectivity factor for this transformation not possible. Despite this, the “kinetic resolution” can be applied in a practical manner to form enantioenriched differentially *trans*-diarylated trisubstituted aminomethyl-cyclopropanes, compounds that would be difficult to make in a straightforward fashion via contemporary methods.

**Chart 2:**
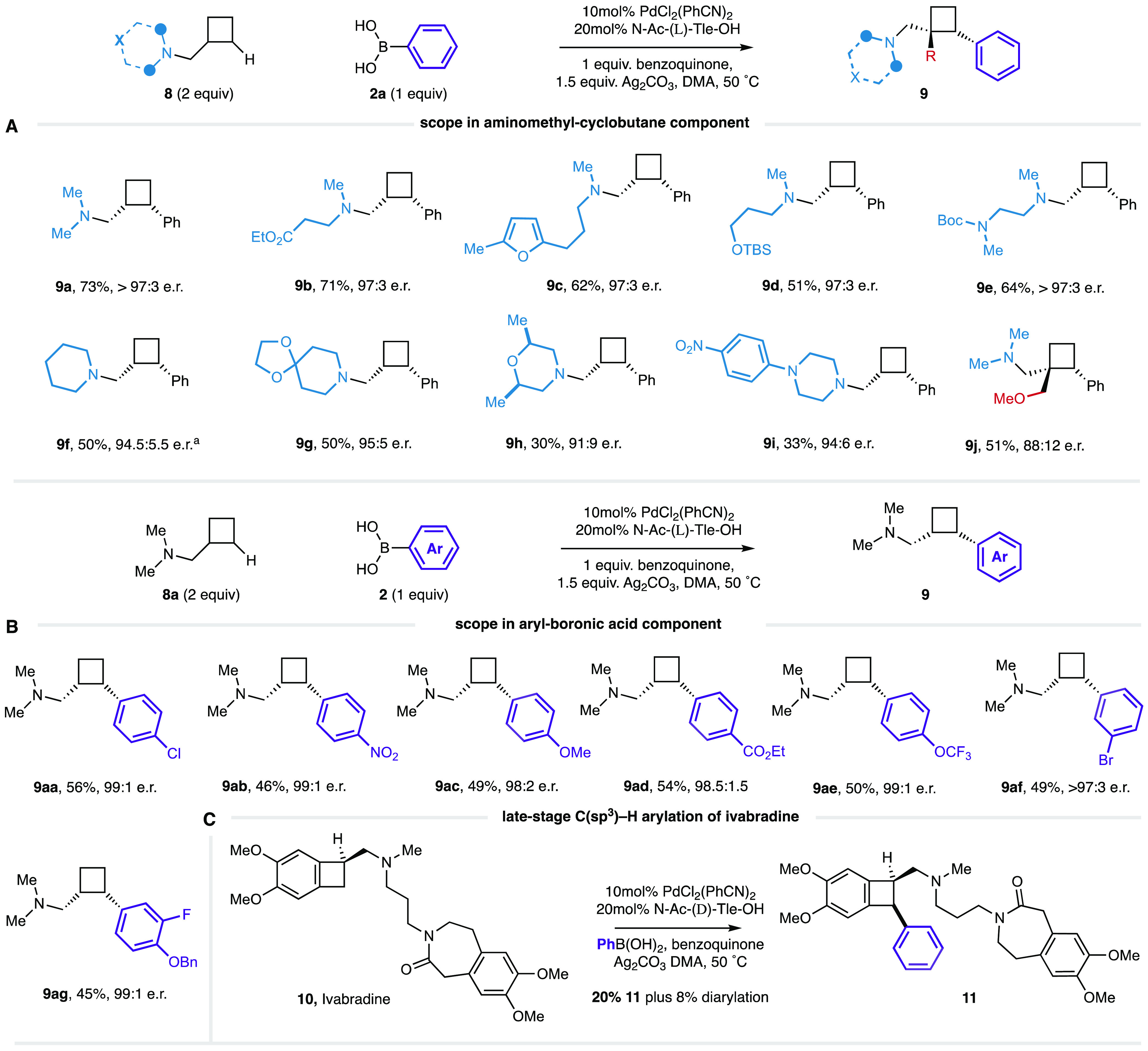
(A, B) Scope of Enantioselective γ-Methylene C(sp^3^)–H Arylation of Aminomethyl-cyclobutanes and (C) Late-Stage Functionalization of Pharmaceutical Agents

Next, we next turned our attention to the development of the, *a priori* more demanding, C–H arylation of AMCBs (**8**). Guided by the studies on γ-C(sp^3^)–H arylation of the cyclopropane series, we found that the same conditions also led to the formation of arylated aminomethyl-cyclobutane **9a** in 63% assay yield. Increasing the reaction temperature to 60 °C, however, provided an optimal 78% assay yield (73% after purification by silica gel chromatography) of **9a** with an e.r. > 97:3 (Chart 2A). In this case, the e.r. was determined by ^1^H NMR analysis after treatment of **9a** with methyl iodide (to make the tetraalkyl ammnonium salt) and counterion exchange with a chiral hexa-coordinate phosphate salt (see Supporting Information for details).^16^

a Reaction with NMP as solvent.

In exploring the scope of the cyclobutane arylation, we found that the amine motifs containing common functional groups like esters (**9b**), electron-rich heteroarenes (**9c**), protected alcohols (**9d**), and amines (**9e**) all delivered good yields of their corresponding arylated aminomethyl-cyclobutanes with excellent e.r’s. A range of substrates containing saturated heterocyclic tertiary alkylamines piperidine (**9f**–**g**), morpholines (**9h**), and piperazine (**9i**) also worked well, although the yields and e.r’s were slightly diminished compared to the corresponding cyclopropane systems (Chart 2A). The C–H bonds in cyclobutanes are less reactive than cyclopropanes as a result of them having less sp^2^ character, which likely explains the lower yields.^17^ Similar to that observed with cyclopropanes, the presence of a substituent in a geminal position to the directing amine can still deliver the expected arylation (**9j**), but a slightly lower e.r. of 88.5:11.5 was observed. A selection of substituted arylboronic acids (**2**) worked well in the reaction to form aminomethyl-cyclobutane products (**9aa**–**ag**) displaying a range of useful functional groups (Chart 2B). Interestingly, we found that the use of a ligand based on phenylalanine generally gave better yields. Enantiomeric ratios were routinely high although the yields were lower than those obtained for the corresponding cyclopropane series.

To test whether the reaction was competent on more complex substrates, we submitted the pharmaceutical agent, Ivabradine,^18^ to the reaction conditions (Chart 2C). To complement the actual enantiomer of Ivabradine, the d-form of the amino acid ligand is used in combination with the otherwise standard catalytic reaction conditions to provide a modest, but synthetically usable yield of the phenylated product **11** as a single diastereoisomer. This late-stage functionalization tactic potentially provides access to modular arylated variants of Ivabradine that would be difficult to access using other methods if required.

**Figure 4:**
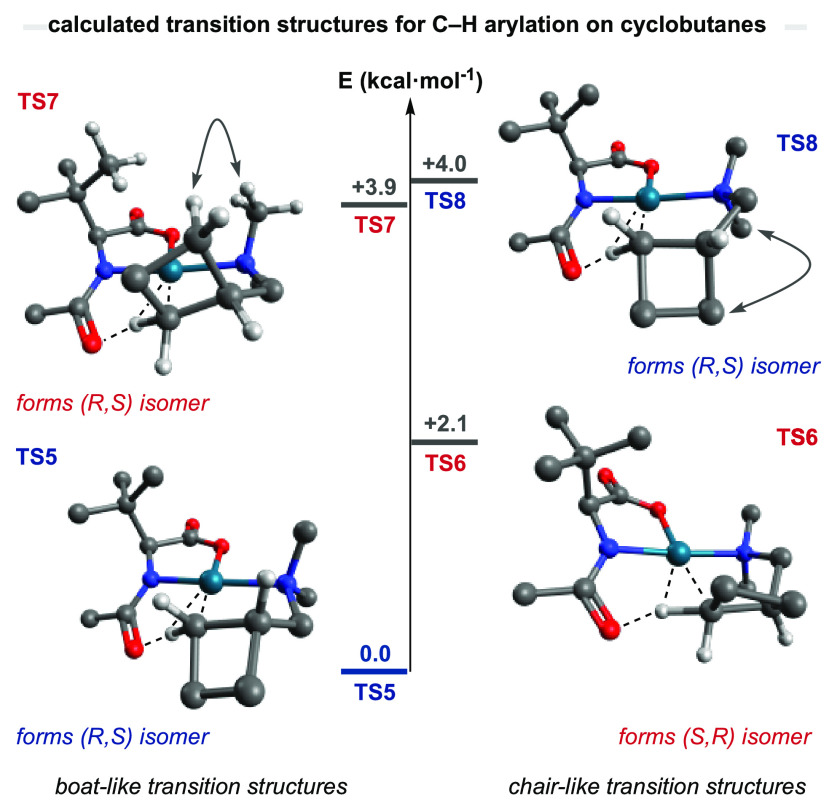
Computational analysis of the enantiodetermining C–H cleavage in cyclobutane rings. Basis set B3LyP-D3(BJ)/[6-311+G(2d,p)/ SDD(Pd)].

Arylated aminomethyl-cyclobutane **9ad** provided a single crystal in its hydrochloride salt, from which we were able to determine its absolute configuration through analysis of the X-ray diffraction. Accordingly, this enabled us to investigate whether our model for the cyclopropane reaction was consistent with the four-membered ring system. Computational calculations determined that the nonplanar AMCBs have access to a few more diastereomeric transition states than the rigid cyclopropane ring (Figure 4). Although a number of transition structures could be identified, only the most relevant pairs are detailed here, but a more detailed analysis can be found in the Supporting Information. The lowest transition structure was found to be **TS5**, where a twist-boat conformation (observed between palladium, nitrogen, the 3-carbon backbone, and the cleaving hydrogen atom) minimizes the eclipsing interactions within the substituted cyclobutane as a result of the puckered conformation of the four membered ring. The lack of steric interactions contrasts with **TS7**, where a H-to-H distance of 2.03 Å is observed between the methylene group of the cyclobutene ring and the *N*-methyl substituent, resulting in an energy difference of 3.9 kcal·mol^–1^. Interestingly, two other transition states (**TS6** and **TS8**) where found to proceed through a chair-like conformation, resembling the ones predicted when C–H activation is attempted on linear *N*-isobutyl alkylamines (Figure 2B). When the system loses its strained character, the chair-like transition states recover their predominant stability among other conformations. **TS8** exhibits a 1,3-diaxial-type interaction between the cyclobutane and the *N*-methyl substituent, which makes it significantly higher in energy. **TS6** presents no detrimental steric interactions, and the reason for its 2.1 kcal·mol^–1^ energy difference compared to **TS5** lies in the presence of torsional strain within the backbone of the substrate. It is important to emphasize that the most stable transition states within each diastereomeric complex (**TS5** and **TS6**) are devoid of destabilizing steric interactions with the ligand and the predicted enantiomeric ratio relies on a much more subtle torsional strain within the aminomethyl-cyclobutane backbone.

## Conclusion

In summary, we have developed a method for the selective C–H arylation of strained cycloalkanes displaying an appendant tertiary amine functionality. With the aid of an inexpensive chiral ligand, it was possible to synthesize a wide range of arylated cycloalkane products all displaying exclusive *cis* diastereoselectivity and enantiomeric ratios frequently >95:5. Common saturated N-heterocycles, such as piperidines, piperazines, morpholines, pyrrolidines, and azetidines as well as acyclic tertiary alkylamines substituents, were amenable to this γ-methylene C(sp^3^)–H arylation strategy. Computational studies were able to accurately predict the observed enantioselectivity for both types of ring-strained systems, and the origin of enantioselectivity relied on the restricted geometry of the internal amidate base, which limits the different conformations accessible to the reacting substituent through which C–H activation can be accessed. We believe that this operationally simple method will be of interest to those interested into the synthesis of conformationally defined biologically active functional cycloalkane scaffolds in industrial and academic institutions.

## Supporting Information Available

All experimental procedures, extended mechanistic discussion, computational calculations, and compound characterization (including ^1^H and ^13^C NMR spectra, IR, HRMS, and X-ray data) are available in the document (PDF)

## Accession Codes

CCDC 2114166–2114167 contain the supplementary crystallographic data for this paper. These data can be obtained free of charge via www.ccdc.cam.ac.uk/data_request/cif, or by emailing data_request@ccdc.cam.ac.uk, or by contacting The Cambridge Crystallographic Data Centre, 12 Union Road, Cambridge CB2 1EZ, UK; fax: +44 1223 336033.
