# Supplementary material for: Pd(II)-Catalyzed Enantioselective C(sp3)–H Arylation of Cyclopropanes and Cyclobutanes Guided by Tertiary Alkylamines
Source: J Am Chem Soc. 2022 Feb 25;144(9):3939–48. doi: 10.1021/jacs.1c11921 (PMC9097487; doi:10.1021/jacs.1c11921)
Supplement: Supplementary file 1 — ja1c11921_si_001.pdf [file ja1c11921_si_001.pdf]

Supplementary Information

Pd(II)-catalyzed enantioselective C(sp^3)–H arylation of cyclopropanes and cyclobutanes guided by tertiary alkylamines

Jesus Rodrigalvarez, Luke A. Reeve, Javier Miró, Matthew J. Gaunt

Correspondence to: mjg32@cam.ac.uk

1. General Information 1
2. Preparation of amine substrates 3
3. Optimization of aryl boronic acid scope 17
4. C(sp^3)–H arylated products 18
5. HPLC, GC, and NMR traces 44
6. DFT calculations 99
7. NMR spectra 112

1. General Information

Solvents and reagents: Et_2O and THF were obtained as anhydrous solvents from distillation over $LiAlH_4$. MeCN, DCM, hexane and toluene were obtained as anhydrous solvents from distillation over from CaH_2. NMP (99.5%), DMA (99.5%) and DMF (99.8%) were purchased from Acros Organics (extra dry, AcroSeal®) and used without further purification. Other solvents used were purchased anhydrous and used without further purification unless otherwise stated. $Pd(OAc)_2$ (Pd 45.9–48.4% needles), Ag_2CO_3 (99%) and phenyl boronic acid (98+%) were purchased from Alfa Aesar and used without further purification. $Pd(PhCN)_2Cl_2$ (95%) was purchased from Sigma Aldrich and used without further purification. 1,4-Benzoquinone was purchased from Sigma Aldrich and purified by recrystallisation from petroleum ether (40–60) before use.

Chromatography: Reactions were monitored by thin layer chromatography (TLC) using pre-coated Merck glass-backed silica gel plates (Kieselgel 60 F254 0.2 mm). Visualisation was performed using ultraviolet light (λ_{max} = 254 nm) and/or chemical staining with basic potassium permanganate solution as appropriate. Alternatively, reactions were also monitored by gas chromatography (GC) using a Shimadzu QP2010-SE GC fitted with a BPX5 column (10 m, 0.1 mm, 0.1 µm film) for FID analysis, or a SHIM-5MS column (30 m, 0.25 mm, 0.25 µm film) for MS analysis. Chiral analysis was performed on a Shimadzu XR high-performance liquid chromatography (HPLC) instrument fitted with a CHIRALPAK® AD-H column, or a Shimadzu QP2010-SE gas chromatography (GC) fitted with a Astec Chiraldex™ B-DM (20 m, 0.25 mm, 0.12 µm film) for FID analysis. Flash column chromatography was performed on Merck Geduran Si 60 [40-63 µm] under a positive pressure of air or by using a Teledyne CombiFlash NextGen 300+.

Characterisation: Nuclear magnetic resonance (NMR) spectra were recorded at room temperature on a 400 MHz Bruker Avance III HD spectrometer, a 500 MHz Bruker Avance III HD Smart Probe spectrometer, or a 700 MHz Bruker Avance II+ spectrometer. Chemical shifts (δ) were reported in ppm and quoted to the nearest 0.01 ppm relative to the residual protons in $CDCl_3$ (7.26 ppm for ^{1}H-NMR, 77.16 ppm for ^{13}C-NMR) or DMSO-d_6 (2.05 ppm for ^{1}H-NMR, 39.52 ppm for ^{13}C-NMR). Coupling constants (*J*) were quoted to the nearest 0.1 Hz and multiplicity reported according to the following convention: s = singlet, d = doublet, t = triplet, q = quartet, p = quintet, hex = sextet, hept = septet, oct = octet, m = multiplet, br = broad and associated combinations, e.g. dd = doublet of doublets. Where coincident coupling constants have been observed, the apparent (app) multiplicity of the proton resonance has been reported. Data were reported as follows: chemical shift (multiplicity, coupling constants, number of protons and molecular assignment). DEPT135 and 2-dimensional experiments (COSY, HMBC, HSQC and NOESY) were used to support assignments when appropriate, but were not included herein.

Infrared spectra (FT-IR) were recorded on a PerkinElmer FT-IR spectrometer fitted with an ATR sampling accessory or a Thermo Fisher Nicolet Summit PRO FT-IR spectrometer and samples were analysed as neat oils or solids. Absorptions were reported in wavenumbers (cm^{-1}) and only the most relevant peaks were reported.

High-resolution mass spectra (HRMS) were measured on a Thermo Scientific LTQ Orbitrap XL at the EPSRC Mass Spectrometry Service from University of Swansea, or on a Micromass Q-TOF spectrometer or Waters XEVO GII-S Q-TOF spectrometer at the Department of Chemistry from University of Cambridge.

Melting points (mp) were recorded using a Gallenkamp melting point apparatus and are uncorrected.

Optical rotations were measured on a Perkin Elmer Model 343 polarimeter using a Na lamp (λ 589 nm, D-line).

X-Ray crystallography was performed on a Nonius Kappa CCD or a Bruker D8-Quest Photon-100 at the University of Cambridge Chemistry X-ray laboratory by Dr. Andrew Bond.

2. Preparation of amine substrates

General procedure A: Reductive amination

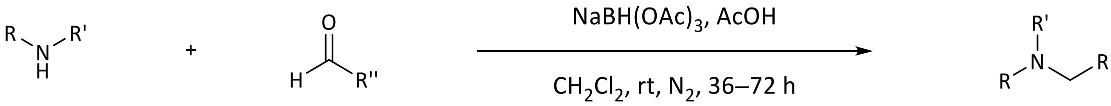

To a solution of amine (1 equiv) in DCM [0.2 – 0.4 M] at 0 °C under N_2, was added the desired aldehyde (1 – 2 equiv) and AcOH (1 equiv). $NaBH(OAc)_3$ (1.2 – 2 equiv) was added portionwise over 15 min while stirring. The resultant mixture was purged briefly with N_2, warmed to rt and stirred for 36–72 h. The reaction was quenched with aq NaOH (2.5 M) and the solution was left stirring until no further effervescence was observed. The phases were separated and the aqueous layer was extracted twice with DCM. The combined organic layers were dried over $MgSO_4$, filtered and concentrated *in vacuo*. The crude product was purified by distillation or column chromatography.

General procedure B: Eschweiler-Clarke methylation

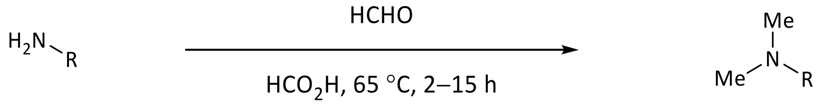

To a solution of formaldehyde (37% in H_2O, 6 equiv) and amine (1 equiv), formic acid was added dropwise while stirring to reach a 1 M solution. The flask was fitted with a condenser and the reaction mixture was heated to 65 °C for 2–15 h. After cooling to 0 °C, the reaction mixture was basified with aq NaOH (2.5 M). The resultant tertiary amine was separated from the aqueous layer as a supernatant, dried over $MgSO_4$ and filtered. The crude product was purified by distillation or column chromatography.

General procedure C: Amine acylation

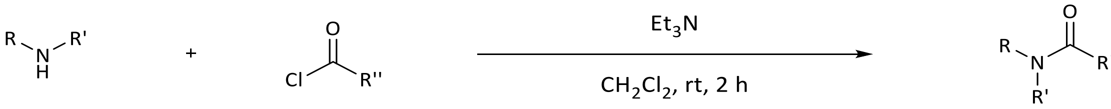

To a solution of amine (1 equiv), Et_3N (1.2 equiv) in DCM [0.5 M] at 0 °C, was added the desired acyl chloride (1.2 equiv). The resultant mixture was warmed to rt and stirred for 2 h. The reaction was quenched with aq NH_4Cl (sat). The organic layer was washed twice with aq HCl (0.5 M), twice with aq NaOH (0.5 M), dried over $MgSO_4$, filtered and concentrated *in vacuo*. The crude product was typically used without further purification.

General procedure D: Amide coupling

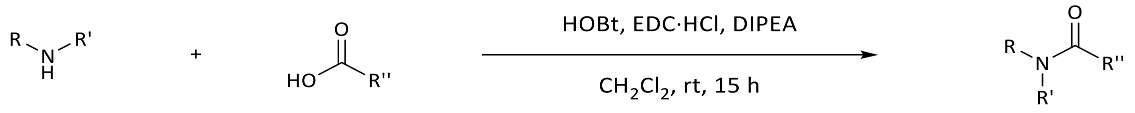

To a solution of amine (1 – 1.2 equiv), carboxylic acid (1 – 1.2 equiv) and DIPEA (1.5 equiv) in DCM [0.3 – 0.5 M] was added HOBt·hydrate (1.5 equiv) and EDC·HCl (1.2 equiv). The resultant mixture was warmed to rt and stirred for 15 h. The reaction was quenched with aq NaOH (2.5 M) and the resultant layers were separated. The organic layer was washed once with aq NaOH (0.5 M), twice with aq HCl (0.5 M), dried over $MgSO_4$, filtered and concentrated *in vacuo*. The crude product was typically used without further purification.

General procedure E: Amide reduction

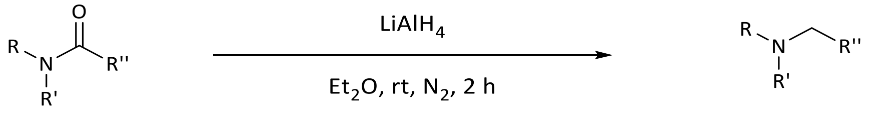

To a solution of $LiAlH_4$ (2 equiv) in Et_2O [1 M] under N_2 at 0 °C, was added dropwise the desired amide (1 equiv) in Et_2O [1 M]. The resultant mixture was warmed to rt and stirred for 2 h. After this time, the reaction was cooled to 0 °C and further Et_2O was added. The reaction was quenched with a dropwise addition of H_2O (2 equiv), NaOH (10%) (2 equiv), and H_2O (6 equiv). The resultant solution was dried over $MgSO_4$, filtered and concentrated *in vacuo*. The crude product was purified by distillation or column chromatography.

General procedure F: Amine alkylation

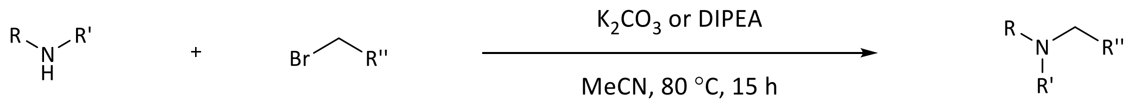

To a solution of amine (1 – 2.5 equiv) and K_2CO_3 (1.5 equiv) or DIPEA (1.5 equiv) in MeCN [0.5 M] was added the desired alkyl bromide (1.0 – 1.5 equiv). The resultant mixture was heated to reflux for 15 h. After this time, volatiles were evaporated *in vacuo*. DCM was added to the reaction crude and the organic layer was washed twice with aq NaOH (2.5 M), dried over $MgSO_4$, filtered and concentrated *in vacuo*. The crude product was typically purified by column chromatography.

1-Cyclopropyl-*N,N*-dimethylmethanamine (1a)

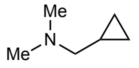

1a

General procedure B was applied to cyclopropylmethanamine (8.70 mL, 10.0 mmol) with formaldehyde (37%, 45.0 mL, 600 mmol) in formic acid (50.0 mL) to provide by distillation at 100 °C under 1 bar the title compound as a colourless oil (6.54 g, 66.0 mmol, 66% yield).

IR ν_{max}/cm^{-1} (thin film) 3079, 2944, 2814, 2763, 1464, 1263, 1183, 1029; **^{1}H NMR** (400 MHz, $CDCl_3$) δ (ppm) 2.28 (s, 6H), 2.16 (app d, *J* = 6.7 Hz, 2H), 0.94 – 0.79 (m, 1H), 0.58 – 0.45 (m, 2H), 0.11 (app q, *J* = 4.7 Hz, 2H); **^{13}C NMR** (101 MHz, $CDCl_3$) δ (ppm) 64.7, 45.5, 9.2, 3.8; **HMRS-ESI** (m/z): found $[M+H]^+$ 100.1124, $C_6H_{14}N$ requires 100.1121.

1-(Cyclopropylmethyl)piperidine (1b)

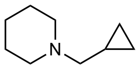

1b

General procedure D was applied to piperidine (1.98 mL, 20.0 mmol) with cyclopropane-carboxylic acid (1.67 mL, 21.0 mmol), DIPEA (5.24 mL, 30.0 mmol), HOBt hydrate (4.00 g, 30.0 mmol) and EDC·HCl (4.22 g, 22.0 mmol) in DCM (40 mL). The resultant crude amide was used without further purification in General procedure E with $LiAlH_4$ (1.52 g, 40.0 mmol) in Et_2O (60 mL). Purification by distillation at 100 °C under 60 mbar provided the title compound as a colourless oil (1.73 g, 12.4 mmol, 62% yield).

IR ν_{max}/cm^{-1} (thin film) 2931, 2769, 1442, 1377, 1332, 1299, 1149, 1018; **^{1}H NMR** (600 MHz, $CDCl_3$) δ (ppm) 2.46 (br s, 4H), 2.22 (d, *J* = 6.5 Hz, 2H), 1.61 (app p, *J* = 5.7 Hz, 4H), 1.44 (br s, 2H), 0.93 – 0.83 (m, 1H), 0.53 – 0.47 (m, 2H), 0.12 – 0.06 (m, 2H); **^{13}C NMR** (151 MHz, $CDCl_3$) δ (ppm) 64.6, 54.6, 26.0, 24.5, 8.4, 4.0; **HMRS-ESI** (m/z): found $[M+H]^+$ 140.1432, $C_9H_{18}N$ requires 140.1434.

***tert*-Butyl (1-(cyclopropylmethyl)piperidin-4-yl)carbamate (1c)**

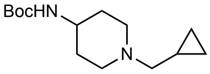

1c

General procedure A was applied to *tert*-butyl piperidin-4-ylcarbamate (3.00 g, 15.0 mmol) with cyclopropanecarboxaldehyde (1.50 mL, 20.0 mmol), AcOH (860 µL, 15.0 mmol) and $NaBH(OAc)_3$ (4.24 g, 20.0 mmol) in DCM (50 mL) to provide by column chromatography (DCM to 5% $NH_{3(MeOH;\ 2M)}$) the title compound as a colourless oil (1.31 g, 7.80 mmol, 78% yield).

mp (°C) 88–90; **IR** ν_{max}/cm^{-1} (thin film) 3170, 2943, 2783, 1702, 1535, 1274, 1242, 1170; **^{1}H NMR** (600 MHz, $CDCl_3$) δ (ppm) 4.44 (br s, 1H), 3.47 (app br s, 1H), 2.98 (app br d, *J* = 8.3 Hz, 2H), 2.24 (d, *J* = 6.5 Hz, 2H), 2.08 (app br t, *J* = 10.9 Hz, 2H), 1.95 (app br d, *J* = 12.1 Hz, 2H), 1.51 – 1.41 (m, 11H), 0.90 – 0.81 (m, 1H), 0.54 – 0.49 (m, 2H), 0.10 (app q, *J* = 4.7 Hz, 2H); **^{13}C NMR** (151 MHz, $CDCl_3$) δ (ppm) 155.2, 79.1, 63.8, 52.4, 47.8, 32.6, 28.4, 8.5, 3.9; **HMRS-ESI** (m/z): found $[M+H]^+$ 255.2062, $C_{14}H_{27}N_2O_2$ requires 255.2073.

1-(Cyclopropylmethyl)-4-tosylpiperazine (1d)

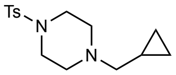

1d

General procedure A was applied to 4-tosylpiperazine (3.61 g, 15.0 mmol) with cyclopropanecarboxaldehyde (1.49 mL, 20.0 mmol), AcOH (860 µL, 15.0 mmol) and

$NaBH(OAc)_3$ (4.24 g, 20.0 mmol) in DCM (50 mL) to provide by trituration in a mixture of PE:Et_2O (1:1) the title compound as a white solid (3.26 g, 11.1 mmol, 74% yield).

mp (°C) 106–108; **IR** ν_{max}/cm^{-1} (thin film) 3074, 2776, 1456, 1344, 1325, 1161, 952; **^{1}H NMR** (600 MHz, $CDCl_3$) δ (ppm) 7.64 (d, *J* = 8.0 Hz, 2H), 7.33 (d, *J* = 8.0 Hz, 2H), 3.04 (br s, 4H), 2.61 (br s, 4H), 2.43 (s, 3H), 2.24 (d, *J* = 6.6 Hz, 2H), 0.82 – 0.73 (m, 1H), 0.54 – 0.46 (m, 2H), 0.09 (q, *J* = 4.7 Hz, 2H); **^{13}C NMR** (151 MHz, $CDCl_3$) δ (ppm) 143.7, 131.8, 129.6, 128.0, 63.3, 52.2, 46.0, 21.6, 8.3, 3.9; **HMRS-ESI** (m/z): found $[M+H]^+$ 295.1466, $C_{15}H_{23}N_2O_2S$ requires 295.1480.

(2*R*,6*S*)-4-(cyclopropylmethyl)-2,6-dimethylmorpholine (1e)

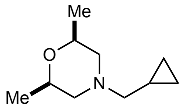

1e

General procedure A was applied to (2*R*,6*S*)-2,6-dimethylmorpholine (1.27 mL, 10.0 mmol) with cyclopropanecarboxaldehyde (1.14 mL, 15.0 mmol), AcOH (570 µL, 10.0 mmol) and $NaBH(OAc)_3$ (4.37 g, 20.0 mmol) in DCM (50 mL). The title compound was obtained without further purification (3.69 g, 14.5 mmol, 97% yield).

IR ν_{max}/cm^{-1} (thin film) 2971, 2867, 2778, 1455, 1321, 1143, 1073; **^{1}H NMR** (600 MHz, $CDCl_3$) δ (ppm) 3.79 – 3.65 (m, 2H), 2.89 (d, *J* = 11.6 Hz, 2H), 2.23 (d, *J* = 6.5 Hz, 2H), 1.72 (t, *J* = 10.8 Hz, 2H), 1.17 (d, *J* = 6.3 Hz, 6H), 0.94 – 0.78 (m, 1H), 0.61 – 0.45 (m, 2H), 0.16 – 0.04 (m, 2H); **^{13}C NMR** (151 MHz, $CDCl_3$) δ (ppm) 71.6, 63.8, 59.7, 19.3, 8.1, 3.9; **HMRS-ESI** (m/z): found $[M+H]^+$ 170.1547, $C_{10}H_{20}NO$ requires 170.1545.

1'-(Cyclopropylmethyl)spiro[chromane-2,4'-piperidin]-4-one (1f)

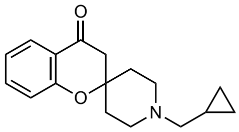

1f

General procedure F was applied to spiro[chromane-2,4'-piperidin]-4-one (1.01 g, 4.00 mmol) with (bromomethyl)cyclopropane (430 µL, 4.40 mmol) and K_2CO_3 (880 mg, 6.0 mmol) in MeCN (20 mL) to provide by column chromatography (DCM to 5% $NH_{3(MeOH;\ 2M)}$) the title compound as a yellow oil (750 mg, 2.76 mmol, 69% yield).

IR ν_{max}/cm^{-1} (thin film) 3076, 2919, 2817, 2780, 1688, 1606, 1461, 1298, 1228, 1114; **^{1}H NMR** (400 MHz, $CDCl_3$) δ (ppm) 7.87 (d, *J* = 8.0 Hz, 1H), 7.49 (t, *J* = 7.7 Hz, 1H), 7.13 – 6.91 (m, 2H), 2.85 – 2.75 (m, 2H), 2.73 (s, 2H), 2.46 (br t, *J* = 10.7 Hz, 2H), 2.31 (d, *J* = 6.5 Hz, 2H), 2.08 (br d, *J* = 12.6 Hz, 2H), 1.85 – 1.74 (m, 2H), 0.94 – 0.82 (m, 1H), 0.53 (q, *J* = 5.1 Hz, 2H), 0.13 (q, *J* = 5.1 Hz, 2H); **^{13}C NMR** (101 MHz, $CDCl_3$) δ (ppm) 192.1, 159.2, 136.2, 126.5, 120.9, 120.8, 118.4, 77.9, 63.7, 48.8, 48.0, 34.3, 8.6, 4.0; **HMRS-ESI** (m/z): found $[M+H]^+$ 272.1640, $C_{17}H_{22}NO_2$ requires 272.1651.

4-(4-Chlorophenyl)-1-(cyclopropylmethyl)piperidin-4-ol (1g)

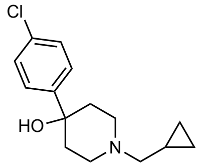

1g

General procedure A was applied to 4-(4-chlorophenyl)piperidin-4-ol (2.12 g, 10.0 mmol) with cyclopropanecarboxaldehyde (1.12 mL, 15.0 mmol), AcOH (572 µL, 10.0 mmol) and $NaBH(OAc)_3$ (4.24 g, 20.0 mmol) in DCM (50 mL) to provide by trituration in a mixture of PE:DCM (9:1) the title compound as a white solid (2.03 g, 7.64 mmol, 76% yield).

mp (°C) 94–96; **IR** ν_{max}/cm^{-1} (thin film) 3072, 2918, 2835, 1482, 1378, 1097, 996; **^{1}H NMR** (600 MHz, $CDCl_3$) δ (ppm) 7.51 – 7.41 (m, 2H), 7.36 – 7.29 (m, 2H), 2.98 (app br d, *J* = 11.4 Hz, 2H), 2.46 (td, *J* = 12.2, 2.3 Hz, 2H), 2.34 (d, *J* = 6.5 Hz, 2H), 2.16 (td + br s, *J* = 13.3, 4.5 Hz, 3H), 1.74 (dd, *J* = 14.1, 2.3 Hz, 2H), 0.96 – 0.85 (m, 1H), 0.59 – 0.51 (m, 2H), 0.14 (q, *J* = 4.7 Hz, 2H); **^{13}C NMR** (151 MHz, $CDCl_3$) δ (ppm) 147.0, 132.7, 128.4, 126.1, 71.0, 63.9, 49.4, 38.4, 8.4, 4.0; **HMRS-ESI** (m/z): found $[M+H]^+$ 266.1301, $C_{15}H_{21}NOCl$ requires 266.1312.

3-(1-(Cyclopropylmethyl)piperidin-4-yl)-6-fluorobenzo[*d*]isoxazole (1h)

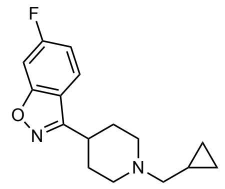

1h

General procedure F was applied to 6-fluoro-3-(piperidin-4-yl)benzo[*d*]isoxazole hydrochloride (1.28 g, 5.00 mmol) with (bromomethyl)cyclopropane (534 µL, 5.50 mmol) and DIPEA (2.10 mL, 12.0 mmol) in MeCN (10 mL) to provide by column chromatography (DCM to 4% $NH_{3(MeOH;\ 2M)}$) the title compound as a white solid (846 mg, 3.08 mmol, 62% yield).

mp (°C) 82–84; **IR** ν_{max}/cm^{-1} (thin film) 2962, 1609, 1414, 1270, 1232, 1120, 954, 829; **^{1}H NMR** (700 MHz, $CDCl_3$) δ (ppm) 7.74 (dd, *J* = 8.7, 5.1 Hz, 1H), 7.25 (dd, *J* = 8.5, 1.8 Hz, 1H), 7.06 (td, *J* = 8.8, 2.0 Hz, 1H), 3.23 (app br d, *J* = 11.3 Hz, 2H), 3.09 (tt, *J* = 11.2, 4.0 Hz, 1H), 2.34 (d, *J* = 6.5 Hz, 2H), 2.23 – 2.18 (m, 2H), 2.14 (ddd, *J* = 14.7, 12.1, 3.1 Hz, 2H), 2.09 (app br d, *J* = 10.7 Hz, 2H), 0.97 – 0.90 (m, 1H), 0.59 – 0.53 (m, 2H), 0.17 – 0.12 (m, 2H); **^{13}C NMR** (176 MHz, $CDCl_3$) δ (ppm) 164.1 (d, *J* = 250.5 Hz), 163.9 (d, *J* = 13.6 Hz), 161.2, 122.7 (d, *J* = 11.1 Hz), 117.3 (d, *J* = 1.3 Hz), 112.3 (d, *J* = 25.3 Hz), 97.4 (d, *J* = 26.7 Hz), 64.1, 53.5, 34.7, 30.5, 8.4, 4.0; **^{19}F NMR** (377 MHz, $CDCl_3$) δ (ppm) –109.8 (s); **HMRS-ESI** (m/z): found $[M+H]^+$ 275.1563, $C_{16}H_{20}N_2OF$ requires 275.1560.

1-(Cyclopropylmethyl)pyrrolidine (1i)

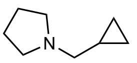

1i

General procedure C was applied to pyrrolidine (4.05 mL, 48.0 mmol) with cyclopropanecarbonyl chloride (3.63 mL, 40.0 mmol) and Et_3N (6.75 mL, 48.0 mmol) in DCM (100 mL). The crude product was used without further purification in General procedure E with $LiAlH_4$ (3.03 g, 80.0 mmol) in Et_2O (80 mL). Purification by distillation at 90 °C under 80 mbar provided the title compound as a colourless oil (2.84 g, 22.7 mmol, 57% yield).

IR ν_{max}/cm^{-1} (thin film) 3076, 2964, 2773, 1346, 1147, 1018; **^{1}H NMR** (600 MHz, $CDCl_3$) δ (ppm) 2.61 – 2.52 (m, 4H), 2.33 (d, *J* = 6.7 Hz, 2H), 1.88 – 1.74 (m, 4H), 1.01 – 0.85 (m, 1H), 0.57 – 0.44 (m, 2H), 0.14 (app q, *J* = 5.0 Hz, 2H); **^{13}C NMR** (151 MHz, $CDCl_3$) δ (ppm) 61.4, 54.4, 23.4, 10.2, 3.8; **HMRS-ESI** (m/z): found $[M+H]^+$ 126.1273, $C_8H_{16}N$ requires 126.1283.

6-(Cyclopropylmethyl)-2-oxa-6-azaspiro[3.3]heptane (1k)

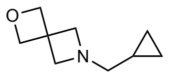

1k

To a solution of 2-oxa-6-azaspiro[3.3]heptane oxalate (2:1) (2.16 g, 15.0 mmol) in DCM (50 mL) at 0 °C under N_2 was added cyclopropanecarboxaldehyde (1.10 mL, 15.0 mmol). $NaBH(OAc)_3$ (4.25 g, 20.0 mmol) was added portionwise over 15 min while stirring. The resultant mixture was purged briefly with N_2, warmed to rt and stirred for 36 h. The reaction was quenched with aq NaOH (2.5 M, 50 mL) and the solution was left stirring until no further effervescence was observed. The phases were separated and the aqueous layer was extracted with further DCM (50 mL). The combined organic layers were extracted twice with 1M HCl (2 x 30 mL), the combined aqueous solution were basified with NaOH (s) until a basic pH > 12 was reached, and it was extracted twice with DCM (2 x 30 mL). The resultant new organic layer was dried over $MgSO_4$, filtered and concentrated *in vacuo* to provide the title compound without further purification (1.24 g, 8.10 mmol, 54% yield).

IR ν_{max}/cm^{-1} (thin film) 2924, 2861, 2806, 1248, 971, 830; **^{1}H NMR** (600 MHz, $CDCl_3$) δ (ppm) 4.74 (s, 4H), 3.37 (s, 4H), 2.23 (d, *J* = 6.8 Hz, 2H), 0.78 – 0.70 (m, 1H), 0.47 – 0.42 (m, 2H), 0.08 (app q, *J* = 4.6 Hz, 2H); **^{13}C NMR** (151 MHz, $CDCl_3$) δ (ppm) 81.4, 64.2, 64.0, 39.5, 9.0, 2.8; **HMRS-ESI** (m/z): found $[M+H]^+$ 154.1226, $C_9H_{16}NO$ requires 154.1232.

(1*R*,5*S*)-3-(cyclopropylmethyl)-8-oxa-3-azabicyclo[3.2.1]octane (1l)

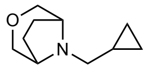

1l

To a solution of (1*R*,5*S*)-8-oxa-3-azabicyclo[3.2.1]octane hydrochloride (1.50 g, 10.0 mmol) and cyclopropanecarboxaldehyde (1.12 mL, 15.0 mmol) in DCM (40 mL) at 0 °C under N_2 was added $NaBH(OAc)_3$ (3.18 g, 15.0 mmol) portionwise. The resultant mixture was stirred at rt for 48 h. After this time, the reaction was quenched with aq NaOH (2.5 M, 10 mL) and concentrated *in vacuo*. Et_2O (50 mL) was added and the organic layer was washed with aq NaOH (3 x 50 mL, 0.25 M), dried over $MgSO_4$, filtered and concentrated *in vacuo*. Purification by distillation at 110 °C under 15 mbar provided the title compound as a colourless oil (775 mg, 4.63 mmol, 46% yield).

IR ν_{max}/cm^{-1} (thin film) 2949, 2804, 1457, 1280, 1163, 1142, 1005; **^{1}H NMR** (600 MHz, $CDCl_3$) δ (ppm) 4.34 – 4.24 (m, 2H), 2.71 (d, *J* = 11.2 Hz, 2H), 2.31 (d, *J* = 11.2 Hz, 2H), 2.19 (d, *J* = 6.5 Hz, 2H), 2.01 – 1.92 (m, 2H), 1.90 – 1.81 (m, 2H), 0.85 – 0.75 (m, 1H), 0.47 (q, *J* = 5.1 Hz, 2H), 0.07 (q, *J* = 5.1 Hz, 2H); **^{13}C NMR** (151 MHz, $CDCl_3$) δ (ppm) 74.8, 63.1, 58.8, 28.6, 8.3, 3.7; **HMRS-ESI** (m/z): found $[M+H]^+$ 168.1380, $C_{10}H_{18}NO$ requires 168.1388.

Ethyl 3-((cyclopropylmethyl)(methyl)amino)propanoate (1m)

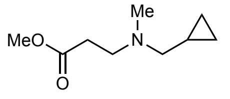

1m

To a solution of cyclopropylmethanamine (5.20 mL, 60.0 mmol) in EtOH (40 mL) at 0 °C was added ethyl acrylate (3.26 mL, 30.0 mmol) in EtOH (10 mL). The resultant mixture was warmed to rt and stirred for 15 h. After this time, the reaction was concentrated *in vacuo* and the crude product was used without further purification in General procedure B with formaldehyde

(37%, 6.75 mL, 90.0 mmol) in formic acid (7.0 mL). The title compound was obtained without further purification (4.35 g, 23.5 mmol, 78% yield).

IR ν_{max}/cm^{-1} (thin film) 2981, 2769, 1733, 1464, 1369, 1180, 1031; ^{1}**H NMR** (400 MHz, $CDCl_3$) δ (ppm) 4.15 (q, *J* = 7.1 Hz, 2H), 2.78 (t, *J* = 7.4 Hz, 2H), 2.49 (t, *J* = 7.4 Hz, 2H), 2.32 (s, 3H), 2.28 (d, *J* = 6.6 Hz, 2H), 1.27 (t, *J* = 7.1 Hz, 3H), 0.95 – 0.78 (m, 1H), 0.57 – 0.47 (m, 2H), 0.12 (q, *J* = 4.7 Hz, 2H); ^{13}**C NMR** (101 MHz, $CDCl_3$) δ (ppm) 172.7, 62.5, 60.3, 52.7, 42.1, 32.6, 14.2, 8.8, 3.9; **HMRS-ESI** (m/z): found $[M+H]^+$ 186.1482, $C_{10}H_{19}NO_2$ requires 186.1494.

Benzyl 3-((cyclopropylmethyl)(methyl)amino)azetidine-1-carboxylate (1n)

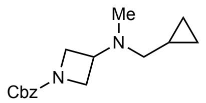

1n

General procedure A was applied to benzyl 3-oxoazetidine-1-carboxylate (2.57 g, 15.0 mmol) with cyclopropylmethanamine (1.73 mL, 20.0 mmol), AcOH (1.15 mL, 20.0 mmol) and $NaBH(OAc)_3$ (4.24 g, 20.0 mmol) in DCM (60.0 mL). The crude product was used without further purification in General procedure B with formaldehyde (37%, 6.75 mL, 90.0 mmol) in formic acid (7.0 mL). Purification by column chromatography (DCM to 5% $NH_{3(MeOH;\ 2M)}$) provided the title compound as a yellow oil (1.76 g, 6.43 mmol, 43% yield).

IR ν_{max}/cm^{-1} (thin film) 2952, 2882, 2799, 1703, 1414, 1350, 1127; ^{1}**H NMR** (600 MHz, $CDCl_3$) δ (ppm) 7.40 – 7.35 (m, 4H), 7.35 – 7.29 (m, 1H), 5.11 (s, 2H), 4.02 (t, *J* = 8.0 Hz, 2H), 3.92 (dd, *J* = 8.5, 5.8 Hz, 2H), 3.28 (p, *J* = 6.3 Hz, 1H), 2.27 (s, 3H), 2.18 (d, *J* = 6.4 Hz, 2H), 0.90 – 0.81 (m, 1H), 0.55 (app q, *J* = 5.1 Hz, 2H), 0.11 (app q, *J* = 5.1 Hz, 2H); ^{13}**C NMR** (151 MHz, $CDCl_3$) δ (ppm) 156.4, 136.7, 128.4, 128.0, 127.9, 66.6, 59.8, 54.1, 54.0, 53.5, 38.4, 8.4, 4.0; **HMRS-ESI** (m/z): found $[M+H]^+$ 275.1750, $C_{16}H_{23}N_2O_2$ requires 275.1760.

***N*-(Cyclopropylmethyl)-2-((5-fluoropyrimidin-2-yl)oxy)-*N*-methylethan-1-amine (1o)**

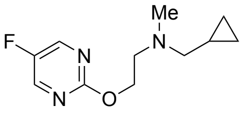

1o

To a solution of NaH (60% in mineral oil, 0.80 g, 20.0 mmol) in THF (30 mL) at 0 °C under N_2 was added 2-(methylamino)ethan-1-ol (1.60 mL, 20.0 mmol) dropwise in THF (10 mL). The resultant mixture was stirred at rt for 30 min before adding 2-chloro-5-fluoropyrimidine (1.84 mL, 20.0 mmol) dropwise at 0 °C. The reaction mixture was stirred at rt for 15 h. After this time, the reaction was concentrated *in vacuo.* The crude mixture was dissolved in DCM (50 mL), washed with aq NaOH (0.5 M, 2 x 50 mL), dried over $MgSO_4$, filtered and concentrated *in vacuo*. The crude amine was used without further purification in General procedure A with cyclopropanecarboxaldehyde (2.24 mL, 30.0 mmol), AcOH (1.15 mL, 20.0 mmol) and $NaBH(OAc)_3$ (6.36 g, 30.0 mmol) in DCM (60 mL). Purification by column chromatography (DCM to 10% $NH_{3(MeOH;\ 2M)}$) provided the title compound as a colourless oil (1.11 g, 4.93 mmol, 25% yield).

IR ν_{max}/cm^{-1} (thin film) 3077, 2948, 2775, 1570, 1426, 1325, 1242, 1032; ^{1}**H NMR** (600 MHz, $CDCl_3$) δ (ppm) 8.38 (s, 2H), 4.46 (t, *J* = 6.1 Hz, 2H), 2.91 (t, *J* = 6.1 Hz, 2H), 2.43 (s, 3H), 2.37 (d, *J* = 6.6 Hz, 2H), 0.94 – 0.86 (m,

1H), 0.57 – 0.49 (m, 2H), 0.15 – 0.10 (m, 2H); **^{13}C NMR** (151 MHz, $CDCl_3$) δ (ppm) 161.4 (d, *J* = 1.2 Hz), 154.2 (d, *J* = 253.4 Hz), 146.6 (d, *J* = 22.5 Hz), 66.4, 63.2, 55.6, 42.9, 8.9, 3.9; **^{19}F NMR** (377 MHz, $CDCl_3$) δ (ppm) –150.5; **HMRS-ESI** (m/z): found $[M+H]^+$ 226.1355, $C_{11}H_{16}N_3OF$ requires 226.1356.

N-(cyclopropylmethyl)-N-ethylethanamine (1p)

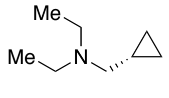

1p

General procedure D was applied to N,N-diethylamine (1.03 mL, 10.0 mmol) with cyclopropane-carboxylic acid (0.80 mL, 10.0 mmol), DIPEA (2.62 mL, 15.0 mmol), HOBt hydrate (2.00 g, 15.0 mmol) and EDC·HCl (2.10 g, 11.0 mmol) in DCM (30 mL). The resultant crude amide was used without further purification in General procedure E with $LiAlH_4$ (0.76 g, 20.0 mmol) in Et_2O (30 mL). Purification by distillation at 140 °C under 1 bar provided the title compound as a colourless oil (840 mg, 6.61 mmol, 66% yield).

IR ν_{max} /cm^{-1} (thin film) 3081, 2976, 2798, 2732, 1455, 1261, 1191, 1032; **^{1}H NMR** (400 MHz, $CDCl_3$) δ (ppm) 2.67 (q, *J* = 8.1 Hz, 4H), 2.18 (app d, *J* = 6.9 Hz, 2H), 1.12 (t, *J* = 8.1 Hz, 6H), 0.90 – 0.79 (m, 1H), 0.56 – 0.48 (m, 2H), 0.15 – 0.09 (m, 2H); **^{13}C NMR** (101 MHz, $CDCl_3$) δ (ppm) 64.7, 45.5, 12.4, 9.2, 3.8; **HMRS-ESI** (m/z): found $[M+H]^+$ 128.1441, $C_8H_{18}N$ requires 128.1439.

N-(cyclopropylmethyl)-N-methylpropan-2-amine (1q)

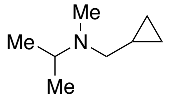

1q

General procedure D was applied to N-methylpropan-2-amine (1.04 mL, 10.0 mmol) with cyclopropane-carboxylic acid (0.80 mL, 10.0 mmol), DIPEA (2.62 mL, 15.0 mmol), HOBt hydrate (2.00 g, 15.0 mmol) and EDC·HCl (2.10 g, 11.0 mmol) in DCM (30 mL). The resultant crude amide was used without further purification in General procedure E with $LiAlH_4$ (0.76 g, 20.0 mmol) in Et_2O (30 mL). Column chromatography (DCM to 7% $NH_{3(MeOH;\ 2M)}$) provided the title compound as a colourless oil (436 mg, 3.42 mmol, 34% yield).

IR ν_{max} /cm^{-1} (thin film) 3092, 2954, 2753, 1477, 1284, 1257, 1168, 1045; **^{1}H NMR** (400 MHz, $CDCl_3$) δ (ppm) 2.73-2.67 (m, 1H), 2.42 (s, 3H), 2.21 (app d, *J* = 7.0 Hz, 2H), 1.12 (d, *J* = 8.2 Hz, 6H), 0.94 – 0.87 (m, 1H), 0.60 – 0.51 (m, 2H), 0.17 – 0.11 (m, 2H); **^{13}C NMR** (101 MHz, $CDCl_3$) δ (ppm) 51.8, 45.5, 21.1, 9.2, 3.8; **HMRS-ESI** (m/z): found $[M+H]^+$ 128.1439, $C_8H_{18}N$ requires 128.1439.

1-(1-(2-Methoxyethyl)cyclopropyl)-N,N-dimethylmethanamine (1r)

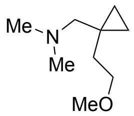

1r

To a solution of cyclopropanecarbonitrile (3.70 mL, 50.0 mmol) in THF (75 mL) at -78 °C was added LDA (30.0 mL, 60.0 mmol, 2 M in THF) dropwise while stirring. The solution was stirred for 30 min at this temperature before the dropwise addition of 1-bromo-2-methoxyethane (5.15 mL, 55.0 mmol). The resultant mixture was left to warm up overnight while stirring. The reaction was quenched with aq NaOH and volatiles were evaporated *in vacuo*. The resultant crude was dissolved in DCM (50 mL) and

aq NaOH (2.5 M, 150 mL). The layers were separated and the aqueous solution was extracted with further DCM (2 x 50 mL). The organic layers were combined, dried over $MgSO_4$, filtered and concentrated *in vacuo*. Purification by column chromatography (PE to DCM) provided 1-(2-methoxyethyl)cyclopropane-1-carbonitrile as a colourless oil (2.73 g, 21.8 mmol, 44% yield).

General procedure E was applied to 1-(2-methoxyethyl)cyclopropane-1-carbonitrile (2.73 g, 21.8 mmol) with $LiAlH_4$ (1.67 g, 44.0 mmol) in Et_2O (100 mL). The resultant primary amine was used without further purification in General procedure B with formaldehyde (37%, 10.0 mL, 130 mmol) in formic acid (11.0 mL). Purification by column chromatography (DCM to 5% $NH_{3(MeOH;\ 2M)}$) provided the title compound as a colourless oil (524 mg, 3.33 mmol, 15% yield).

IR ν_{max}/cm^{-1} (thin film) 2938, 2812, 2762, 1455, 1188, 1117, 1035; **^{1}H NMR** (700 MHz, $CDCl_3$) δ (ppm) 3.50 (t, *J* = 6.8 Hz, 2H), 3.34 (s, 3H), 2.23 (s, 6H), 2.07 (s, 2H), 1.63 (t, *J* = 6.8 Hz, 2H), 0.38 (br s, 2H), 0.26 (br s, 2H); **^{13}C NMR** (176 MHz, $CDCl_3$) δ (ppm) 71.2, 66.1, 58.6, 45.9, 34.3, 15.8, 10.8; **HMRS-ESI** (m/z): found $[M+H]^+$ 158.1543, $C_9H_{20}NO$ requires 158.1545.

***N,N*-Dimethyl-1-(1-methylcyclopropyl)methanamine (1s)**

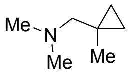

1s

General procedure D was applied to dimethylamine (2.0 M in THF, 30.0 mL, 60.0 mmol) with 1-methylcyclopropane-1-carboxylic acid (5.00 g, 50.0 mmol), DIPEA (13.1 mL, 75.0 mmol), HOBt hydrate (10.0 g, 75.0 mmol) and EDC·HCl (11.5 g, 60.0 mmol) in DCM (100 mL). The resultant crude amide was used without further purification in General procedure E with $LiAlH_4$ (3.80 g, 100.0 mmol) in Et_2O (100 mL) heating to reflux for 16 h. Purification by distillation at 70 °C under 200 mbar provided the title compound as a colourless oil (1.17 g, 10.3 mmol, 21% yield).

IR ν_{max}/cm^{-1} (thin film) 2947, 2812, 2761, 1455, 1252, 1035; **^{1}H NMR** (600 MHz, $CDCl_3$) δ (ppm) 2.24 (s, 6H), 2.09 (s, 2H), 1.10 (s, 3H), 0.33 – 0.28 (m, 2H), 0.28 – 0.24 (m, 2H); **^{13}C NMR** (151 MHz, $CDCl_3$) δ (ppm) 68.7, 45.8, 21.6, 13.8, 12.0; **HMRS-ESI** (m/z): found $[M+H]^+$ 114.1270, $C_7H_{16}N$ requires 114.1283.

1-(1-(4-Chlorophenyl)cyclopropyl)-*N,N*-dimethylmethanamine (1t)

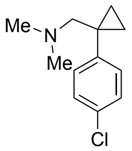

1t

General procedure D was applied to dimethylamine (2.0 M in MeOH, 12.5 mL, 25.0 mmol) with 1-(4-chlorophenyl)cyclopropane-1-carboxylic acid (3.93 g, 20.0 mmol), DIPEA (5.24 mL, 30.0 mmol), HOBt hydrate (4.00 g, 30.0 mmol) and EDC·HCl (4.80 g, 25.0 mmol) in DCM (50 mL). The resultant crude amide was used without further purification in General procedure E with $LiAlH_4$ (1.52 g, 40.0 mmol) in Et_2O (50 mL) heating to reflux for 4 h. The title compound was obtained as a colourless oil without further purification (2.37 g, 11.3 mmol, 57% yield).

IR ν_{max}/cm^{-1} (thin film) 3078, 2941, 2813, 2764, 1494, 1456, 1102, 1035, 1013; **^{1}H NMR** (600 MHz, $CDCl_3$) δ (ppm) 7.32 – 7.23 (m, 4H), 2.47 (s, 2H), 2.22 (s, 6H), 0.87 (dd, *J* = 6.2, 4.4 Hz, 2H), 0.74 (dd, *J* = 6.2, 4.3 Hz, 2H);

^{13}C NMR (151 MHz, $CDCl_3$) δ (ppm) 143.0, 131.5, 129.7, 128.3, 68.8, 46.0, 23.2, 12.7; **HMRS-ESI** (m/z): found $[M+H]^+$ 210.1043, $C_{12}H_{17}NCl$ requires 210.1050.

***trans-N,N*-Dimethyl-1-(2-phenylcyclopropyl)methanamine (5)**

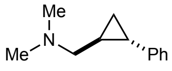

5

General procedure D was applied to dimethylamine (2.0 M in THF, 15.0 mL, 30.0 mmol) with *trans* 2-phenylcyclopropane-1-carboxylic acid (3.24 g, 20.0 mmol), DIPEA (5.3 mL, 30.0 mmol), HOBt hydrate (3.33 g, 25.0 mmol) and EDC·HCl (4.78 g, 25.0 mmol) in DCM (100 mL). The resultant crude amide was used without further purification in THF (20.0 mL) in General procedure E with $LiAlH_4$ (1.52 g, 40.0 mmol) in Et_2O (80.0 mL). The title compound was obtained without further purification as a colourless oil (3.00 g, 17.1 mmol, 86% yield).

IR ν_{max}/cm^{-1} (thin film) 2940, 2761, 1604, 1497, 1455, 1033, 753, 695; **^{1}H NMR** (700 MHz, $CDCl_3$) δ (ppm) 7.27 (app t, *J* = 7.7 Hz, 2H), 7.16 (t, *J* = 7.0 Hz, 1H), 7.09 (d, *J* = 7.7 Hz, 2H), 2.43 (dd, *J* = 12.5, 6.3 Hz, 1H), 2.34 – 2.29 (m, 7H), 1.74 – 1.68 (m, 1H), 1.28 – 1.22 (m, 1H), 0.99 (dt, *J* = 8.7, 5.1 Hz, 1H), 0.86 (dt, *J* = 8.9, 5.3 Hz, 1H); **^{13}C NMR** (176 MHz, $CDCl_3$) δ (ppm) 143.0, 128.3, 125.7, 125.4, 64.1, 45.5, 22.6, 21.6, 15.0; **HMRS-ESI** (m/z): found $[M+H]^+$ 176.1438, $C_{12}H_{18}N$ requires 176.1439.

1-Cyclobutyl-*N,N*-dimethylmethanamine (8a)

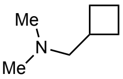

8a

To an aq solution of dimethylamine (40%, 32.0 mL, 250 mmol) at 0 °C was added (bromo-methyl)cyclobutane (5.62 mL, 50.0 mmol). The resultant mixture was stirred at rt for 15 h. After this time, aq NaOH (50 mL, 2.5 M) and sat NaCl (150 mL) were added and an organic layer was collected as a supernatant. The crude product was dissolved in Et_2O (50 mL) and the organic solution was extracted with HCl (0.5 M, 2 x 50 mL). The aqueous layers were combined and basified with NaOH while stirring at 0 °C. The resultant aqueous solution was extracted with Et_2O (2 x 50 mL), dried over $MgSO_4$, filtered and concentrated *in vacuo* to provide the title compound as a colourless oil (1.28 g, 11.3 mmol, 23% yield).

IR ν_{max}/cm^{-1} (thin film) 2969, 2940, 2763, 1456, 1265, 1041, 1023, 842; **^{1}H NMR** (400 MHz, $CDCl_3$) δ (ppm) 2.51 (app hept, *J* = 7.7 Hz, 1H), 2.31 (d, *J* = 7.1 Hz, 2H), 2.21 (s, 6H), 2.14 – 2.03 (m, 2H), 1.96 – 1.86 (m, 1H), 1.85 – 1.78 (m, 1H), 1.72 – 1.64 (m, 2H); **^{13}C NMR** (101 MHz, $CDCl_3$) δ (ppm) 66.5, 45.8, 34.3, 27.5, 18.7; **HMRS-ESI** (m/z): found $[M+H]^+$ 114.1276, $C_7H_{16}N$ requires 114.1277.

Ethyl 3-((cyclobutylmethyl)(methyl)amino)propanoate (8b)

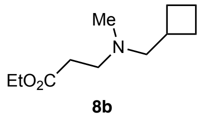

8b

To a solution of methylamine (33% in EtOH, 6.17 mL, 50.0 mmol) in EtOH (40 mL) at 0 °C was added ethyl acrylate (2.72 mL, 25.0 mmol) in EtOH (10 mL). The resultant mixture was warmed to rt and stirred for 15 h. After this time, the reaction was concentrated *in vacuo* and the crude product was dissolved in DCM (50 mL) and washed with aq NaOH (2.5 M, 2 x 50 mL), dried over

$MgSO_4$, filtered and concentrated *in vacuo*. The crude mixture was used without further purification in General procedure F with (bromomethyl)cyclobutane (2.80 mL, 25.0 mmol) and DIPEA (5.24 mL, 30.0 mmol) in MeCN (50 mL). Purification by column chromatography (DCM to 3% $NH_{3(MeOH;\ 2M)}$) provided the title compound as a colourless oil (690 mg, 3.46 mmol, 14% yield).

IR ν_{max}/cm^{-1} (thin film) 2972, 2772, 1733, 1178, 1027; **^{1}H NMR** (700 MHz, $CDCl_3$) δ (ppm) 4.15 (q, *J* = 7.1 Hz, 2H), 2.69 (t, *J* = 7.3 Hz, 2H), 2.52 (hept, *J* = 7.8 Hz, 1H), 2.47 (t, *J* = 7.3 Hz, 2H), 2.40 (d, *J* = 7.0 Hz, 2H), 2.21 (s, 3H), 2.12 – 2.03 (m, 2H), 1.95 – 1.87 (app hex, *J* = 9.2 Hz, 1H), 1.87 – 1.75 (m, 1H), 1.67 (app p, *J* = 9.1 Hz, 2H), 1.28 (t, *J* = 7.1 Hz, 3H); **^{13}C NMR** (176 MHz, $CDCl_3$) δ (ppm) 172.8, 63.9, 60.4, 53.0, 42.3, 34.0, 32.5, 27.5, 18.8, 14.2; **HMRS-ESI** (m/z): found $[M+H]^+$ 200.1646, $C_{11}H_{22}NO_2$ requires 200.1651.

_N_-(Cyclobutylmethyl)-_N_-methyl-3-(5-methylfuran-2-yl)propan-1-amine (8c)

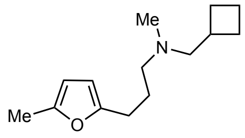

8c

To a solution of methylamine hydrochloride (10.1 g, 150 mmol) in MeOH (100 mL), KOH (8.40 g, 150 mmol) was added portionwise while stirring at 0 °C. (Bromomethyl)-cyclobutane (5.60 mL, 50.0 mmol) was added to the resultant mixture and the reaction was heated to 50 °C for 15 h. The reaction was quenched with aq HCl (3 M, 30 mL) and volatiles were evaporated *in vacuo*. The resultant aqueous layer was basified with aq NaOH until a basic pH > 12 was reached, and extracted with Et_2O (3 x 30 mL). The organic layers were combined, dried over $MgSO_4$, filtered and concentrated *in vacuo*. 7.5 mmol of the resultant crude amine were used without further purification in General procedure A with 3-(5-methylfuran-2-yl)propanal (1.33 mL, 10.0 mmol), AcOH (430 µL, 7.5 mmol) and $NaBH(OAc)_3$ (2.54 g, 12.0 mmol) in DCM (30 mL). Purification by column chromatography (DCM to 4% $NH_{3(MeOH;\ 2M)}$) provided the title compound as a yellow oil (862 mg, 3.90 mmol, 52% yield).

IR ν_{max}/cm^{-1} (thin film) 2945, 2783, 1569, 1453, 1219, 1018, 775; **^{1}H NMR** (700 MHz, $CDCl_3$) δ (ppm) 5.86 (m, 2H), 2.59 (t, *J* = 7.5 Hz, 2H), 2.56 – 2.48 (app hept, *J* = 7.2 Hz, 1H), 2.39 (d, *J* = 7.0 Hz, 2H), 2.36 (t, *J* = 7.4 Hz, 2H), 2.27 (s, 3H), 2.21 (s, 3H), 2.11 – 2.03 (m, 2H), 1.95 – 1.88 (m, 1H), 1.85 – 1.76 (m, 3H), 1.68 (p, *J* = 9.0 Hz, 2H); **^{13}C NMR** (176 MHz, $CDCl_3$) δ (ppm) 154.3, 150.1, 105.7, 105.3, 64.2, 57.3, 42.7, 34.2, 27.6, 26.0, 25.8, 18.8, 13.5; **HMRS-ESI** (m/z): found $[M+H]^+$ 222.1858, $C_{14}H_{24}NO$ requires 222.1858.

3-((_tert_-Butyldimethylsilyl)oxy)-_N_-(cyclobutylmethyl)-_N_-methylpropan-1-amine (8d)

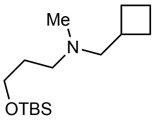

8d

To a solution of methylamine hydrochloride (10.1 g, 150 mmol) in MeOH (100 mL), KOH (8.40 g, 150 mmol) was added portionwise while stirring at 0 °C. (Bromomethyl)cyclobutane (5.60 mL, 50.0 mmol) was added to the resultant mixture and the reaction was heated to 50 °C for 15 h. The reaction was quenched with aq HCl (3 M, 30 mL) and volatiles were evaporated *in vacuo*. The resultant aqueous layer was basified with aq NaOH until a basic pH > 12 was reached, and extracted with Et_2O (3 x 30 mL). The organic layers were combined, dried over $MgSO_4$, filtered and concentrated *in vacuo*. 12.0

mmol of the resultant crude amine were used without further purification in General procedure F with (3-bromopropoxy)(*tert*-butyl)dimethylsilane (2.32 mL, 10.0 mmol) and DIPEA (2.62 mL, 15.0 mmol) in MeCN (20 mL). Purification by column chromatography (DCM to 4% $NH_{3(MeOH;\ 2M)}$) provided the title compound as a colourless oil (1.62 g, 5.97 mmol, 60% yield).

IR ν_{max}/cm^{-1} (thin film) 2952, 2856, 1462, 1251, 1096, 832, 772; **^{1}H NMR** (700 MHz, $CDCl_3$) δ (ppm) 3.66 (t, *J* = 6.4 Hz, 2H), 2.56 – 2.48 (m, 1H), 2.42 – 2.36 (m, 4H), 2.19 (s, 3H), 2.11 – 2.04 (m, 2H), 1.95 – 1.87 (m, 1H), 1.85 – 1.79 (m, 1H), 1.72 – 1.64 (m, 4H), 0.91 (s, 9H), 0.07 (s, 6H); **^{13}C NMR** (176 MHz, $CDCl_3$) δ (ppm) 64.3, 61.6, 54.6, 42.7, 34.2, 30.5, 27.7, 26.0, 18.8, 18.3, -5.3; **HMRS-ESI** (m/z): found $[M+H]^+$ 272.2409, $C_{15}H_{34}NOSi$ requires 272.2410.

***tert*-Butyl (2-((cyclobutylmethyl)(methyl)amino)ethyl)(methyl)carbamate (8e)**

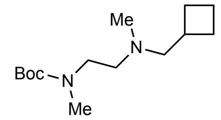

8e

N^1,N^2-dimethylethane-1,2-diamine (8.00 mL, 75.0 mmol) was added to a solution of Boc_2O (5.46 g, 25.0 mmol) and Et_3N (3.48 mL, 25.0 mmol) in DCM (100 mL) at 0 °C. The resultant mixture was warmed to rt and stirred for 15 h. After this time, the organic layer was washed with aq NaOH (2.5 M, 2 x 50 mL), dried over $MgSO_4$, filtered and concentrated *in vacuo*. The crude mixture was used without further purification in General procedure F with (bromomethyl)cyclobutane (2.80 mL, 25.0 mmol) and DIPEA (5.24 mL, 30.0 mmol) in MeCN (50 mL). Purification by column chromatography (DCM to 3% $NH_{3(MeOH;\ 2M)}$) provided the title compound as a colourless oil (600 mg, 2.34 mmol, 9% yield).

IR ν_{max}/cm^{-1} (thin film) 2971, 2931, 2781, 1692, 1389, 1159; **^{1}H NMR** (700 MHz, $CDCl_3$) δ (ppm) 3.30 (m, 2H, H_5), 2.88 (s, 3H), 2.56 – 2.45 (m, 3H), 2.42 (d, *J* = 7.0 Hz, 2H), 2.24 (s, 3H), 2.12 – 2.02 (m, 2H), 1.95 – 1.87 (m, 1H), 1.86 – 1.80 (m, 1H), 1.71 – 1.64 (m, 2H), 1.48 (s, 9H); **^{13}C NMR** (126 MHz, $CDCl_3$) δ (ppm) 155.7, 79.2, 64.5, 55.4, 47.0, 42.9, 34.6, 34.2, 28.5, 27.5, 18.8; **HMRS-ESI** (m/z): found $[M+H]^+$ 257.2220, $C_{14}H_{29}N_2O_2$ requires 257.2229.

1-(Cyclobutylmethyl)piperidine (8f)

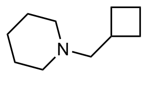

8f

General procedure D was applied to piperidine (1.98 mL, 20.0 mmol) with cyclobutane-carboxylic acid (2.00 mL, 21.0 mmol), DIPEA (5.24 mL, 30.0 mmol), HOBt hydrate (4.00 g, 30.0 mmol) and EDC·HCl (4.22 g, 22.0 mmol) in DCM (40 mL). The resultant crude amide was used without further purification in General procedure E with $LiAlH_4$ (1.52 g, 40.0 mmol) in Et_2O (60 mL). Purification by distillation at 100 °C under 30 mbar provided the title compound as a colourless oil (2.33 g, 15.2 mmol, 76% yield).

IR ν_{max}/cm^{-1} (thin film) 2931, 2854, 2756, 1442, 1154, 1125, 1038, 995; **^{1}H NMR** (600 MHz, $CDCl_3$) δ (ppm) 2.56 (app hept, *J* = 7.6 Hz, 1H), 2.38 (d, *J* = 6.7 Hz, 2H), 2.34 (br s, 4H), 2.11 – 2.02 (m, 2H), 1.89 (app hept, *J* = 8.8 Hz, 1H), 1.82 – 1.75 (m, 1H), 1.68 (pd, *J* = 8.9, 2.1 Hz, 2H), 1.57 (app p, *J* = 5.6 Hz, 4H), 1.47 – 1.35 (m, 2H); **^{13}C**

NMR (151 MHz, $CDCl_3$) δ (ppm) 66.2, 54.6, 34.2, 28.3, 25.9, 24.3, 18.8; **HMRS-ESI** (m/z): found $[M+H]^+$ 154.1588, $C_{10}H_{20}N$ requires 154.1590.

8-(Cyclobutylmethyl)-1,4-dioxa-8-azaspiro[4.5]decane (8g)

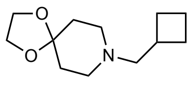

8g

General procedure F was applied to 1,4-dioxa-8-azaspiro[4.5]decane (1.28 mL, 10.0 mmol) with (bromomethyl)cyclobutane (1.23 mL, 11.0 mmol) and DIPEA (2.62 mL, 15.0 mmol) in MeCN (25 mL) to provide by column chromatography (DCM to 5% $NH_{3(MeOH;\ 2M)}$) the title compound as a yellow oil (1.16 g, 5.50 mmol, 55% yield).

IR ν_{max}/cm^{-1} (thin film) 2953, 2873, 2807, 1307, 1089, 1039, 915; **^{1}H NMR** (700 MHz, $CDCl_3$) δ (ppm) 3.96 (s, 4H), 2.60 – 2.45 (m, 5H), 2.44 (d, *J* = 6.7 Hz, 2H), 2.12 – 2.03 (m, 2H), 1.94 – 1.85 (m, 1H), 1.85 – 1.77 (m, 1H), 1.76 – 1.72 (m, 4H), 1.72 – 1.65 (m, 2H); **^{13}C NMR** (176 MHz, $CDCl_3$) δ (ppm) 107.3, 65.0, 64.2, 51.4, 34.8, 34.3, 28.1, 18.8; **HMRS-ESI** (m/z): found $[M+H]^+$ 212.1652, $C_{11}H_{22}NO_2$ requires 212.1651.

(2*S*,6*R*)-4-(Cyclobutylmethyl)-2,6-dimethylmorpholine (8h)

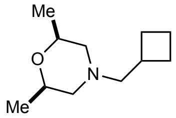

8h

General procedure F was applied to (2*R*,6*S*)-2,6-dimethylmorpholine (1.23 mL, 10.0 mmol) with (bromomethyl)cyclobutane (1.23 mL, 11.0 mmol) and DIPEA (2.62 mL, 15.0 mmol) in MeCN (25 mL) to provide by column chromatography (DCM to 5% $NH_{3(MeOH;\ 2M)}$) the title compound as a yellow oil (1.01 g, 5.51 mmol, 55% yield).

IR ν_{max}/cm^{-1} (thin film) 2969, 2931, 2865, 1455, 1373, 1322, 1143, 1083; **^{1}H NMR** (700 MHz, $CDCl_3$) δ (ppm) 3.71 – 3.62 (m, 2H), 2.67 (d, *J* = 11.2 Hz, 2H), 2.55 (hept, *J* = 7.0 Hz, 1H), 2.38 (d, *J* = 6.8 Hz, 2H), 2.12 – 2.02 (m, 2H), 1.95 – 1.86 (m, 1H), 1.86 – 1.78 (m, 1H), 1.74 – 1.65 (m, 4H), 1.15 (d, *J* = 6.2 Hz, 6H); **^{13}C NMR** (176 MHz, $CDCl_3$) δ (ppm) 71.6, 65.3, 59.7, 33.7, 27.9, 19.2, 18.9; **HMRS-ESI** (m/z): found $[M+H]^+$ 184.1698, $C_{11}H_{22}NO$ requires 184.1701.

1-(Cyclobutylmethyl)-4-(4-nitrophenyl)piperazine (8i)

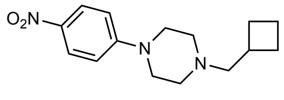

8i

General procedure F was applied to 1-(4-nitrophenyl)piperazine (2.07 g, 10.0 mmol) with (bromomethyl)cyclobutane (1.12 mL, 10.0 mmol) and DIPEA (2.10 mL, 12.0 mmol) in MeCN (20.0 mL) to provide by column chromatography (DCM to 3% $NH_{3(MeOH;\ 2M)}$) the title compound as a yellow solid (1.46 g, 5.30 mmol, 53% yield).

mp (°C) 88–90; **IR** ν_{max}/cm^{-1} (thin film) 2922, 1588, 1492, 1324, 1239, 1114, 1002, 923, 823; **^{1}H NMR** (700 MHz, $CDCl_3$) δ (ppm) 8.13 (d, *J* = 9.1 Hz, 2H), 6.82 (d, *J* = 9.1 Hz, 2H), 3.42 (br s, *J* = 4.1 Hz, 4H), 2.62 – 2.52 (m, 5H), 2.47 (d, *J* = 6.9 Hz, 2H), 2.15 – 2.06 (m, 2H), 1.98 – 1.89 (app hex, *J* = 9.3 Hz, 1H), 1.88 – 1.80 (m, 1H), 1.73 (app p, *J* = 9.2 Hz, 2H); **^{13}C NMR** (176 MHz, $CDCl_3$) δ (ppm) 154.9, 138.3, 125.9, 112.5, 65.0, 52.8, 47.0, 33.7, 27.8, 18.9; **HMRS-ESI** (m/z): found $[M+H]^+$ 276.1711, $C_{15}H_{22}N_3O_2$ requires 276.1712.

1-(1-(Methoxymethyl)cyclobutyl)-*N*,*N*-dimethylmethanamine (8j)

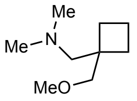

8j

To a solution of (1-(methoxymethyl)cyclobutyl)methanamine (1.00 g, 6.00 mmol) and aq formaldehyde (37%, 2.23 mL, 30.0 mmol) in MeOH (12 mL) under N_2 at 0 °C was added $NaBH_3CN$ (1.51 g, 24.0 mmol). The resultant mixture was stirred at rt for 15 h. The reaction was quenched with aq HCl (3 M, 15 mL) and concentrated *in vacuo*. DCM was added (50 mL) and the organic layer was washed with NaOH (2.5 M, 2 x 50 mL), dried over $MgSO_4$, filtered and concentrated *in vacuo*. Purification by column chromatography (DCM to 5% $NH_{3(MeOH;\ 2M)}$) provided the title compound as a colourless oil (577 mg, 3.67 mmol, 61% yield).

IR ν_{max} /cm^{-1} (thin film) 2973, 2938, 2871, 2814, 2763, 1456, 1108, 1032; **^{1}H NMR** (700 MHz, $CDCl_3$) δ (ppm) 3.46 (s, 2H), 3.41 (s, 3H), 2.37 (s, 2H), 2.22 (s, 6H), 1.97 – 1.77 (m, 6H); **^{13}C NMR** (176 MHz, $CDCl_3$) δ (ppm) 76.7, 66.3, 59.3, 47.2, 42.5, 28.7, 16.0; **HMRS-ESI** (m/z): found $[M+H]^+$ 158.1546, $C_9H_{20}NO$ requires 158.1545.

3. Optimization of aryl boronic acid scope

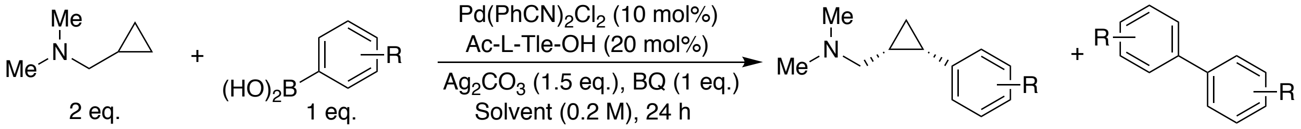

R	Solvent	T (˚C)	t (h)	%Yield product	%Yield biaryl
3-NO_2	DMF	40	15	45	19
3-NO_2	NMP	40	15	55	15
3-NO_2	DMA	40	15	58	12
3-NO_2	DMA	40	24	56	12
3-NO_2	DMA	40	40	61	14
3-NO_2	DMA	30	40	69 (**64**)	9
4-CO_2Et	DMF	40	15	55	15
4-CO_2Et	NMP	40	15	65	10
4-CO_2Et	DMA	40	15	65	8
4-CO_2Et	DMA	40	24	71 (**66**)	7
4-CO_2Et	DMA	30	24	59	8
4-CO_2Et	DMA	30	40	67	7
4-OMe	DMA	30	24	58	14
4-OMe	DMA	40	24	75 (**70**)	8
4-OMe	DMA	50	24	66	10
4-OMe	DMA	30	40	70	8
4-OMe	DMA	40	40	73	8

Yields were determined by ^{1}H-NMR using 1,1,2,2-tetrachloroethane as internal standard. Yield bold in brackets of isolated product after purification by silica gel chromatography.

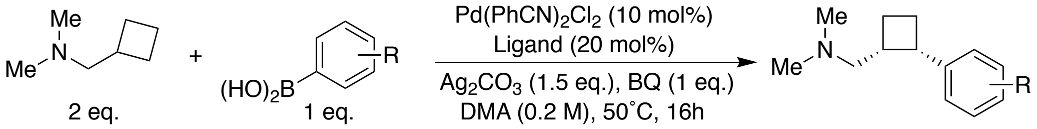

R	Ligand	%Yield
4-OMe	N-Ac-(L)-Tle-OH	52
4-OMe	N-Ac-(L)-Phe-OH	62 (**49**)
4-OMe	N-Ac-(L)-Ala-OH	57
4-CO_2Et	N-Ac-(L)-Tle-OH	55
4-CO_2Et	N-Ac-(L)-Phe-OH	68 (**54**)
4-CO_2Et	N-Ac-(L)-Ala-OH	58

Yields were determined by ^{1}H-NMR using 1,1,2,2-tetrachloroethane as internal standard. Yield bold in brackets of isolated product after purification by silica gel chromatography.

4. C(sp³)–H arylated products

General procedure G: Methylene cyclopropane C–H arylation

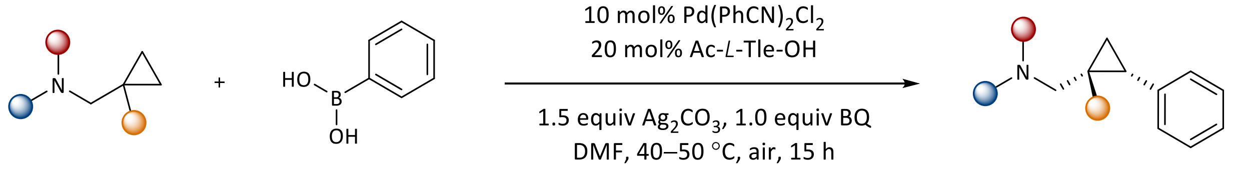

The desired tertiary amine (0.600 mmol) was added to a 2–5 mL microwave vial containing Pd(PhCN)$_2$Cl$_2$ (11.5 mg, 0.030 mmol), Ac-*L*-Tle-OH (10.4 mg, 0.060 mmol), Ag_2CO_3 (124 mg, 0.450 mmol) and 1,4-benzoquinone (BQ) (32.5 mg, 0.300 mmol) in anhydrous DMF (0.75 mL). The reaction was sealed and stirred at the required temperature for 5 minutes. The desired (hetero)aryl boronic acid (0.300 mmol) in DMF (0.75 mL) was added dropwise and the reaction mixture was stirred at 1000 rpm for 15 h.

The reaction was cooled to rt and Et_2O (5 mL) added, forcing the formation of a precipitate. The dark mixture was filtered through a pad of Celite and washed with Et_2O (25 mL). The resulting organic layer was washed with aq NaOH (0.25 M, 3 x 50 mL), dried over $MgSO_4$, filtered and concentrated *in vacuo*. Purification of the crude oil by column chromatography afforded the pure arylated product. Asymmetry was determined by HPLC analysis using a CHIRALPAK® AD-H column, GC-FID analysis using a Astec Chiraldex™ B-DM column, or ^{1}H-NMR analysis of the methylated tertiary amine product following a literature procedure reported by Lacour (*Org. Lett.* **2002**, *4*, 8, 1351–1354).

N,N-Dimethyl-1-((1*R*,2*S*)-2-phenylcyclopropyl)methanamine (3a)

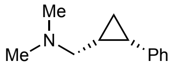

3a

General procedure G was applied to 1-cyclopropyl-*N,N*-dimethylmethanamine (59.6 mg, 0.600 mmol) with phenyl boronic acid (36.6 mg, 0.300 mmol) at 40 °C to provide by column chromatography (DCM to 4% $NH_{3(MeOH;\ 2M)}$) the title compound as a brown oil (44.2 mg, 0.245 mmol, 82% yield, >99:1 e.r.). Enantiomeric excess was determined by ^{1}H-NMR from the corresponding methylated ammonium derivative.

IR ν_{max} /cm^{-1} (thin film) 2941, 2814, 1497, 1457, 1262, 1029, 844; **^{1}H NMR** (600 MHz, $CDCl_3$) δ (ppm) 7.31 – 7.26 (m, 2H), 7.24 – 7.17 (m, 3H), 2.30 (dd, *J* = 12.7, 5.0 Hz, 1H), 2.23 – 2.15 (m, 7H), 1.67 (dd, *J* = 12.7, 8.4 Hz, 1H), 1.31 – 1.24 (m, 1H), 1.17 – 1.07 (m, 1H), 0.82 (q, *J* = 5.8 Hz, 1H); **^{13}C NMR** (151 MHz, $CDCl_3$) δ (ppm) 138.8, 129.0, 127.9, 125.8, 59.0, 45.4, 20.3, 17.0, 9.6; **HMRS-ESI** (m/z): found $[M+H]^+$ 176.1428, $C_{12}H_{18}N$ requires 176.1434; **ee analysis:** ^{1}H-NMR from the corresponding methylated ammonium derivative; HPLC Chiralpak AD-H (hexane(0.1% DEA):2-propanol 98:2, 1.0 mL·min^{-1}, 30 °C) t_R = 5.1 min (major), t_R = 5.5 min (minor); $[\alpha]_D^{25.0}$ = –77° (c = 1.0, $CHCl_3$).

1-(((1*R*,2*S*)-2-Phenylcyclopropyl)methyl)piperidine (3b)

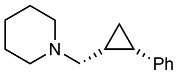

3b

General procedure G was applied to 1-(cyclopropylmethyl)piperidine (83.5 mg, 0.600 mmol) with phenyl boronic acid (36.6 mg, 0.300 mmol) at 50 °C to provide by column chromatography (DCM to 4% $NH_{3(MeOH;\ 2M)}$) the title compound as a brown oil (49.2 mg, 0.229 mmol, 76% yield, >97:3 e.r.). Enantiomeric excess was determined by ^{1}H-NMR from the corresponding methylated ammonium derivative.

IR ν_{max}/cm^{-1} (thin film) 2931, 1497, 1453, 1299, 1115, 1028, 763, 696; **^{1}H NMR** (600 MHz, $CDCl_3$) δ (ppm) 7.30 – 7.25 (m, 2H), 7.22 – 7.16 (m, 3H), 2.36 (dd, *J* = 12.9, 5.0 Hz, 1H), 2.34 (br s, 4H), 2.21 – 2.15 (m, 1H), 1.79 (dd, *J* = 12.9, 8.0 Hz, 1H), 1.58 (app p, *J* = 5.6 Hz, 4H), 1.39 (br s, 2H), 1.36 – 1.28 (m, 1H), 1.12 (td, *J* = 8.4, 5.2 Hz, 1H), 0.82 (q, *J* = 5.7 Hz, 1H); **^{13}C NMR** (151 MHz, $CDCl_3$) δ (ppm) 138.8, 129.0, 127.9, 125.8, 58.7, 54.4, 25.8, 24.3, 20.2, 16.4, 10.0; **HMRS-ESI** (m/z): found $[M+H]^+$ 216.1739, $C_{15}H_{22}N$ requires 216.1747; **ee analysis:** ^{1}H-NMR (H_7) from the corresponding methylated ammonium derivative; $[\boldsymbol{\alpha}]_{\boldsymbol{D}}^{\mathbf{25.0}}$ = –51° (c = 0.8, $CHCl_3$).

***tert*-Butyl (1-(((1*R*,2*S*)-2-phenylcyclopropyl)methyl)piperidin-4-yl)carbamate (3c)**

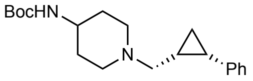

3c

General procedure G was applied to *tert*-butyl (1-(cyclopropyl-methyl)piperidin-4-yl)carbamate (153 mg, 0.600 mmol) with phenyl boronic acid (36.6 mg, 0.300 mmol) at 40 °C to provide by column chromatography (DCM to 6% $NH_{3(MeOH;\ 2M)}$) the title compound as a yellow oil (75.0 mg, 0.227 mmol, 76% yield, >99:1 e.r). Enantiomeric excess was determined by HPLC analysis.

IR ν_{max}/cm^{-1} (thin film) 3321, 2930, 2781, 1686, 1497, 1168, 765; **^{1}H NMR** (600 MHz, $CDCl_3$) δ (ppm) 7.29 – 7.23 (m, 2H), 7.21 – 7.15 (m, 3H), 4.43 (br s, 1H), 3.39 (app br s, 1H), 2.89 (br s, 1H), 2.77 (br s, 1H), 2.35 (dd, *J* = 12.9, 5.2 Hz, 1H), 2.18 (app q, *J* = 7.8 Hz, 1H), 1.97 (br t, *J* = 11.2 Hz, 1H), 1.88 (br s, 3H), 1.83 (dd, *J* = 12.9, 7.9 Hz, 1H), 1.50 – 1.37 (m, 11H), 1.31 – 1.24 (m, 1H), 1.11 (td, *J* = 8.4, 5.2 Hz, 1H), 0.81 (q, *J* = 5.7 Hz, 1H); **^{13}C NMR** (151 MHz, $CDCl_3$) δ (ppm) 155.2, 138.6, 128.9, 127.9, 125.9, 79.2, 57.9, 52.3, 47.6, 32.5, 28.4, 20.3, 16.4, 9.8; **HMRS-ESI** (m/z): found $[M+H]^+$ 331.2380, $C_{20}H_{31}N_2O_2$ requires 331.2386; **ee analysis:** HPLC Chiralpak AD-H (hexane(0.1% DEA):2-propanol 98:2, 1.0 mL·min^{-1}, 45 °C) t_R = 17.9 min (minor), t_R = 18.7 min (major); $[\boldsymbol{\alpha}]_{\boldsymbol{D}}^{\mathbf{25.0}}$ = –39° (c = 1.0, $CHCl_3$).

1-(((1*R*,2*S*)-2-Phenylcyclopropyl)methyl)-4-tosylpiperazine (3d)

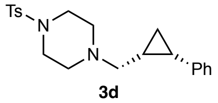

3d

General procedure G was applied to 1-(cyclopropylmethyl)-4-tosylpiperazine (176.6 mg, 0.600 mmol) with phenyl boronic acid (36.6 mg, 0.300 mmol) at 40 °C to provide by column chromatography (DCM to 3% $NH_{3(MeOH;\ 2M)}$) the title compound as a white solid (55.3 mg, 0.149 mmol, 50% yield, 98.5:1.5 e.r.). Enantiomeric excess was determined by HPLC analysis.

mp 72 – 74 °C; **IR** ν_{max}/cm^{-1} (thin film) 2856, 2801, 1595, 1452, 1347, 1301, 1163; **^{1}H NMR** (600 MHz, $CDCl_3$) δ (ppm) 7.63 (d, *J* = 8.0 Hz, 2H), 7.32 (d, *J* = 8.0 Hz, 2H), 7.28 – 7.25 (m, 2H), 7.22 – 7.17 (m, 1H), 7.15 (d, *J* = 7.2

Hz, 2H), 2.98 (br s, 4H), 2.44 (app br s, 7H), 2.32 (dd, J = 13.0, 5.5 Hz, 1H), 2.18 (td, J = 8.7, 6.1 Hz, 1H), 1.87 (dd, J = 13.0, 7.7 Hz, 1H), 1.19 (qt, J = 8.4, 5.7 Hz, 1H), 1.08 (td, J = 8.4, 5.3 Hz, 1H), 0.77 (q, J = 5.8 Hz, 1H); **^{13}C NMR** (151 MHz, $CDCl_3$) δ (ppm) 143.6, 138.4, 132.2, 129.6, 128.9, 128.0, 127.9, 126.0, 57.4, 52.0, 46.0, 21.5, 20.3, 16.2, 9.4; **HMRS-ESI** (m/z): found $[M+H]^+$ 371.1785, $C_{21}H_{27}N_2O_2S$ requires 371.1793; **ee analysis:** HPLC Chiralpak AD-H (hexane:2-propanol 90:10, 1.0 mL·min^{-1}, 30 °C) t_R = 16.3 min (major), t_R = 19.2 min (minor); $[\alpha]_D^{25.0}$ = –23° (c = 1.0, $CHCl_3$).

(2*R*,6*S*)-2,6-dimethyl-4-(((1*R*,2*S*)-2-phenylcyclopropyl)methyl)morpholine (3e)

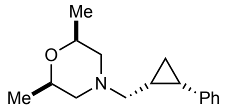

3e

General procedure G was applied to (2*R*,6*S*)-4-(cyclopropylmethyl)-2,6-dimethyl-morpholine (101 mg, 0.600 mmol) with phenyl boronic acid (36.6 mg, 0.300 mmol) at 50 °C to provide by column chromatography (DCM to 4% $NH_{3(MeOH;\ 2M)}$) the title compound as a brown oil (79.0 mg, 0.195 mmol, 65% yield, 99:1 e.r.). Enantiomeric excess was determined by HPLC analysis.

IR ν_{max}/cm^{-1} (thin film) 2970, 2932, 2867, 1455, 1143, 1082, 769, 698; **^{1}H NMR** (600 MHz, $CDCl_3$) δ (ppm) 7.31 – 7.24 (m, 2H), 7.23 – 7.15 (m, 3H), 3.72 – 3.60 (m, 2H), 2.80 (d, J = 11.3 Hz, 1H), 2.67 (d, J = 11.3 Hz, 1H), 2.33 (dd, J = 12.8, 5.4 Hz, 1H), 2.24 – 2.16 (m, 1H), 1.83 (dd, J = 12.8, 7.8 Hz, 1H), 1.62 (t, J = 10.7 Hz, 1H), 1.49 (t, J = 10.8 Hz, 1H), 1.34 – 1.25 (m, 1H), 1.17 – 1.06 (m, 7H, H_1), 0.83 (q, J = 5.7 Hz, 1H); **^{13}C NMR** (151 MHz, $CDCl_3$) δ (ppm) 138.7, 129.0, 127.9, 125.9, 71.5, 59.5, 59.4, 57.9, 20.2, 19.2, 19.1, 16.1, 9.7; **HMRS-ESI** (m/z): found $[M+H]^+$ 246.1855, $C_{16}H_{24}NO$ requires 246.1858; **ee analysis:** HPLC Chiralpak AD-H (hexane:2-propanol 99.5:0.5, 1.0 mL·min^{-1}, 30 °C) t_R = 5.8 min (major), t_R = 7.0 min (minor); $[\alpha]_D^{25.0}$ = -52° (c = 1.0, $CHCl_3$).

1'-(((1*R*,2*S*)-2-Phenylcyclopropyl)methyl)spiro[chromane-2,4'-piperidin]-4-one (3f)

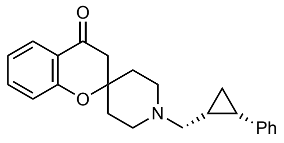

3f

General procedure G was applied to 1'-(cyclopropylmethyl)-spiro[chromane-2,4'-piperidin]-4-one (163 mg, 0.600 mmol) with phenyl boronic acid (36.6 mg, 0.300 mmol) at 40 °C to provide by column chromatography (DCM to 5% $NH_{3(MeOH;\ 2M)}$) the title compound as a yellow oil (74.8 mg, 0.215 mmol, 72% yield, >99:1 e.r.). Enantiomeric excess was determined by HPLC analysis.

IR ν_{max}/cm^{-1} (thin film) 3076, 2919, 2786, 1687, 1605, 1461, 1298, 1228, 1114; **^{1}H NMR** (600 MHz, $CDCl_3$) δ (ppm) 7.85 (dd, J = 8.0, 1.7 Hz, 1H), 7.47 (td, J = 7.7, 1.7 Hz, 1H), 7.32 – 7.26 (m, 2H), 7.25 – 7.17 (m, 3H), 6.99 (td, J = 7.6, 0.8 Hz, 1H), 6.94 (d, J = 8.3 Hz, 1H), 2.81 – 2.65 (m, 3H), 2.57 (br d, J = 11.0 Hz, 1H), 2.45 (dd, J = 12.9, 4.7 Hz, 1H), 2.31 (br q, J = 10.2 Hz, 2H), 2.20 (app q, J = 8.3 Hz, 1H), 2.06 – 1.97 (m, 2H), 1.79 (dd, J = 12.9, 8.3 Hz, 1H), 1.76 – 1.70 (m, 2H), 1.32 – 1.24 (m, 1H), 1.13 (td, J = 8.3, 5.2 Hz, 1H), 0.83 (q, J = 5.7 Hz, 1H); **^{13}C NMR** (101 MHz, $CDCl_3$) δ (ppm) 192.1, 159.2, 138.8, 136.1, 129.0, 127.9, 126.5, 125.9, 120.9, 120.8, 118.4, 77.9, 58.1, 48.9, 48.5, 48.0, 34.3, 34.3, 20.1, 16.5, 10.0; **HMRS-ESI** (m/z): found $[M+H]^+$ 348.1963, $C_{23}H_{26}NO_2$

requires 348.1964; **ee analysis:** HPLC Chiralpak AD-H (hexane:2-propanol 97:3, 1.0 mL·min^{-1}, 30 °C) t_R = 14.5 min (major), t_R = 16.4 min (minor); $[\alpha]_D^{25.0}$ = –37° (c = 1.0, $CHCl_3$).

4-(4-Chlorophenyl)-1-(((1*R*,2*S*)-2-phenylcyclopropyl)methyl)piperidin-4-ol (3g)

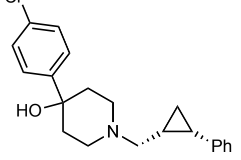

3g

General procedure G was applied to 4-(4-chlorophenyl)-1-(cyclopropylmethyl)piperidin-4-ol (159.5 mg, 0.600 mmol) with phenyl boronic acid (36.6 mg, 0.300 mmol) at 50 °C to provide by column chromatography (DCM to 7% $NH_{3(MeOH;\ 2M)}$) the title compound as a colourless oil (52.1 mg, 0.152 mmol, 51% yield, >99:1 e.r.). Enantiomeric excess was determined by HPLC analysis.

IR ν_{max}/cm^{-1} (thin film) 3063, 2924, 2833, 1495, 1082, 1047; **^{1}H NMR** (400 MHz, $CDCl_3$) δ (ppm) 7.45 – 7.41 (m, 2H), 7.33 – 7.27 (m, 4H), 7.23 – 7.18 (m, 3H), 2.94 (app br d, *J* = 11.1 Hz, 1H), 2.79 (app br d, *J* = 11.4 Hz, 1H), 2.48 (dd, *J* = 12.9, 5.0 Hz, 1H), 2.39 (br t, *J* = 11.8 Hz, 1H), 2.31 (br t, *J* = 11.5 Hz, 1H), 2.23 (app q, *J* = 8.1 Hz, 1H), 2.15 (br t, *J* = 11.3 Hz, 2H), 1.93 (dd, *J* = 12.1, 8.3 Hz, 1H), 1.90 – 1.75 (br s, 1H), 1.74 – 1.64 (m, 2H), 1.39 – 1.30 (m, 1H), 1.15 (td, *J* = 8.4, 5.3 Hz, 1H), 0.89 (q, *J* = 5.7 Hz, 1H); **^{13}C NMR** (101 MHz, $CDCl_3$) δ (ppm) 146.8, 138.5, 132.8, 128.9, 128.4, 128.0, 126.1, 125.9, 70.9, 58.0, 49.1, 38.1, 20.3, 16.1, 10.0; **HMRS-ESI** (m/z): found $[M+H]^+$ 342.1617, $C_{21}H_{25}NOCl$ requires 342.1625; **ee analysis:** HPLC Chiralpak AD-H (hexane:2-propanol 95:5, 1.0 mL·min^{-1}, 30 °C) t_R = 24.2 min (minor), t_R = 26.1 min (major); $[\alpha]_D^{25.0}$ = –32° (c = 1.0, $CHCl_3$).

6-Fluoro-3-(1-(((1*R*,2*S*)-2-phenylcyclopropyl)methyl)piperidin-4-yl)benzo[*d*]isoxazole (3h)

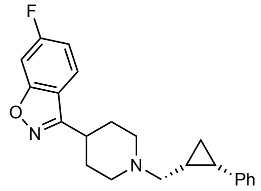

3h

General procedure G was applied to 3-(1-(cyclopropylmethyl)-piperidin-4-yl)-6-fluorobenzo[*d*]isoxazole (164 mg, 0.600 mmol) with phenyl boronic acid (36.6 mg, 0.300 mmol) at 50°C to provide by column chromatography (DCM to 3% $NH_{3(MeOH;\ 2M)}$) the title compound as a brown oil (39.9 mg, 0.114 mmol, 38% yield, >99:1 e.r.). Enantiomeric excess was determined by HPLC analysis.

IR ν_{max}/cm^{-1} (thin film) 2943, 1614, 1495, 1122, 955, 698; **^{1}H NMR** (700 MHz, $CDCl_3$) δ (ppm) 7.71 (dd, *J* = 8.6, 5.1 Hz, 1H), 7.30 (t, *J* = 7.5 Hz, 2H), 7.26 – 7.20 (m, 4H), 7.06 (td, *J* = 8.8, 1.8 Hz, 1H), 3.20 – 3.12 (m, 1H), 3.06 – 2.97 (m, 2H), 2.44 (dd, *J* = 12.8, 5.1 Hz, 1H), 2.23 (app q, *J* = 8.0 Hz, 1H), 2.11 – 1.96 (m, 6H), 1.92 (dd, *J* = 12.8, 7.8 Hz, 1H), 1.38 – 1.32 (m, 1H), 1.18 – 1.13 (m, 1H), 0.86 (app q, *J* = 5.6 Hz, 1H); **^{13}C NMR** (176 MHz, $CDCl_3$) δ (ppm) 164.1 (d, *J* = 250.5 Hz), 163.9 (d, *J* = 13.6 Hz), 161.2, 138.8, 129.0, 127.9, 125.9, 122.6 (d, *J* = 11.1 Hz), 117.3, 112.2 (d, *J* = 25.3 Hz), 97.4 (d, *J* = 26.6 Hz), 58.2, 53.6, 34.6, 30.5, 20.3, 16.5, 9.9; **^{19}F NMR** (377 MHz, $CDCl_3$) δ (ppm) –109.8 (s); **HMRS-ESI** (m/z): found $[M+H]^+$ 351.1877, $C_{22}H_{24}N_2OF$ requires 351.1873; **ee analysis:** HPLC Chiralpak AD-H (hexane(0.1% DEA):2-propanol 90:10, 1.0 mL·min^{-1}, 30 °C) t_R = 6.2 min (major), t_R = 8.2 min (minor); $[\alpha]_D^{25.0}$ = –30° (c = 1.0, $CHCl_3$).

1-(((1*R*,2*S*)-2-Phenylcyclopropyl)methyl)pyrrolidine (3i)

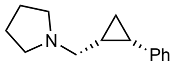

3i

General procedure G was applied to 1-(cyclopropylmethyl)pyrrolidine (94.0 mg, 0.750 mmol) with phenyl boronic acid (36.6 mg, 0.300 mmol) at 50 °C to provide by column chromatography (DCM to 3% $NH_{3(MeOH;\ 2M)}$) the title compound as a brown oil (19.2 mg, 0.095 mmol, 32% yield, >97:3 e.r.). Enantiomeric excess was determined by ^{1}H-NMR from the corresponding methylated ammonium derivative.

IR ν_{max}/cm^{-1} (thin film) 2969, 2901, 2777, 1394, 1230, 1056; **^{1}H NMR** (600 MHz, $CDCl_3$) δ (ppm) 7.30 – 7.26 (m, 2H), 7.24 – 7.17 (m, 3H), 2.50 (dd, *J* = 12.5, 4.7 Hz, 1H), 2.48 – 2.38 (m, 4H), 2.20 (dt, *J* = 8.7, 6.4 Hz, 1H), 1.81 (dd, *J* = 12.5, 8.8 Hz, 1H), 1.78 – 1.70 (m, 4H), 1.37 – 1.30 (m, 1H), 1.10 (tdd, *J* = 8.3, 5.2, 0.7 Hz, 1H), 0.85 (q, *J* = 5.7 Hz, 1H); **^{13}C NMR** (151 MHz, $CDCl_3$) δ (ppm) 139.0, 129.1, 127.9, 125.7, 55.9, 54.3, 23.4, 20.3, 17.9, 9.7; **HMRS-ESI** (m/z): found $[M+H]^+$ 202.1588, $C_{14}H_{20}N$ requires 202.1596; **ee analysis:** ^{1}H-NMR from the corresponding methylated ammonium derivative; $[\alpha]_D^{25.0}$ = –42° (c = 1.0, $CHCl_3$).

Methyl 1-(((1*R*,2*S*)-2-phenylcyclopropyl)methyl)azetidine-3-carboxylate (3j)

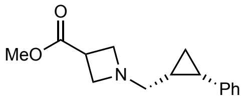

3j

General procedure G was applied to methyl 1-(cyclopropylmethyl)-azetidine-3-carboxylate (101.4 mg, 0.600 mmol) with phenyl boronic acid (36.6 mg, 0.300 mmol) at 50 °C to provide by column chromatography (DCM to 5% $NH_{3(MeOH;\ 2M)}$) the title compound as a brown oil (21.9 mg, 0.089 mmol, 30% yield, 99:1 e.r.). Enantiomeric excess was determined by HPLC analysis.

IR ν_{max}/cm^{-1} (thin film) 3060, 2834, 1732, 1436, 1197, 1172; **^{1}H NMR** (600 MHz, $CDCl_3$) δ (ppm) 7.30 – 7.26 (m, 2H), 7.23 – 7.16 (m, 3H), 3.68 (s, 3H), 3.49 – 3.42 (m, 2H), 3.28 (p, *J* = 7.6 Hz, 1H), 3.19 (t, *J* = 7.5 Hz, 1H), 3.10 (t, *J* = 7.5 Hz, 1H), 2.46 (dd, *J* = 12.3, 5.0 Hz, 1H), 2.16 (td, *J* = 8.7, 6.2 Hz, 1H), 1.79 (dd, *J* = 12.3, 8.8 Hz, 1H), 1.21 – 1.12 (m, 1H), 1.04 (tdd, *J* = 8.3, 5.2, 0.8 Hz, 1H), 0.81 (q, *J* = 5.6 Hz, 1H); **^{13}C NMR** (151 MHz, $CDCl_3$) δ (ppm) 173.5, 138.8, 129.0, 128.0, 125.9, 58.7, 57.0, 56.9, 51.8, 34.2, 19.5, 16.6, 8.9; **HMRS-ESI** (m/z): found $[M+H]^+$ 246.1482, $C_{15}H_{20}NO_2$ requires 246.1494; **ee analysis:** HPLC Chiralpak AD-H (hexane:2-propanol 97:3, 1.0 mL·min^{-1}, 30 °C) t_R = 7.8 min (major), t_R = 8.8 min (minor); $[\alpha]_D^{25.0}$ = –47° (c = 0.8, $CHCl_3$).

6-(((1*R*,2*S*)-2-phenylcyclopropyl)methyl)-2-oxa-6-azaspiro[3.3]heptane (3k)

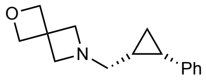

3k

General procedure G was applied to 6-(cyclopropylmethyl)-2-oxa-6-azaspiro[3.3]heptane (91.9 mg, 0.600 mmol) and phenyl boronic acid pinacol ester (61.2 mg, 0.300 mmol) at 50 °C to provide by column chromatography (DCM to 8% $NH_{3(MeOH;\ 2M)}$) the title compound as a yellow oil (21.8 mg, 0.095 mmol, 32% yield, >99:1 e.r.). Enantiomeric excess was determined by HPLC analysis.

IR ν_{max}/cm^{-1} (thin film) 2927, 2860, 2814, 1458, 1247, 970, 728, 698; **^{1}H NMR** (600 MHz, $CDCl_3$) δ (ppm) 7.30 – 7.26 (m, 2H), 7.24 – 7.16 (m, 3H), 4.72 – 4.65 (m, 4H), 3.27 (d, *J* = 7.3 Hz, 2H), 3.21 (d, *J* = 7.3 Hz, 2H), 2.37 (dd, *J* = 12.3, 5.1 Hz, 1H), 2.16 (app q, *J* = 8.0 Hz, 1H), 1.77 (dd, *J* = 12.3, 8.6 Hz, 1H), 1.14 (qt, *J* = 8.8, 5.5 Hz, 1H),

1.05 (td, *J* = 8.3, 5.3 Hz, 1H), 0.80 (q, *J* = 5.7 Hz, 1H); **^{13}C NMR** (151 MHz, $CDCl_3$) δ (ppm) 138.8, 129.0, 128.0, 125.9, 81.3, 63.8, 58.8, 39.2, 19.6, 16.8, 8.9; **HMRS-ESI** (m/z): found $[M+H]^+$ 230.1543, $C_{15}H_{20}NO$ requires 230.1545; **ee analysis:** HPLC Chiralpak AD-H (hexane(0.1% DEA):2-propanol 97:3, 1.0 mL·min^{-1}, 30 °C) t_R = 9.4 min (major), t_R = 11.0 min (minor); $[\alpha]_D^{25.0}$ = –54° (c = 0.7, $CHCl_3$).

Ethyl 3-(methyl(((1*R*,2*S*)-2-phenylcyclopropyl)methyl)amino)propanoate (3m)

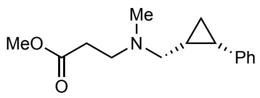

3m

General procedure G was applied to ethyl 3-((cyclopropylmethyl)-(methyl)amino)-propanoate (111 mg, 0.600 mmol) with phenyl boronic acid (36.6 mg, 0.300 mmol) at 60 °C to provide by column chromatography (DCM to 5% $NH_{3(MeOH;\ 2M)}$) the title compound as a brown oil (50.8 mg, 0.194 mmol, 65% yield, 96:4 e.r.). Enantiomeric excess was determined by HPLC analysis.

IR ν_{max}/cm^{-1} (thin film) 2980, 2772, 1731, 1459, 1179, 1028; **^{1}H NMR** (600 MHz, $CDCl_3$) δ (ppm) 7.31 – 7.26 (m, 2H), 7.23 – 7.17 (m, 3H), 4.12 (q, *J* = 7.1 Hz, 2H), 2.64 (t, *J* = 7.4 Hz, 2H), 2.41 – 2.30 (m, 3H), 2.24 – 2.16 (m, 4H), 1.83 (dd, *J* = 12.9, 7.9 Hz, 1H), 1.31 – 1.23 (m, 4H), 1.12 (td, *J* = 8.4, 5.3 Hz, 1H), 0.82 (q, *J* = 5.8 Hz, 1H); **^{13}C NMR** (151 MHz, $CDCl_3$) δ (ppm) 172.7, 138.8, 129.0, 128.0, 125.8, 60.4, 56.7, 52.5, 42.1, 32.4, 20.4, 16.9, 14.2, 9.6; **HMRS-ESI** (m/z): found $[M+H]^+$ 262.1794, $C_{16}H_{24}NO_2$ requires 262.1807; **ee analysis:** HPLC Chiralpak AD-H (hexane:2-propanol 99.5:0.5, 1.0 mL·min^{-1}, 30 °C) t_R = 13.1 min (major), t_R = 17.2 min (minor); $[\alpha]_D^{25.0}$ = –36° (c = 1.0, $CHCl_3$).

Benzyl 3-(methyl(((1*R*,2*S*)-2-phenylcyclopropyl)methyl)amino)azetidine-1-carboxylate (3n)

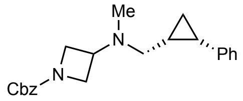

3n

General procedure G was applied to benzyl 3-((cyclopropylmethyl)(methyl)amino)-azetidine-1-carboxylate (164.6 mg, 0.600 mmol) with phenyl boronic acid (36.6 mg, 0.300 mmol) at 50 °C to provide by column chromatography (DCM to 5% $NH_{3(MeOH;\ 2M)}$) the title compound as a brown oil (48.1 mg, 0.138 mmol, 46% yield, 92:8 e.r.). Enantiomeric excess was determined by HPLC analysis.

IR ν_{max}/cm^{-1} (thin film) 3029, 2955, 1704, 1415, 1351, 1127; **^{1}H NMR** (600 MHz, $CDCl_3$) δ (ppm) 7.40 – 7.31 (m, 5H), 7.29 – 7.24 (m, 2H), 7.18 (app br d, *J* = 7.7 Hz, 3H), 5.09 (s, 2H), 3.87 (t, *J* = 7.9 Hz, 1H), 3.81 (t, *J* = 7.9 Hz, 1H), 3.76 – 3.72 (m, 1H), 3.67 (br s, 1H), 3.08 (br s, 1H), 2.26 – 2.15 (m, 2H), 2.13 (s, 3H), 1.78 (dd, *J* = 13.0, 7.5 Hz, 1H), 1.29 – 1.21 (m, 1H), 1.11 (td, *J* = 8.4, 5.5 Hz, 1H), 0.80 (q, *J* = 5.7 Hz, 1H); **^{13}C NMR** (151 MHz, $CDCl_3$) δ (ppm) 156.4, 138.3, 136.7, 128.9, 128.4, 128.0, 128.0, 127.9, 126.0, 66.5, 54.1, 53.8, 38.1, 20.6, 16.4, 9.3; **HMRS-ESI** (m/z): found $[M+H]^+$ 351.2060, $C_{22}H_{27}N_2O_2$ requires 351.2073; **ee analysis:** HPLC Chiralpak AD-H (hexane:2-propanol 97:3, 1.0 mL·min^{-1}, 30 °C) t_R = 20.3 min (major), t_R = 23.1 min (minor); $[\alpha]_D^{25.0}$ = –39° (c = 0.7, $CHCl_3$).

2-((5-Fluoropyrimidin-2-yl)oxy)-*N*-methyl-*N*-(((1*R*,2*S*)-2-phenylcyclopropyl)methyl)ethan-1-amine (3o)

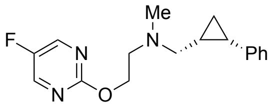

3o

General procedure G was applied to *N*-(cyclopropylmethyl)-2-((5-fluoropyrimidin-2-yl)oxy)-*N*-methylethan-1-amine (135.2 mg, 0.600 mmol) with phenyl boronic acid (36.6 mg, 0.300 mmol) in NMP at 50 °C to provide by column chromatography (DCM to 6% $NH_{3(MeOH;\ 2M)}$) the title compound as a brown oil (43.3 mg, 0.144 mmol, 48% yield, 89:11 e.r.). Enantiomeric excess was determined by HPLC analysis.

IR ν_{max}/cm^{-1} (thin film) 3077, 2774, 1570, 1426, 1325, 1242, 1029; **^{1}H NMR** (400 MHz, $CDCl_3$) δ (ppm) 8.36 (s, 2H), 7.28 – 7.23 (m, 2H), 7.23 – 7.13 (m, 3H), 4.34 (t, *J* = 6.2 Hz, 2H), 2.76 (t, *J* = 6.2 Hz, 2H), 2.44 (dd, *J* = 12.9, 5.5 Hz, 1H), 2.30 (s, 3H), 2.21 (td, *J* = 8.7, 6.3 Hz, 1H), 1.97 (dd, *J* = 13.0, 7.6 Hz, 1H), 1.36 – 1.26 (m, 1H), 1.12 (td, *J* = 8.4, 5.2 Hz, 1H), 0.84 (q, *J* = 5.7 Hz, 1H); **^{13}C NMR** (151 MHz, $CDCl_3$) δ (ppm) 161.4 (d, *J* = 0.8 Hz), 154.2 (d, *J* = 253.4 Hz), 146.5 (d, *J* = 22.6 Hz), 138.8, 129.0, 127.9, 125.8, 66.3, 57.3, 55.5, 42.8, 20.4, 16.9, 9.6; **^{19}F NMR** (377 MHz, $CDCl_3$) δ (ppm) –150.5 (s); **HMRS-ESI** (m/z): found $[M+H]^+$ 302.1660, $C_{17}H_{21}N_3OF$ requires 302.1669; **ee analysis:** HPLC Chiralpak AD-H (hexane:2-propanol 98:2, 1.0 mL·min^{-1}, 30 °C) t_R = 13.0 min (major), t_R = 14.4 min (minor); $[\alpha]_D^{25.0}$ = –29° (c = 1.0, $CHCl_3$).

***N*-ethyl-*N*-(((1*R*,2*S*)-2-phenylcyclopropyl)methyl)ethanamine (3p)**

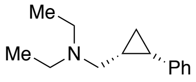

3p

General procedure G was applied to *N*-(cyclopropylmethyl)-*N*-ethylethanamine (76.4 mg, 0.600 mmol) with phenyl boronic acid (36.6 mg, 0.300 mmol) at 50 °C to provide by column chromatography (DCM to 5% $NH_{3(MeOH;\ 2M)}$) the title compound as a yellow oil (43.3 mg, 0.213 mmol, 71% yield, 98.5:1.5 e.r.). Enantiomeric excess was determined by HPLC analysis.

IR ν_{max}/cm^{-1} (thin film) 2976, 2802, 1507, 1431, 1233, 1068, 832; **^{1}H NMR** (400 MHz, $CDCl_3$) δ (ppm) 7.46 (d, *J* = 8.2 Hz, 2H), 7.43 – 7.36 (m, 3H), 2.72 (q, *J* = 8.0 Hz, 4H), 1.70 (dd, *J* = 12.7, 8.0 Hz, 1H), 1.39 – 1.32 (m, 1H) 1.20 – 1.16 (m, 1H), 0.85 (app q, *J* = 5.7 Hz, 1H); **^{13}C NMR** (101 MHz, $CDCl_3$) δ (ppm) 138.0, 131.0, 125.4, 119.6 59.0, 45.4, 19.9, 17.1, 16.5, 9.8; **HMRS-ESI** (m/z): found $[M+H]^+$ 204.1749, $C_{14}H_{21}N$ requires 204.1752; **ee analysis:** HPLC Chiralpak AD-H (hexane:2-propanol 98:2, 1.0 mL·min^{-1}, 30 °C) t_R = 16.3 min (major), t_R = 19.2 min (minor); $[\alpha]_D^{25.0}$ = –41° (c = 1.0, $CHCl_3$).

***N*-methyl-*N*-(((1*R*,2*S*)-2-phenylcyclopropyl)methyl)propan-2-amine (3q)**

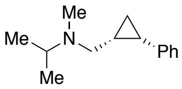

3q

General procedure G was applied to *N*-(cyclopropylmethyl)-*N*-ethylethanamine (76.4 mg, 0.600 mmol) with phenyl boronic acid (36.6 mg, 0.300 mmol) at 50 °C to provide by column chromatography (DCM to 5% $NH_{3(MeOH;\ 2M)}$) the title compound as a brown oil (25.6 mg, 0.126 mmol, 42% yield, 98:2 e.r.). Enantiomeric excess was determined by HPLC analysis.

IR ν_{max}/cm^{-1} (thin film) 2971, 2982, 1511, 1412, 1221, 1123, 1063, 845; **^{1}H NMR** (400 MHz, $CDCl_3$) δ (ppm) 7.29 – 7.26 (m, 2H), 7.22 – 7.18 (m, 3H), 2.82 – 2.77 (m, 1H), 2.51 (s, 4H), 2.31 (dd, *J* = 12.6, 7.4 Hz, 1H), 1.73 (dd, *J*

= 12.7, 7.5 Hz, 1H), 1.31 – 1.26 (m, 1H) 1.18 – 1.11 (m, 7H), 0.82 (app q, *J* = 6.1 Hz, 1H);); **^{13}C NMR** (101 MHz, $CDCl_3$) δ (ppm) 137.6, 130.9, 125.6, 120.0, 59.2, 54.9, 43.9, 23.8, 19.7, 17.0, 9.8; **HMRS-ESI** (m/z): found $[M+H]^+$ 204.1750, $C_{14}H_{21}N$ requires 204.1752; **ee analysis:** HPLC Chiralpak AD-H (hexane:2-propanol 98:2, 0.5 mL·min^{-1}, 45 °C) t_R = 18.9 min (major), t_R = 20.7 min (minor); $[\alpha]_D^{25.0}$ = –45° (c = 1.0, $CHCl_3$).

1-((1*S*,2*R*)-1-(2-Methoxyethyl)-2-phenylcyclopropyl)-*N*,*N*-dimethylmethanamine (3r)

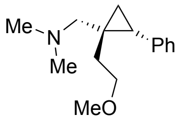

3r

General procedure G was applied to 1-(1-(2-methoxyethyl)cyclopropyl)-N,N-dimethylmethanamine (94.4 mg, 0.600 mmol) with phenyl boronic acid (36.6 mg, 0.300 mmol) at 50 °C to provide by column chromatography (DCM to 3% $NH_{3(MeOH;\ 2M)}$) the title compound as a yellow oil (30.7 mg, 0.132 mmol, 44% yield, 87.5:12.5 e.r.). Enantiomeric excess was determined by ^{1}H-NMR from the corresponding methylated ammonium derivative.

IR ν_{max}/cm^{-1} (thin film) 2936, 2813, 1456, 1263, 1115, 1030, 728, 697; **^{1}H NMR** (700 MHz, $CDCl_3$) δ (ppm) 7.28 (t, *J* = 7.2 Hz, 2H), 7.23 – 7.17 (m, 3H), 3.67 – 3.58 (m, 2H), 3.42 (s, 3H), 2.40 (d, *J* = 12.8 Hz, 1H), 2.22 – 2.16 (m, 1H), 2.11 (s, 6H), 2.04 (t, *J* = 7.3 Hz, 1H), 1.34 (dt, *J* = 13.9, 7.1 Hz, 1H), 1.26 (d, *J* = 12.8 Hz, 1H), 1.01 (t, *J* = 5.4 Hz, 1H), 0.96 – 0.92 (m, 1H); **^{13}C NMR** (176 MHz, $CDCl_3$) δ (ppm) 139.0, 129.0, 127.8, 125.7, 71.1, 61.1, 58.7, 45.7, 36.3, 26.8, 22.6, 17.2; **HMRS-ESI** (m/z): found $[M+H]^+$ 234.1853, $C_{15}H_{24}NO$ requires 234.1858; **ee analysis:** ^{1}H-NMR (H_6) from the corresponding methylated ammonium derivative; $[\alpha]_D^{25.0}$ = +13° (c = 0.5, $CHCl_3$).

***N*,*N*-Dimethyl-1-((1*R*,2*R*)-1-methyl-2-phenylcyclopropyl)methanamine (3s)**

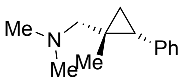

3s

General procedure G was applied to *N*,*N*-dimethyl-1-(1-methylcyclopropyl)methanamine (102 mg, 0.900 mmol) with phenyl boronic acid (36.6 mg, 0.300 mmol) at 50 °C to provide by column chromatography (DCM to 2% $NH_{3(MeOH;\ 2M)}$) the title compound as a yellow oil (22.6 mg, 0.119 mmol, 40% yield, 89:11 e.r.). Enantiomeric excess was determined by GC-FID analysis.

IR ν_{max}/cm^{-1} (thin film) 2942, 2812, 2761, 1455, 1265, 1026, 857, 696; **^{1}H NMR** (700 MHz, $CDCl_3$) δ (ppm) 7.30 – 7.25 (m, 2H), 7.22 – 7.16 (m, 3H), 2.30 (d, *J* = 12.5 Hz, 1H), 2.11 (s, 6H), 1.96 – 1.91 (m, 1H), 1.52 (d, *J* = 12.5 Hz, 1H), 1.30 (s, 3H), 1.02 (app t, *J* = 5.6 Hz, 1H), 0.92 (dd, *J* = 8.4, 5.0 Hz, 1H); **^{13}C NMR** (176 MHz, $CDCl_3$) δ (ppm) 139.2, 128.9, 127.8, 125.6, 63.5, 45.6, 28.6, 24.0, 21.4, 17.8; **HMRS-ESI** (m/z): found $[M+H]^+$ 190.1591, $C_{13}H_{20}N$ requires 190.1596; **ee analysis:** GC-FID ChiralDex β-DM (50 °C to 150 °C; 5 °C/min; linear velocity 40 cm·s^{-1}; split ratio 10.0) t_R = 9.0 min (minor), t_R = 9.2 min (major); $[\alpha]_D^{25.0}$ = +7.4° (c = 0.4, $CHCl_3$).

1-(1-Benzylcyclopropyl)-*N*,*N*-dimethylmethanamine (4a)

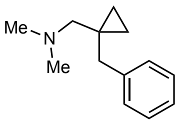

4a

General procedure G was applied to *N*,*N*-dimethyl-1-(1-methylcyclopropyl)methanamine (102 mg, 0.900 mmol) with phenyl boronic acid (36.6 mg, 0.300 mmol) at 50 °C to provide by column chromatography (DCM to 2% $NH_{3(MeOH;\ 2M)}$) the title compound as an inseparable

mixture with 1-(1-([1,1'-biphenyl]-2-ylmethyl)-cyclopropyl)-*N*,*N*-dimethylmethan-amine (9.0 mg, **4**:1 ratio (major), 0.035 mmol, 12% yield).

^{1}H NMR (700 MHz, $CDCl_3$) δ (ppm) 7.32 – 7.17 (m, 5H), 4.96 (s, 1H), 4.91 (s, 1H), 2.86 (s, 2H), 2.83 – 2.79 (app t, *J* = 8.0 Hz, 2H), 2.44 – 2.39 (app t, *J* = 8.0 Hz, 2H), 2.21 (s, 6H); **^{13}C NMR** (176 MHz, $CDCl_3$) δ (ppm) 146.6, 142.2, 128.4, 128.2, 125.7, 112.4, 65.8, 45.5, 35.6, 34.1; **HMRS-ESI** (m/z): found $[M+H]^+$ 176.1435, $C_{12}H_{18}N$ requires 176.1434.

1-(1-([1,1'-Biphenyl]-2-ylmethyl)cyclopropyl)-*N*,*N*-dimethylmethanamine (4aa)

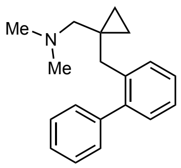

4aa

General procedure G was applied to *N*,*N*-dimethyl-1-(1-methylcyclopropyl)methan-amine (102 mg, 0.900 mmol) with phenyl boronic acid (36.6 mg, 0.300 mmol) at 50 °C to provide by column chromatography (DCM to 2% $NH_{3(MeOH;\ 2M)}$) the title compound as an inseparable mixture with 1-(1-benzylcyclo-propyl)-*N*,*N*-dimethyl-methanamine (9.0 mg, 4:**1** ratio (minor), 0.009 mmol, 3% yield).

^{1}H NMR (700 MHz, $CDCl_3$) δ (ppm) 7.32 – 7.17 (m, 9H), 2.70 (s, 2H), 2.27 (s, 6H), 2.01 (s, 2H), 0.49 – 0.45 (m, 2H), 0.31 – 0.27 (m, 2H); **HMRS-ESI** (m/z): found $[M+H]^+$ 252.1738, $C_{18}H_{22}N$ requires 252.1747.

1-((1*R*,2*R*)-1-(4-Chlorophenyl)-2-phenylcyclopropyl)-*N*,*N*-dimethylmethanamine (3t)

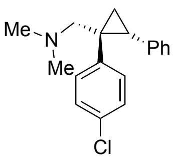

3t

General procedure G was applied to 1-(1-(4-chlorophenyl)cyclopropyl)-*N,N*-dimethyl-methanamine (126.0 mg, 0.600 mmol) with phenyl boronic acid (36.6 mg, 0.300 mmol) at 50 °C to provide by column chromatography (DCM to 3% $NH_{3(MeOH;\ 2M)}$) the title compound as a brown oil (18.2 mg, 0.064 mmol, 22% yield, 96:4 e.r.). Enantiomeric excess was determined by HPLC analysis.

IR ν_{max}/cm^{-1} (thin film) 2968, 2815, 2764, 1493, 1456, 1093; **^{1}H NMR** (600 MHz, $CDCl_3$) δ (ppm) 7.39 – 7.36 (m, 2H), 7.36 – 7.33 (m, 2H), 7.33 – 7.31 (m, 2H), 7.31 – 7.28 (m, 2H), 7.27 – 7.24 (m, 1H), 2.78 (dd, *J* = 13.0, 1.5 Hz, 1H), 2.22 (dd, *J* = 8.8, 6.7 Hz, 1H), 2.07 (s, 6H), 1.68 (ddd, *J* = 8.8, 5.5, 1.5 Hz, 1H), 1.65 (d, *J* = 13.1 Hz, 1H), 1.44 (dd, *J* = 6.7, 5.5 Hz, 1H); **^{13}C NMR** (151 MHz, $CDCl_3$) δ (ppm) 144.1, 137.8, 131.7, 129.4, 129.0, 128.5, 128.2, 126.3, 64.0, 45.8, 30.3, 30.0, 16.5; **HMRS-ESI** (m/z): found $[M+H]^+$ 286.1354, $C_{18}H_{21}NCl$ requires 286.1363; **ee analysis:** HPLC Chiralpak AD-H (hexane:2-propanol 99:1, 1.0 mL·min^{-1}, 30 °C) t_R = 8.5 min (major), t_R = 12.4 min (minor); $[\alpha]_D^{25.0}$ = +84° (c = 1.0, $CHCl_3$).

1-(1-(5-Chloro-[1,1'-biphenyl]-2-yl)cyclopropyl)-*N,N*-dimethylmethanamine (4b)

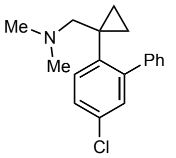

4b

General procedure G was applied to 1-(1-(4-chlorophenyl)cyclopropyl)-*N,N*-dimethyl-methanamine (126.0 mg, 0.600 mmol) with phenyl boronic acid (36.6 mg, 0.300 mmol) at 50

°C to provide by column chromatography (DCM to 3% $NH_{3(MeOH;\ 2M)}$) the title compound as a pale oil (42.9 mg, 0.150 mmol, 50% yield).

IR ν_{max}/cm^{-1} (thin film) 2941, 2811, 2763, 1461, 1267, 1113, 1034; **^{1}H NMR** (600 MHz, $CDCl_3$) δ (ppm) 7.45 – 7.41 (m, 2H), 7.41 – 7.36 (m, 3H), 7.31 (d, *J* = 8.3 Hz, 1H), 7.26 (dd, *J* = 8.3, 2.3 Hz, 1H), 7.17 (d, *J* = 2.3 Hz, 1H), 2.08 (s, 6H), 1.91 (s, 2H), 0.87 (app q, *J* = 4.5 Hz, 2H), 0.55 (app q, *J* = 4.5 Hz, 2H); **^{13}C NMR** (151 MHz, $CDCl_3$) δ (ppm) 145.1, 141.6, 139.9, 131.8, 131.7, 130.6, 129.3, 127.8, 127.1, 127.1, 67.6, 46.0, 23.1, 13.3; **HMRS-ESI** (m/z): found $[M+H]^+$ 286.1353, $C_{18}H_{21}NCl$ requires 286.1363.

1-((1*R*,2*R*)-1-(5-Chloro-[1,1'-biphenyl]-2-yl)-2-phenylcyclopropyl)-*N,N*-dimethylmethanamine (4ba)

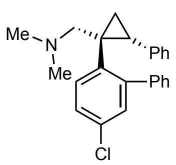

4ba

General procedure G was applied to 1-(1-(4-chlorophenyl)cyclopropyl)-*N,N*-dimethylmethanamine (126 mg, 0.600 mmol) with phenyl boronic acid (36.6 mg, 0.300 mmol) at 50 °C to provide by column chromatography (DCM to 3% $NH_{3(MeOH;\ 2M)}$) the title compound (13.3 mg, 0.036 mmol, 12% yield).

IR ν_{max}/cm^{-1} (thin film) 3061, 2941, 2813, 2761, 1587, 1461, 1038; **^{1}H NMR** (600 MHz, $CDCl_3$) δ (ppm) 7.50 (d, *J* = 8.4 Hz, 1H), 7.46 – 7.41 (m, 3H), 7.41 – 7.36 (m, 2H), 7.32 (dd, *J* = 8.4, 2.4 Hz, 1H), 7.17 (d, *J* = 2.3 Hz, 1H), 7.16 – 7.10 (m, 3H), 6.61 (d, *J* = 6.4 Hz, 2H), 2.38 (d, *J* = 13.2 Hz, 1H), 2.19 (dd, *J* = 8.5, 7.1 Hz, 1H), 2.03 (s, 6H), 1.65 (d, *J* = 13.2 Hz, 1H), 1.43 (dd, *J* = 8.9, 5.0 Hz, 1H), 1.31 – 1.27 (m, 1H); **^{13}C NMR** (151 MHz, $CDCl_3$) δ (ppm) 145.0, 141.7, 141.6, 137.8, 132.6, 131.7, 130.8, 129.4, 129.1, 128.2, 127.6, 127.3, 127.2, 125.8, 63.8, 46.0, 30.6, 29.5, 18.4; **HMRS-ESI** (m/z): found $[M+H]^+$ 362.1669, $C_{24}H_{25}NCl$ requires 362.1676.

Ethyl 4-((1*S*,2*R*)-2-((dimethylamino)methyl)cyclopropyl)benzoate (3aa)

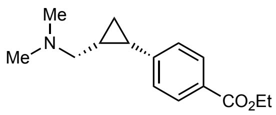

3aa

General procedure G was applied to 1-cyclopropyl-*N,N*-dimethylmethanamine (59.6 mg, 0.600 mmol) with (4-(ethoxycarbonyl)phenyl)-boronic acid (58.2 mg, 0.300 mmol) in DMA (1.5 mL) at 40˚C for 24 hours to provide the title compound as a brown oil (49.0 mg, 0.198 mmol, 66% yield, >99:1 e.r.). Enantiomeric excess was determined by HPLC analysis.

IR ν_{max}/cm^{-1} (thin film) 2979, 2764, 1712, 1271, 1101, 1020, 706; **^{1}H NMR** (600 MHz, $CDCl_3$) δ (ppm) 7.94 (d, *J* = 8.2 Hz, 2H), 7.24 (d, *J* = 8.2 Hz, 2H), 4.36 (q, *J* = 7.1 Hz, 2H), 2.30 (dd, *J* = 12.8, 5.4 Hz, 1H), 2.27-2.23 (m, 1H), 2.20 (s, 6H), 1.81 (dd, *J* = 12.8, 8.0 Hz, 1H), 1.44-1.37 (m, 4H), 1.21-1.18 (m, 1H), 0.92 (app q, *J* = 5.8 Hz, 1H); **^{13}C NMR** (151 MHz, $CDCl_3$) δ (ppm) 166.6, 144.2, 129.2, 128.8, 128.2, 60.8, 58.5, 45.0, 20.7, 17.5, 14.3, 10.0; **HRMS-ESI** (m/z): found $[M+H]^+$ 248.1676, $C_{15}H_{22}NO_2$ requires 248.1651. **ee analysis:** HPLC Chiralpak AD-H (hexane(0.1% DEA):2-propanol 99:1, 1.0 mL·min^{-1}, 30°C), t_R = 17.2 min (major), tR = 19.7 min (minor); $[\alpha]_D^{25.0}$ = –68° (c = 1.0, $CHCl_3$).

1-(4-((1*S*,2*R*)-2-((dimethylamino)methyl)cyclopropyl)phenyl)ethan-1-one (3ab)

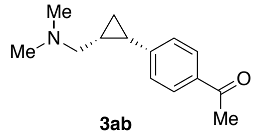

3ab

General procedure G was applied to 1-cyclopropyl-*N,N*-dimethylmethanamine (59.6 mg, 0.600 mmol) with (4-acetylphenyl)boronic acid (49.2 mg, 0.300 mmol) in DMA (1.5 mL) at 40˚C for 24 hours to provide the title compound as a brown oil (44.3 mg, 0.204 mmol, 68% yield, 99:1 e.r.). Enantiomeric excess was determined by HPLC analysis.

IR ν_{max}/cm^{-1} (thin film) 2946, 2713, 1691, 1587, 1089, 1068, 856; **^{1}H NMR** (700 MHz, $CDCl_3$) δ (ppm) 8.03 (d, *J* = 8.0 Hz, 2H), 7.33 (d, *J* = 8.0 Hz, 2H), 2.61 (s, 3H), 2.42-2.38 (m, 1H), 2.37-2.33 (m, 1H), 2.29 (s, 6H), 1.91 (dd, *J* = 12.8, 8.0 Hz, 1H), 1.52-1.45 (m, 1H), 1.31-1.26 (m, 1H), 1.01 (app q, *J* = 5.7 Hz, 1H); **^{13}C NMR** (151 MHz, $CDCl_3$) δ (ppm) 186.5, 152.6, 144.2, 129.2, 116.9, 58.5, 45.0, 26.5, 20.7, 17.4, 10.0; **HRMS-ESI** (m/z): found $[M+H]^+$ 218.1536, $C_{14}H_{20}NO$ requires 218.1539; **ee analysis:** HPLC Chiralpak AD-H (hexane(0.1% DEA):2-propanol 99.5:0.5, 1.0 mL·min^{-1}, 30°C), t_R = 7.8 min (major), t_R = 9.0 min (minor); $[\alpha]_D^{25.0}$ = –49° (c = 1.0, $CHCl_3$).

1-((1*R*,2*S*)-2-(4-Bromophenyl)cyclopropyl)-*N,N*-dimethylmethanamine (3ac)

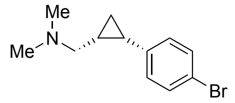

3ac

General procedure G was applied to 1-cyclopropyl-*N,N*-dimethylmethanamine (59.6 mg, 0.600 mmol) with (4-bromophenyl)boronic acid (60.2 mg, 0.300 mmol) in DMA (1.5 mL) at 40˚C for 24 hours to provide the title compound as a brown oil (55.8 mg, 0.219 mmol, 73% yield, >99:1 e.r.). Enantiomeric excess was determined by HPLC analysis.

IR ν_{max}/cm^{-1} (thin film) 2940, 2764, 1489, 1073, 1009, 819, 731; **^{1}H NMR** (700 MHz, $CDCl_3$) δ (ppm) 7.40 (d, *J* = 8.3 Hz, 2H), 7.08 (d, *J* = 8.3 Hz, 2H), 2.27 (dd, *J* = 12.7, 5.2 Hz, 1H), 2.20 (s, 6H), 2.17-2.13 (m, 1H), 1.69 (dd, *J* = 12.7, 8.2 Hz, 1H), 1.32-1.28 (m, 1H), 1.16-1.13 (m, 1H), 0.79 (app q, *J* = 5.8 Hz, 1H); **^{13}C NMR** (177 MHz, $CDCl_3$) δ (ppm) 138.0, 131.0, 130.8, 119.6, 59.0, 45.5, 19.9, 17.1, 9.8; **HRMS-ESI** (m/z): found $[M+H]^+$ 254.0538, $C_{12}H_{17}NBr$ requires 254.0544; **ee analysis:** HPLC Chiralpak AD-H (hexane(0.1% DEA):2-propanol 98:2, 1.0 mL·min^{-1}, 30 °C), t_R = 5.3 min (major), t_R = 6.1 min (minor); $[\alpha]_D^{25.0}$ = –79° (c = 1.0, $CHCl_3$).

1-((1*R*,2*S*)-2-(4-Fluorophenyl)cyclopropyl)-*N,N*-dimethylmethanamine (3ad)

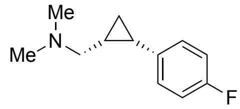

3ad

General procedure G was applied to 1-cyclopropyl-*N,N*-dimethylmethanamine (59.6 mg, 0.600 mmol) with (4-fluorophenyl)boronic acid (42.0 mg, 0.300 mmol) in DMA (1.5 mL) at 40˚C for 24 hours to provide the title compound as a yellow oil (43.4 mg, 0.225 mmol, 75% yield, >99:1 e.r.). Enantiomeric excess was determined by HPLC analysis.

IR ν_{max}/cm^{-1} (thin film) 2941, 2764, 1509, 1218, 1032, 832; **^{1}H NMR** (700 MHz, $CDCl_3$) δ (ppm) 7.16 (app t, *J* = 6.8 Hz, 2H), 6.96 (t, *J* = 8.3 Hz, 2H), 2.26 (dd, *J* = 12.6, 4.6 Hz, 1H) 2.17-2.14 (m, 7H), 1.64 (dd, 12.6, 7.9 Hz, 1H), 1.28-1.23 (m, 1H), 1.13-1.10 (m, 1H), 0.74 (app q, *J* = 5.5 Hz, 1H); **^{13}C NMR** (177 MHz, $CDCl_3$) δ (ppm) 161.3 (d, J_{C-F} = 245 Hz), 134.5, 134.5, 114.7, 59.1, 45.5, 19.5, 16.9, 9.8; **HRMS-ESI** (m/z): found $[M+H]^+$ 194.1349, $C_{12}H_{17}NF$

requires 194.1345; **ee analysis:** HPLC Chiralpak AD-H (hexane(0.1% DEA):2-propanol 98:2, 1.0 mL·min^{-1}, 30°C), t_R = 5.1 min (major), t_R = 6.8 min (minor); $[\alpha]_D^{25.0}$ = –53° (c = 1.0, $CHCl_3$).

tert-Butyl (4-((1_S_,2_R_)-2-((dimethylamino)methyl)cyclopropyl)phenyl)carbamate (3ae)

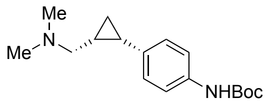

3ae

General procedure G was applied to 1-cyclopropyl-*N,N*-dimethylmethanamine (59.6 mg, 0.600 mmol) with (4-((*tert*-butoxycarbonyl)amino)-phenyl)boronic acid (71.1 mg, 0.300 mmol) in DMA (1.5 mL) at 50˚C to provide the title compound as a brown solid (54.9 mg, 0.189 mmol, 63% yield, >99:1 e.r.). Enantiomeric excess was determined by HPLC analysis.

mp (˚C) 96-99 ; **IR** ν_{max}/cm^{-1} (thin film) 2967, 1661, 1537, 1488, 921, 699; **^{1}H NMR** (700 MHz, $CDCl_3$) δ (ppm) 7.42 (s, 1H), 7.39 (m, *J* = 8.3 Hz, 2H), 7.22 (app t, *J* = 7.8 Hz, 1H), 6.94 (d, *J* = 7.6 Hz, 1H), 2.41 (dd, *J* = 12.8, 5.1 Hz, 1H), 2.24 (s, 6H), 2.21-2.18 (m, 1H), 1.78 (dd, *J* = 12.8, 8.4 Hz, 1H), 1.36-1.29 (m, 10H), 1.14-1.11 (m, 1H), 0.87 (app q, *J* = 5.7 Hz, 1H); **^{13}C NMR** (177 MHz, $CDCl_3$) δ (ppm) 176.7, 139.6, 137.9, 128.5, 124.8, 120.7, 117.7, 58.8, 45.1, 39.6, 27.7, 20.4, 16.6, 9.8; **HRMS-ESI** (m/z): found $[M+H]^+$ 291.2076, $C_{17}H_{27}N_2O_2$ requires 291.2073; **ee analysis:** HPLC Chiralpak AD-H (hexane(0.1% DEA):2-propanol 98:2, 1.0 mL·min^{-1}, 30 °C), t_R = 28.4 min (major), t_R = 31.2 min (minor); $[\alpha]_D^{25.0}$ = –86° (c = 1.0, $CHCl_3$).

4-((1_S_,2_R_)-2-((dimethylamino)methyl)cyclopropyl)-_N,N_-diphenylaniline (3af)

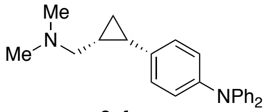

3af

General procedure G was applied to 1-cyclopropyl-*N,N*-dimethylmethanamine (59.6 mg, 0.600 mmol) with (4-(diphenylamino)phenyl)boronic acid (86.7 mg, 0.300 mmol) in DMA (1.5 mL) at 40˚C for 24 hours to provide the title compound as a brown oil (64.0 mg, 0.187 mmol, 62% yield, 99:1 e.r.). Enantiomeric excess was determined by HPLC analysis.

IR ν_{max}/cm^{-1} (thin film) 2967, 2754, 1523, 1178, 1076, 810; **^{1}H NMR** (400 MHz, $CDCl_3$) δ (ppm) 7.49-7.41 (m, 4H), 7.36-7.15 (m, 8H), 2.38-2.32 (m, 2H), 2.18 (s, 6H), 1.73 (dd, *J* = 12.7, 8.2 Hz, 1H), 1.42-1.33 (m, 1H), 1.23-1.17 (m, 1H), 0.98 (app q, *J* = 5.6 Hz, 1H); **^{13}C NMR** (101 MHz, $CDCl_3$) δ (ppm) 149.7, 135.2, 132.5, 131.1, 124.5, 113.9, 59.3, 45.7, 19.7, 17.1, 10.0; **HRMS-ESI** (m/z): found $[M+H]^+$ 313.2176, $C_{24}H_{27}N_2$ requires 313.2174; **ee analysis:** HPLC Chiralpak AD-H (hexane(0.1% DEA):2-propanol 97:3, 1.0 mL·min^{-1}, 30°C), t_R = 17.1 min (major), t_R = 19.2 min (minor); $[\alpha]_D^{25.0}$ = –56° (c = 1.0, $CHCl_3$).

1-((1_R_,2_S_)-2-(4-Methoxyphenyl)cyclopropyl)-_N,N_-dimethylmethanamine (3ag)

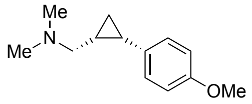

3ag

General procedure G was applied to 1-cyclopropyl-*N,N*-dimethylmethanamine (59.6 mg, 0.600 mmol) with (4-methoxyphenyl)boronic acid (45.6 mg, 0.300 mmol) in DMA (1.5 mL) at 40˚C for 24 hours to provide the title compound as a brown oil (43.2 mg, 0.210 mmol, 70% yield, 99:1 e.r.). Enantiomeric excess was determined by HPLC analysis.

IR ν_{max}/cm^{-1} (thin film) 2939, 2762, 1512, 1456, 1243, 1031, 828, 797; **^{1}H NMR** (600 MHz, $CDCl_3$) δ (ppm) 7.12 (d, *J* = 8.4 Hz, 2H), 6.82 (d, *J* = 8.4 Hz, 2H), 3.80 (s, 3H), 2.29 (dd, *J* = 12.7, 5.0 Hz, 1H), 2.18 (s, 6H), 2.15-2.11 (m, 1H), 1.65 (dd, *J* = 12.7, 8.3 Hz, 1H), 1.25-1.19 (m, 1H), 1.10-1.06 (m, 1H), 0.72 (app q, *J* = 5.7 Hz, 1H); **^{13}C NMR** (151 MHz, $CDCl_3$) δ (ppm) 157.8, 130.8, 130.0, 113.4, 59.2, 55.2, 45.5, 19.4, 16.7, 9.7; **HRMS-ESI** (m/z): found $[M+H]^+$ 206.1548, $C_{13}H_{20}NO$ requires 206.1545. **ee analysis:** HPLC Chiralpak AD-H (hexane(0.1% DEA):2-propanol 98:2, 1.0 mL·min^{-1}, 30°C), t_R = 7.3 min (major), t_R = 7.9 min (minor); $[\alpha]_D^{25.0}$ = –65° (c = 1.0, $CHCl_3$).

***N,N*-Dimethyl-1-((1*R*,2*S*)-2-(3-nitrophenyl)cyclopropyl)methanamine (3ah)**

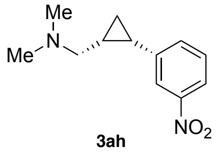

3ah

General procedure G was applied to 1-cyclopropyl-*N,N*-dimethylmethanamine (59.6 mg, 0.600 mmol) with (3-nitrophenyl)boronic acid (50.1 mg, 0.300 mmol) in DMA (1.5 mL) at 30˚C for 40 hours to provide the title compound as a yellow oil (42.4 mg, 0.192 mmol, 64% yield, 99:1 e.r.). Enantiomeric excess was determined by HPLC analysis.

IR ν_{max}/cm^{-1} (thin film) 2940, 2764, 1525, 1341, 1032, 735, 689; **^{1}H NMR** (600 MHz, $CDCl_3$) δ (ppm) 8.05-8.04 (m, 2H), 7.55 (d, *J* = 7.5 Hz, 1H), 7.44 (app t, *J* = 7.5 Hz, 1H), 2.30-2.27 (m, 2H), 2.20 (s, 6H), 1.74 (dd, *J* = 12.8, 8.0 Hz, 1H), 1.45 (m, 1H), 1.26-1.22 (m, 1H), 0.92 (app q, *J* = 5.9 Hz, 1H); **^{13}C NMR** (151 MHz, $CDCl_3$) δ (ppm) 148.1, 141.2, 135.3, 128.8, 123.6, 121.1, 58.7, 45.2, 20.2, 17.3, 10.2; **HRMS-ESI** (m/z): found $[M+H]^+$ 221.1294, $C_{12}H_{17}N_2O_2$ requires 221.1290. **ee analysis:** HPLC Chiralpak AD-H (hexane(0.1% DEA):2-propanol 99.6:0.4, 1.0 mL·min^{-1}, 20°C), t_R = 27.5 min (major), t_R = 29.1 min (minor); $[\alpha]_D^{25.0}$ = –60° (c = 1.0, $CHCl_3$).

***N,N*-Dimethyl-1-((1*S*,2*R*)-2-(3-(trifluoromethyl)phenyl)cyclopropyl)methanamine (3ai)**

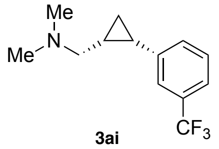

3ai

General procedure G was applied to 1-cyclopropyl-*N,N*-dimethylmethanamine (59.6 mg, 0.600 mmol) with (3-(trifluoromethyl)phenyl)boronic acid (57.0 mg, 0.300 mmol) in DMA (1.5 mL) at 40˚C for 24 hours to provide the title compound as a yellow oil (43.8 mg, 0.180 mmol, 60% yield, >99:1 e.r.). Enantiomeric excess was determined by HPLC analysis.

IR ν_{max}/cm^{-1} (thin film) 2970, 1325, 1162, 1162, 1074, 703; **^{1}H NMR** (600 MHz, $CDCl_3$) δ (ppm) 7.47-7.44 (m, 2H), 7.39-7.37 (m, 2H), 2.27-2.22 (m, 2H), 2.18 (S, 6H), 1.70 (dd, *J* = 12.8, 8.1 Hz, 1H), 1.37-1.34 (m, 1H), 1.20-1.17 (m, 1H), 0.85 (app q, *J* = 5.8 Hz, 1H); **^{13}C NMR** (101 MHz, $CDCl_3$) δ 140.2, 132.5, 130.6 (q, J_{C-F} = 34 Hz) 128.41, 125.91, 125.8 (q, J_{C-F} = 4.0 Hz), 124.4 (q, J_{C-F} = 272 Hz), 122.8 (q, J_{C-F} = 4.0 Hz), 59.0, 45.6, 20.3, 17.5; **HRMS-ESI** (m/z): found $[M+H]^+$ 244.1320, $C_{13}H_{17}NF_3$ requires 244.1313; **ee analysis:** HPLC Chiralpak AD-H (hexane(0.1% DEA):2-propanol 99.6:0.4, 1.0 mL·min^{-1}, 10°C), t_R = 7.5 min (major), t_R = 8.4 min (minor); $[\alpha]_D^{25.0}$ = –46° (c = 1.0, $CHCl_3$).

1-((1*R*,2*S*)-2-(3-Methoxyphenyl)cyclopropyl)-*N,N*-dimethylmethanamine (3aj)

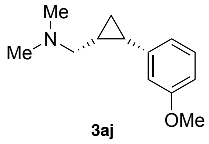

3aj

General procedure G was applied to 1-cyclopropyl-*N,N*-dimethylmethanamine (59.6 mg, 0.600 mmol) with (3-methoxyphenyl)boronic acid (45.6 mg, 0.300 mmol) in DMA (1.5 mL) at 40˚C for 24 hours to provide the title compound as a brown oil (38.7 mg, 0.189 mmol, 63% yield, 99:1 e.r.). Enantiomeric excess was determined by HPLC analysis.

IR ν_{max}/cm^{-1} (thin film) 2941, 2764, 1509, 1218, 1032, 832; **^{1}H NMR** (600 MHz, $CDCl_3$) δ (ppm) 7.19 (t, *J* = 7.8 Hz, 1H), 6.79 (d, *J* = 7.8 Hz, 1H), 6.76-6.73 (m, 2H), 3.81 (s, 3H), 2.32 (dd, *J* = 12.7, 4.9 Hz), 2.19-2.15 (m, 7H), 1.70 (dd, *J* = 12.7, 8.4 Hz, 1H), 1.30-1.24 (m, 1H), 1.12-1.10 (m, 1H), 0.80 (app q, *J* = 5.8 Hz, 1H); **^{13}C NMR** (151 MHz, $CDCl_3$) δ (ppm) 159.3, 140.6, 128.8, 121.5, 114.9, 111.1, 59.0, 55.1, 45.5, 20.3, 17.1, 9.8; **HRMS-ESI** (m/z): found $[M+H]^+$ 206.1550, $C_{13}H_{20}NO$ requires 206.1545. **ee analysis:** HPLC Chiralpak AD-H (hexane(0.1% DEA):2-propanol 98:2, 1.0 mL·min^{-1}, 30°C), t_R = 7.1 min (major), t_R = 7.6 min (minor); $[\alpha]_D^{25.0}$ = –67° (c = 1.0, $CHCl_3$).

***N,N*-Dimethyl-1-((1*R*,2*S*)-2-(naphthalen-2-yl)cyclopropyl)methanamine (3ak)**

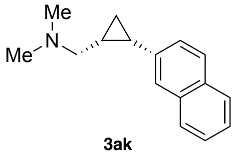

3ak

General procedure G was applied to 1-cyclopropyl-*N,N*-dimethylmethanamine (59.6 mg, 0.600 mmol) with naphthalen-2-ylboronic acid (51.6 mg, 0.300 mmol) in DMA (1.5 mL) at 40˚C for 24 hours to provide the title compound as a brown oil (50.1 mg, 0.222 mmol, 74% yield, 98:2 e.r.). Enantiomeric excess was determined by HPLC analysis.

IR ν_{max}/cm^{-1} (thin film) 2952, 2809, 1505, 1450, 1272, 1022; **^{1}H NMR** (400 MHz, $CDCl_3$) δ (ppm) 7.89 – 7.74 (m, 3H), 7.60 (d, *J* = 1.7 Hz, 1H), 7.49 – 7.37 (m, 3H), 2.40 – 2.30 (m, 2H), 2.15 (s, 6H), 1.73 (dd, *J* = 12.7, 8.3 Hz, 1H), 1.45 – 1.32 (m, 1H), 1.25 – 1.16 (m, 1H), 0.98 (q, *J* = 5.7 Hz, 1H); **^{13}C NMR** (101 MHz, $CDCl_3$) δ 136.50, 133.30, 132.05, 128.19, 127.56, 127.48, 127.41, 126.81, 125.91, 125.20, 59.07, 45.40, 20.56, 17.27, 9.77; **HRMS-ESI** (m/z): found $[M+H]^+$ 225.1513, $C_{16}H_{19}N$ requires 225.1517; **ee analysis:** HPLC Chiralpak AD-H (hexane(0.1% DEA):2-propanol 99:1, 1.0 mL·min^{-1}, 30°C), t_R = 15.1 min (major), t_R = 16.8 min (minor); $[\alpha]_D^{25.0}$ = –64° (c = 1.0, $CHCl_3$).

1-((1*R*,2*S*)-2-(Benzo[*d*][1,3]dioxol-5-yl)cyclopropyl)-*N,N*-dimethylmethanamine (3al)

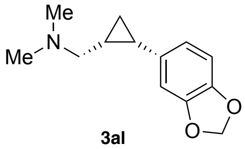

3al

General procedure G was applied to 1-cyclopropyl-*N,N*-dimethylmethanamine (59.6 mg, 0.600 mmol) with benzo[*d*][1,3]dioxol-5-ylboronic acid (49.8 mg, 0.300 mmol) in DMA (1.5 mL) at 40˚C for 24 hours to provide the title compound as a brown oil (42.2 mg, 0.192 mmol, 64% yield, 99:1 e.r.). Enantiomeric excess was determined by HPLC analysis.

IR ν_{max}/cm^{-1} (thin film) 2814, 2764, 1489, 1437, 1232, 1032, 934, 807; **^{1}H NMR** (600 MHz, $CDCl_3$) δ (ppm) 6.71 (d, *J* = 8.0 Hz, 1H), 6.69 (s, 1H), 6.65 (d, *J* = 8.0 Hz, 1H), 5.92 (s, 2H), 2.39 (dd, *J* = 12.7, 5.2 Hz, 1H), 2.24 (s, 6H), 2.15-2.11 (m, 1H), 1.72 (dd, *J* = 12.7, 8.6 Hz, 1H), 1.28-1.24 (m, 1H), 1.11-1.07 (m, 1H), 0.73 (app q, *J* = 5.5 Hz, 1H); **^{13}C NMR** (151 MHz, $CDCl_3$) δ (ppm) 147.3, 145.8, 132.4, 121.9, 109.5, 107.8, 100.8, 59.1, 45.1, 20.0, 16.3,

10.0; **HRMS-ESI** (m/z): found $[M+H]^+$ 220.1344, $C_{13}H_{18}NO_2$ requires 220.1338; **ee analysis:** HPLC Chiralpak AD-H (hexane(0.1% DEA):2-propanol 99.5:0.5, 1.0 mL·min^{-1}, 30°C), t_R = 18.8 min (major), t_R = 20.7 min (minor); $[\alpha]_D^{25.0}$ = –54° (c = 1.0, $CHCl_3$).

1-((1*R*,2*S*)-2-(6-Chloropyridin-3-yl)cyclopropyl)-*N,N*-dimethylmethanamine (3am)

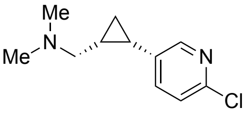

3am

General procedure G was applied to 1-cyclopropyl-*N,N*-dimethylmethanamine (59.6 mg, 0.600 mmol) with (6-chloropyridin-3-yl)boronic acid (47.2 mg, 0.300 mmol) in DMA (1.5 mL) at 50˚C for 24 hours to provide the title compound as a brown oil (22.0 mg, 0.105 mmol, 35% yield, 96:4 e.r.). Enantiomeric excess was determined by HPLC analysis.

IR ν_{max}/cm^{-1} (thin film) 3062, 2654, 1589, 1437, 1060, 863; **^{1}H NMR** (400 MHz, $CDCl_3$) δ (ppm) 8.28 (s, 1H), 7.49 (d, *J* = 8.2 Hz, 1H), 7.25 (d, *J* = 8.2 Hz, 1H), 2.23 (dd, *J* = 12.7, 5.5 Hz, 1H), 2.18 (s, 6H), 2.16 – 2.10 (m, 1H), 1.70 (dd, *J* = 12.7, 7.9 Hz, 1H), 1.43 – 1.32 (m, 1H), 1.22 (td, *J* = 8.2, 5.3 Hz, 1H), 0.82 (q, *J* = 5.8 Hz, 1H); **^{13}C NMR** (176 MHz, $CDCl_3$) δ (ppm) 150.4, 149.0, 139.3, 133.6, 123.4, 58.9, 45.3, 17.2, 17.0, 9.8; **HRMS-ESI** (m/z): found $[M+H]^+$ 210.0919, $C_{11}H_{15}ClN_2$ requires 210.0924; **ee analysis:** HPLC Chiralpak AD-H (hexane(0.1% DEA):2-propanol 95:5, 1.0 mL·min^{-1}, 30°C), t_R = 6.8 min (major), t_R = 10.8 min (minor); $[\alpha]_D^{25.0}$ = –45° (c = 1.0, $CHCl_3$).

***N,N*-Dimethyl-1-((1*R*,2*S*)-2-(6-(trifluoromethyl)pyridin-3-yl)cyclopropyl)methanamine (3an)**

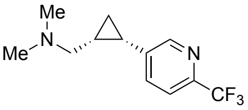

3an

General procedure G was applied to 1-cyclopropyl-*N,N*-dimethylmethanamine (59.6 mg, 0.600 mmol) with (6-(trifluoromethyl)pyridin-3-yl)boronic acid (47.2 mg, 0.300 mmol) in DMA (1.5 mL) at 50˚C for 24 hours to provide the title compound as a brown oil (33.0 mg, 0.135 mmol, 45% yield, >99:1 e.r.). Enantiomeric excess was determined by HPLC analysis.

IR ν_{max}/cm^{-1} (thin film) 3091, 2863, 1601, 1450, 1254, 905; **^{1}H NMR** (400 MHz, CDCl3) δ (ppm) 8.58 (s, 1H), 7.64 (dd, *J* = 8.0, 1.5 Hz, 1H), 7.56 (d, *J* = 8.0 Hz, 1H), 2.18 (d, *J* = 5.8 Hz, 1H), 2.14 (s, 4H), 1.69 (dd, *J* = 12.6, 7.9 Hz, 1H), 1.41 (qt, *J* = 8.4, 5.8 Hz, 1H), 1.25 (td, *J* = 8.3, 5.4 Hz, 1H), 0.88 (q, *J* = 5.9 Hz, 1H); **^{13}C NMR** (101 MHz, $CDCl_3$) δ (ppm) 151.0, 145.8 (q, J_{C-F} = 34 Hz), 138.6, 137.3, 121.9 (q, J_{C-F} = 275 Hz) , 119.7, 58.9, 45.6, 18.0, 17.9, 10.2; **HRMS-ESI** (m/z): found $[M+H]^+$ 244.1190, $C_{12}H_{15}F_3N_2$ requires 244.1187; **ee analysis:** HPLC Chiralpak AD-H (hexane(0.1% DEA):2-propanol 95:5, 1.0 mL·min^{-1}, 30 °C), t_R = 5.3 min (major), t_R = 6.2 min (minor); $[\alpha]_D^{25.0}$ = –36° (c = 1.0, $CHCl_3$).

1-((1*R*,2*S*)-2-(2-Fluoropyridin-4-yl)cyclopropyl)-*N,N*-dimethylmethanamine (3ao)

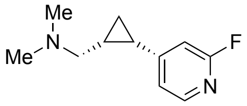

3ao

General procedure G was applied to 1-cyclopropyl-*N,N*-dimethylmethanamine (59.6 mg, 0.600 mmol) with (2-fluoropyridin-4-yl)boronic acid (42.3 mg, 0.300 mmol) in DMA (1.5 mL) at 50˚C for 24 hours to provide the title compound as a brown oil (18.7 mg, 0.096 mmol, 32% yield, >99:1 e.r.). Enantiomeric excess was determined by HPLC analysis.

IR ν_{max}/cm^{-1} (thin film) 3056, 2711, 1582, 1412, 1313, 751; **^{1}H NMR** (400 MHz, $CDCl_3$) δ (ppm) 8.09 (d, *J* = 5.2 Hz, 1H), 7.00 (d, *J* = 5.2 Hz, 1H), 6.72 (s, 1H), 2.26 – 2.19 (m, 2H), 2.17 (s, 7H), 1.83 (dd, *J* = 12.8, 7.6 Hz, 1H), 1.50 – 1.39 (m, 1H), 1.24 (td, *J* = 8.2, 5.5 Hz, 1H), 0.91 (q, *J* = 5.9 Hz, 1H); **^{13}C NMR** (176 MHz, $CDCl_3$) δ (ppm) 150.4, 149.0, 139.3, 133.6, 123.4, 58.9, 45.3, 17.2, 17.0, 9.8; **HRMS-ESI** (m/z): found $[M+H]^+$ 194.1218, $C_{11}H_{15}FN_2$ requires 194.1219; **ee analysis:** HPLC Chiralpak AD-H (hexane(0.1% DEA):2-propanol 95:5, 1.0 mL·min^{-1}, 30°C), t_R = 7.6 min (major) t_R = 8.0 min (minor); $[\alpha]_D^{25.0}$ = -67° (c = 1.0, $CHCl_3$).

***trans*-*N*,*N*-Dimethyl-1-(2-phenylcyclopropyl)methanamine (5)**

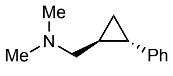

5

Subjecting the title compound (105 mg, 0.600 mmol) to General procedure G with (4-methoxyphenyl)boronic acid (45.6 mg, 0.300 mmol) in DMA at 50 °C provided by column chromatography (DCM to 2% $NH_{3(MeOH;\ 2M)}$) the unreacted starting material as a yellow oil (43.0 mg, 0.245 mmol, 82% yield, 75:25 e.r.). Enantiomeric excess was determined by HPLC analysis.

IR ν_{max}/cm^{-1} (thin film) 2940, 2761, 1604, 1497, 1455, 1033, 753, 695; **^{1}H NMR** (700 MHz, $CDCl_3$) δ (ppm) 7.27 (app t, *J* = 7.7 Hz, 2H), 7.16 (t, *J* = 7.0 Hz, 1H), 7.09 (d, *J* = 7.7 Hz, 2H), 2.43 (dd, *J* = 12.5, 6.3 Hz, 1H), 2.34 – 2.29 (m, 7H), 1.74 – 1.68 (m, 1H), 1.28 – 1.22 (m, 1H), 0.99 (dt, *J* = 8.7, 5.1 Hz, 1H), 0.86 (dt, *J* = 8.9, 5.3 Hz, 1H); **^{13}C NMR** (176 MHz, $CDCl_3$) δ (ppm) 143.0, 128.3, 125.7, 125.4, 64.1, 45.5, 22.6, 21.6, 15.0; **HMRS-ESI** (m/z): found $[M+H]^+$ 176.1438, $C_{12}H_{18}N$ requires 176.1439; **ee analysis:** HPLC Chiralpak AD-H (hexane(0.1% DEA):2-propanol 98:2, 1.0 mL·min^{-1}, 30 °C) t_R = 5.4 min (major), t_R = 5.8 min (minor); $[\alpha]_D^{25.0}$ = –49° (c = 0.4, $CHCl_3$).

1-((1*R*,2*S*,3*S*)-2-(4-Methoxyphenyl)-3-phenylcyclopropyl)-*N*,*N*-dimethylmethanamine (6)

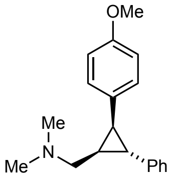

6

General procedure G was applied to *trans*-*N*,*N*-dimethyl-1-(2-phenylcyclopropyl)-methanamine (105 mg, 0.600 mmol) and (4-methoxyphenyl)boronic acid (45.6 mg, 0.300 mmol) in DMA at 50 °C to provide by column chromatography (DCM to 2% $NH_{3(MeOH;\ 2M)}$) the title compound as a yellow oil (51.4 mg, 0.183 mmol, 61% yield, 98:2 e.r.). Enantiomeric excess was determined by HPLC analysis.

IR ν_{max}/cm^{-1} (thin film) 2937, 2764, 1609, 1512, 1454, 1243, 1031, 830, 755, 695; **^{1}H NMR** (700 MHz, $CDCl_3$) δ (ppm) 7.32 (t, *J* = 7.5 Hz, 2H), 7.26 – 7.18 (m, 5H), 6.88 (d, *J* = 8.4 Hz, 2H), 3.83 (s, 3H), 2.54 (dd, *J* = 12.6, 4.6 Hz, 1H), 2.49 (dd, *J* = 8.6, 6.2 Hz, 1H), 2.25 (t, *J* = 5.2 Hz, 1H), 2.22 (s, 6H), 1.88 (dd, *J* = 12.6, 8.4 Hz, 1H), 1.66 – 1.60 (m, 1H); **^{13}C NMR** (176 MHz, $CDCl_3$) δ (ppm) 158.1, 142.3, 130.1, 129.9, 128.4, 126.1, 125.7, 113.6, 58.9, 55.3, 45.5, 30.6, 28.6, 27.7; **HMRS-ESI** (m/z): found $[M+H]^+$ 282.1852, $C_{19}H_{24}NO$ requires 282.1858; **ee analysis:** HPLC Chiralpak AD-H (hexane(0.1% DEA):2-propanol 98:2, 1.0 mL·min^{-1}, 30 °C) t_R = 11.6 min (minor), t_R = 14.0 min (major); $[\alpha]_D^{25.0}$ = –130° (c = 1.0, $CHCl_3$).

1-((1*S*,2*R*)-2-(4-Methoxyphenyl)-2-phenylcyclopropyl)-*N*,*N*-dimethylmethanamine (7)

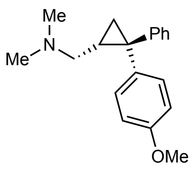

7

General procedure G was applied to *trans*-*N*,*N*-dimethyl-1-(2-phenylcyclo-propyl)-methanamine (105 mg, 0.600 mmol) and (4-methoxyphenyl)boronic acid (45.6 mg, 0.300 mmol) in DMA at 50 °C to provide by column chromatography (DCM to 2% $NH_{3(MeOH;\ 2M)}$) the title compound as a yellow oil (5.2 mg, 0.019 mmol, 6% yield, >99:1 e.r.). Enantiomeric excess was determined by HPLC analysis.

^{1}H NMR (700 MHz, $CDCl_3$) δ (ppm) 7.30 – 7.26 (m, 2H), 7.24 (t, *J* = 7.4 Hz, 2H), 7.20 (d, *J* = 7.6 Hz, 2H), 7.14 (t, *J* = 7.0 Hz, 1H), 6.86 (d, *J* = 8.5 Hz, 2H), 3.82 (s, 3H), 2.60 (dd, *J* = 12.4, 3.4 Hz, 1H), 2.26 (s, 6H), 1.82 – 1.76 (m, 1H), 1.55 (dd, *J* = 12.4, 9.2 Hz, 1H), 1.37 (dd, *J* = 8.0, 4.7 Hz, 1H), 1.33 – 1.29 (m, 1H); **^{13}C NMR** (176 MHz, $CDCl_3$) δ (ppm) 158.1, 147.3, 133.5, 131.4, 128.2, 127.5, 125.6, 113.6, 61.3, 55.2, 45.7, 33.4, 24.3, 21.3; **HMRS-ESI** (m/z): found $[M+H]^+$ 282.1854, $C_{19}H_{24}NO$ requires 282.1858; **ee analysis:** HPLC Chiralpak AD-H (hexane(0.1% DEA):2-propanol 98:2, 1.0 mL·min^{-1}, 30 °C) t_R = 8.3 min (minor), t_R = 12.2 min (major).

General procedure H: Methylene cyclobutane C–H arylation

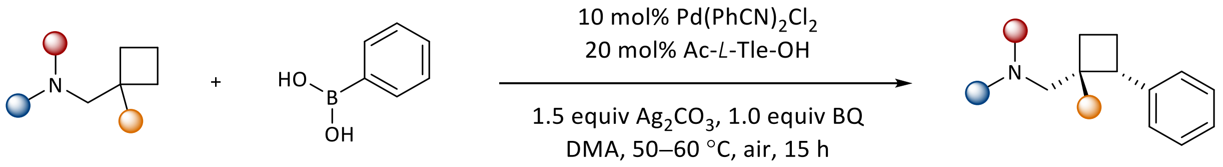

The desired tertiary amine (0.600 mmol) was added to a 2–5 mL microwave vial containing $Pd(PhCN)_2Cl_2$ (11.5 mg, 0.030 mmol), Ac-*L*-Tle-OH (10.4 mg, 0.060 mmol), Ag_2CO_3 (124 mg, 0.450 mmol) and 1,4-benzoquinone (BQ) (32.5 mg, 0.300 mmol) in anhydrous DMA (0.75 mL). The reaction was sealed and stirred at the required temperature for 5 minutes. The desired aryl boronic acid in DMA (0.75 mL) was added dropwise and the reaction mixture was stirred at 1000 rpm for 15 h.

The reaction was cooled to rt and Et_2O (5 mL) added, forcing the formation of a precipitate. The dark mixture was filtered through a pad of Celite and washed with Et_2O (25 mL). The resulting organic layer was washed with aq NaOH (0.25 M, 3 x 50 mL), dried over $MgSO_4$, filtered and concentrated *in vacuo*. Purification of the crude oil by column chromatography afforded the pure arylated product. Asymmetry was determined by HPLC analysis using a CHIRALPAK® AD-H column, GC-FID analysis using a Astec Chiraldex™ B-DM column, or ^{1}H-NMR analysis of the methylated tertiary amine product following a literature procedure reported by Lacour (*Org. Lett.* **2002**, *4*, 8, 1351–1354).

***N,N-D*imethyl-1-((1*R*,2*S*)-2-phenylcyclobutyl)methanamine (9a)**

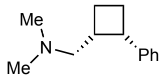

9a

General procedure H was applied to 1-cyclobutyl-*N,N*-dimethylmethanamine (67.9 mg, 0.600 mmol) with phenyl boronic acid (36.6 mg, 0.300 mmol) at 60 °C to provide by column chromatography (DCM to 4% $NH_{3(MeOH;\ 2M)}$) the title compound as a brown oil (41.3 mg, 0.218 mmol, 73% yield, >97:3 e.r.). Enantiomeric excess was determined by ^{1}H-NMR from the corresponding methylated ammonium derivative.

IR ν_{max}/cm^{-1} (thin film) 2941, 2763, 1494, 1455, 1031, 764, 737, 697; **^{1}H NMR** (600 MHz, $CDCl_3$) δ (ppm) 7.34 – 7.30 (m, 2H), 7.23 – 7.19 (m, 3H), 3.77 (q, *J* = 8.3 Hz, 1H), 2.90 – 2.81 (m, 1H), 2.41 – 2.29 (m, 2H), 2.24 (tt, *J* = 7.9, 4.9 Hz, 1H), 2.13 – 2.06 (m, 7H), 1.92 – 1.84 (m, 2H); **^{13}C NMR** (151 MHz, $CDCl_3$) δ (ppm) 141.6, 128.0, 127.8, 125.8, 61.2, 45.9, 41.8, 37.5, 24.2, 23.5; **HMRS-ESI** (m/z): found $[M+H]^+$ 190.1584, $C_{13}H_{20}N$ requires 190.1590; **ee analysis:** ^{1}H-NMR from the corresponding methylated ammonium derivative; $[\alpha]_D^{25.0}$ = –69° (c = 1.0, $CHCl_3$).

Ethyl 3-(methyl(((1*R*,2*S*)-2-phenylcyclobutyl)methyl)amino)propanoate (9b)

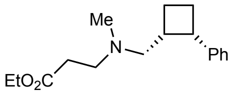

9b

General procedure H was applied to ethyl 3-((cyclobutylmethyl) (methyl)amino)-propanoate (120 mg, 0.600 mmol) with phenyl boronic acid (36.6 mg, 0.300 mmol) at 50

°C to provide by column chromatography (DCM to 3% $NH_{3(MeOH;\ 2M)}$) the title compound as a brown oil (58.5 mg, 0.212 mmol, 71% yield, 97:3 e.r.). Enantiomeric excess was determined by ^{1}H-NMR from the corresponding methylated ammonium derivative.

IR ν_{max}/cm^{-1} (thin film) 2973, 1731, 1458, 1177, 1030, 698; **^{1}H NMR** (700 MHz, $CDCl_3$) δ (ppm) 7.32 (t, *J* = 7.5 Hz, 2H), 7.24 – 7.18 (m, 3H), 4.12 (q, *J* = 7.1 Hz, 2H), 3.77 (q, *J* = 8.3 Hz, 1H), 2.91 – 2.83 (m, 1H), 2.63 – 2.57 (m, 1H), 2.55 – 2.50 (m, 1H), 2.40 – 2.29 (m, 4H), 2.24 – 2.17 (m, 1H), 2.14 (app t, *J* = 11.4 Hz, 1H), 2.10 (s, 3H), 1.99 (dd, *J* = 12.6, 4.7 Hz, 1H), 1.86 (tt, *J* = 11.3, 5.7 Hz, 1H), 1.26 (t, *J* = 7.1 Hz, 3H); **^{13}C NMR** (176 MHz, $CDCl_3$) δ (ppm) 172.7, 141.6, 128.0, 127.8, 125.8, 60.3, 58.7, 53.1, 42.3, 41.8, 37.2, 32.5, 24.0, 23.5, 14.2; **HMRS-ESI** (m/z): found $[M+H]^+$ 276.1964, $C_{17}H_{26}NO_2$ requires 276.1964; **ee analysis:** ^{1}H-NMR from the corresponding methylated ammonium derivative; $[\alpha]_D^{25.0}$ = –71° (c = 0.6, $CHCl_3$).

_N_-Methyl-3-(5-methylfuran-2-yl)-_N_-(((1_R_,2_S_)-2-phenylcyclobutyl)methyl)propan-1-amine (9c)

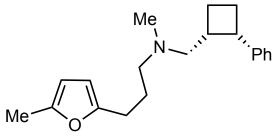

9c

General procedure H was applied to *N*-(cyclobutylmethyl)-*N*-methyl-3-(5-methylfuran-2-yl)propan-1-amine (133 mg, 0.600 mmol) with phenyl boronic acid (36.6 mg, 0.300 mmol) at 50 °C to provide by column chromatography (DCM to 4% $NH_{3(MeOH;\ 2M)}$) the title compound as a yellow oil (55.0 mg, 0.185 mmol, 62% yield, 97:3 e.r.). Enantiomeric excess was determined by HPLC analysis.

IR ν_{max}/cm^{-1} (thin film) 2942, 2788, 1453, 1218, 1018, 776, 697; **^{1}H NMR** (700 MHz, $CDCl_3$) δ (ppm) 7.31 (t, *J* = 7.4 Hz, 2H), 7.24 – 7.18 (m, 3H), 5.85 (s, 1H), 5.83 (s, 1H), 3.76 (q, *J* = 8.3 Hz, 1H), 2.92 – 2.83 (m, 1H), 2.58 – 2.48 (m, 2H), 2.40 – 2.29 (m, 2H), 2.27 (s, 3H), 2.26 – 2.16 (m, 3H), 2.13 (app t, *J* = 11.3 Hz, 1H), 2.09 (s, 3H), 1.98 (dd, *J* = 12.6, 4.6 Hz, 1H), 1.92 – 1.86 (m, 1H), 1.69 – 1.64 (m, 2H); **^{13}C NMR** (176 MHz, $CDCl_3$) δ (ppm) 154.4, 150.1, 141.8, 127.9, 127.8, 125.7, 105.7, 105.2, 59.1, 57.4, 42.6, 41.9, 37.4, 25.9, 25.8, 24.3, 23.6, 13.5; **HMRS-ESI** (m/z): found $[M+H]^+$ 298.2168, $C_{20}H_{28}NO$ requires 298.2171; **ee analysis:** HPLC Chiralpak AD-H (hexane(0.1% DEA):2-propanol 99.6:0.4, 1.0 mL·min^{-1}, 15 °C) t_R = 4.8 min (major), t_R = 5.1 min (minor); $[\alpha]_D^{25.0}$ = –64° (c = 1.0, $CHCl_3$).

3-((_tert_-Butyldimethylsilyl)oxy)-_N_-methyl-_N_-(((1_R_,2_S_)-2-phenylcyclobutyl)methyl)propan-1-amine (9d)

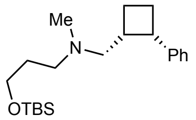

9d

General procedure H was applied to 3-((*tert*-butyldimethylsilyl)oxy)-*N*-(cyclobutylmethyl)-*N*-methylpropan-1-amine (163 mg, 0.600 mmol) with phenyl boronic acid (36.6 mg, 0.300 mmol) at 50 °C to provide by column chromatography (DCM to 3% $NH_{3(MeOH;\ 2M)}$) the title compound as a yellow oil (53.0 mg, 0.152 mmol, 51% yield, 97:3 e.r.). Enantiomeric excess was determined by ^{1}H-NMR from the corresponding methylated ammonium derivative.

IR ν_{max}/cm^{-1} (thin film) 2950, 2855, 1461, 1253, 1094, 832, 772, 697; **^{1}H NMR** (700 MHz, $CDCl_3$) δ (ppm) 7.32 (app t, *J* = 7.6 Hz, 2H), 7.24 – 7.18 (m, 3H), 3.77 (q, *J* = 8.3 Hz, 1H), 3.62 – 3.56 (m, 2H), 2.92 – 2.85 (m, 1H), 2.40

– 2.28 (m, 3H), 2.26 – 2.20 (m, 2H), 2.13 (dd, *J* = 12.4, 10.4 Hz, 1H), 2.09 (s, 3H), 1.98 (dd, *J* = 12.4, 4.7 Hz, 1H), 1.89 (tt, *J* = 11.2, 5.7 Hz, 1H), 1.57 (app p, *J* = 6.8 Hz, 2H), 0.91 (s, 9H), 0.06 (s, 6H); **^{13}C NMR** (176 MHz, $CDCl_3$) δ (ppm) 141.7, 128.0, 127.8, 125.7, 61.5, 59.1, 54.7, 42.7, 41.9, 37.4, 30.5, 26.0, 24.3, 23.6, 18.3, -5.3; **HMRS-ESI** (m/z): found $[M+H]^+$ 348.2718, $C_{21}H_{38}NOSi$ requires 348.2723; **ee analysis:** ^{1}H-NMR from the corresponding methylated ammonium derivative; $[\alpha]_D^{25.0}$ = –62° (c = 1.0, $CHCl_3$).

***tert*-Butyl methyl(2-(methyl(((1*R*,2*S*)-2-phenylcyclobutyl)methyl)amino)ethyl)carbamate (9e)**

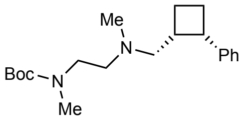

9e

General procedure H was applied to *tert*-butyl (2-((cyclobutyl-methyl)(methyl)amino)-ethyl)(methyl)carbamate (155 mg, 0.600 mmol) with phenyl boronic acid (36.6 mg, 0.300 mmol) at 50 °C to provide by column chromatography (DCM to 3% $NH_{3(MeOH;\,2M)}$) the title compound as a brown oil (63.3 mg, 0.190 mmol, 64% yield, >97:3 e.r.). Enantiomeric excess was determined by ^{1}H-NMR from the corresponding methylated ammonium derivative.

IR ν_{max}/cm^{-1} (thin film) 2971, 1691, 1390, 1153, 1044, 765, 698; **^{1}H NMR** (700 MHz, $CDCl_3$) δ (ppm) 7.31 (t, *J* = 7.6 Hz, 2H), 7.24 – 7.18 (m, 3H), 3.77 (q, *J* = 8.2 Hz, 1H), 3.28 – 3.04 (m, 2H), 2.89 – 2.77 (m, 4H), 2.40 – 2.26 (m, 4H), 2.23 – 2.17 (m, 1H), 2.17 – 2.09 (m, 4H), 2.07 – 2.00 (m, 1H), 1.87 (tt, *J* = 11.2, 5.8 Hz, 1H), 1.45 (s, 9H); **^{13}C NMR** (176 MHz, $CDCl_3$) δ (ppm) 155.7, 141.6, 127.9, 127.8, 125.8, 79.1, 59.4, 55.6, 47.0, 42.8, 41.8, 37.3, 34.6, 28.5, 24.0, 23.5; **HMRS-ESI** (m/z): found $[M+H]^+$ 333.2535, $C_{20}H_{33}N_2O_2$ requires 333.2542; **ee analysis:** ^{1}H-NMR from the corresponding methylated ammonium derivative; $[\alpha]_D^{25.0}$ = –63° (c = 1.0, $CHCl_3$).

1-(((1*R*,2*S*)-2-Phenylcyclobutyl)methyl)piperidine (9f)

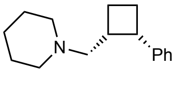

9f

General procedure H was applied to 1-(cyclobutylmethyl)piperidine (67.9 mg, 0.600 mmol) and phenyl boronic acid (36.6 mg, 0.300 mmol) with phenyl boronic acid (36.6 mg, 0.300 mmol) in NMP at 50 °C to provide by column chromatography (DCM to 4% $NH_{3(MeOH;\,2M)}$) the title compound as a brown oil (33.3 mg, 0.148 mmol, 50% yield, 94.5:5.5 e.r.). Enantiomeric excess was determined by ^{1}H-NMR from the corresponding methylated ammonium derivative.

IR ν_{max}/cm^{-1} (thin film) 2932, 2850, 2765, 1602, 1494, 1441, 1302, 1153, 1121, 1038, 995, 736, 696; **^{1}H NMR** (600 MHz, $CDCl_3$) δ (ppm) 7.31 (app t, *J* = 7.6 Hz, 2H), 7.24 – 7.17 (m, 3H), 3.75 (q, *J* = 8.2 Hz, 1H), 2.95 – 2.86 (m, 1H), 2.37 – 2.28 (m, 2H), 2.28 – 2.08 (m, 5H), 2.07 – 2.00 (m, 2H), 1.94 – 1.88 (m, 1H), 1.54 – 1.46 (m, 4H), 1.42 – 1.29 (m, 2H); **^{13}C NMR** (151 MHz, $CDCl_3$) δ (ppm) 141.8, 128.0, 127.9, 125.7, 60.8, 54.8, 42.3, 37.4, 26.0, 25.3, 24.4, 23.7; **HMRS-ESI** (m/z): found $[M+H]^+$ 230.1896, $C_{16}H_{24}N$ requires 230.1903; **ee analysis:** ^{1}H-NMR from the corresponding methylated ammonium derivative; $[\alpha]_D^{25.0}$ = –55° (c = 1.0, $CHCl_3$).

8-(((1*R*,2*S*)-2-Phenylcyclobutyl)methyl)-1,4-dioxa-8-azaspiro[4.5]decane (9g)

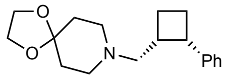

9g

General procedure H was applied to 8-(cyclobutylmethyl)-1,4-dioxa-8-azaspiro[4.5]-decane (127 mg, 0.600 mmol) with phenyl boronic acid (36.6 mg, 0.300 mmol) at 50 °C to provide by column chromatography (DCM to 4% $NH_{3(MeOH;\ 2M)}$) the title compound as a yellow oil (42.6 mg, 0.148 mmol, 50% yield, 95:5 e.r.). Enantiomeric excess was determined by HPLC analysis.

IR ν_{max}/cm^{-1} (thin film) 2948, 2808, 1308, 1142, 1090, 1038, 913, 765, 698; **^{1}H NMR** (700 MHz, $CDCl_3$) δ (ppm) 7.33 – 7.27 (m, 2H), 7.22 (d, *J* = 7.5 Hz, 2H), 7.20 (t, *J* = 7.3 Hz, 1H), 3.93 (s, 4H), 3.77 (q, *J* = 8.2 Hz, 1H), 2.90 (app hex, *J* = 7.2 Hz, 1H), 2.44 – 2.26 (m, 6H), 2.23 (app p, *J* = 8.1 Hz, 1H), 2.11 (d, *J* = 7.2 Hz, 2H), 1.92 – 1.86 (m, 1H), 1.66 (br s, 4H); **^{13}C NMR** (176 MHz, $CDCl_3$) δ (ppm) 141.7, 128.0, 127.9, 125.7, 107.3, 64.1, 59.4, 51.5, 42.2, 37.4, 34.8, 24.8, 23.6; **HMRS-ESI** (m/z): found $[M+H]^+$ 288.1962, $C_{18}H_{28}NO_2$ requires 288.1964; **ee analysis:** HPLC Chiralpak AD-H (hexane(0.1% DEA):2-propanol 97:3, 1.0 mL·min^{-1}, 30 °C) t_R = 5.7 min (major), t_R = 6.0 min (minor); $[\alpha]_D^{25.0}$ = –52° (c = 1.0, $CHCl_3$).

(2*S*,6*R*)-2,6-Dimethyl-4-(((1*R*,2*S*)-2-phenylcyclobutyl)methyl)morpholine (9h)

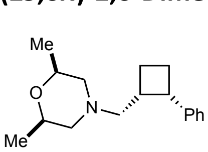

9h

General procedure H was applied to (2*S*,6*R*)-4-(cyclobutylmethyl)-2,6-dimethylmorpholine (110 mg, 0.600 mmol) with phenyl boronic acid (36.6 mg, 0.300 mmol) at 50 °C to provide by column chromatography (DCM to 3% $NH_{3(MeOH;\ 2M)}$) the title compound as a yellow oil (23.1 mg, 0.089 mmol, 30% yield, 91:9 e.r.). Enantiomeric excess was determined by GC-FID analysis.

IR ν_{max}/cm^{-1} (thin film) 2969, 2933, 2864, 1454, 1322, 1142, 1080, 766, 697; **^{1}H NMR** (700 MHz, $CDCl_3$) δ (ppm) 7.31 (t, *J* = 7.4 Hz, 2H), 7.25 – 7.18 (m, 3H), 3.78 (q, *J* = 8.2 Hz, 1H), 3.65 – 3.59 (m, 1H), 3.58 – 3.52 (m, 1H), 2.95 – 2.87 (m, 1H), 2.58 (d, *J* = 11.1 Hz, 1H), 2.43 (d, *J* = 11.1 Hz, 1H), 2.41 – 2.29 (m, 2H), 2.22 (app p, *J* = 8.8 Hz, 1H), 2.08 (app t, *J* = 10.9 Hz, 1H), 2.03 (dd, *J* = 12.5, 5.0 Hz, 1H), 1.92 – 1.86 (m, 1H), 1.63 (app t, *J* = 10.7 Hz, 1H), 1.51 (app t, *J* = 10.7 Hz, 1H), 1.12 (d, *J* = 6.2 Hz, 3H), 1.09 (d, *J* = 6.2 Hz, 3H); **^{13}C NMR** (176 MHz, $CDCl_3$) δ (ppm) 141.5, 128.0, 127.9, 125.8, 71.6, 71.6, 60.2, 59.8, 59.4, 42.0, 36.8, 24.5, 23.5, 19.2, 19.1; **HMRS-ESI** (m/z): found $[M+H]^+$ 260.2012, $C_{17}H_{26}NO$ requires 260.2014; **ee analysis:** GC-FID ChiralDex β-DM (110 °C; isocratic; linear velocity 40 cm·s^{-1}; split ratio 10.0) t_R = 25.6 min (major), t_R = 26.5 min (minor); $[\alpha]_D^{25.0}$ = –60° (c = 0.8, $CHCl_3$).

1-(4-Nitrophenyl)-4-(((1*R*,2*S*)-2-phenylcyclobutyl)methyl)piperazine (9l)

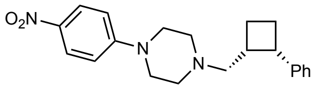

9i

General procedure H was applied to 1-(cyclobutylmethyl)-4-(4-nitrophenyl)-piperazine (165 mg, 0.600 mmol) with phenyl boronic acid (36.6 mg, 0.300 mmol) at 50 °C to provide by column chromatography (DCM to 3% $NH_{3(MeOH;\ 2M)}$) the title compound as a yellow oil (34.6 mg, 0.098 mmol, 33% yield, 94:6 e.r.). Enantiomeric excess was determined by HPLC analysis.

IR ν_{max}/cm^{-1} (thin film) 2970, 1956, 1492, 1320, 1239, 1112, 1001, 732, 699; **^{1}H NMR** (700 MHz, $CDCl_3$) δ (ppm) 8.17 – 8.08 (m, 2H), 7.36 – 7.29 (m, 2H), 7.27 – 7.19 (m, 3H), 6.81 – 6.76 (m, 2H), 3.81 (q, *J* = 8.3 Hz, 1H), 3.39 – 3.29 (m, 4H), 2.99 – 2.88 (m, 1H), 2.45 – 2.31 (m, 6H), 2.30 – 2.20 (m, 1H), 2.19 – 2.10 (m, 2H), 1.94 – 1.85 (m, 1H); **^{13}C NMR** (176 MHz, $CDCl_3$) δ (ppm) 154.9, 141.4, 138.2, 128.0, 126.0, 125.9, 112.5, 59.6, 52.7, 47.0, 42.0, 36.8, 24.2, 23.5; **HMRS-ESI** (m/z): found [M+H]$^+$ 352.2031, $C_{21}H_{26}N_3O_2$ requires 352.2025; **ee analysis:** HPLC Chiralpak AD-H (hexane(0.1% DEA):2-propanol 98:2, 1.0 mL·min^{-1}, 30 °C) t_R = 23.3 min (major), t_R = 26.5 min (minor); $[\alpha]_D^{25.0}$ = –5.9° (c = 0.8, $CHCl_3$).

1-((1*S*,2*R*)-1-(Methoxymethyl)-2-phenylcyclobutyl)-*N,N*-dimethylmethanamine (9j)

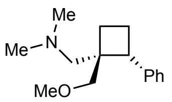

9j

General procedure H was applied to 1-(1-(methoxymethyl)cyclobutyl)-*N*,*N*-dimethyl-methanamine (94.3 mg, 0.600 mmol) with phenyl boronic acid (36.6 mg, 0.300 mmol) at 50 °C to provide by column chromatography (DCM to 2% $NH_{3(MeOH;\ 2M)}$) the title compound as a yellow oil (33.8 mg, 0.151 mmol, 51% yield, 88:12 e.r.). Enantiomeric excess was determined by GC-FID analysis.

IR ν_{max}/cm^{-1} (thin film) 2973, 2939, 2815, 2763, 1452, 1103, 1032, 697; **^{1}H NMR** (700 MHz, $CDCl_3$) δ (ppm) 7.31 (t, *J* = 7.6 Hz, 2H), 7.24 (d, *J* = 7.5 Hz, 2H), 7.20 (t, *J* = 7.3 Hz, 1H), 3.71 (t, *J* = 9.0 Hz, 1H), 3.55 – 3.45 (m, 5H), 2.32 (app p, *J* = 9.3 Hz, 1H), 2.21 – 2.16 (m, 1H), 2.13 (s, 6H), 2.10 – 2.03 (m, 2H), 2.03 – 1.97 (m, 1H), 1.91 (td, *J* = 10.7, 3.9 Hz, 1H); **^{13}C NMR** (176 MHz, $CDCl_3$) δ (ppm) 140.9, 128.1, 127.8, 125.7, 77.8, 61.1, 59.2, 47.8, 47.1, 44.7, 24.9, 20.2; **HMRS-ESI** (m/z): found [M+H]$^+$ 234.1857, $C_{15}H_{24}NO$ requires 234.1858; **ee analysis:** GC-FID ChiralDex β-DM (50 °C to 72 °C; 0.15 °C/min; linear velocity 40 cm·s^{-1}; split ratio 10.0) t_R = 131.3 min (minor), t_R = 134.3 min (major); $[\alpha]_D^{25.0}$ = –20° (c = 1.0, $CHCl_3$).

1-((1*R*,2*S*)-2-(4-Chlorophenyl)cyclobutyl)-*N,N*-dimethylmethanamine (9aa)

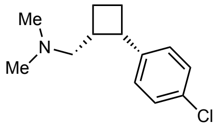

9aa

General procedure H was applied to 1-cyclobutyl-*N,N*-dimethylmethanamine (67.9 mg, 0.600 mmol) with (4-chlorophenyl)boronic acid (46.9 mg, 0.300 mmol) and *N*-acetyl-*L*-phenylalanine (12.4 mg, 0.060 mmol) at 50˚C to provide the title compound as a yellow oil (37.7 mg, 0.168 mmol, 56% yield, 99:1 e.r.). Enantiomeric excess was determined by HPLC analysis.

IR ν_{max}/cm^{-1} 2937, 2732, 1562, 1401, 1096, 1031, 727; **^{1}H NMR** (700 MHz, CDCl3) δ (ppm) 7.28 (d, *J* = 8.3 Hz, 2H), 7.15 (d, *J* = 8.3 Hz, 2H), 3.73 (q, *J* = 8.3 Hz, 1H), 2.88-2.82 (m, 1H), 2.35-2.29 (m, 2H), 2.27 – 2.18 (m, 1H), 2.10 (s, 6H), 2.06 – 2.02 (m, 1H), 1.91 – 1.82 (m, 2H); **^{13}C NMR** (176 MHz, $CDCl_3$) δ 140.3, 131.6, 129.3, 128.2, 61.3, 46.0, 41.4, 37.5, 24.2, 23.7; **HRMS-ESI** (m/z): found [M+H]$^+$ 223.1133, $C_{13}H_{18}ClN$ requires 223.1128; **ee analysis:** HPLC Chiralpak AD-H (hexane(0.1% DEA):2-propanol 99:1, 1.0 mL·min^{-1}, 30°C), t_R = 15.0 min (minor), t_R = 8.0 min (major); $[\alpha]_D^{25.0}$ = –79° (c = 1.0, $CHCl_3$).

N,N-Dimethyl-1-((1R,2S)-2-(4-nitrophenyl)cyclobutyl)methanamine (9ab)

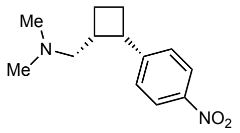

9ab

General procedure H was applied to 1-cyclobutyl-*N,N*-dimethylmethanamine (67.9 mg, 0.600 mmol) with (4-nitrophenyl)boronic acid (50.1 mg, 0.300 mmol) and *N*-acetyl-*L*-phenylalanine (12.4 mg, 0.060 mmol) at 50˚C to provide the title compound as a brown oil (32.1 mg, 0.138 mmol, 46% yield, 99:1 e.r.). Enantiomeric excess was determined by HPLC analysis.

IR ν_{max}/cm^{-1} (thin film) 2941, 2745, 1515, 1453, 1236, 1032, 842; **^{1}H NMR** (700 MHz, $CDCl_3$) δ (ppm) 8.17 (d, *J* = 8.7 Hz, 2H), 7.37 (d, *J* = 8.6 Hz, 2H), 3.86 (q, *J* = 8.2 Hz, 1H), 2.99 – 2.90 (m, 1H), 2.39 (q, *J* = 7.8 Hz, 2H), 2.30 – 2.24 (m, 1H), 2.08 (s, 6H), 2.04 – 1.99 (m, 2H), 1.93 – 1.88 (m, 1H), 1.85 (dd, *J* = 12.4, 5.2 Hz, 1H); **^{13}C NMR** (101 MHz, $CDCl_3$) δ (ppm) 149.7, 146.3, 128.6, 123.3, 60.9, 45.7, 41.8, 37.5, 24.0, 23.4; **HRMS-ESI** (m/z): found $[M+H]^+$ 234.1366, $C_{13}H_{18}N_2O_2$ requires 234.1368; **ee analysis:** HPLC Chiralpak AD-H (hexane(0.1% DEA):2-propanol 99.6:0.4, 1.0 mL·min^{-1}, 30°C), t_R = 23.0 min (major) t_R = 8.0 min (minor); $[\alpha]_D^{25.0}$ = –54° (c = 1.0, $CHCl_3$).

1-((1R,2S)-2-(4-Methoxyphenyl)cyclobutyl)-N,N-dimethylmethanamine (9ac)

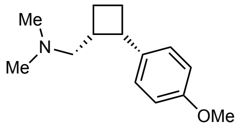

9ac

General procedure H was applied to 1-cyclobutyl-*N,N*-dimethylmethanamine (67.9 mg, 0.600 mmol) with (4-methoxyphenyl)boronic acid (45.6 mg, 0.300 mmol) and *N*-acetyl-*L*-phenylalanine (12.4 mg, 0.060 mmol) at 50˚C to provide the title compound as a brown oil (32.2 mg, 0.147 mmol, 49% yield, 98:2 e.r.). Enantiomeric excess was determined by HPLC analysis.

IR ν_{max}/cm^{-1} (thin film) 2952, 2729, 1498, 1432, 1143, 952; **^{1}H NMR** (700 MHz, $CDCl_3$) δ (ppm) 7.11 (d, *J* = 8.7 Hz, 2H), 6.85 (d, *J* = 8.7 Hz, 2H), 3.80 (s, 3H), 3.70 (q, *J* = 8.2 Hz, 1H), 2.86 – 2.77 (m, 1H), 2.29 (q, *J* = 7.7 Hz, 2H), 2.24 – 2.18 (m, 1H), 2.10 (s, 6H), 2.09 – 2.05 (m, 1H), 1.91 – 1.83 (m, 2H); **^{13}C NMR** (176 MHz, $CDCl_3$) δ (ppm) 157.9, 133.8, 128.9, 113.6, 61.4, 55.4, 46.0, 41.4, 37.6, 24.5, 24.0; **HRMS-ESI** (m/z): found $[M+H]^+$ 219.1619, $C_{14}H_{21}NO$ requires 219.1623; **ee analysis:** HPLC Chiralpak AD-H (hexane(0.1% DEA):2-propanol 99:1, 1.0 mL·min^{-1}, 30 °C), t_R = 13.4 min (minor), t_R = 14.1 min (major); $[\alpha]_D^{25.0}$ = –62° (c = 1.0, $CHCl_3$).

Ethyl 4-((1S,2R)-2-((dimethylamino)methyl)cyclobutyl)benzoate (9ad)

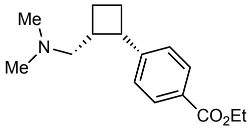

9ad

General procedure H was applied to 1-cyclobutyl-*N,N*-dimethylmethanamine (67.9 mg, 0.600 mmol) with (4-(ethoxycarbonyl)phenyl)boronic acid (58.2 mg, 0.300 mmol) and *N*-acetyl-*L*-phenylalanine (12.4 mg, 0.060 mmol) at 50˚C to provide the title compound as a yellow oil (42.3 mg, 0.162 mmol, 54% yield, 98.5:1.5 e.r.). Enantiomeric excess was determined by HPLC analysis.

IR ν_{max}/cm^{-1} (thin film) 2981, 2746, 1705, 1234, 1089, 1001, 757; **^{1}H NMR** (700 MHz, CDCl3) δ (ppm) 7.98 (d, *J* = 8.2 Hz, 2H), 7.26 (d, *J* = 8.2 Hz, 2H), 4.37 (q, *J* = 7.1 Hz, 2H), 3.80 (q, *J* = 8.2 Hz, 1H), 2.93 – 2.82 (m, 1H), 2.40 – 2.30 (m, 2H), 2.24 (dq, *J* = 11.4, 7.8 Hz, 1H), 2.08 (s, 6H), 2.03 (dd, *J* = 12.4, 10.4 Hz, 1H), 1.90 (ddd, *J* = 15.2, 11.5, 5.9 Hz, 1H), 1.84 (dd, *J* = 12.4, 4.6 Hz, 1H), 1.39 (t, *J* = 7.1 Hz, 3H); **^{13}C NMR** (176 MHz, $CDCl_3$) δ (ppm)

166.9, 147.3, 129.5, 128.2, 127.9, 61.2, 61.0, 45.9, 42.0, 37.6, 24.4, 23.5, 14.5; **HRMS-ESI** (m/z): found $[M+H]^+$ 261.1731, $C_{16}H_{23}NO_2$ requires 261.1729; **ee analysis:** HPLC Chiralpak AD-H (hexane(0.1% DEA):2-propanol 99:1, 1.0 mL·min^{-1}, 30 °C), t_R = 18.9 min (minor), t_R = 20.0 min (major); $[\alpha]_D^{25.0}$ = –75° (c = 1.0, $CHCl_3$).

_N,N_-Dimethyl-1-((1_R_,2_S_)-2-(4-(trifluoromethoxy)phenyl)cyclobutyl)methanamine (9ae)

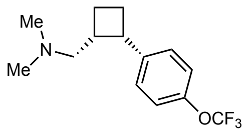

9ae

General procedure H was applied to 1-cyclobutyl-*N,N*-dimethylmethanamine (67.9 mg, 0.600 mmol) with ((4-trifluoromethoxy)phenyl)boronic acid (61.8 mg, 0.300 mmol) and *N*-acetyl-*L*-phenylalanine (12.4 mg, 0.060 mmol) at 50˚C to provide the title compound as a brown oil (41.0 mg, 0.150 mmol, 50% yield, 99:1 e.r.). Enantiomeric excess was determined by HPLC analysis.

IR ν_{max}/cm^{-1} (thin film) 2952, 2729, 1498, 1432, 1143, 952; **^{1}H NMR** (700 MHz, $CDCl_3$) δ (ppm) 7.20 (d, *J* = 8.6 Hz, 2H), 7.14 (d, *J* = 8.6 Hz, 2H), 3.74 (q, *J* = 8.3 Hz, 1H), 2.87-2.81 (m, 1H), 2.35-2.28 (m, 2H), 2.25 – 2.17 (m, 1H), 2.08 (s, 6H), 2.02 (dd, *J* = 12.3, 10.3 Hz, 1H), 1.89 – 1.81 (m, 2H); **^{13}C NMR** (176 MHz, $CDCl_3$) δ (ppm) 147.5, 140.5, 129.2, 120.6, 61.2, 46.0, 41.4, 37.5, 24.2, 23.8; **HRMS-ESI** (m/z): found $[M+H]^+$ 273.1334, $C_{14}H_{18}F_3NO$ requires 273.1340; **ee analysis:** HPLC Chiralpak AD-H (hexane(0.1% DEA):2-propanol 98:2, 1.0 mL·min^{-1}, 20°C), t_R = 10.8 min (minor) t_R = 12.0 min (major); $[\alpha]_D^{25.0}$ = –58° (c = 1.0, $CHCl_3$).

1-((1_R_,2_S_)-2-(3-Bromophenyl)cyclobutyl)-_N,N_-dimethylmethanamine (9af)

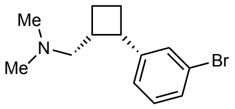

9af

General procedure H was applied to 1-cyclobutyl-*N,N*-dimethylmethanamine (67.9 mg, 0.600 mmol) with (3-bromophenyl)boronic acid (60.2 mg, 0.300 mmol) and *N*-acetyl-*L*-phenylalanine (12.4 mg, 0.060 mmol) at 50˚C to provide the title compound as a brown oil (39.5 mg, 0.147 mmol, 49% yield, >97:3 e.r.). Enantiomeric excess was determined by ^{1}H-NMR from the corresponding methylated ammonium derivative.

IR ν_{max}/cm^{-1} (thin film) 2960, 2737, 1511, 1066, 982, 851; **^{1}H NMR** (700 MHz, $CDCl_3$) δ (ppm) 7.34 (s, 1H), 7.32 (d, *J* = 7.7 Hz, 1H), 7.16 (t, *J* = 7.7 Hz, 1H), 7.12 (d, *J* = 7.7 Hz, 1H), 3.72 (q, *J* = 8.3 Hz, 1H), 2.90 – 2.77 (m, 1H), 2.36 – 2.27 (m, 2H), 2.26 – 2.16 (m, 1H), 2.09 (s, 6H), 2.04 (dd, *J* = 12.6, 10.5 Hz, 1H), 1.86 (dt, *J* = 12.6, 6.5 Hz, 2H); **^{13}C NMR** (176 MHz, $CDCl_3$) δ (ppm) 144.3, 130.9, 129.7, 129.0, 126.7, 122.5, 61.1, 46.0, 41.6, 37.6, 24.1, 23.6; **HRMS-ESI** (m/z): found $[M+H]^+$ 267.0619, $C_{13}H_{18}BrN$ requires 267.0623; **ee analysis:** ^{1}H-NMR from the corresponding methylated ammonium derivative; $[\alpha]_D^{25.0}$ = –55° (c = 1.0, $CHCl_3$).

1-((1_R_,2_S_)-2-(4-(Benzyloxy)-3-fluorophenyl)cyclobutyl)-_N,N_-dimethylmethanamine (9ag)

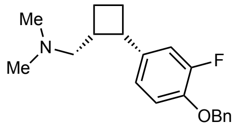

9ag

General procedure H was applied to 1-cyclobutyl-*N,N*-dimethylmethanamine (67.9 mg, 0.600 mmol) with (4-(benzyloxy)-3-fluorophenyl)-boronic acid (73.8 mg, 0.300 mmol) and *N*-acetyl-*L*-phenylalanine (12.4 mg, 0.060 mmol) at 50˚C to provide the title

compound as a brown oil (42.3 mg, 0.135 mmol, 45% yield, 99:1 e.r.). Enantiomeric excess was determined by HPLC analysis.

IR ν_{max}/cm^{-1} (thin film) 2952, 2780, 1450, 1364, 1123, 960; **^{1}H NMR** (700 MHz, $CDCl_3$) δ (ppm) 7.47 – 7.42 (m, 2H), 7.38 (dd, *J* = 8.4, 6.8 Hz, 2H), 7.35 – 7.30 (m, 1H), 6.99 – 6.90 (m, 2H), 6.84 (ddd, *J* = 8.4, 2.1, 1.0 Hz, 1H), 5.13 (s, 2H), 3.67 (q, *J* = 8.2 Hz, 1H), 2.86 – 2.77 (m, 1H), 2.33 – 2.16 (m, 3H), 2.11 (s, 6H), 2.06 (dd, *J* = 12.5, 10.4 Hz, 1H), 1.91 – 1.81 (m, 2H); **^{13}C NMR** (176 MHz, $CDCl_3$) δ (ppm) 153.5, 152.1, 144.8, 136.9, 128.7, 128.2, 127.6, 123.4, 115.8, 71.7, 61.1, 45.9, 41.2, 37.4, 24.3, 23.9; **HRMS-ESI** (m/z): found $[M+H]^+$ 313.1844, $C_{20}H_{24}FNO$ requires 313.1842; **ee analysis:** HPLC Chiralpak AD-H (hexane(0.1% DEA):2-propanol 99.5:0.5, 1.0 mL·min^{-1}, 40°C), t_R = 17.9 min (minor), t_R = 20.0 min (major); $[\alpha]_D^{25.0}$ = –42° (c = 1.0, $CHCl_3$).

3-(3-((((7*S*,8*S*)-3,4-Dimethoxy-8-phenylbicyclo[4.2.0]octa-1(6),2,4-trien-7-yl)methyl)(methyl)amino)-propyl)-7,8-dimethoxy-1,3,4,5-tetrahydro-2H-benzo[d]azepin-2-one (11)

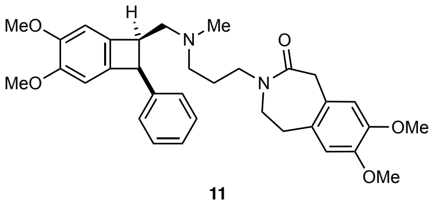

11

General procedure H was applied to Ivabradine (70.3 mg, 0.150 mmol) with *D*-Ac-Tle-OH (3.5 mg, 0.020 mmol) and phenyl boronic acid (36.6 mg, 0.300 mmol) at 50 °C to provide by column chromatography (DCM to 3% $NH_{3(MeOH;\ 2M)}$) the title compound as a yellow oil (10.8 mg, 0.020 mmol, 20% yield).

^{1}H NMR (700 MHz, $CDCl_3$) δ (ppm) 7.30 (t, *J* = 7.5 Hz, 2H, 7.24 (t, *J* = 7.2 Hz, 1H), 7.17 (d, *J* = 7.5 Hz, 2H), 6.84 (s, 1H), 6.79 (s, 1H), 6.60 (s, 1H), 6.55 (s, 1H), 4.79 (d, *J* = 5.3 Hz, 1H), 3.92 (s, 3H), 3.89 (s, 3H), 3.88 – 3.86 (m, 1H), 3.85 (s, 3H), 3.84 (s, 3H), 3.83 – 3.77 (m, 2H), 3.70 – 3.62 (m, 2H), 3.50 – 3.41 (m, 1H), 3.37 – 3.28 (m, 1H), 3.06 – 2.97 (m, 2H), 2.21 (t, *J* = 7.1 Hz, 2H), 2.17 (s, 3H), 2.12 (d, *J* = 7.4 Hz, 2H), 1.64 – 1.54 (m, 2H); **^{13}C NMR** (176 MHz, $CDCl_3$) δ (ppm) 172.0, 150.1, 150.0, 147.8, 147.1, 140.0, 138.9, 136.3, 128.8, 128.0, 127.4, 126.6, 123.5, 114.0, 113.1, 107.5, 107.1, 58.8, 56.4, 56.3, 55.9, 55.9, 55.1, 50.3, 46.5, 45.9, 44.9, 42.7, 42.3, 32.4, 26.2; **HMRS-ESI** (m/z): found $[M+H]^+$ 545.3014, $C_{33}H_{41}N_2O_5$ requires 545.3015; $[\alpha]_D^{25.0}$ = –93° (c = 0.5, $CHCl_3$).

3-(3-((((7*S*,8*R*)-8-([1,1'-Biphenyl]-2-yl)-3,4-dimethoxybicyclo[4.2.0]octa-1(6),2,4-trien-7-yl)methyl)-(methyl)amino)propyl)-7,8-dimethoxy-1,3,4,5-tetrahydro-2H-benzo[d]azepin-2-one (12)

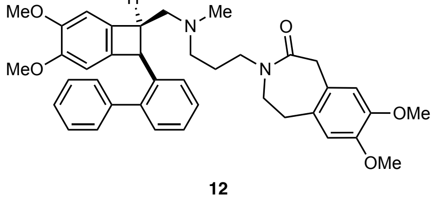

12

General procedure H was applied to Ivabradine (70.3 mg, 0.150 mmol) with *D*-Ac-Tle-OH (3.5 mg, 0.020 mmol) and phenyl boronic acid (36.6 mg, 0.300 mmol) at 50 °C to provide by column chromatography (DCM to 3% $NH_{3(MeOH;\ 2M)}$) the title compound as a brown oil (5.1 mg, 0.008 mmol, 8% yield).

^{1}H NMR (700 MHz, $CDCl_3$) δ (ppm) 7.54 (d, *J* = 7.4 Hz, 2H), 7.46 (t, *J* = 7.6 Hz, 2H), 7.38 (t, *J* = 7.4 Hz, 1H), 7.33 – 7.25 (m, 3H), 7.12 (d, *J* = 7.5 Hz, 1H), 6.80 (s, 1H), 6.67 (s, 1H), 6.59 (s, 1H), 6.50 (s, 1H), 4.78 (d, *J* = 5.1 Hz,

1H), 3.90 (s, 3H), 3.85 (s, 2H), 3.84 (s, 3H), 3.81 (s, 3H), 3.80 – 3.76 (m, 2H), 3.74 (app q, *J* = 6.8 Hz, 1H), 3.63 – 3.55 (m, 2H), 3.44 – 3.34 (m, 1H), 3.22 – 3.13 (m, 1H), 2.95 – 2.87 (m, 2H), 2.41 (dd, *J* = 12.9, 7.0 Hz, 1H), 2.31 (dd, *J* = 12.8, 7.9 Hz, 1H), 2.27 – 2.20 (m, 4H), 2.20 – 2.12 (m, 1H), 1.55 – 1.43 (m, 2H); **HMRS-ESI** (m/z): found $[M+H]^+$ 621.3328, $C_{39}H_{45}N_2O_5$ requires 621.3328.

5. HPLC, GC, and NMR traces

N,*N*-Dimethyl-1-((1*R*,2*S*)-2-phenylcyclopropyl)methanamine (3a)

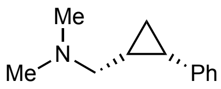

3a

^{1}H-NMR analysis:

racemic

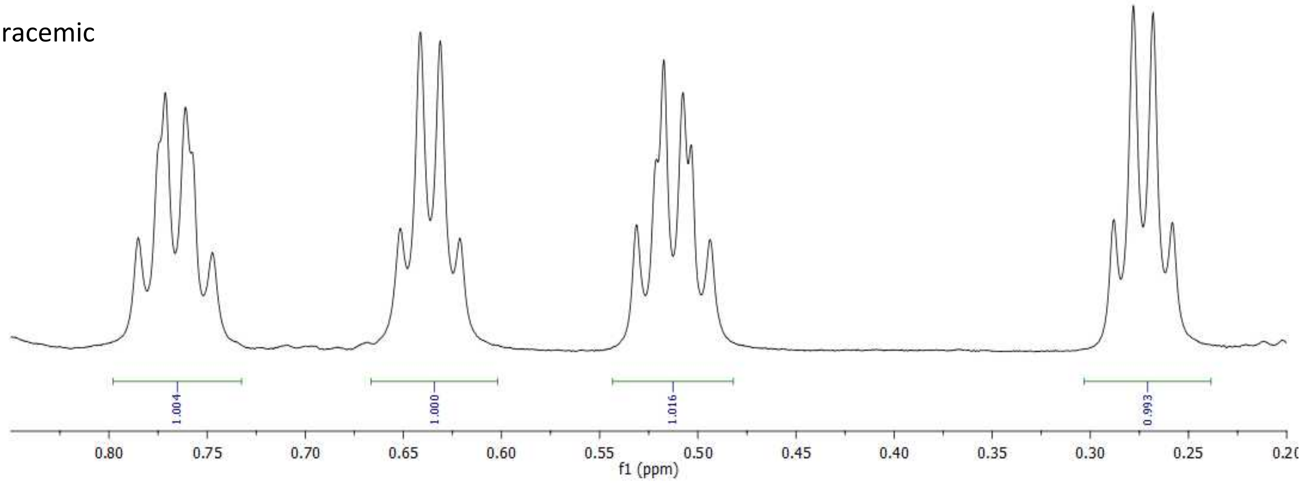

with (*S*)-BINPHAT

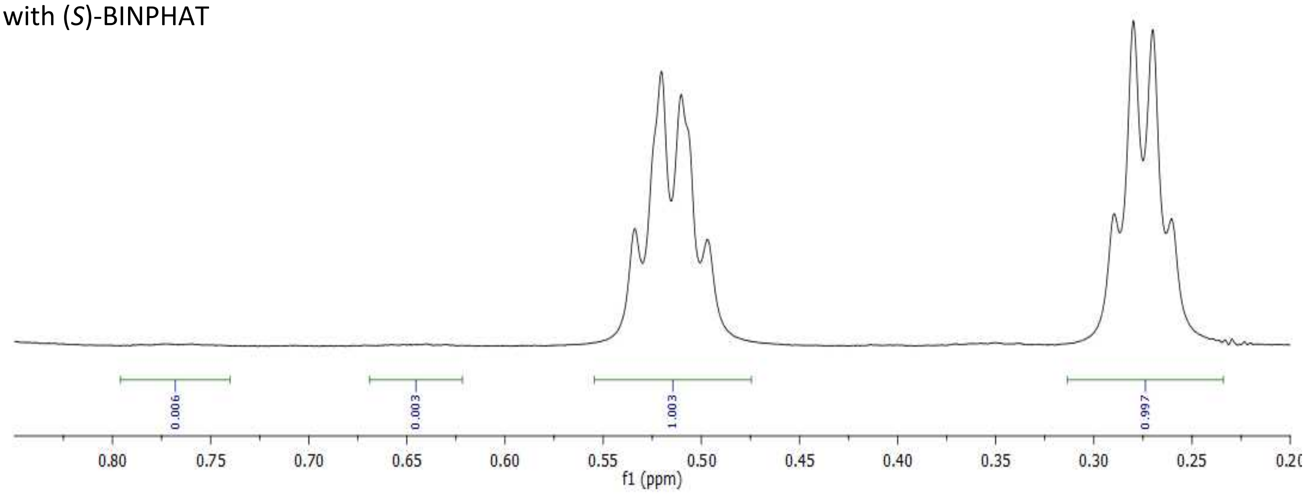

with (*R*)-BINPHAT

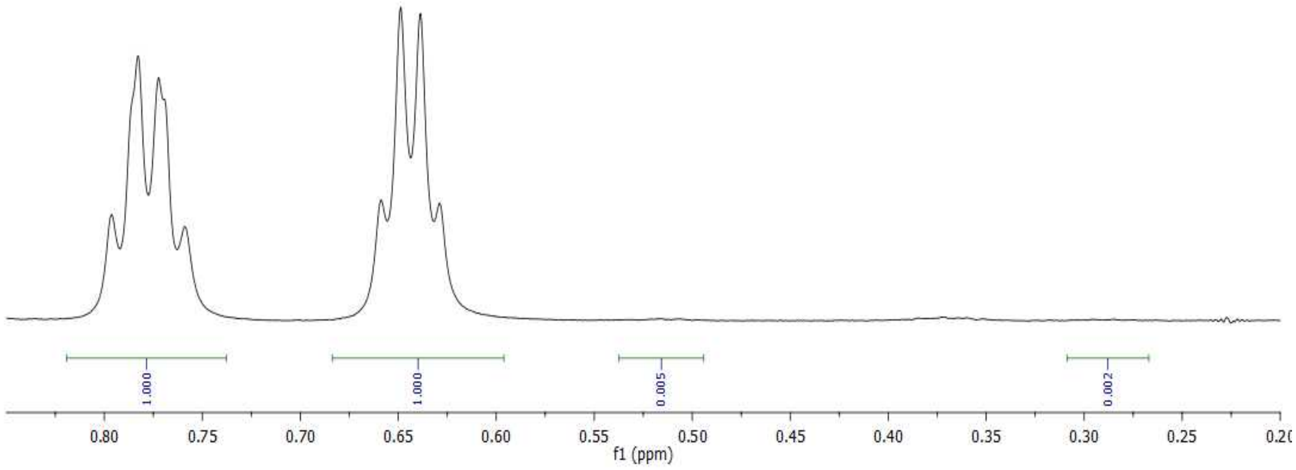

HPLC analysis: Chiralpak AD-H (hexane(0.1% DEA):2-propanol 98:2, 1.0 mL·min^{-1}, 30 °C) t_R = 5.1 min (major, 99.8%), t_R = 5.5 min (minor, 0.2%)

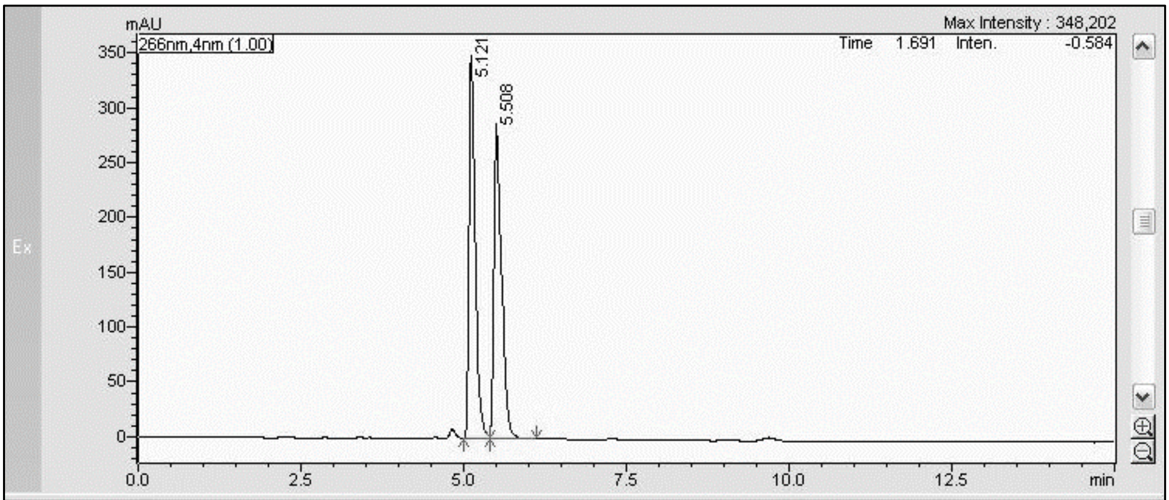

Results View - Peak Table

Peak Table | Compound | Group | Calibration Curve

Peak#	Ret. Time	Area	Height	Area%
1	5.121	2348681	349743	50.120
2	5.508	2337395	287059	49.880
Total		4686076	636802	100.000

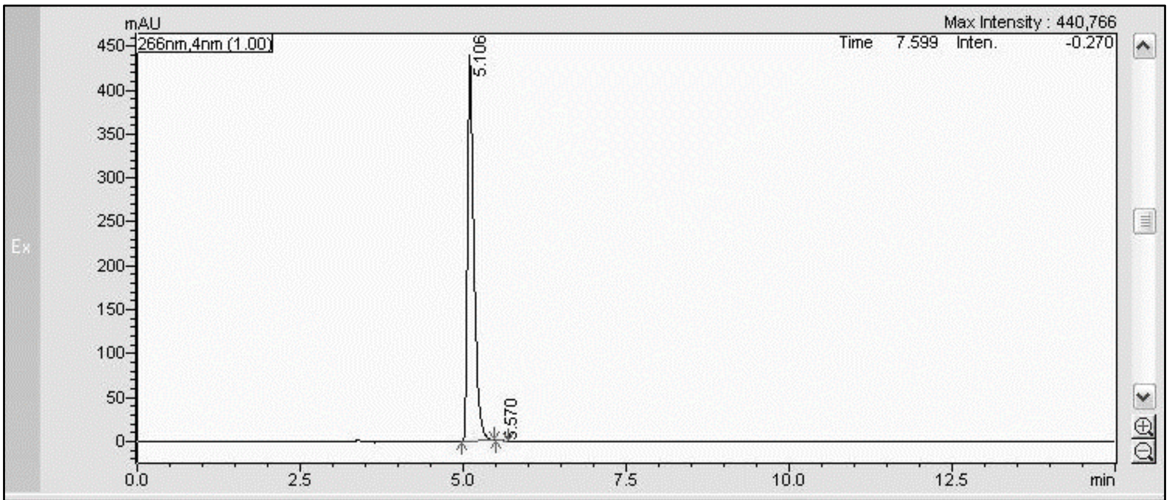

Results View - Peak Table

Peak Table | Compound | Group | Calibration Curve

Peak#	Ret. Time	Area	Height	Area%
1	5.106	3025755	440799	99.801
2	5.570	6039	1297	0.199
Total		3031795	442096	100.000

1-(((1*R*,2*S*)-2-Phenylcyclopropyl)methyl)piperidine (3b)

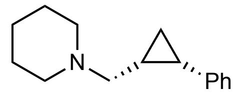

3b

^{1}H-NMR analysis:

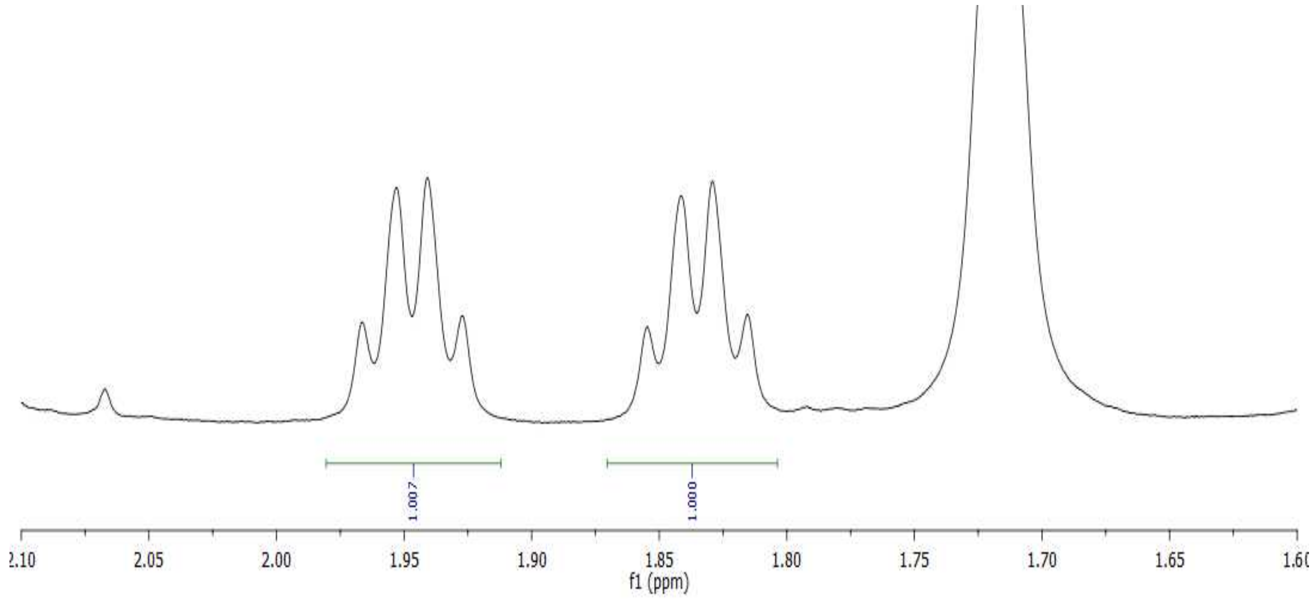

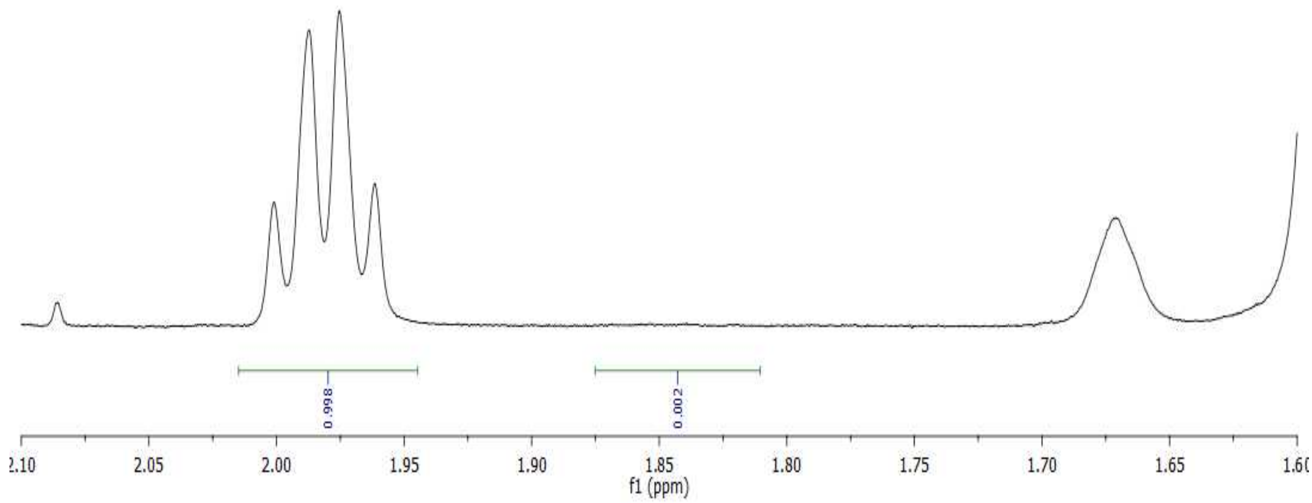

***tert*-Butyl (1-(((1*R*,2*S*)-2-phenylcyclopropyl)methyl)piperidin-4-yl)carbamate (3c)**

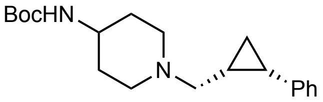

3c

HPLC analysis: Chiralpak AD-H (hexane(0.1% DEA):2-propanol 98:2, 1.0 mL·min^{-1}, 45 °C) t_R = 17.9 min (minor, <0.1%), t_R = 18.7 min (major, >99.9%)

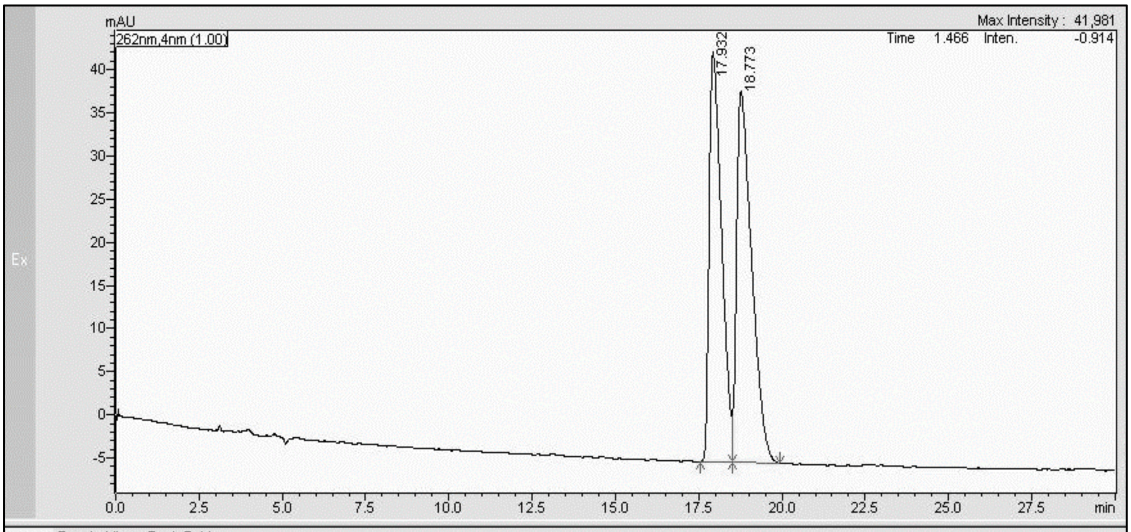

Peak#	Ret. Time	Area	Height	Area%
1	17.932	1225705	47414	47.358
2	18.773	1362445	42977	52.642
Total		2588150	90391	100.000

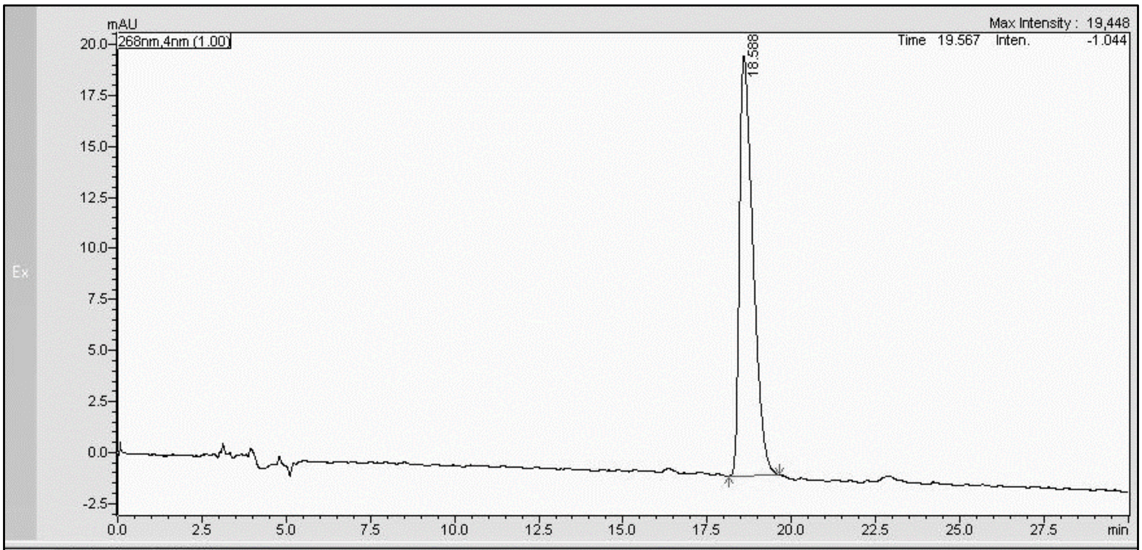

Peak#	Ret. Time	Area	Height	Area%
1	18.588	577605	20590	100.000
Total		577605	20590	100.000

1-(((1*R*,2*S*)-2-Phenylcyclopropyl)methyl)-4-tosylpiperazine (3d)

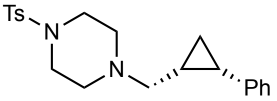

3d

HPLC analysis: Chiralpak AD-H (hexane:2-propanol 90:10, 1.0 mL·min^{-1}, 30 °C) t_R = 16.3 min (major, 98.6%), t_R = 19.2 min (minor, 1.4%)

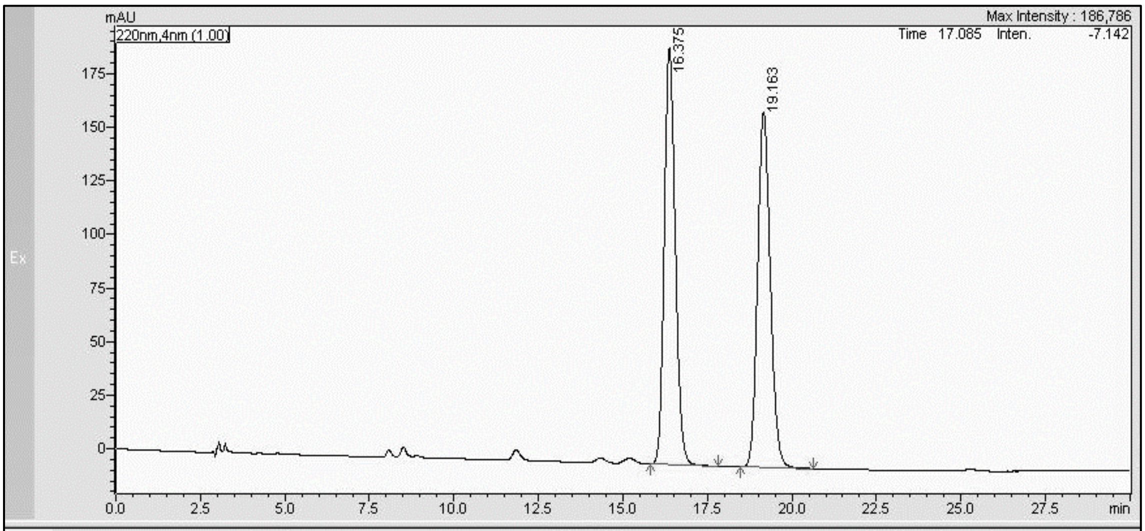

Results View - Peak Table

Peak Table | Compound | Group | Calibration Curve

Peak#	Ret. Time	Area	Height	Area%
1	16.375	4399813	194308	50.291
2	19.163	4348926	165790	49.709
Total		8748740	360098	100.000

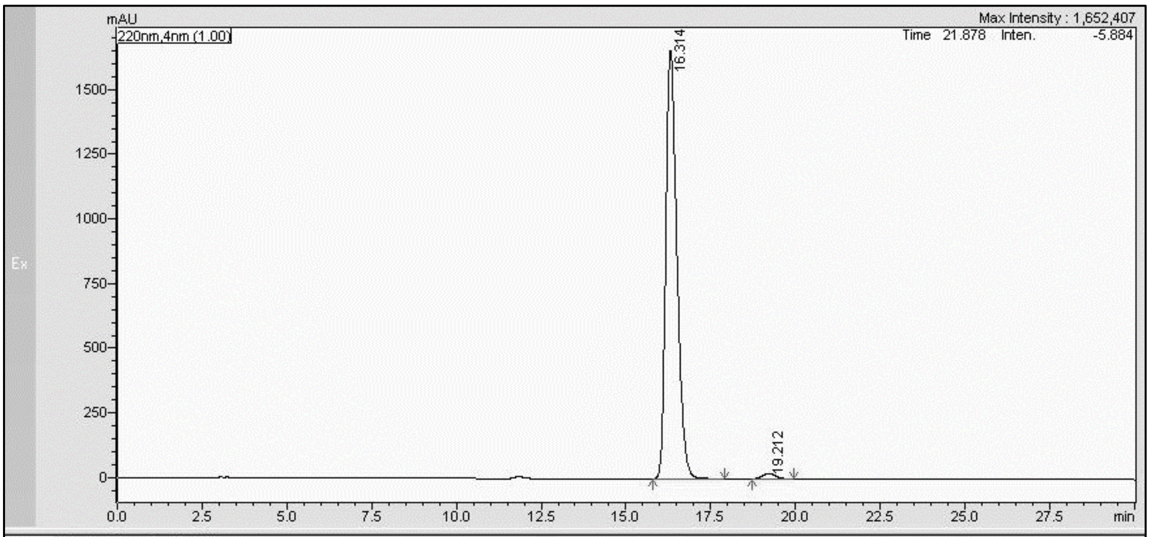

Results View - Peak Table

Peak Table | Compound | Group | Calibration Curve

Peak#	Ret. Time	Area	Height	Area%
1	16.314	37612537	1656764	98.595
2	19.212	535881	20880	1.405
Total		38148417	1677644	100.000

(2*R*,6*S*)-2,6-dimethyl-4-(((1*R*,2*S*)-2-phenylcyclopropyl)methyl)morpholine (3e)

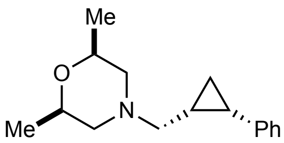

3e

HPLC analysis: Chiralpak AD-H (hexane:2-propanol 99.5:0.5, 1.0 mL·min^{-1}, 30 ºC) t_R = 5.8 min (major, 98.8%), t_R = 7.0 min (minor, 1.2%)

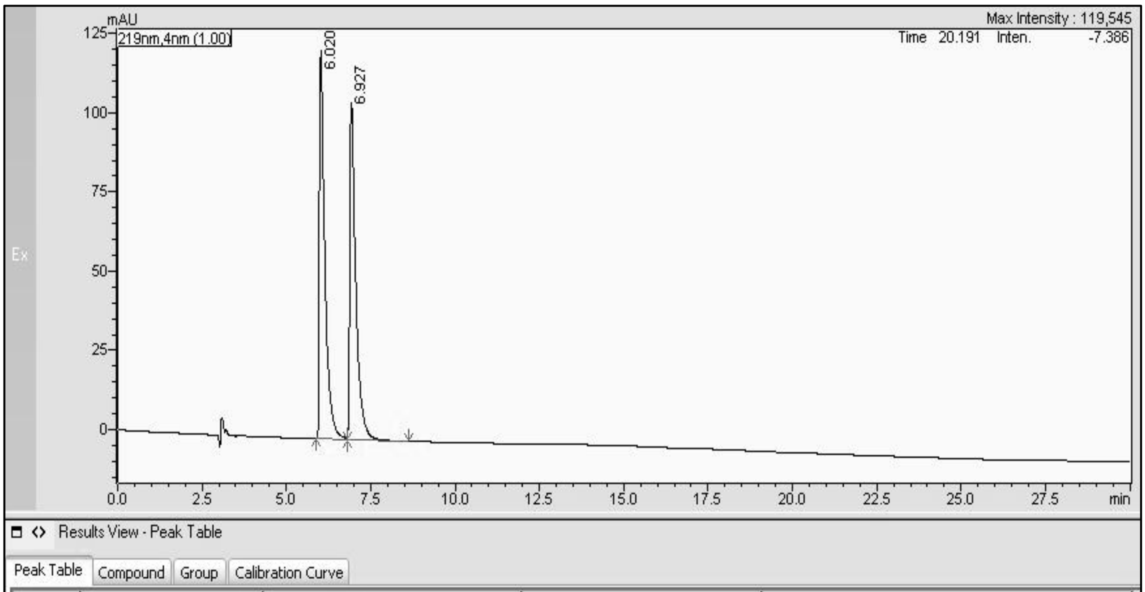

Peak#	Ret. Time	Area	Height	Area%
1	6.020	1404905	122497	51.283
2	6.927	1334597	106182	48.717
Total		2739503	228678	100.000

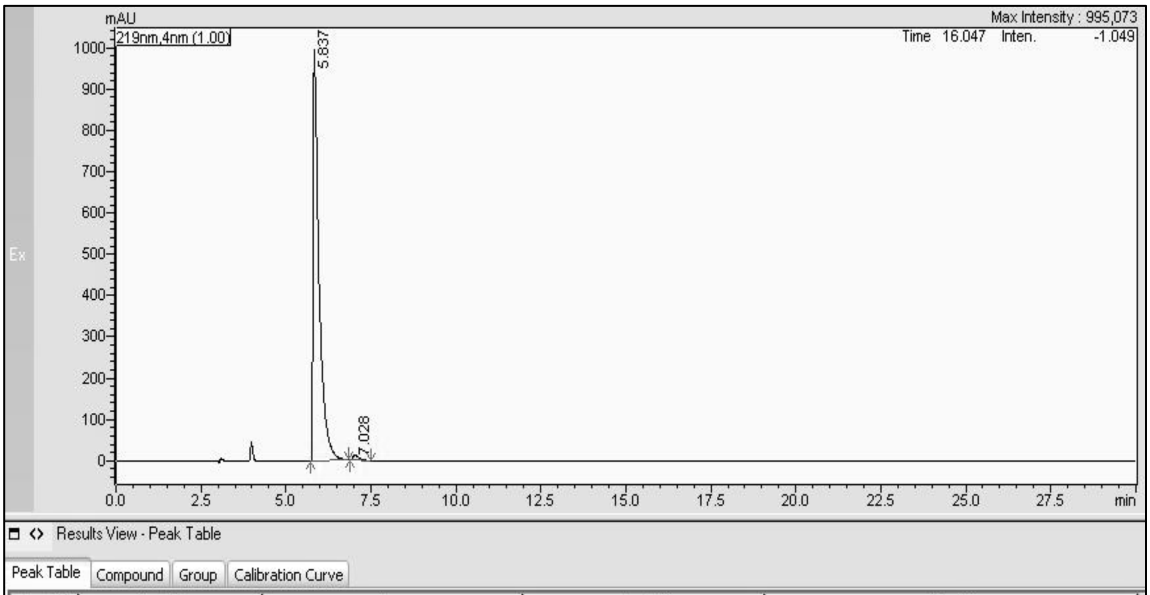

Peak#	Ret. Time	Area	Height	Area%
1	5.837	12391020	995456	98.811
2	7.028	149086	12041	1.189
Total		12540106	1007497	100.000

1'-(((1*R*,2*S*)-2-Phenylcyclopropyl)methyl)spiro[chromane-2,4'-piperidin]-4-one (3f)

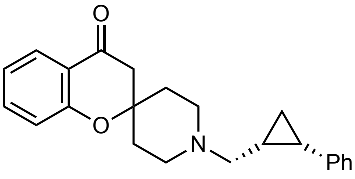

3f

HPLC analysis: Chiralpak AD-H (hexane:2-propanol 97:3, 1.0 mL·min^{-1}, 30 °C) t_R = 14.5 min (major, 99.8%), t_R = 16.4 min (minor, 0.2%)

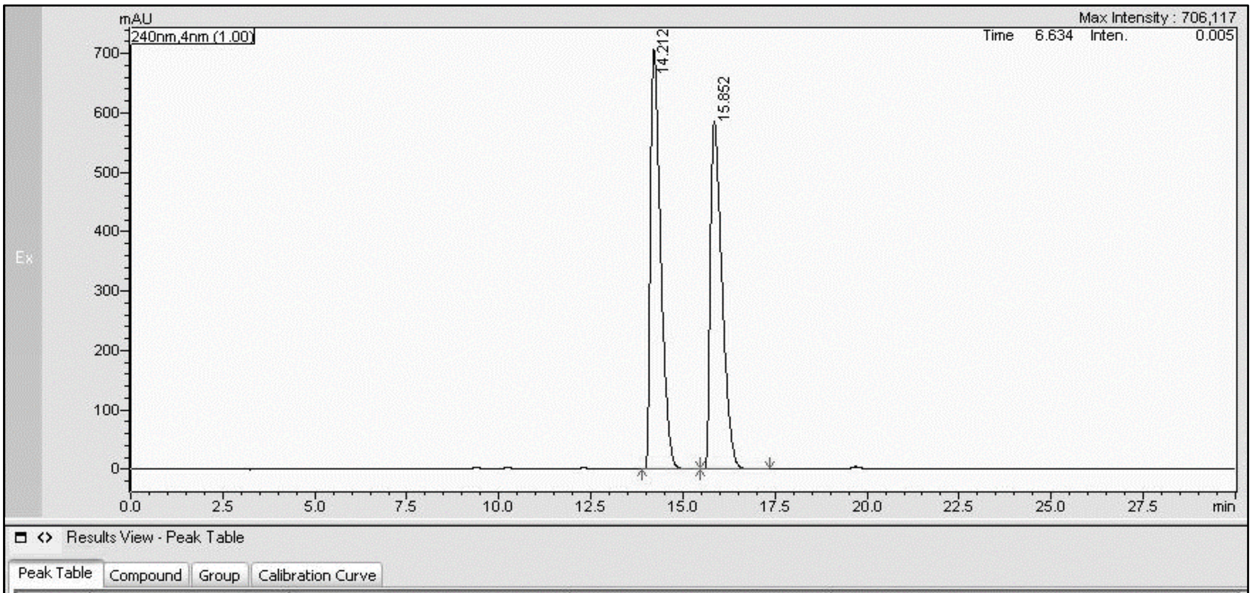

Peak#	Ret. Time	Area	Height	Area%
1	14.212	13718312	706168	51.486
2	15.852	12926623	585226	48.514
Total		26644935	1291394	100.000

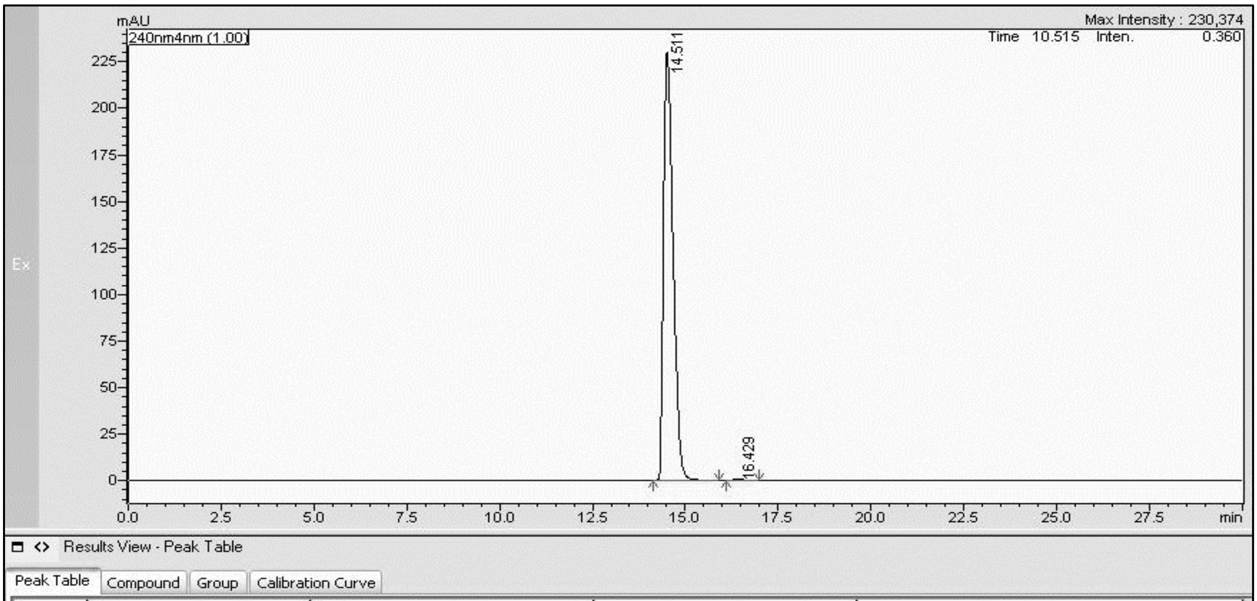

Peak#	Ret. Time	Area	Height	Area%
1	14.511	4140731	230092	99.799
2	16.429	8324	385	0.201
Total		4149056	230477	100.000

4-(4-Chlorophenyl)-1-(((1*R*,2*S*)-2-phenylcyclopropyl)methyl)piperidin-4-ol (3g)

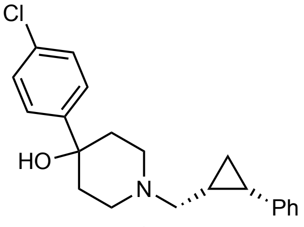

3g

HPLC analysis: Chiralpak AD-H (hexane:2-propanol 95:5, 1.0 mL·min^{-1}, 30 °C) t_R = 24.2 min (minor, 0.9%), t_R = 26.1 min (major, 99.1%)

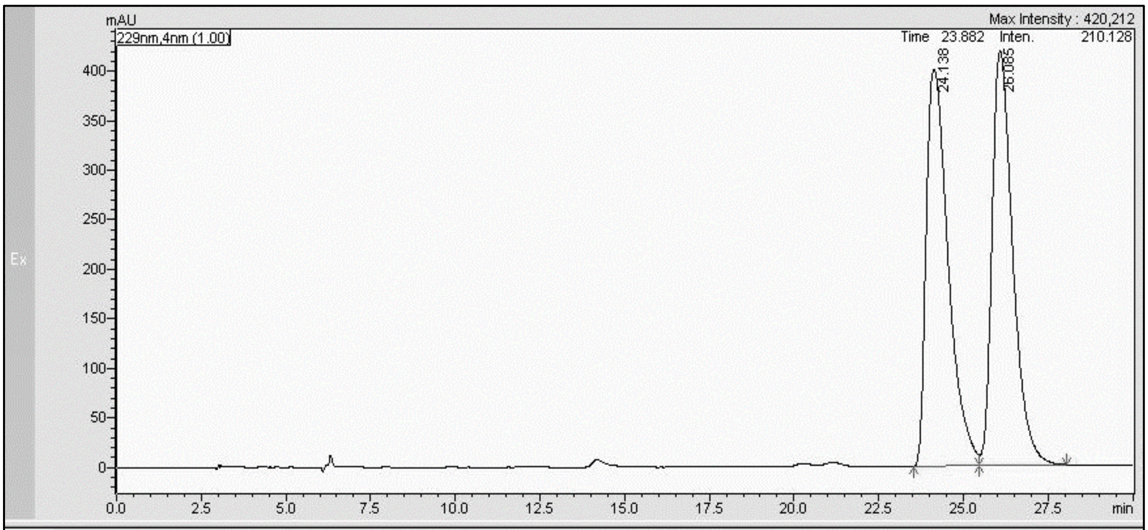

Results View - Peak Table

Peak Table | Compound | Group | Calibration Curve

Peak#	Ret. Time	Area	Height	Area%
1	24.138	18179690	400610	50.498
2	26.085	17820990	418130	49.502
Total		36000680	818740	100.000

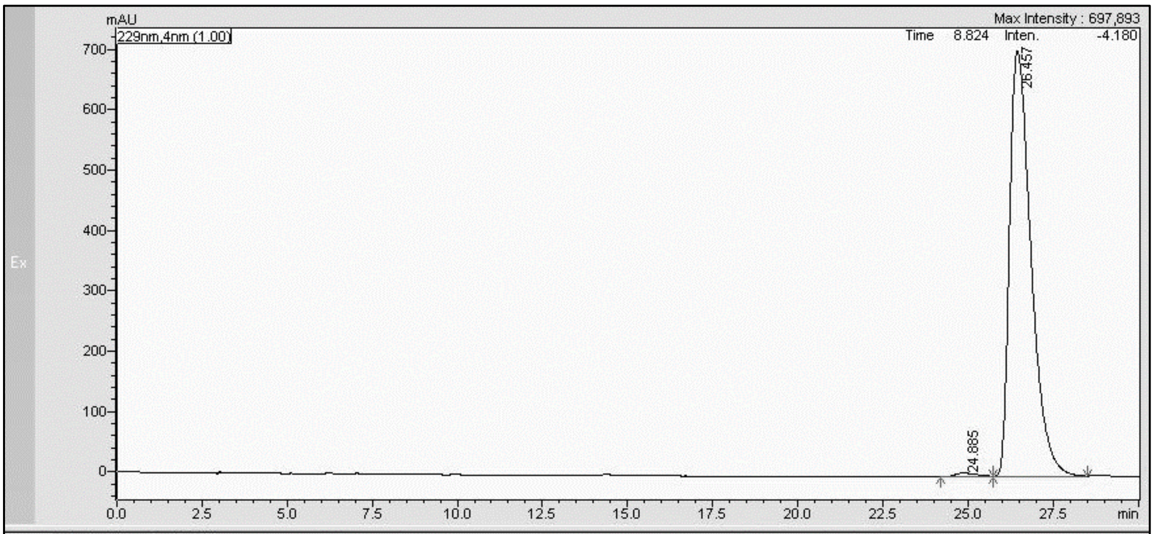

Results View - Peak Table

Peak Table | Compound | Group | Calibration Curve

Peak#	Ret. Time	Area	Height	Area%
1	24.885	270741	6118	0.868
2	26.457	30909371	705427	99.132
Total		31180112	711546	100.000

6-Fluoro-3-(1-(((1*R*,2*S*)-2-phenylcyclopropyl)methyl)piperidin-4-yl)benzo[*d*]isoxazole (3h)

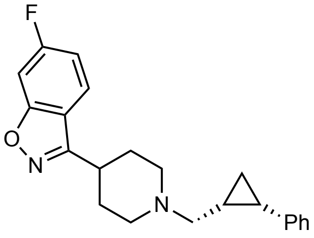

3h

HPLC analysis: Chiralpak AD-H (hexane(0.1% DEA):2-propanol 90:10, 1.0 mL·min^{-1}, 30 °C) t_R = 6.2 min (major, 99.3%), t_R = 8.2 min (minor, 0.7%)

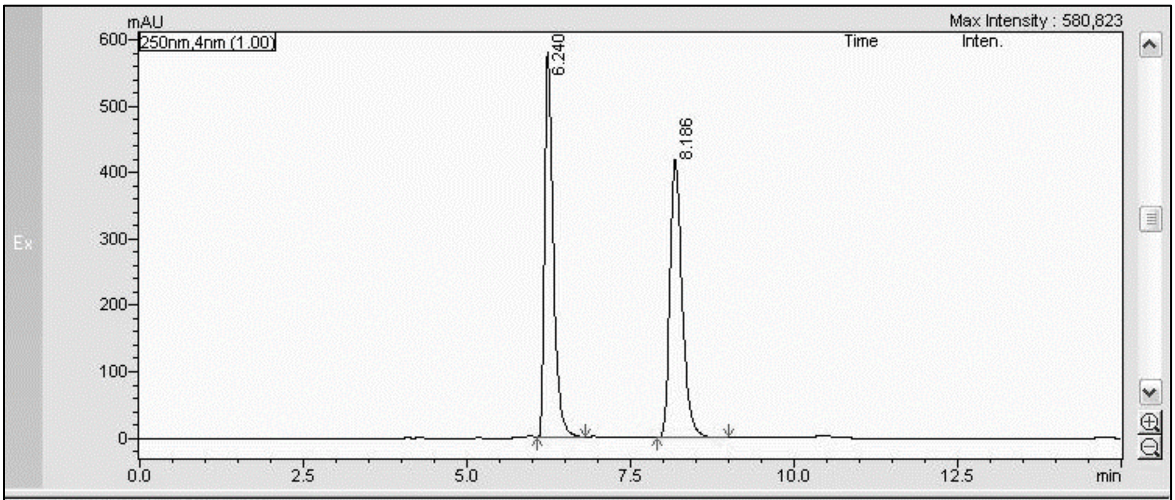

Results View - Peak Table

Peak Table | Compound | Group | Calibration Curve

Peak#	Ret. Time	Area	Height	Area%
1	6.240	5337513	579249	49.233
2	8.186	5503841	419122	50.767
Total		10841354	998370	100.000

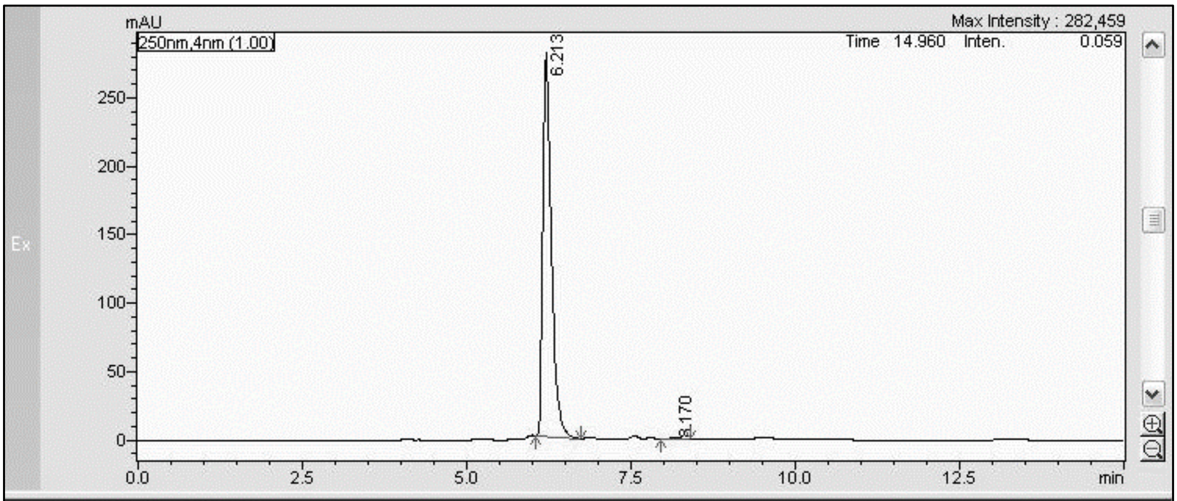

Results View - Peak Table

Peak Table | Compound | Group | Calibration Curve

Peak#	Ret. Time	Area	Height	Area%
1	6.213	2532025	280129	99.256
2	8.170	18981	1756	0.744
Total		2551005	281885	100.000

1-(((1*R*,2*S*)-2-Phenylcyclopropyl)methyl)pyrrolidine (3i)

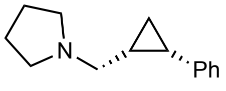

3i

^{1}H-NMR analysis:

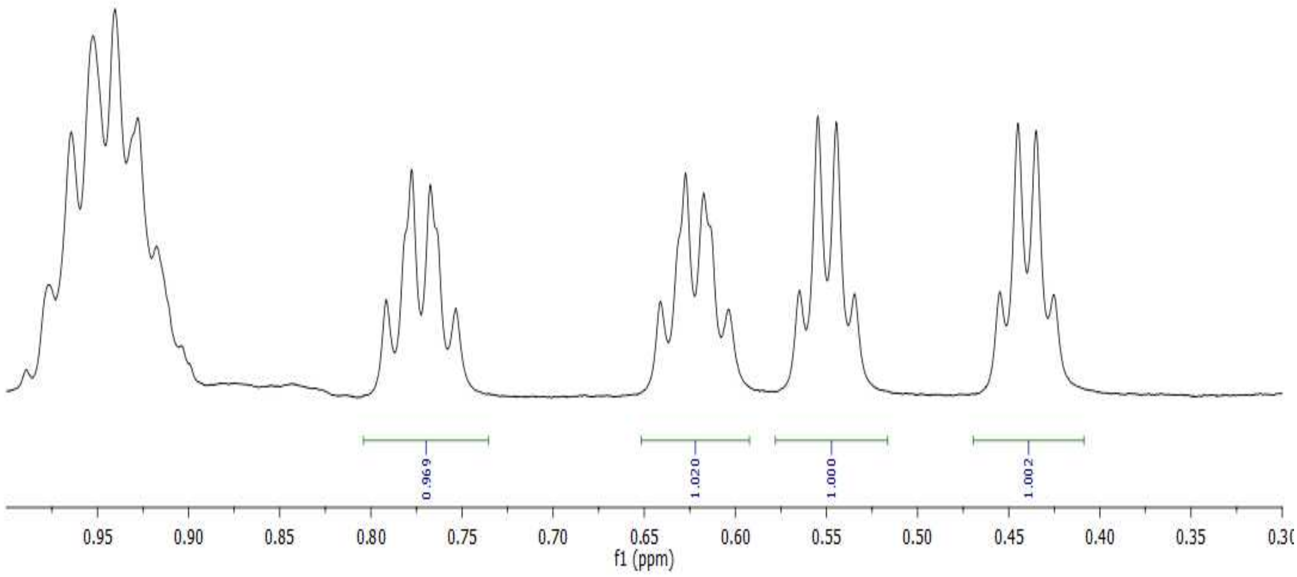

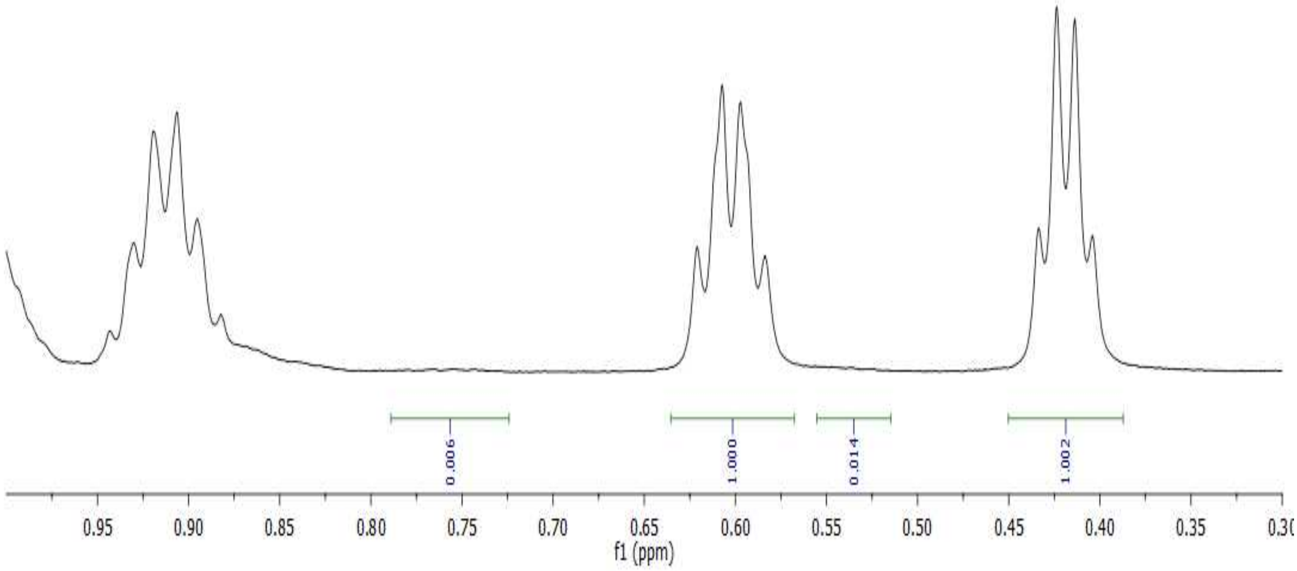

Methyl 1-(((1*R*,2*S*)-2-phenylcyclopropyl)methyl)azetidine-3-carboxylate (3j)

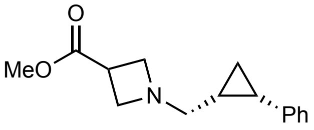

3j

HPLC analysis: Chiralpak AD-H (hexane:2-propanol 97:3, 1.0 mL·min^{-1}, 30 °C) t_R = 7.8 min (major, 99.0%), t_R = 8.8 min (minor, 1.0%)

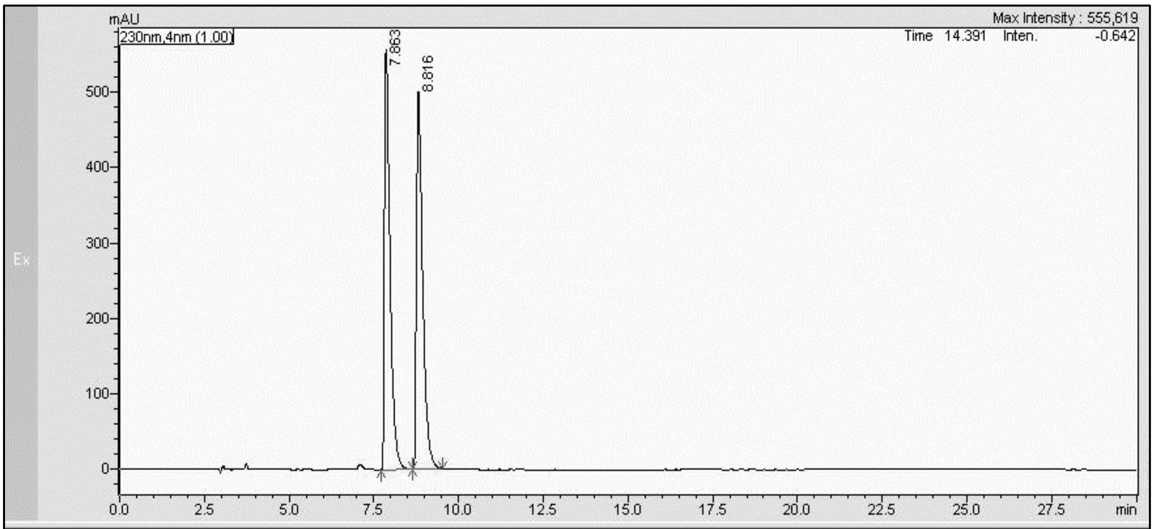

Results View - Peak Table

Peak Table | Compound | Group | Calibration Curve

Peak#	Ret. Time	Area	Height	Area%
1	7.863	6420563	556110	50.131
2	8.816	6386964	499544	49.869
Total		12807528	1055654	100.000

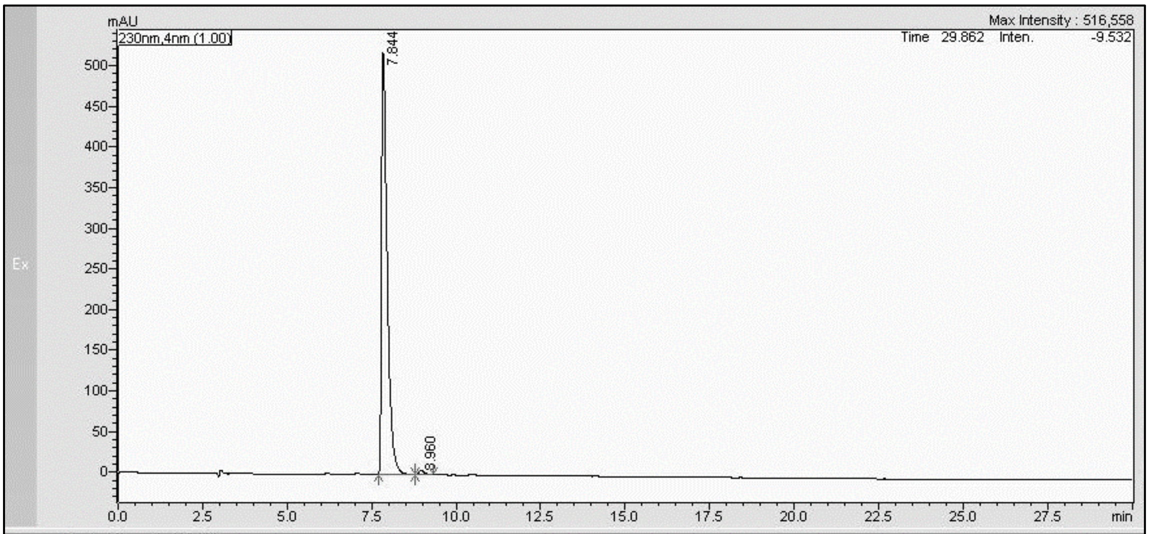

Results View - Peak Table

Peak Table | Compound | Group | Calibration Curve

Peak#	Ret. Time	Area	Height	Area%
1	7.844	6135595	519623	98.967
2	8.960	64033	5282	1.033
Total		6199628	524905	100.000

6-(((1*R*,2*S*)-2-phenylcyclopropyl)methyl)-2-oxa-6-azaspiro[3.3]heptane (3k)

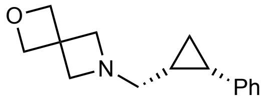

3k

HPLC analysis: Chiralpak AD-H (hexane(0.1% DEA):2-propanol 97:3, 1.0 mL·min^{-1}, 30 °C) t_R = 9.4 min (major, >99.9%), t_R = 11.0 min (minor, <0.1%)

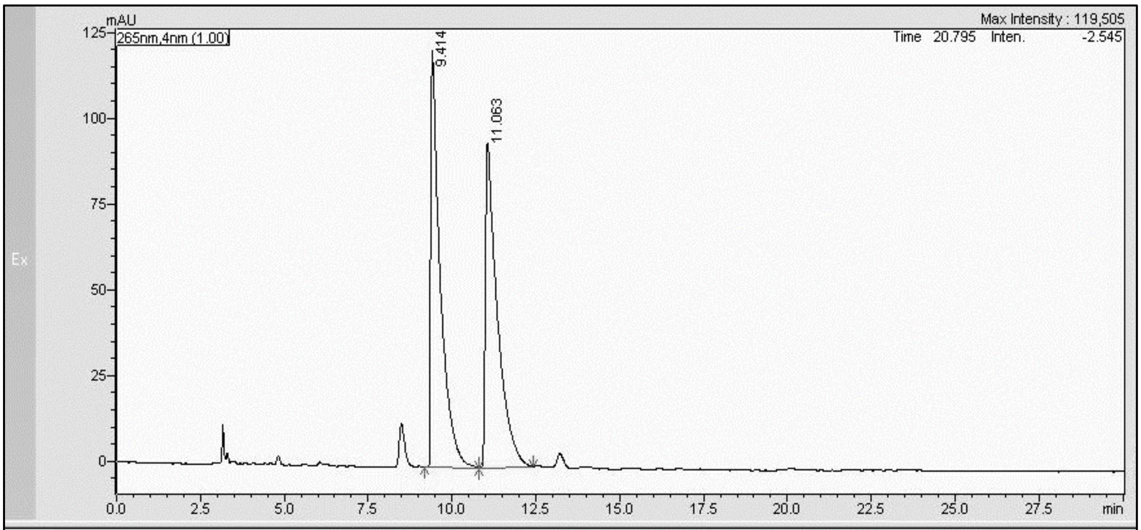

Results View - Peak Table

Peak Table | Compound | Group | Calibration Curve

Peak#	Ret. Time	Area	Height	Area%
1	9.414	2378319	121321	50.534
2	11.063	2328026	94463	49.466
Total		4706345	215784	100.000

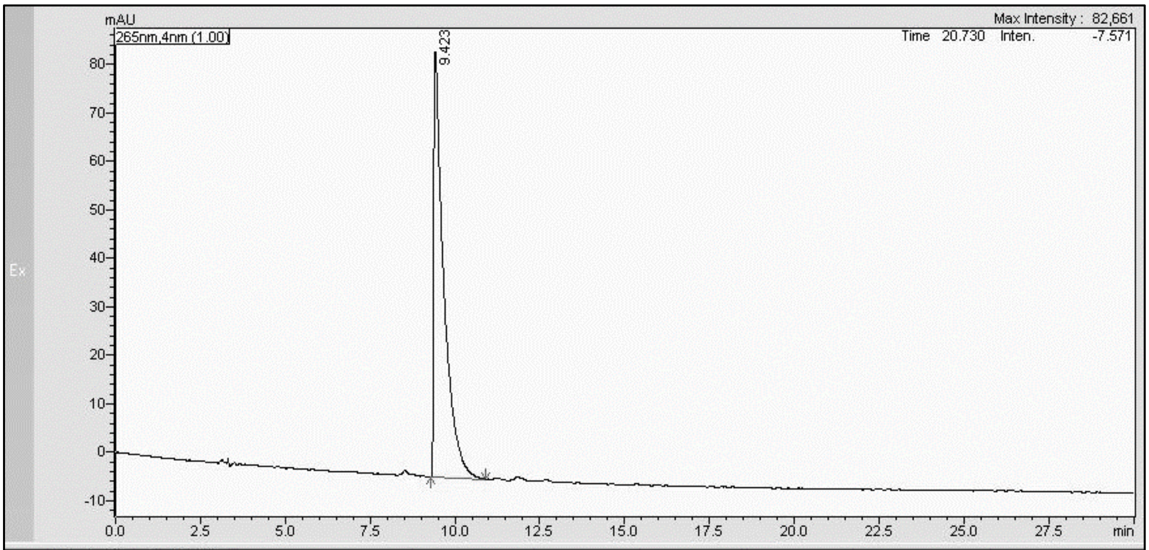

Results View - Peak Table

Peak Table | Compound | Group | Calibration Curve

Peak#	Ret. Time	Area	Height	Area%
1	9.423	1829787	87818	100.000
Total		1829787	87818	100.000

Ethyl 3-(methyl(((1*R*,2*S*)-2-phenylcyclopropyl)methyl)amino)propanoate (3m)

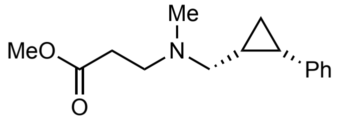

3m

HPLC analysis: Chiralpak AD-H (hexane:2-propanol 99.5:0.5, 1.0 mL·min^{-1}, 30 °C) t_R = 13.1 min (major, 96.2%), t_R = 17.2 min (minor, 3.8%)

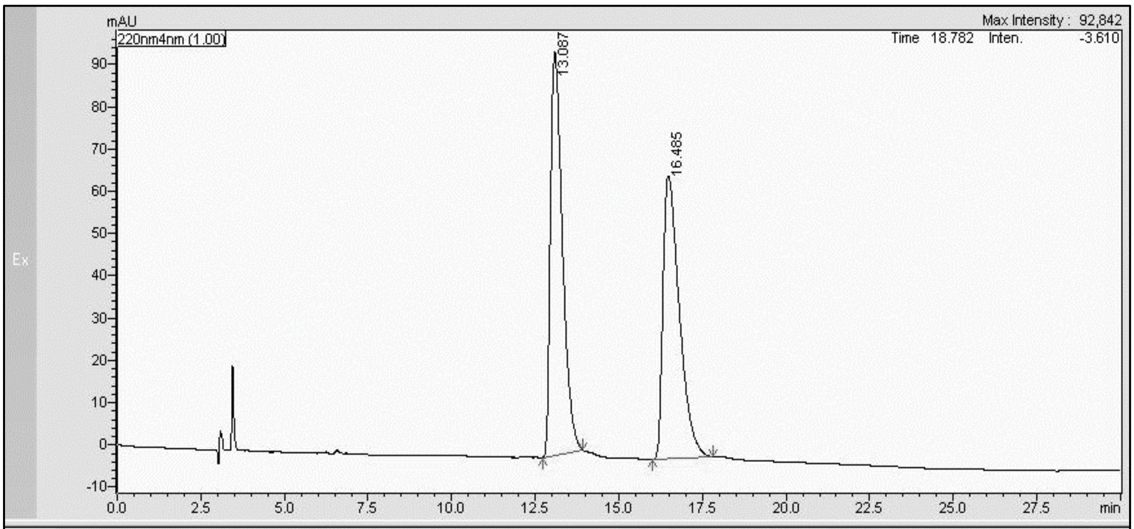

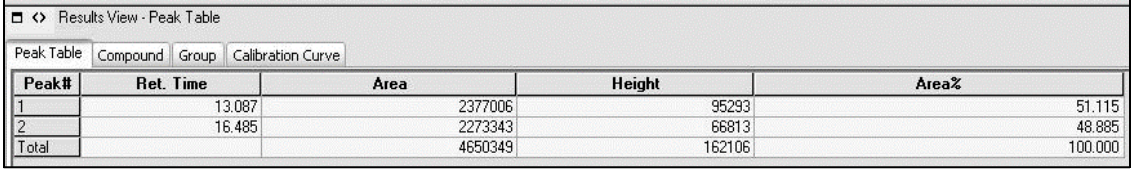

Peak#	Ret. Time	Area	Height	Area%
1	13.087	2377006	95293	51.115
2	16.485	2273343	66813	48.885
Total		4650349	162106	100.000

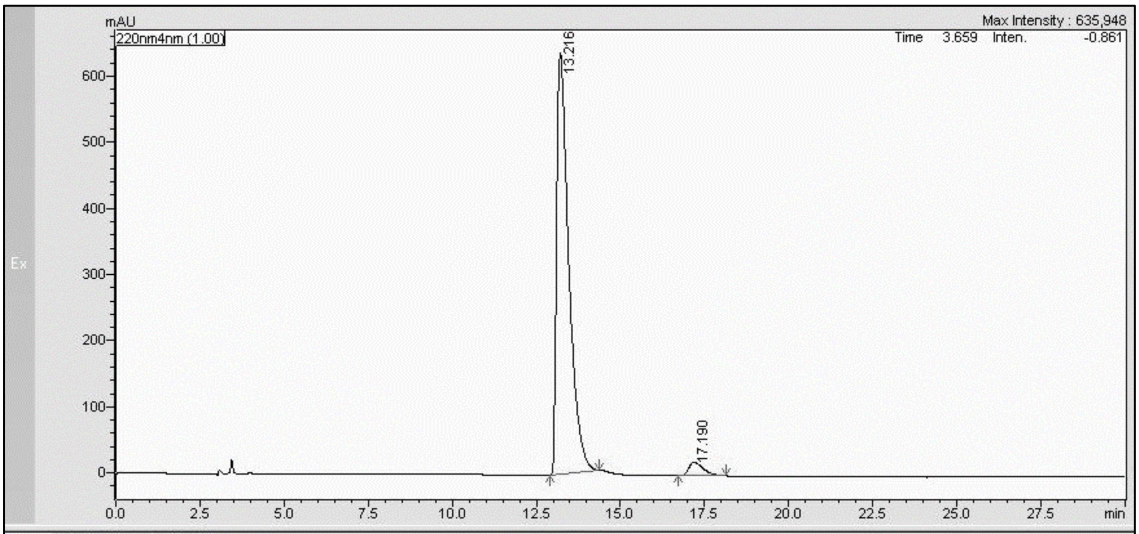

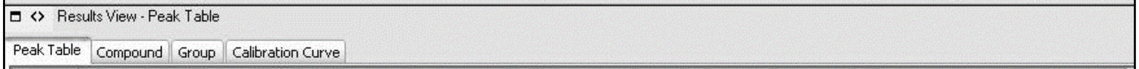

Peak#	Ret. Time	Area	Height	Area%
1	13.216	16025427	637311	96.224
2	17.190	628796	20824	3.776
Total		16654223	658135	100.000

Benzyl 3-(methyl(((1*R*,2*S*)-2-phenylcyclopropyl)methyl)amino)azetidine-1-carboxylate (3n)

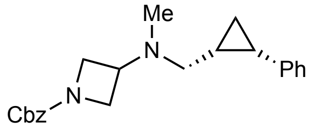

3n

HPLC analysis: Chiralpak AD-H (hexane:2-propanol 97:3, 1.0 mL·min^{-1}, 30 °C) t_R = 20.3 min (major, 91.8%), t_R = 23.1 min (minor, 8.2%)

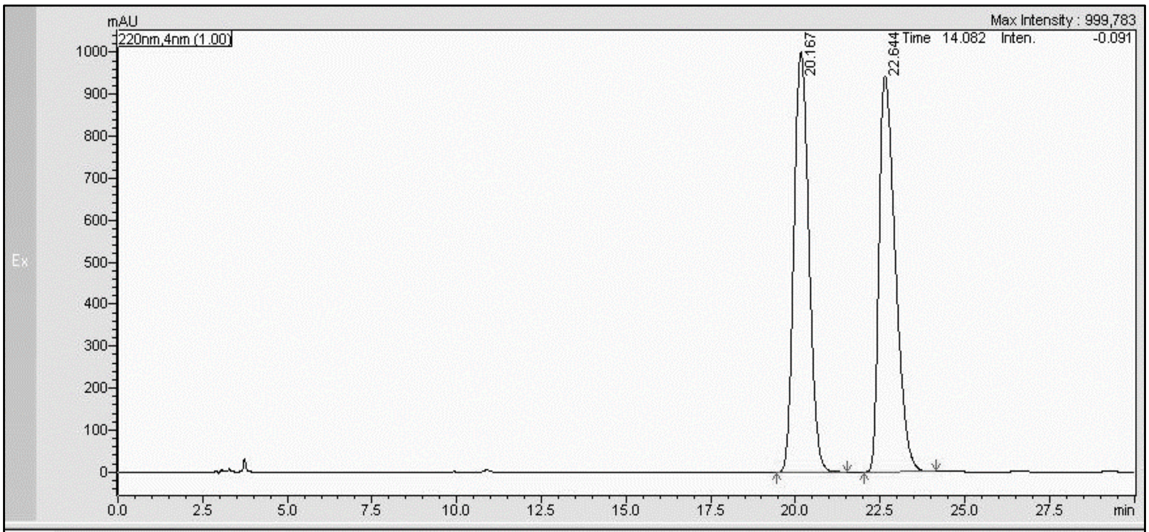

Results View - Peak Table

Peak Table | Compound | Group | Calibration Curve

Peak#	Ret. Time	Area	Height	Area%
1	20.167	30696758	999647	49.300
2	22.644	31568691	942575	50.700
Total		62265449	1942222	100.000

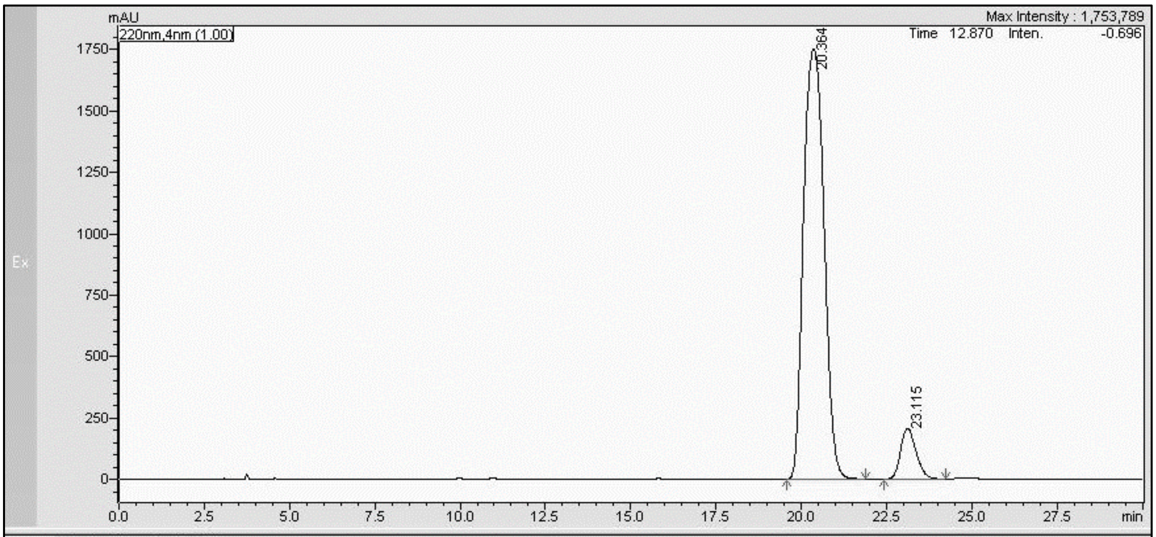

Results View - Peak Table

Peak Table | Compound | Group | Calibration Curve

Peak#	Ret. Time	Area	Height	Area%
1	20.364	73316501	1752288	91.843
2	23.115	6511950	205379	8.157
Total		79828451	1957667	100.000

2-((5-Fluoropyrimidin-2-yl)oxy)-*N*-methyl-*N*-(((1*R*,2*S*)-2-phenylcyclopropyl)methyl)ethan-1-amine (3o)

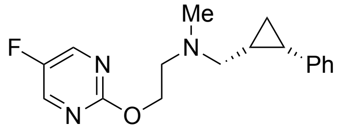

3o

HPLC analysis: Chiralpak AD-H (hexane:2-propanol 98:2, 1.0 mL·min^{-1}, 30 °C) t_R = 13.0 min (major, 89.1%), t_R = 14.4 min (minor, 10.9%)

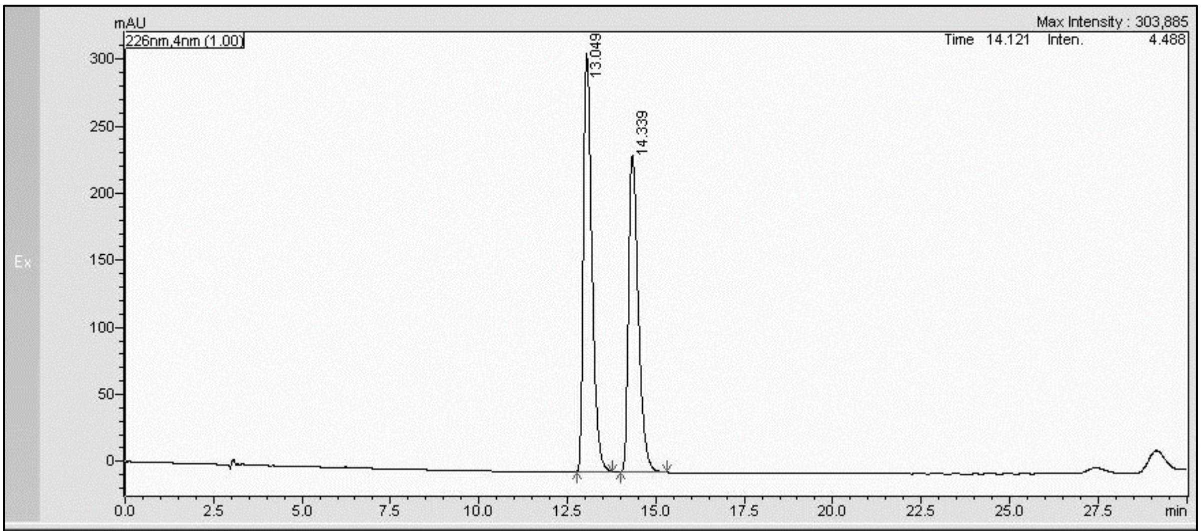

Results View - Peak Table

Peak Table | Compound | Group | Calibration Curve

Peak#	Ret. Time	Area	Height	Area%
1	13.049	5235020	311570	53.415
2	14.339	4565571	236115	46.585
Total		9800590	547685	100.000

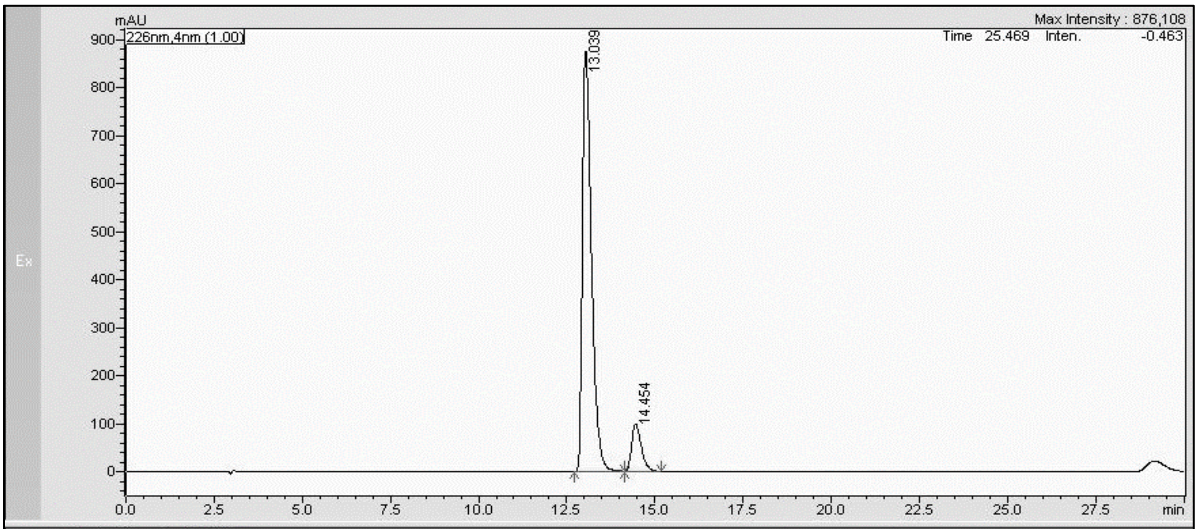

Results View - Peak Table

Peak Table | Compound | Group | Calibration Curve

Peak#	Ret. Time	Area	Height	Area%
1	13.039	15845576	876669	89.125
2	14.454	1933528	100188	10.875
Total		17779104	976857	100.000

_N_-ethyl-_N_-(((1_R_,2_S_)-2-phenylcyclopropyl)methyl)ethanamine (3p)

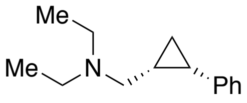

3p

HPLC analysis: Chiralpak AD-H (hexane:2-propanol 98:2, 1.0 mL·min^{-1}, 30 °C) t_R = 16.3 min (major, 98.6%), t_R = 19.2 min (minor, 1.4%)

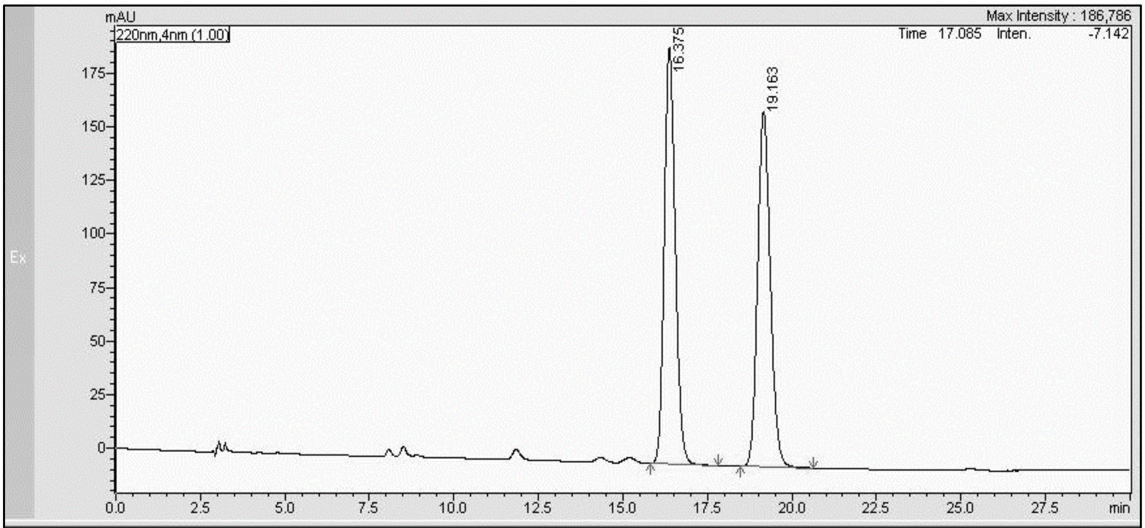

Results View - Peak Table

Peak Table | Compound | Group | Calibration Curve

Peak#	Ret. Time	Area	Height	Area%
1	16.375	4399813	194308	50.291
2	19.163	4348926	165790	49.709
Total		8748740	360098	100.000

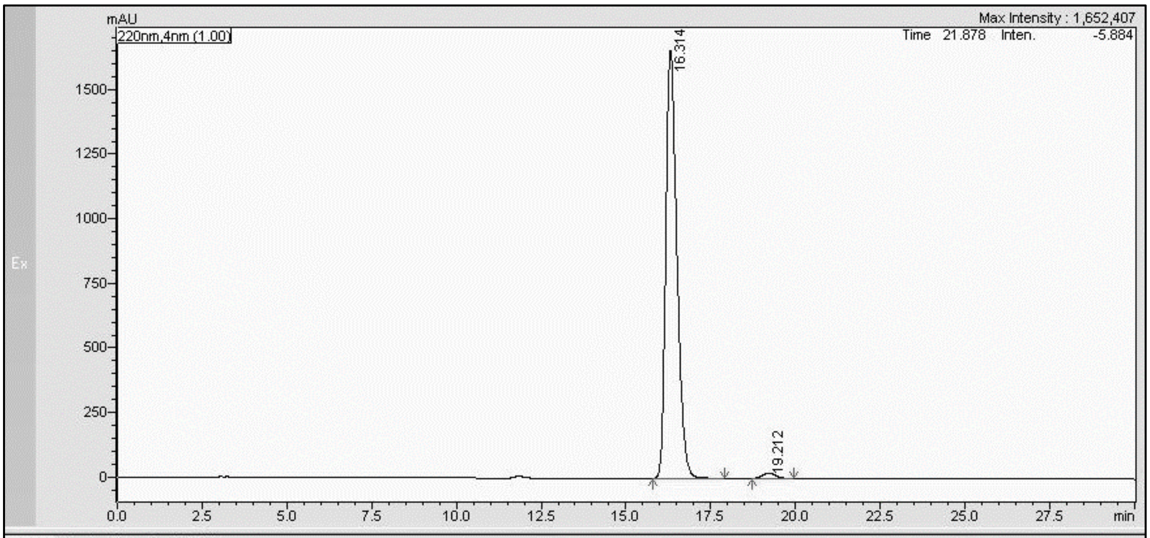

Results View - Peak Table

Peak Table | Compound | Group | Calibration Curve

Peak#	Ret. Time	Area	Height	Area%
1	16.314	37612537	1656764	98.595
2	19.212	535881	20880	1.405
Total		38148417	1677644	100.000

***N*-methyl-*N*-(((1*R*,2*S*)-2-phenylcyclopropyl)methyl)propan-2-amine (3q)**

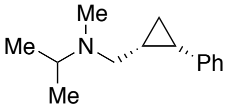

3q

HPLC analysis: Chiralpak AD-H (hexane:2-propanol 98:2, 0.5 mL·min^{-1}, 45 °C) t_R = 18.9 min (major, 98.1%), t_R = 20.7 min (minor, 1.9%)

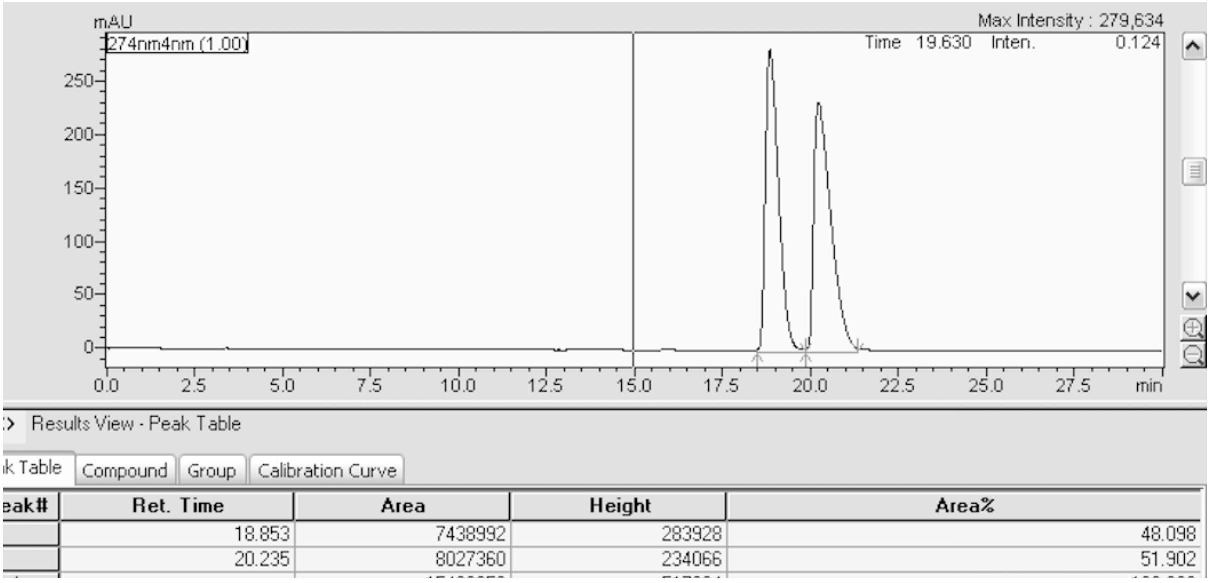

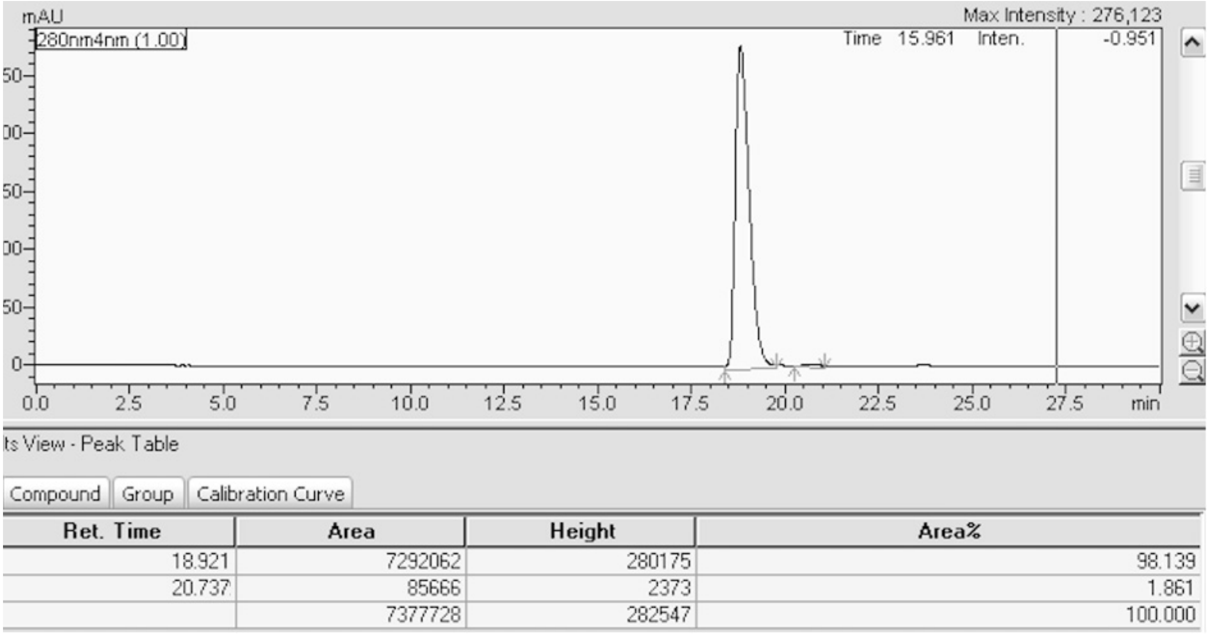

1-((1*S*,2*R*)-1-(2-Methoxyethyl)-2-phenylcyclopropyl)-*N*,*N*-dimethylmethanamine (3r)

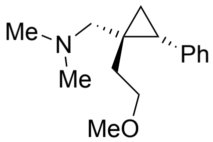

3r

^{1}H-NMR analysis:

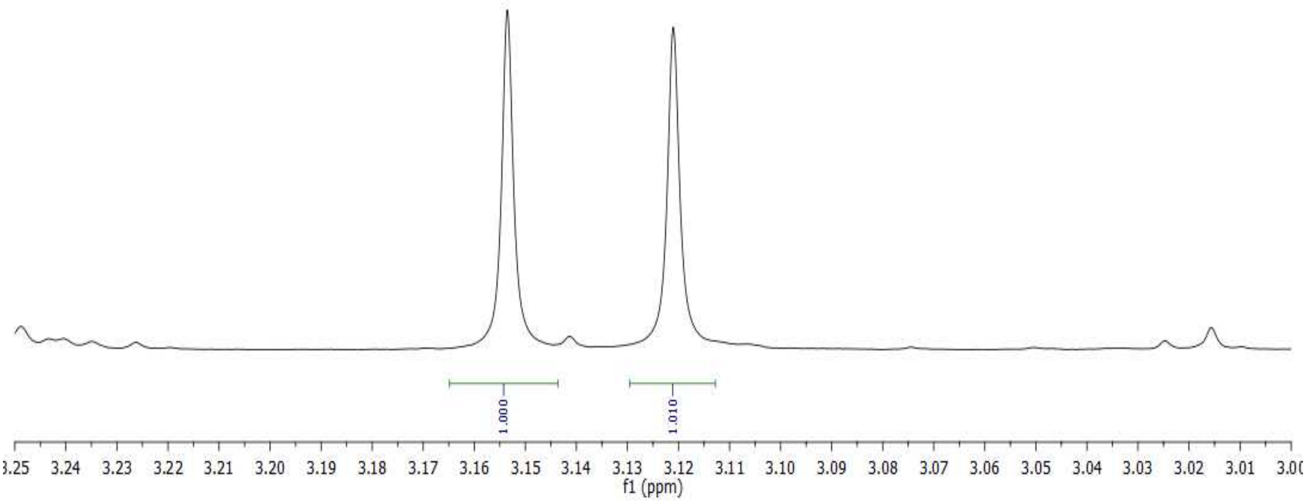

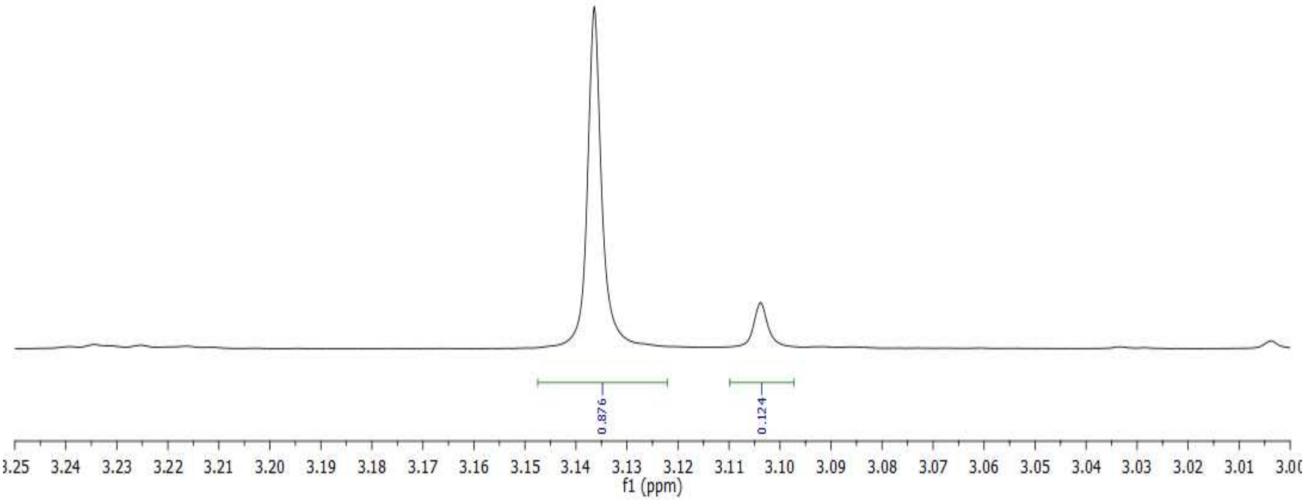

***N,N*-Dimethyl-1-((1*R*,2*R*)-1-methyl-2-phenylcyclopropyl)methanamine (3s)**

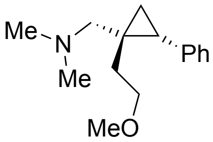

3s

GC-FID analysis: ChiralDex β-DM (50 °C to 150 °C; 5 °C/min; linear velocity 40 cm·s^{-1}; split ratio 10.0) t_R = 9.0 min (minor, 11.1%), t_R = 9.2 min (major, 88.9%)

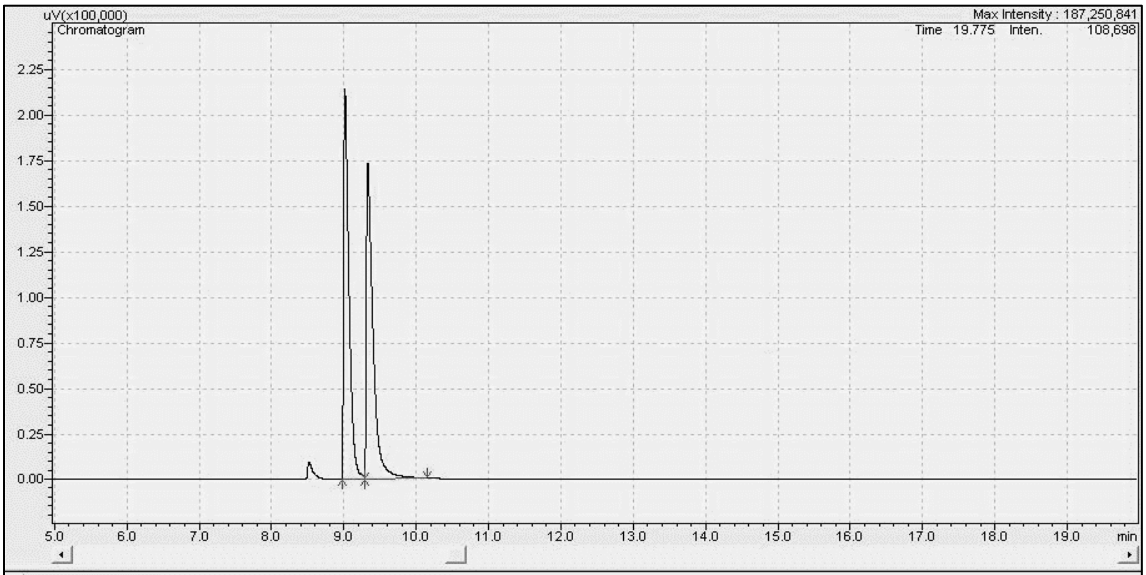

Results - Peak Table

Peak Table | Compound | Group | Calibration Curve

Peak#	Ret.Time	Area	Height	Conc.	Units	Mark	Compound ID#	Compound Name
1	9.016	1103626.0	214260.9	49.62123				
2	9.331	1120474.5	173196.3	50.37877		V		

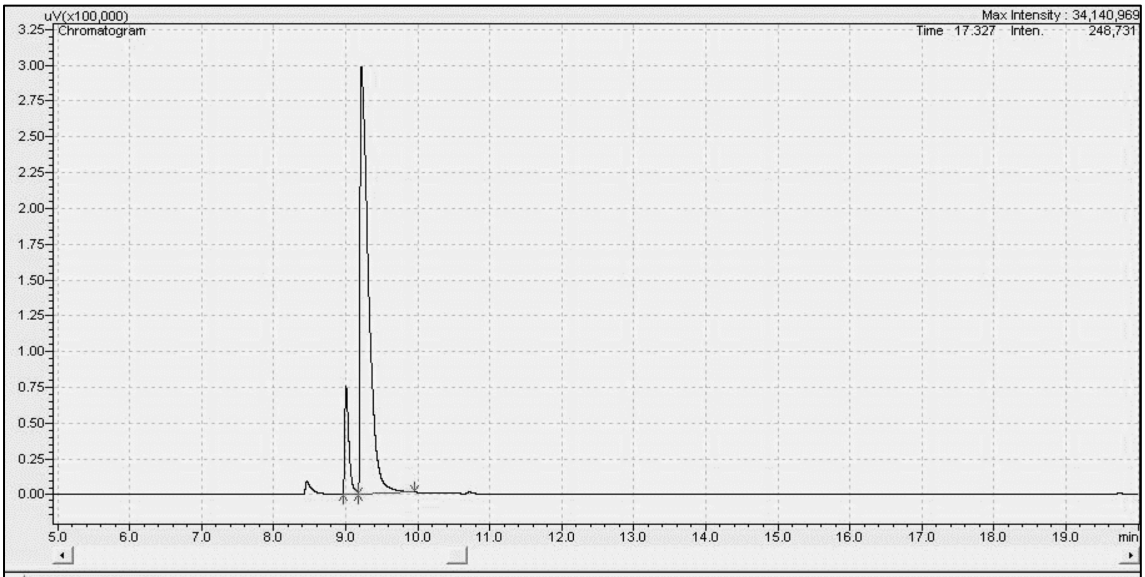

Results - Peak Table

Peak Table | Compound | Group | Calibration Curve

Peak#	Ret.Time	Area	Height	Conc.	Units	Mark	Compound ID#	Compound Name
1	9.007	304270.9	75062.1	11.05783				
2	9.219	2447362.9	298048.2	88.94217		V		

1-((1*R*,2*R*)-1-(4-Chlorophenyl)-2-phenylcyclopropyl)-*N*,*N*-dimethylmethanamine (3t)

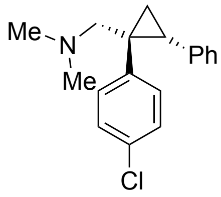

3t

HPLC analysis: Chiralpak AD-H (hexane:2-propanol 99:1, 1.0 mL·min^{-1}, 30 °C) t_R = 8.5 min (major, 95.7%), t_R = 12.4 min (minor, 4.3%)

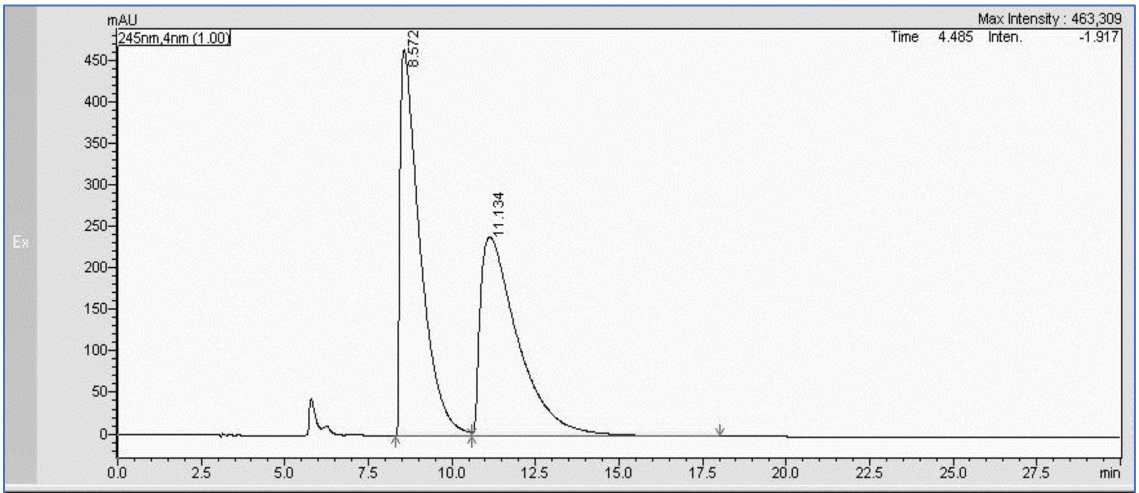

Results View - Peak Table

Peak Table | Compound | Group | Calibration Curve

Peak#	Ret. Time	Area	Height	Area%
1	8.572	19441293	465865	50.710
2	11.134	18896622	240167	49.290
Total		38337915	706032	100.000

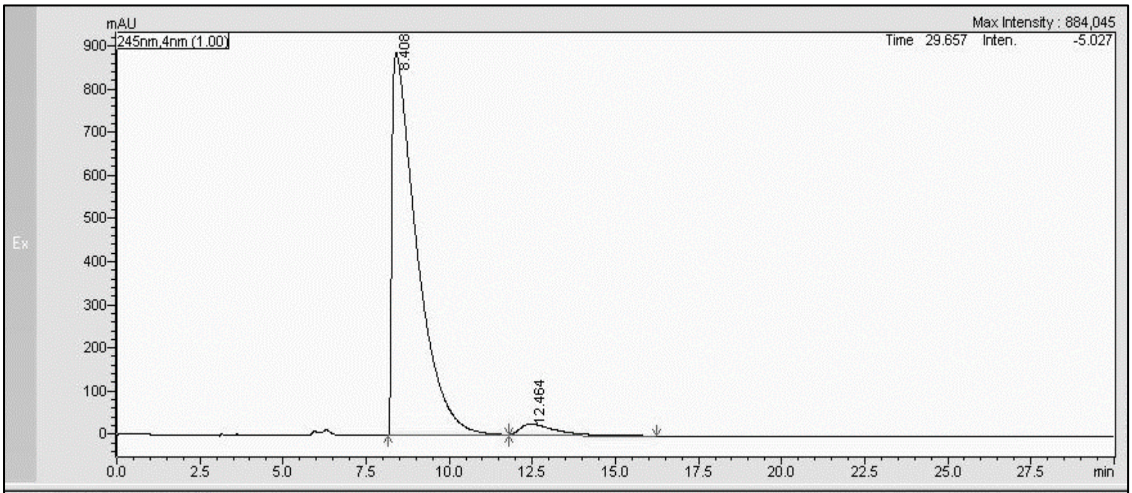

Results View - Peak Table

Peak Table | Compound | Group | Calibration Curve

Peak#	Ret. Time	Area	Height	Area%
1	8.408	46349307	886827	95.675
2	12.464	2095110	26925	4.325
Total		48444417	913752	100.000

Ethyl 4-((1*S*,2*R*)-2-((dimethylamino)methyl)cyclopropyl)benzoate (3aa)

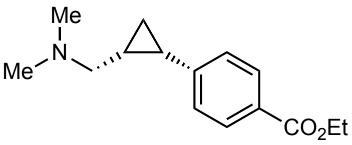

3aa

HPLC analysis: Chiralpak AD-H (hexane(0.1% DEA):2-propanol 99:1, 1.0 mL·min^{-1}, 30°C), t_R = 17.2 min (major, 99.3%), t_R = 19.7 min (minor, 0.7%)

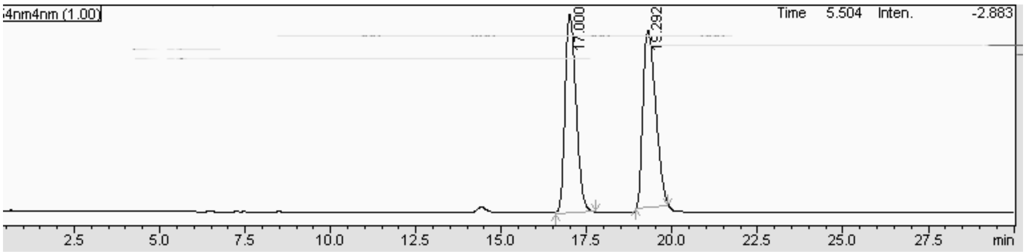

w - Peak Table

ound | Group | Calibration Curve

Ret. Time	Area	Height	Area%
17.000	18437518	823363	49.050
19.292	19151838	735332	50.950

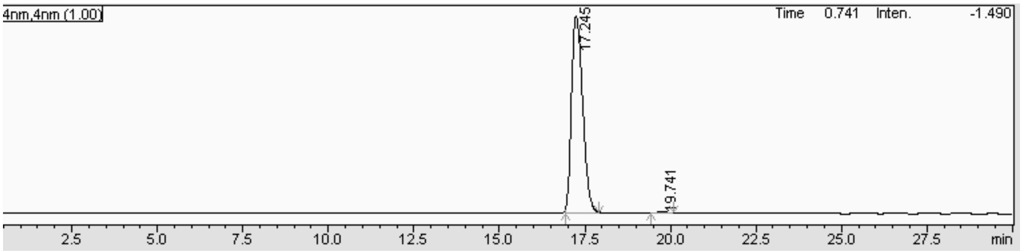

w - Peak Table

ound | Group | Calibration Curve

Ret. Time	Area	Height	Area%
17.245	21652242	946376	99.317
19.741	148946	5956	0.683

1-(4-((1*S*,2*R*)-2-((dimethylamino)methyl)cyclopropyl)phenyl)ethan-1-one (3ab)

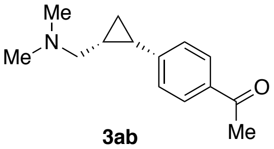

3ab

HPLC analysis: Chiralpak AD-H (hexane(0.1% DEA):2-propanol 99.5:0.5, 1.0 mL·min^{-1}, 30°C), t_R = 7.8 min (major, 99.0%), t_R = 9.0 min (minor, 1.0%)

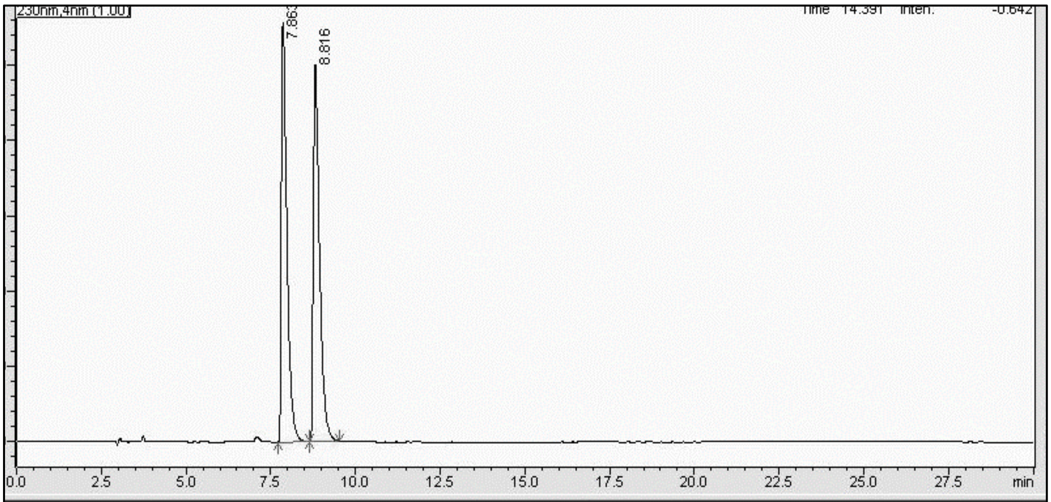

View - Peak Table

ompound | Group | Calibration Curve

Ret. Time	Area	Height	Area%
7.863	6420563	556110	50.131
8.816	6386964	499544	49.869
	12807528	1055654	100.000

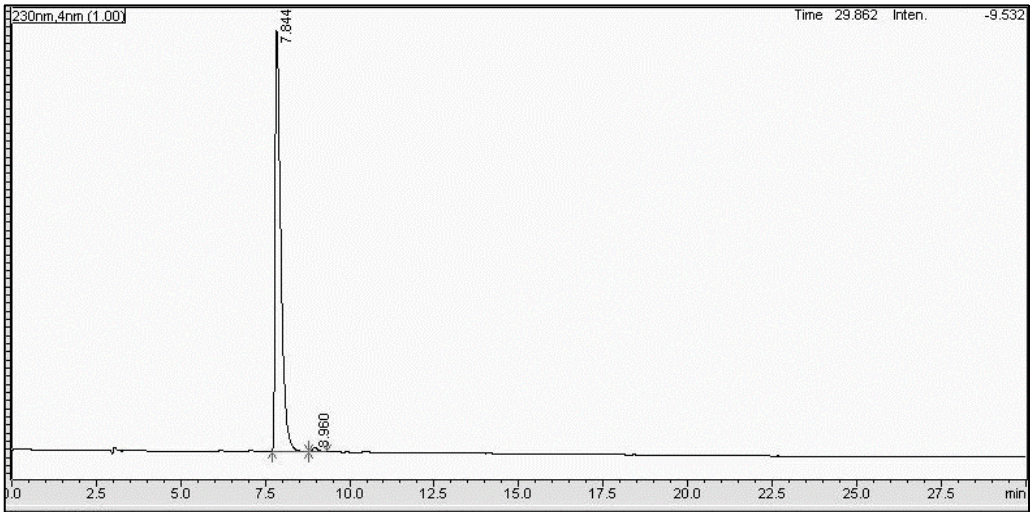

View - Peak Table

ompound | Group | Calibration Curve

Ret. Time	Area	Height	Area%
7.844	6135595	519623	98.967
8.960	64033	5282	1.033
	6199628	524905	100.000

1-((1*R*,2*S*)-2-(4-Bromophenyl)cyclopropyl)-*N*,*N*-dimethylmethanamine (3ac)

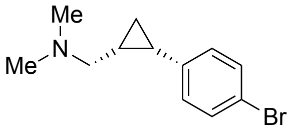

3ac

HPLC analysis: Chiralpak AD-H (hexane(0.1% DEA):2-propanol 98:2, 1.0 mL·min^{-1}, 30°C), t_R = 5.3 min (major, 99.6%), t_R = 6.1 min (minor, 0.4%)

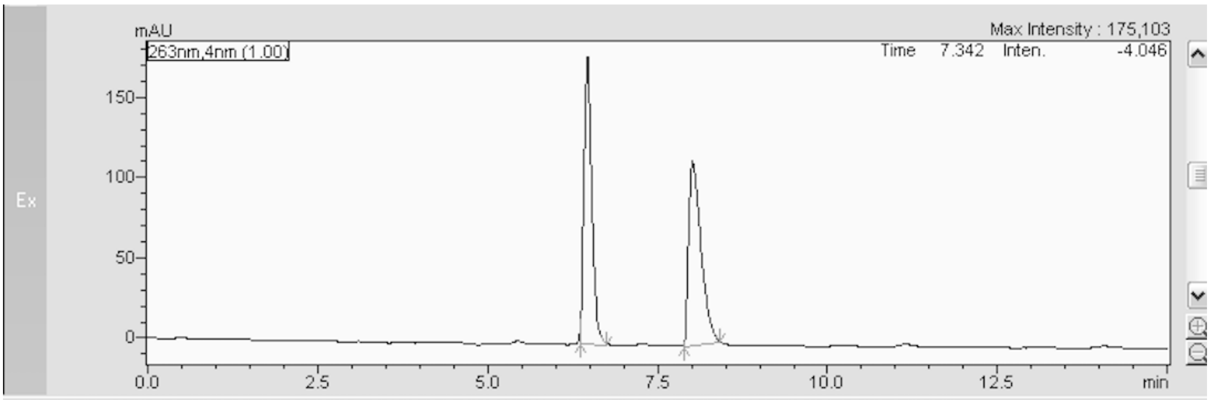

Results View - Peak Table

Peak Table | Compound | Group | Calibration Curve

Peak#	Ret. Time	Area	Height	Area%
1	6.470	1436681	178781	49.593
2	8.016	1460258	115432	50.407
Total		2896939	294213	100.000

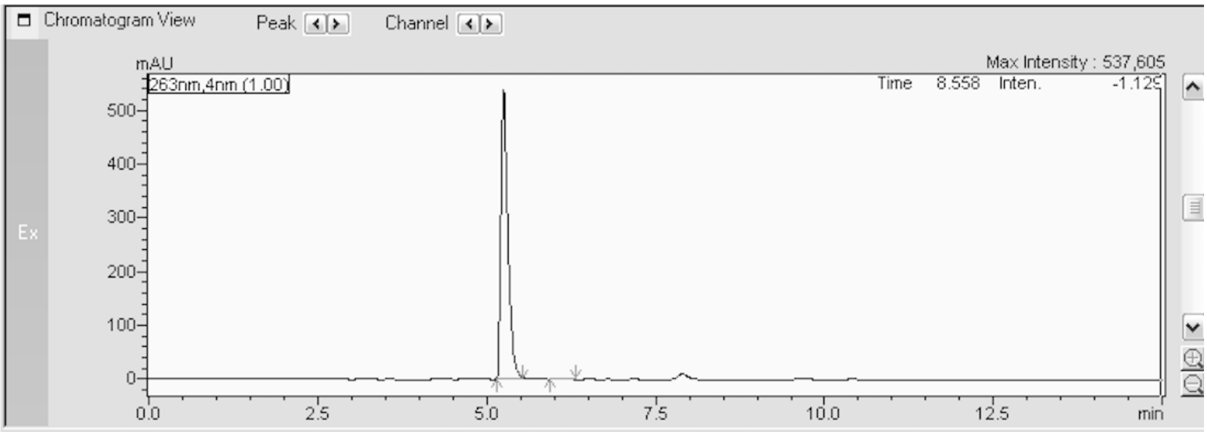

Results View - Peak Table

Peak Table | Compound | Group | Calibration Curve

Peak#	Ret. Time	Area	Height	Area%
1	5.251	3872655	537797	99.634
2	6.050	14215	2158	0.366
Total		3886871	539955	100.000

1-((1*R*,2*S*)-2-(4-Fluorophenyl)cyclopropyl)-*N*,*N*-dimethylmethanamine (3ad)

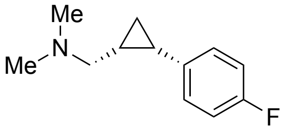

3ad

HPLC analysis: Chiralpak AD-H (hexane(0.1% DEA):2-propanol 98:2, 1.0 mL·min^{-1}, 30°C), t_R = 5.1 min (major, 99.4%), t_R = 6.8 min (minor, 0.6%)

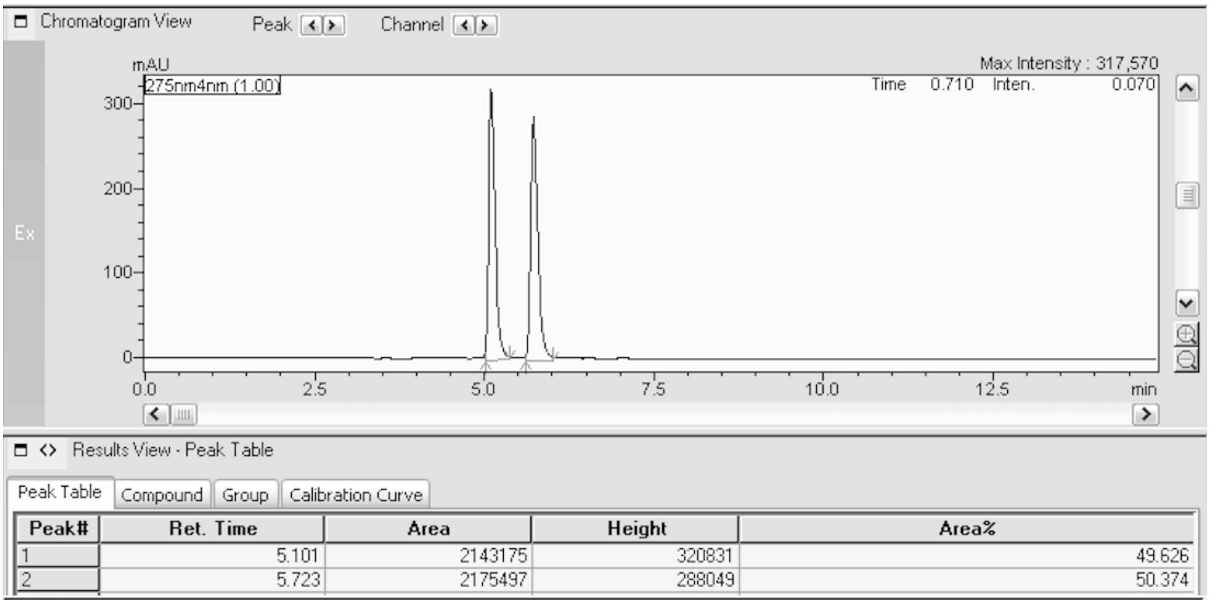

Peak#	Ret. Time	Area	Height	Area%
1	5.101	2143175	320831	49.626
2	5.723	2175497	288049	50.374

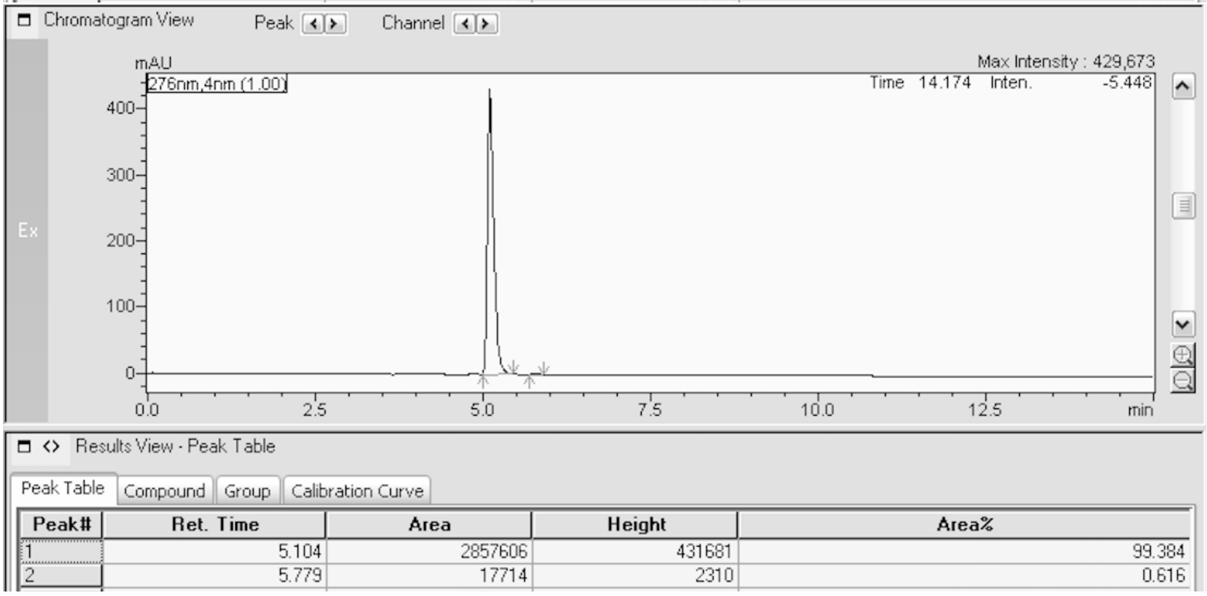

Peak#	Ret. Time	Area	Height	Area%
1	5.104	2857606	431681	99.384
2	5.779	17714	2310	0.616

***tert*-Butyl (4-((1*S*,2*R*)-2-((dimethylamino)methyl)cyclopropyl)phenyl)carbamate (3ae)**

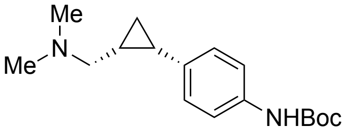

3ae

HPLC analysis: Chiralpak AD-H (hexane(0.1% DEA):2-propanol 98:2, 1.0 mL·min^{-1}, 30°C), t_R = 28.4 min (major, 99.1%), t_R = 31.2 min (minor, 0.9%)

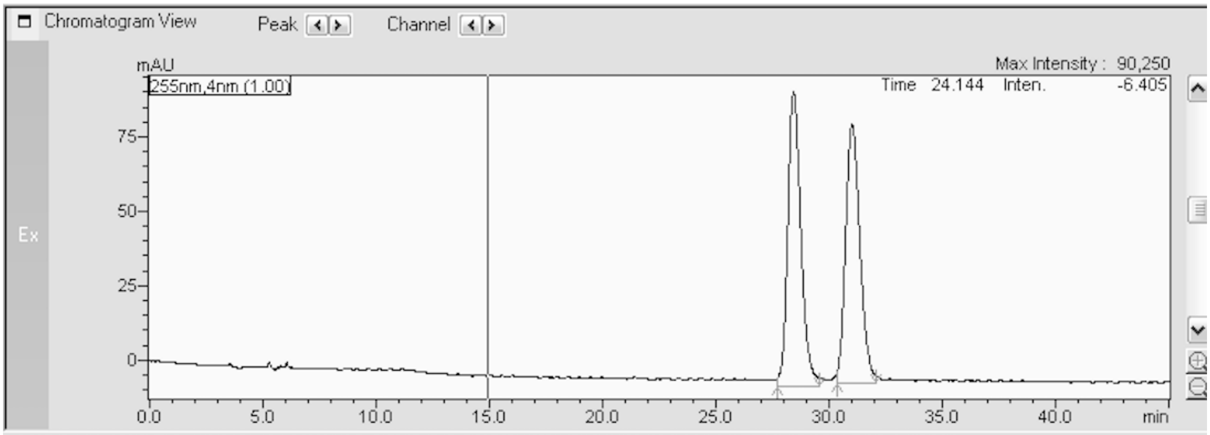

Results View - Peak Table

Peak Table | Compound | Group | Calibration Curve

Peak#	Ret. Time	Area	Height	Area%
1	28.430	3992786	98872	50.951
2	31.021	3843772	86908	49.049

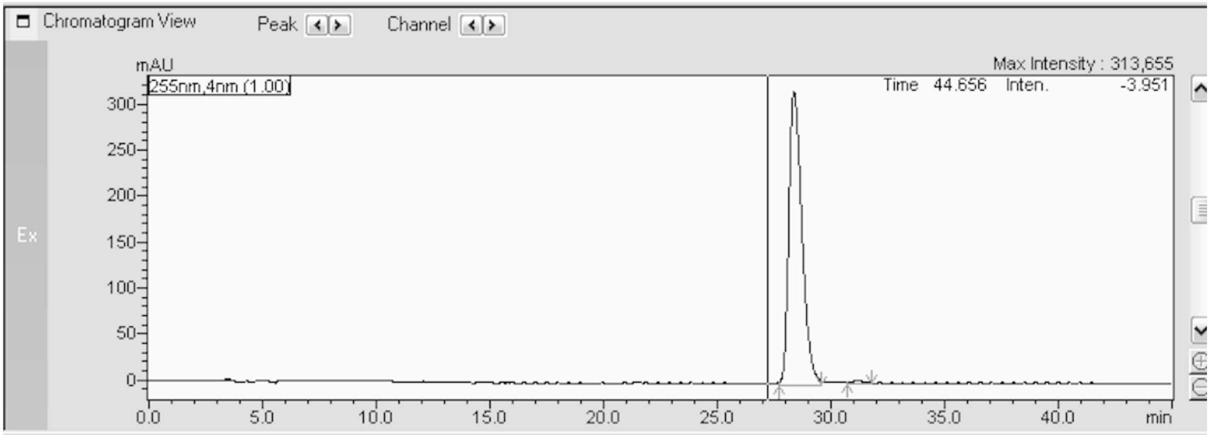

Results View - Peak Table

Peak Table | Compound | Group | Calibration Curve

Peak#	Ret. Time	Area	Height	Area%
1	28.379	12832209	319082	99.063
2	31.188	121316	2965	0.937

4-((1*S*,2*R*)-2-((dimethylamino)methyl)cyclopropyl)-*N*,*N*-diphenylaniline (3af)

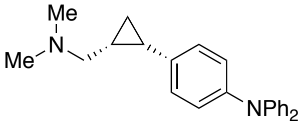

3af

HPLC analysis: Chiralpak AD-H (hexane(0.1% DEA):2-propanol 97:3, 1.0 mL·min^{-1}, 30°C), t_R = 17.1 min (major, 98.1%), t_R = 19.2 min (minor, 1.9%)

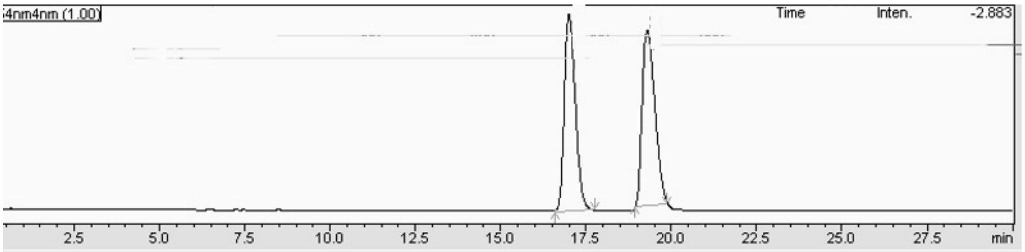

- Peak Table

ound | Group | Calibration Curve

Ret. Time	Area	Height	Area%
17.082	18437518	823363	49.050
19.283	19151838	735332	50.950

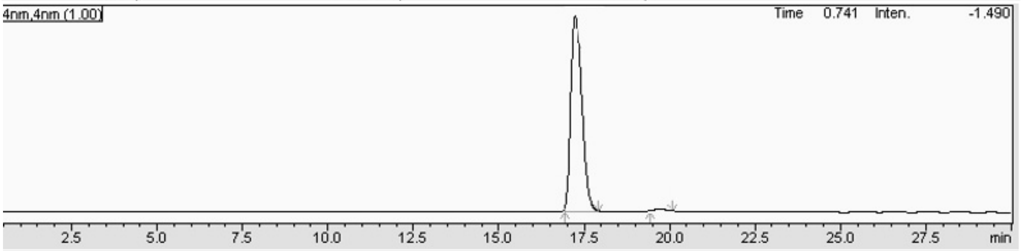

- Peak Table

ound | Group | Calibration Curve

Ret. Time	Area	Height	Area%
17.145	21652242	946376	98.117
19.221	148946	5956	1.883

1-((1*R*,2*S*)-2-(4-methoxyphenyl)cyclopropyl)-*N*,*N*-dimethylmethanamine (3ag)

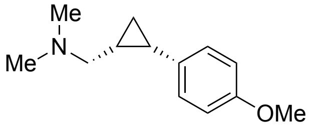

3ag

HPLC analysis: Chiralpak AD-H (hexane(0.1% DEA):2-propanol 98:2, 1.0 mL·min^{-1}, 30°C), t_R = 7.3 min (major, 98.9%), t_R = 7.9 min (minor, 1.1%)

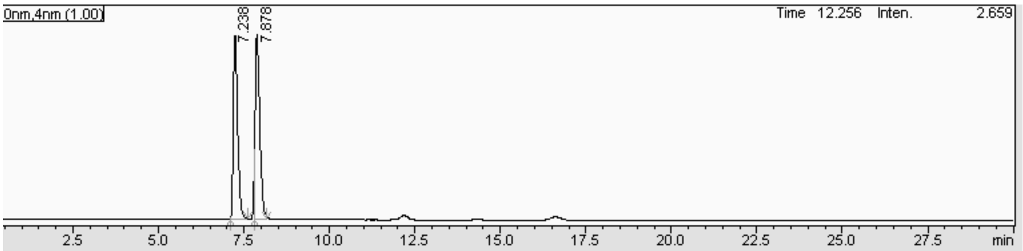

v - Peak Table

ound | Group | Calibration Curve

Ret. Time	Area	Height	Area%
7.238	2049494	233288	49.499
7.878	2091021	233578	50.501

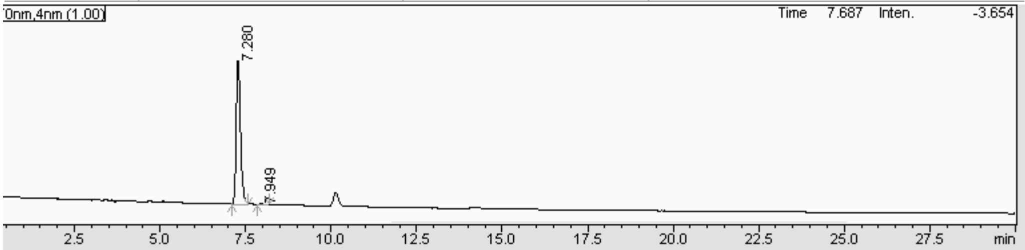

v - Peak Table

ound | Group | Calibration Curve

Ret. Time	Area	Height	Area%
7.280	625476	70107	98.907
7.949	6913	476	1.093

***N*,*N*-dimethyl-1-((1*R*,2*S*)-2-(3-nitrophenyl)cyclopropyl)methanamine (3ah)**

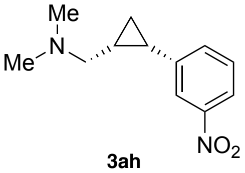

3ah

HPLC analysis: Chiralpak AD-H (hexane(0.1% DEA):2-propanol 99.6:0.4, 1.0 mL·min^{-1}, 20°C), t_R = 27.5 min (major, 98.9%), t_R = 29.1 min (minor, 1.1%)

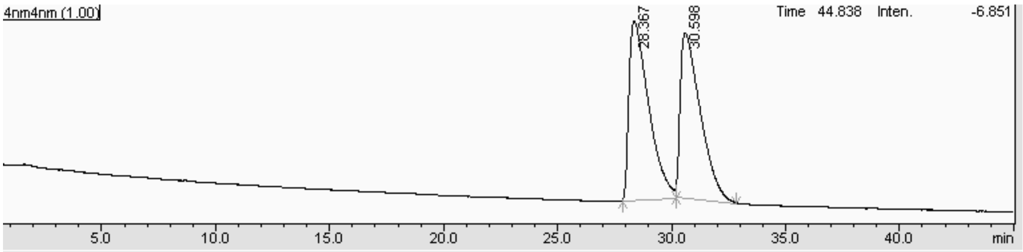

v - Peak Table

ound | Group | Calibration Curve

Ret. Time	Area	Height	Area%
28.367	1499870	24701	51.648
30.598	1404152	22781	48.352

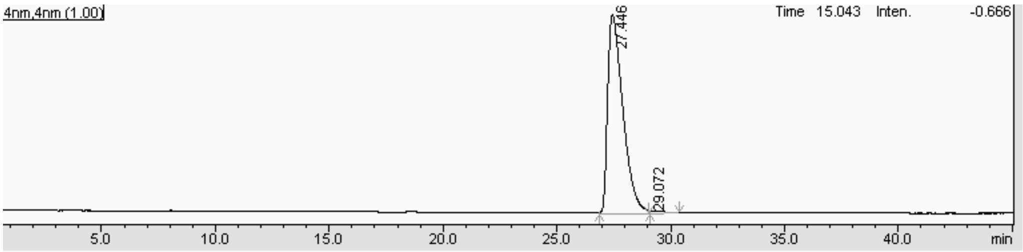

v - Peak Table

ound | Group | Calibration Curve

Ret. Time	Area	Height	Area%
27.446	3739128	80299	98.859
29.072	43164	1130	1.141

***N*,*N*-dimethyl-1-((1*S*,2*R*)-2-(3-(trifluoromethyl)phenyl)cyclopropyl)methanamine (3ai)**

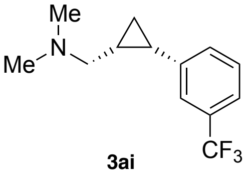

3ai

HPLC analysis: Chiralpak AD-H (hexane(0.1% DEA):2-propanol 99.6:0.4, 1.0 mL·min^{-1}, 10°C), t_R = 7.5 min (major, 99.3%), t_R = 8.4 min (minor, 0.7%)

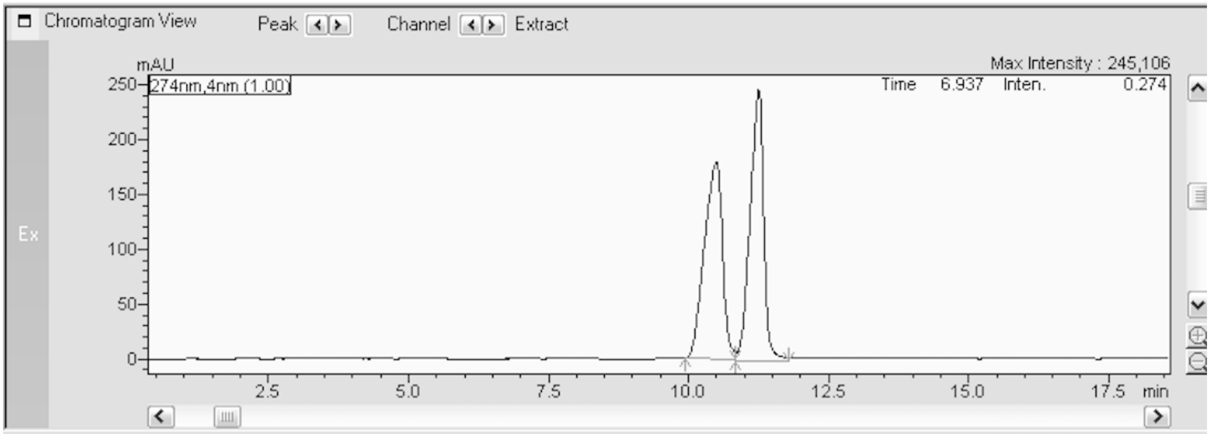

Results View - Peak Table

Peak Table | Compound | Group | Calibration Curve

Peak#	Ret. Time	Area	Height	Area%
1	10.498	4068035	179510	49.226
2	11.257	4195963	247002	50.774

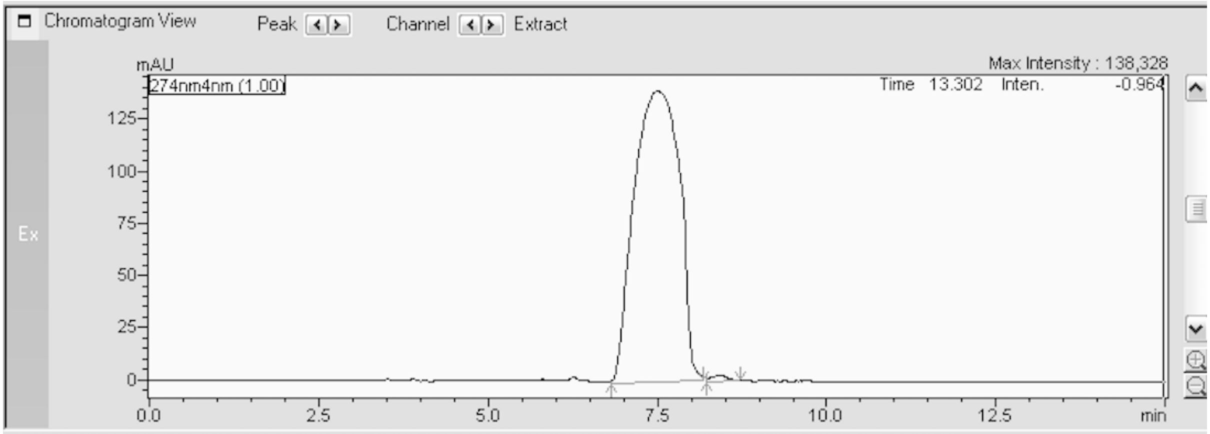

Results View - Peak Table

Peak Table | Compound | Group | Calibration Curve

Peak#	Ret. Time	Area	Height	Area%
1	7.503	6517782	139346	99.283
2	8.424	47038	2802	0.717

1-((1*R*,2*S*)-2-(3-methoxyphenyl)cyclopropyl)-*N*,*N*-dimethylmethanamine (3aj)

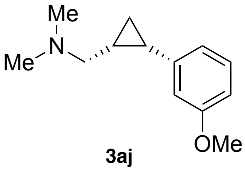

3aj

HPLC analysis: Chiralpak AD-H (hexane(0.1% DEA):2-propanol 98:2, 1.0 mL·min^{-1}, 30°C), t_R = 7.1 min (major, 98.8%), t_R = 7.6 min (minor, 1.2%)

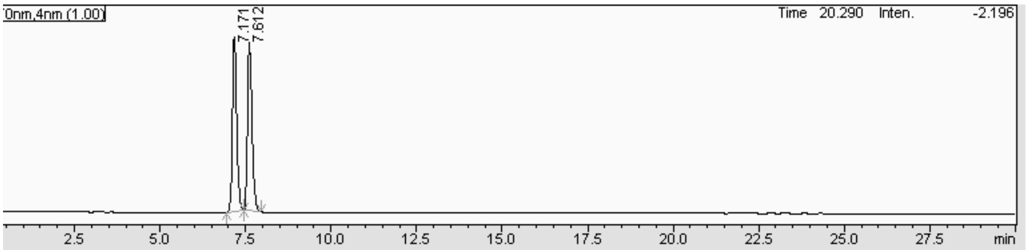

w - Peak Table

ound | Group | Calibration Curve

Ret. Time	Area	Height	Area%
7.171	1958452	223752	49.480
7.612	1999645	215961	50.520

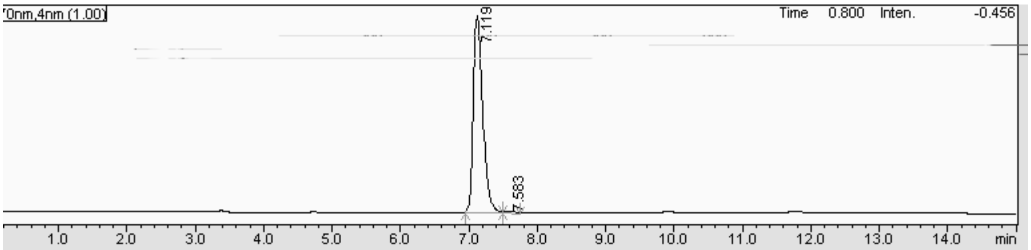

w - Peak Table

ound | Group | Calibration Curve

Ret. Time	Area	Height	Area%
7.119	5174600	531739	98.844
7.583	60527	4697	1.156

_N_,_N_-Dimethyl-1-((1_R_,2_S_)-2-(naphthalen-2-yl)cyclopropyl)methanamine (3ak)

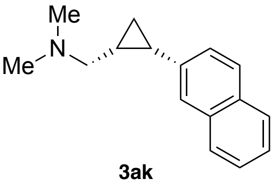

3ak

HPLC analysis: Chiralpak AD-H (hexane(0.1% DEA):2-propanol 99:1, 1.0 mL·min^{-1}, 30°C), t_R = 15.1 min (major, 98.1%), t_R = 16.8 min (minor, 1.9%)

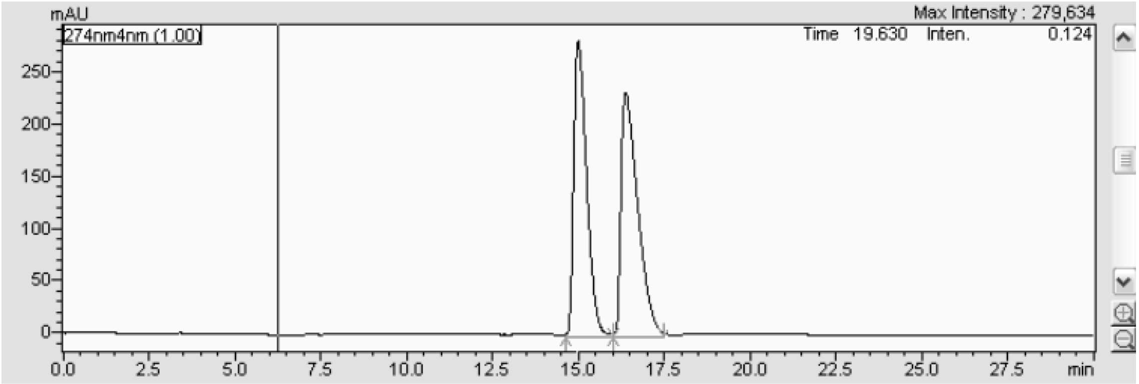

esults View - Peak Table

ble | Compound | Group | Calibration Curve

Ret. Time	Area	Height	Area%
15.023	7438992	283928	48.998
16.638	8027360	234066	51.002
	15466352	517994	100.000

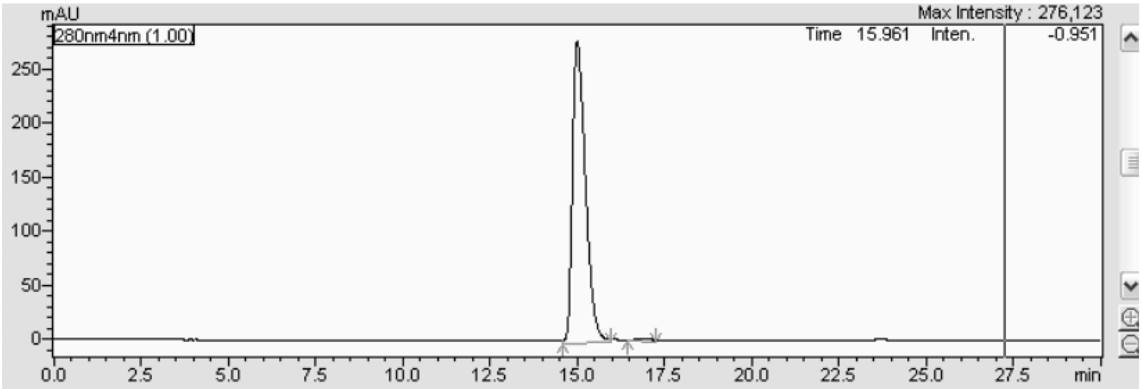

sults View - Peak Table

e | Compound | Group | Calibration Curve

Ret. Time	Area	Height	Area%
15.081	7292062	280175	98.130
16.802	85666	2373	1.870
	7377728	282547	100.000

1-((1*R*,2*S*)-2-(benzo[*d*][1,3]dioxol-5-yl)cyclopropyl)-*N*,*N*-dimethylmethanamine (3al)

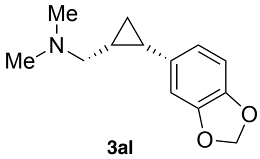

3al

HPLC analysis: Chiralpak AD-H (hexane(0.1% DEA):2-propanol 99.5:0.5, 1.0 mL·min^{-1}, 30°C), t_R = 18.8 min (major, 98.8%), t_R = 20.7 min (minor, 1.2%)

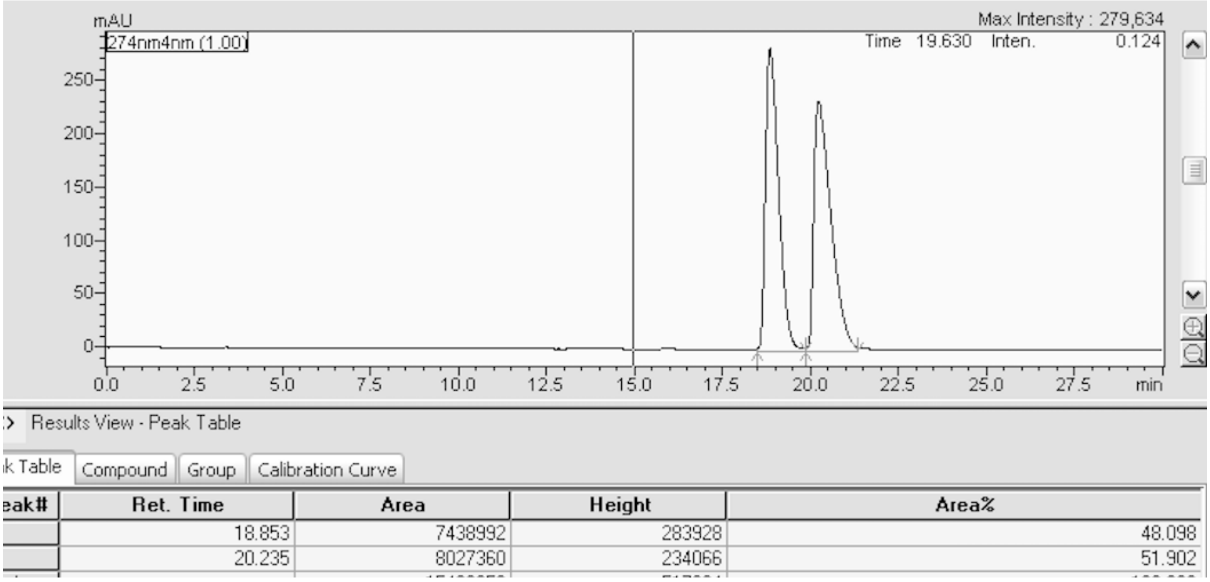

eak#	Ret. Time	Area	Height	Area%
	18.853	7438992	283928	48.098
	20.235	8027360	234066	51.902
		[illegible]	[illegible]	[illegible]

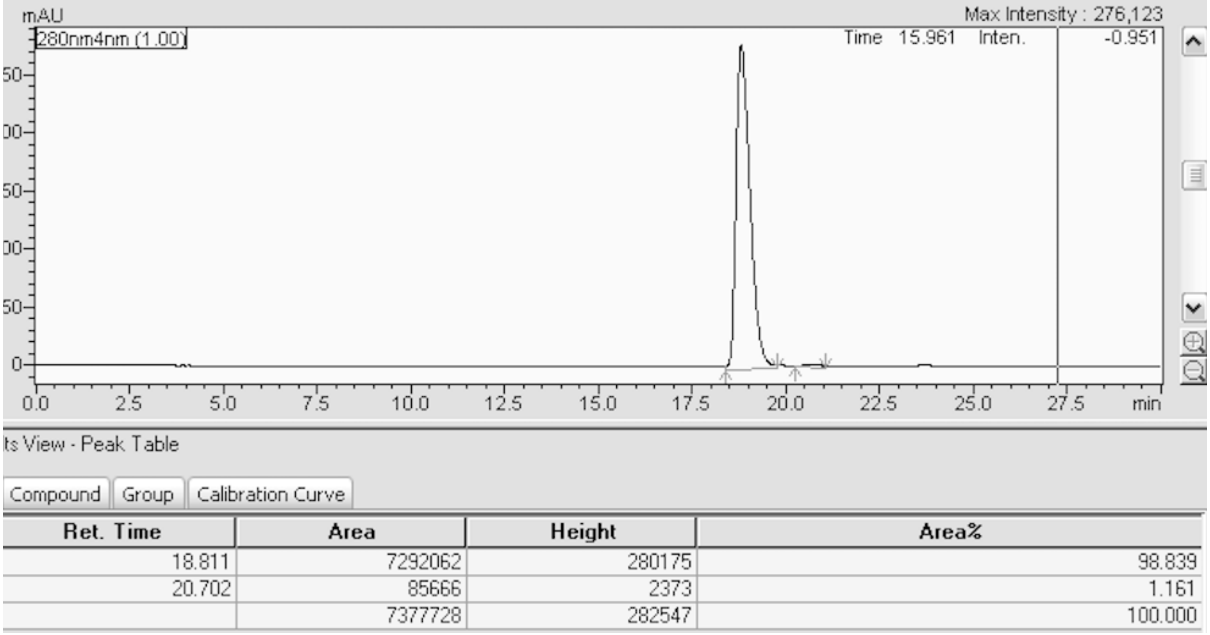

Ret. Time	Area	Height	Area%
18.811	7292062	280175	98.839
20.702	85666	2373	1.161
	7377728	282547	100.000

1-((1*R*,2*S*)-2-(6-chloropyridin-3-yl)cyclopropyl)-*N*,*N*-dimethylmethanamine (3am)

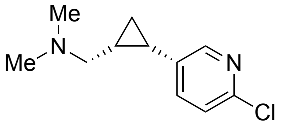

3am

HPLC analysis: Chiralpak AD-H (hexane(0.1% DEA):2-propanol 95:5, 1.0 mL·min^{-1}, 30°C), t_R = 6.8 min (major, 98.3%), t_R = 10.8 min (minor, 1.7%)

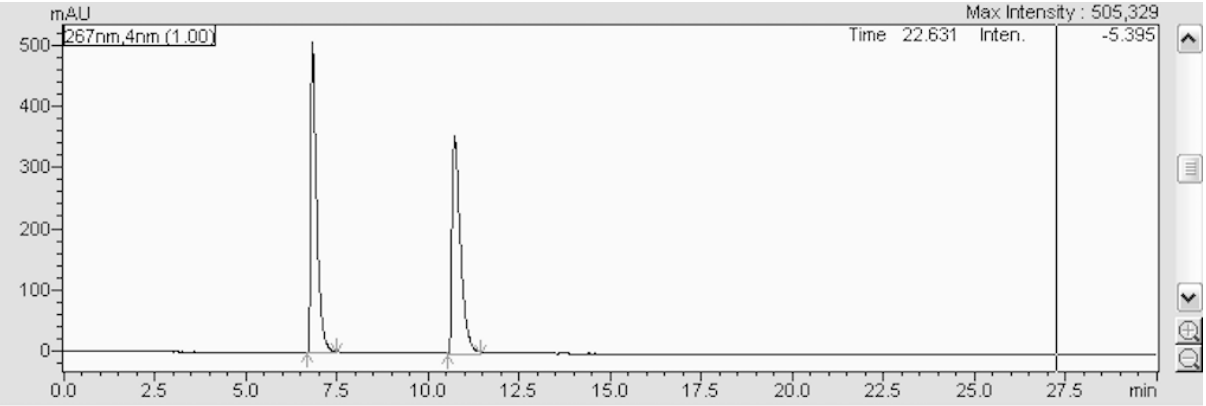

esults View - Peak Table

le | Compound | Group | Calibration Curve

Ret. Time	Area	Height	Area%
6.816	5685455	508411	49.119
10.722	5889368	359065	50.881

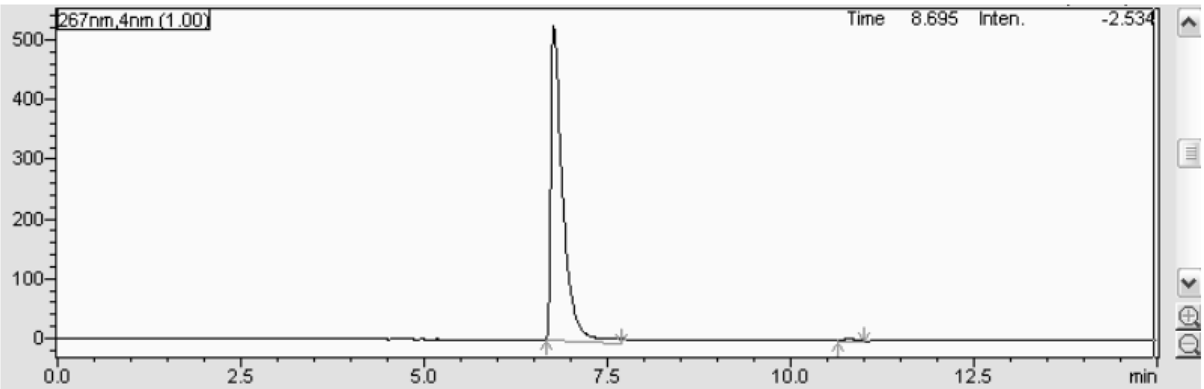

sults View - Peak Table

e | Compound | Group | Calibration Curve

Ret. Time	Area	Height	Area%
6.780	6073912	527192	98.284
10.814	106048	4355	1.716
	6179960	531547	100.000

***N*,*N*-dimethyl-1-((1*R*,2*S*)-2-(6-(trifluoromethyl)pyridin-3-yl)cyclopropyl)methanamine (3an)**

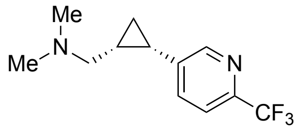

3an

HPLC analysis: Chiralpak AD-H (hexane(0.1% DEA):2-propanol 95:5, 1.0 mL·min^{-1}, 30°C), t_R = 5.3 min (major, 99.3%), t_R = 6.1 min (minor, 0.7%)

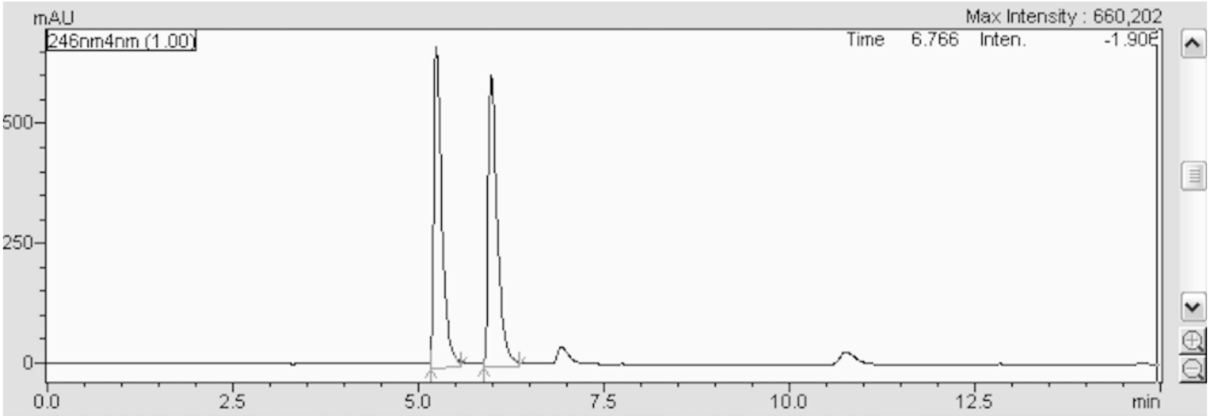

ults View - Peak Table

Compound | Group | Calibration Curve

Ret. Time	Area	Height	Area%
5.244	5259021	671026	49.849
5.985	5290973	607103	50.151

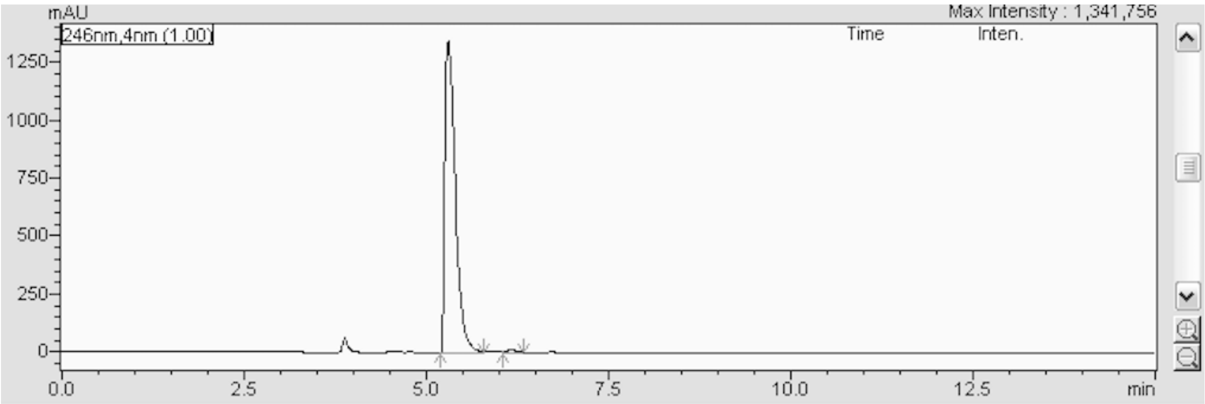

esults View - Peak Table

le | Compound | Group | Calibration Curve

Ret. Time	Area	Height	Area%
5.300	13693053	1346824	99.326
6.171	92903	11758	0.674
	[illegible]	[illegible]	[illegible]

1-((1*R*,2*S*)-2-(2-Fluoropyridin-4-yl)cyclopropyl)-*N*,*N*-dimethylmethanamine (3ao)

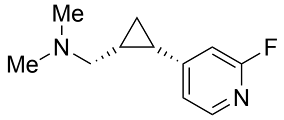

3ao

HPLC analysis: Chiralpak AD-H (hexane(0.1% DEA):2-propanol 95:5, 1.0 mL·min^{-1}, 30°C), t_R = 7.6 min (major, 99.7%), t_R = 8.0 min (minor, 0.3%)

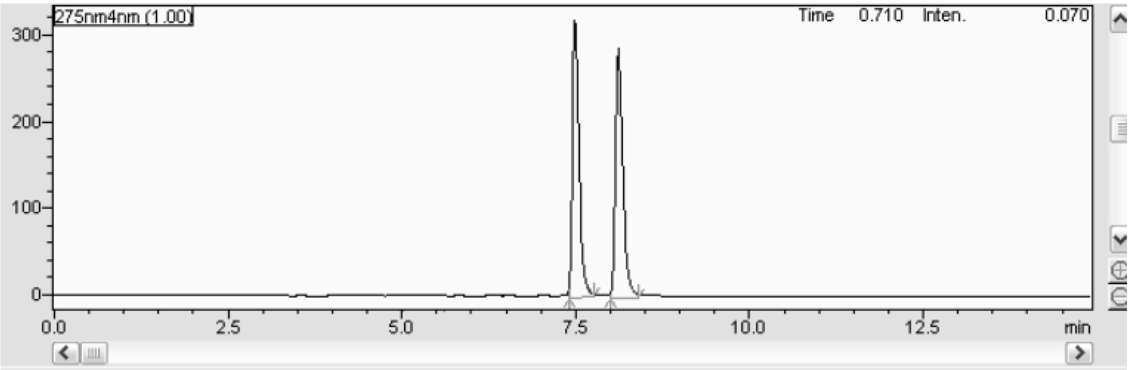

sults View - Peak Table

e | Compound | Group | Calibration Curve

Ret. Time	Area	Height	Area%
7.507	2143175	320831	49.826
7.820	2165497	288049	50.174
	4318672	608880	100.000

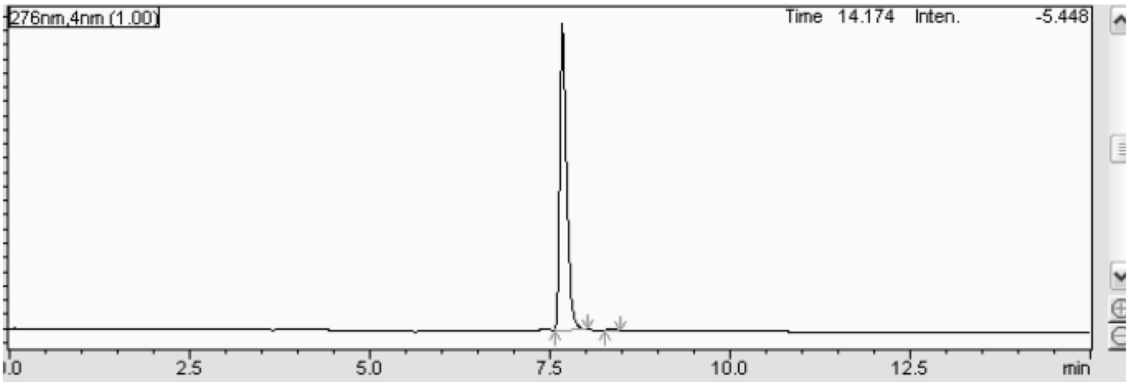

View - Peak Table

mpound | Group | Calibration Curve

Ret. Time	Area	Height	Area%
7.594	2857606	431681	99.684
7.989	17714	2310	0.316
	2875320	433991	100.000

_N_,_N_-Dimethyl-1-((1_S_,2_S_)-2-phenylcyclopropyl)methanamine (5)

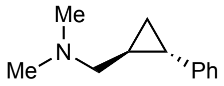

5

HPLC analysis: Chiralpak AD-H (hexane(0.1% DEA):2-propanol 98:2, 1.0 mL·min^{-1}, 30 °C) t_R = 5.4 min (major, 96.4%), t_R = 5.8 min (minor, 3.6%)

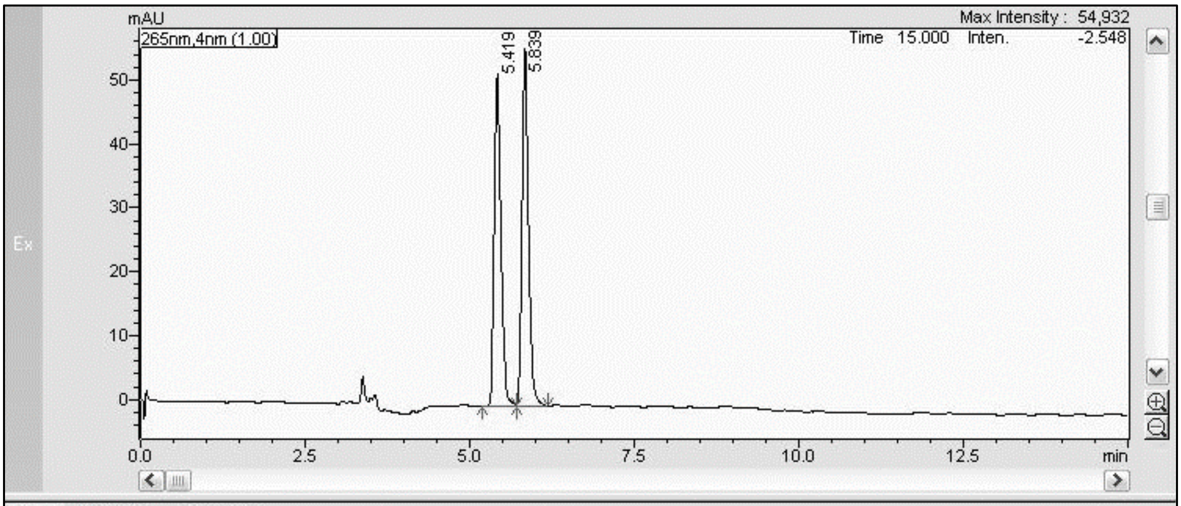

Results View - Peak Table

Peak Table | Compound | Group | Calibration Curve

Peak#	Ret. Time	Area	Height	Area%
1	5.419	369906	51884	49.402
2	5.839	378859	55946	50.598
Total		748765	107830	100.000

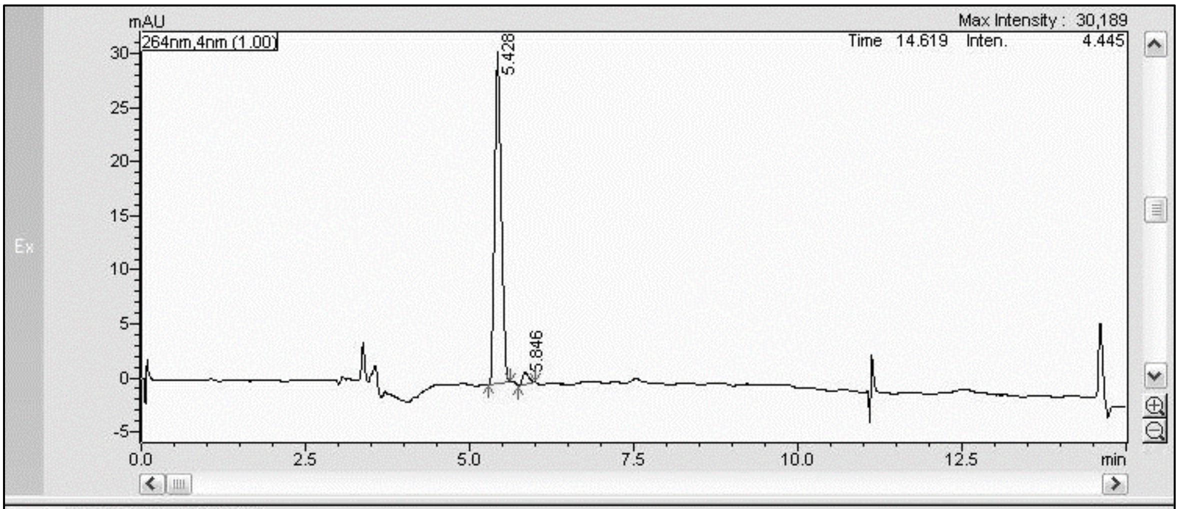

Results View - Peak Table

Peak Table | Compound | Group | Calibration Curve

Peak#	Ret. Time	Area	Height	Area%
1	5.428	214369	30739	96.376
2	5.846	8062	1194	3.624
Total		222431	31933	100.000

1-((1*R*,2*S*,3*S*)-2-(4-Methoxyphenyl)-3-phenylcyclopropyl)-*N*,*N*-dimethylmethanamine (6)

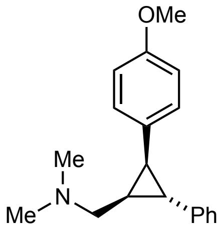

6

HPLC analysis: Chiralpak AD-H (hexane(0.1% DEA):2-propanol 98:2, 1.0 mL·min^{-1}, 30 °C) t_R = 11.6 min (minor, 2.1%), t_R = 14.0 min (major, 97.9%)

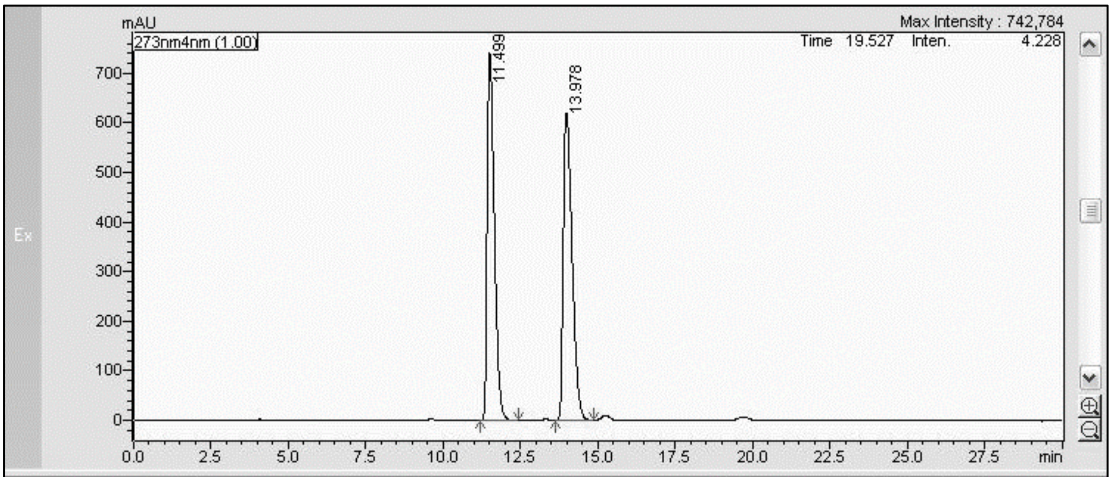

Results View - Peak Table

Peak Table | Compound | Group | Calibration Curve

Peak#	Ret. Time	Area	Height	Area%
1	11.499	12081159	743327	49.607
2	13.978	12272460	620844	50.393
Total		24353619	1364171	100.000

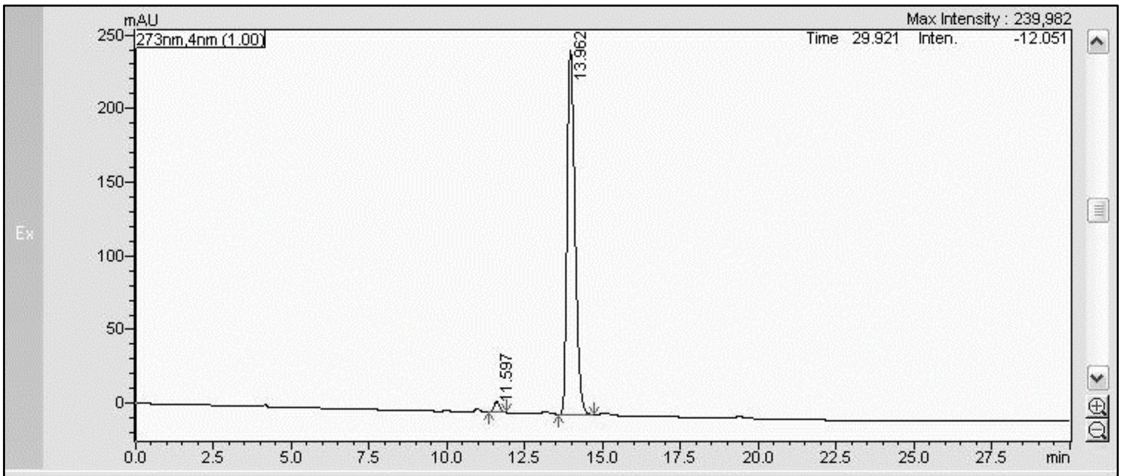

Results View - Peak Table

Peak Table | Compound | Group | Calibration Curve

Peak#	Ret. Time	Area	Height	Area%
1	11.597	91657	7066	2.070
2	13.962	4335920	247627	97.930
Total		4427577	254693	100.000

1-((1*S*,2*R*)-2-(4-Methoxyphenyl)-2-phenylcyclopropyl)-*N*,*N*-dimethylmethanamine (7)

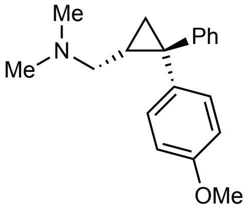

7

HPLC analysis: Chiralpak AD-H (hexane(0.1% DEA):2-propanol 98:2, 1.0 mL·min^{-1}, 30 °C) t_R = 8.3 min (minor, 0.5%), t_R = 12.2 min (major, 99.5%)

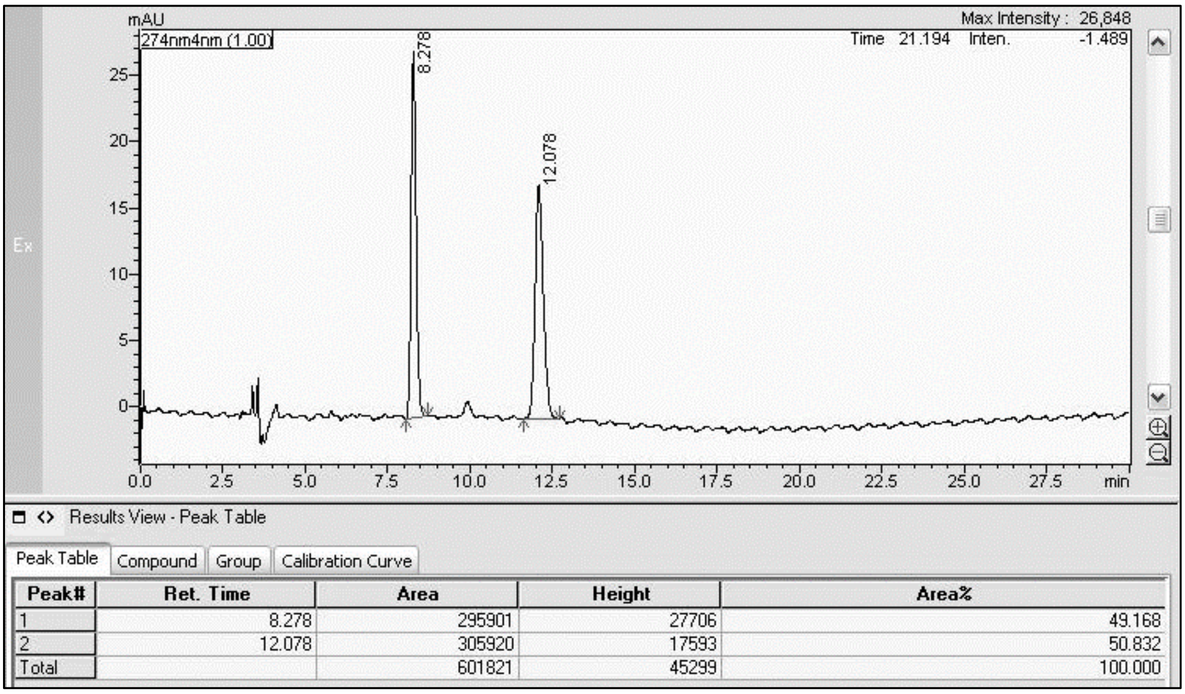

Peak#	Ret. Time	Area	Height	Area%
1	8.278	295901	27706	49.168
2	12.078	305920	17593	50.832
Total		601821	45299	100.000

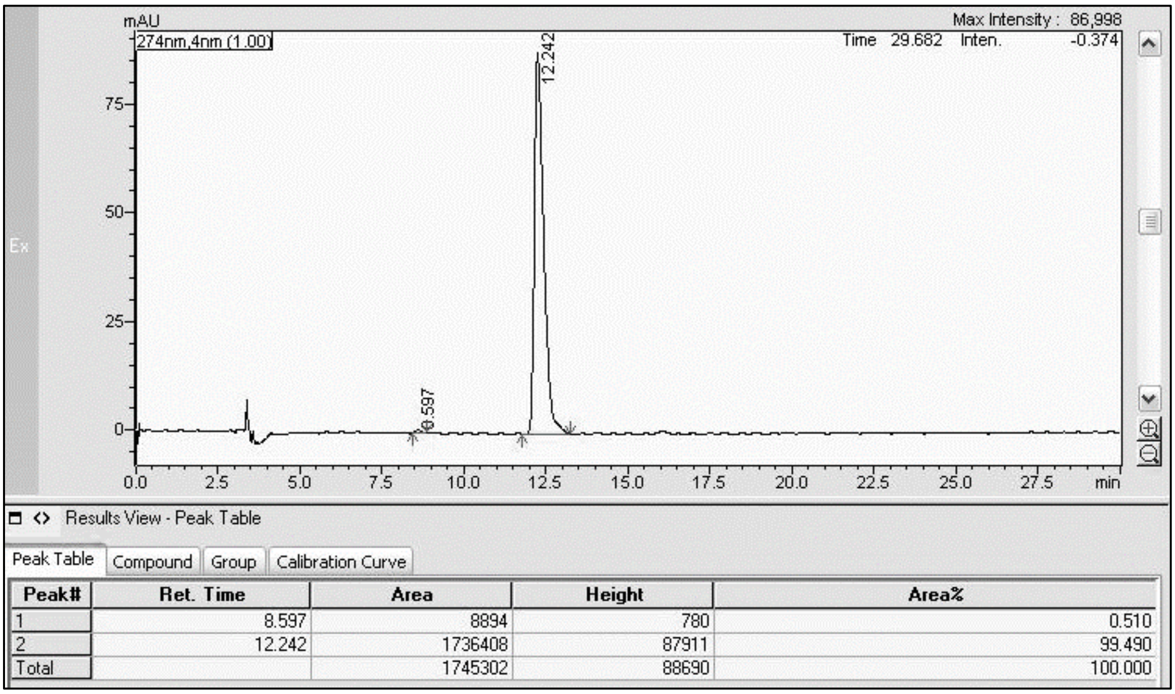

Peak#	Ret. Time	Area	Height	Area%
1	8.597	8894	780	0.510
2	12.242	1736408	87911	99.490
Total		1745302	88690	100.000

***N*,*N*-*D*imethyl-1-((1*R*,2*S*)-2-phenylcyclobutyl)methanamine (9a)**

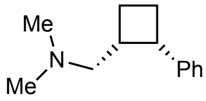

9a

^{1}H-NMR analysis:

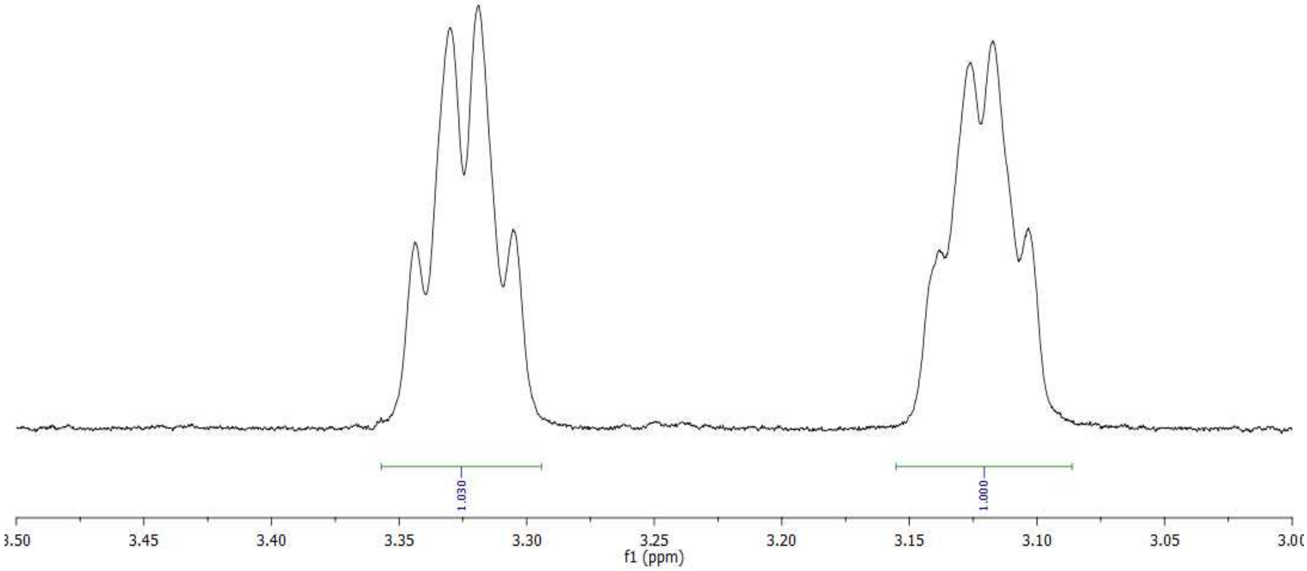

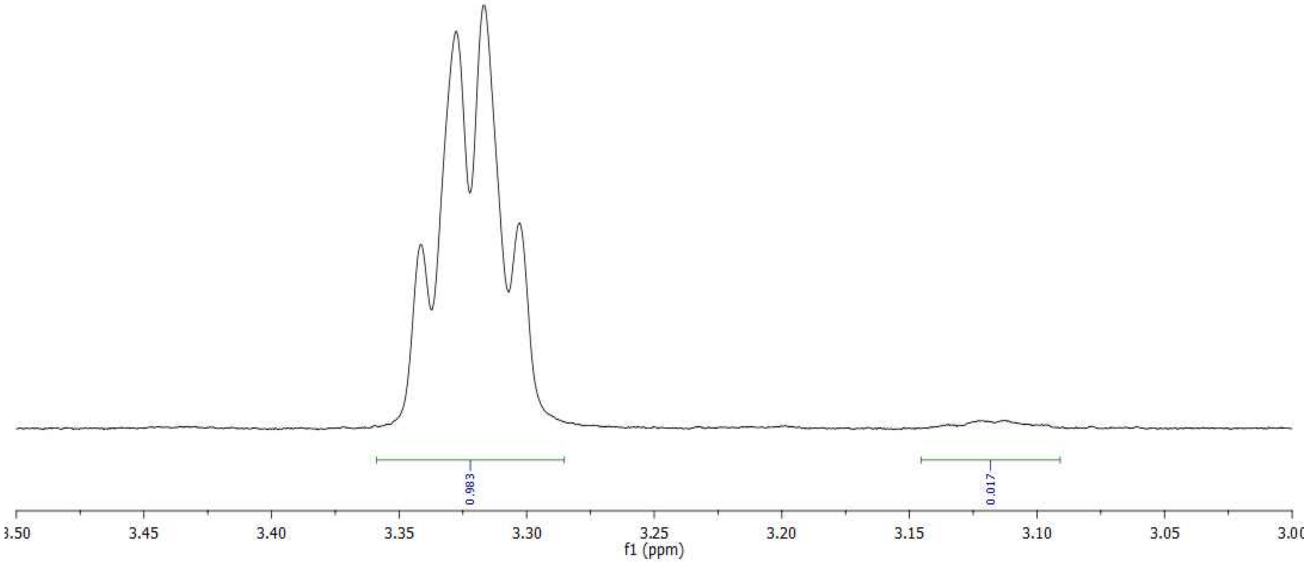

Ethyl 3-(methyl(((1*R*,2*S*)-2-phenylcyclobutyl)methyl)amino)propanoate (9b)

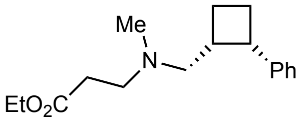

9b

^{1}H-NMR analysis:

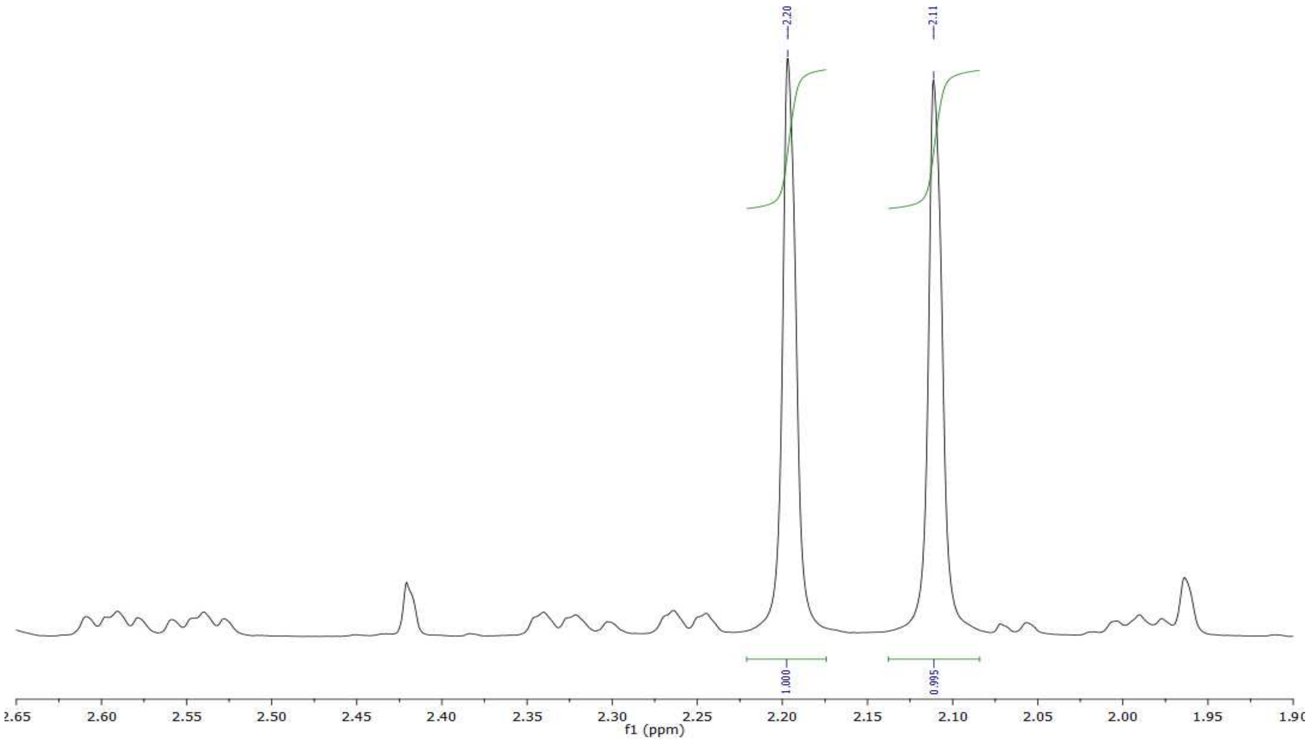

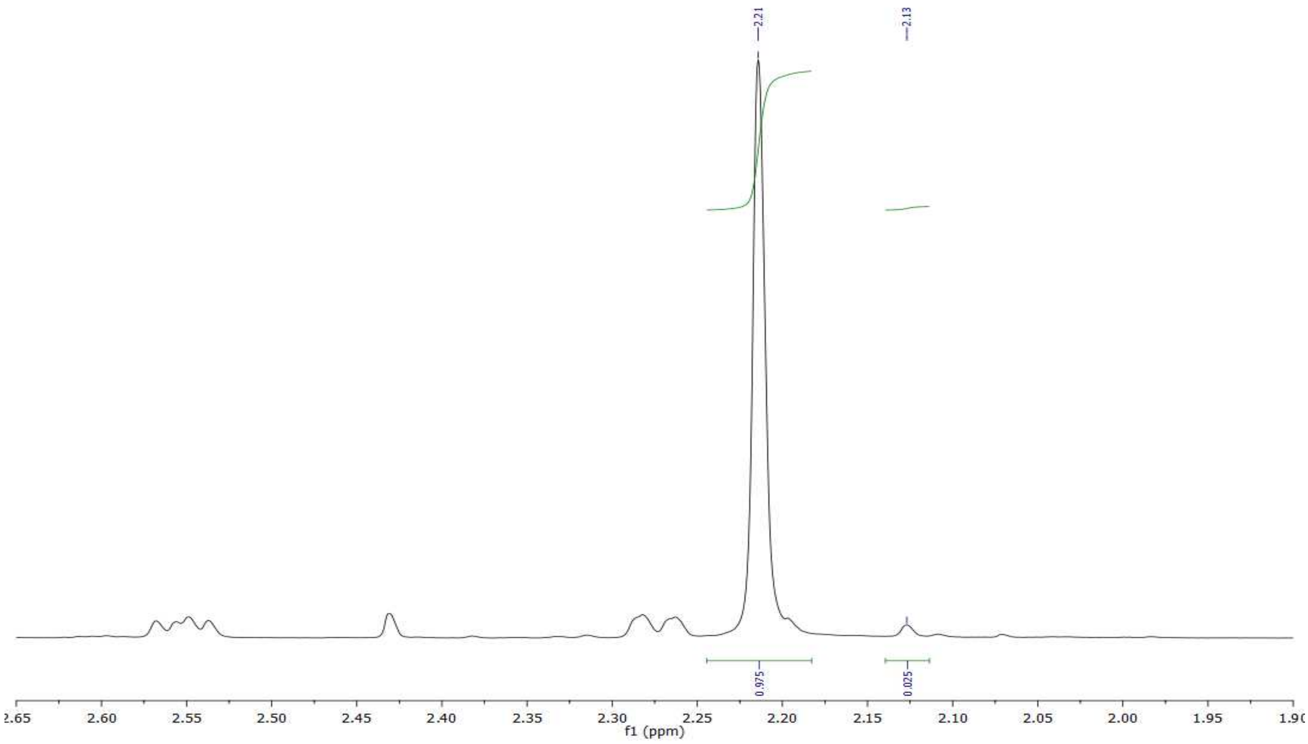

N-Methyl-3-(5-methylfuran-2-yl)-N-(((1R,2S)-2-phenylcyclobutyl)methyl)propan-1-amine (9c)

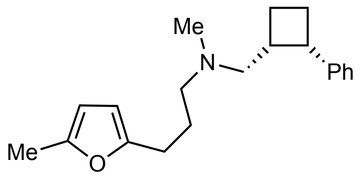

9c

HPLC analysis: Chiralpak AD-H (hexane(0.1% DEA):2-propanol 99.6:0.4, 1.0 mL·min^{-1}, 15 °C) t_R = 4.8 min (major, 97.0%), t_R = 5.1 min (minor, 3.0%)

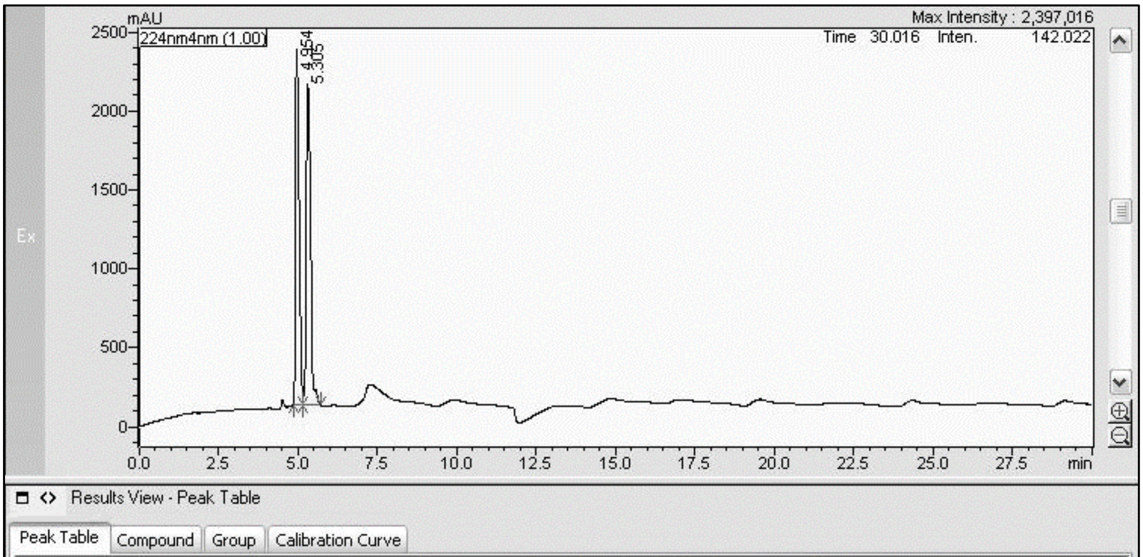

Peak#	Ret. Time	Area	Height	Area%
1	4.954	18434716	2261315	49.074
2	5.305	19130780	2033664	50.926
Total		37565496	4294979	100.000

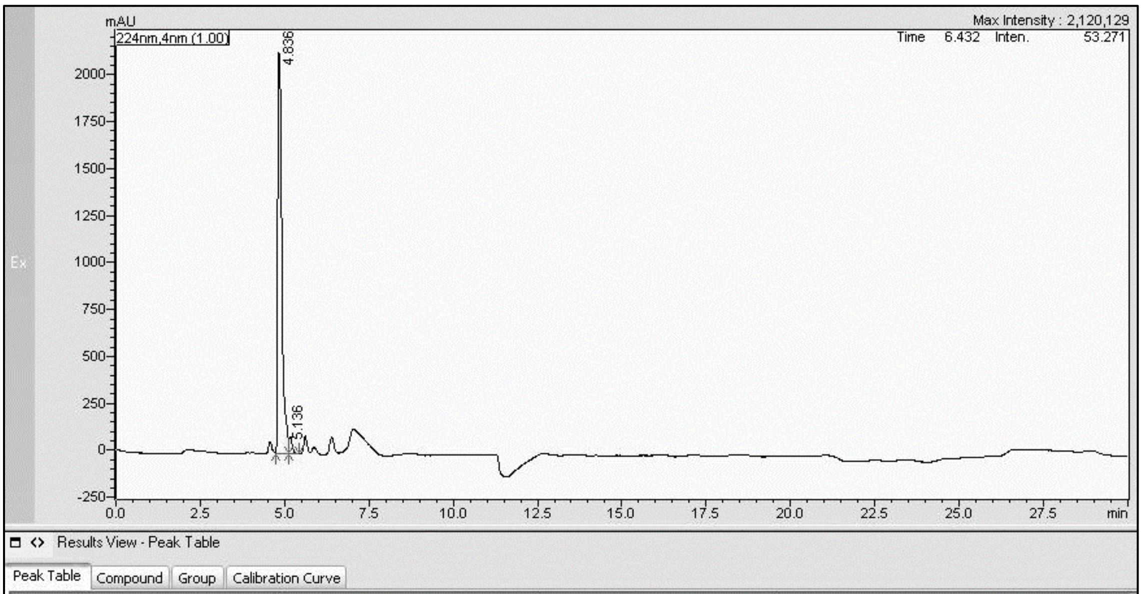

Peak#	Ret. Time	Area	Height	Area%
1	4.836	18618715	2135269	96.954
2	5.136	584913	73976	3.046
Total		19203628	2209246	100.000

3-((*tert*-Butyldimethylsilyl)oxy)-*N*-methyl-*N*-(((1*R*,2*S*)-2-phenylcyclobutyl)methyl)propan-1-amine (9d)

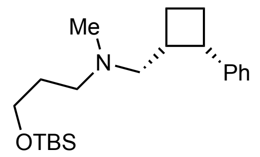

9d

^{1}H-NMR analysis:

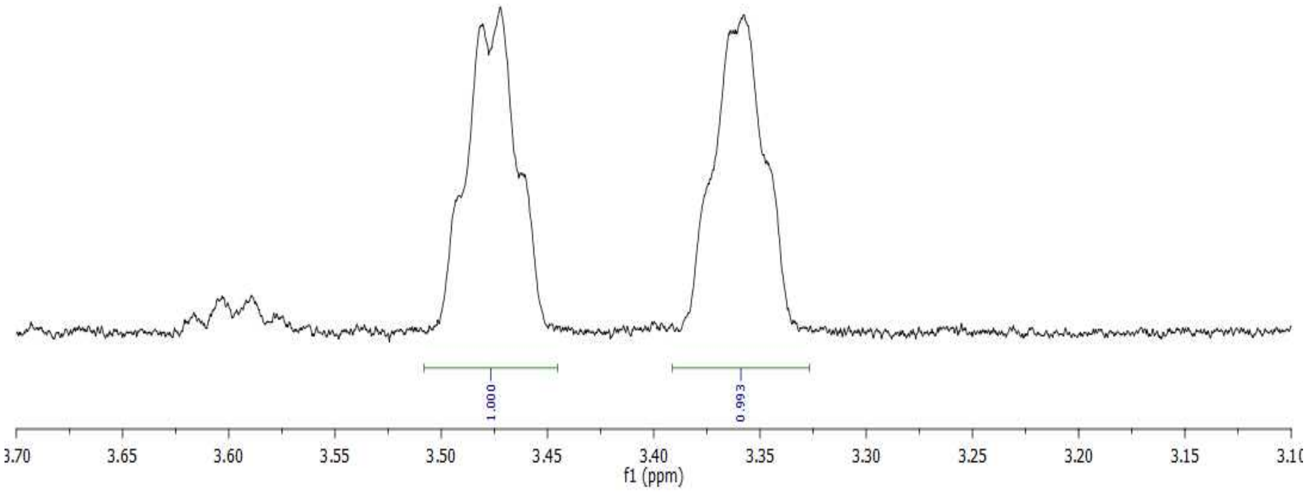

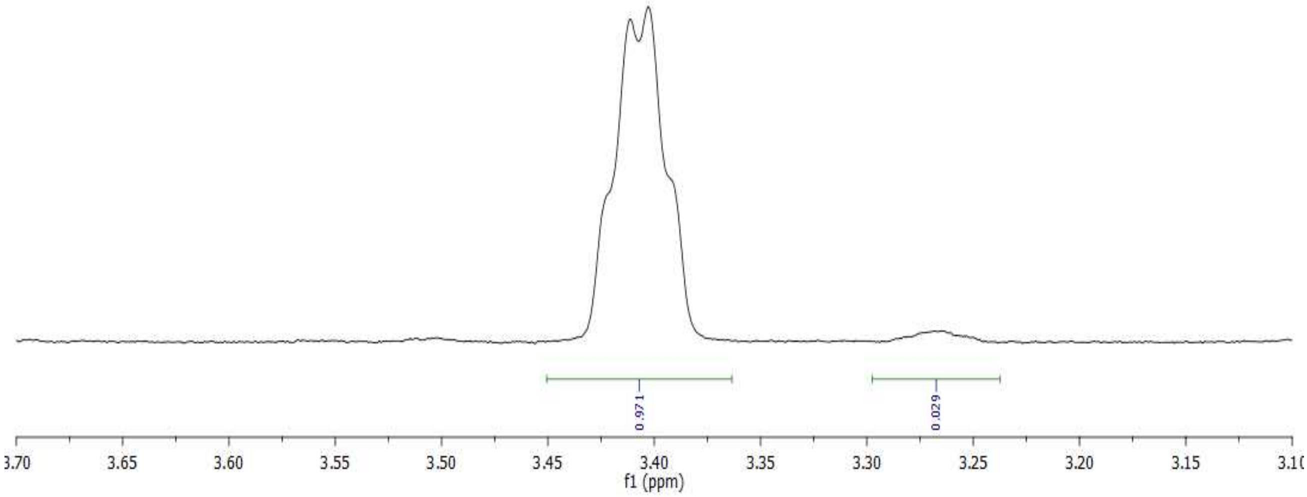

***tert*-Butyl methyl(2-(methyl(((1*R*,2*S*)-2-phenylcyclobutyl)methyl)amino)ethyl)carbamate (9e)**

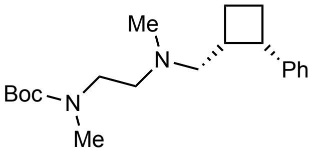

9e

^{1}H-NMR analysis:

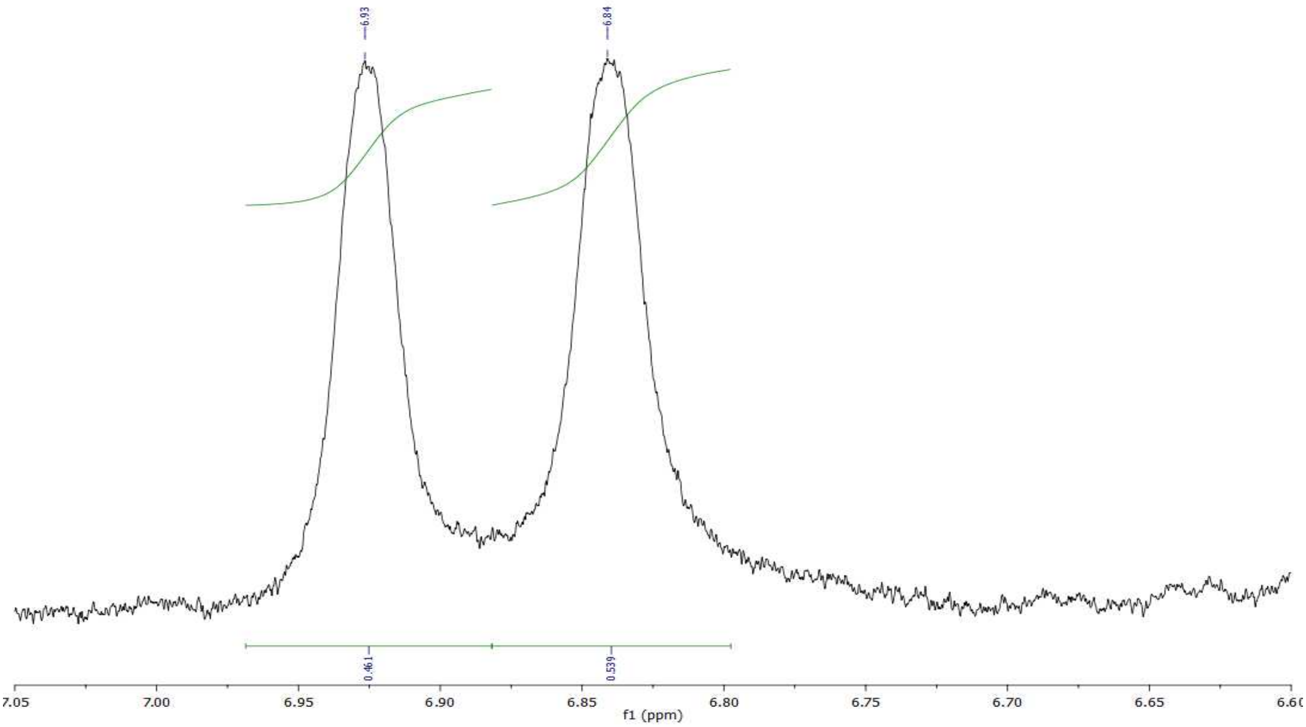

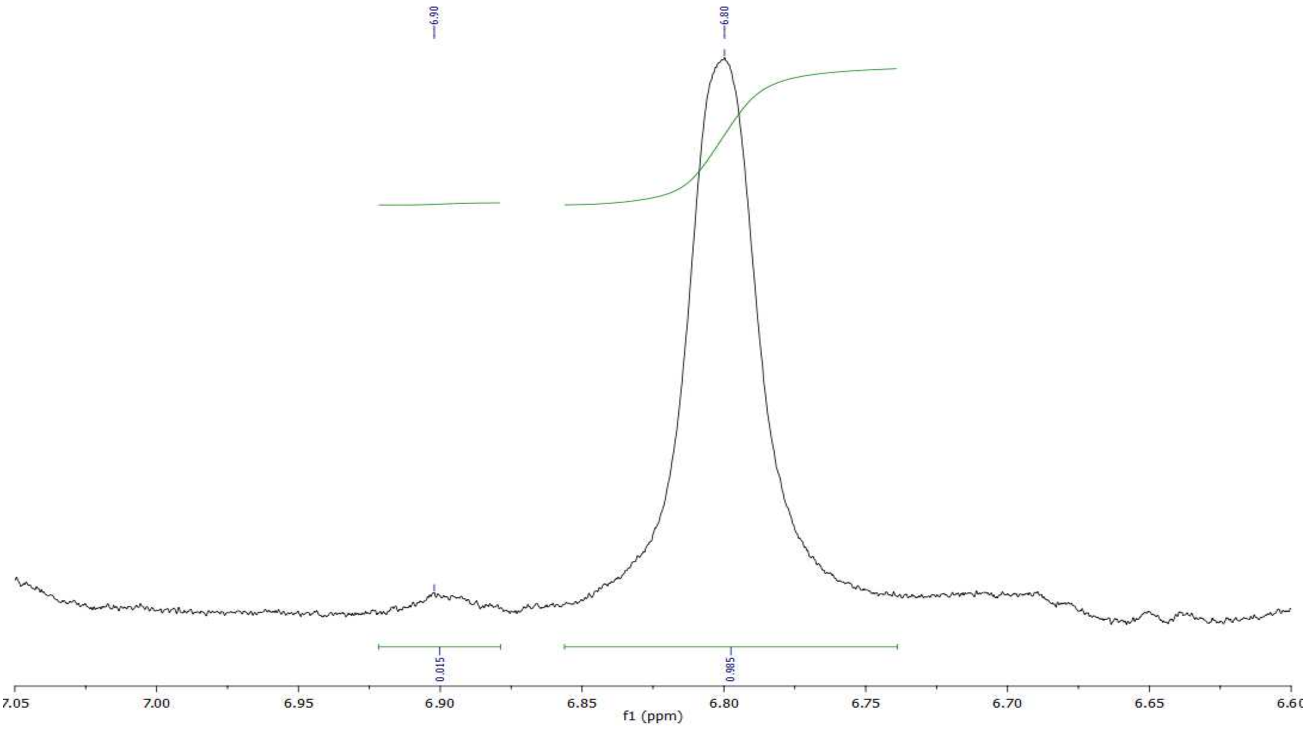

1-(((1*R*,2*S*)-2-Phenylcyclobutyl)methyl)piperidine (9f)

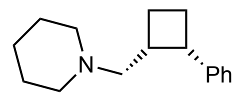

9f

^{1}H-NMR analysis:

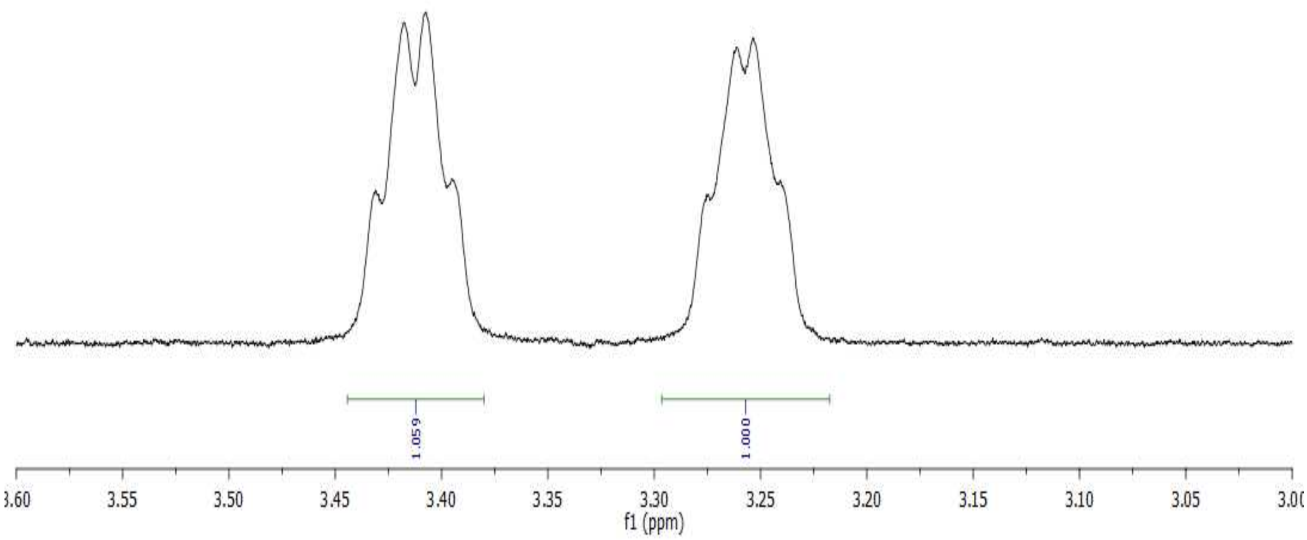

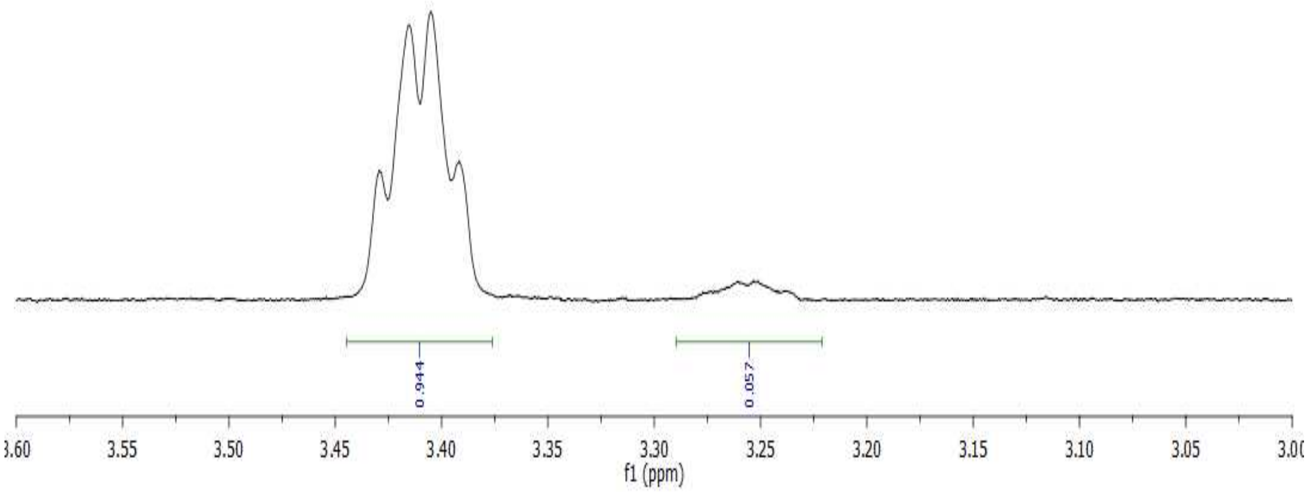

8-(((1*R*,2*S*)-2-Phenylcyclobutyl)methyl)-1,4-dioxa-8-azaspiro[4.5]decane (9g)

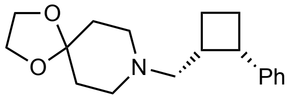

9g

HPLC analysis: Chiralpak AD-H (hexane(0.1% DEA):2-propanol 97:3, 1.0 mL·min^{-1}, 30 °C) t_R = 5.7 min (major, 95.0%), t_R = 6.0 min (minor, 5.0%)

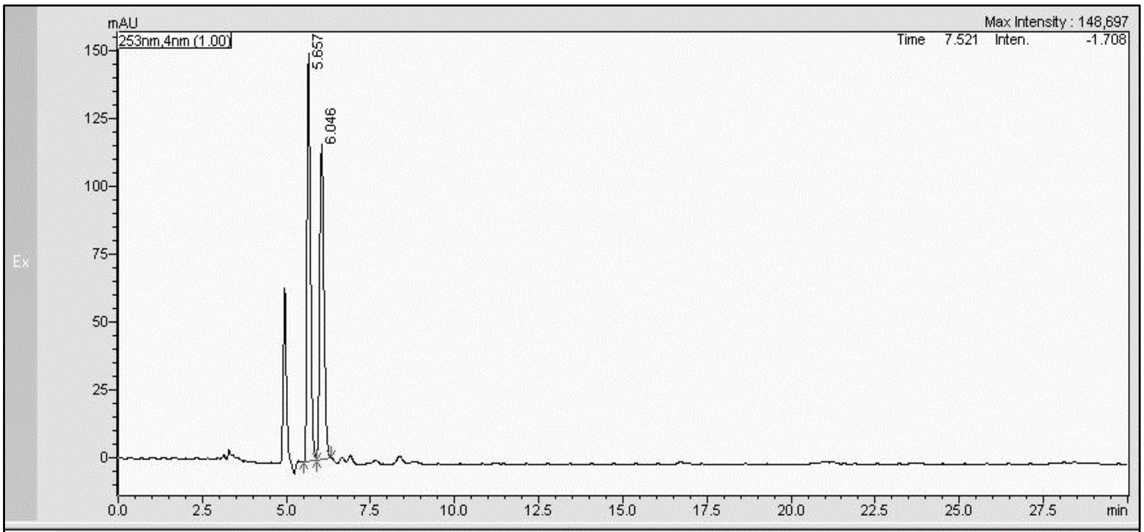

Results View - Peak Table

Peak Table | Compound | Group | Calibration Curve

Peak#	Ret. Time	Area	Height	Area%
1	5.657	1045907	149917	50.504
2	6.046	1025035	115967	49.496
Total		2070942	265884	100.000

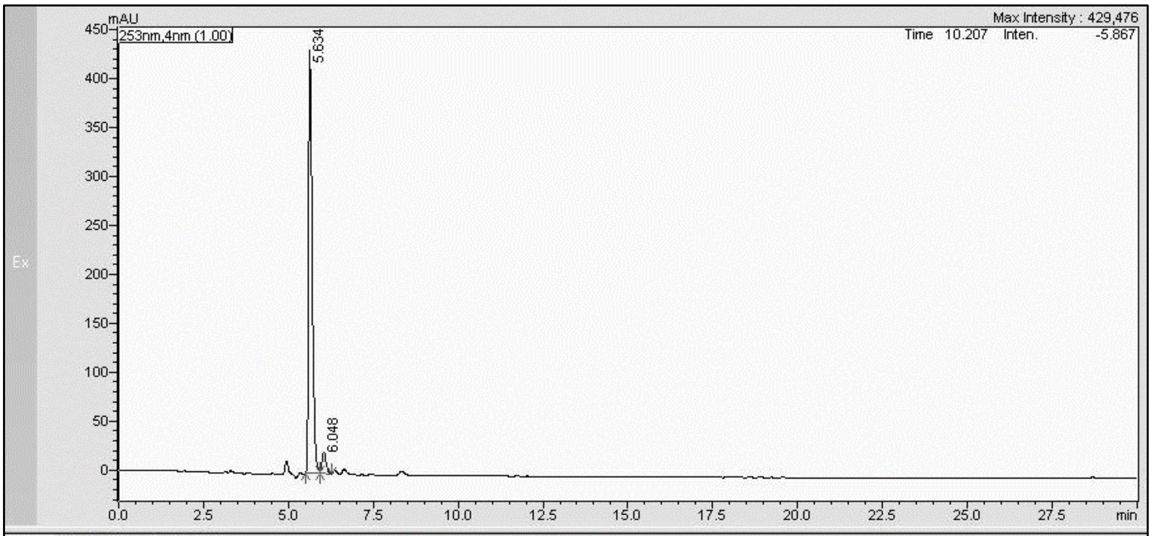

Results View - Peak Table

Peak Table | Compound | Group | Calibration Curve

Peak#	Ret. Time	Area	Height	Area%
1	5.634	3242325	431925	94.950
2	6.048	172445	21275	5.050
Total		3414769	453200	100.000

(2*S*,6*R*)-2,6-Dimethyl-4-(((1*R*,2*S*)-2-phenylcyclobutyl)methyl)morpholine (9h)

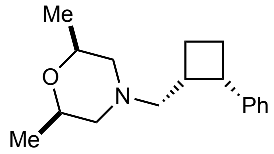

9h

GC-FID analysis: ChiralDex β-DM (110 °C; isocratic; linear velocity 40 cm·s^{-1}; split ratio 10.0) t_R = 25.6 min (major, 91.3%), t_R = 26.5 min (minor, 8.7%)

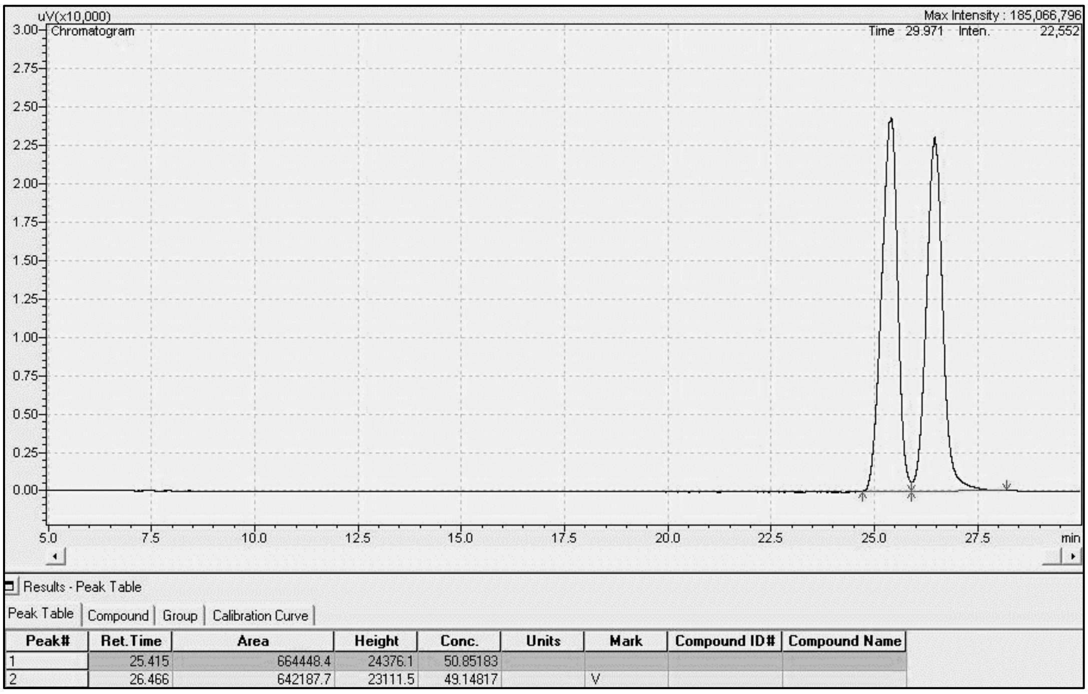

Peak#	Ret.Time	Area	Height	Conc.	Units	Mark	Compound ID#	Compound Name
1	25.415	664448.4	24376.1	50.85183				
2	26.466	642187.7	23111.5	49.14817		V		

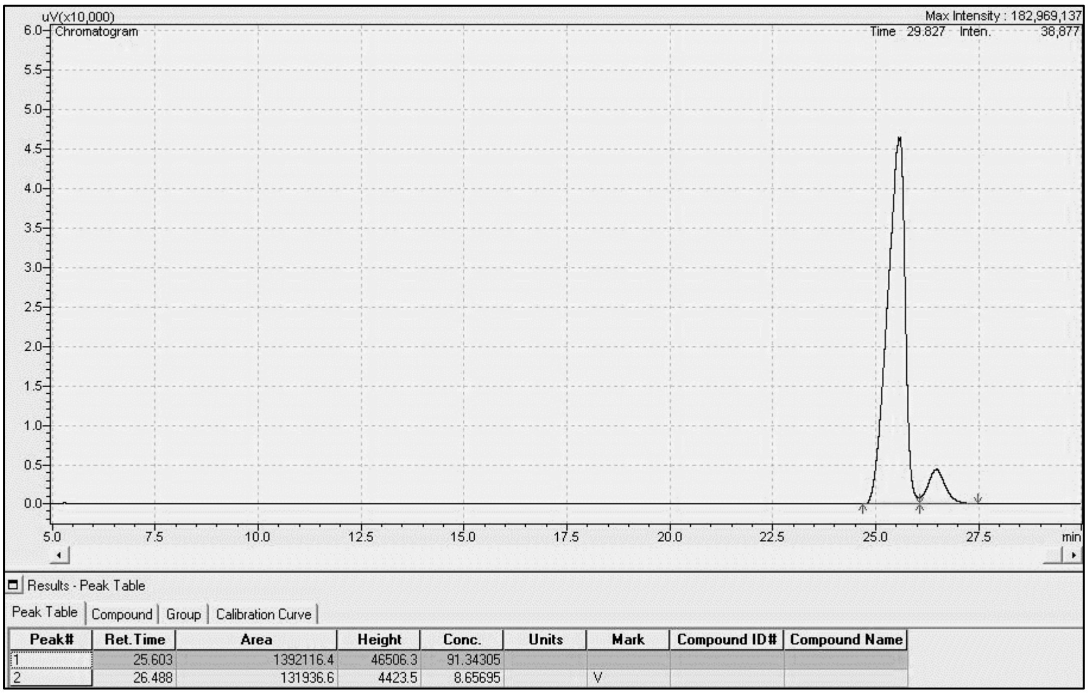

Peak#	Ret.Time	Area	Height	Conc.	Units	Mark	Compound ID#	Compound Name
1	25.603	1392116.4	46506.3	91.34305				
2	26.488	131936.6	4423.5	8.65695		V		

1-(4-Nitrophenyl)-4-(((1*R*,2*S*)-2-phenylcyclobutyl)methyl)piperazine (9i)

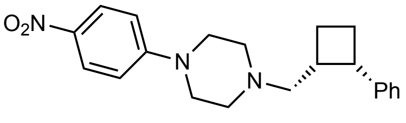

9i

HPLC analysis: Chiralpak AD-H (hexane(0.1% DEA):2-propanol 98:2, 1.0 mL·min^{-1}, 30 °C) t_R = 23.3 min (major, 94.1%), t_R = 26.5 min (minor, 5.9%)

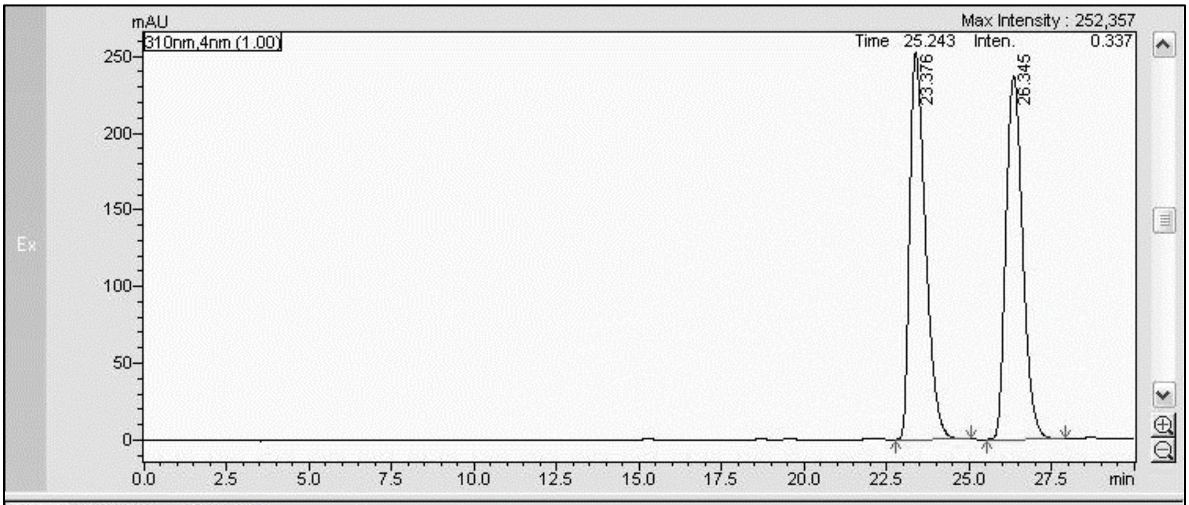

Results View - Peak Table

Peak Table | Compound | Group | Calibration Curve

Peak#	Ret. Time	Area	Height	Area%
1	23.376	8405251	252063	50.042
2	26.345	8391033	237142	49.958
Total		16796285	489206	100.000

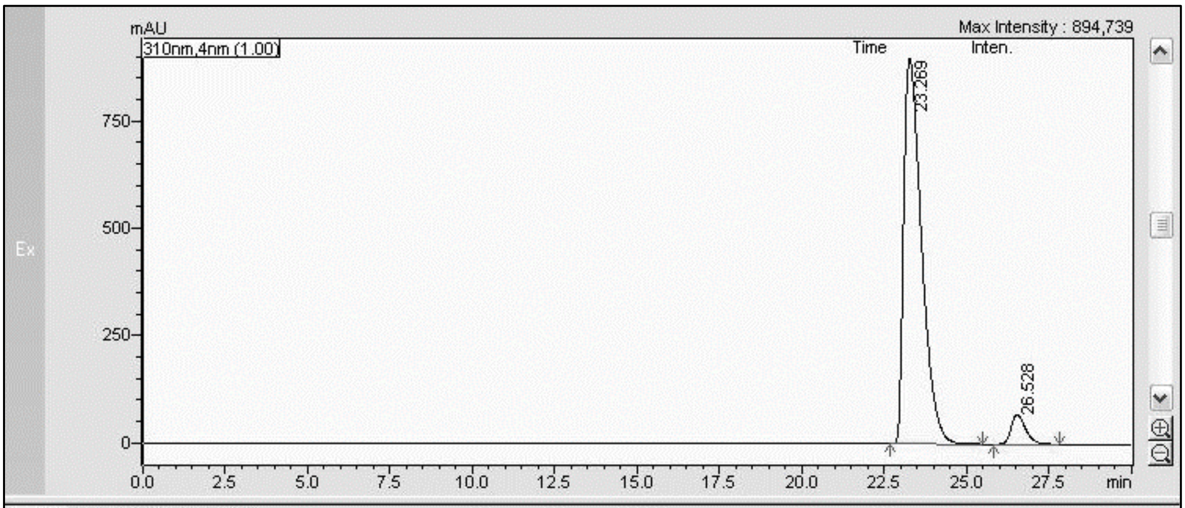

Results View - Peak Table

Peak Table | Compound | Group | Calibration Curve

Peak#	Ret. Time	Area	Height	Area%
1	23.269	33898114	897172	94.062
2	26.528	2139766	69422	5.938
Total		36037880	966594	100.000

1-((1*S*,2*R*)-1-(Methoxymethyl)-2-phenylcyclobutyl)-*N*,*N*-dimethylmethanamine (9j)

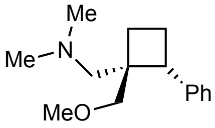

9j

GC-FID analysis: ChiralDex β-DM (50 °C to 72 °C; 0.15 °C/min; linear velocity 40 cm·s^{-1}; split ratio 10.0) t_R = 131.3 min (minor, 11.7%), t_R = 134.3 min (major, 88.3%)

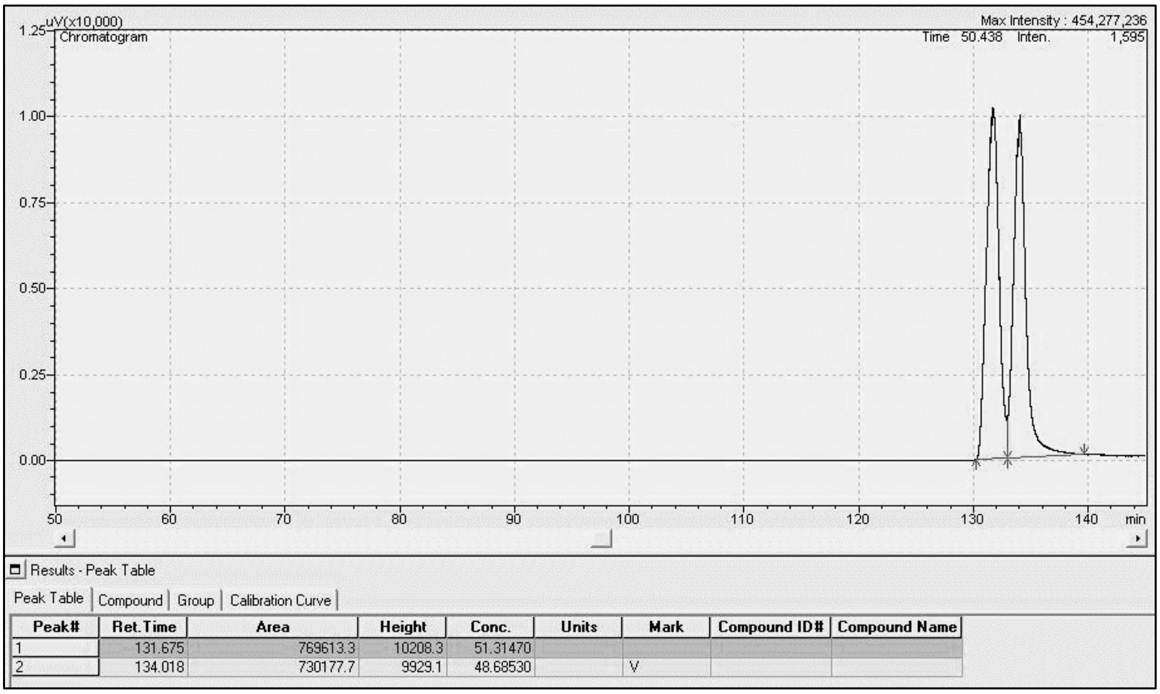

Peak#	Ret.Time	Area	Height	Conc.	Units	Mark	Compound ID#	Compound Name
1	131.675	769613.3	10208.3	51.31470				
2	134.018	730177.7	9929.1	48.68530		V		

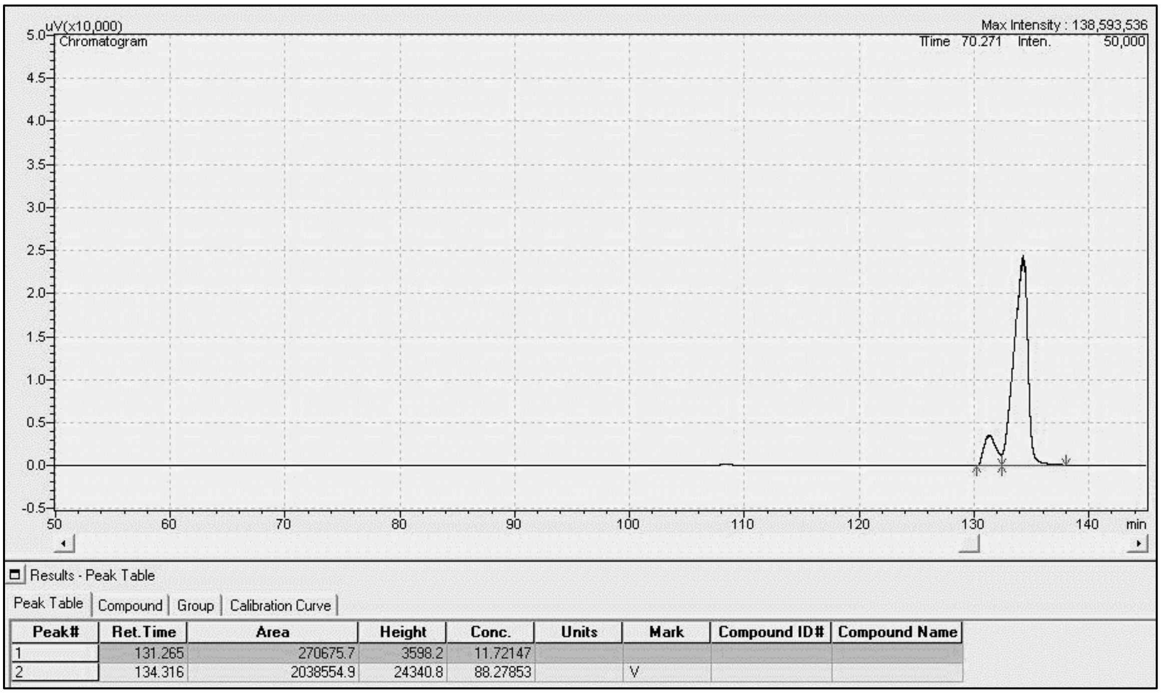

Peak#	Ret.Time	Area	Height	Conc.	Units	Mark	Compound ID#	Compound Name
1	131.265	270675.7	3598.2	11.72147				
2	134.316	2038554.9	24340.8	88.27853		V		

1-((1*R*,2*S*)-2-(4-Chlorophenyl)cyclobutyl)-*N*,*N*-dimethylmethanamine (9aa)

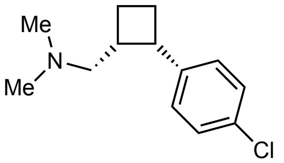

9aa

HPLC analysis: Chiralpak AD-H (hexane(0.1% DEA):2-propanol 99:1, 1.0 mL·min^{-1}, 30°C), t_R = 15.0 min (minor, 1.1%), t_R = 8.0 min (major, 98.9%)

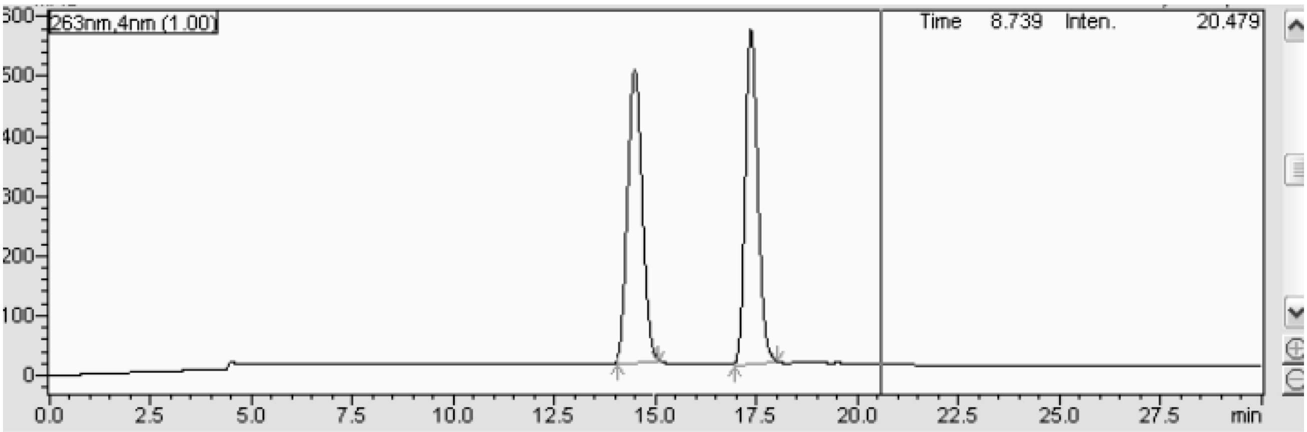

ılts View - Peak Table

Compound | Group | Calibration Curve

Ret. Time	Area	Height	Area%
14.767	12377872	490565	50.548
17.495	12585563	562466	49.461
	24963435	1053261	100.000

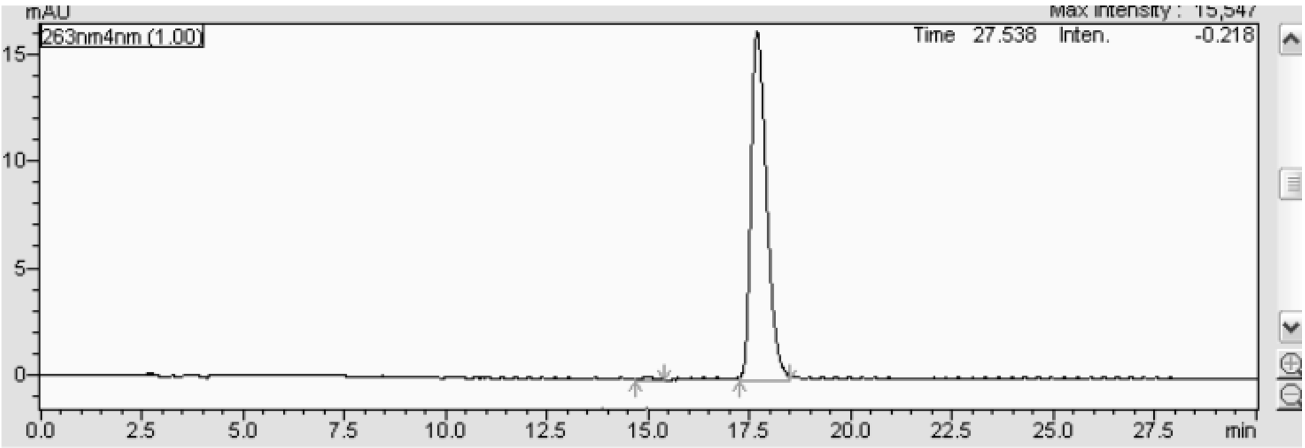

lts View - Peak Table

Compound | Group | Calibration Curve

Ret. Time	Area	Height	Area%
15.003	8800	209	1.109
17.784	512080	16476	98.891
	520880	16684	100.000

***N*,*N*-Dimethyl-1-((1*R*,2*S*)-2-(4-nitrophenyl)cyclobutyl)methanamine (9ab)**

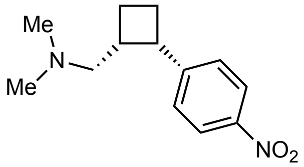

9ab

HPLC analysis: Chiralpak AD-H (hexane(0.1% DEA):2-propanol 99.6:0.4, 1.0 mL·min^{-1}, 30°C), t_R = 23.0 min (major, 98.7%), t_R = 8.0 min (minor, 1.3%)

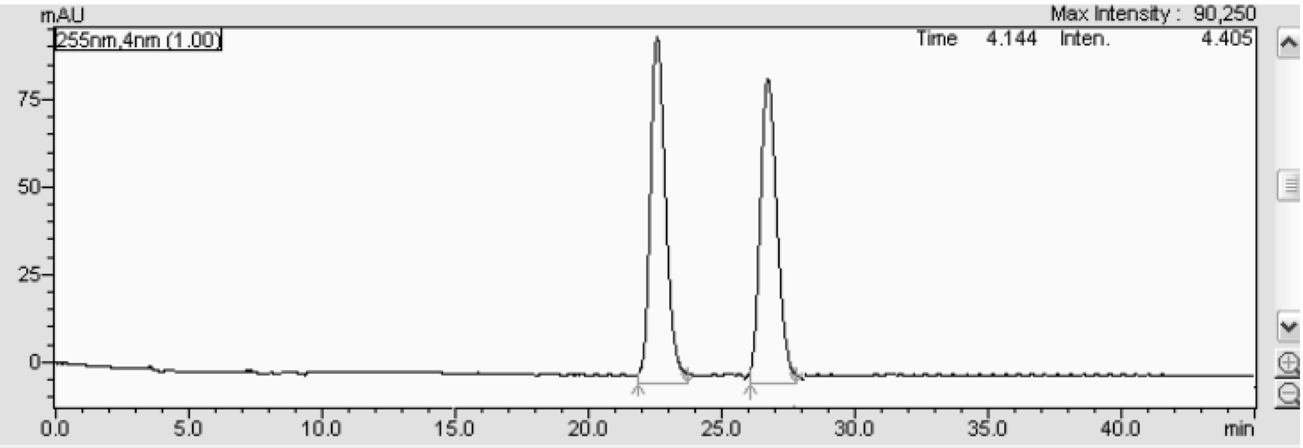

sults View - Peak Table

Compound | Group | Calibration Curve

Ret. Time	Area	Height	Area%
22.898	3992786	98872	50.451
26.971	3843772	86908	49.549
	7836558	185780	100.000

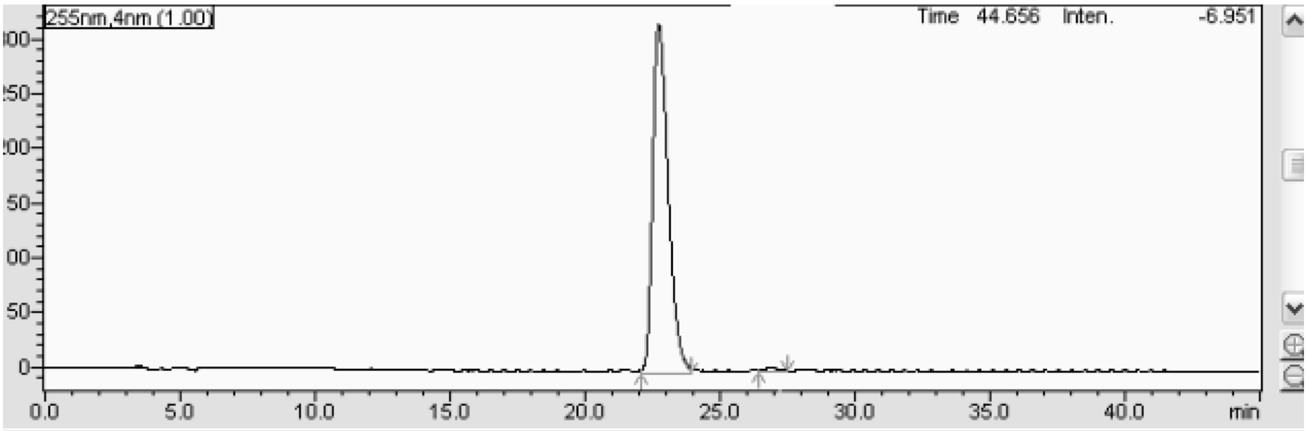

lts View - Peak Table

Compound | Group | Calibration Curve

Ret. Time	Area	Height	Area%
22.979	12832209	319082	98.663
26.988	151316	4065	1.337
	12958525	322047	100.000

1-((1*R*,2*S*)-2-(4-Methoxyphenyl)cyclobutyl)-*N*,*N*-dimethylmethanamine (9ac)

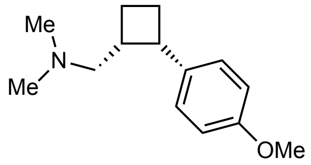

9ac

HPLC analysis: Chiralpak AD-H (hexane(0.1% DEA):2-propanol 99:1, 1.0 mL·min^{-1}, 30°C), t_R = 13.4 min (minor, 1.7%), t_R = 14.1 min (major, 98.3%)

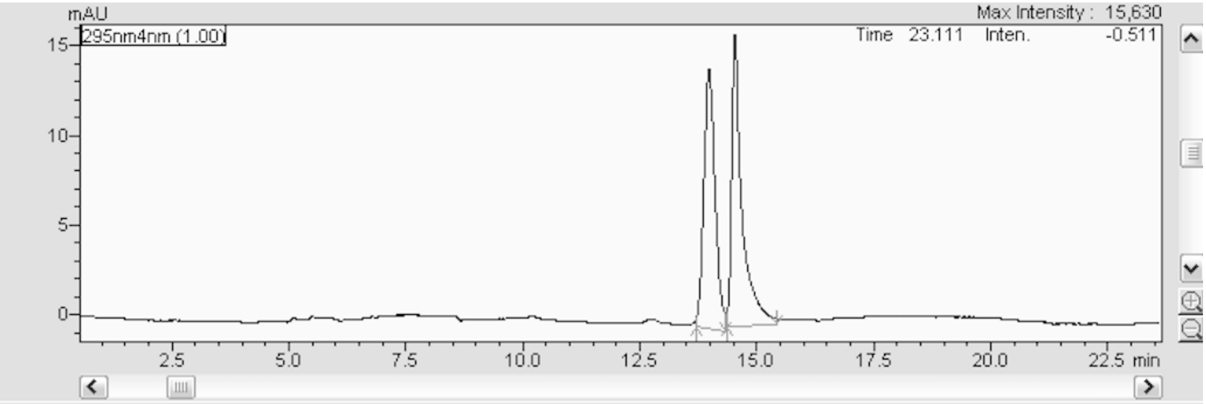

Results View - Peak Table

Table | Compound | Group | Calibration Curve

Pk#	Ret. Time	Area	Height	Area%
	13.979	241837	14436	48.915
	14.532	252565	16274	51.085

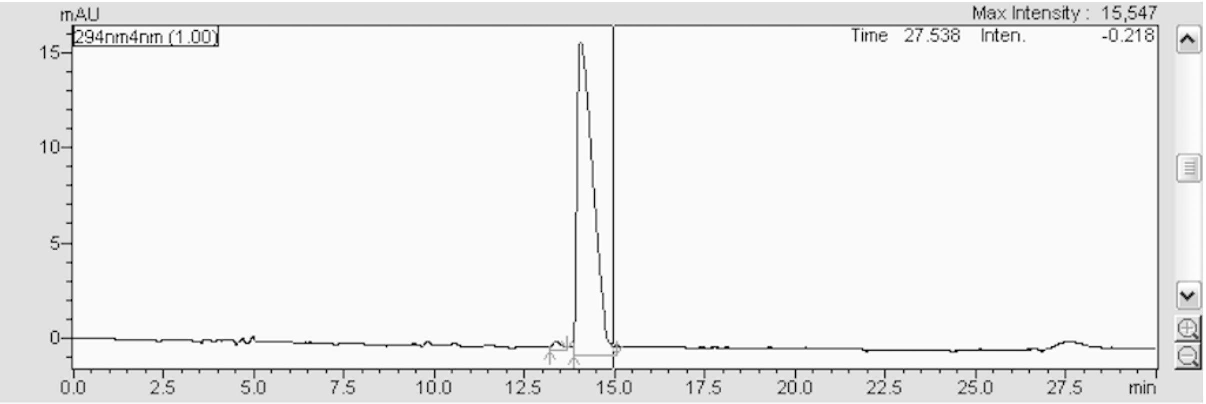

Results View - Peak Table

Table | Compound | Group | Calibration Curve

#	Ret. Time	Area	Height	Area%
	13.383	8800	469	1.689
	14.054	512080	16476	98.311

Ethyl 4-((1*S*,2*R*)-2-((dimethylamino)methyl)cyclobutyl)benzoate (9ad)

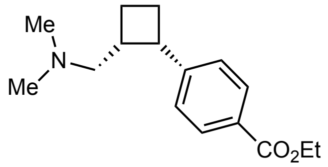

9ad

Chiralpak AD-H (hexane(0.1% DEA):2-propanol 99:1, 1.0 mL·min^{-1}, 30°C), t_R = 18.9 min (minor, 1.6%), t_R = 20.0 min (major, 98.4%)

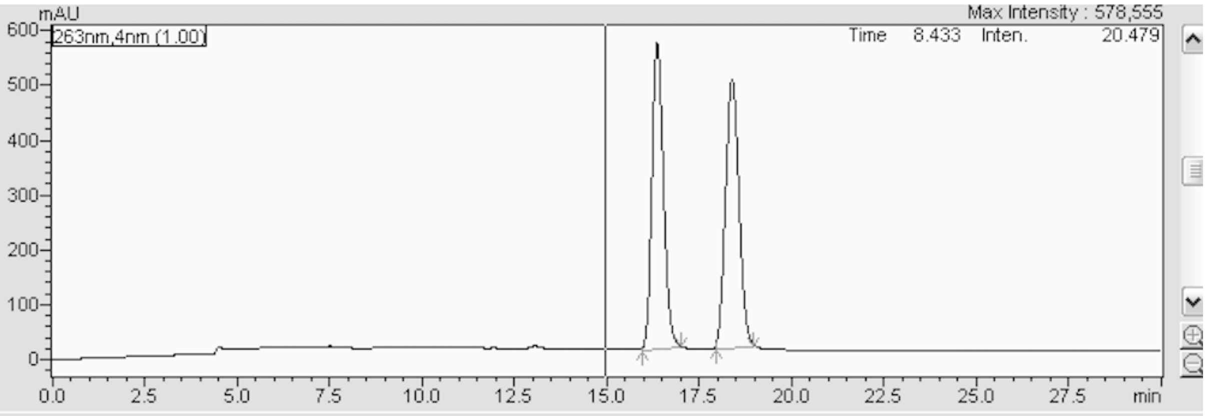

sults View - Peak Table

Compound | Group | Calibration Curve

Ret. Time	Area	Height	Area%
16.361	12377872	560765	49.584
18.392	12585563	492496	50.416

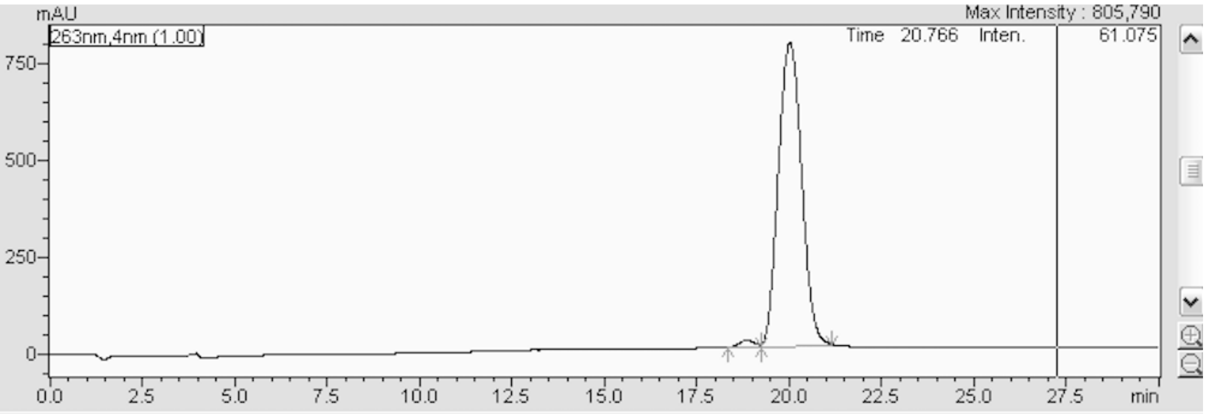

sults View - Peak Table

Compound | Group | Calibration Curve

Ret. Time	Area	Height	Area%
18.858	555127	18137	1.586
20.017	34446751	784827	98.414

_N_,_N_-Dimethyl-1-((1_R_,2_S_)-2-(4-(trifluoromethoxy)phenyl)cyclobutyl)methanamine (9ae)

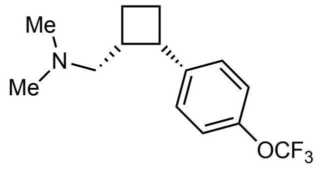

9ae

HPLC analysis: Chiralpak AD-H (hexane(0.1% DEA):2-propanol 98:2, 1.0 mL·min^{-1}, 20°C), t_R = 10.8 min (minor, 1.0%), t_R = 12.0 min (major, 99.0%)

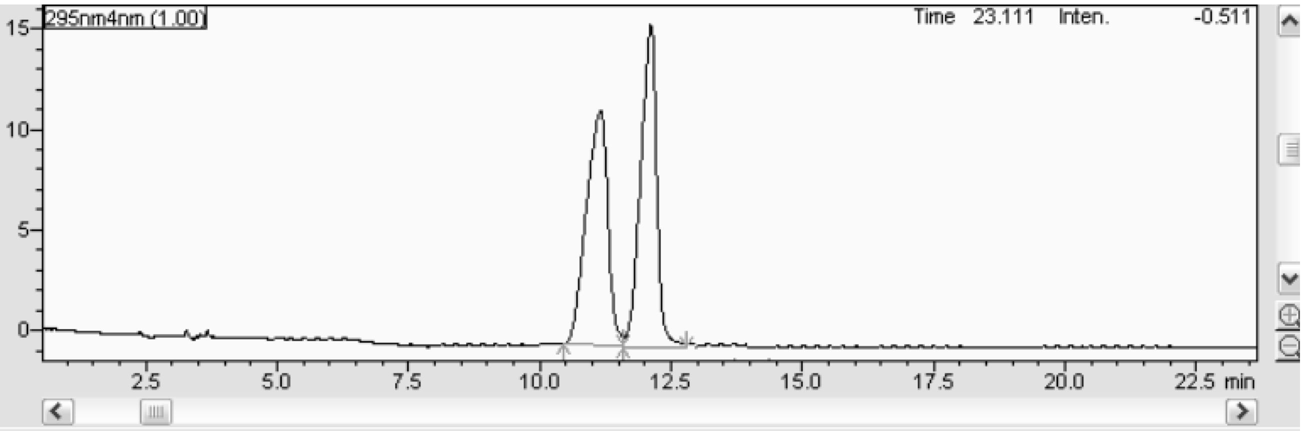

lts View - Peak Table

Compound | Group | Calibration Curve

Ret. Time	Area	Height	Area%
11.029	241837	15136	48.915
12.032	252565	11274	51.085
	494402	30710	100.000

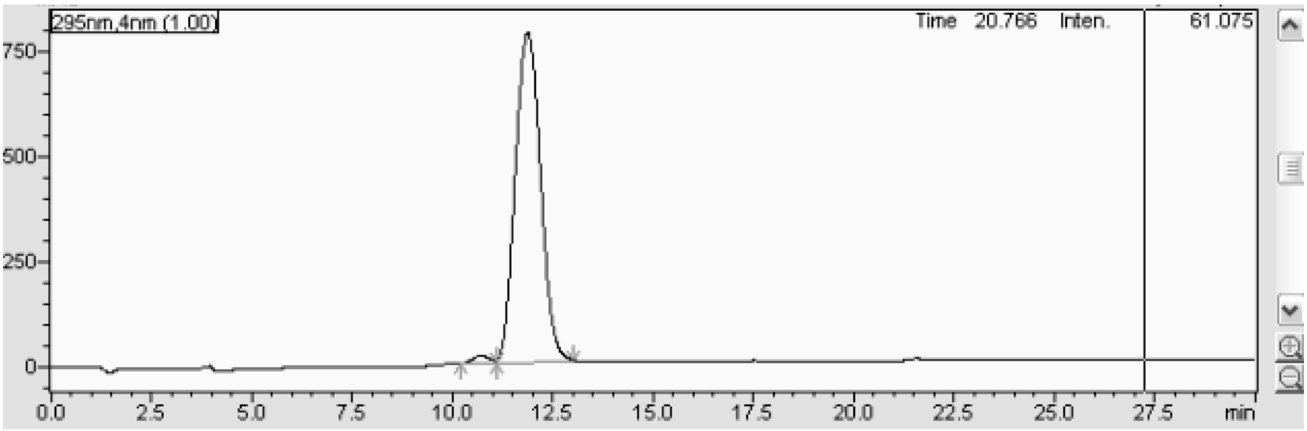

lts View - Peak Table

Compound | Group | Calibration Curve

Ret. Time	Area	Height	Area%
10.808	455127	18137	0.986
12.017	34446751	824827	99.114
	35001877	842964	100.000

1-((1*R*,2*S*)-2-(3-bromophenyl)cyclobutyl)-*N*,*N*-dimethylmethanamine (9af)

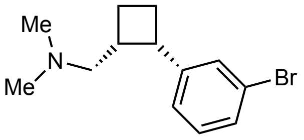

9af

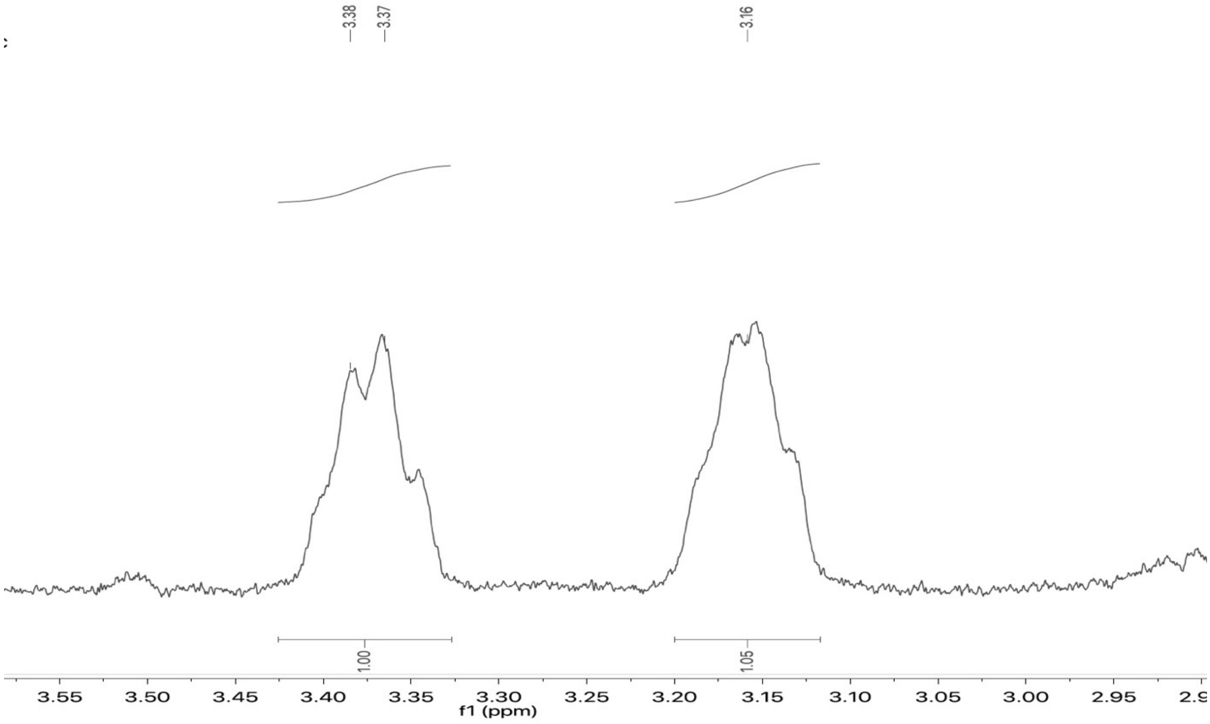

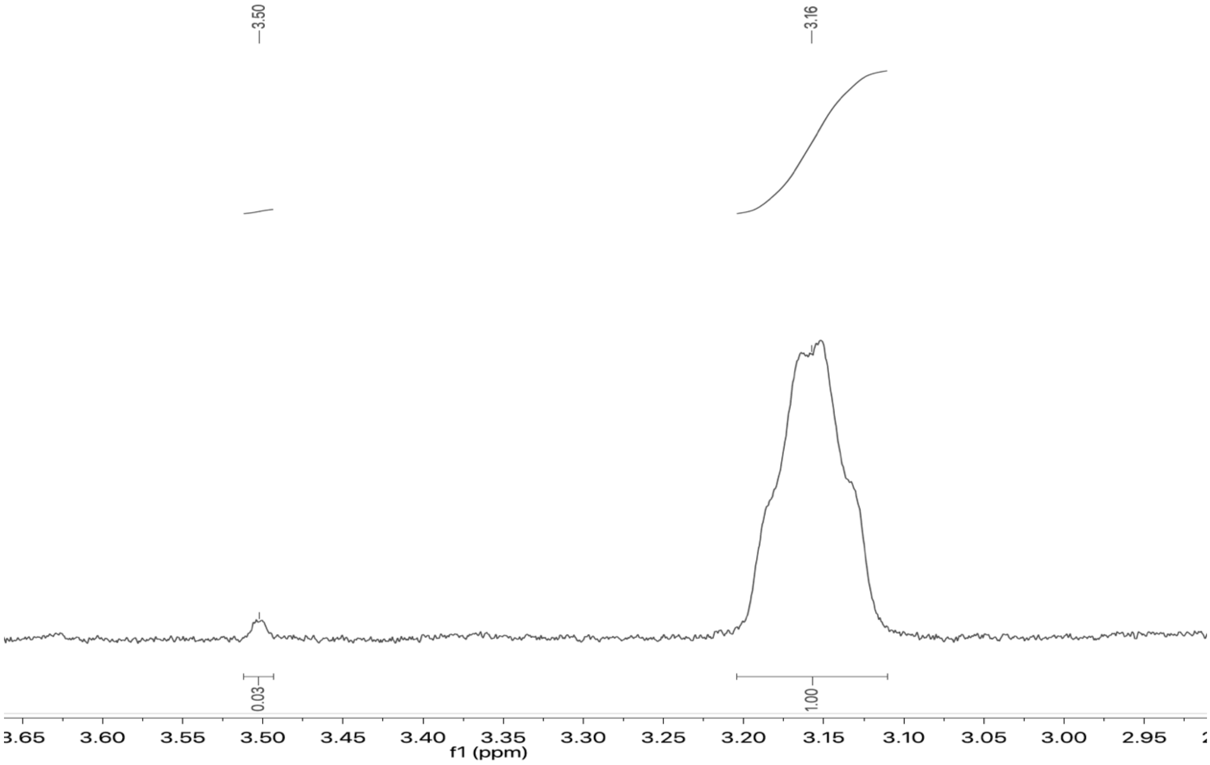

1-((1*R*,2*S*)-2-(4-(Benzyloxy)-3-fluorophenyl)cyclobutyl)-*N*,*N*-dimethylmethanamine (9ag)

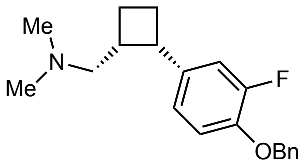

9ag

HPLC analysis: Chiralpak AD-H (hexane(0.1% DEA):2-propanol 99.5:0.5, 1.0 mL·min^{-1},40°C), t_R = 17.9 min (minor, 1.1%), t_R = 20.0 min (major, 98.9%)

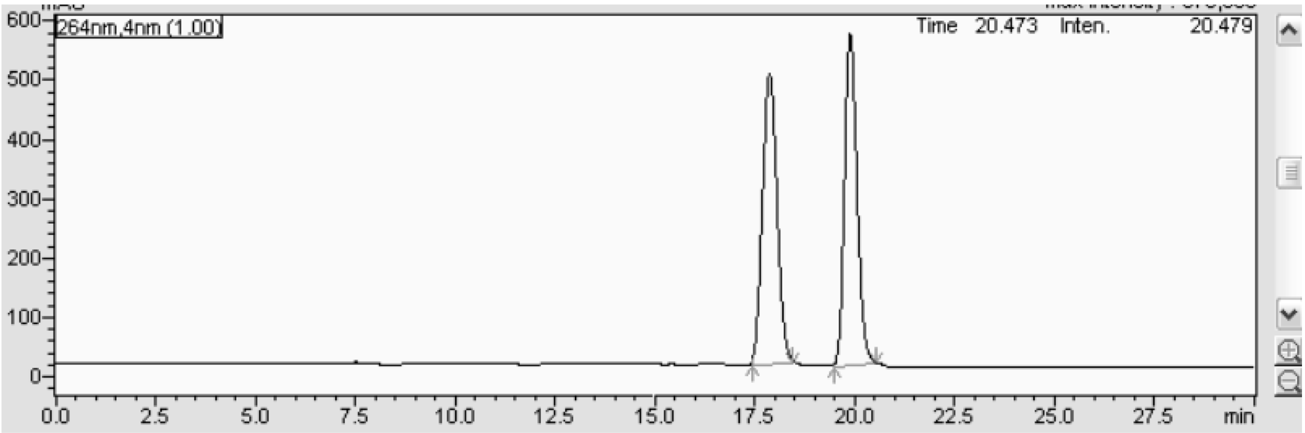

ults View - Peak Table

Compound | Group | Calibration Curve

Ret. Time	Area	Height	Area%
17.961	12377872	490765	49.584
19.982	12585563	562496	50.416
	24963435	1053261	100.000

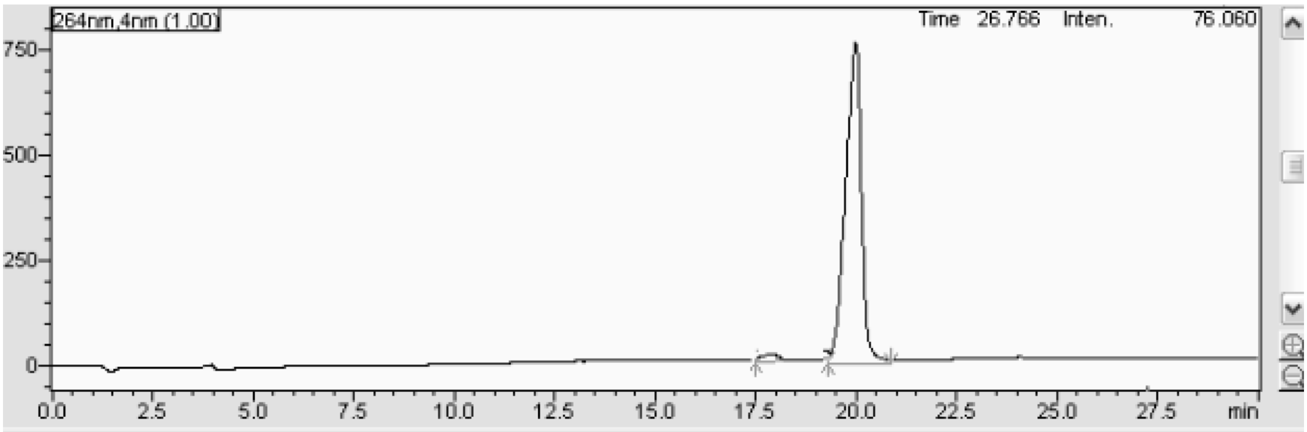

ults View - Peak Table

Compound | Group | Calibration Curve

Ret. Time	Area	Height	Area%
17.858	485377	16137	1.076
19.987	37846780	787967	98.924
	38322057	802094	100.000

6. DFT calculations

All calculations were made using the Gaussian 16 (Revision A.03) program.[1] All geometries were optimised at the B3LYP-D3BJ/[6-31G(d,p)/Lanl2dz(Pd)] level of theory, accounting for solvation effects using the self-consistent reaction field polarizable continuum model (IEF-PCM)[2] in DMF t 323.15 K, c = 0.1 M. Single-point energies of the optimised structures were calculated at the B3LYP-D3BJ/[6-311+G(2d,p)/SDD(Pd)] level of theory, accounting for solvation as above. These functionals and basis sets have been employed in Pd-catalysed C – H activation previously.[3] Dispersion was accounted for using Grimme's DFT-D3 scheme[4] with Becke-Johnson damping.[5] To confirm that the obtained transition states reside along a relevant reaction coordinate, IRC-calculations were undertaken for selected transition states.[6] Basis-set superposition error (BSSE) was assumed to be negligible for the large basis sets employed as indicated by previous studies.[7]

[1] Gaussian 16, Revision A.03, Frisch, M. J.; Trucks, G. W.; Schlegel, H. B.; Scuseria, G. E.; Robb, M. A.; Cheeseman, J. R.; Scalmani, G.; Barone, V.; Petersson, G. A.; Nakatsuji, H.; Li, X.; Caricato, M.; Marenich, A. V.; Bloino, J.; Janesko, B. G.; Gomperts, R.; Mennucci, B.; Hratchian, H. P.; Ortiz, J. V.; Izmaylov, A. F.; Sonnenberg, J. L.; Williams-Young, D.; Ding, F.; Lipparini, F.; Egidi, F.; Goings, J.; Peng, B.; Petrone, A.; Henderson, T.; Ranasinghe, D.; Zakrzewski, V. G.; Gao, J.; Rega, N.; Zheng, G.; Liang, W.; Hada, M.; Ehara, M.; Toyota, K.; Fukuda, R.; Hasegawa, J.; Ishida, M.; Nakajima, T.; Honda, Y.; Kitao, O.; Nakai, H.; Vreven, T.; Throssell, K.; Montgomery, J. A., Jr.; Peralta, J. E.; Ogliaro, F.; Bearpark, M. J.; Heyd, J. J.; Brothers, E. N.; Kudin, K. N.; Staroverov, V. N.; Keith, T. A.; Kobayashi, R.; Normand, J.; Raghavachari, K.; Rendell, A. P.; Burant, J. C.; Iyengar, S. S.; Tomasi, J.; Cossi, M.; Millam, J. M.; Klene, M.; Adamo, C.; Cammi, R.; Ochterski, J. W.; Martin, R. L.; Morokuma, K.; Farkas, O.; Foresman, J. B.; Fox, D. J. Gaussian, Inc., Wallingford CT, 2016.

[2] Original report: Tomasi, J. *et al. Chem. Phys.*, **1981**, *55*, 117-129. Review including references to further modifications of the model: J. Tomasi, J. *et al. Chem. Rev.*, **2005**, *105*, 2999-3093.

[3] Musaev, D.G. *et al. ACS Catal.* **2015**, *5*, 830−840; Musaev, D.G. *et al. ACS Catal.* **2017**, 7, 4344−4354; Yu, J.-Q. *et al. J. Am. Chem.Soc.* **2012**, *134*, 1690−1698; Wu, Y.-D. *J. Am. Chem. Soc.,* **2014**, *136*, 894−897

[4] Grimme, S. *et. al. Chem. Phys.* **2010**, *132*, 154104

[5] Grimme, S. *et. al. J. Comput. Chem.,* **2011**, *32*, 1456-1465.

[6] Hratchian, H.P. & Schlegel, H.B. *J. Chem. Theory and Comput.*, **2005**, *1*, 61-69.

[7] Musaev, D.G. *et al. ACS Catal.* **2017**, 7, 4344−4354; Rzepa, H. *et al. Chem. Sci.*, **2014**, *5*, 2057-2071

Nuclear Coordinates for TSs

TS1

Geometry optimisation:

B3LYP-D3BJ/631G(d,p)/Lanl2dz-IEFPCM(DMF) Energy = -1011.20942385

Thermal correction to Gibbs Free Energy = 0.334309

Imaginary frequency = -940.7262

Single-point energy calculation:

B3LYP-D3BJ/6311+G(2d,p)/SDD-IEFPCM(DMF) Energy = -1012.64713405

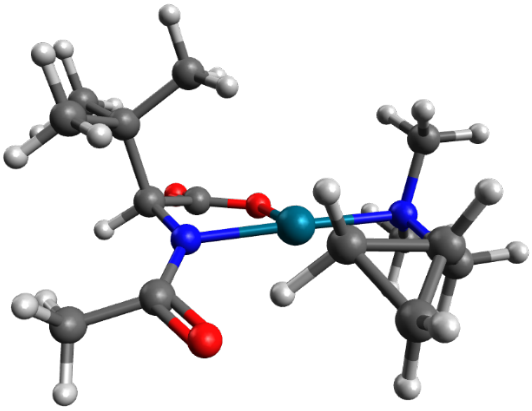

O	2.45186	-2.26693	-1.55257	H	3.72139	0.88865	2.51472
C	1.67646	-1.45984	-1.04034	H	2.33406	1.64008	1.71398
C	2.19833	-0.12646	-0.45878	H	3.90961	1.58058	0.89900
H	2.97960	0.23296	-1.13474	H	2.39071	-1.18824	2.88992
C	2.86437	-0.34135	0.94909	H	1.66195	-2.07498	1.54200
C	4.15445	-1.16033	0.77491	H	0.98137	-0.52163	2.04733
C	3.22439	1.02739	1.54914	H	3.13797	2.15862	-1.50389
C	1.91482	-1.07614	1.91021	H	2.64173	3.47127	-0.42424
N	1.07827	0.80678	-0.41581	H	2.04612	3.43816	-2.08656
Pd	-0.68580	-0.11953	-0.15634	H	-1.32796	1.79517	1.78726
O	0.39197	-1.68561	-0.92799	H	-0.97156	1.97320	-0.09335
N	-2.51825	-1.17455	-0.00590	H	-3.57177	0.89043	1.91279
C	-3.56255	-0.10571	-0.05794	C	-2.99184	2.38661	0.42682
C	-3.20075	1.00288	0.89841	H	-3.11195	2.59458	-0.63262
C	-1.75557	1.53822	0.82138	H	-3.24486	3.20988	1.08673
C	-2.69066	-2.12924	-1.12223	H	-4.54813	-0.53712	0.16313
C	-2.55589	-1.90027	1.28502	H	-3.57563	0.26775	-1.08458
C	1.07105	2.07397	-0.78075	H	-1.75741	-2.64320	1.29545
C	2.31036	2.81216	-1.23212	H	-3.52366	-2.40328	1.41057
O	-0.01313	2.75457	-0.74517	H	-2.40095	-1.20098	2.10594
H	4.64053	-1.29858	1.74637	H	-1.86683	-2.84218	-1.10566
H	4.86058	-0.64234	0.11621	H	-2.66791	-1.58378	-2.06665
H	3.94895	-2.14072	0.34216	H	-3.64688	-2.65906	-1.02894

TS2

Geometry optimisation:

B3LYP-D3BJ/631G(d,p)/Lanl2dz-IEFPCM(DMF) Energy = -1011.18927119

Thermal correction to Gibbs Free Energy = 0.333940

Imaginary frequency = -1247.7195

Single-point energy calculation:

B3LYP-D3BJ/6311+G(2d,p)/SDD-IEFPCM(DMF) Energy = -1012.62775810

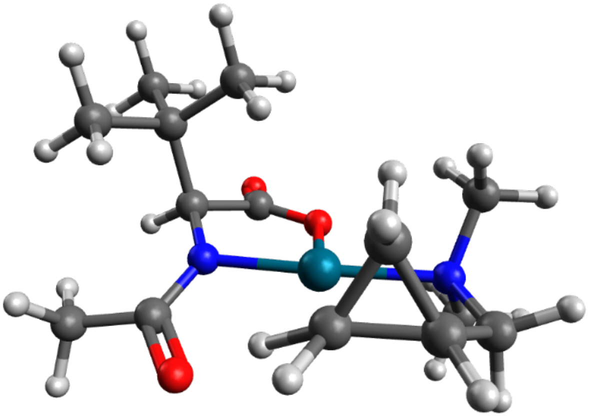

O	2.22742	-2.52190	-1.33923	H	3.75348	0.84673	2.46046
C	1.53561	-1.59962	-0.91474	H	2.47244	1.66537	1.55707
C	2.17241	-0.27391	-0.44373	H	4.04579	1.35810	0.79384
H	2.99144	-0.04442	-1.13242	H	2.20019	-1.03385	2.98927
C	2.79323	-0.42550	0.99283	H	1.39134	-1.95123	1.70935
C	3.98930	-1.39014	0.92629	H	0.88888	-0.29071	2.05817
C	3.29210	0.94686	1.47280	H	3.31965	1.89504	-1.57814
C	1.75323	-0.95841	1.99272	H	2.87366	3.31088	-0.61205
N	1.13923	0.75108	-0.50700	H	2.34054	3.19952	-2.29261
Pd	-0.71556	0.02848	-0.26062	H	-0.83076	1.96633	-0.33168
O	0.23077	-1.69365	-0.80083	H	-4.00120	2.03432	0.88374
N	-2.59563	-0.95619	-0.11941	C	-2.19157	1.33585	1.93513
C	-3.68330	0.04147	0.12634	H	-1.74880	0.41573	2.29766
C	-3.20468	1.29593	0.81288	H	-2.34815	2.07374	2.71741
C	-1.82219	1.84776	0.55222	H	-1.64982	-2.59507	0.80273
C	-2.82834	-1.66687	-1.40267	H	-3.42951	-2.57498	0.97891
C	-2.52050	-1.96090	0.97141	H	-2.42537	-1.46142	1.93369
C	1.23156	1.99212	-0.94151	H	-2.83707	-0.94265	-2.21787
C	2.52932	2.61979	-1.38697	H	-3.78759	-2.19837	-1.37347
O	0.18263	2.72344	-0.97985	H	-2.01584	-2.37567	-1.56039
H	4.44835	-1.47921	1.91634	H	-4.49278	-0.44580	0.68436
H	4.75303	-1.01917	0.23364	H	-4.08653	0.32811	-0.84870
H	3.68562	-2.38296	0.59057	H	-1.80402	2.93757	0.51511

TS3

Geometry optimisation:

B3LYP-D3BJ/631G(d,p)/Lanl2dz-IEFPCM(DMF) Energy = -1011.19988749

Thermal correction to Gibbs Free Energy = 0.333709

Imaginary frequency = -935.8338

Single-point energy calculation:

B3LYP-D3BJ/6311+G(2d,p)/SDD-IEFPCM(DMF) Energy = -1012.63840447

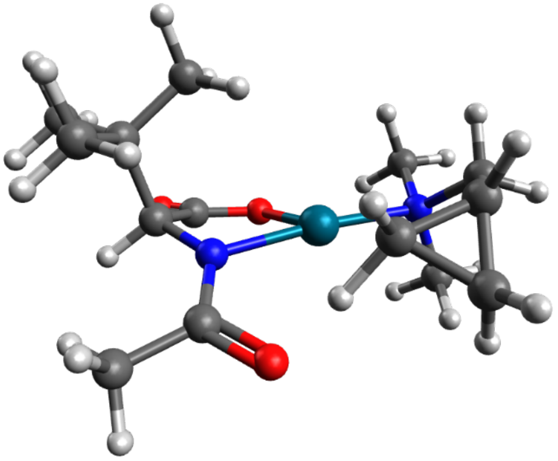

O	2.44595	-2.29791	-1.49834	H	3.75248	0.94375	2.49110
C	1.67488	-1.47518	-1.00508	H	2.35667	1.67236	1.68094
C	2.20678	-0.13597	-0.44244	H	3.92573	1.59579	0.85649
H	2.98175	0.21242	-1.13130	H	2.42394	-1.13439	2.92185
C	2.88423	-0.32527	0.96113	H	1.68687	-2.04668	1.59577
C	4.17248	-1.14810	0.79336	H	1.00886	-0.48384	2.07631
C	3.24733	1.05746	1.52646	H	-3.14037	-1.09127	1.93672
C	1.94162	-1.04073	1.94332	H	-4.42640	-0.61591	0.82623
N	1.08895	0.79890	-0.40426	H	-2.57871	-1.30784	-2.16455
Pd	-0.67490	-0.10980	-0.12084	H	-3.32836	0.18530	-1.56928
O	0.38664	-1.68505	-0.90218	H	-4.19171	-1.36281	-1.39956
N	-2.55453	-1.09725	-0.06631	H	-1.87410	-2.71672	1.10212
C	-3.35649	-0.50062	1.04426	H	-1.76458	-2.95782	-0.65040
C	-2.99005	0.93403	1.36159	H	-3.35725	-3.05602	0.16350
C	-1.68566	1.57947	0.85999	H	3.12229	2.12133	-1.57177
C	-3.20631	-0.87996	-1.38201	H	2.58598	3.47511	-0.56665
C	-2.38199	-2.55396	0.15110	H	2.01725	3.34976	-2.23499
C	1.04743	2.03367	-0.87257	H	-1.15064	2.12187	1.63539
C	2.27831	2.77191	-1.34665	H	-1.01119	1.87718	-0.17610
O	-0.05682	2.67783	-0.90719	H	-3.16957	1.15475	2.41100
H	4.66654	-1.26693	1.76338	C	-3.08441	2.12091	0.48244
H	4.87339	-0.64411	0.11841	H	-3.36781	2.00380	-0.55840
H	3.96261	-2.13690	0.38228	H	-3.39405	3.06215	0.92624

TS4

Geometry optimisation:

B3LYP-D3BJ/631G(d,p)/Lanl2dz-IEFPCM(DMF) Energy = -1011.20061195

Thermal correction to Gibbs Free Energy = 0.334976

Imaginary frequency = -947.0548

Single-point energy calculation:

B3LYP-D3BJ/6311+G(2d,p)/SDD-IEFPCM(DMF) Energy = -1012.63836876

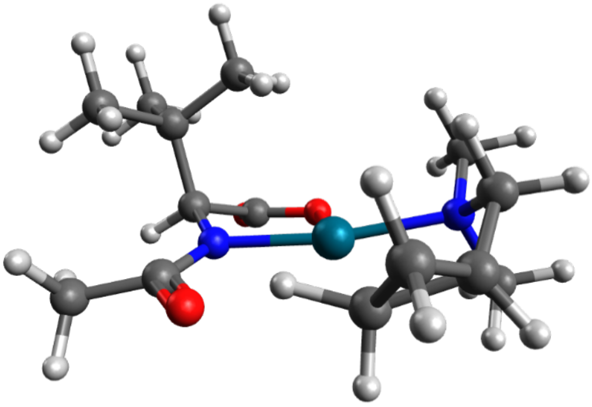

O	2.35797	-2.31980	-1.61048	H	3.76744	0.77512	2.47450
C	1.63484	-1.50553	-1.03876	H	2.48064	1.65214	1.64728
C	2.18910	-0.16958	-0.51419	H	4.05556	1.42152	0.85679
H	2.99627	0.14170	-1.18443	H	2.26516	-1.11332	2.88319
C	2.82082	-0.38814	0.91804	H	1.46135	-1.98426	1.56601
C	4.03540	-1.32408	0.78464	H	0.92396	-0.35841	2.00436
C	3.30522	0.95025	1.49771	H	-2.67321	-2.12229	-1.70138
C	1.80383	-1.00062	1.89664	H	-3.49750	-0.55631	-1.56272
N	1.09319	0.79897	-0.52579	H	-4.19233	-1.99861	-0.77079
Pd	-0.71630	-0.07611	-0.29339	H	-1.52619	-2.23122	1.74512
O	0.37142	-1.73797	-0.77972	H	-1.48986	-3.05371	0.17450
N	-2.46431	-1.19090	0.17069	H	-3.00906	-3.01047	1.12362
C	-3.19582	-0.28617	1.10987	H	3.27763	2.14249	-1.30512
C	-3.35182	1.06337	0.46200	H	2.83757	3.43942	-0.17523
C	-2.11236	1.68093	-0.21114	H	2.23354	3.48142	-1.83173
C	-3.26175	-1.48323	-1.04210	H	-2.34764	2.14647	-1.16604
C	-2.10346	-2.45634	0.84740	H	-0.90350	2.01176	-0.07085
C	1.20449	2.11427	-0.58955	C	-2.78753	2.31248	1.02227
C	2.48033	2.81741	-0.99918	H	-4.25309	1.18491	-0.13201
O	0.20070	2.86386	-0.35316	H	-2.58868	-0.21538	2.01568
H	4.50997	-1.45490	1.76258	H	-4.16370	-0.73135	1.37744
H	4.78200	-0.90258	0.10241	H	-2.22149	2.24805	1.94731
H	3.74932	-2.30552	0.40288	H	-3.33516	3.23922	0.88333

TS available for cyclobutane activation

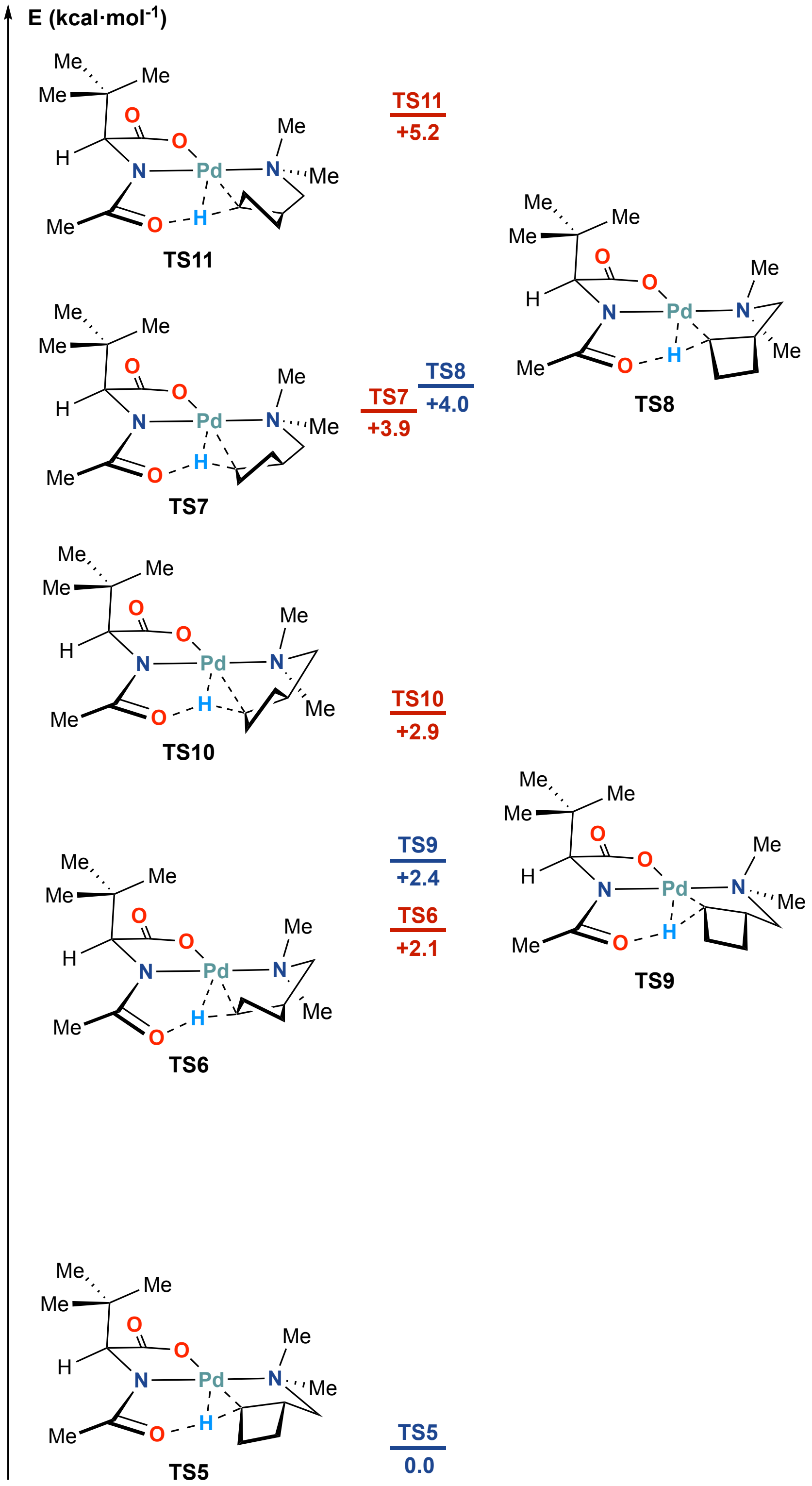

Note: It was not possible to find the diastereomeric pair of TS10. We hypothesize that the far from ideal amidate angle combined with the steric interaction along the amine backbone impedes this TS to find an appropriate energy minimum.

TS5

Geometry optimisation:

B3LYP-D3BJ/631G(d,p)/Lanl2dz-IEFPCM(DMF) Energy = -1050.52662338

Thermal correction to Gibbs Free Energy = 0.361992

Imaginary frequency = -1237.6546

Single-point energy calculation:

B3LYP-D3BJ/6311+G(2d,p)/SDD-IEFPCM(DMF) Energy = -1051.97327000

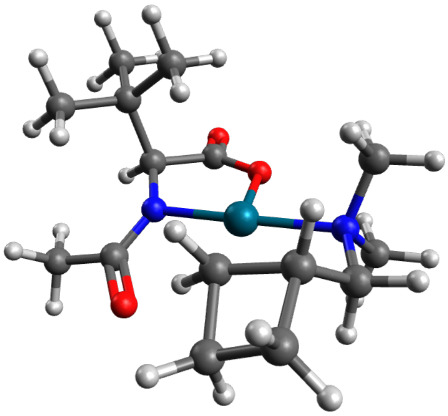

O	2.88521	-2.19742	-1.28350	H	3.89357	1.98144	0.79995
C	2.01351	-1.43519	-0.86770	H	2.56295	-0.70003	3.03467
C	2.37647	-0.00318	-0.40693	H	1.94334	-1.77214	1.76915
H	3.13392	0.37477	-1.10055	H	1.12780	-0.23754	2.10417
C	3.01749	-0.01122	1.02808	H	2.35729	3.65178	-0.66131
C	4.37990	-0.72235	0.96972	H	1.86991	3.38341	-2.33784
C	3.23688	1.43927	1.48777	H	3.08373	2.32342	-1.58023
C	2.10556	-0.72466	2.04019	H	-1.34282	1.73372	1.49797
N	1.16387	0.80206	-0.47543	H	-0.98619	1.61382	-0.36065
Pd	-0.51623	-0.26483	-0.22616	H	-2.97864	0.04918	2.08127
O	0.75684	-1.78830	-0.75882	C	-2.90934	2.38790	0.09443
N	-2.21199	-1.53429	-0.10097	C	-4.00194	1.69189	0.93880
C	-3.39670	-0.64675	0.08018	H	-4.97477	1.53256	0.46356
C	-3.07764	0.45463	1.07111	H	-4.15692	2.18354	1.90209
C	-1.86306	1.34016	0.62083	H	-1.11787	-3.02705	0.89466
C	-2.33732	-2.33341	-1.34118	H	-2.88624	-3.12412	1.14251
C	-2.02731	-2.44665	1.05255	H	-1.92433	-1.87012	1.97113
C	1.01437	2.02222	-0.94798	H	-4.26650	-1.24235	0.38907
C	2.16342	2.88164	-1.41373	H	-3.61699	-0.21110	-0.89842
O	-0.15796	2.53792	-1.00898	H	-1.42866	-2.92106	-1.47191
H	4.84927	-0.70494	1.95887	H	-2.45457	-1.66009	-2.19137
H	5.05462	-0.21874	0.26840	H	-3.20666	-3.00047	-1.28059
H	4.27622	-1.76005	0.64881	H	-2.68358	3.43458	0.30840
H	3.71026	1.44664	2.47484	H	-3.08463	2.28243	-0.97954
H	2.29155	1.98306	1.56475				

TS6

Geometry optimisation:

B3LYP-D3BJ/631G(d,p)/Lanl2dz-IEFPCM(DMF) Energy = -1050.52273402

Thermal correction to Gibbs Free Energy = 0.361915

Imaginary frequency = -1247.2650

Single-point energy calculation:

B3LYP-D3BJ/6311+G(2d,p)/SDD-IEFPCM(DMF) Energy = -1051.96992113

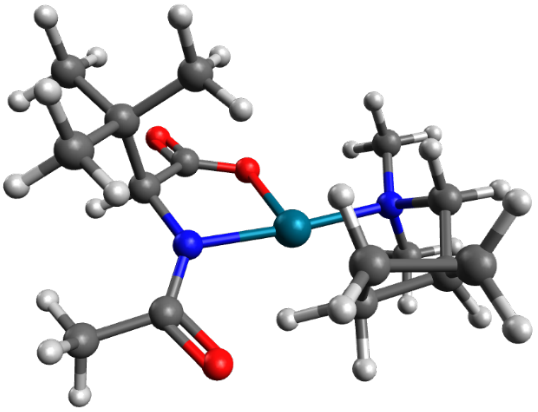

O	2.71878	-2.57379	-0.81692
C	1.89071	-1.68779	-0.60966
C	2.33405	-0.23385	-0.34008
H	3.19286	-0.02920	-0.98666
C	2.81246	-0.05672	1.14775
C	4.11064	-0.85525	1.35375
C	3.09641	1.42998	1.41501
C	1.74376	-0.54545	2.13997
N	1.21115	0.62859	-0.68105
Pd	-0.57716	-0.24733	-0.40560
O	0.60218	-1.91919	-0.56648
N	-2.37280	-1.33286	-0.08062
C	-3.10291	-0.52177	0.93192
C	-3.24362	0.91656	0.46841
C	-1.91120	1.56908	-0.04968
C	-3.13455	-1.44686	-1.34618
C	-2.09708	-2.68470	0.45531
C	1.22607	1.78554	-1.31074
C	2.49315	2.47431	-1.75526
O	0.12035	2.37924	-1.55747
H	4.46873	-0.72394	2.38013
H	4.89715	-0.50538	0.67575
H	3.95768	-1.91995	1.16939
H	3.46090	1.55747	2.43942
H	2.19416	2.03709	1.30250
H	3.86360	1.81933	0.73849
H	2.09014	-0.38804	3.16657
H	1.53146	-1.61118	2.01446
H	0.80490	0.00188	2.01126
H	-2.51061	-0.57031	1.84998
H	-4.08216	-0.97679	1.13771
H	-2.56154	-2.05288	-2.04894
H	-3.29168	-0.45936	-1.77773
H	-4.10614	-1.92393	-1.16264
H	-1.54172	-2.59312	1.38928
H	-1.48881	-3.23620	-0.25983
H	-3.03893	-3.21628	0.63865
H	3.36862	1.82633	-1.74245
H	2.68323	3.32697	-1.09690
H	2.34121	2.85972	-2.76546
H	-2.24052	2.34924	-0.74873
H	-0.86142	1.59573	-0.88580
C	-1.70680	2.21209	1.34444
C	-3.20772	1.94311	1.63689
H	-3.46537	1.56018	2.62890
H	-3.82969	2.81547	1.42214
H	-1.36508	3.25020	1.36510
H	-1.05257	1.61763	1.98719
H	-4.08843	1.01639	-0.21691

TS7

Geometry optimisation:

B3LYP-D3BJ/631G(d,p)/Lanl2dz-IEFPCM(DMF) Energy = -1050.52153746

Thermal correction to Gibbs Free Energy = 0.362753

Imaginary frequency = -1259.5041

Single-point energy calculation:

B3LYP-D3BJ/6311+G(2d,p)/SDD-IEFPCM(DMF) Energy = -1051.96787617

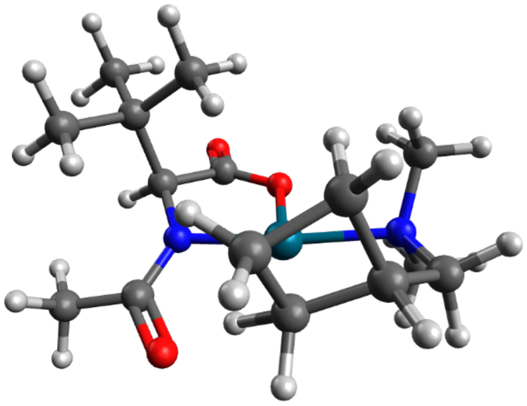

O	2.40364	-2.72878	-0.89708
C	1.66074	-1.77522	-0.67193
C	2.23778	-0.37237	-0.37905
H	3.10284	-0.23581	-1.03531
C	2.75295	-0.26931	1.10342
C	3.96298	-1.20185	1.28186
C	3.19755	1.17503	1.38449
C	1.65177	-0.65515	2.10515
N	1.19668	0.59990	-0.68497
Pd	-0.66158	-0.12059	-0.42388
O	0.35513	-1.89005	-0.63119
N	-2.50576	-1.13221	-0.14157
C	-3.57334	-0.12454	0.14488
C	-3.01141	1.15525	0.72208
C	-1.80143	1.83891	0.00650
C	-2.81176	-1.89857	-1.37383
C	-2.34921	-2.08668	0.98506
C	1.31248	1.76132	-1.29529
C	2.63174	2.33959	-1.74432
O	0.26308	2.45872	-1.51866
H	4.35151	-1.11297	2.30168
H	4.76939	-0.93363	0.59017
H	3.69616	-2.24381	1.09812
H	3.58297	1.25003	2.40635
H	2.36543	1.87764	1.28863
H	3.99571	1.48813	0.70418
H	2.03140	-0.56295	3.12793
H	1.31530	-1.68604	1.96138
H	0.78160	0.00019	2.00772
H	-4.33837	-0.57434	0.78993
H	-4.05051	0.11978	-0.80828
H	-2.00225	-2.60662	-1.55370
H	-2.88469	-1.21160	-2.21774
H	-3.75818	-2.44212	-1.26080
H	-2.19633	-1.54183	1.91442
H	-1.48321	-2.71893	0.79033
H	-3.25097	-2.70486	1.07733
H	2.90469	3.16170	-1.07634
H	2.50473	2.75227	-2.74717
H	3.44515	1.61535	-1.75065
H	-2.13199	2.61106	-0.69927
H	-0.77249	1.76513	-0.84976
H	-3.83612	1.87905	0.74672
C	-1.33769	2.34603	1.39822
C	-2.23721	1.27004	2.06480
H	-1.68993	3.36260	1.60252
H	-0.26732	2.29530	1.61062
H	-1.65913	0.37538	2.30617
H	-2.81940	1.56137	2.94256

TS8

Geometry optimisation:

B3LYP-D3BJ/631G(d,p)/Lanl2dz-IEFPCM(DMF) Energy = -1050.52038307

Thermal correction to Gibbs Free Energy = 0.361667

Imaginary frequency = -1297.2184

Single-point energy calculation:

B3LYP-D3BJ/6311+G(2d,p)/SDD-IEFPCM(DMF) Energy = -1051.96658591

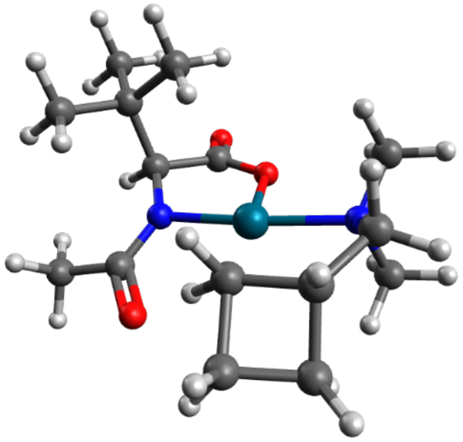

O	2.78206	-2.08508	-1.57346
C	1.94602	-1.35175	-1.04733
C	2.35027	0.01814	-0.46160
H	3.09791	0.45023	-1.13349
C	3.02486	-0.14220	0.94884
C	4.37684	-0.85569	0.77933
C	3.27165	1.25133	1.54876
C	2.13333	-0.95057	1.90669
N	1.15002	0.84322	-0.42400
Pd	-0.53705	-0.21949	-0.16558
O	0.68739	-1.68958	-0.91897
N	-2.18679	-1.54098	0.01150
C	-3.10183	-0.97884	1.04730
C	-3.14761	0.53471	1.05526
C	-1.84458	1.37707	0.83609
C	-2.84460	-1.67853	-1.31031
C	-1.71045	-2.87978	0.44114
C	1.01855	2.10407	-0.77306
C	2.17105	2.98138	-1.19384
O	-0.14226	2.64564	-0.74221
H	4.87114	-0.94969	1.75184
H	5.03999	-0.28608	0.11868
H	4.25237	-1.85208	0.35200
H	3.77152	1.15356	2.51781
H	2.33442	1.79156	1.70723
H	3.91515	1.85641	0.90212
H	2.61737	-1.03098	2.88547
H	1.95496	-1.96357	1.53390
H	1.16097	-0.46832	2.04774
H	-2.70741	-1.30934	2.01140
H	-4.10304	-1.41678	0.93280
H	-2.12146	-2.07946	-2.02162
H	-3.18881	-0.70998	-1.66318
H	-3.70024	-2.36221	-1.23682
H	-1.19292	-2.78807	1.39663
H	-1.01300	-3.26215	-0.30217
H	-2.56088	-3.56435	0.54945
H	2.41099	3.66651	-0.37536
H	1.85693	3.58062	-2.05060
H	3.07048	2.42363	-1.45098
H	-1.28657	1.63173	1.74098
H	-0.97592	1.70756	-0.15098
H	-3.54446	0.82194	2.03768
C	-2.82050	2.49850	0.29550
C	-3.87566	1.41071	0.01217
H	-2.47868	3.10739	-0.54095
H	-3.12002	3.15438	1.11925
H	-4.92669	1.64390	0.20101
H	-3.77409	1.03561	-1.00826

TS9

Geometry optimisation:

B3LYP-D3BJ/631G(d,p)/Lanl2dz-IEFPCM(DMF) Energy = -1050.52413194

Thermal correction to Gibbs Free Energy = 0.362303

Imaginary frequency = -1356.0790

Single-point energy calculation:

B3LYP-D3BJ/6311+G(2d,p)/SDD-IEFPCM(DMF) Energy = -1051.96979036

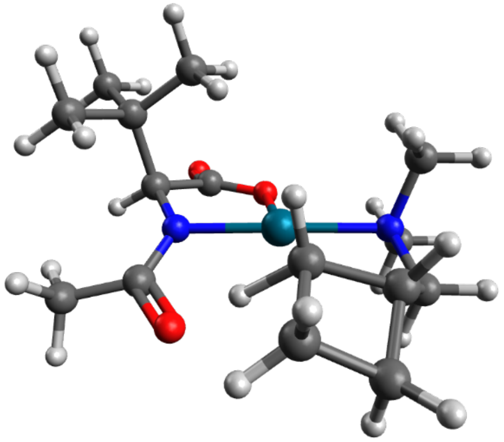

O	2.88126	-2.14506	-1.39508
C	2.01093	-1.40871	-0.93142
C	2.36063	0.02226	-0.46279
H	3.07734	0.43120	-1.18161
C	3.06416	0.00532	0.94292
C	4.44105	-0.66677	0.80870
C	3.26624	1.45155	1.42328
C	2.21834	-0.75289	1.97961
N	1.12619	0.79676	-0.46483
Pd	-0.52378	-0.30924	-0.16781
O	0.76874	-1.79018	-0.77661
N	-2.20300	-1.59324	0.00721
C	-3.38511	-0.71637	-0.22873
C	-3.30438	0.52141	0.64421
C	-1.90369	1.20928	0.78397
C	-2.15172	-2.67386	-1.00276
C	-2.22544	-2.18513	1.36492
C	0.93776	2.02579	-0.88556
C	2.03933	2.92255	-1.38972
O	-0.24620	2.52777	-0.86560
H	4.95315	-0.65793	1.77652
H	5.07032	-0.12963	0.09023
H	4.35037	-1.69957	0.46877
H	3.78369	1.45104	2.38798
H	2.31199	1.96849	1.55534
H	3.87703	2.02509	0.71893
H	2.71978	-0.73482	2.95275
H	2.07298	-1.79913	1.69469
H	1.23178	-0.29492	2.09792
H	2.25245	3.68266	-0.63249
H	1.68613	3.43485	-2.28690
H	2.96228	2.39026	-1.61484
H	-1.41706	1.09253	1.75633
H	-1.03376	1.66193	-0.22033
C	-2.67274	2.58916	0.66687
C	-3.89302	1.84057	0.09461
H	-2.86182	2.98664	1.66866
H	-2.22826	3.37357	0.05496
H	-3.88976	1.84798	-1.00047
H	-4.88345	2.13797	0.44786
H	-3.70814	0.31621	1.64153
H	-4.30856	-1.28956	-0.06254
H	-3.34785	-0.42727	-1.28252
H	-1.33781	-2.80580	1.49351
H	-3.12354	-2.80301	1.49548
H	-2.21801	-1.39663	2.11641
H	-1.23183	-3.24133	-0.86758
H	-2.15070	-2.23160	-1.99975
H	-3.02160	-3.33394	-0.89661

TS10

Geometry optimisation:

B3LYP-D3BJ/631G(d,p)/Lanl2dz-IEFPCM(DMF) Energy = -1050.52152865

Thermal correction to Gibbs Free Energy = 0.362015

Imaginary frequency = -1194.1234

Single-point energy calculation:

B3LYP-D3BJ/6311+G(2d,p)/SDD-IEFPCM(DMF) Energy = -1051.96861621

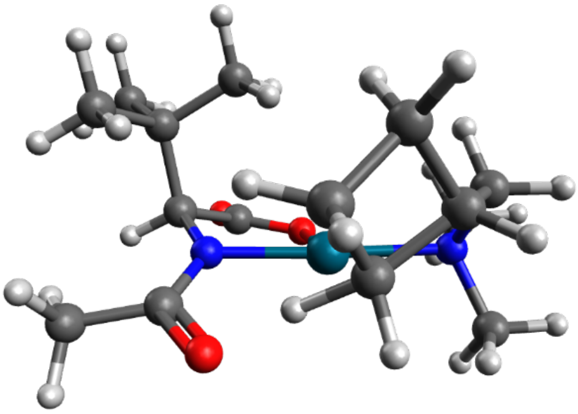

O	2.55643	-2.70599	-0.80310
C	1.76143	-1.78560	-0.61696
C	2.26167	-0.34558	-0.36762
H	3.12610	-0.18355	-1.01889
C	2.74882	-0.16217	1.11605
C	4.01265	-1.00963	1.33828
C	3.09466	1.31640	1.35443
C	1.65957	-0.58692	2.11504
N	1.17196	0.55405	-0.71746
Pd	-0.64570	-0.24603	-0.42446
O	0.46433	-1.96662	-0.58344
N	-2.50614	-1.19959	-0.07136
C	-2.99200	-0.50136	1.15033
C	-2.85551	1.01402	1.00321
C	-1.91704	1.63705	-0.10537
C	-3.43668	-1.00057	-1.20705
C	-2.34787	-2.64730	0.18988
C	1.22181	1.69209	-1.38072
C	2.50966	2.32580	-1.84620
O	0.13520	2.31634	-1.63743
H	4.37553	-0.87371	2.36239
H	4.81309	-0.70530	0.65460
H	3.81567	-2.07029	1.17382
H	3.46836	1.44834	2.37492
H	2.21771	1.95807	1.23344
H	3.87396	1.66121	0.66750
H	2.00655	-0.41715	3.13946
H	1.40907	-1.64708	2.01422
H	0.74208	-0.00935	1.96656
H	-2.36854	-0.85204	1.97663
H	-4.02666	-0.80869	1.35643
H	-3.02005	-1.47502	-2.09645
H	-3.56853	0.06291	-1.39970
H	-4.41175	-1.44889	-0.97706
H	-1.63289	-2.79221	0.99916
H	-1.96292	-3.13240	-0.70655
H	-3.31427	-3.08794	0.46398
H	3.36006	1.64529	-1.83031
H	2.73708	3.17942	-1.20091
H	2.36423	2.70282	-2.86040
H	-2.49095	2.03990	-0.94744
H	-0.84850	1.58971	-0.89137
C	-1.45890	2.67865	0.95836
C	-2.05290	1.79083	2.07457
H	-3.84344	1.46801	0.87718
H	-1.98434	3.63207	0.84669
H	-0.38580	2.87479	1.00581
H	-1.28867	1.15324	2.53040
H	-2.62488	2.28000	2.86683

TS11

Geometry optimisation:

B3LYP-D3BJ/631G(d,p)/Lanl2dz-IEFPCM(DMF) Energy = -1050.51867732

Thermal correction to Gibbs Free Energy = 0.362473

Imaginary frequency = -1303.9832

Single-point energy calculation:

B3LYP-D3BJ/6311+G(2d,p)/SDD-IEFPCM(DMF) Energy = -1051.96544161

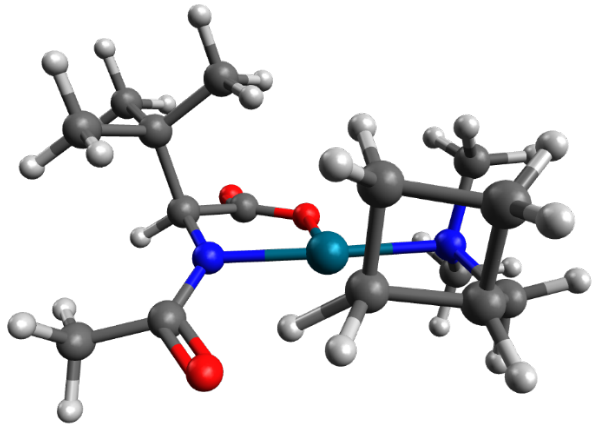

O	2.60023	-2.57897	-0.94197
C	1.81668	-1.66710	-0.68214
C	2.33379	-0.24693	-0.36257
H	3.17228	-0.04990	-1.03775
C	2.88682	-0.16347	1.10720
C	4.15124	-1.03186	1.22046
C	3.25861	1.29367	1.42550
C	1.83828	-0.64372	2.12416
N	1.24016	0.68313	-0.61136
Pd	-0.58321	-0.13410	-0.37658
O	0.51957	-1.84444	-0.62621
N	-2.40738	-1.20289	-0.19508
C	-3.53668	-0.24996	-0.41827
C	-3.30974	1.13440	0.16368
C	-1.83305	1.66248	0.17267
C	-2.45397	-2.27821	-1.21723
C	-2.44289	-1.83144	1.14804
C	1.28626	1.86147	-1.19835
C	2.56614	2.51385	-1.65992
O	0.19979	2.51234	-1.38541
H	4.56360	-0.95509	2.23194
H	4.92125	-0.69584	0.51698
H	3.93682	-2.08028	1.00754
H	3.66783	1.35701	2.43891
H	2.38629	1.95074	1.37476
H	4.01929	1.67267	0.73579
H	2.24225	-0.56787	3.13892
H	1.55285	-1.68558	1.95140
H	0.93288	-0.03254	2.07476
H	3.41345	1.83070	-1.70074
H	2.81363	3.33223	-0.97757
H	2.39773	2.94357	-2.64945
H	-0.79781	1.77656	-0.71039
H	-3.99625	1.80036	-0.36804
H	-1.59828	-2.51623	1.23178
H	-3.37956	-2.38722	1.28207
H	-2.35986	-1.07572	1.92472
H	-2.39785	-1.83256	-2.21089
H	-3.38684	-2.84815	-1.12392
H	-1.59788	-2.93590	-1.07314
H	-4.46854	-0.69946	-0.04778
H	-3.62771	-0.14713	-1.50227
H	-1.86993	2.71172	-0.14351
C	-1.82460	1.64129	1.73166
C	-3.35144	1.35278	1.70572
H	-1.51985	2.57524	2.21130
H	-1.24089	0.83207	2.17624
H	-3.70143	0.51088	2.30857
H	-3.94894	2.22773	1.97260

7. NMR spectra

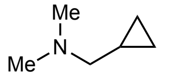

1a

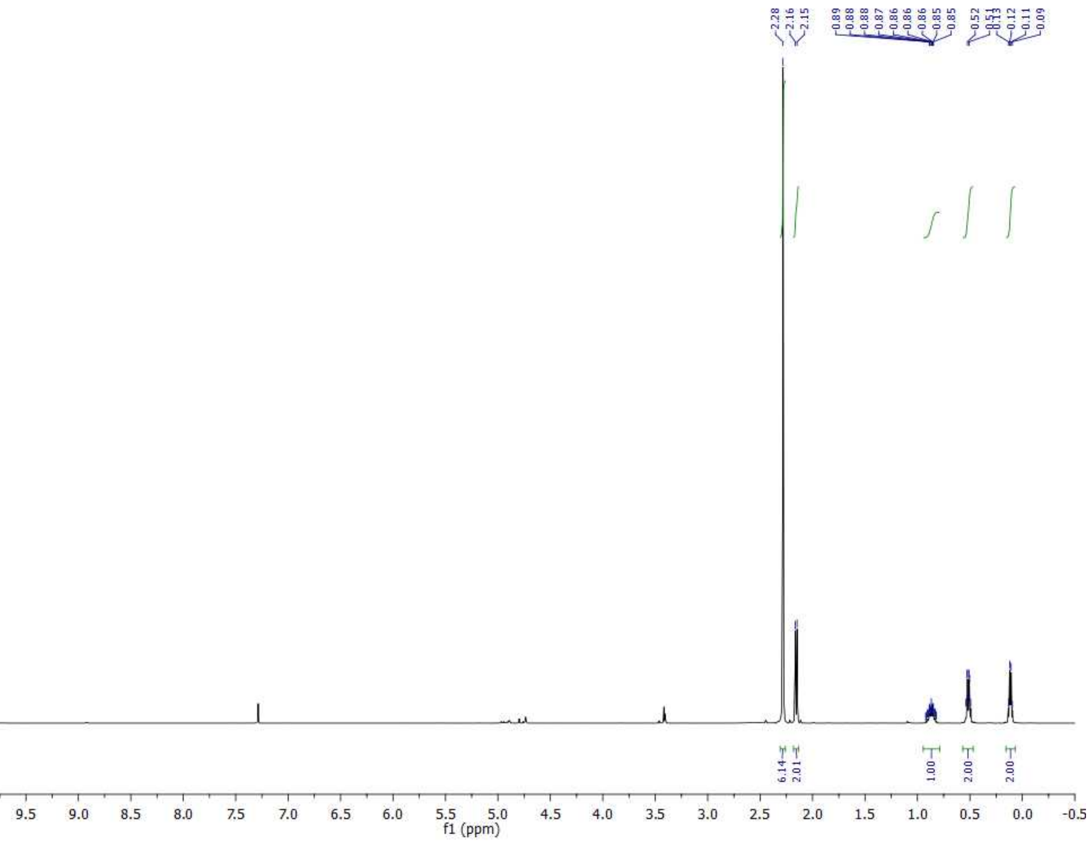

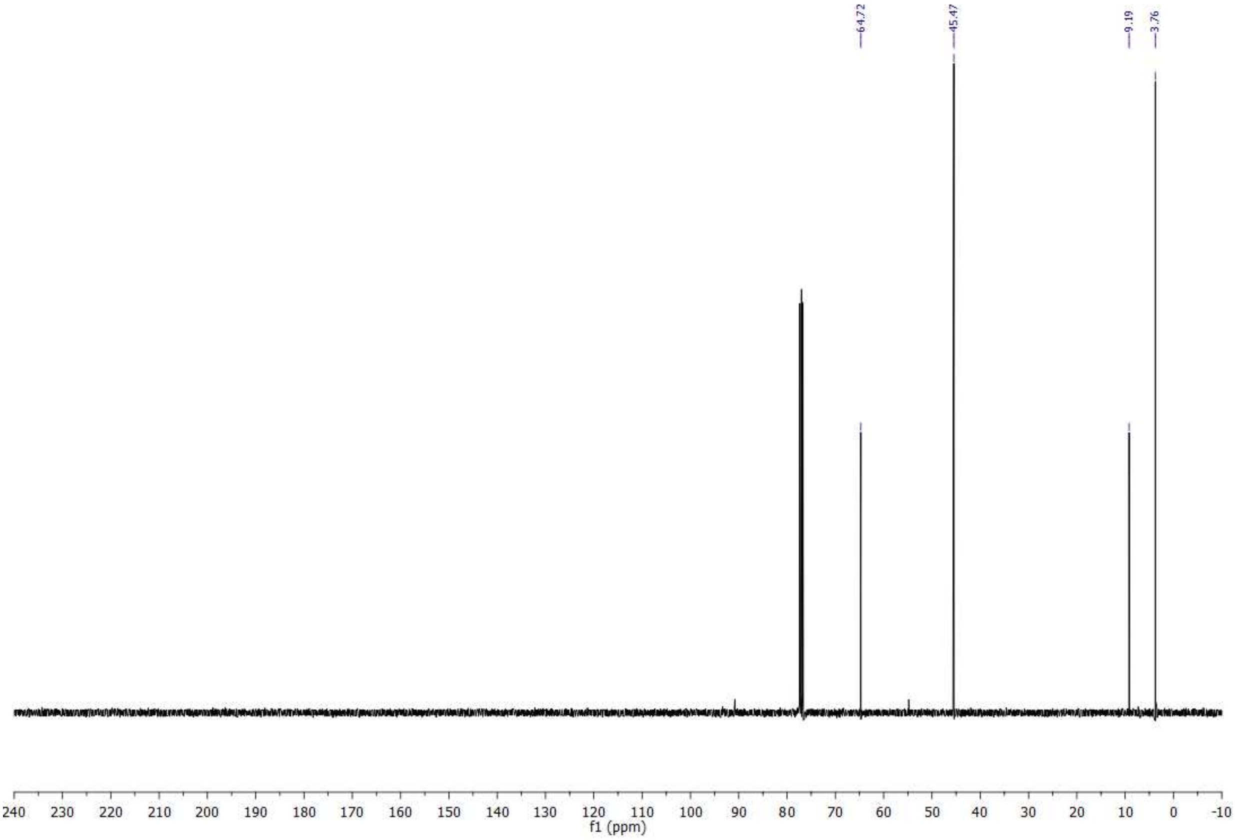

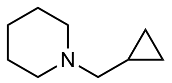
N

1b

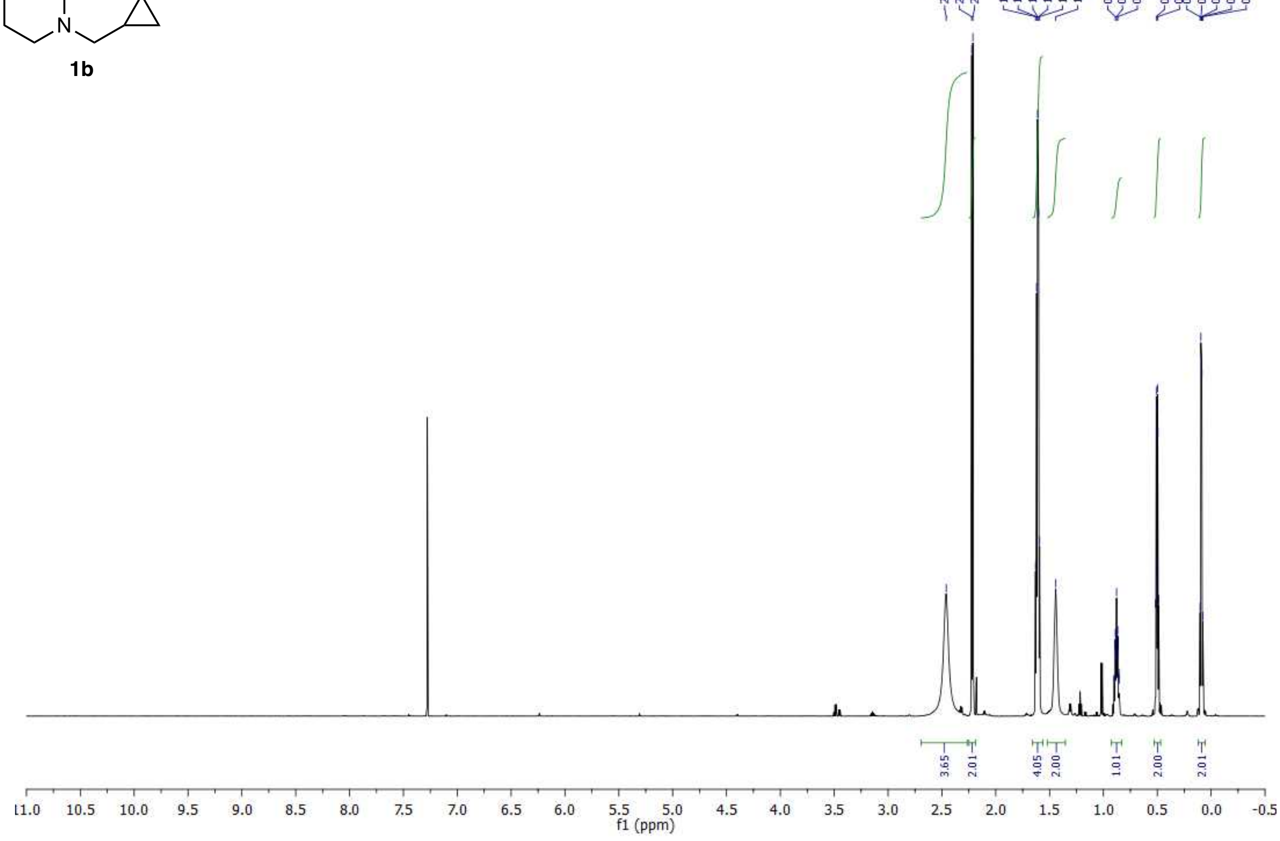
2.46
2.22
2.21
1.63
1.62
1.61
1.60
1.59
1.44
0.89
0.88
0.87
0.51
0.50
0.10
0.09
0.09
0.09
0.08
3.65
2.01
4.05
2.00
1.01
2.00
2.01
11.0 10.5 10.0 9.5 9.0 8.5 8.0 7.5 7.0 6.5 6.0 5.5 5.0 4.5 4.0 3.5 3.0 2.5 2.0 1.5 1.0 0.5 0.0 -0.5
f1 (ppm)

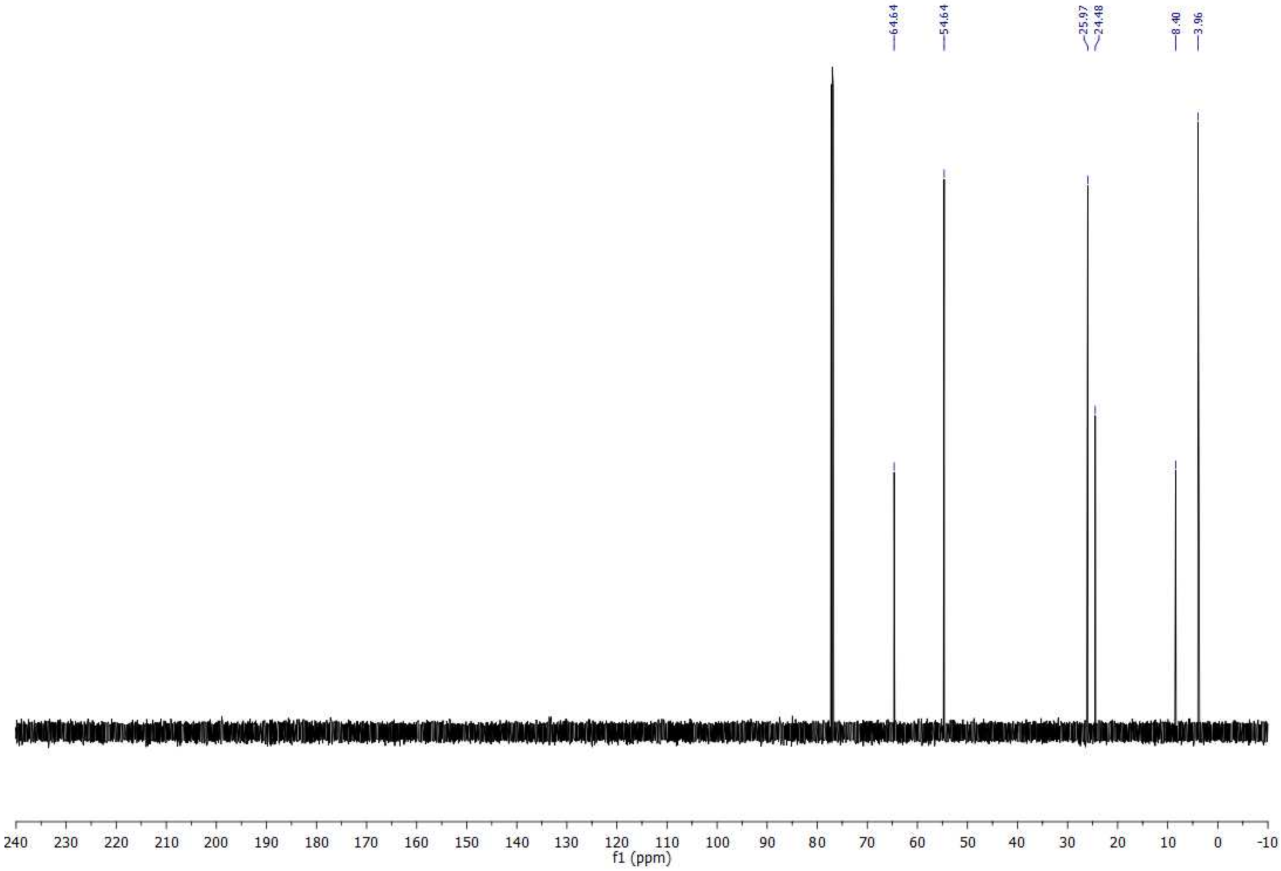
64.64
54.64
25.97
24.48
8.40
3.96
240 230 220 210 200 190 180 170 160 150 140 130 120 110 100 90 80 70 60 50 40 30 20 10 0 -10
f1 (ppm)

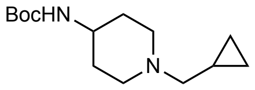
BocHN
N

1c

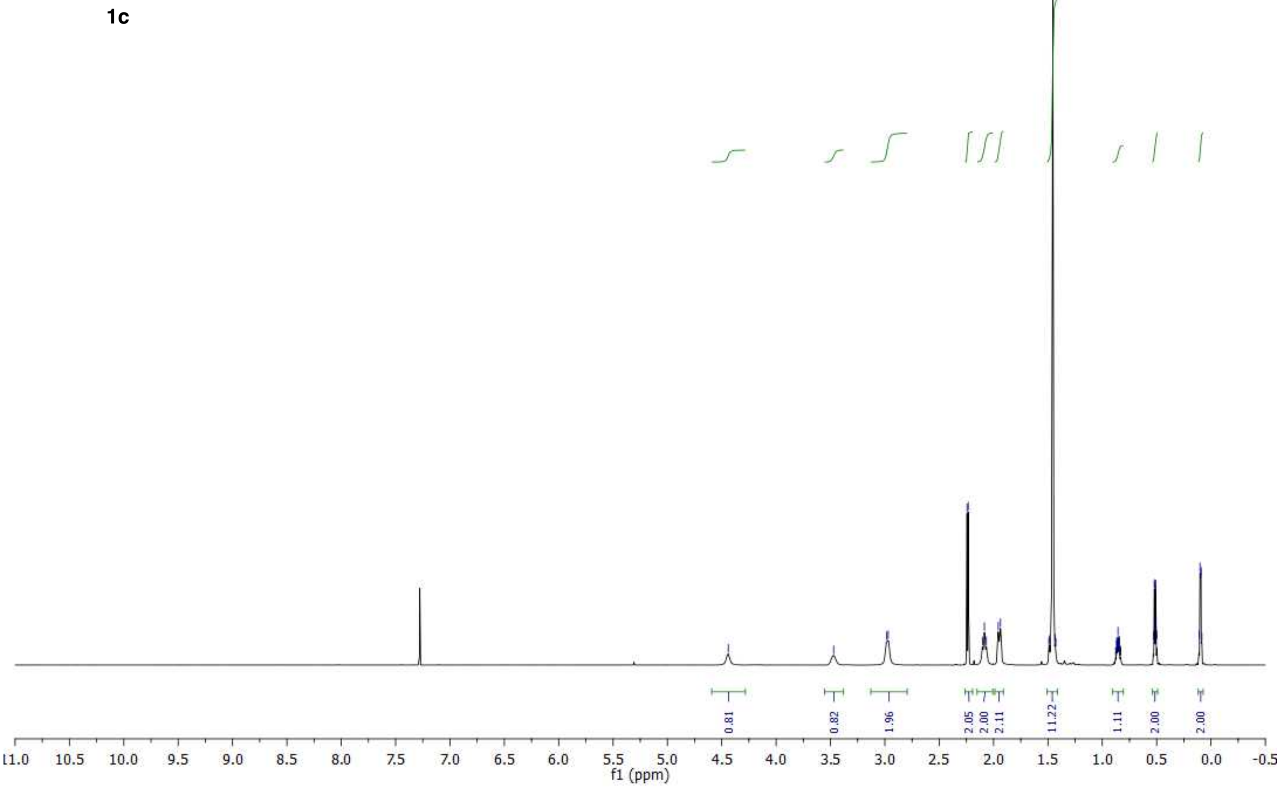
4.44
3.47
2.98
2.97
2.24
2.23
2.10
2.08
2.07
1.96
1.94
1.49
1.49
1.46
1.43
0.87
0.86
0.84
0.52
0.51
0.11
0.10
0.09
0.09
0.08
0.81
0.82
1.96
2.05
2.00
2.11
11.22
1.11
2.00
2.00
f1 (ppm)

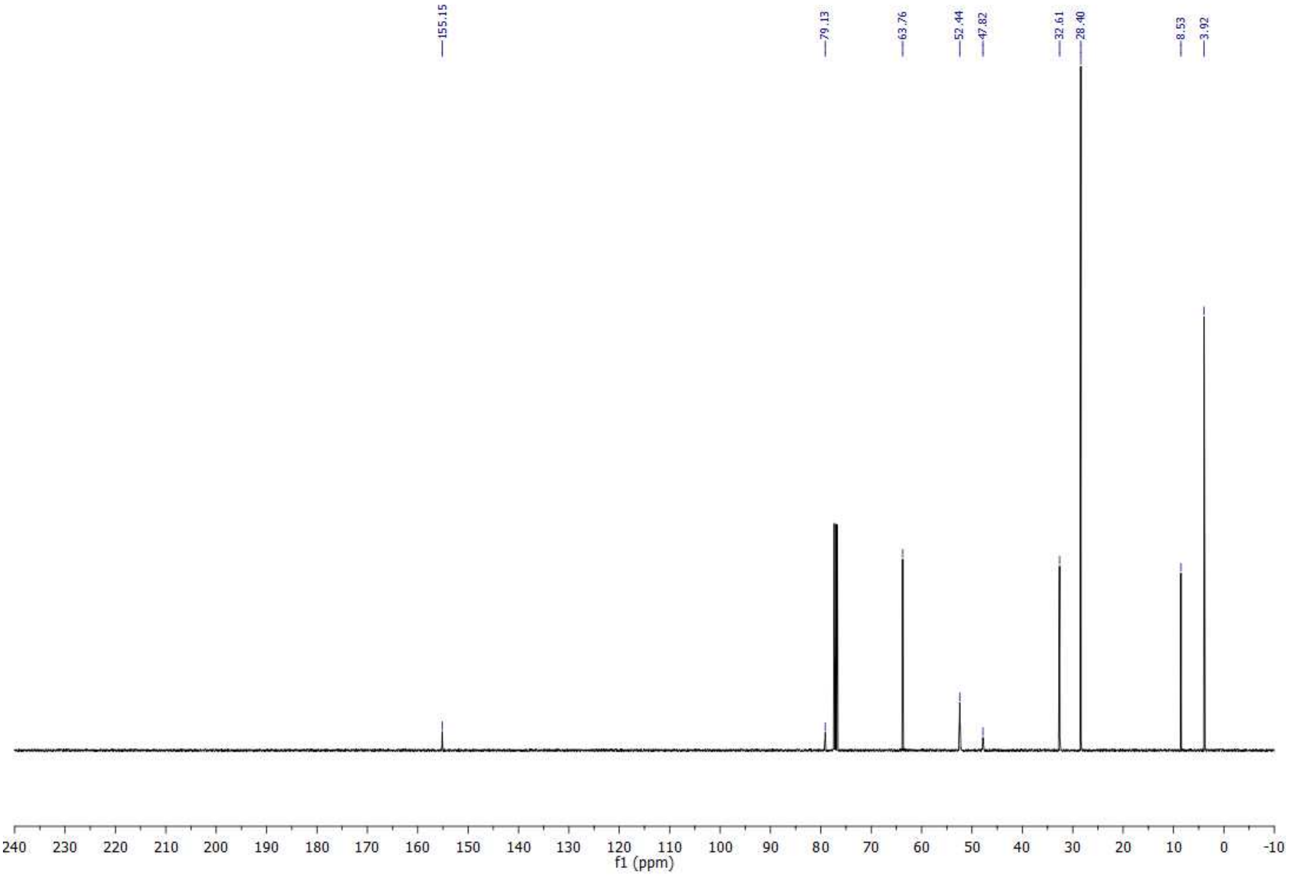
155.15
79.13
63.76
52.44
47.82
32.61
28.40
8.53
3.92
f1 (ppm)

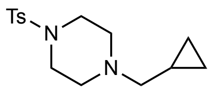

Ts
N
N

1d

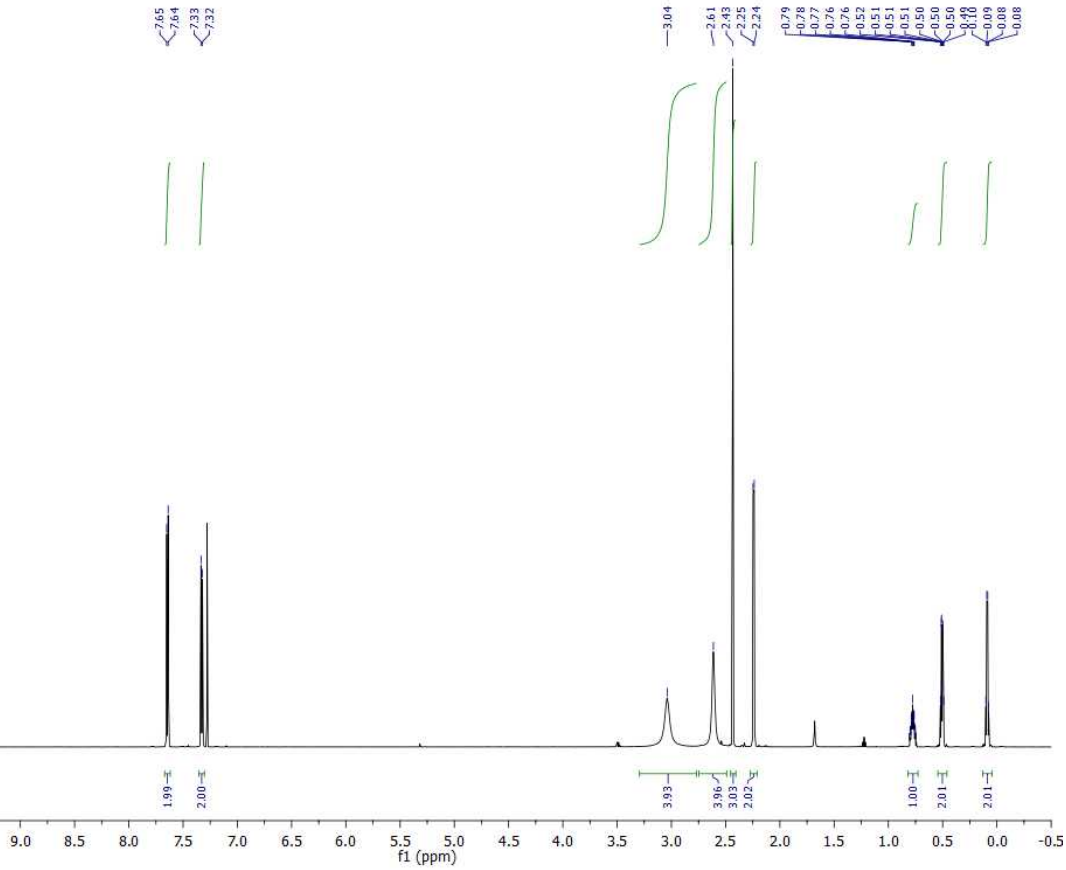

7.65
7.64
7.33
7.32
3.04
2.61
2.43
2.25
2.24
1.99
2.00
3.93
3.96
3.03
2.02
1.00
2.01
2.01
f1 (ppm)

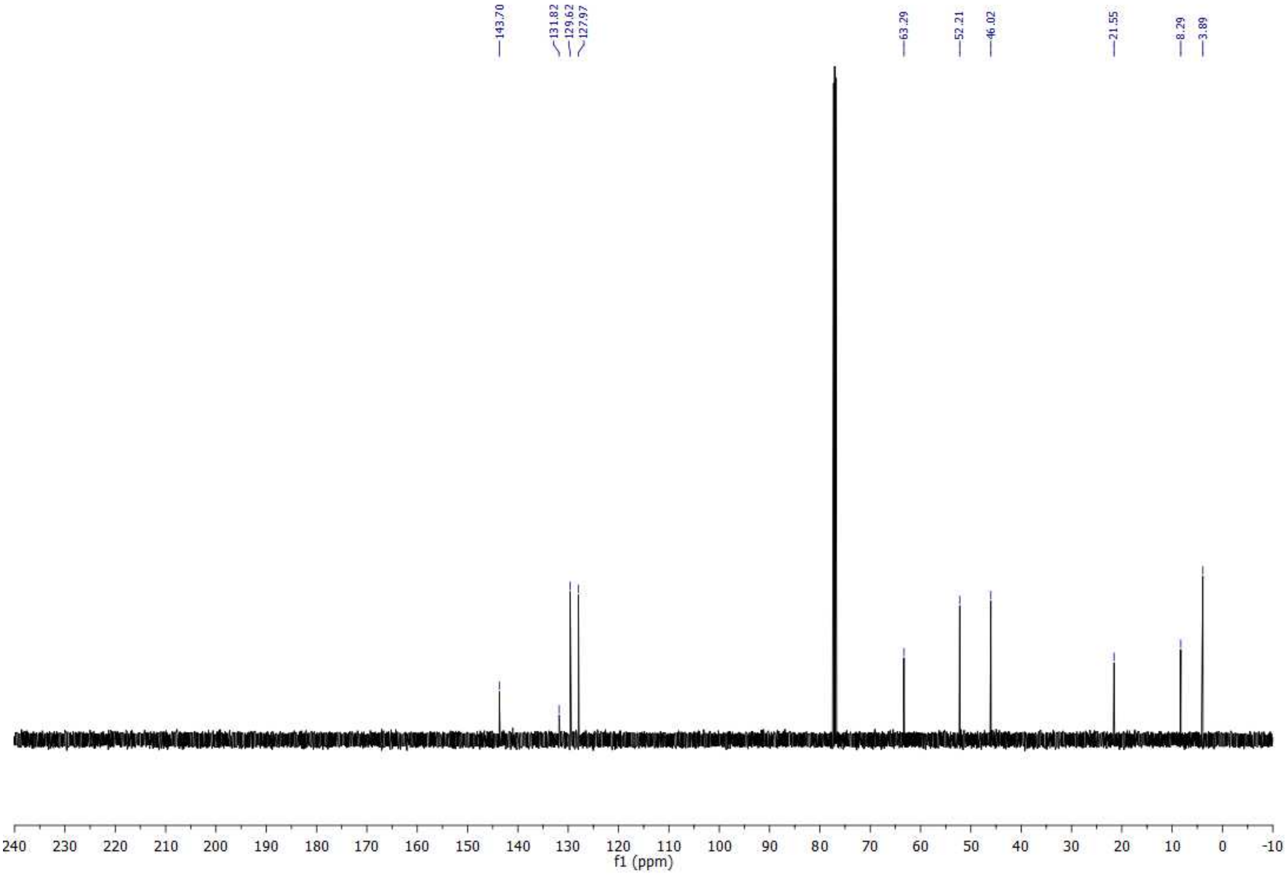

143.70
131.82
129.62
127.97
63.29
52.21
46.02
21.55
8.29
3.89
f1 (ppm)

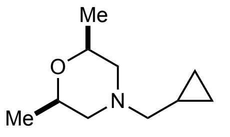
Me
O
N
Me

1e

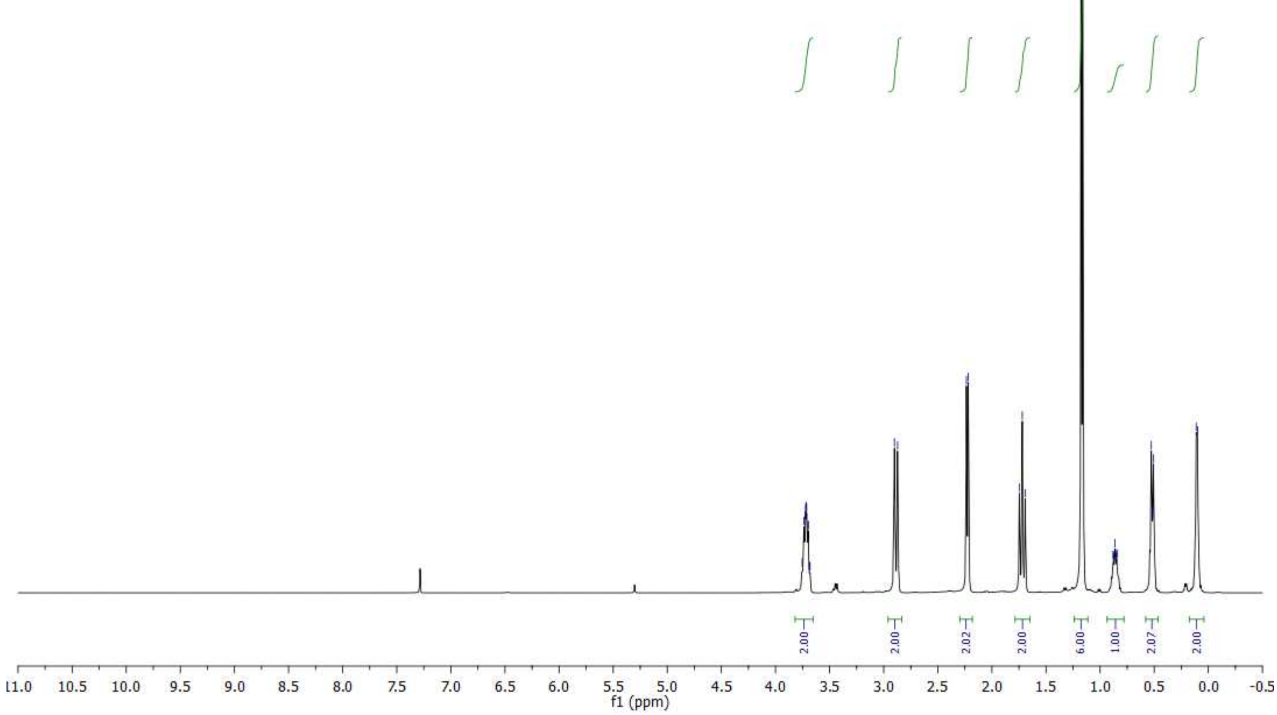
3.75
3.74
3.73
3.72
3.72
3.71
3.70
3.70
3.69
3.68
2.90
2.87
2.24
2.22
1.75
1.72
1.69
1.18
1.16
0.86
0.52
0.51
0.11
0.10
2.00
2.00
2.02
2.00
6.00
1.00
2.07
2.00
f1 (ppm)

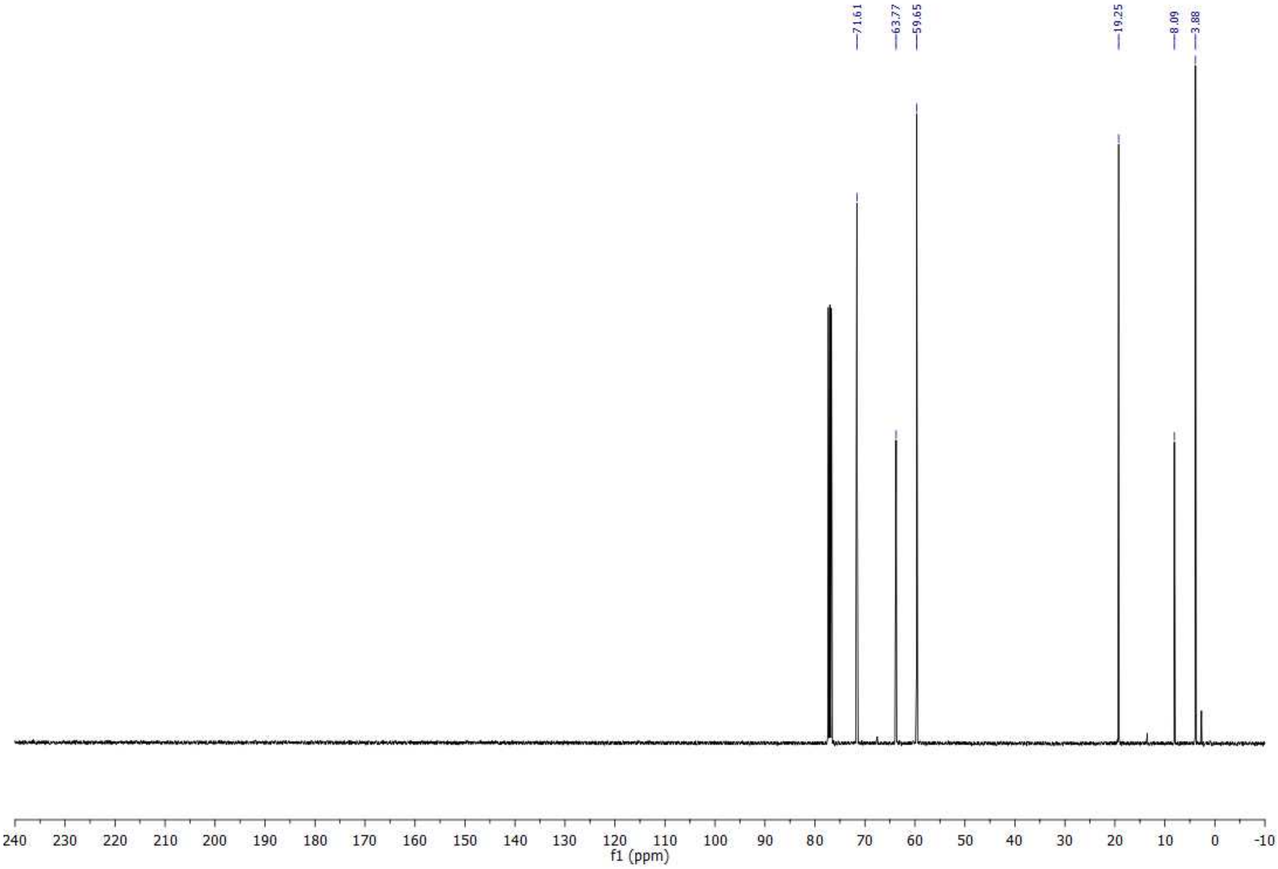
71.61
63.77
59.65
19.25
8.09
3.88
f1 (ppm)

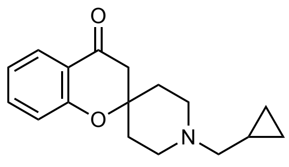

1f

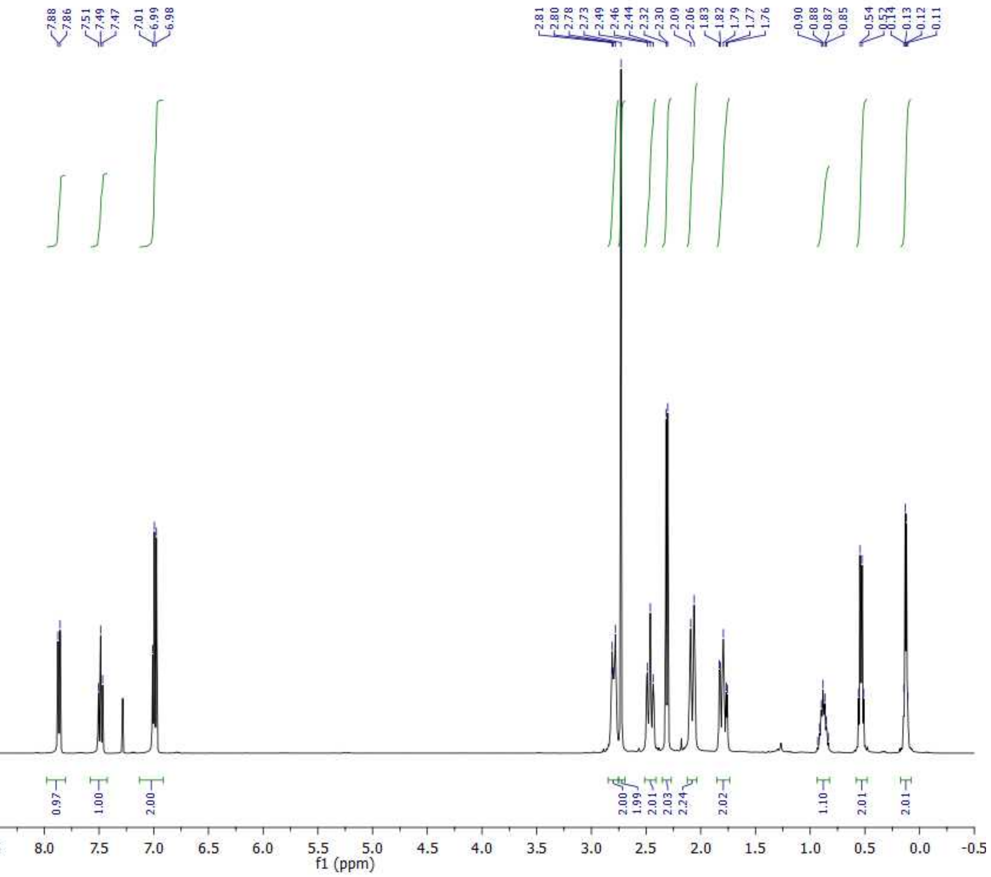
7.88
7.86
7.51
7.49
7.47
7.01
6.99
6.98
2.81
2.80
2.78
2.73
2.49
2.46
2.44
2.32
2.30
2.09
2.06
1.83
1.82
1.79
1.77
1.76
0.90
0.88
0.87
0.85
0.54
0.52
0.14
0.13
0.12
0.11
0.97
1.00
2.00
2.00
1.99
2.01
2.03
2.24
2.02
1.10
2.01
2.01
f1 (ppm)

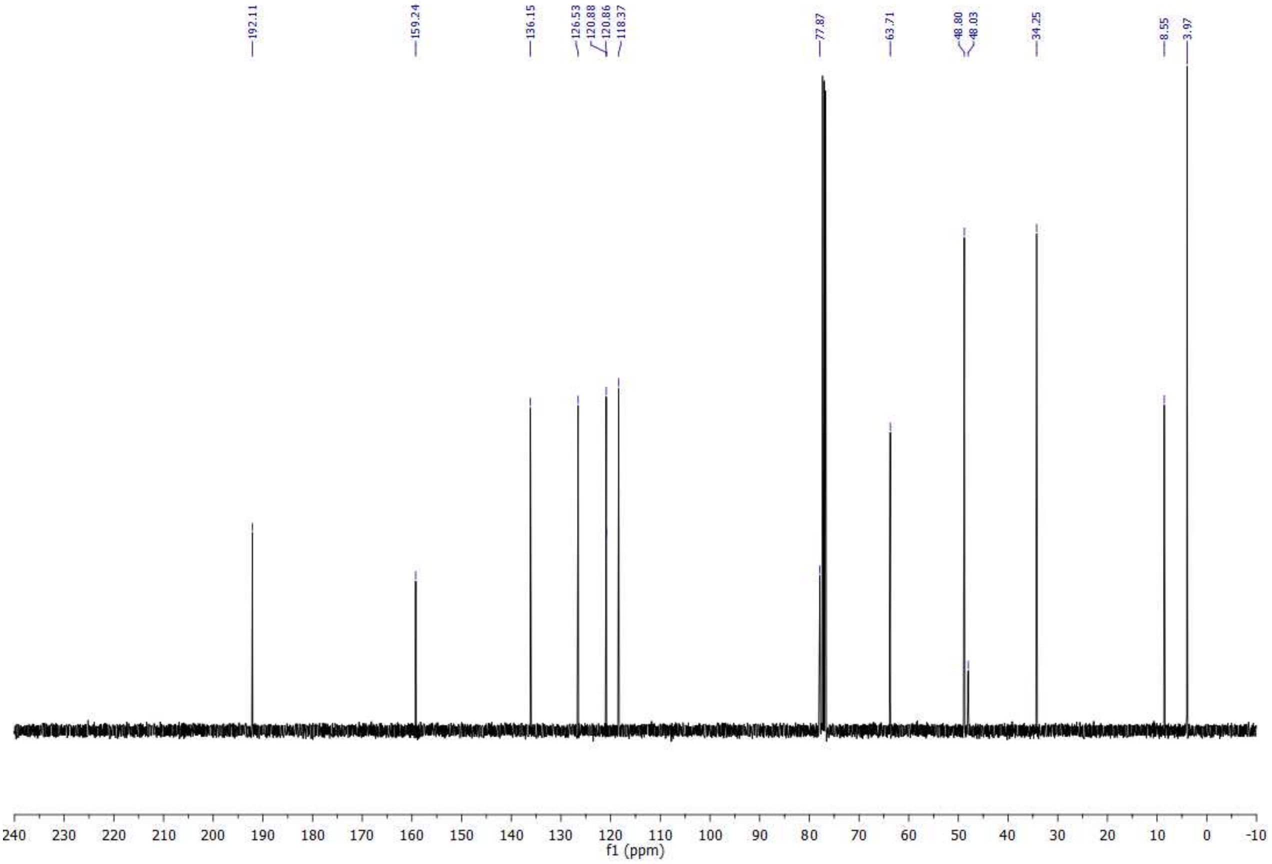
192.11
159.24
136.15
126.53
120.88
120.86
118.37
77.87
63.71
48.80
48.03
34.25
8.55
3.97
f1 (ppm)

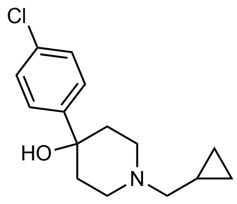
Cl
HO
N

1g

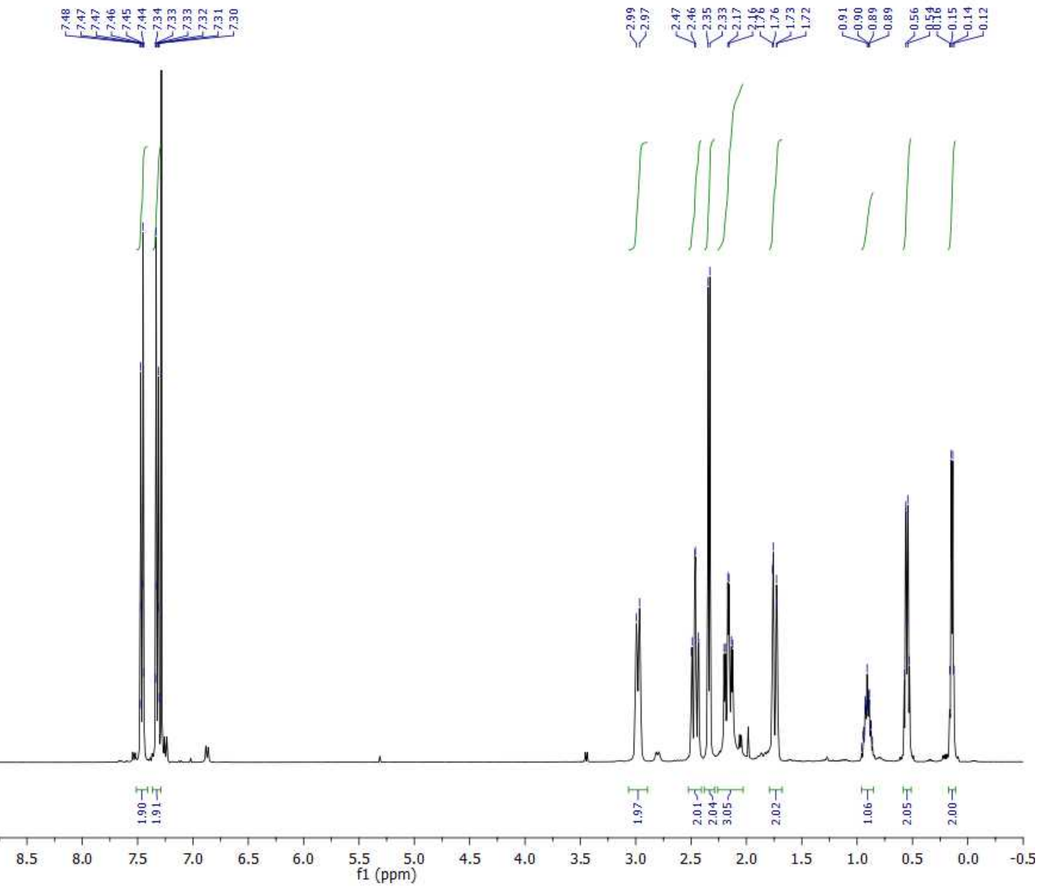
7.48 7.47 7.47 7.46 7.45 7.44 7.34 7.33 7.33 7.32 7.31 7.30
2.99 2.97
2.47 2.46 2.35 2.33 2.17 2.16 1.76 1.73 1.72
0.91 0.90 0.89 0.89
0.56 0.15 0.14 0.12
1.90 1.91 1.97 2.01 2.04 3.05 2.02 1.06 2.05 2.00
f1 (ppm)

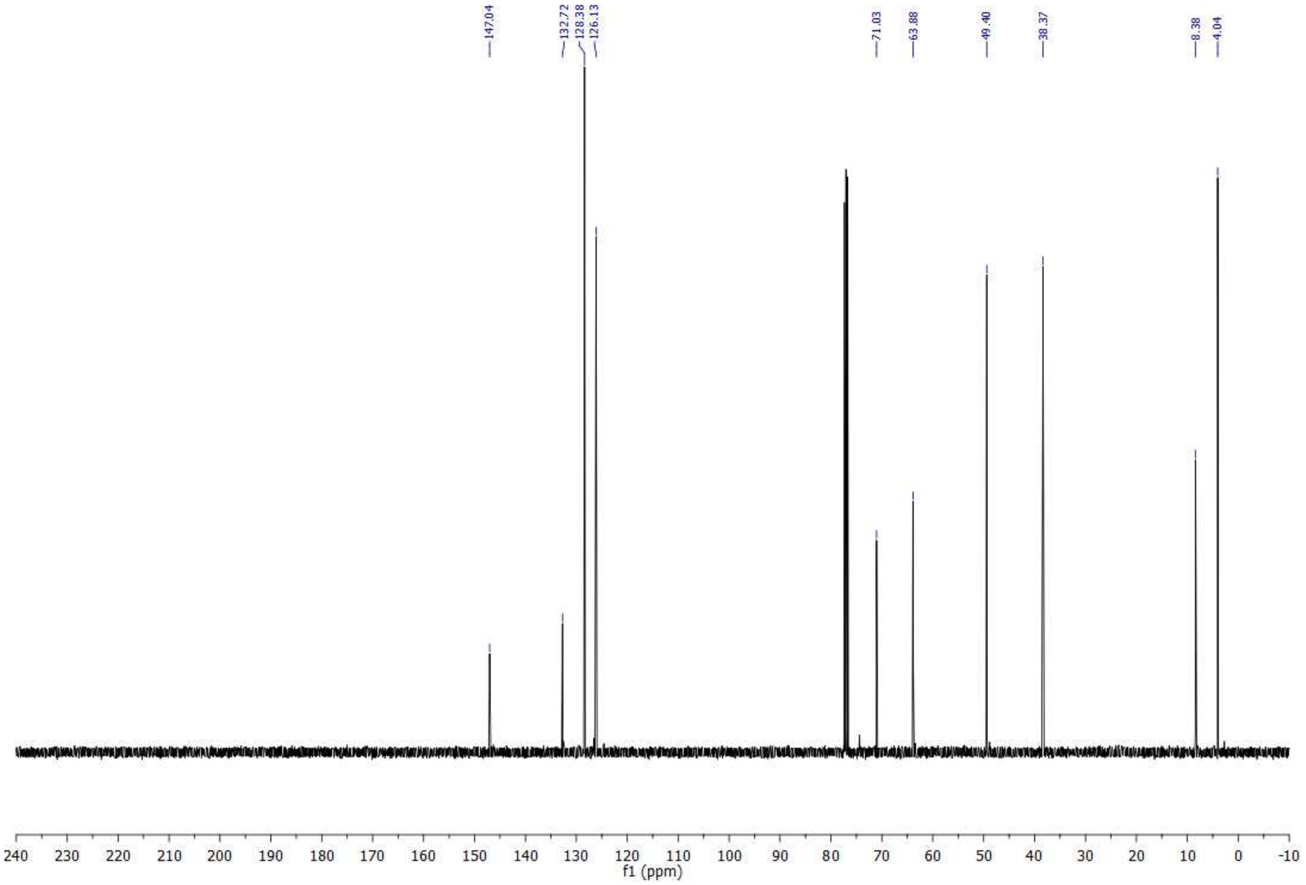
147.04
132.72 128.38 126.13
71.03 63.88
49.40
38.37
8.38 4.04
f1 (ppm)

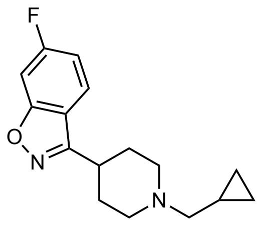

1h

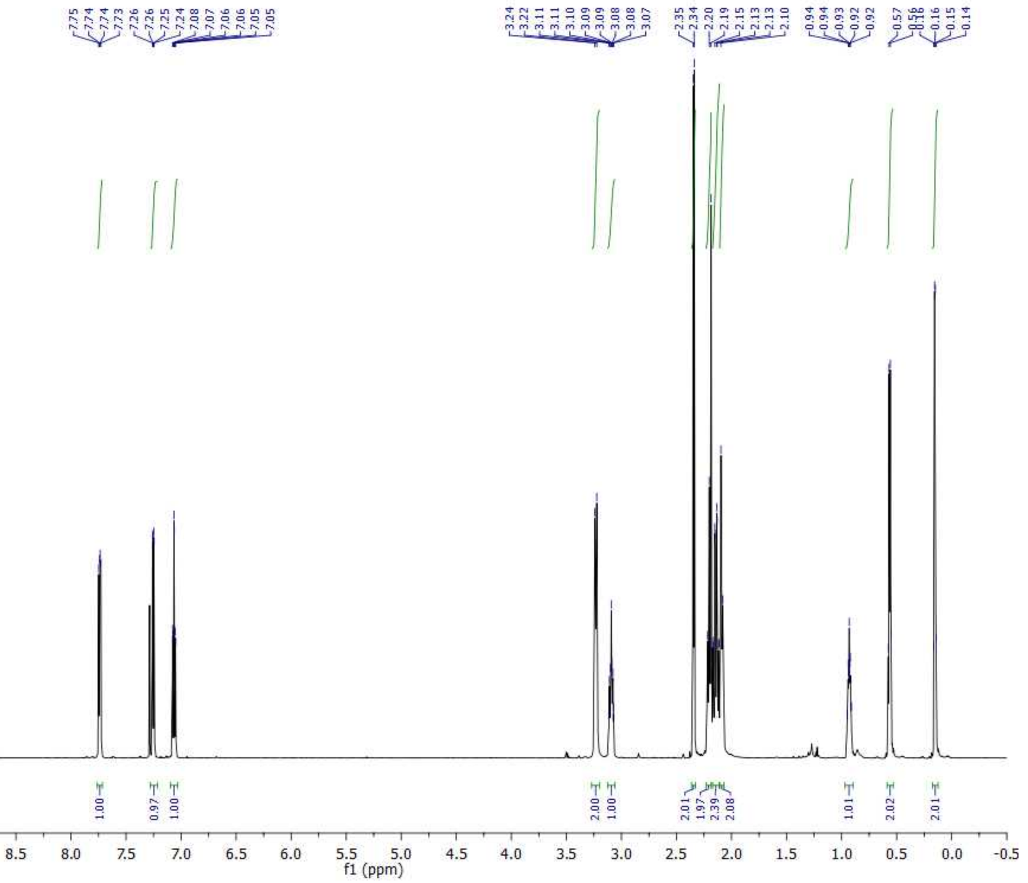

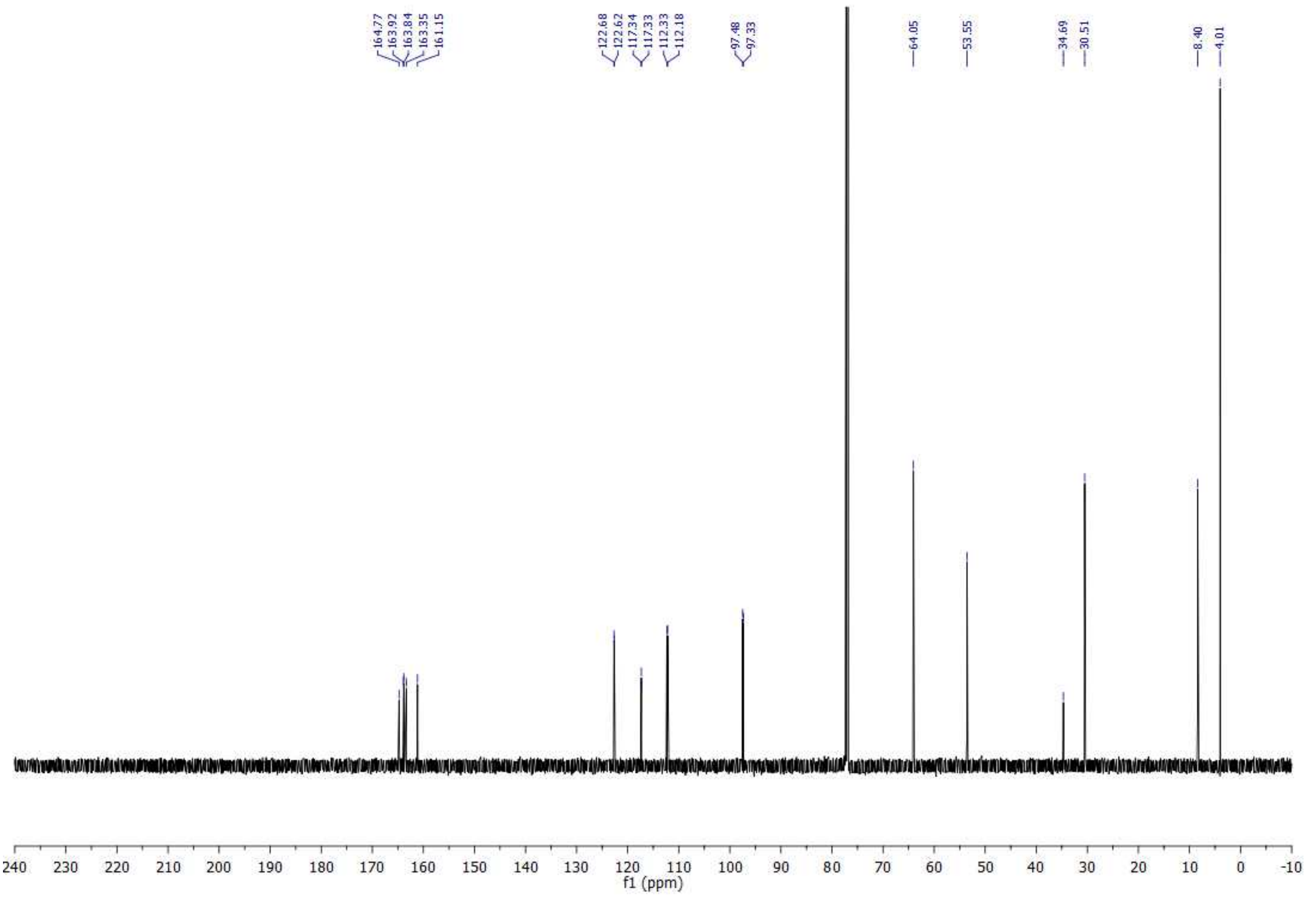

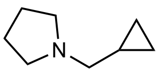
N

1i

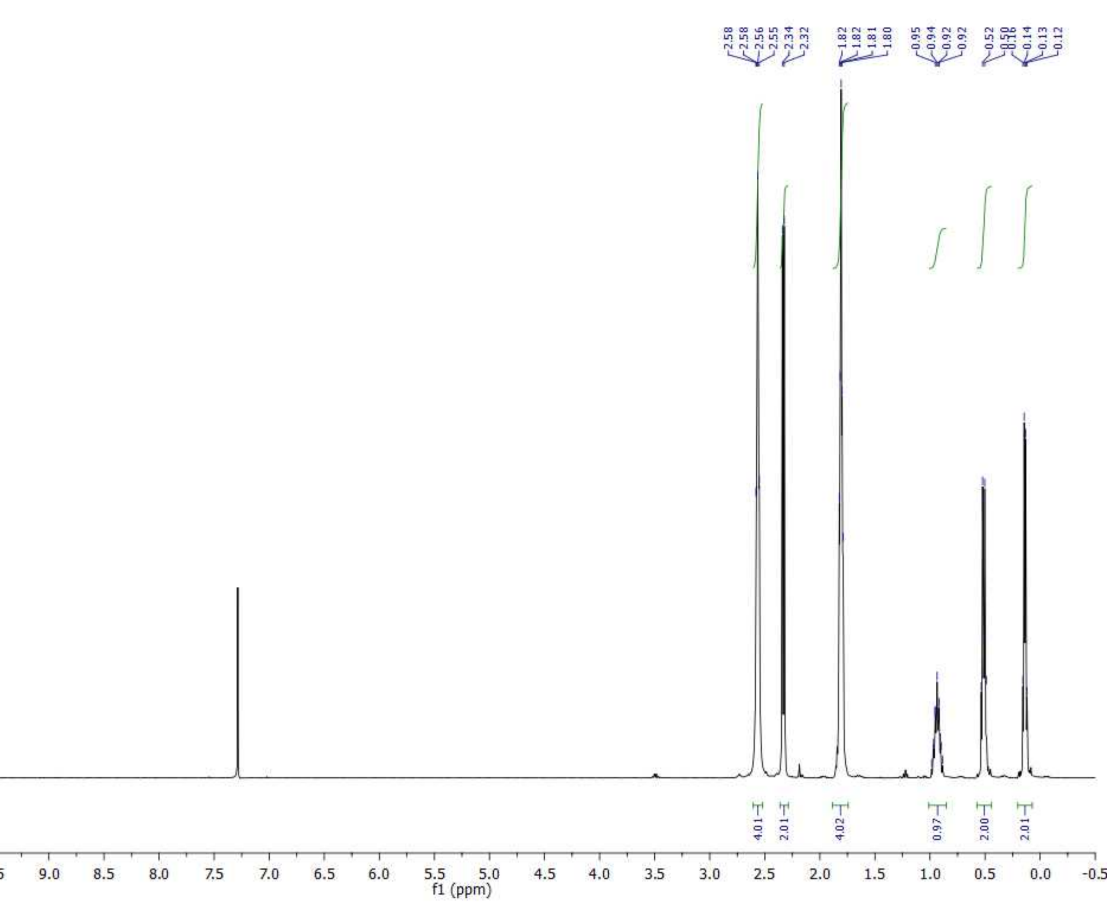
2.58
2.58
2.56
2.55
2.34
2.32
1.82
1.82
1.81
1.80
0.95
0.94
0.92
0.92
0.52
0.50
0.14
0.13
0.12
4.01
2.01
4.02
0.97
2.00
2.01
f1 (ppm)

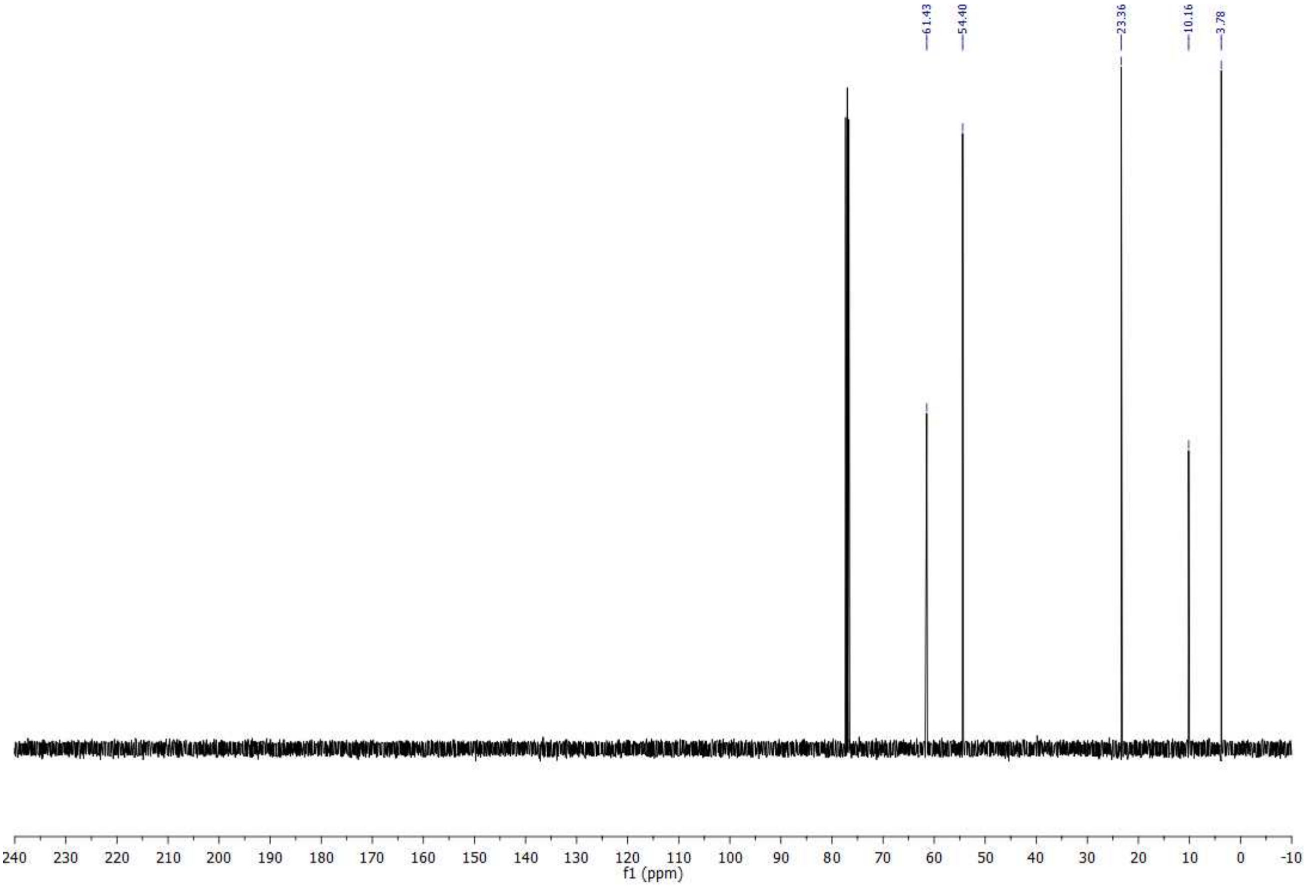
61.43
54.40
23.36
10.16
3.78
f1 (ppm)

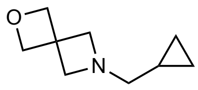

1k

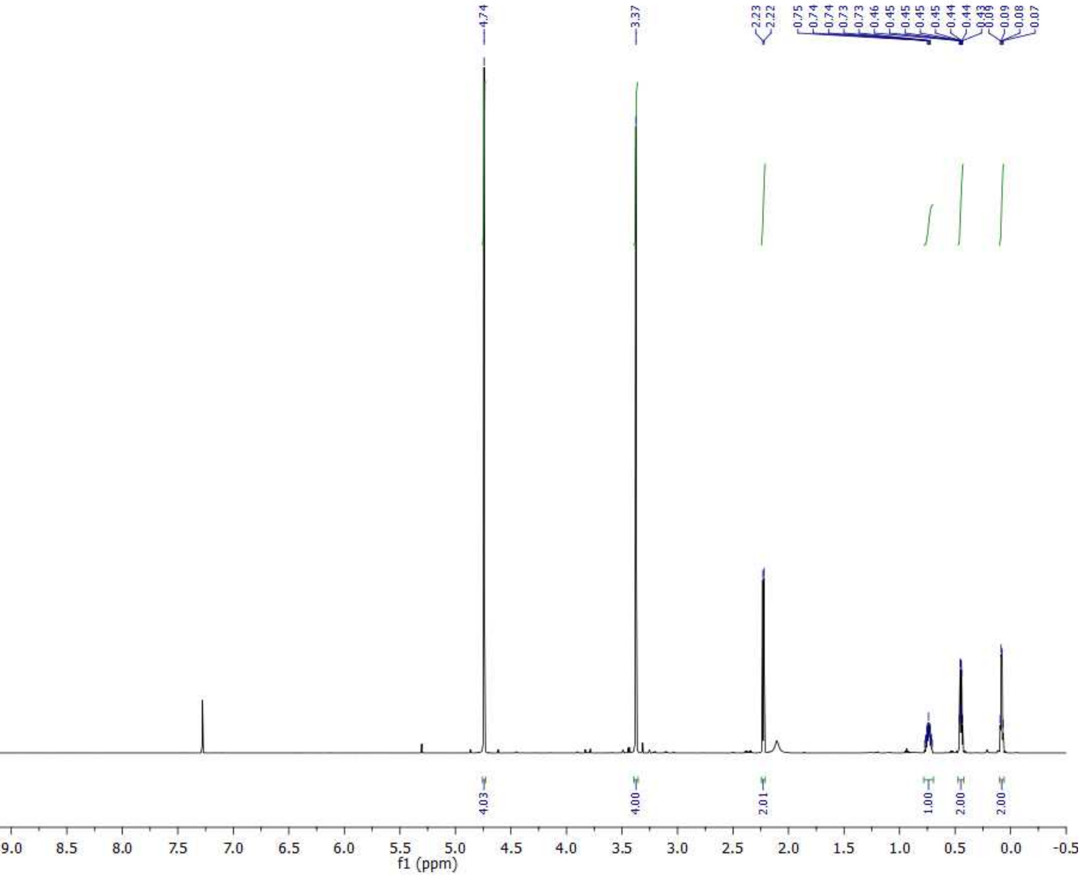

4.74
3.37
2.23
2.22
4.03
4.00
2.01
1.00
2.00
2.00
f1 (ppm)

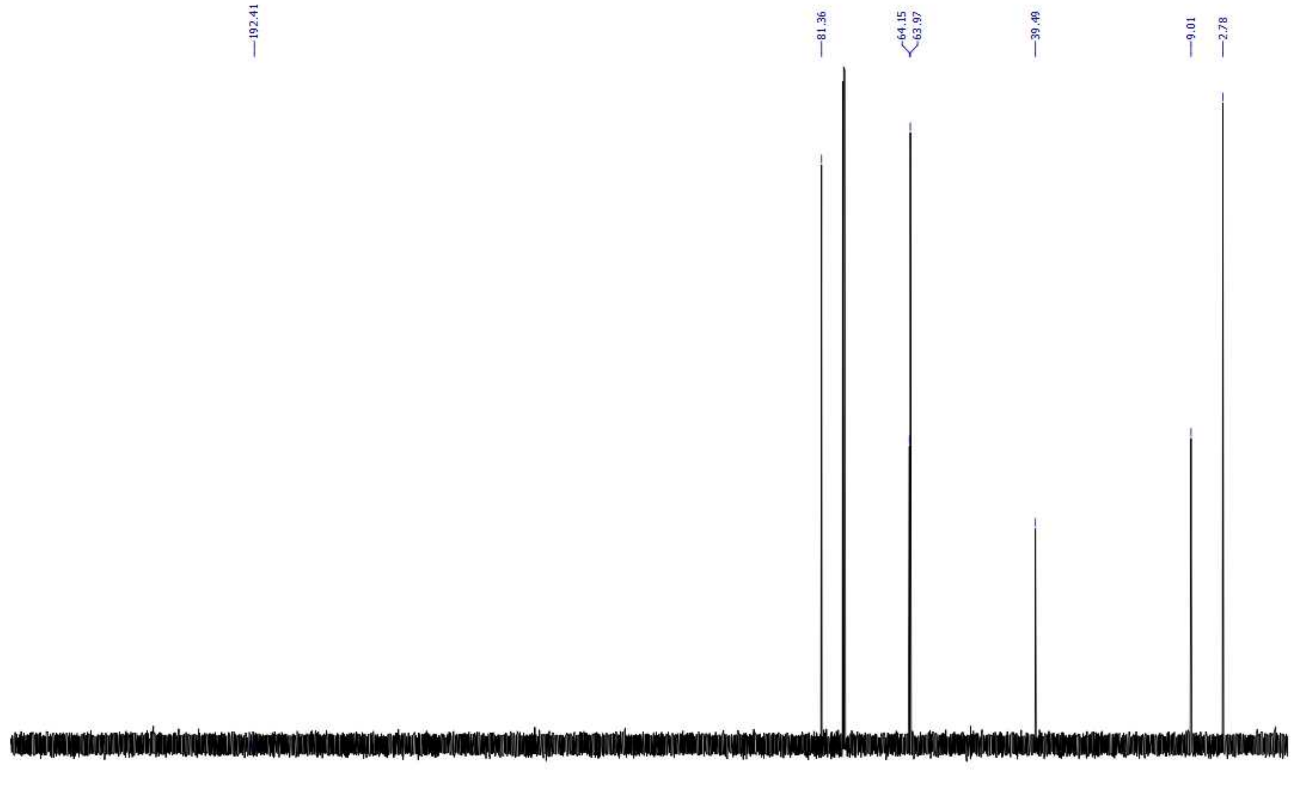

192.41
81.36
64.15
63.97
39.49
9.01
2.78
f1 (ppm)

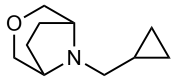

1l

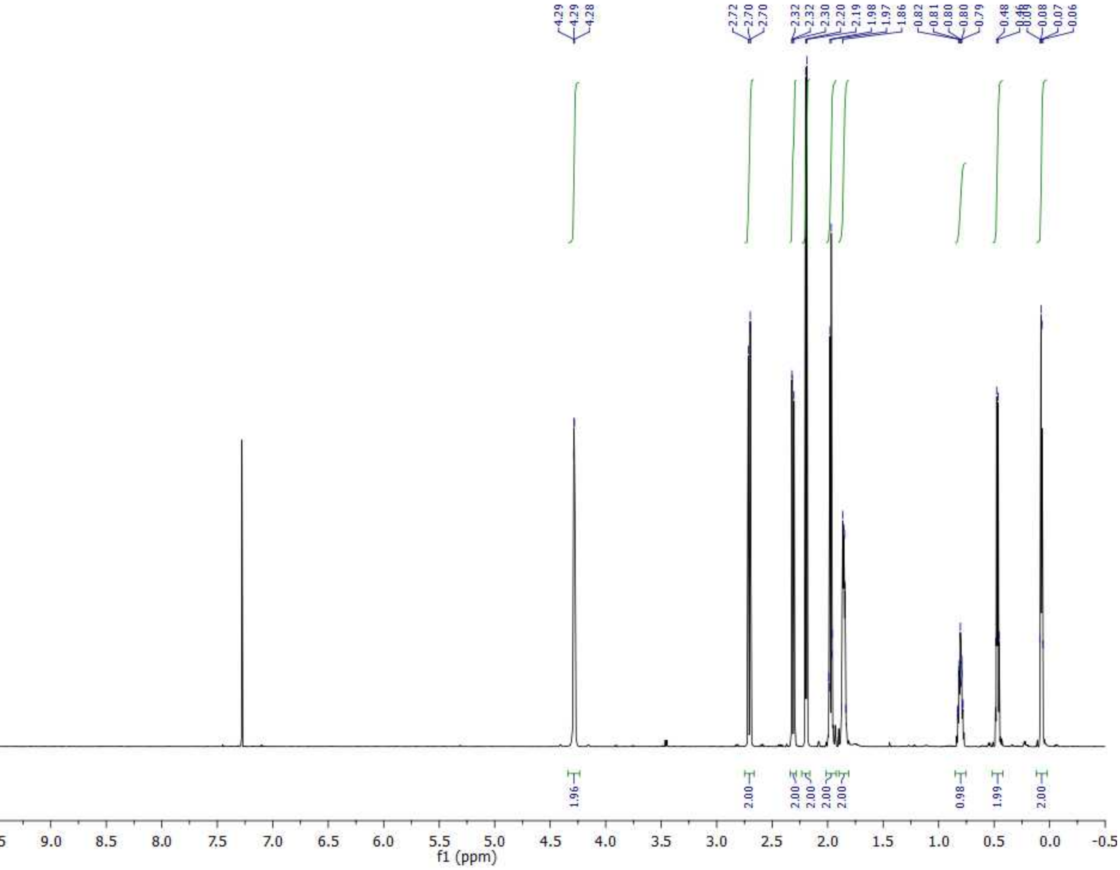
4.29
4.29
4.28
2.72
2.70
2.70
2.32
2.32
2.30
2.20
2.19
1.98
1.97
1.86
0.82
0.81
0.80
0.80
0.79
0.48
0.08
0.07
0.06
1.96
2.00
2.00
2.00
2.00
2.00
0.98
1.99
2.00
11.0 10.5 10.0 9.5 9.0 8.5 8.0 7.5 7.0 6.5 6.0 5.5 5.0 4.5 4.0 3.5 3.0 2.5 2.0 1.5 1.0 0.5 0.0 -0.5
f1 (ppm)

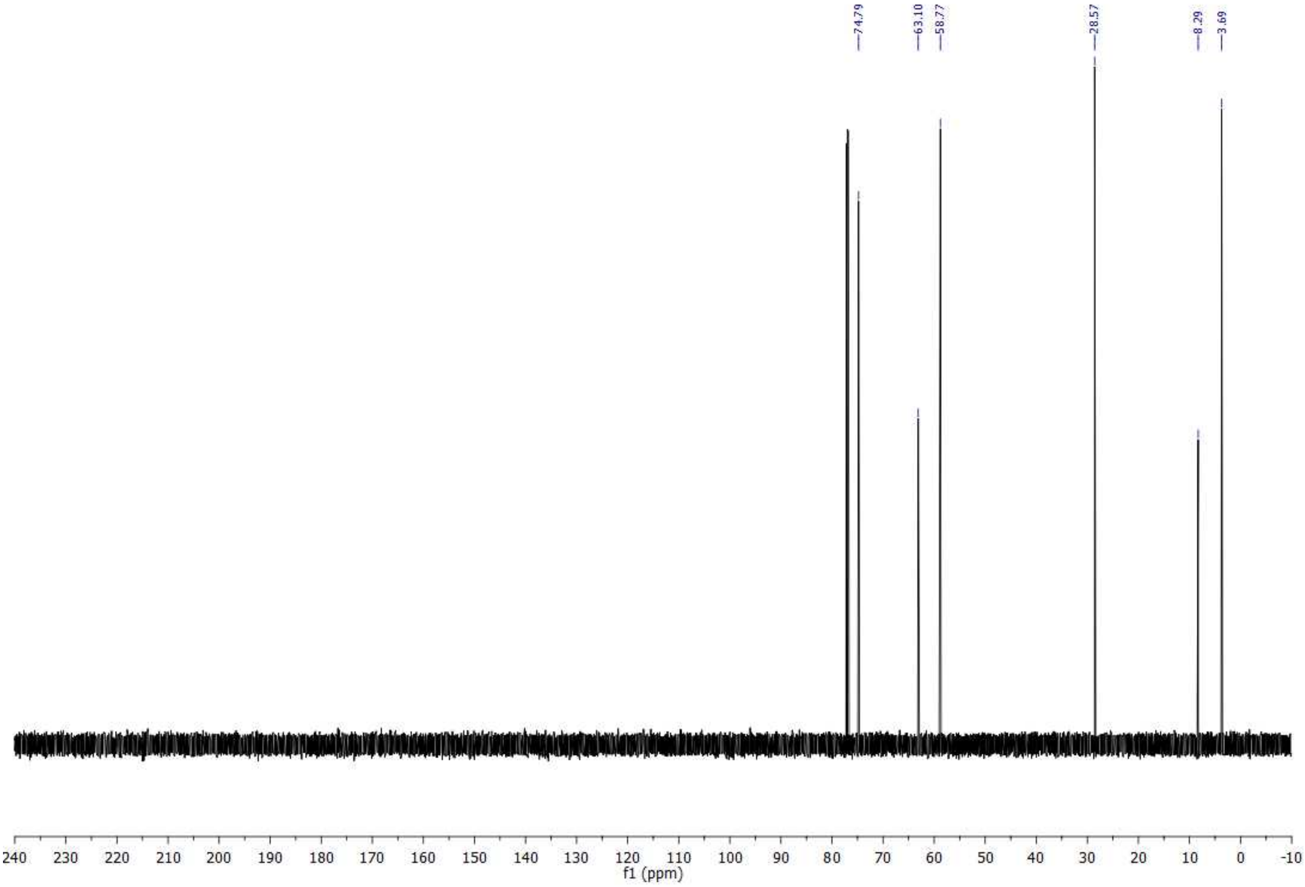
74.79
63.10
58.77
28.57
8.29
3.69
240 230 220 210 200 190 180 170 160 150 140 130 120 110 100 90 80 70 60 50 40 30 20 10 0 -10
f1 (ppm)

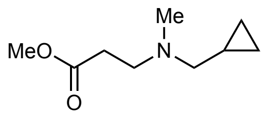
Me
MeO
N
O

1m

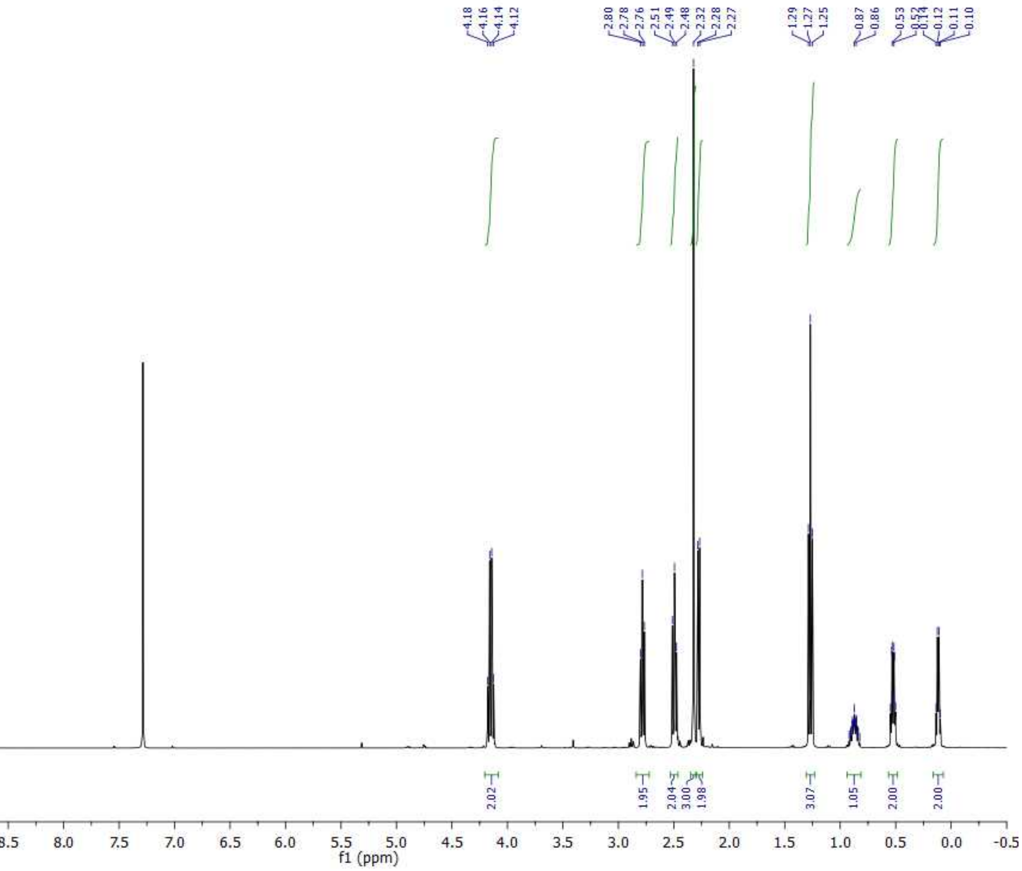
f1 (ppm)

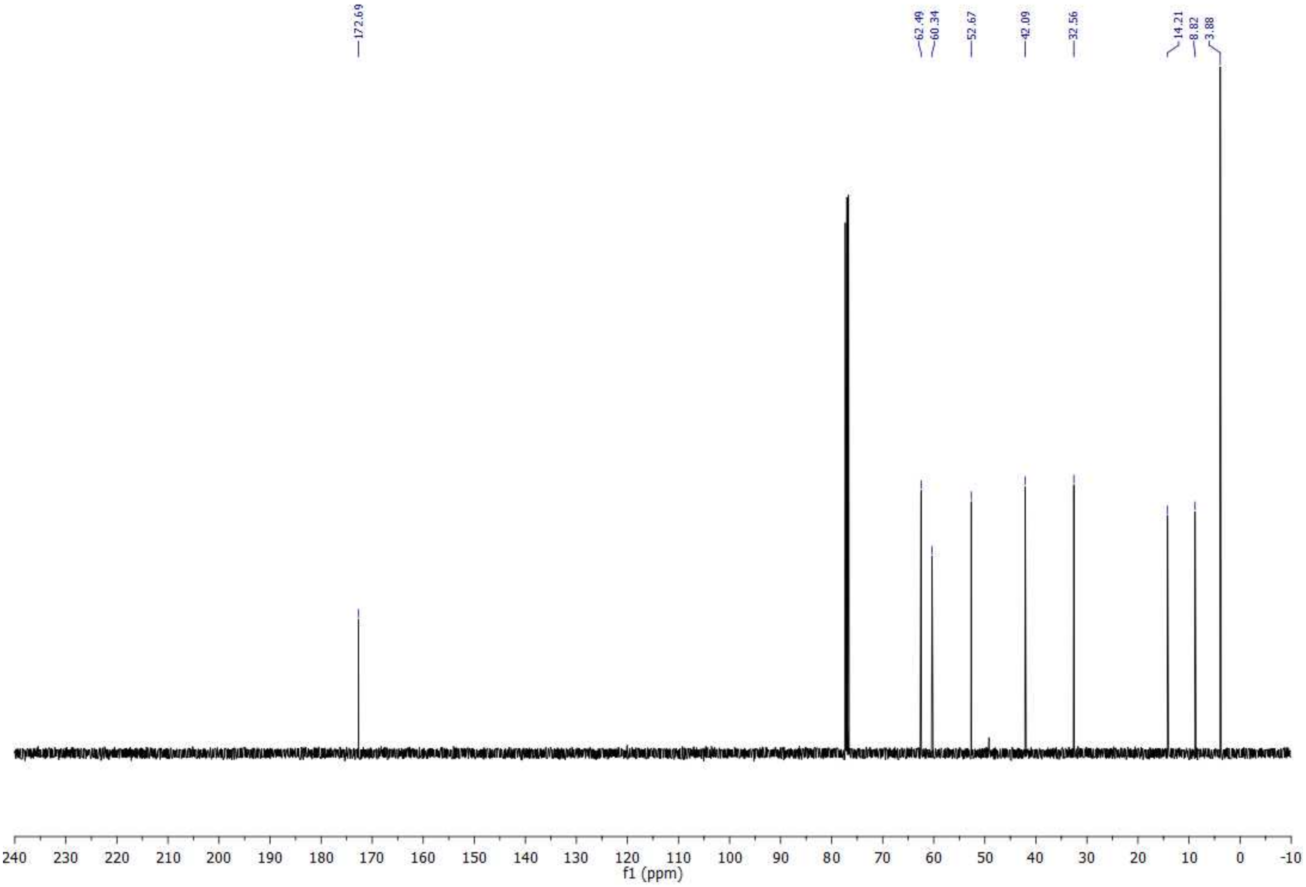
172.69
62.49
60.34
52.67
42.09
32.56
14.21
8.82
3.88
f1 (ppm)

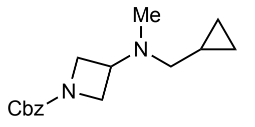

1n

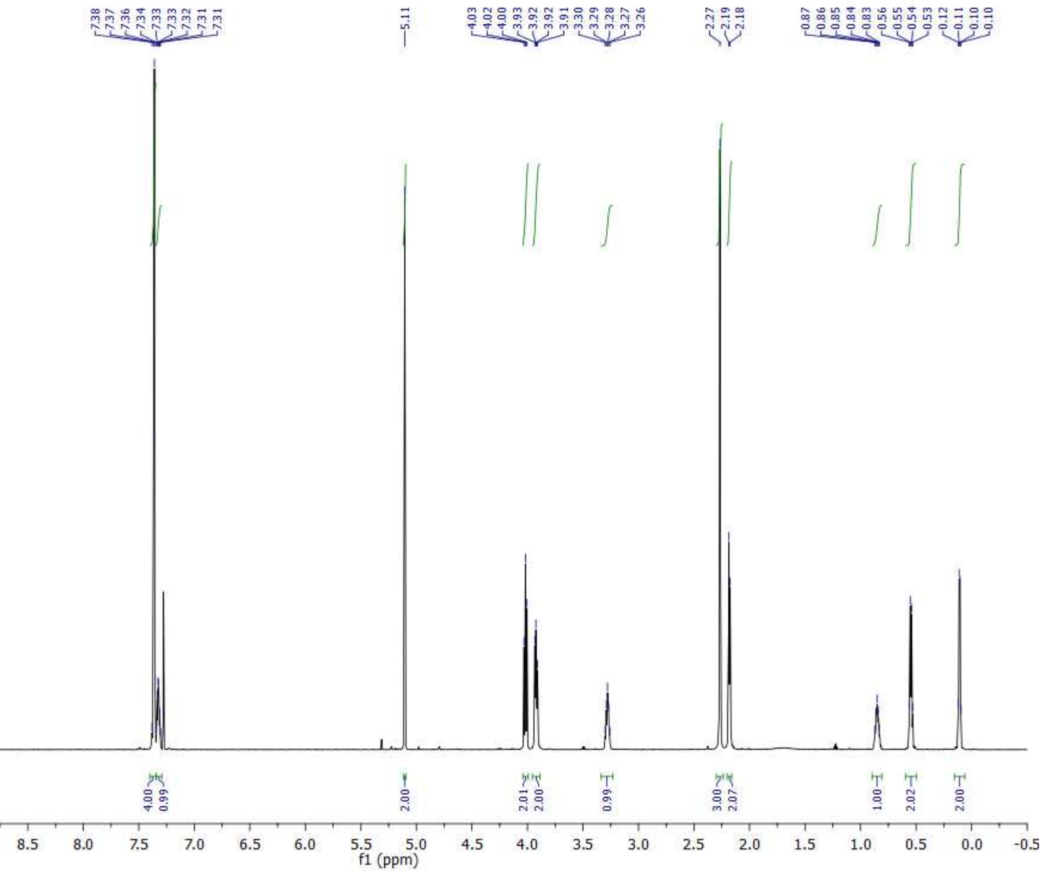

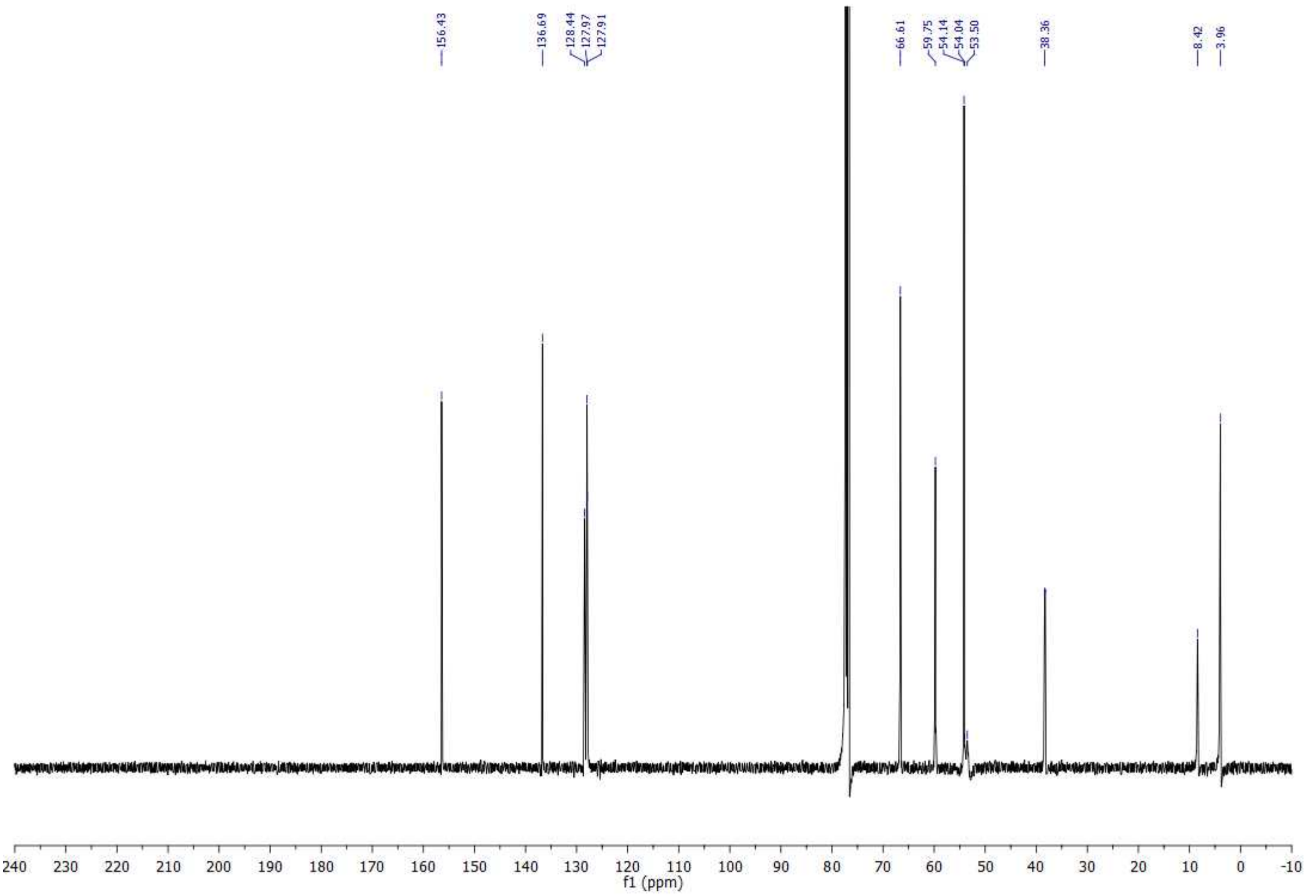

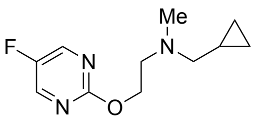

Me
F
N
N
O
N

1o

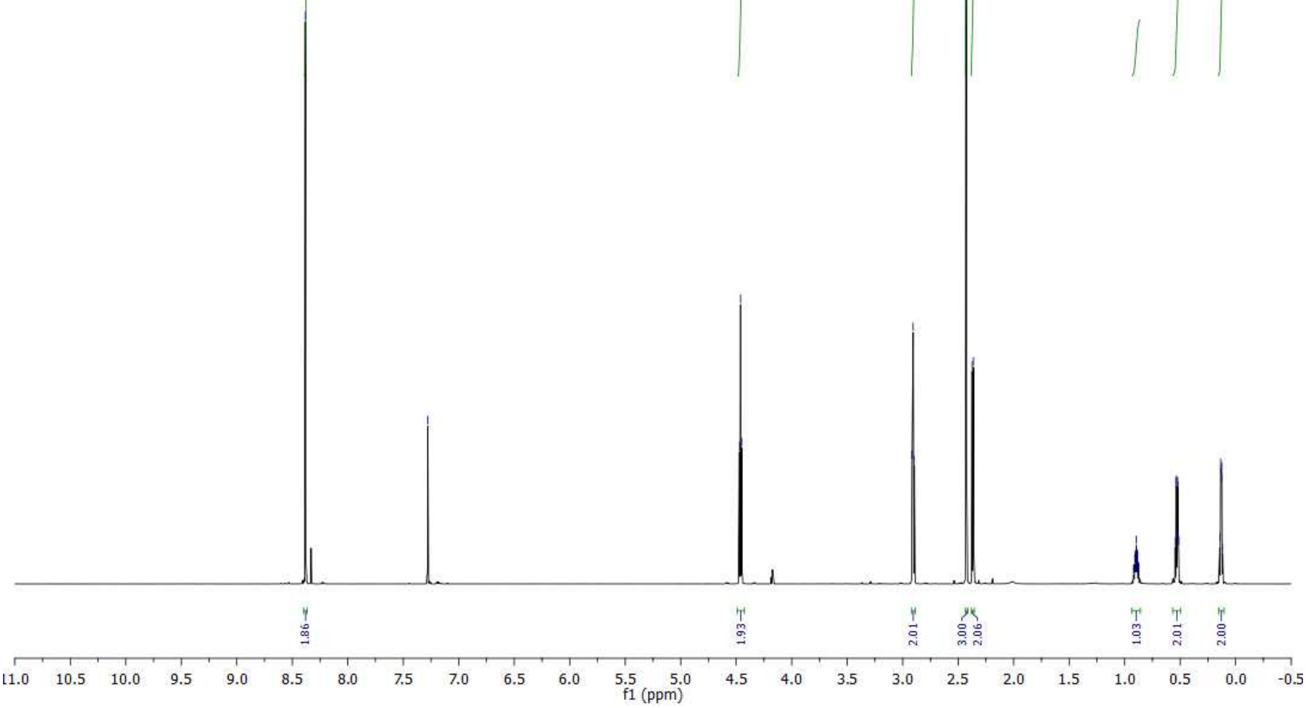

8.38
7.28
4.47
4.46
4.45
2.92
2.91
2.90
2.43
2.37
2.36
0.91
0.91
0.90
0.90
0.90
0.89
0.89
0.89
0.88
0.88
0.53
0.52
0.14
0.13
0.13
0.13
0.13
0.13
0.12
1.86
1.93
2.01
3.00
2.06
1.03
2.01
2.00
f1 (ppm)

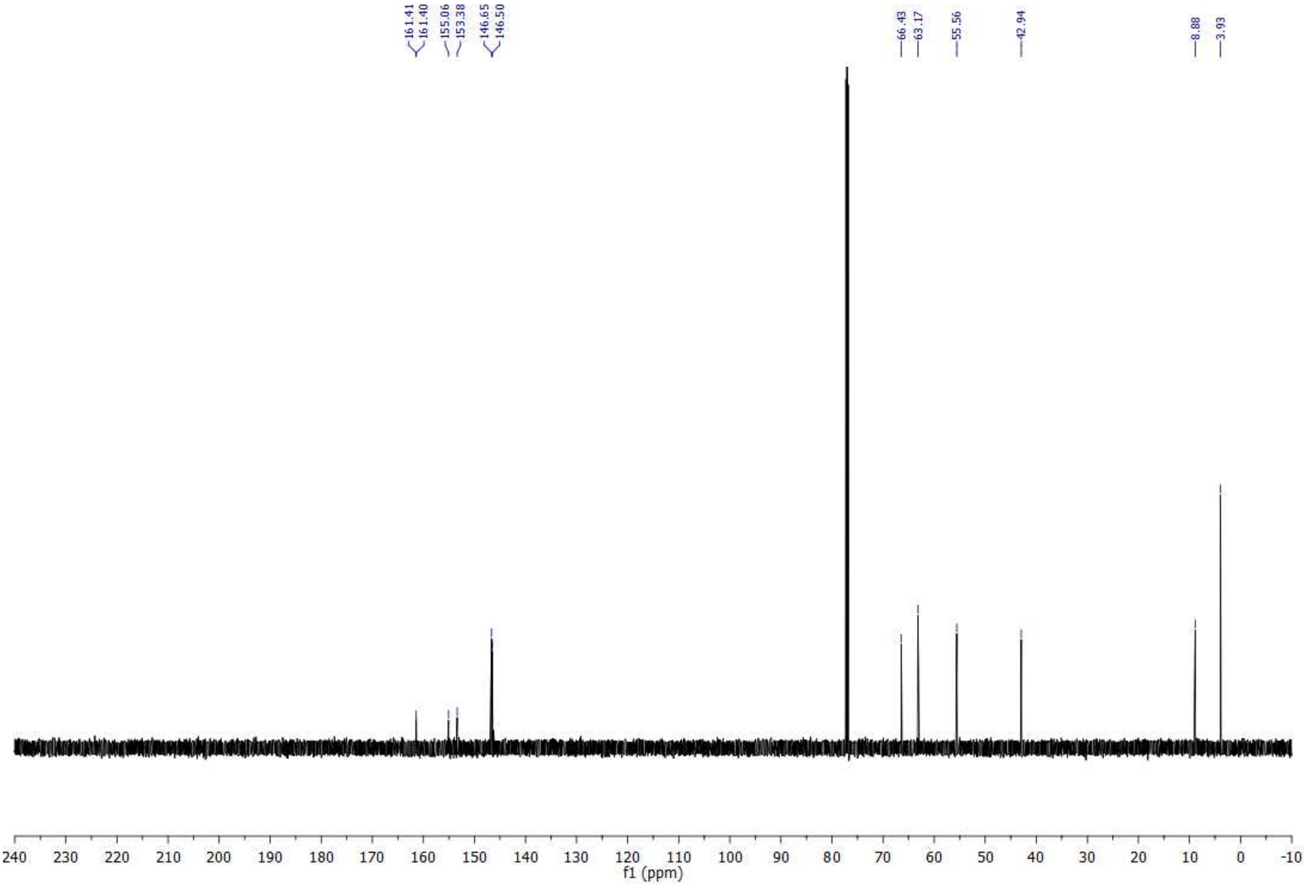

161.41
161.40
155.06
153.38
146.65
146.50
66.43
63.17
55.56
42.94
8.88
3.93
f1 (ppm)

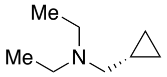
Me
Me
N

1p

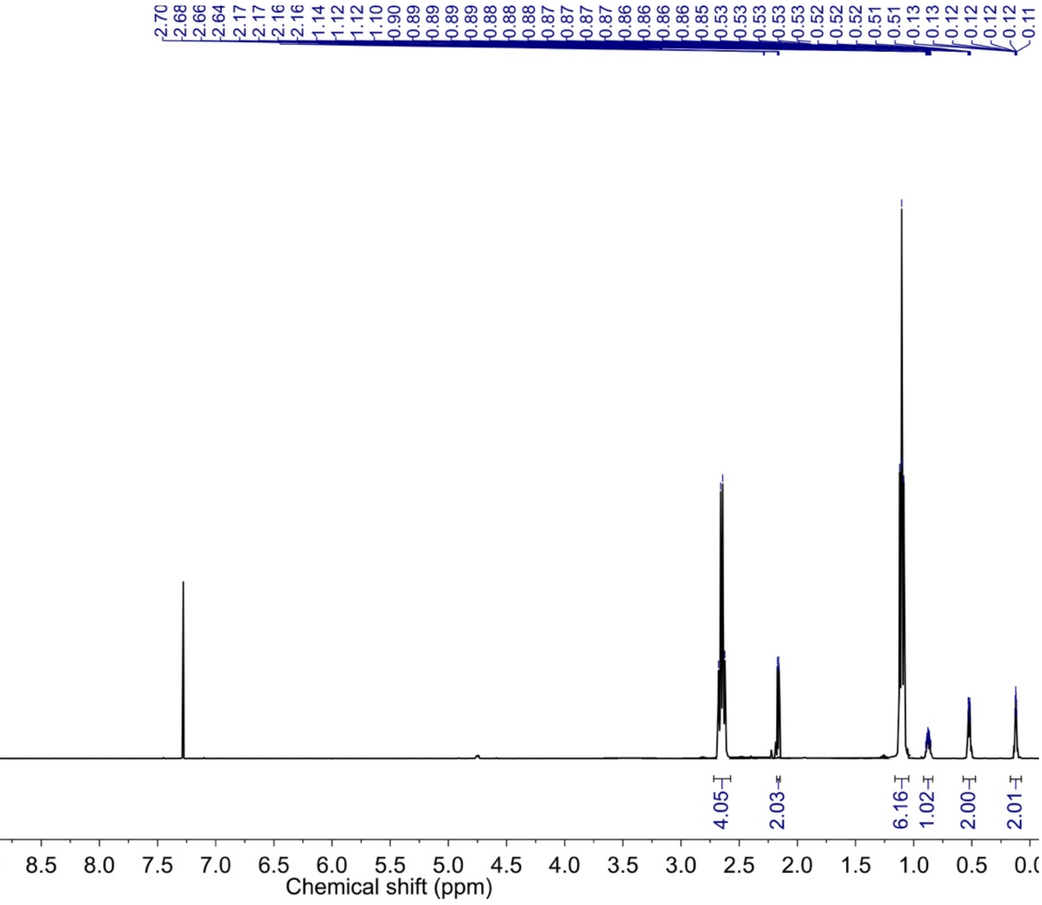
2.70 2.68 2.66 2.64 2.17 2.17 2.16 2.16 1.14 1.12 1.12 1.10 0.90 0.89 0.89 0.89 0.88 0.88 0.88 0.87 0.87 0.87 0.87 0.86 0.86 0.86 0.86 0.85 0.53 0.53 0.53 0.53 0.53 0.52 0.52 0.52 0.51 0.51 0.13 0.13 0.12 0.12 0.12 0.12 0.11
4.05
2.03
6.16
1.02
2.00
2.01
Chemical shift (ppm)

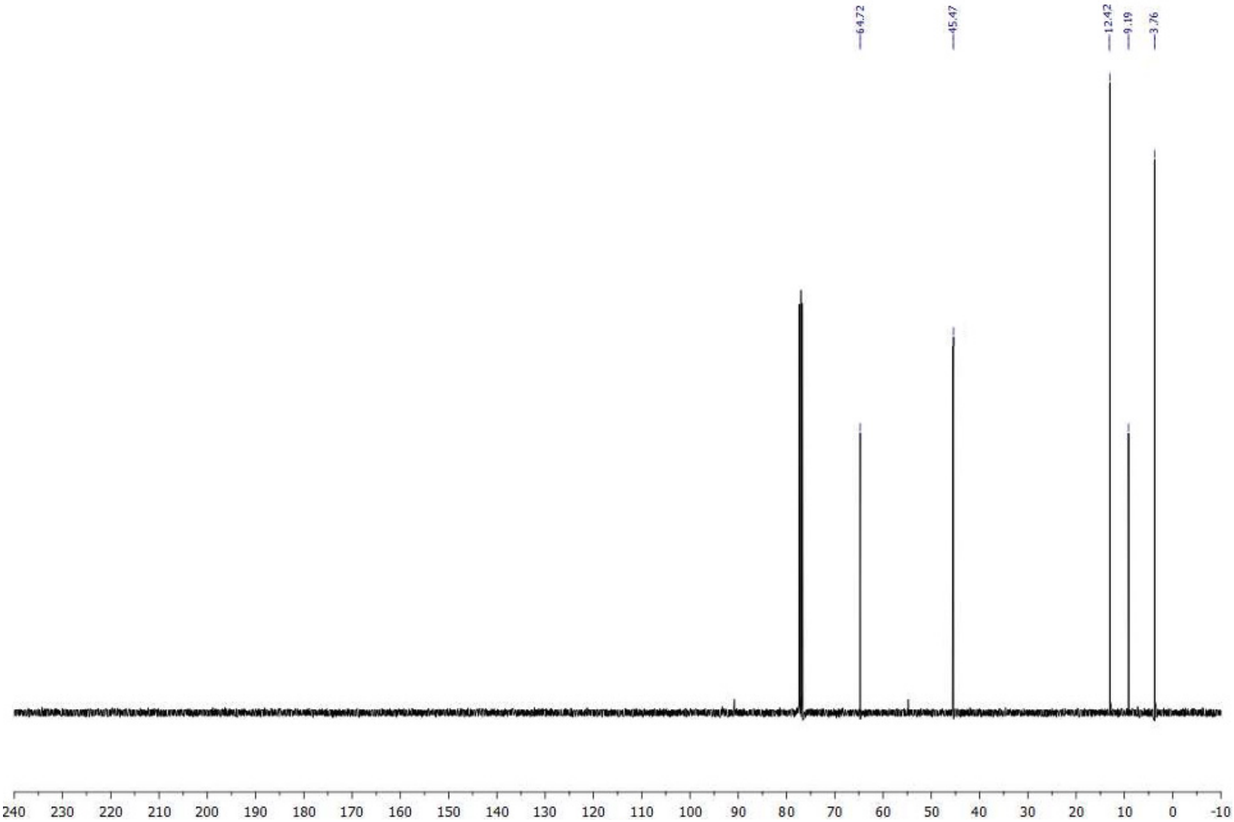
64.72
45.47
12.42
9.19
3.76

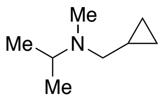

Me
Me
N
Me

1q

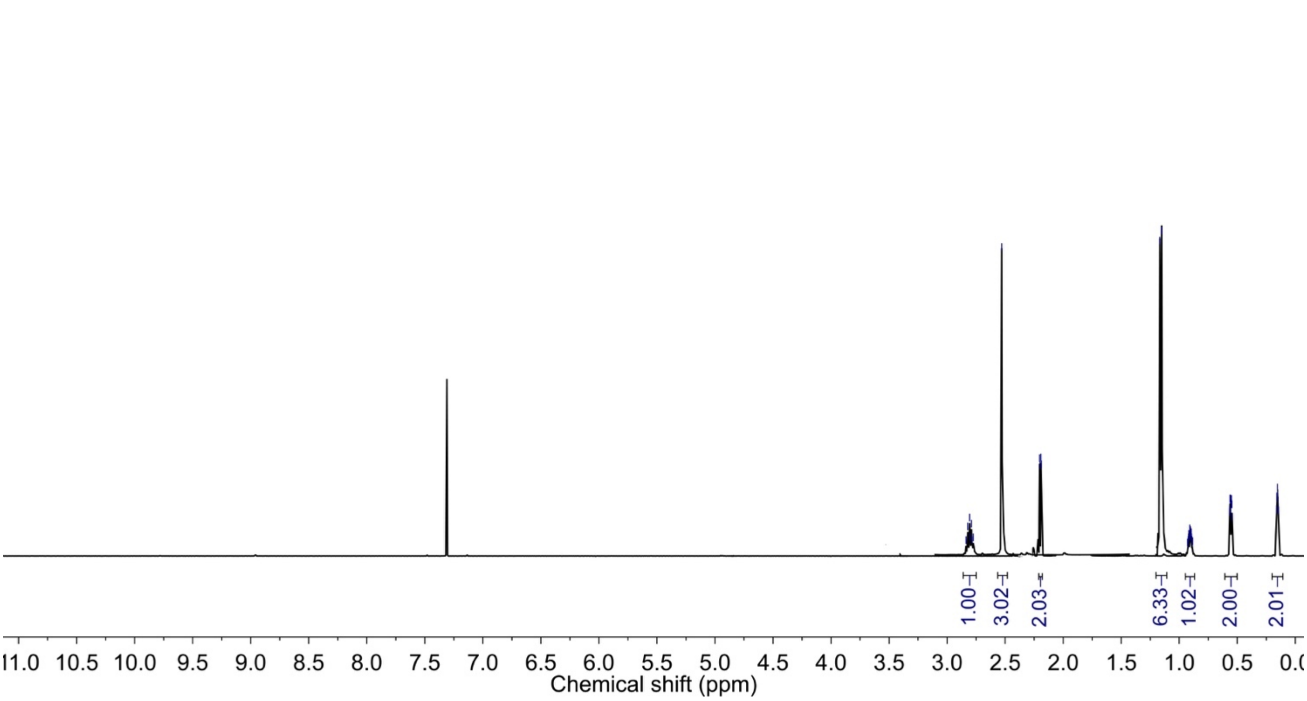

2.72
2.71
2.69
2.69
2.68
2.67
2.42
2.20
2.20
2.19
2.19
1.07
1.05
0.93
0.93
0.92
0.92
0.92
0.92
0.91
0.91
0.91
0.90
0.90
0.90
0.90
0.89
0.89
0.89
0.88
0.57
0.56
0.56
0.56
0.56
0.55
0.55
0.55
0.54
0.16
0.16
0.16
0.15
0.15
0.15
0.15
1.00
3.02
2.03
6.33
1.02
2.00
2.01
11.0 10.5 10.0 9.5 9.0 8.5 8.0 7.5 7.0 6.5 6.0 5.5 5.0 4.5 4.0 3.5 3.0 2.5 2.0 1.5 1.0 0.5 0.0
Chemical shift (ppm)

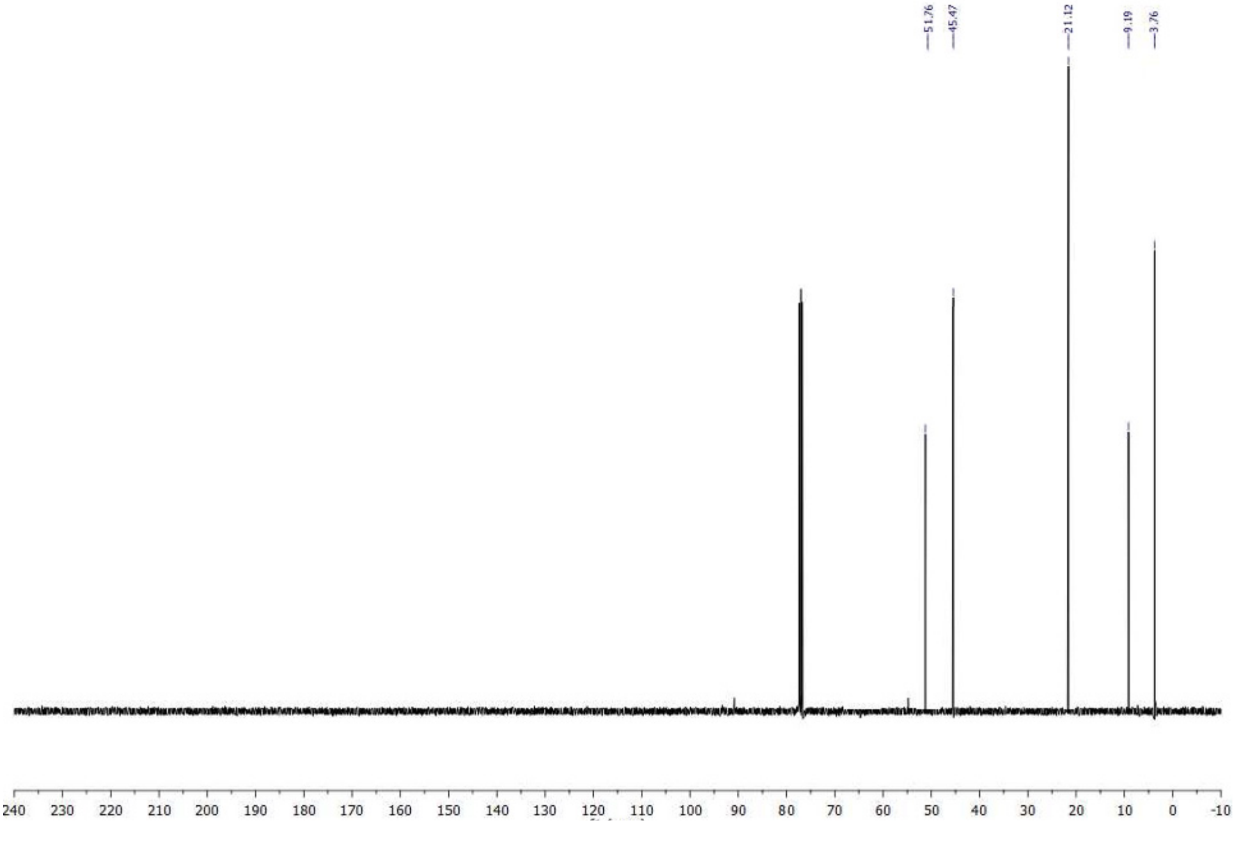

51.76
45.47
21.12
9.19
3.76
240 230 220 210 200 190 180 170 160 150 140 130 120 110 100 90 80 70 60 50 40 30 20 10 0 -10

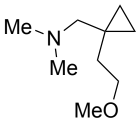
Me
N
Me
MeO

1r

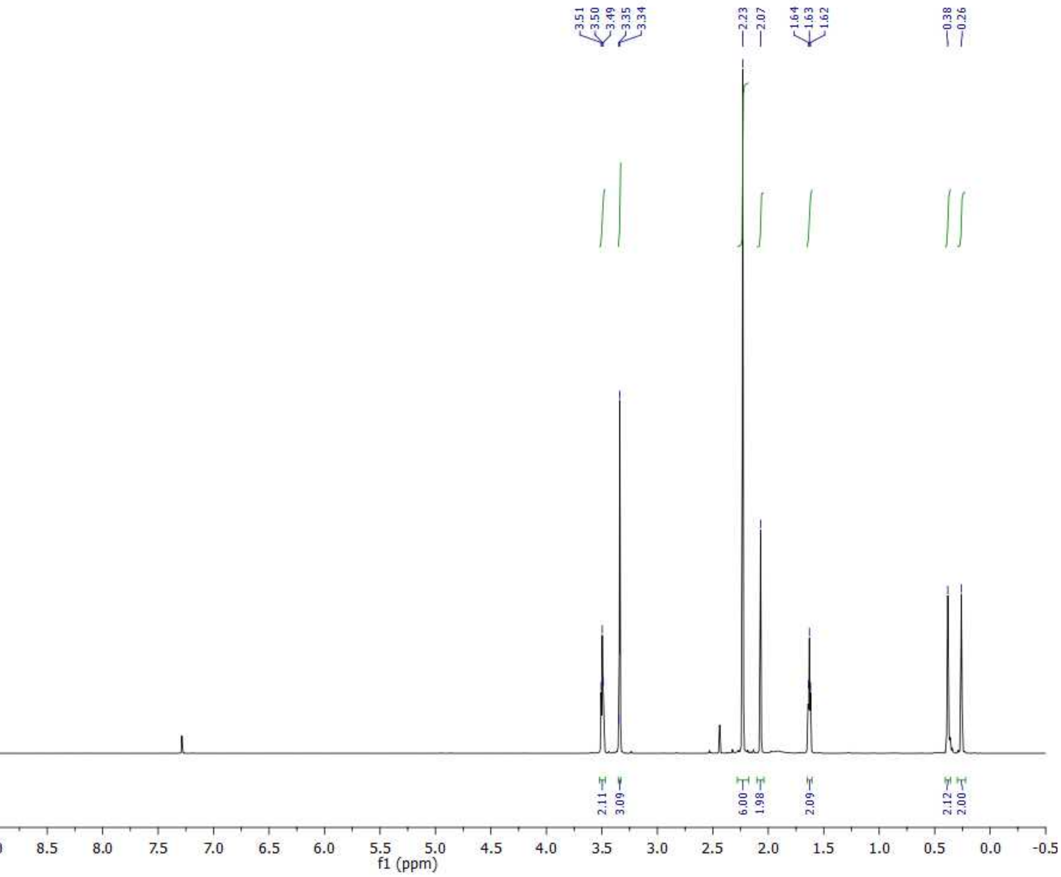
3.51
3.50
3.49
3.35
3.34
2.23
2.07
1.64
1.63
1.62
0.38
0.26
2.11
3.09
6.00
1.98
2.09
2.12
2.00
f1 (ppm)

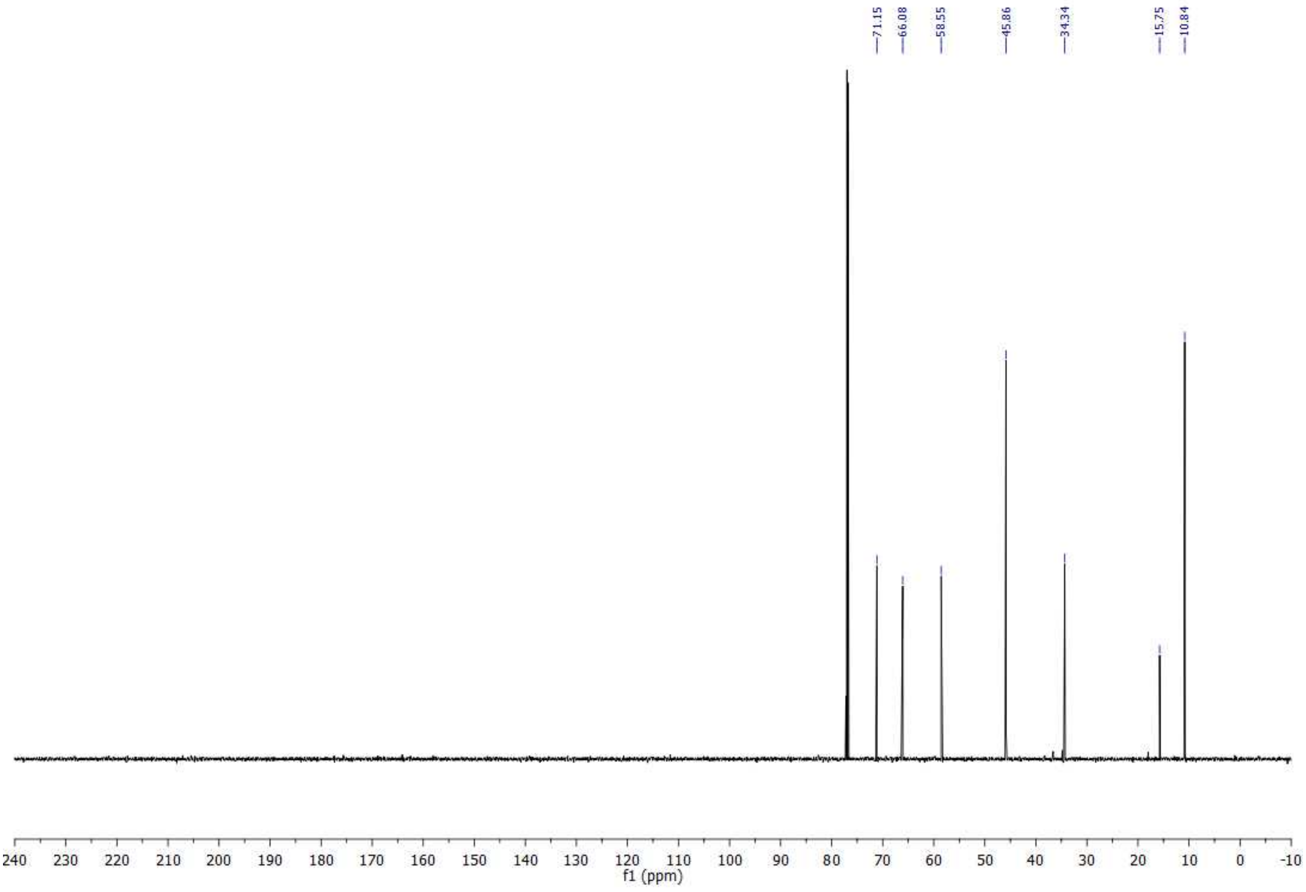
71.15
66.08
58.55
45.86
34.34
15.75
10.84
f1 (ppm)

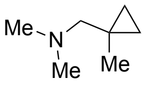
Me
N
Me
Me

1s

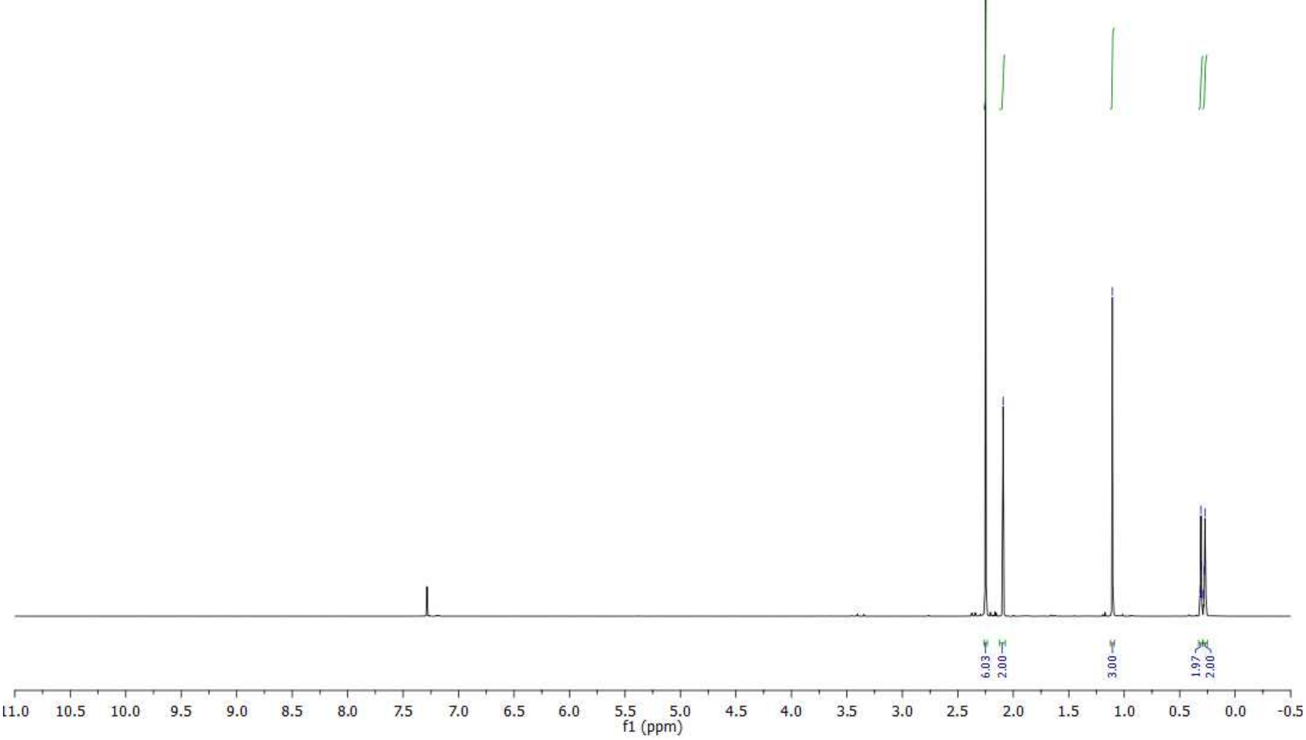
2.25
2.09
1.11
0.32
0.31
0.30
0.30
0.28
0.28
0.27
6.03
2.00
3.00
1.97
2.00
11.0 10.5 10.0 9.5 9.0 8.5 8.0 7.5 7.0 6.5 6.0 5.5 5.0 4.5 4.0 3.5 3.0 2.5 2.0 1.5 1.0 0.5 0.0 -0.5
f1 (ppm)

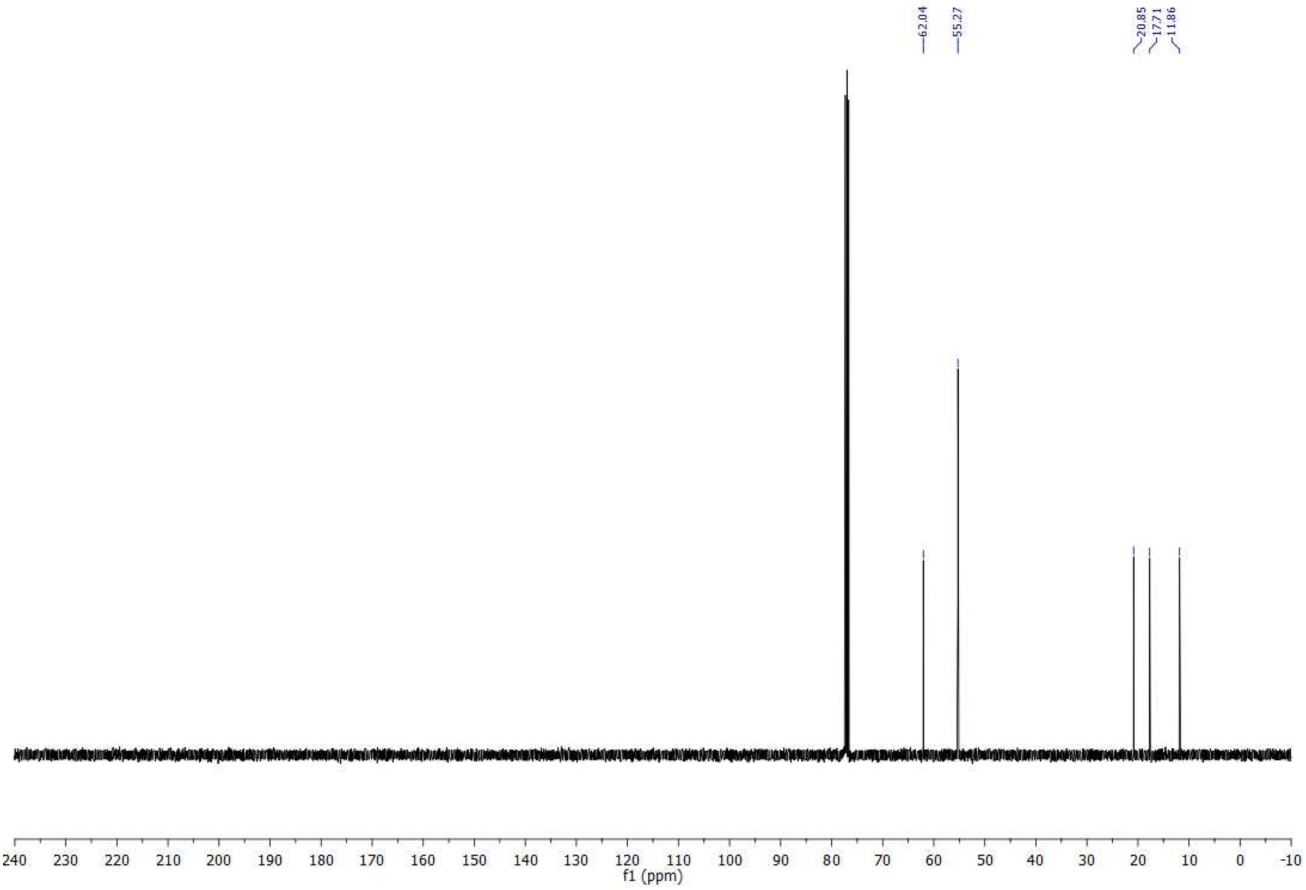
62.04
55.27
20.85
17.71
11.86
240 230 220 210 200 190 180 170 160 150 140 130 120 110 100 90 80 70 60 50 40 30 20 10 0 -10
f1 (ppm)

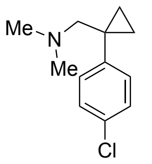
Me
N
Me
Cl

1t

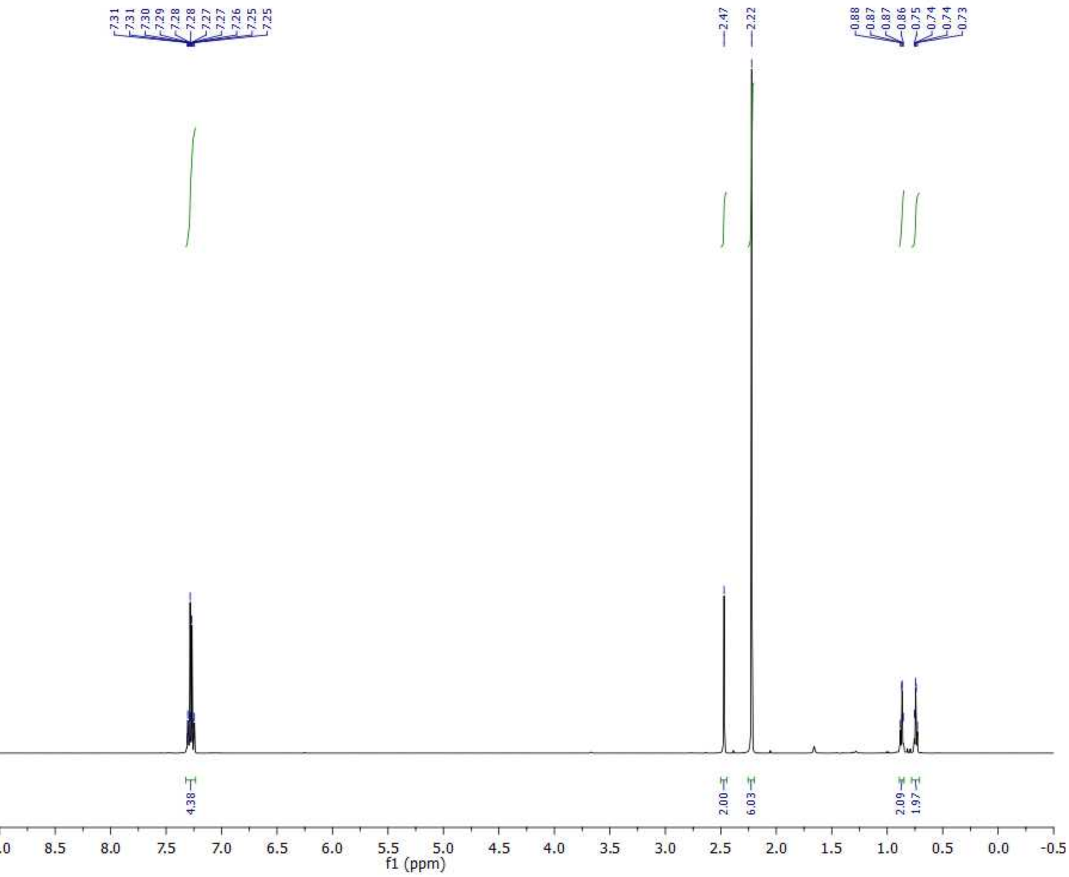
7.31
7.31
7.30
7.29
7.28
7.28
7.27
7.27
7.26
7.25
7.25
2.47
2.22
0.88
0.87
0.87
0.86
0.75
0.74
0.74
0.73
4.38
2.00
6.03
2.09
1.97
f1 (ppm)

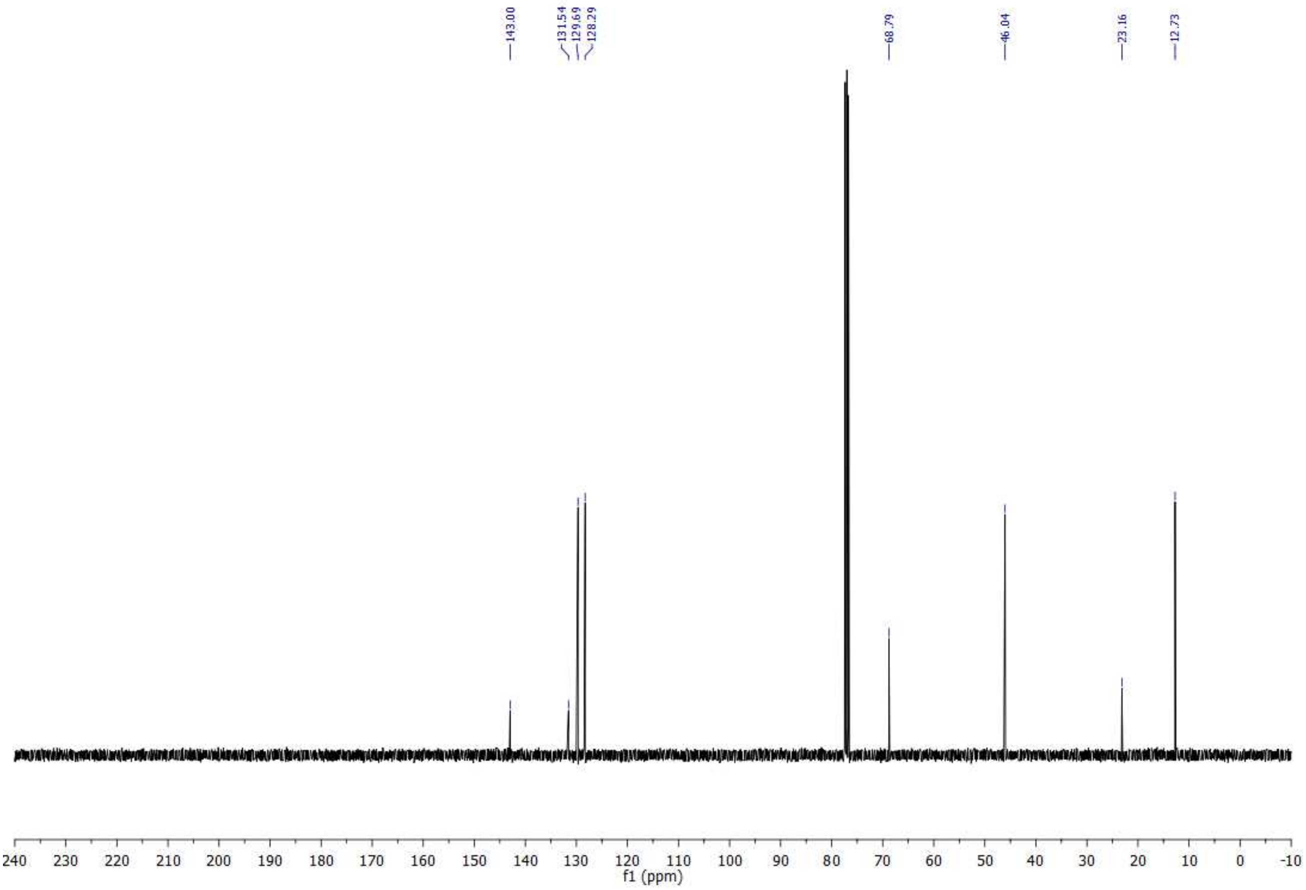
143.00
131.54
129.69
128.29
68.79
46.04
23.16
12.73
f1 (ppm)

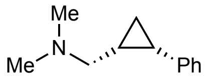
Me
N
Me
Ph

3a

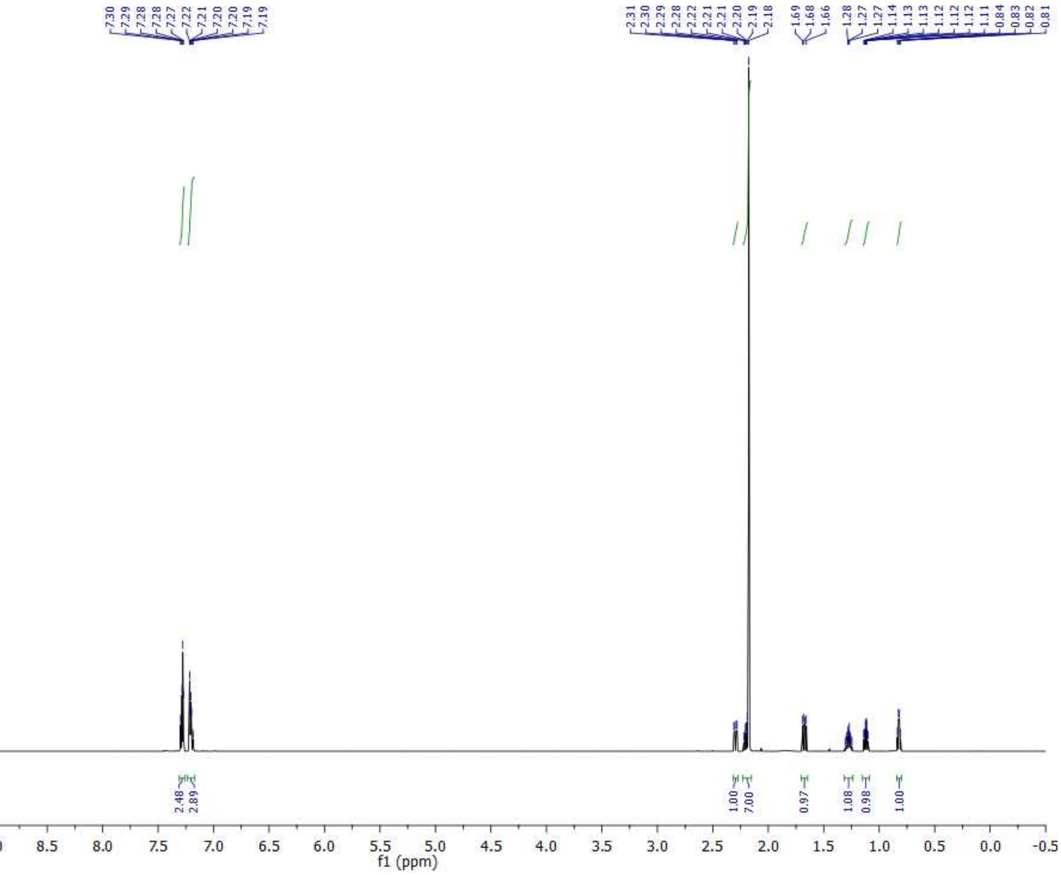
7.30 7.29 7.28 7.28 7.27 7.22 7.21 7.21 7.20 7.20 7.19 7.19
2.31 2.30 2.29 2.28 2.22 2.21 2.21 2.20 2.19 2.19 2.18
1.69 1.68 1.66
1.28 1.27 1.27 1.14 1.13 1.13 1.12 1.12 1.12 1.11 0.84 0.83 0.82 0.81
2.48 2.89
1.00 7.00
0.97
1.08 0.98
1.00
f1 (ppm)

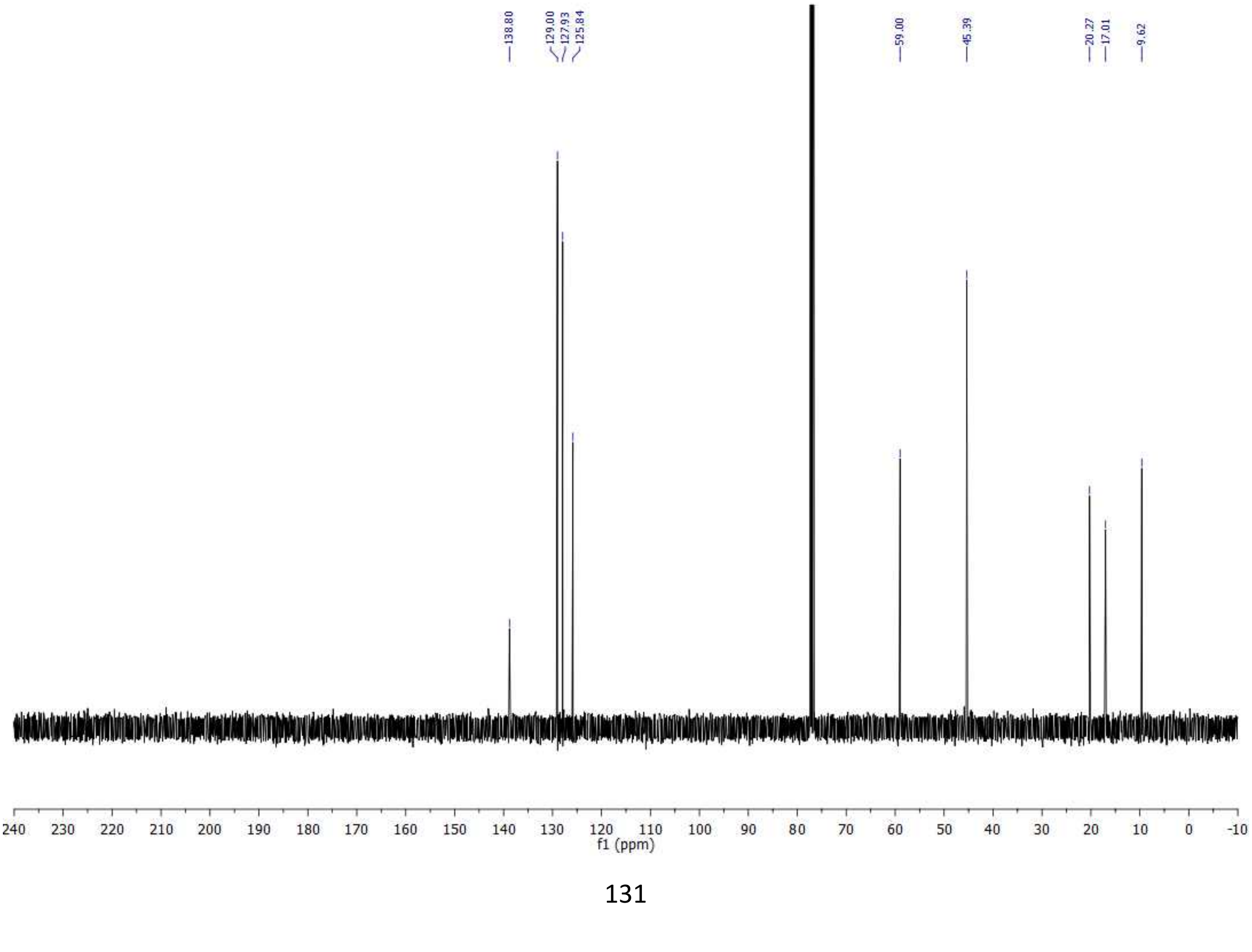
138.80
129.00 127.93 125.84
59.00
45.39
20.27 17.01
9.62
f1 (ppm)

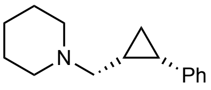
N
Ph

3b

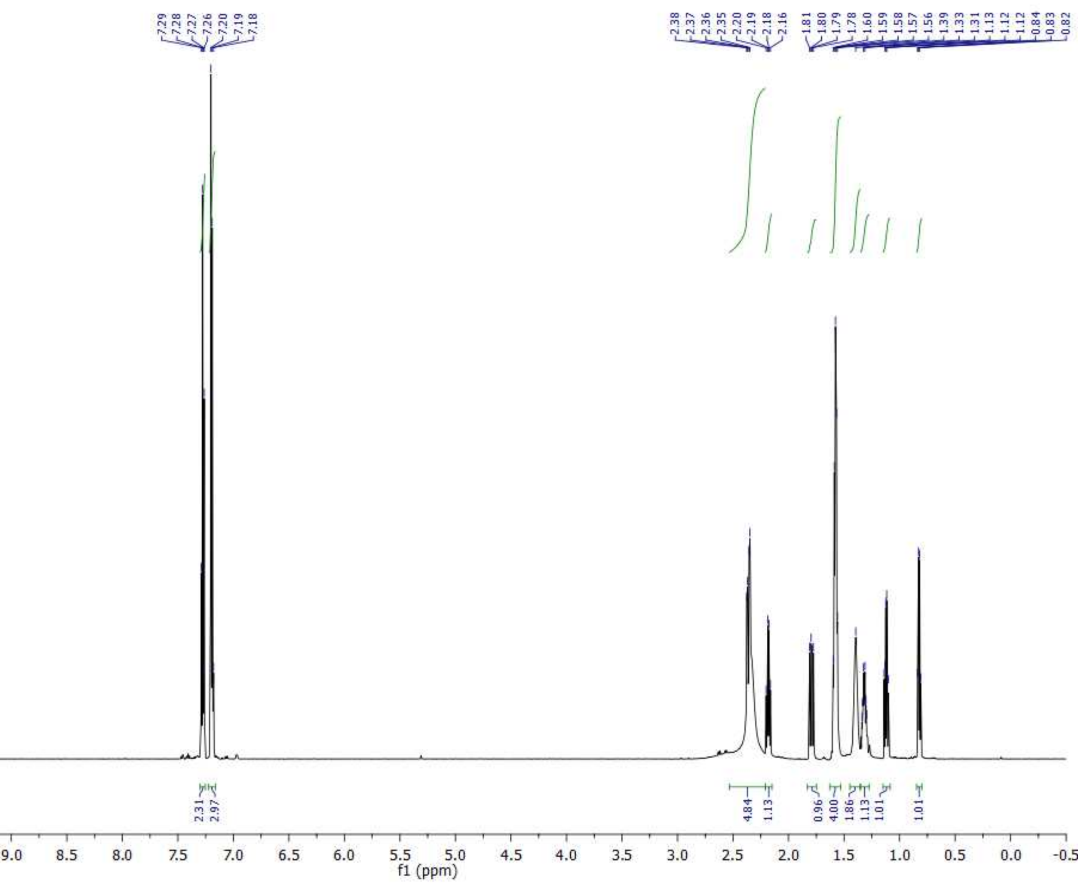
7.29
7.28
7.27
7.26
7.20
7.19
7.18
2.38
2.37
2.36
2.35
2.20
2.19
2.18
2.16
1.81
1.80
1.79
1.78
1.60
1.59
1.58
1.57
1.56
1.39
1.33
1.31
1.13
1.12
1.12
0.84
0.83
0.82
2.31
2.97
4.84
1.13
0.96
4.00
1.86
1.13
1.01
1.01
f1 (ppm)

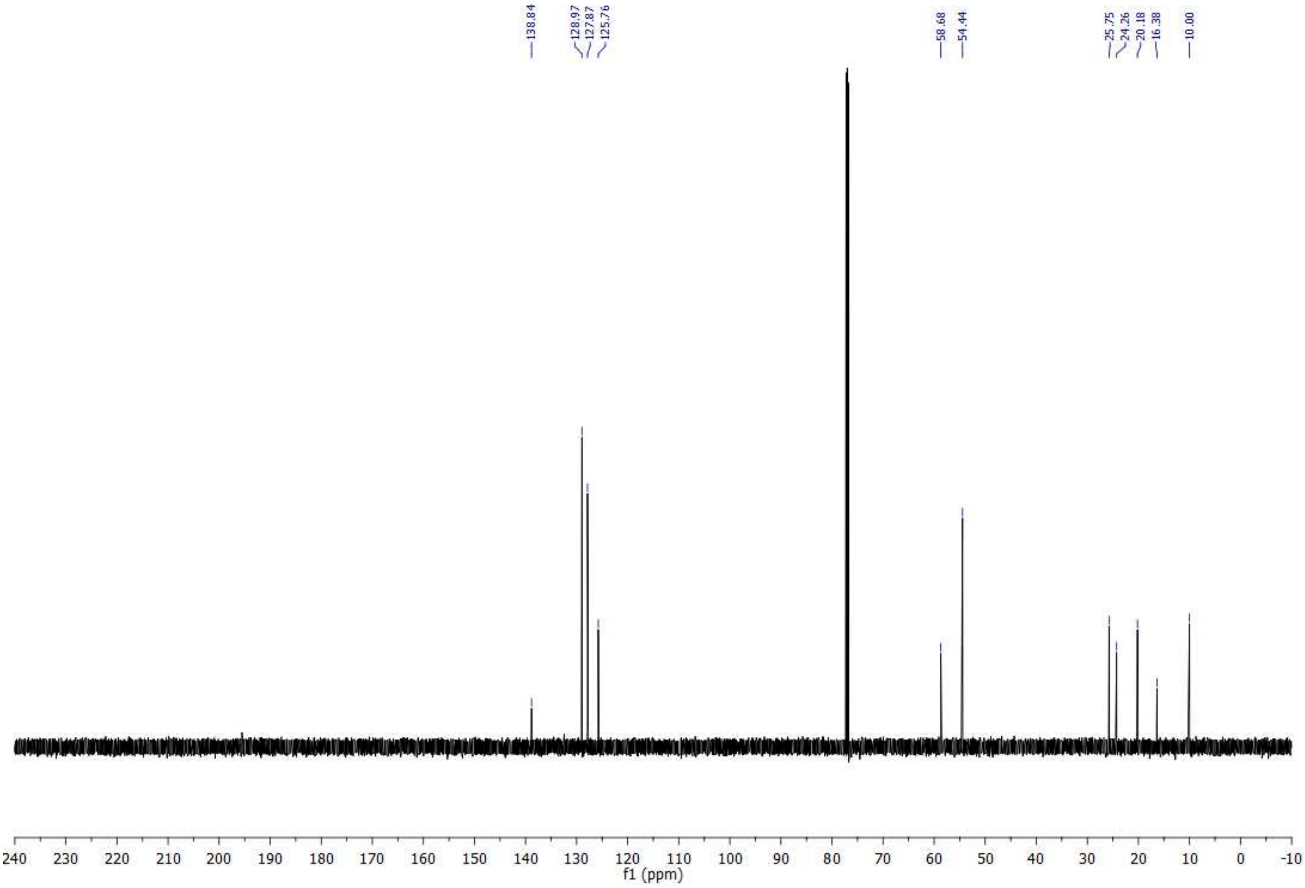
138.84
128.97
127.87
125.76
58.68
54.44
25.75
24.26
20.18
16.38
10.00
f1 (ppm)

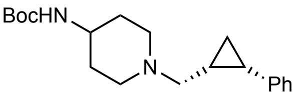

3c

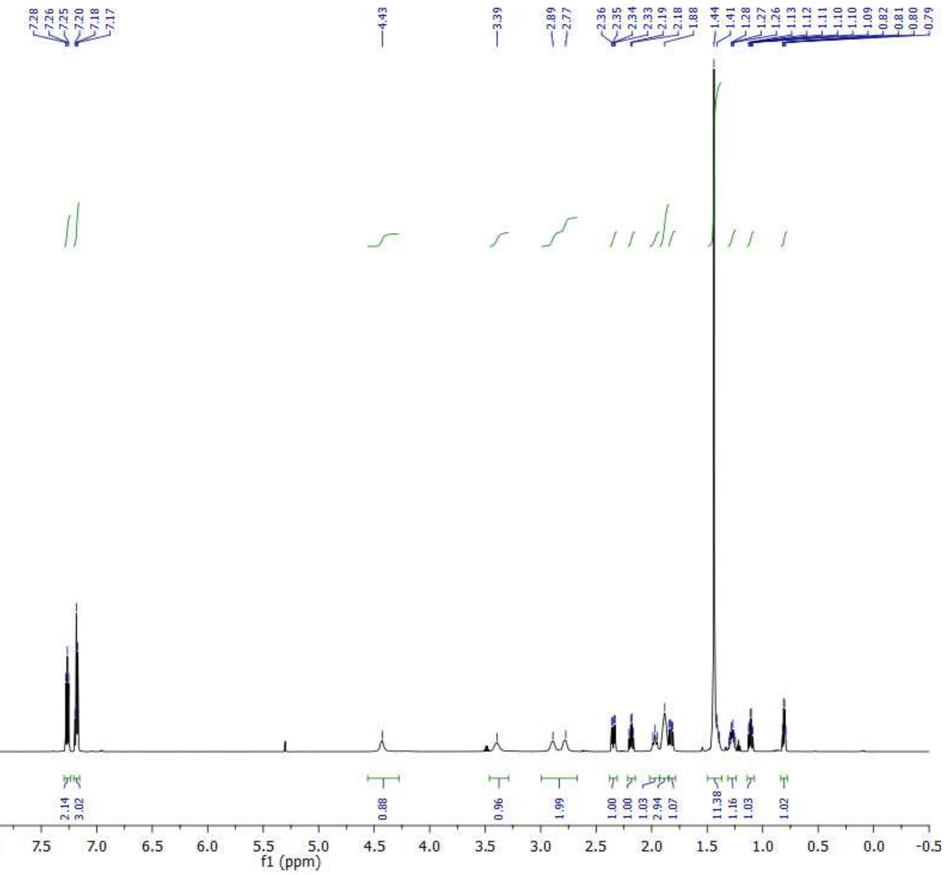

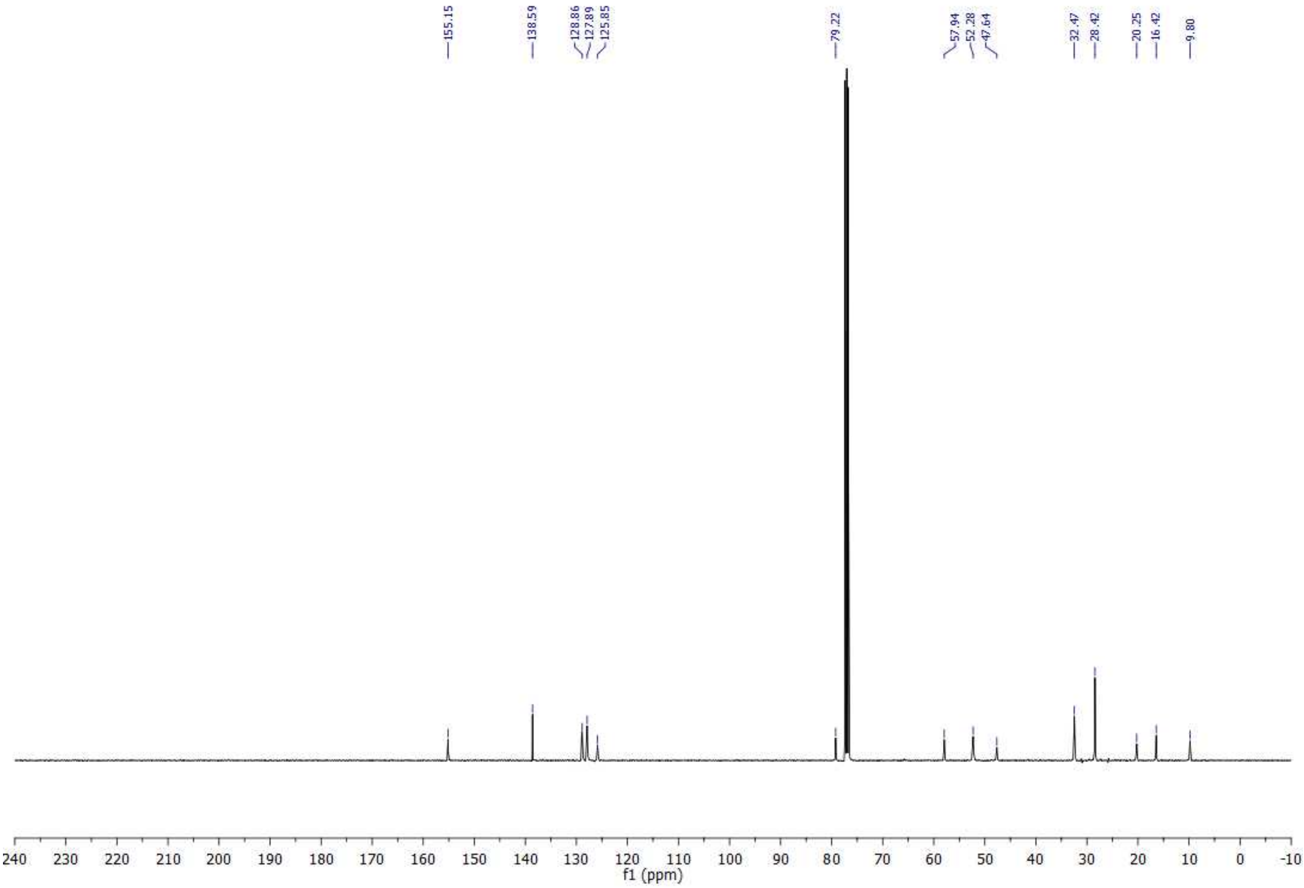

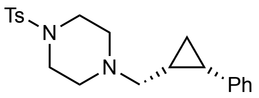

Ts
N
N
Ph

3d

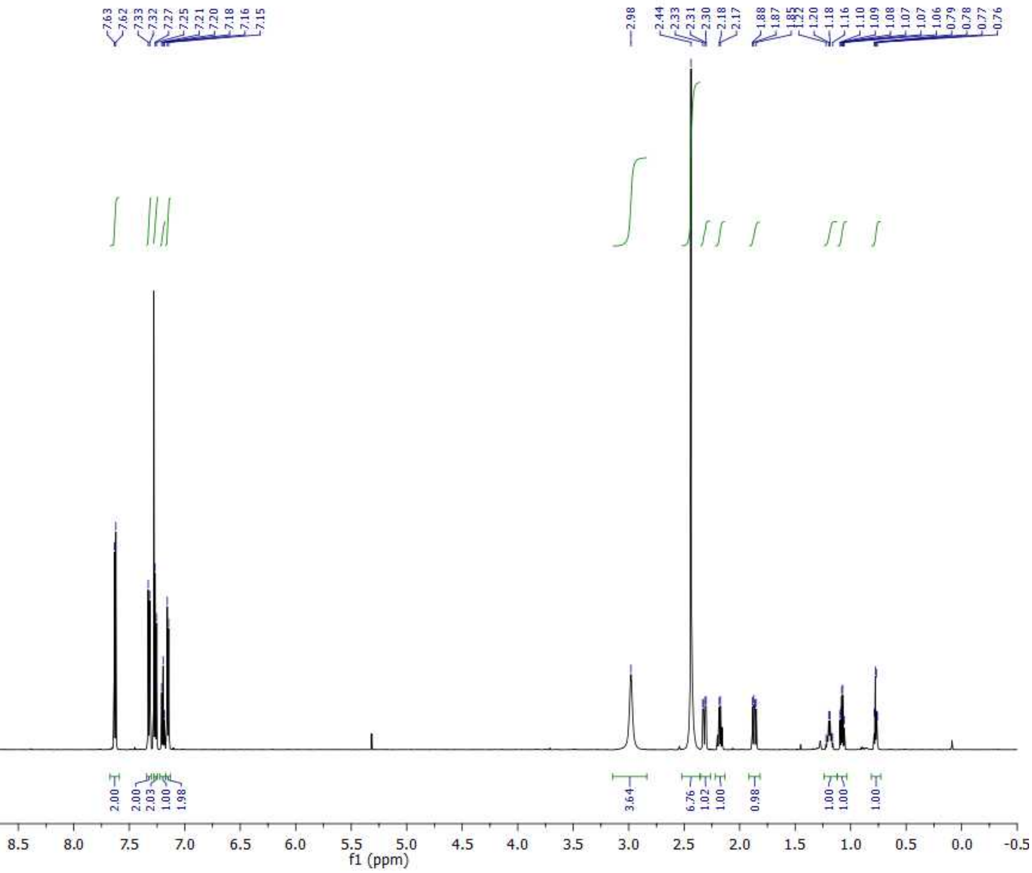

f1 (ppm)

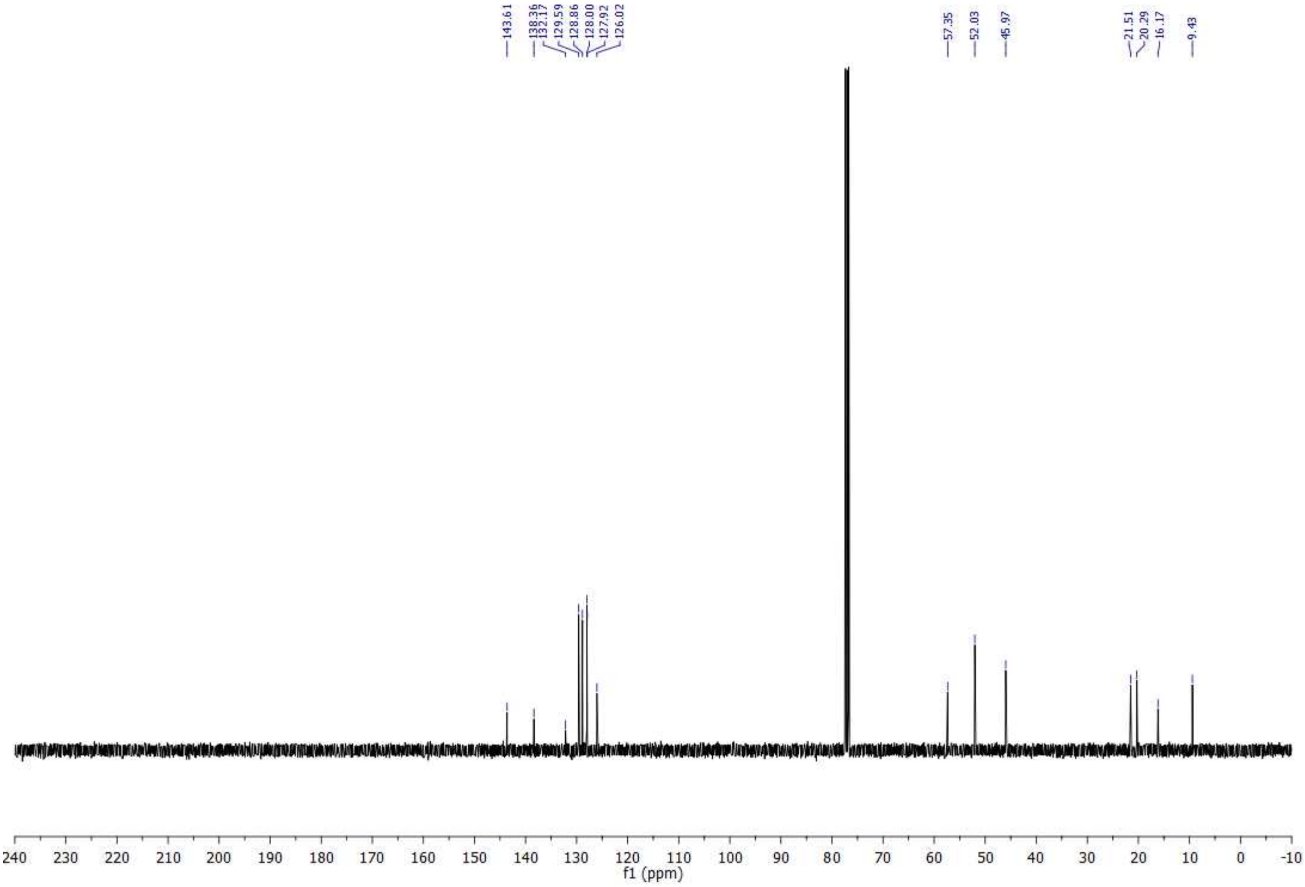

143.61
138.36
132.17
129.59
128.86
128.00
127.92
126.02
57.35
52.03
45.97
21.51
20.29
16.17
9.43
f1 (ppm)

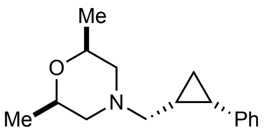
Me
O
N
Me
Ph

3e

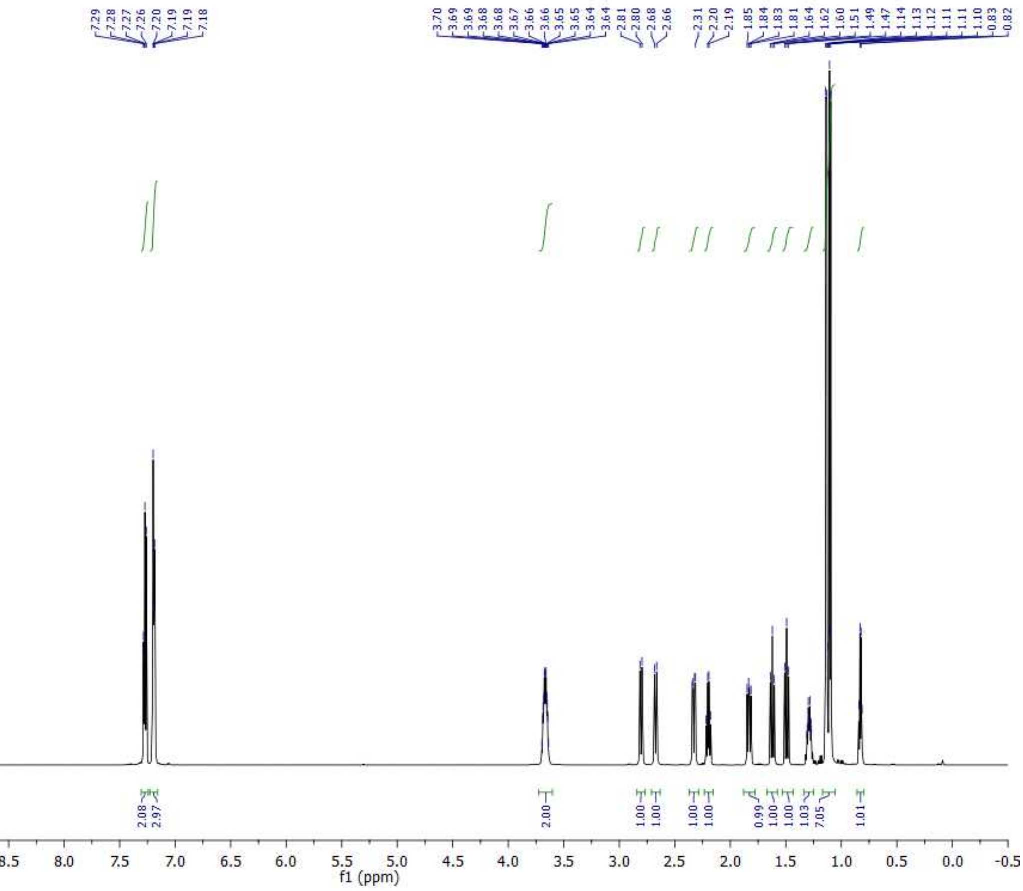
7.29 7.28 7.27 7.26 7.20 7.19 7.19 7.18
3.70 3.69 3.69 3.68 3.67 3.66 3.66 3.65 3.65 3.64 3.64
2.81 2.80 2.68 2.66
2.31 2.20 2.19
1.85 1.84 1.83 1.81 1.64 1.62 1.60 1.51 1.49 1.47 1.14 1.13 1.12 1.11 1.11 1.10 0.83 0.82
2.08 2.97 2.00 1.00 1.00 1.00 1.00 0.99 1.00 1.00 1.03 7.05 1.01
f1 (ppm)

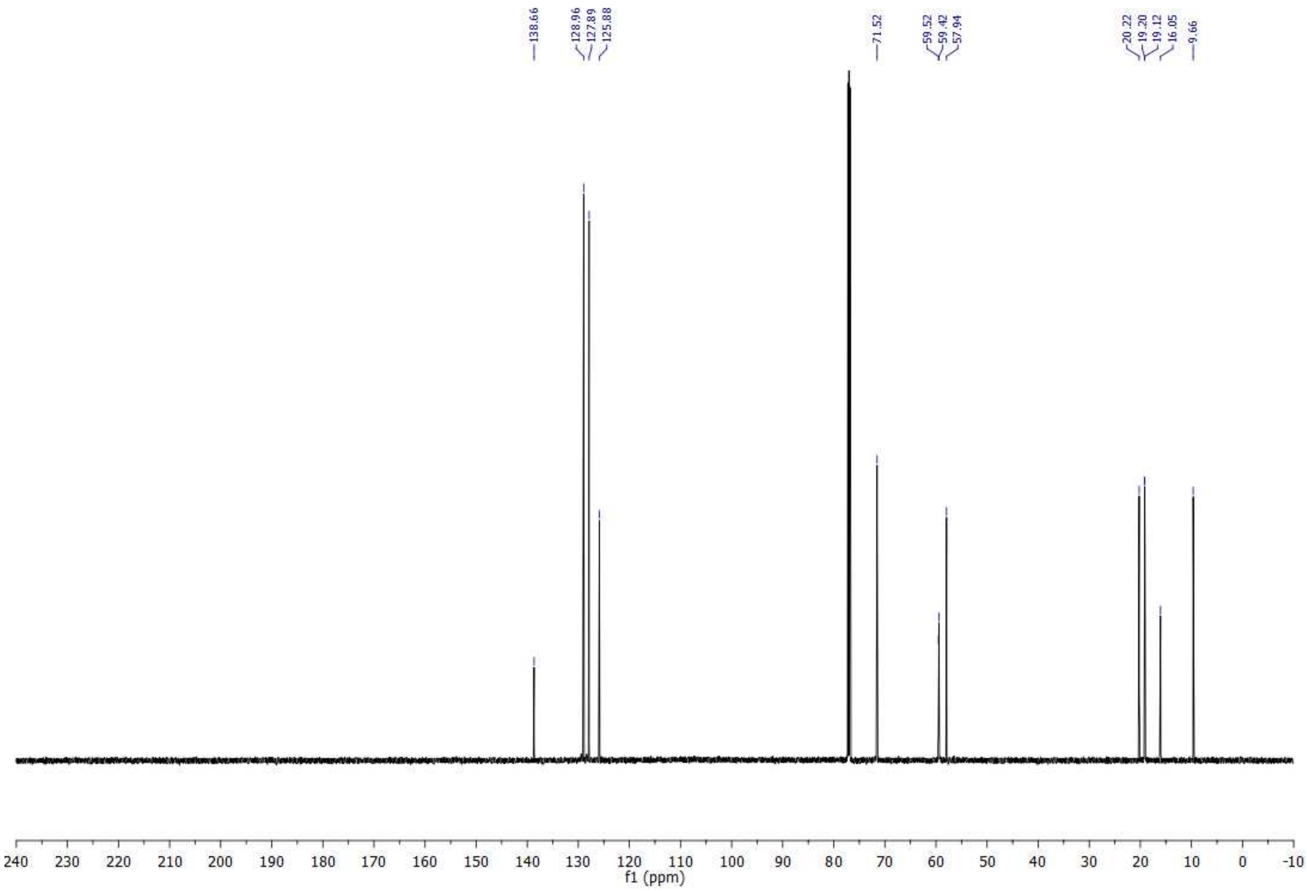
138.66
128.96 127.89 125.88
71.52
59.52 59.42 57.94
20.22 19.20 19.12 16.05 9.66
f1 (ppm)

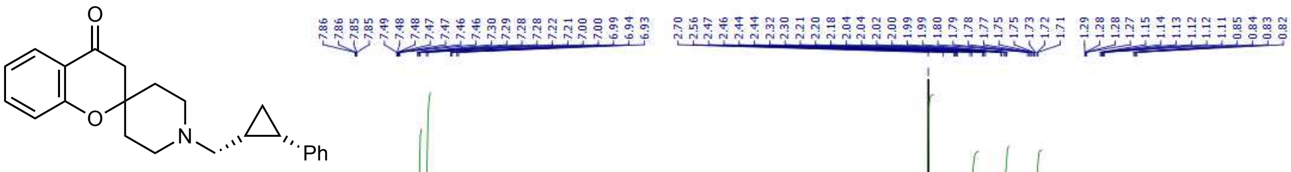

3f

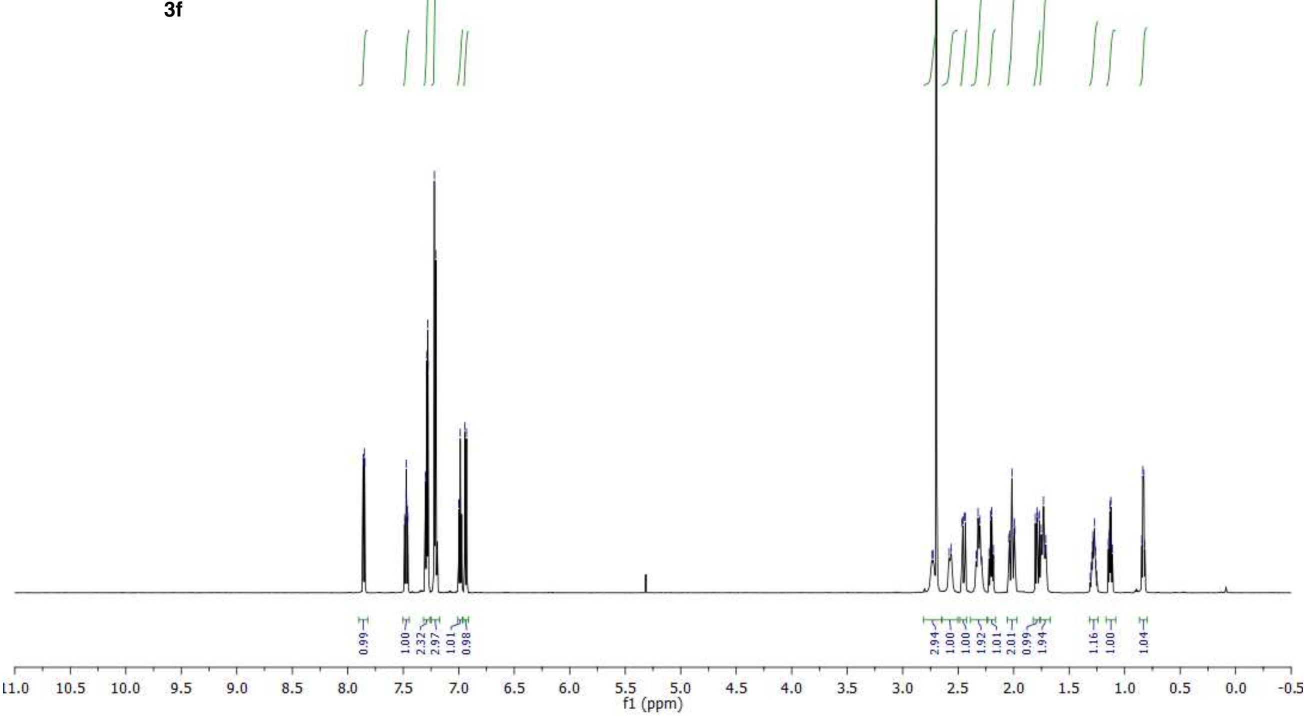
7.86
7.86
7.85
7.85
7.49
7.48
7.48
7.47
7.47
7.46
7.46
7.30
7.29
7.28
7.28
7.22
7.21
7.00
7.00
6.99
6.94
6.93
2.70
2.56
2.47
2.46
2.44
2.44
2.32
2.30
2.21
2.20
2.18
2.04
2.04
2.02
2.00
1.99
1.99
1.80
1.79
1.78
1.77
1.75
1.75
1.75
1.73
1.72
1.71
1.29
1.28
1.28
1.27
1.15
1.14
1.13
1.12
1.12
1.11
0.85
0.84
0.83
0.82
0.99
1.00
2.32
2.97
1.01
0.98
2.94
1.00
1.00
1.92
1.01
2.01
0.99
1.94
1.16
1.00
1.04
f1 (ppm)

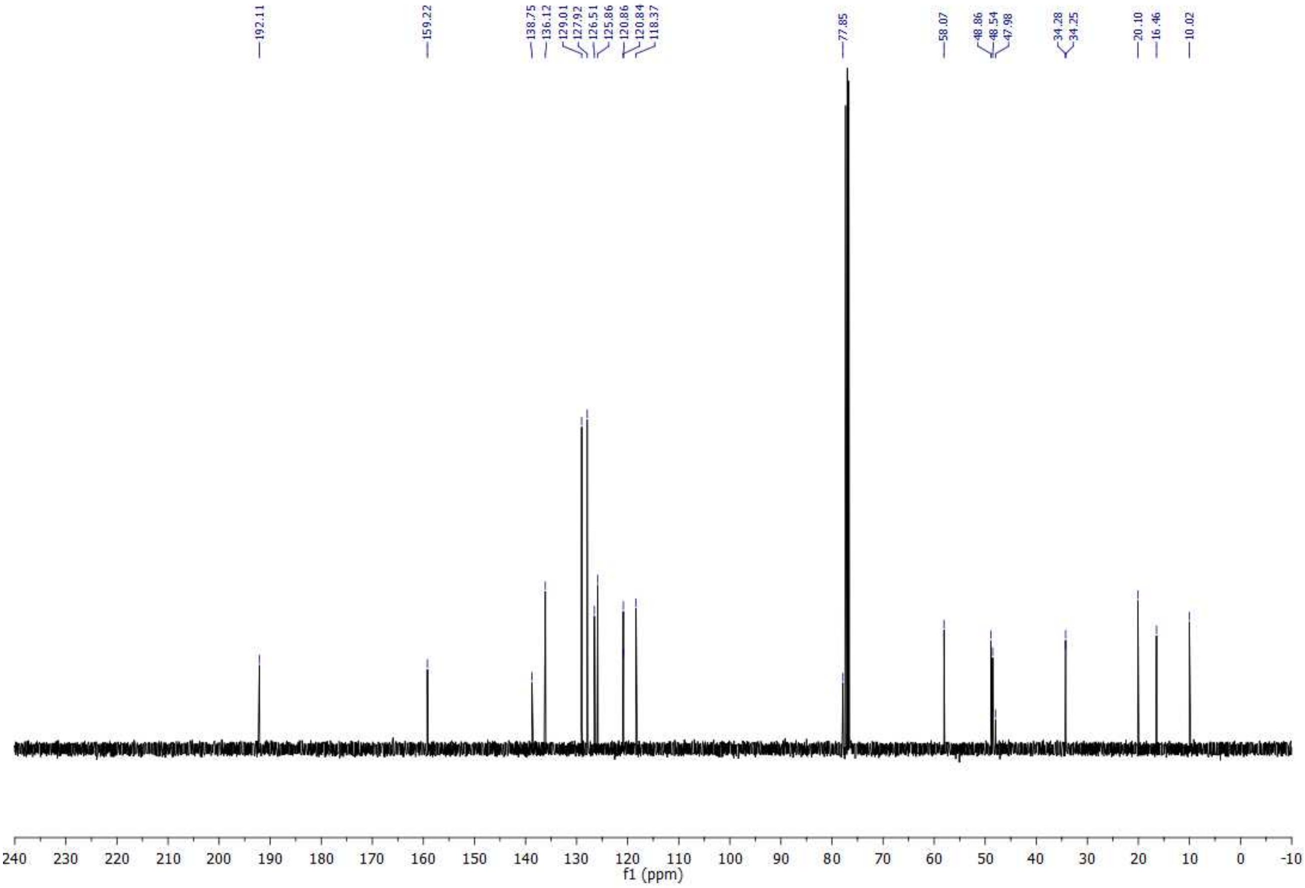
192.11
159.22
138.75
136.12
129.01
127.92
126.51
125.86
120.86
120.84
118.37
77.85
58.07
48.86
48.54
47.98
34.28
34.25
20.10
16.46
10.02
f1 (ppm)

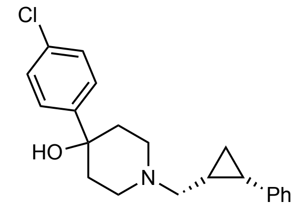
Cl
HO
N
Ph

3g

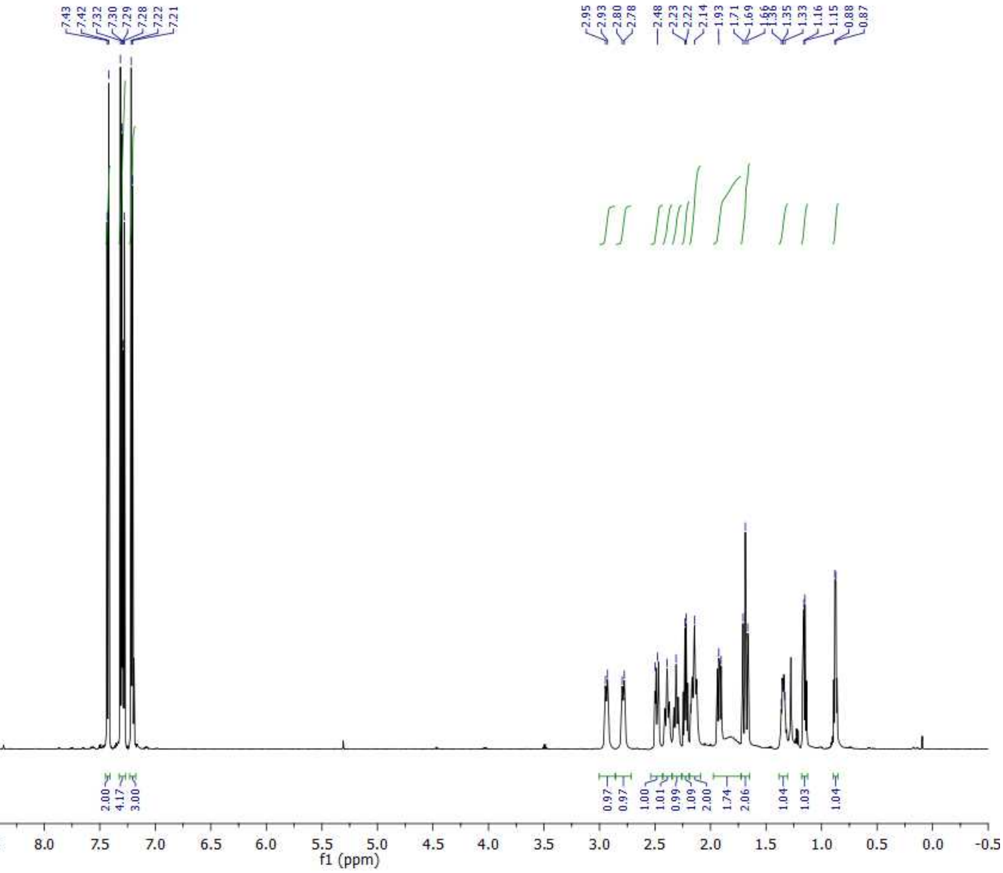
7.43 7.42 7.32 7.30 7.29 7.28 7.22 7.21
2.95 2.93 2.80 2.78 2.48 2.23 2.22 2.14 1.93 1.71 1.69 1.35 1.33 1.16 1.15 0.88 0.87
2.00 4.17 3.00
0.97 0.97 1.00 1.01 0.99 1.09 2.00 1.74 2.06 1.04 1.03 1.04
f1 (ppm)

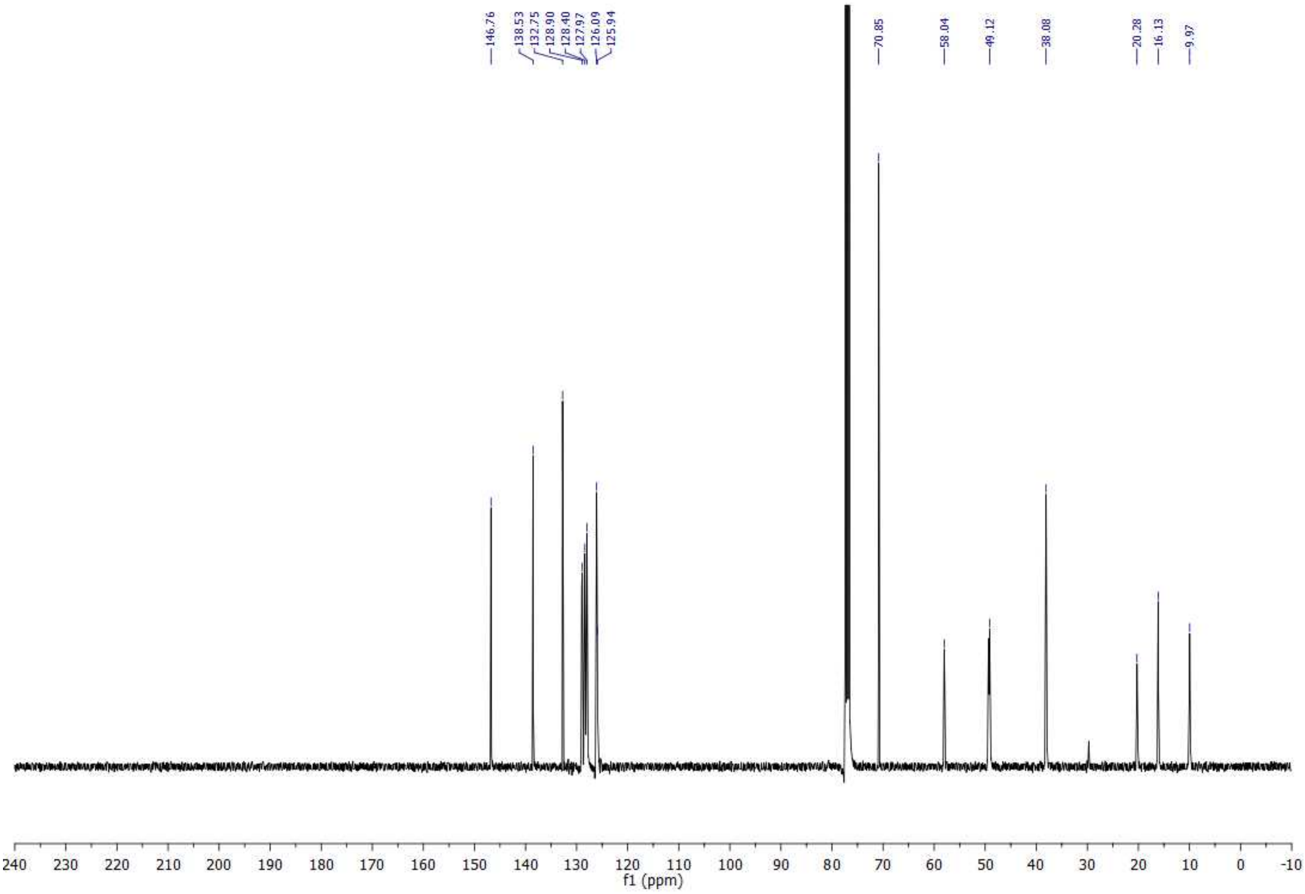
146.76
138.53 132.75 128.90 128.40 127.97 126.09 125.94
70.85
58.04
49.12
38.08
20.28
16.13
9.97
f1 (ppm)

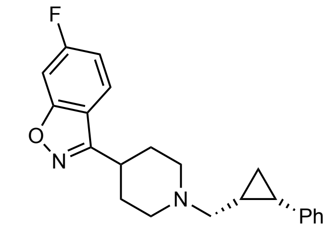
F
O
N
N
Ph

3h

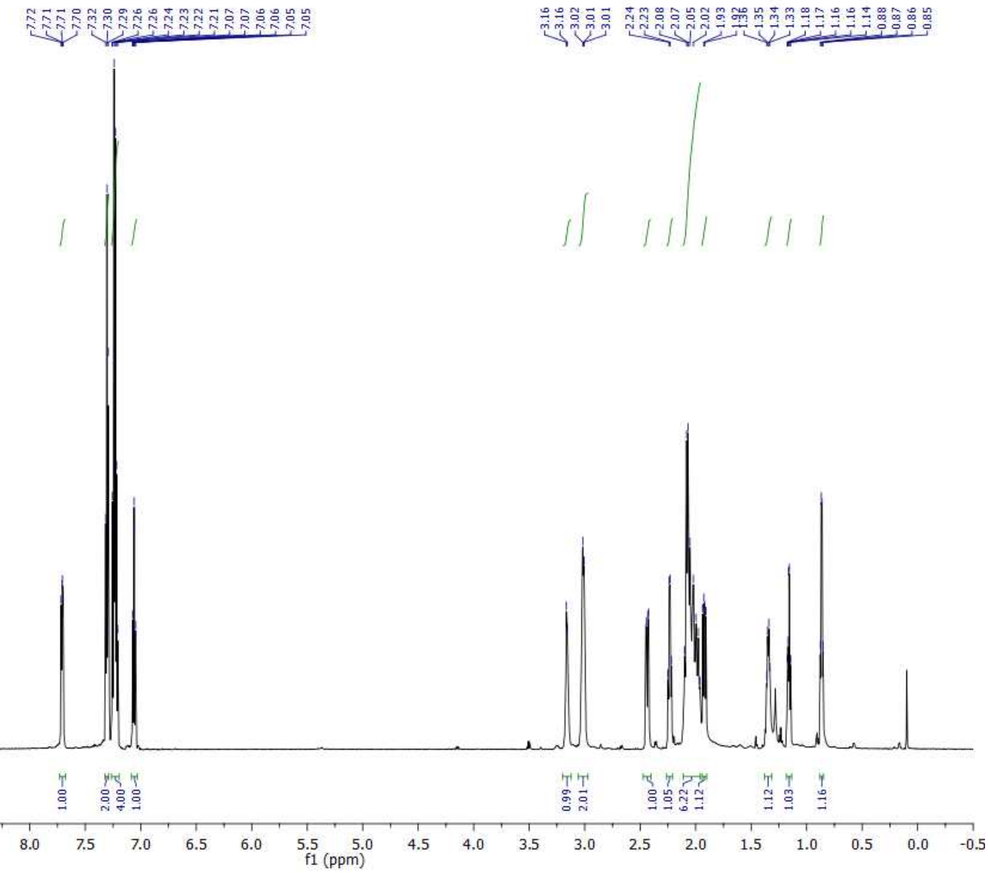
7.72 7.71 7.71 7.70 7.32 7.30 7.29 7.26 7.26 7.24 7.23 7.23 7.22 7.21 7.07 7.07 7.06 7.05 7.05
3.16 3.16 3.02 3.01 3.01 2.24 2.23 2.08 2.07 2.05 2.02 1.93 1.92 1.36 1.35 1.34 1.33 1.18 1.17 1.16 1.16 1.14 0.88 0.87 0.86 0.85
1.00 2.00 4.00 1.00 0.99 2.01 1.00 1.05 6.22 1.12 1.12 1.03 1.16
f1 (ppm)

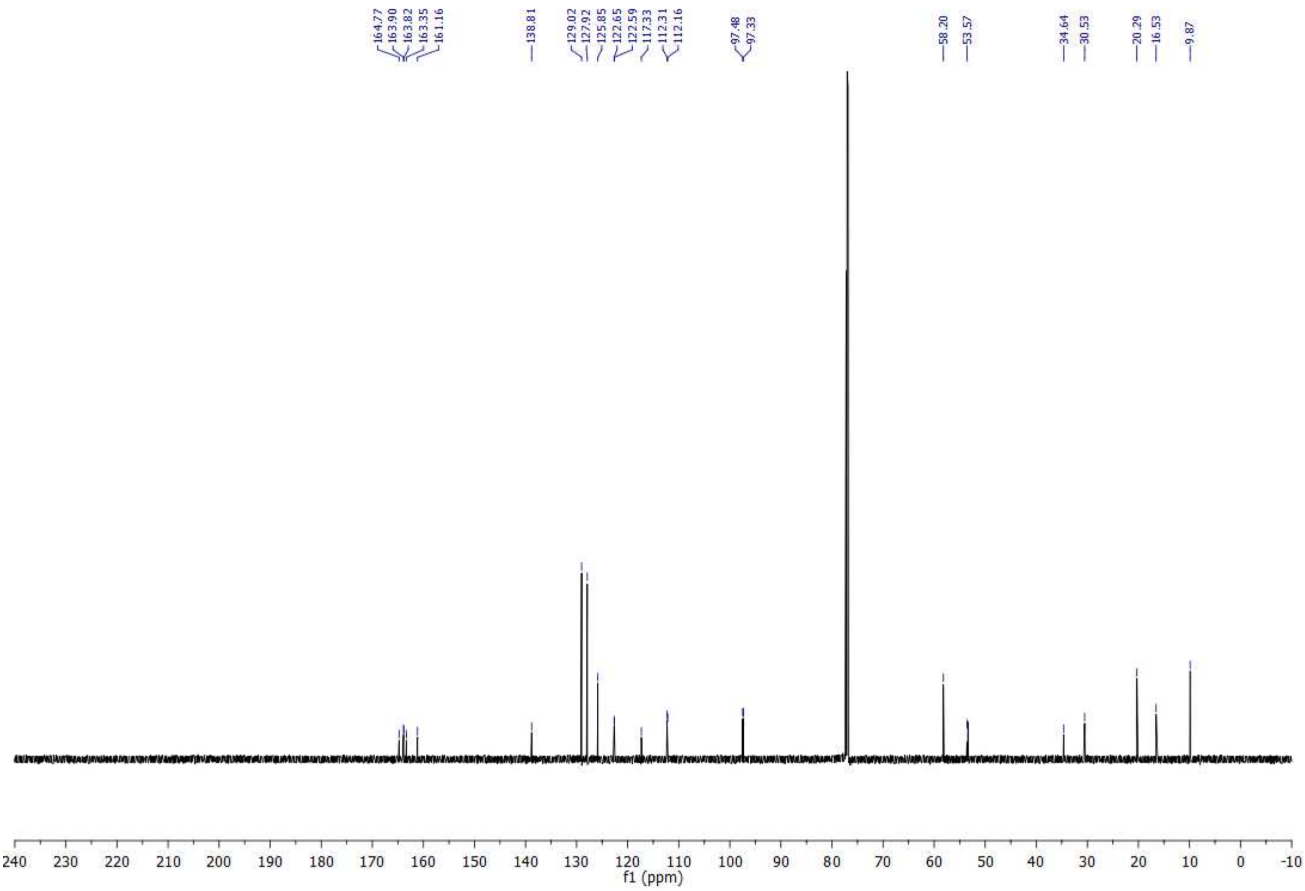
164.77 163.90 163.82 163.35 161.16 138.81 129.02 127.92 125.85 122.65 122.59 117.33 112.31 112.16 97.48 97.33 58.20 53.57 34.64 30.53 20.29 16.53 9.87
f1 (ppm)

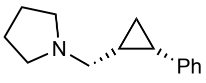
N
Ph

3i

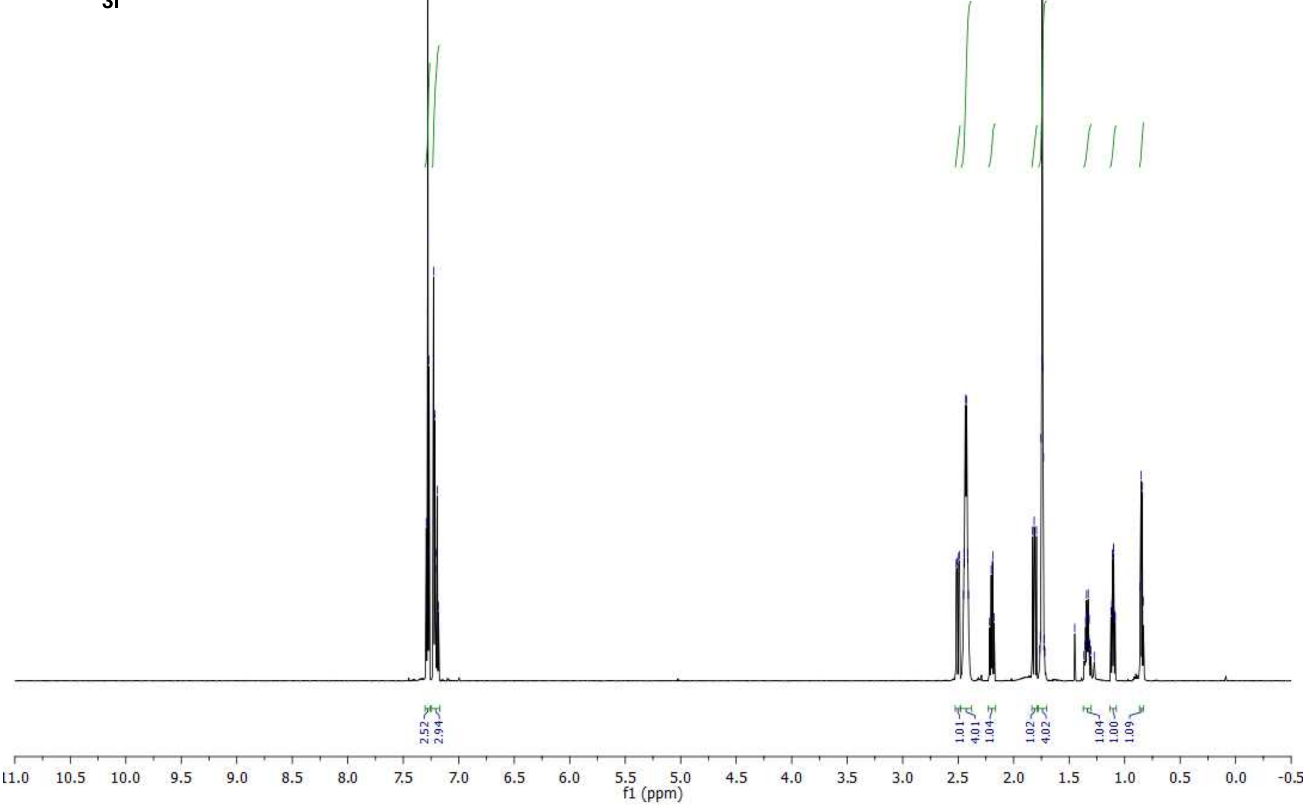
7.29 7.29 7.28 7.28 7.27 7.27 7.23 7.21 7.21 7.21 7.19 7.18 7.18
2.52 2.51 2.50 2.49 2.45 2.45 2.44 2.42 2.41 2.41 2.22 2.20 2.19 2.19 2.18
1.83 1.82 1.81 1.79 1.75 1.75 1.74 1.74 1.73 1.34 1.33 1.11 1.11 1.10 1.10 0.86 0.85 0.84
2.52
2.94
1.01
4.01
1.04
1.02
4.02
1.04
1.00
1.09
f1 (ppm)

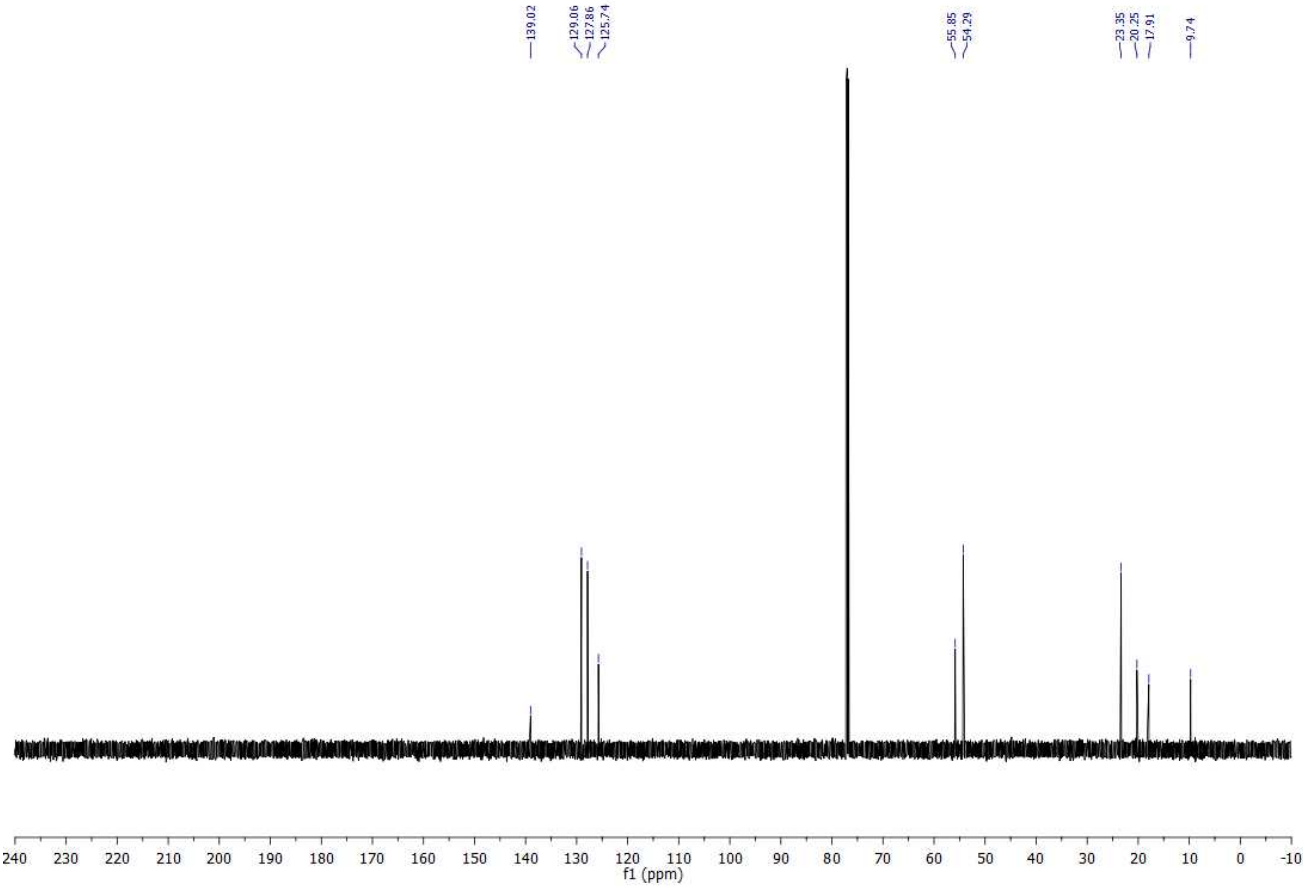
139.02
129.06
127.86
125.74
55.85
54.29
23.35
20.25
17.91
9.74
f1 (ppm)

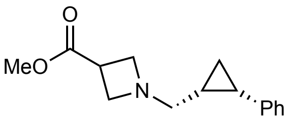
O
MeO
N
Ph

3j

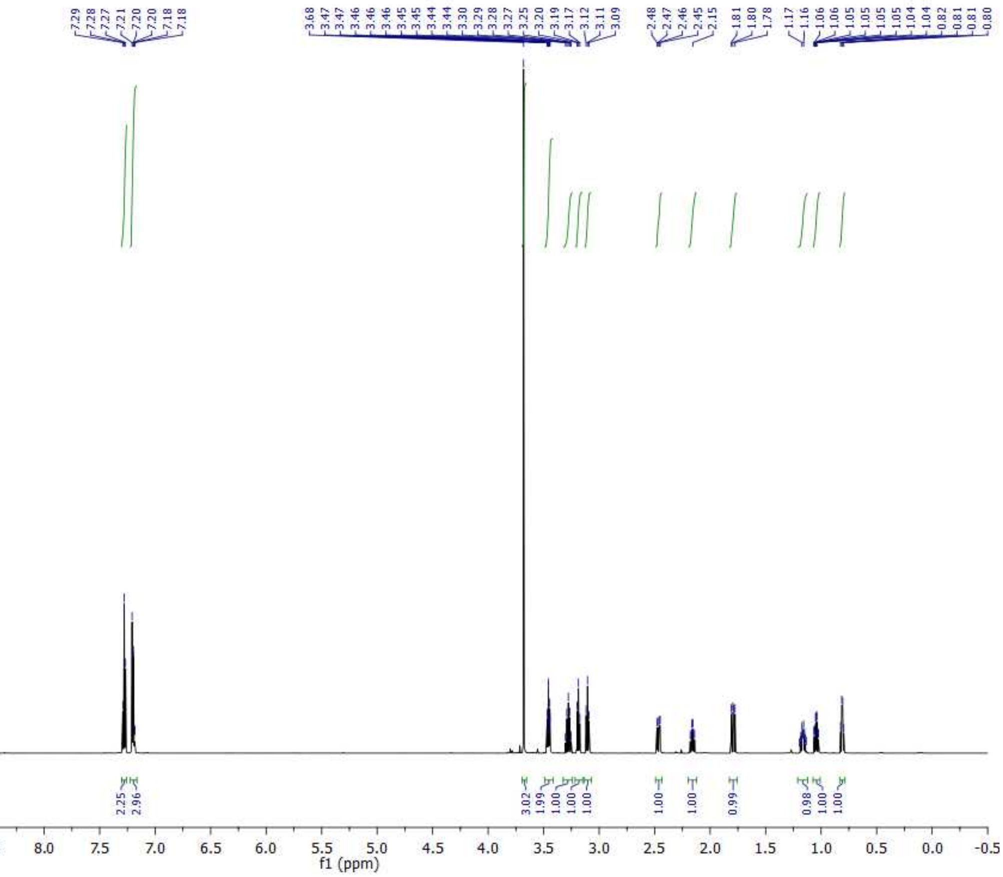
7.29 7.28 7.27 7.21 7.20 7.20 7.18 7.18
3.68 3.47 3.46 3.46 3.45 3.45 3.45 3.44 3.30 3.29 3.28 3.27 3.25 3.20 3.19 3.17 3.12 3.11 3.09
2.48 2.47 2.46 2.45 2.15 1.81 1.80 1.78
1.17 1.16 1.06 1.06 1.05 1.05 1.05 1.05 1.04 1.04 0.82 0.81 0.80
2.25 2.96
3.02 1.99 1.00 1.00 1.00
1.00 1.00 0.99
0.98 1.00 1.00
f1 (ppm)

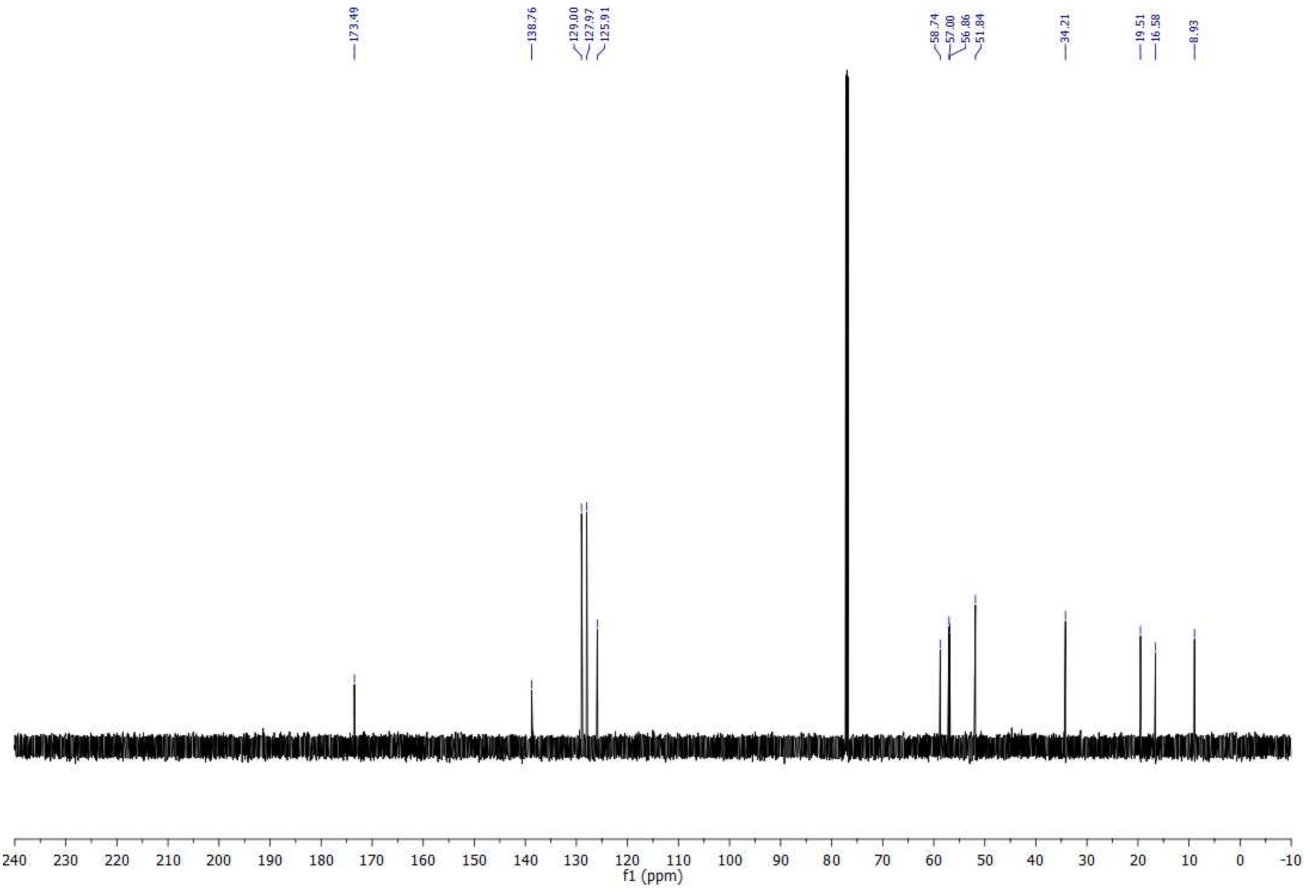
173.49
138.76
129.00 127.97 125.91
58.74 57.00 56.86 51.84
34.21
19.51 16.58
8.93
f1 (ppm)

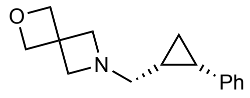

3k

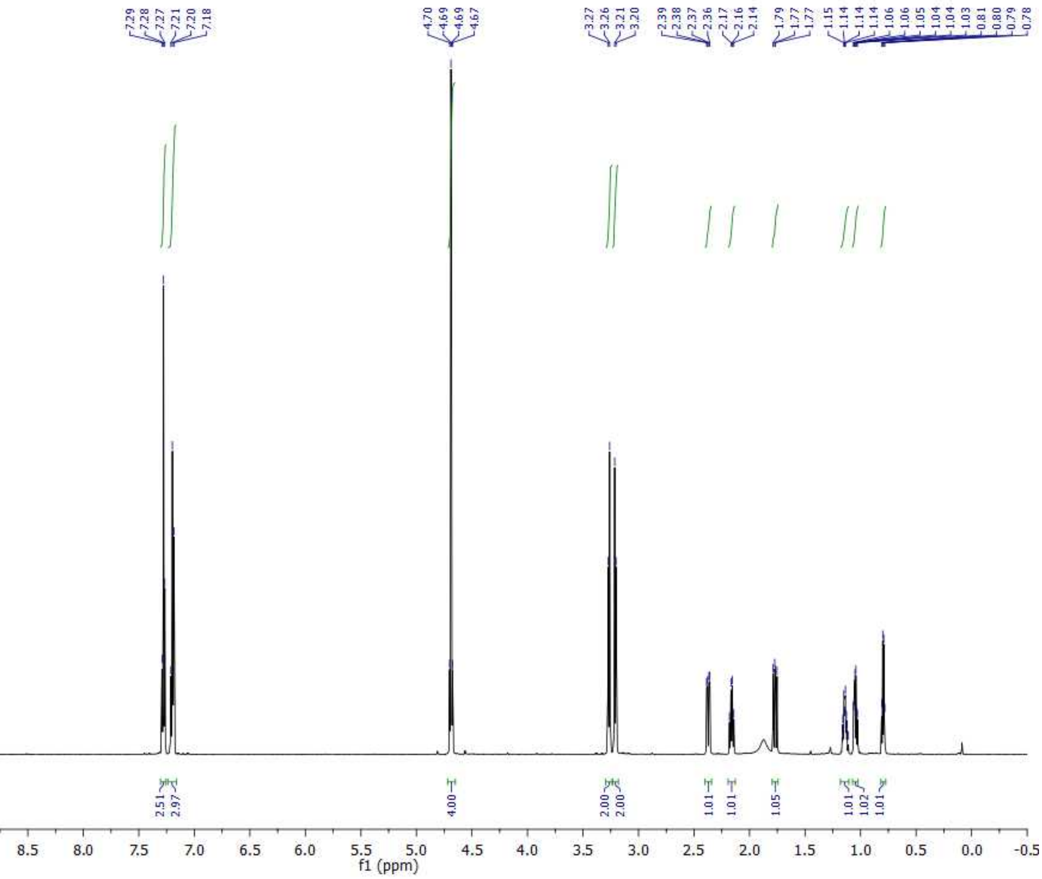

7.29 7.28 7.27 7.21 7.20 7.18
4.70 4.69 4.69 4.67
3.27 3.26 3.21 3.20
2.39 2.38 2.37 2.36 2.17 2.16 2.14
1.79 1.77 1.77
1.15 1.14 1.14 1.14 1.06 1.06 1.05 1.04 1.04 1.03 0.81 0.80 0.79 0.78
2.51 2.97 4.00 2.00 2.00 1.01 1.01 1.05 1.01 1.02 1.01
f1 (ppm)

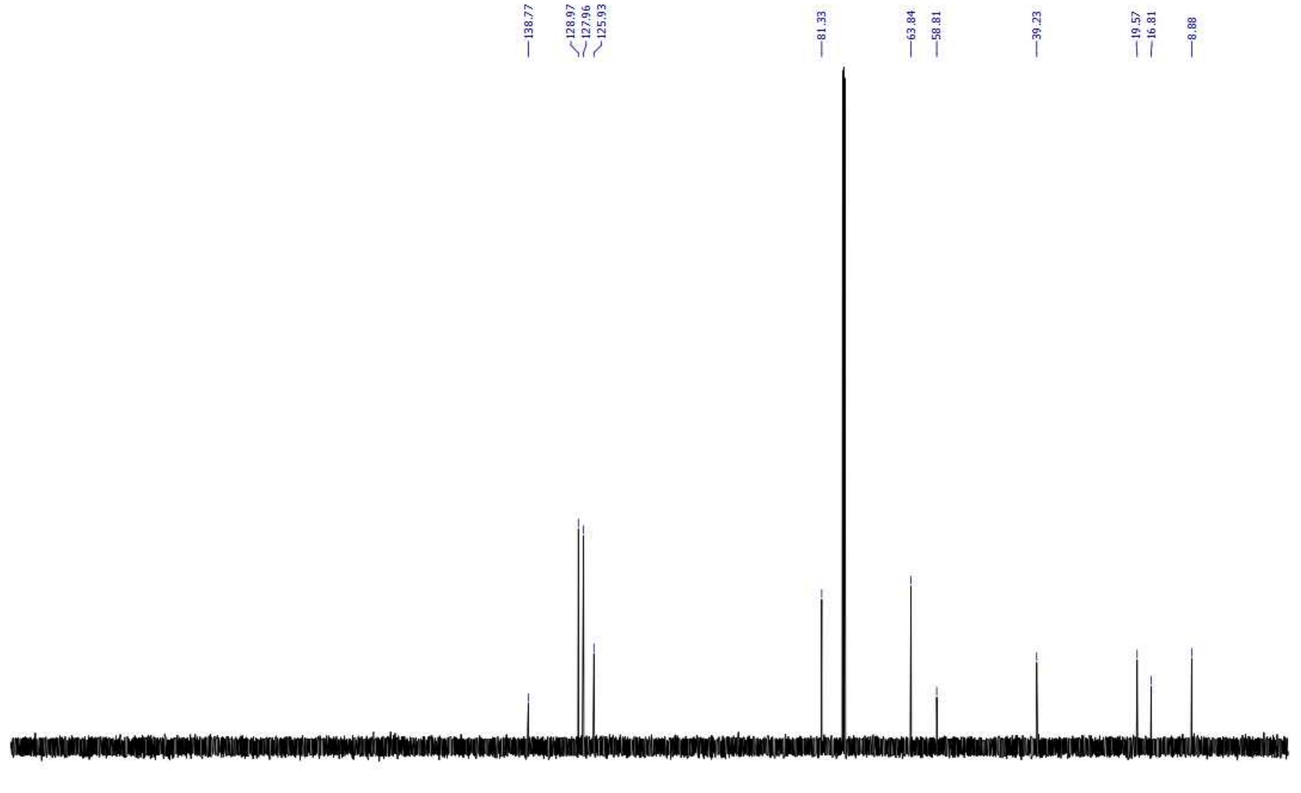

138.77
128.97 127.96 125.93
81.33
63.84 58.81
39.23
19.57 16.81
8.88
f1 (ppm)

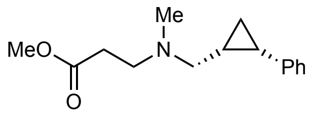

Me
MeO
N
Ph
O

3m

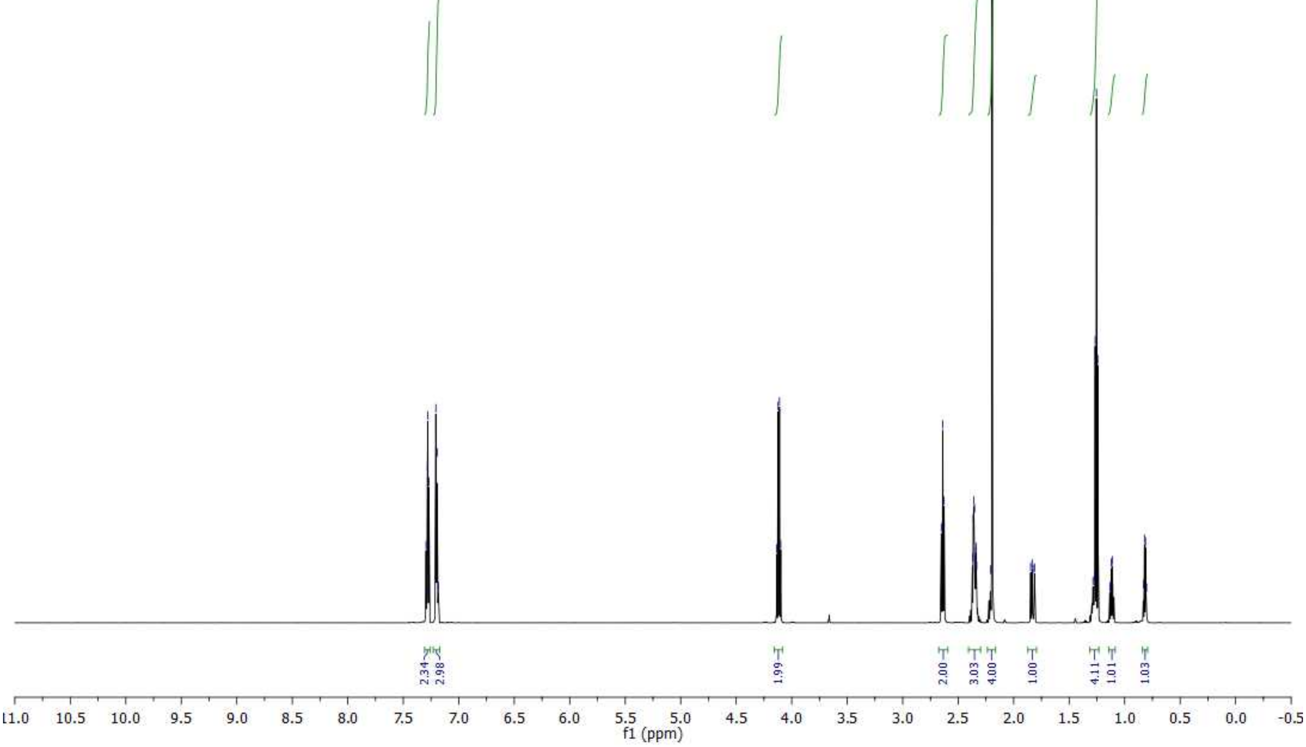

7.29 7.28 7.28 7.28 7.27 7.21 7.20 7.19 7.19 7.18
4.13 4.12 4.11 4.10
2.65 2.64 2.63 2.37 2.36 2.36 2.35 2.35 2.34 2.34 2.33 2.21 2.19
1.85 1.83 1.81
1.27 1.25 1.24 1.11 0.83 0.82 0.81 0.80
f1 (ppm)

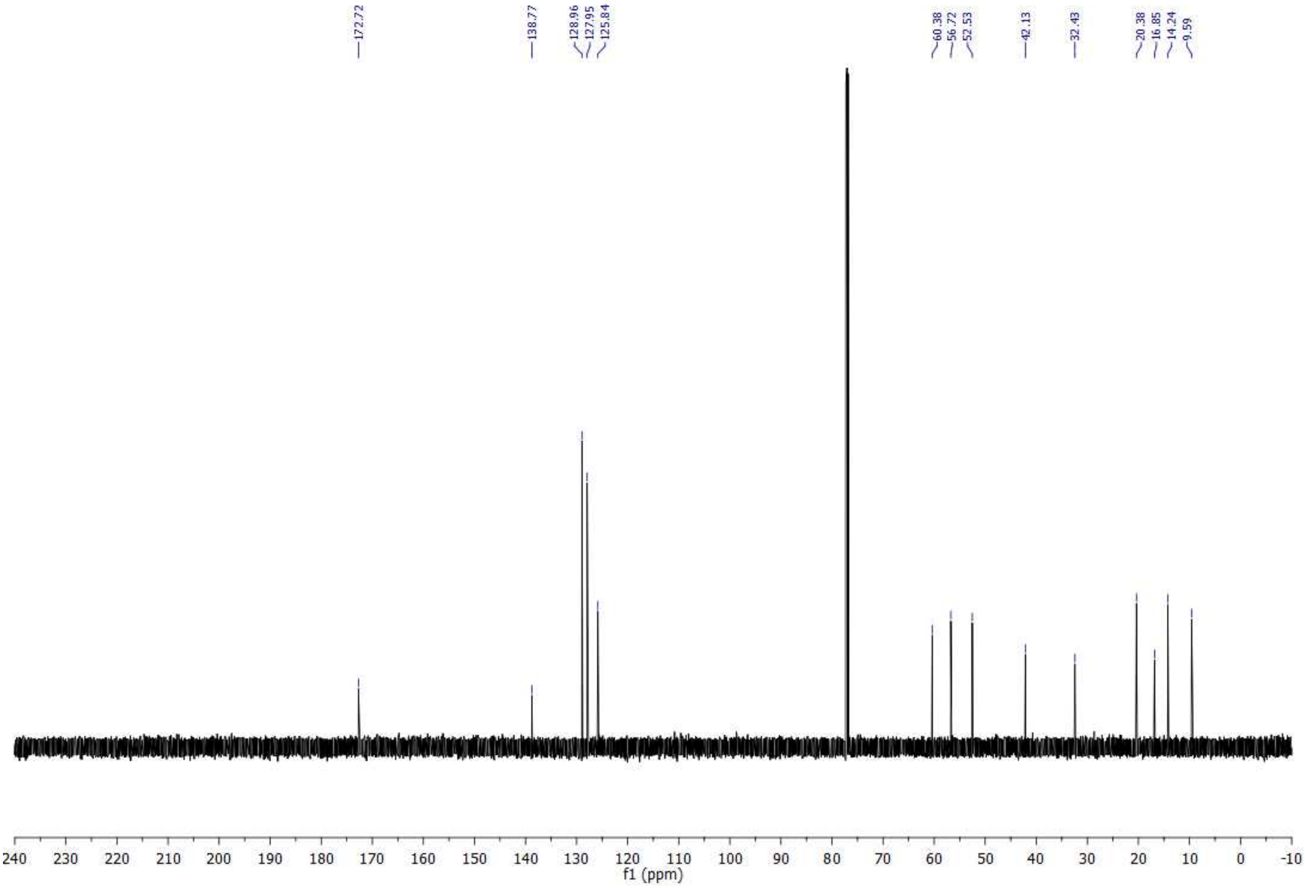

172.72
138.77
128.96 127.95 125.84
60.38 56.72 52.53
42.13
32.43
20.38 16.85 14.24 9.59
f1 (ppm)

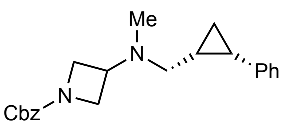
Me
N
Ph
N
Cbz

3n

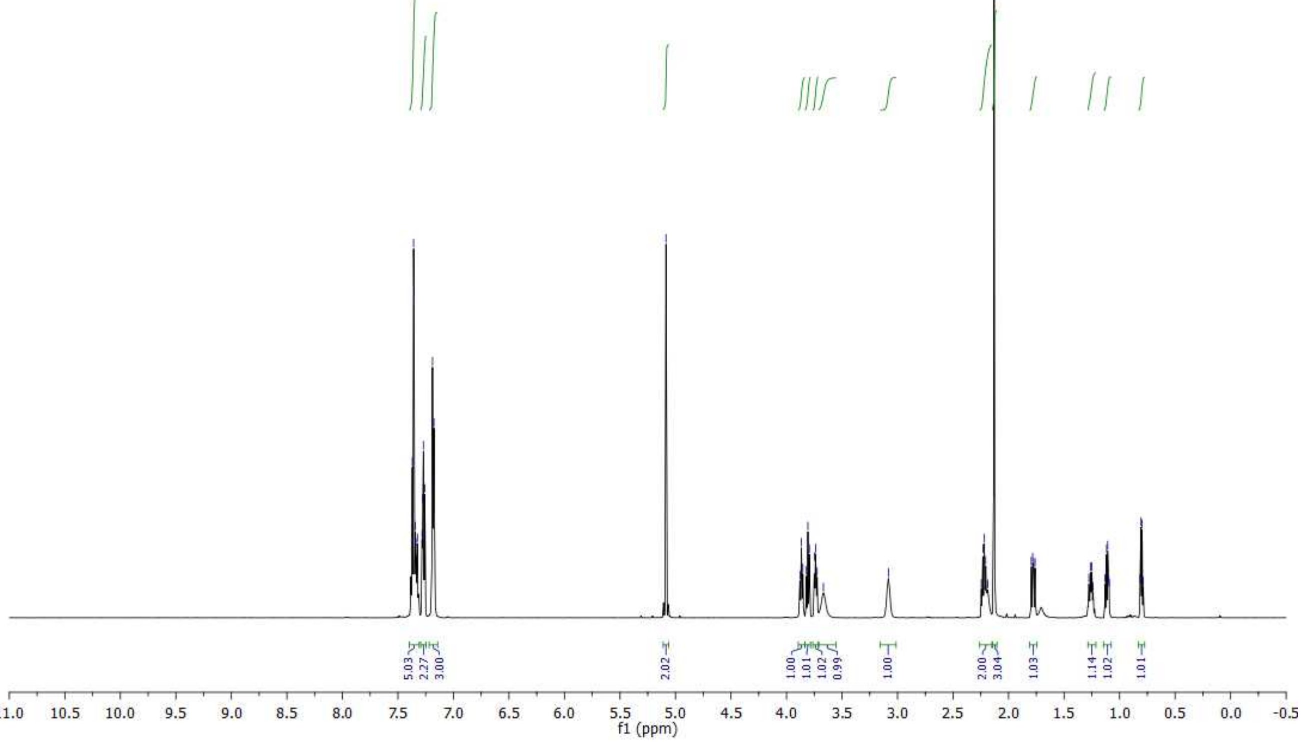
7.37 7.36 7.36 7.34 7.34 7.32 7.28 7.28 7.27 7.26 7.19 7.18
5.09
3.88 3.87 3.85 3.82 3.81 3.79 3.75 3.74 3.72 3.67 3.08
2.24 2.23 2.22 2.21 2.19 2.13
1.80 1.78 1.76 1.26 1.25 1.12 1.11 0.82 0.81 0.80 0.79
5.03 2.27 3.00
2.02
1.00 1.01 1.02 0.99
1.00
2.00 3.04
1.03
1.14 1.02
1.01
11.0 10.5 10.0 9.5 9.0 8.5 8.0 7.5 7.0 6.5 6.0 5.5 5.0 4.5 4.0 3.5 3.0 2.5 2.0 1.5 1.0 0.5 0.0 -0.5
f1 (ppm)

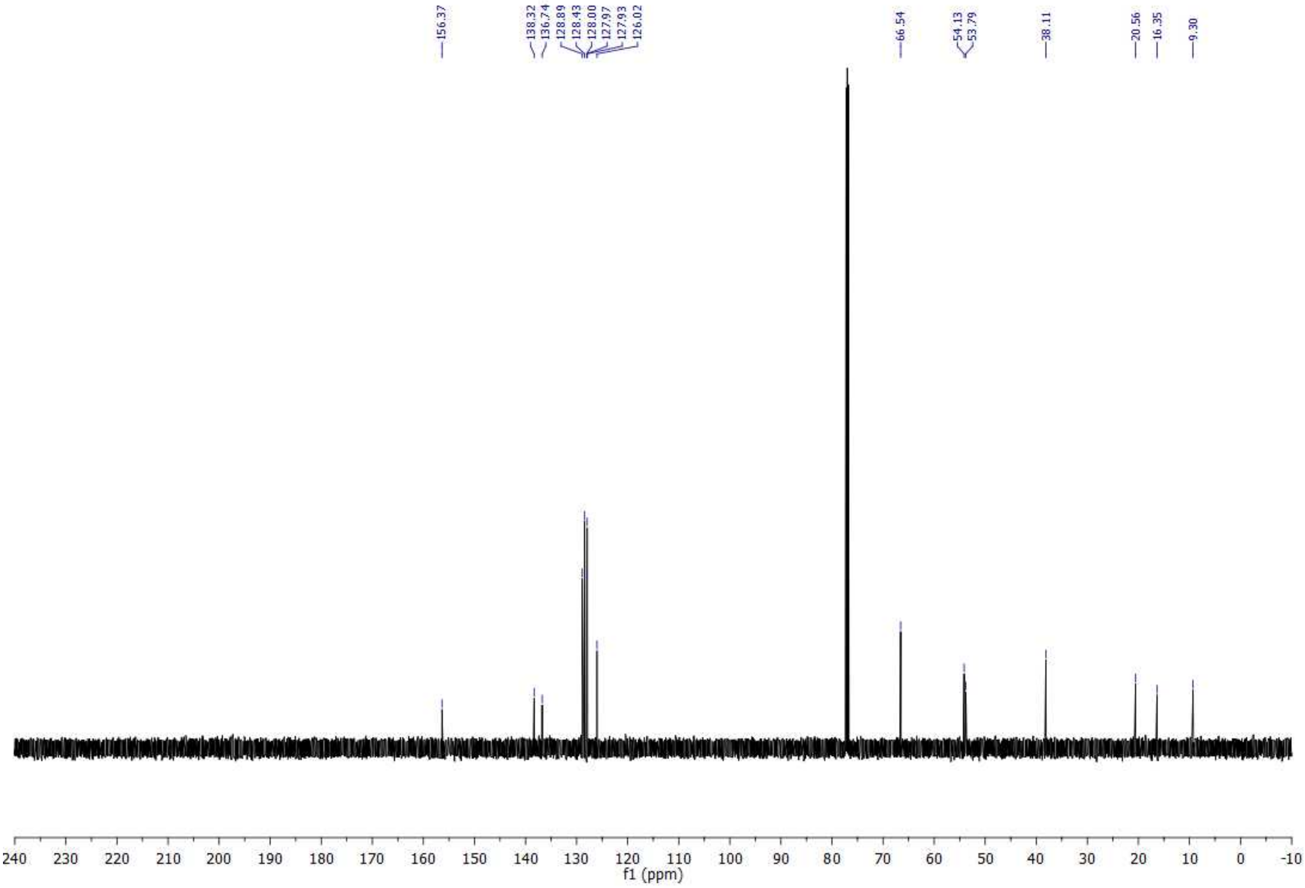
156.37
138.32 136.74 128.89 128.43 128.00 127.97 127.93 126.02
66.54
54.13 53.79
38.11
20.56 16.35 9.30
240 230 220 210 200 190 180 170 160 150 140 130 120 110 100 90 80 70 60 50 40 30 20 10 0 -10
f1 (ppm)

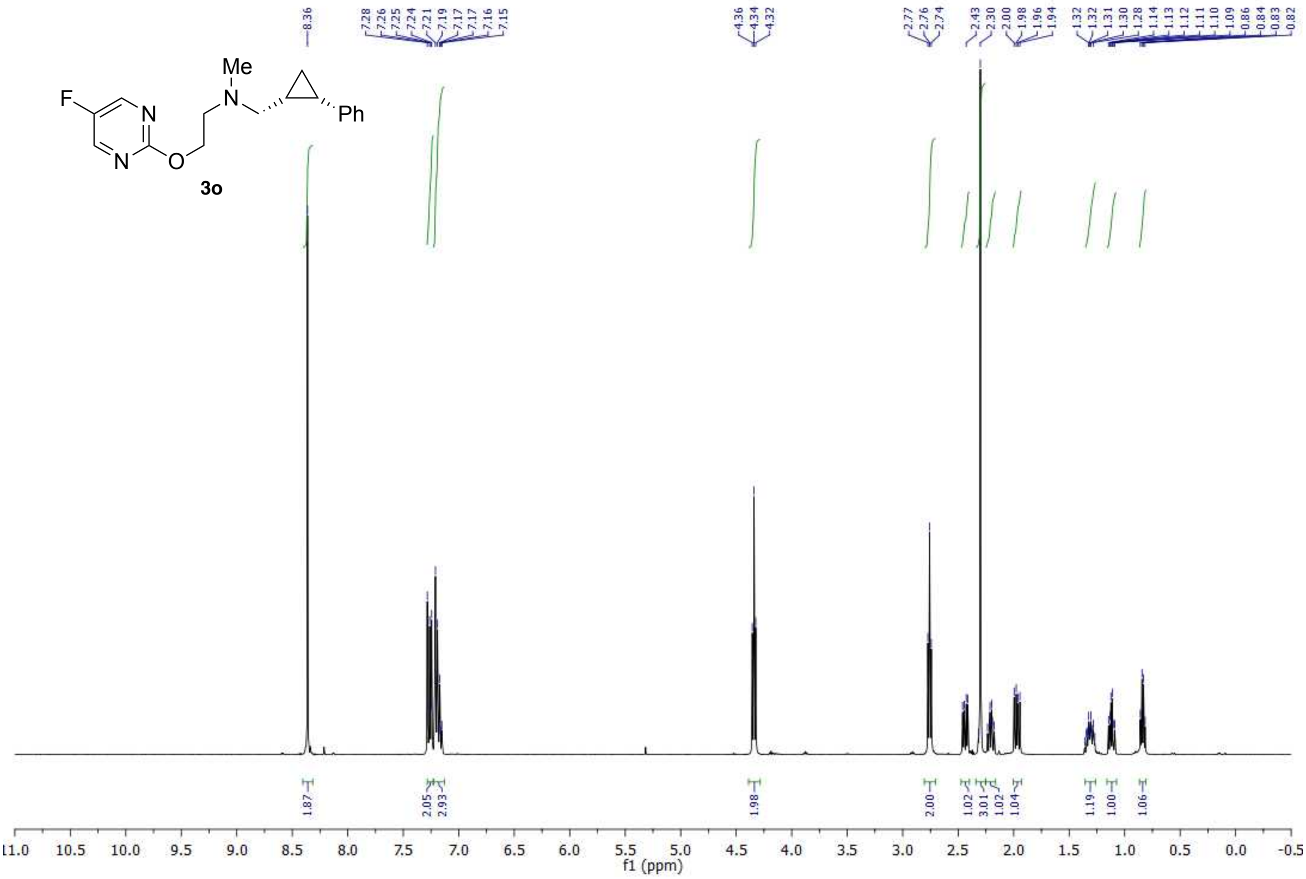

3o

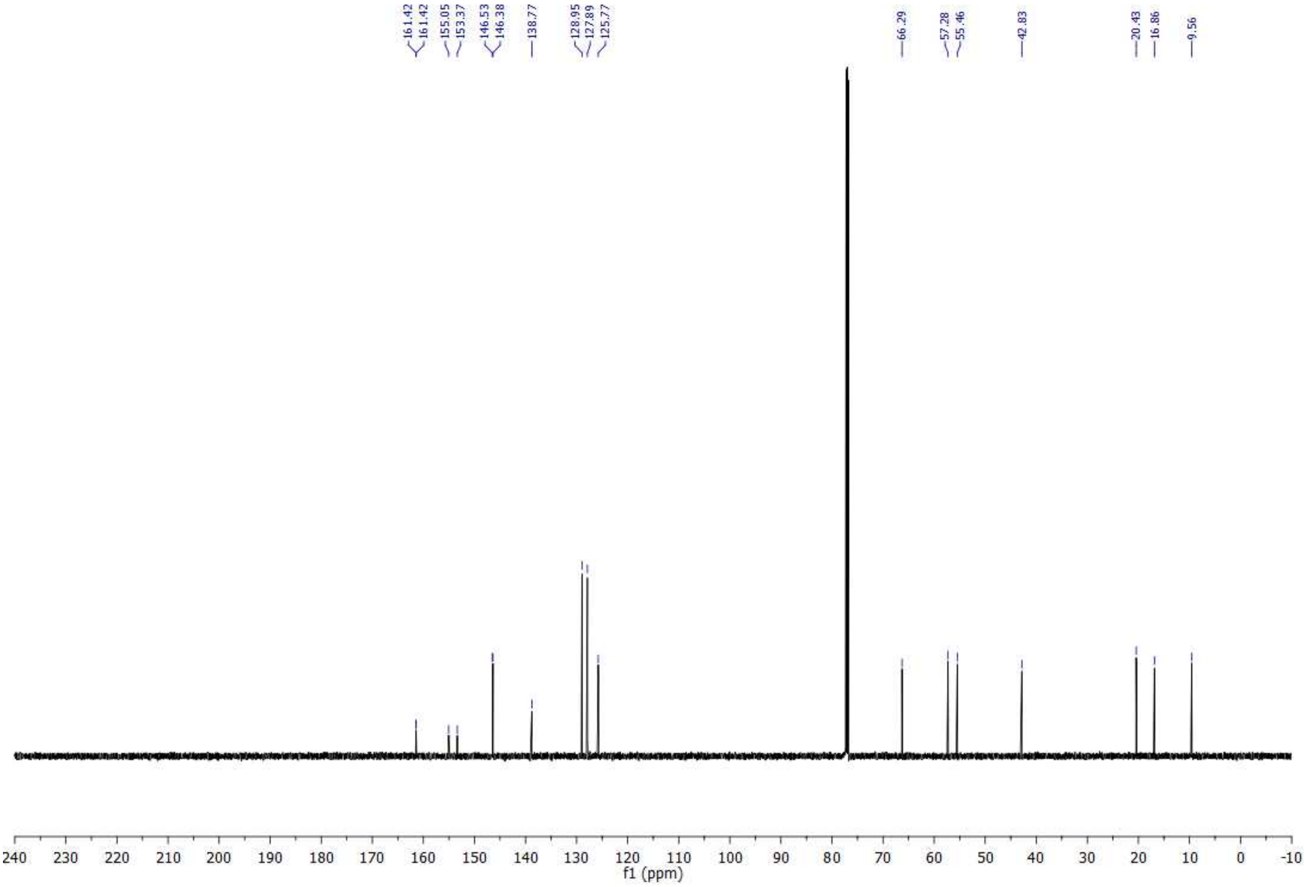

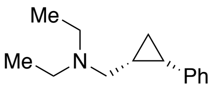
Me
Me
N
Ph

3p

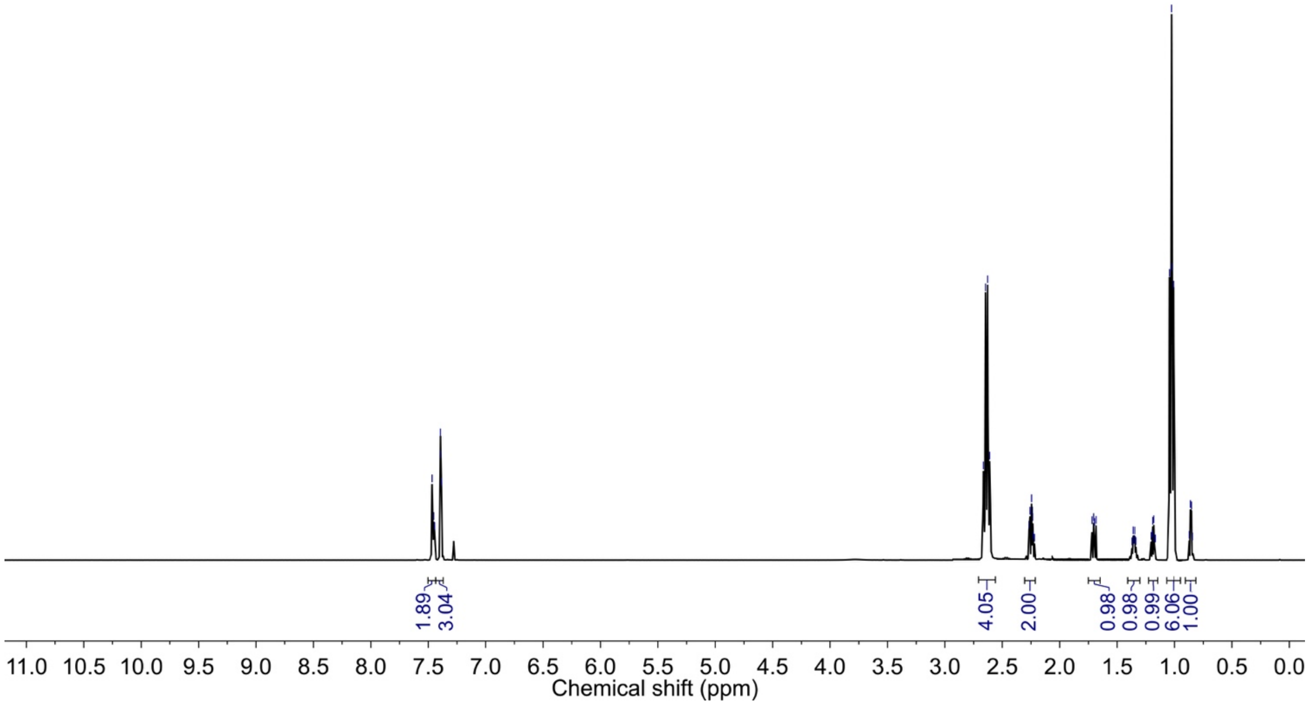
7.47
7.45
7.45
7.39
7.39
7.38
2.27
2.26
2.25
2.24
2.23
2.23
2.22
1.72
1.70
1.70
1.68
1.37
1.36
1.36
1.35
1.34
1.20
1.19
1.19
1.18
1.18
1.17
0.87
0.86
0.85
0.84
1.89
3.04
4.05
2.00
0.98
0.98
0.99
6.06
1.00
11.0 10.5 10.0 9.5 9.0 8.5 8.0 7.5 7.0 6.5 6.0 5.5 5.0 4.5 4.0 3.5 3.0 2.5 2.0 1.5 1.0 0.5 0.0
Chemical shift (ppm)

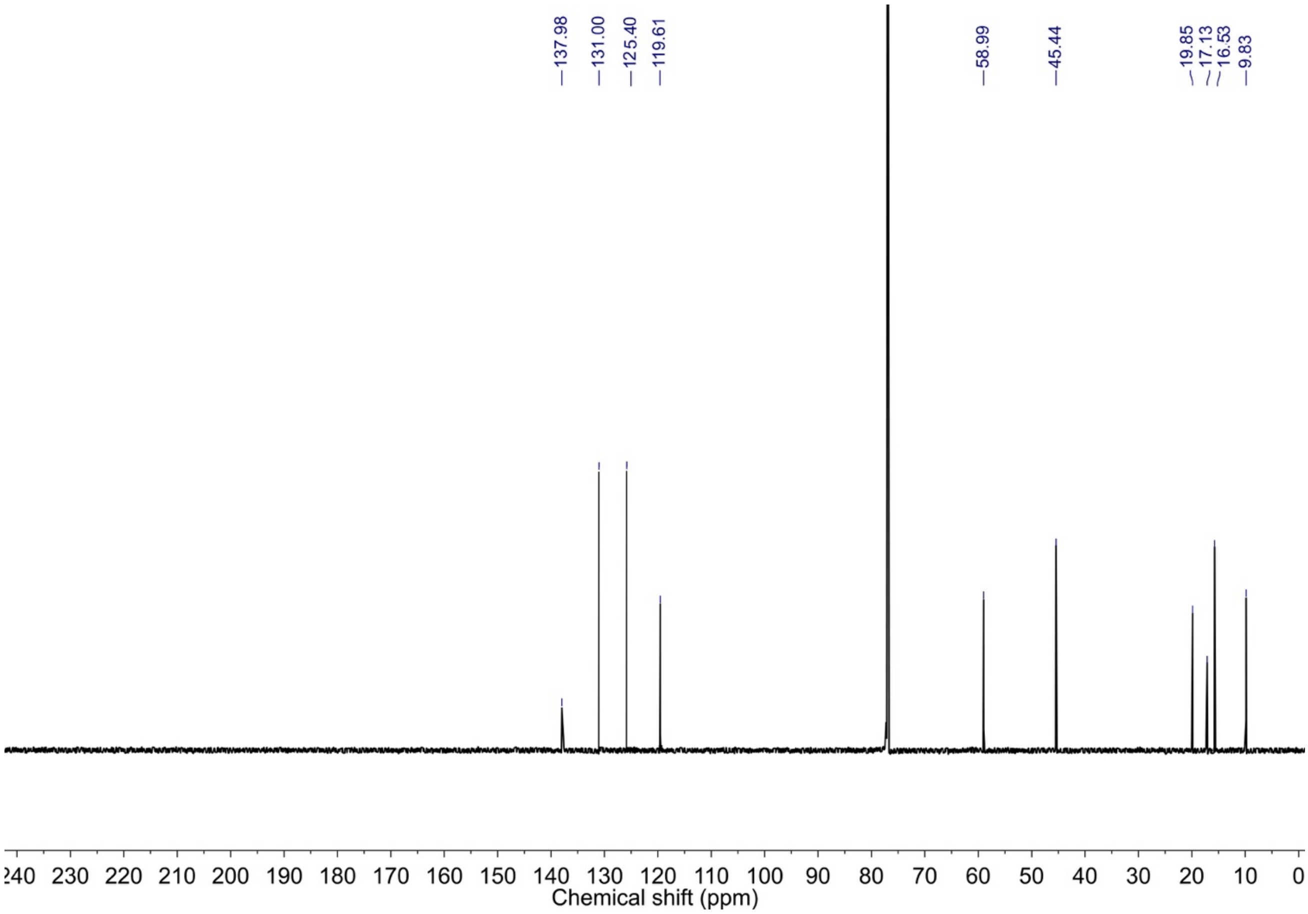
137.98
131.00
125.40
119.61
58.99
45.44
19.85
17.13
16.53
9.83
240 230 220 210 200 190 180 170 160 150 140 130 120 110 100 90 80 70 60 50 40 30 20 10 0
Chemical shift (ppm)

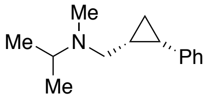

3q

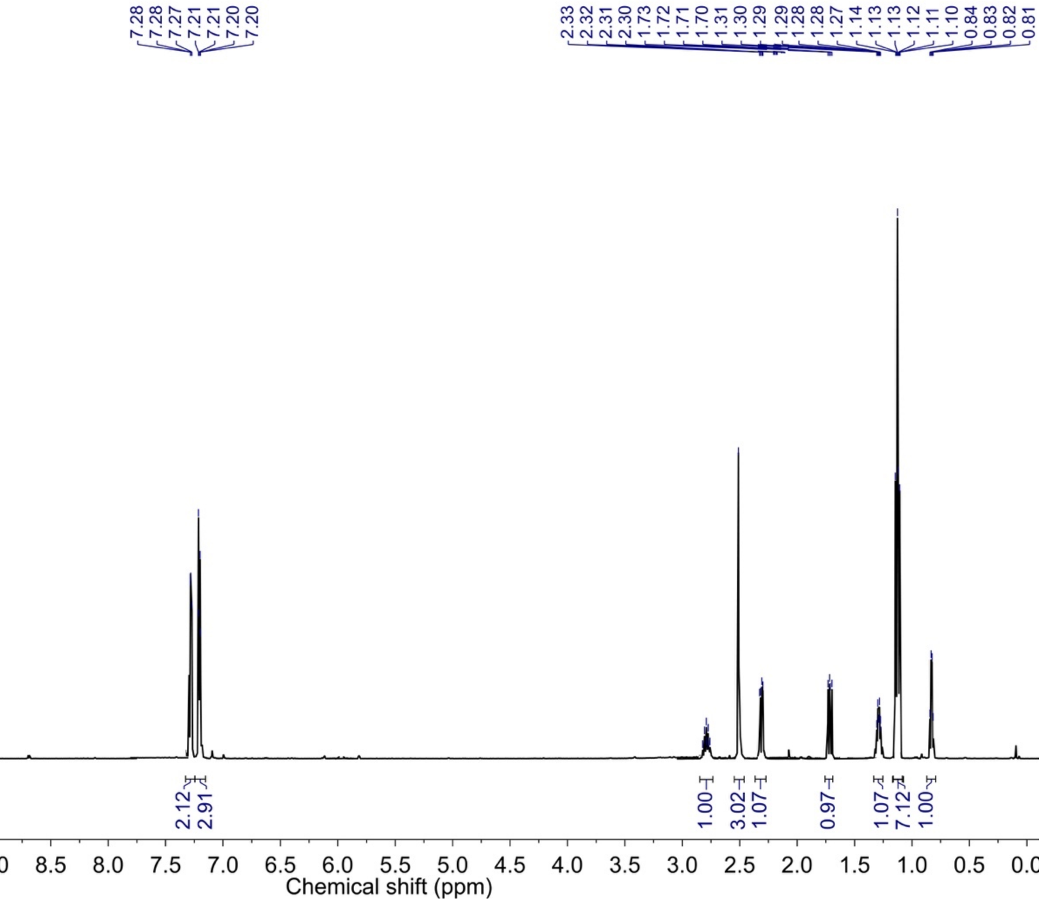

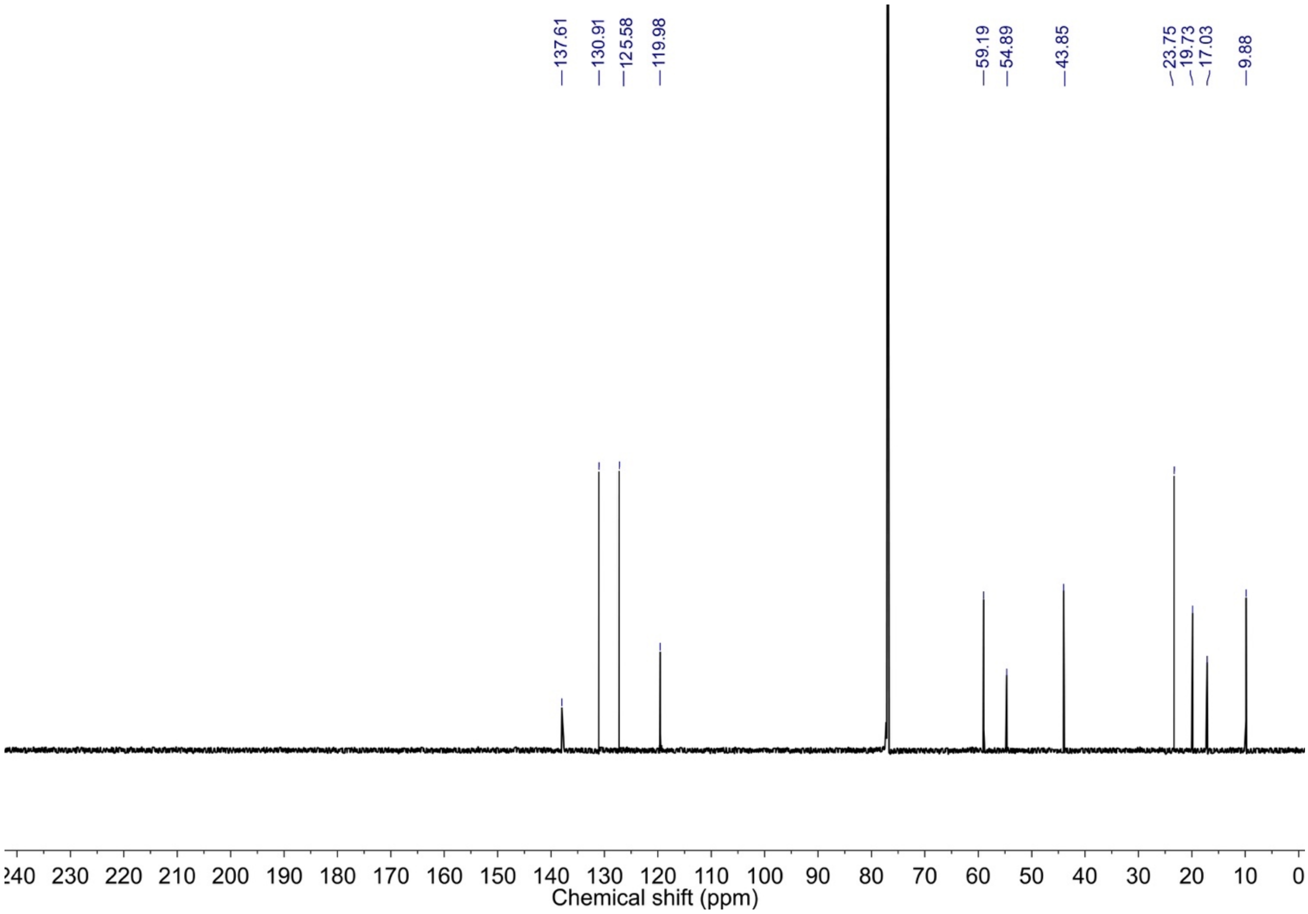

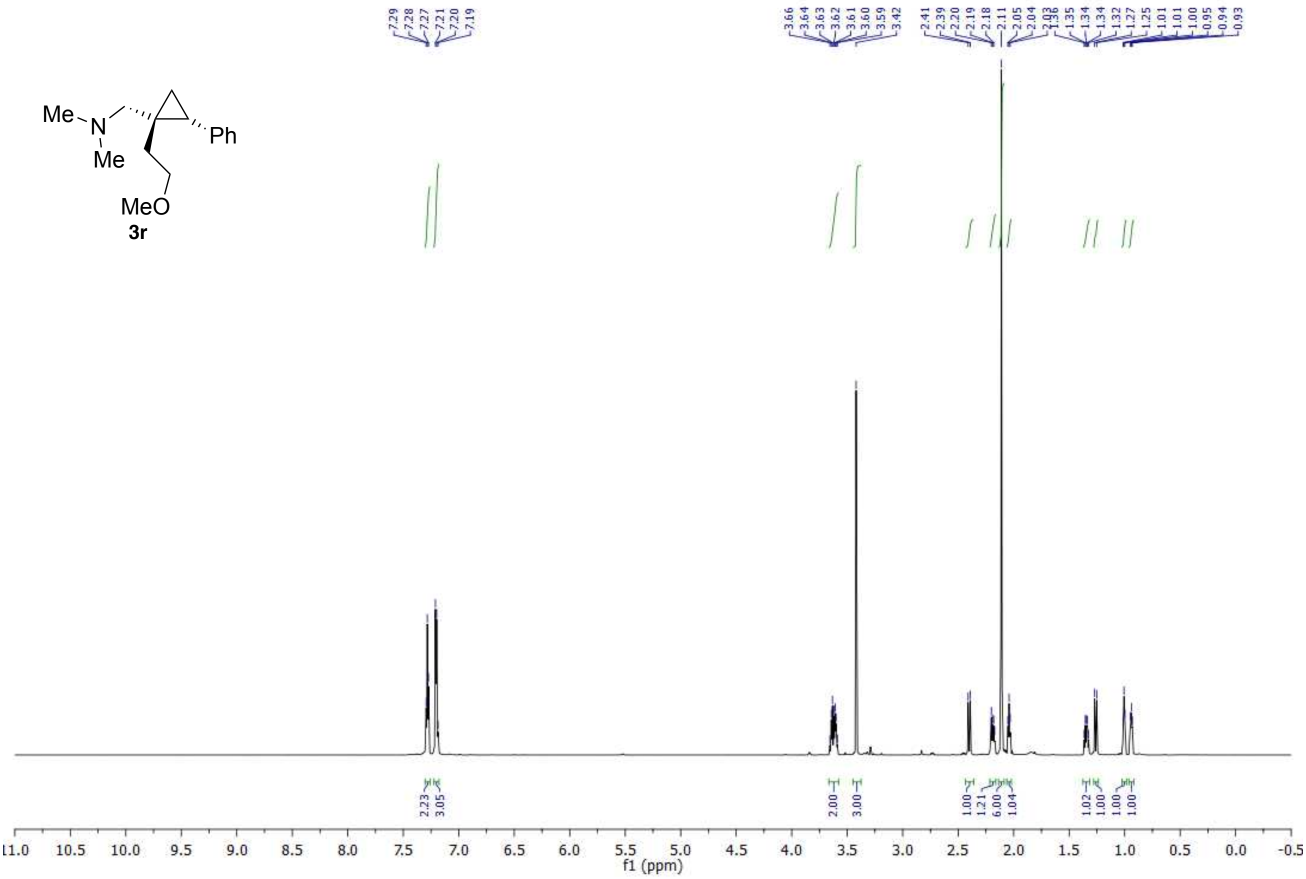
Me
N
Me
Ph
MeO
3r
f1 (ppm)

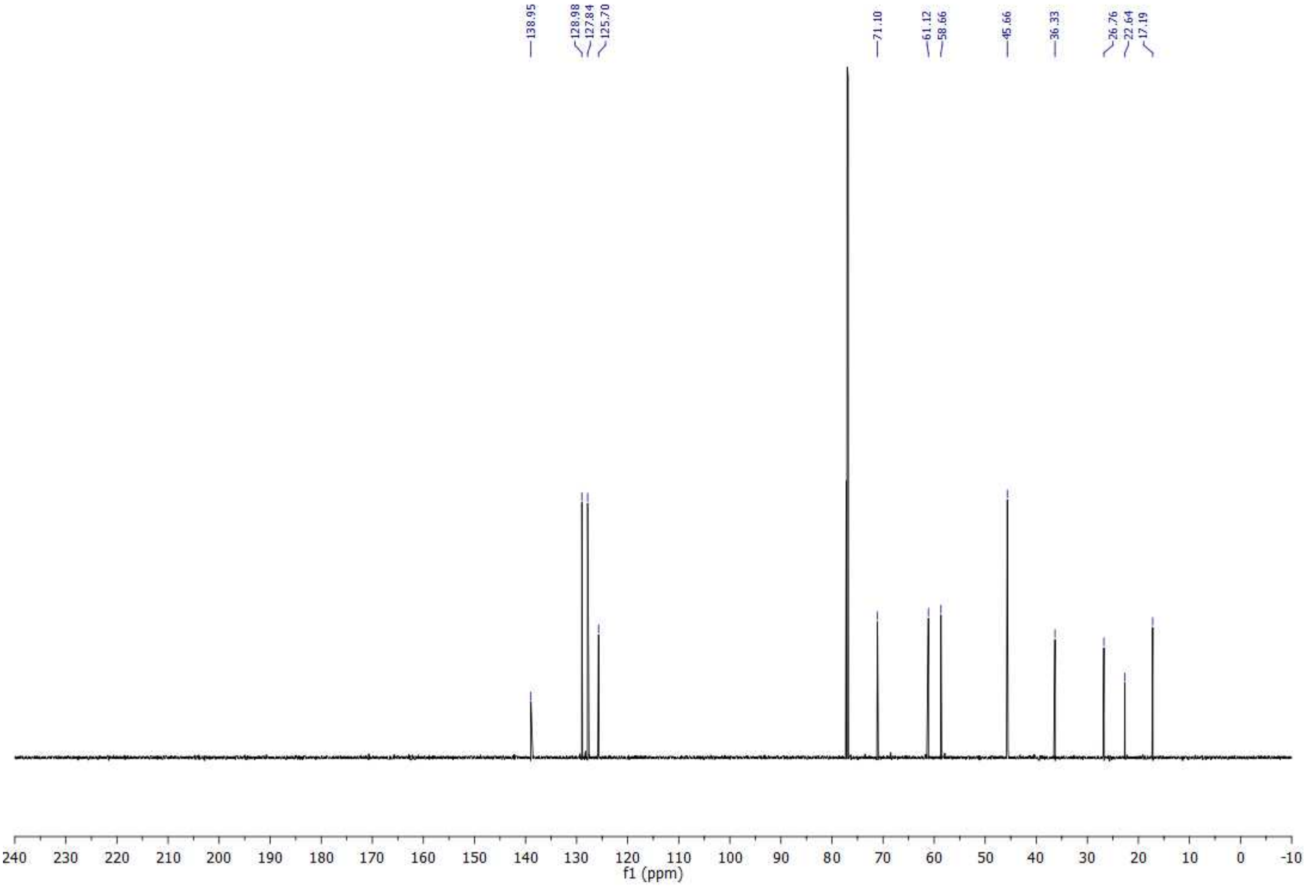
138.95
128.98
127.84
125.70
71.10
61.12
58.66
45.66
36.33
26.76
22.64
17.19
f1 (ppm)

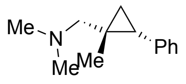
Me
N
Me
Me
Ph

3s

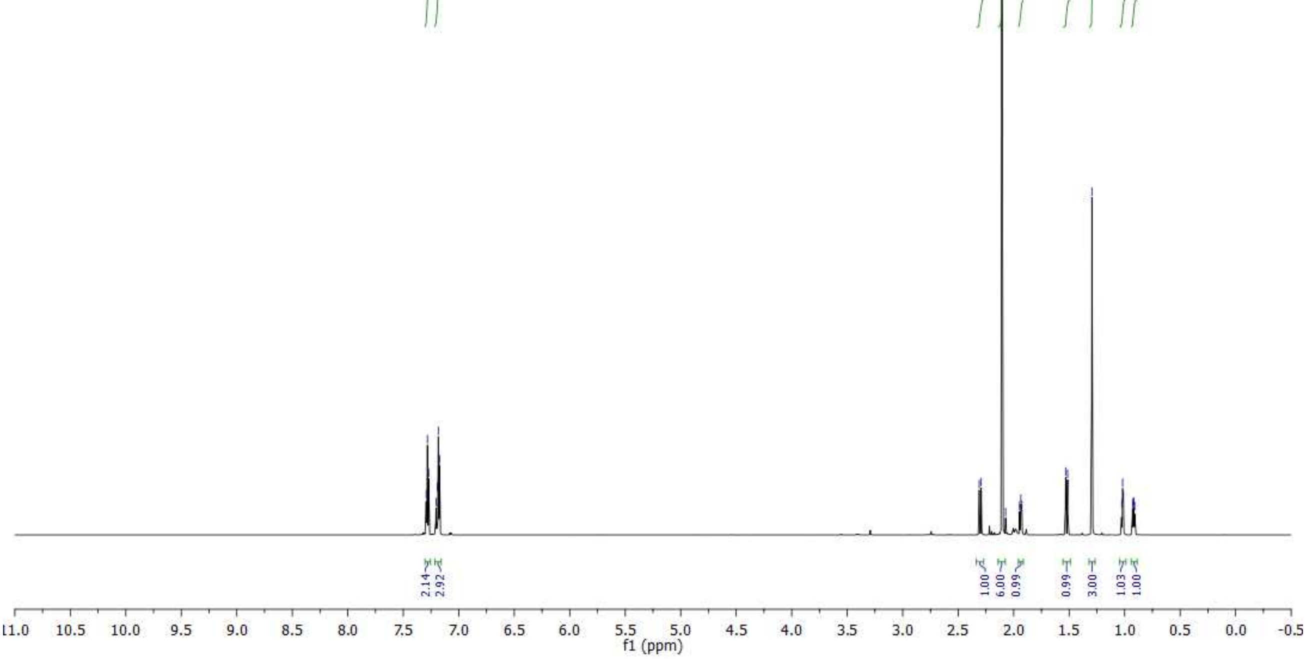
7.29
7.28
7.27
7.20
7.19
7.18
7.17
2.31
2.29
2.11
2.07
1.95
1.94
1.93
1.53
1.51
1.30
1.03
1.02
1.01
0.93
0.92
0.92
0.91
2.14
2.92
1.00
6.00
0.99
0.99
3.00
1.03
1.00
f1 (ppm)

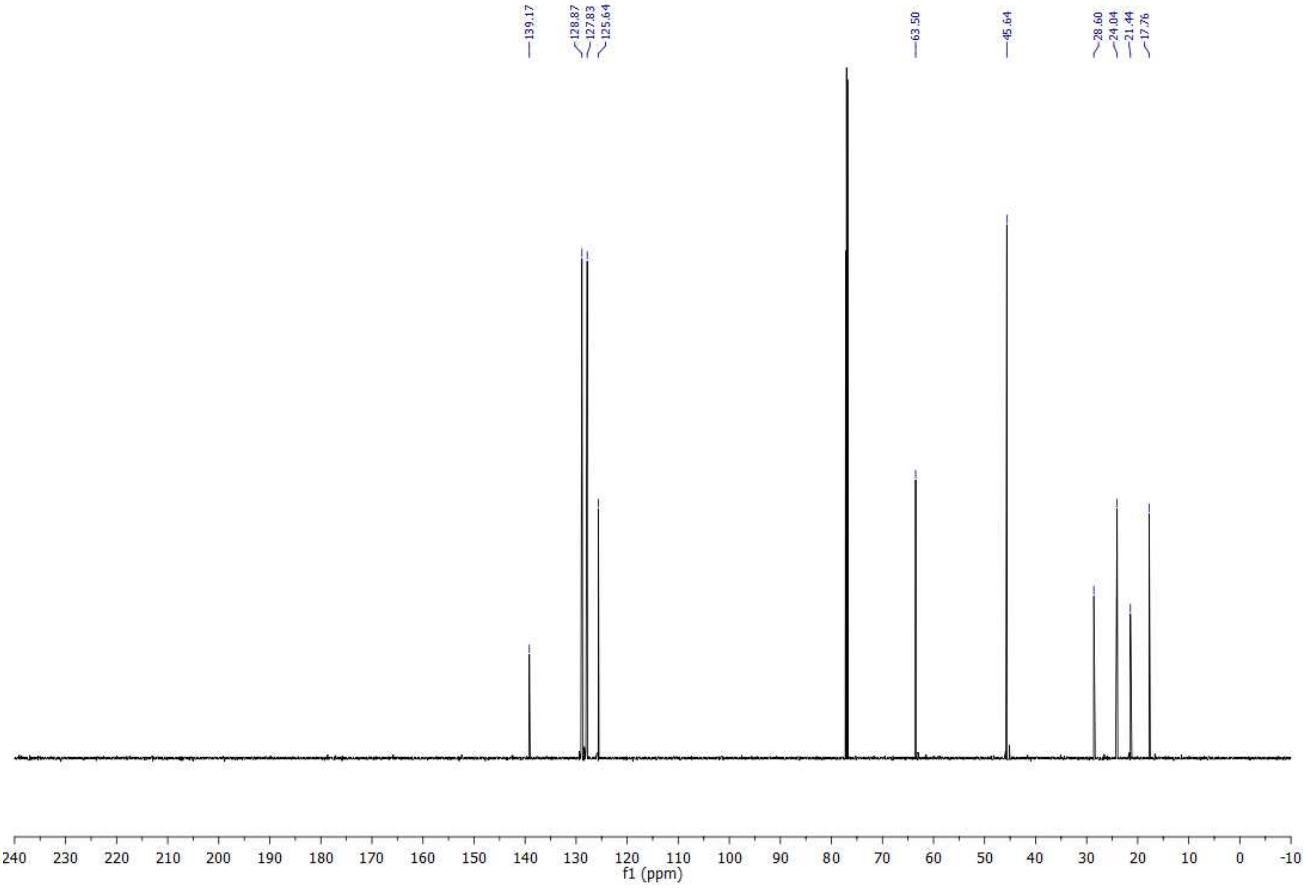
139.17
128.87
127.83
125.64
63.50
45.64
28.60
24.04
21.44
17.76
f1 (ppm)

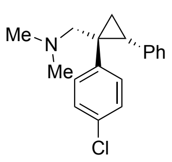

3t

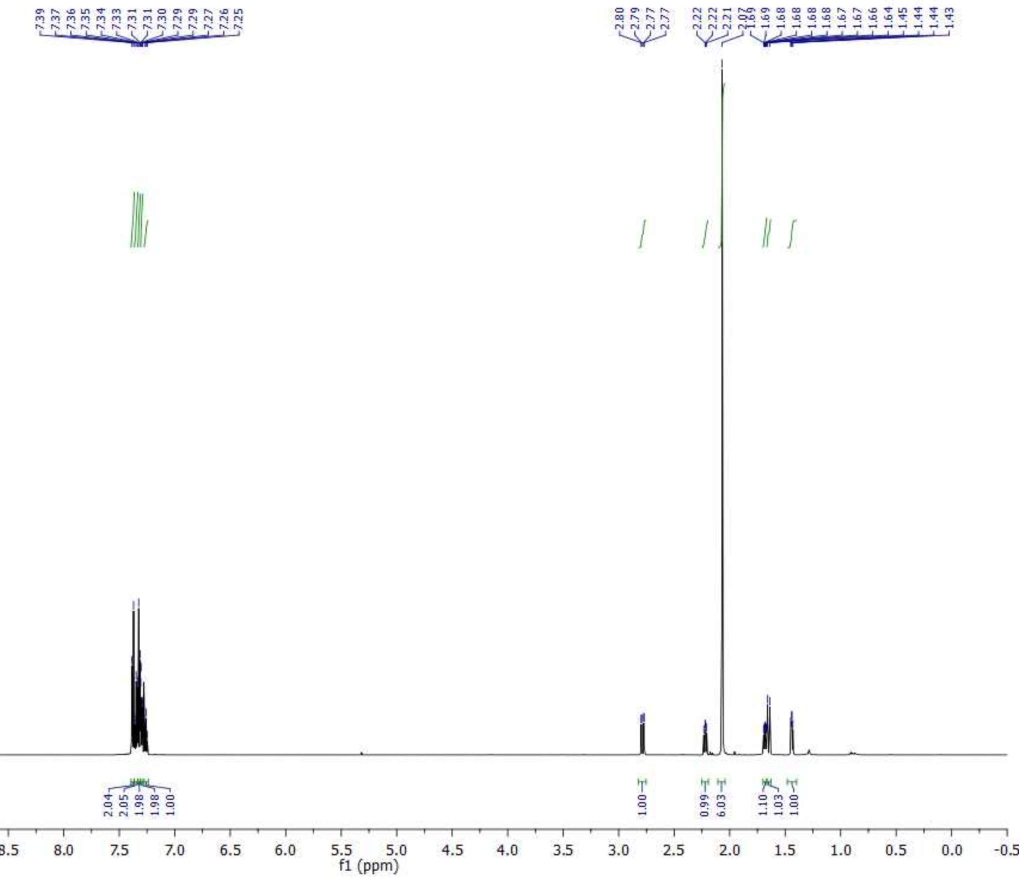

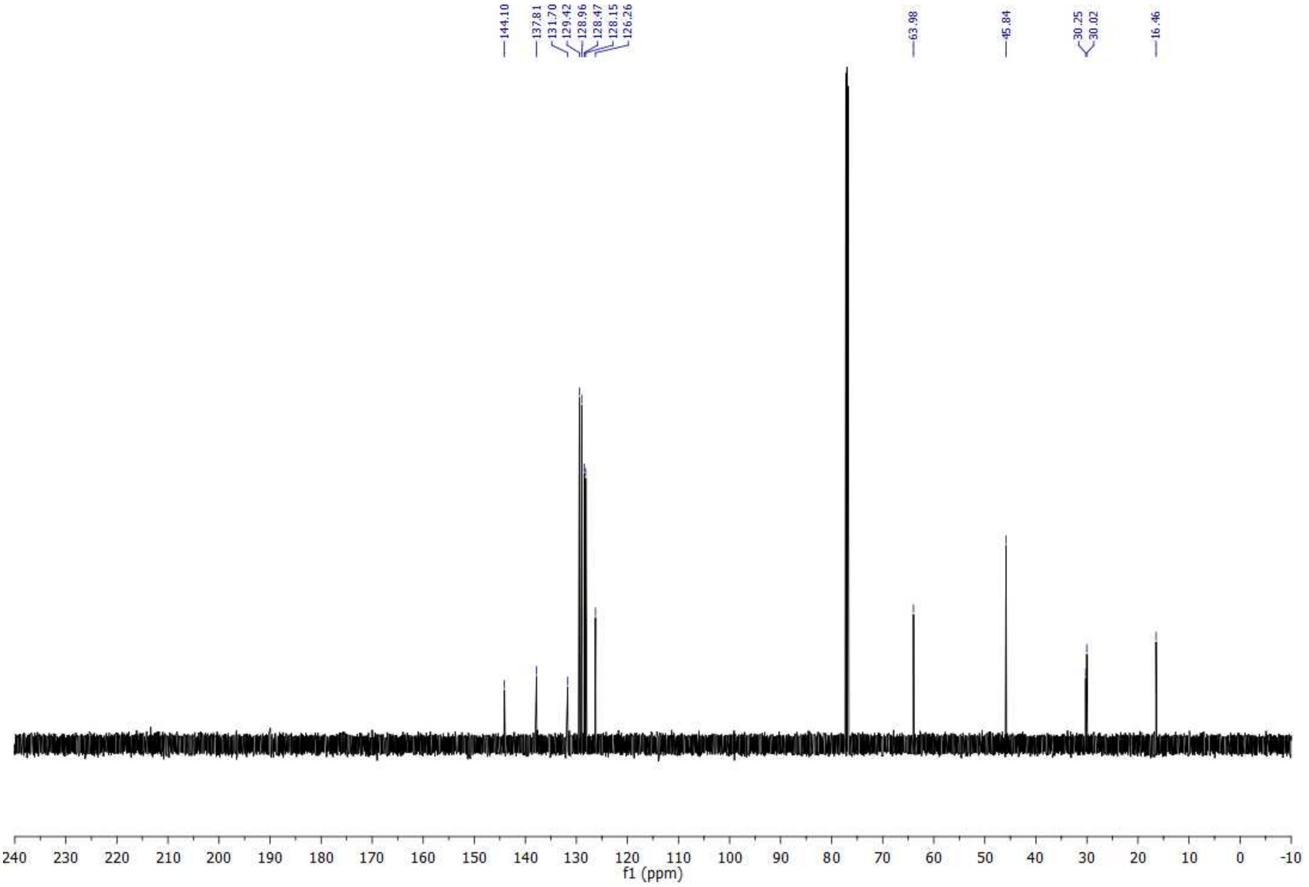

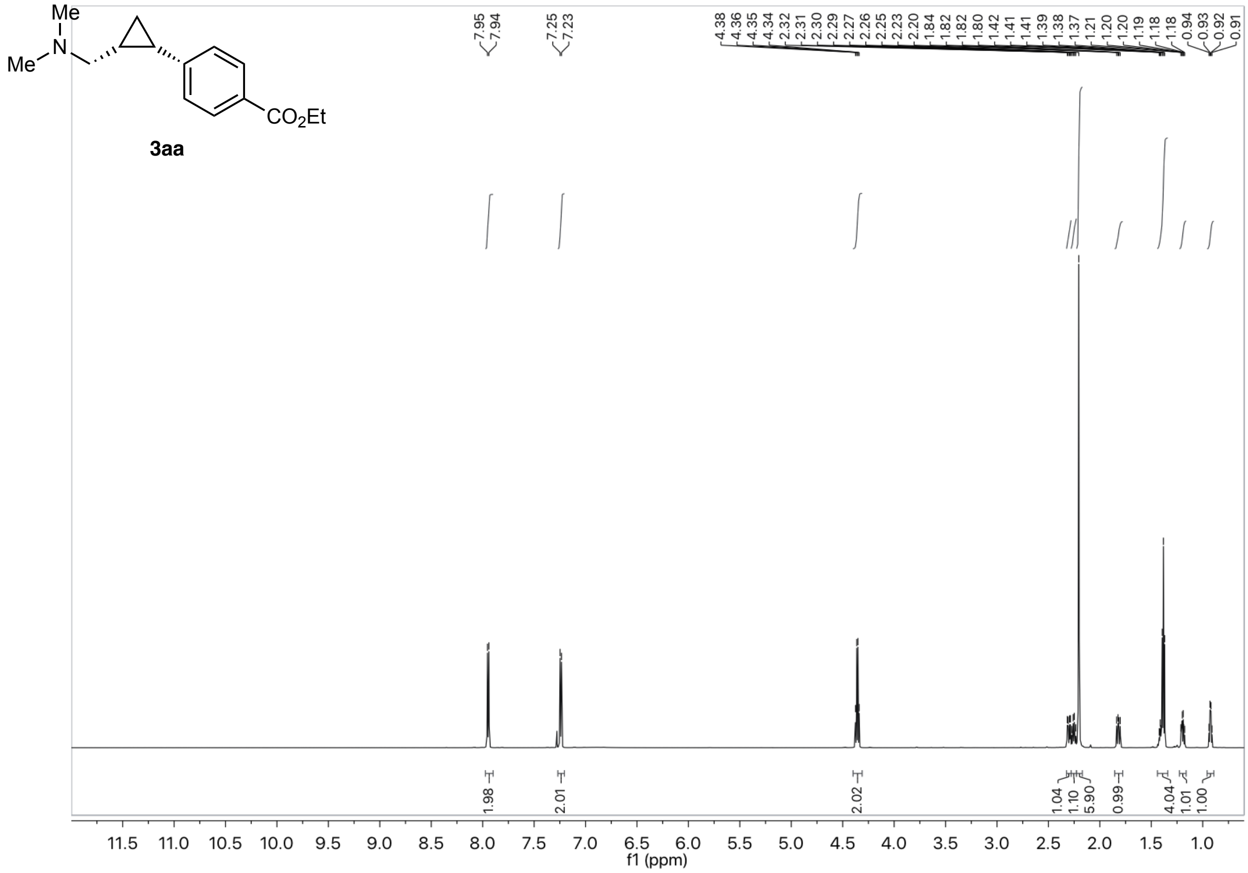
3aa
CO2Et
f1 (ppm)

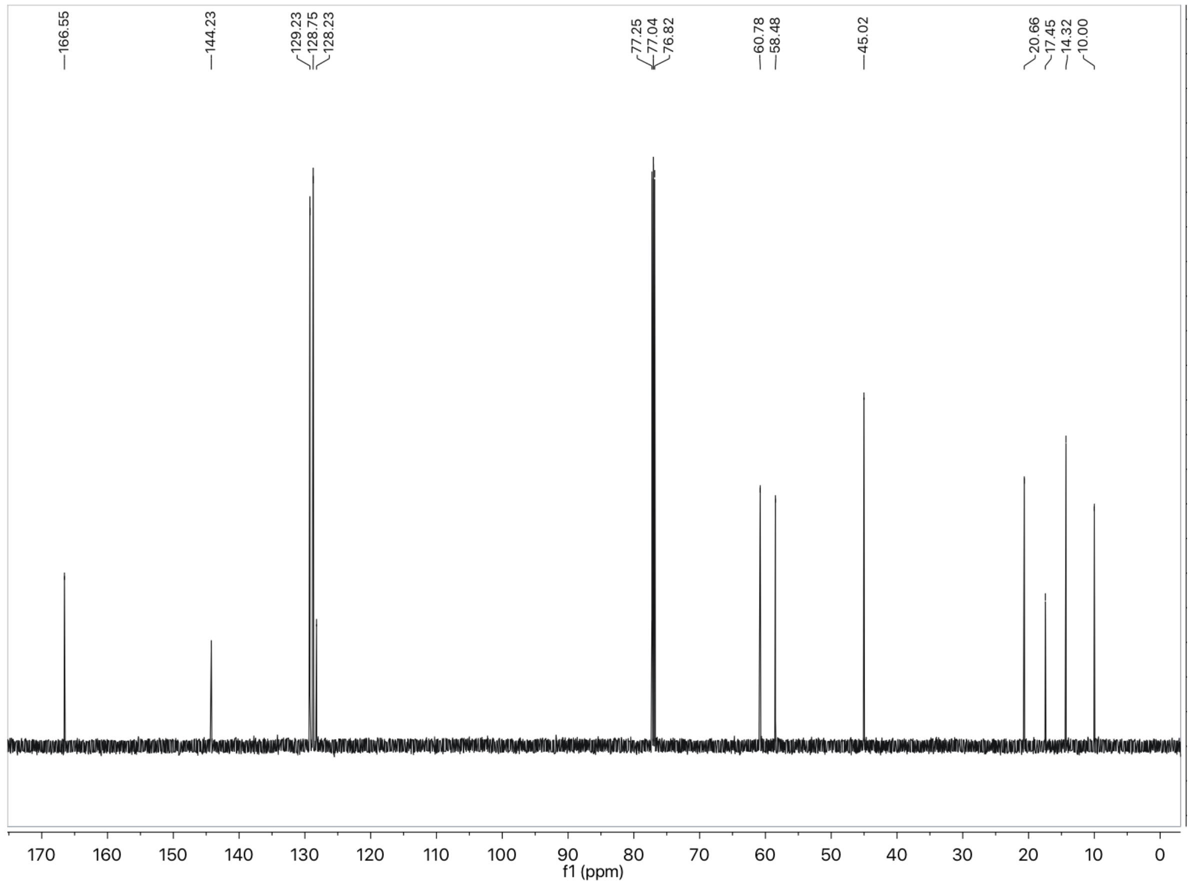
f1 (ppm)

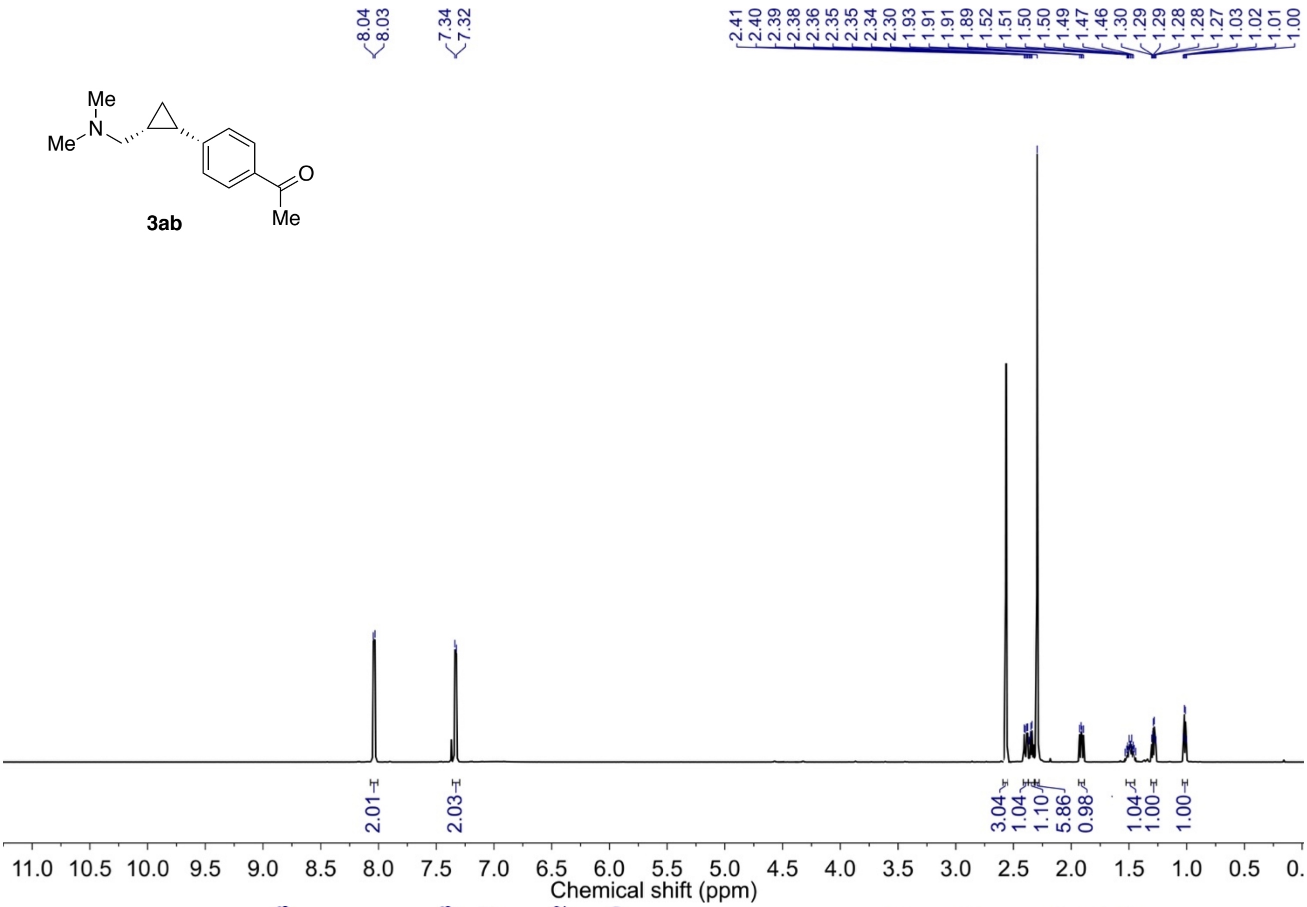
Me
N
Me
O
Me
3ab
8.04
8.03
7.34
7.32
2.41
2.40
2.39
2.38
2.36
2.35
2.35
2.34
2.30
1.93
1.91
1.91
1.89
1.52
1.51
1.50
1.50
1.49
1.47
1.46
1.30
1.29
1.29
1.28
1.28
1.27
1.03
1.02
1.01
1.00
2.01
2.03
3.04
1.04
1.10
5.86
0.98
1.04
1.00
1.00
11.0 10.5 10.0 9.5 9.0 8.5 8.0 7.5 7.0 6.5 6.0 5.5 5.0 4.5 4.0 3.5 3.0 2.5 2.0 1.5 1.0 0.5 0.
Chemical shift (ppm)

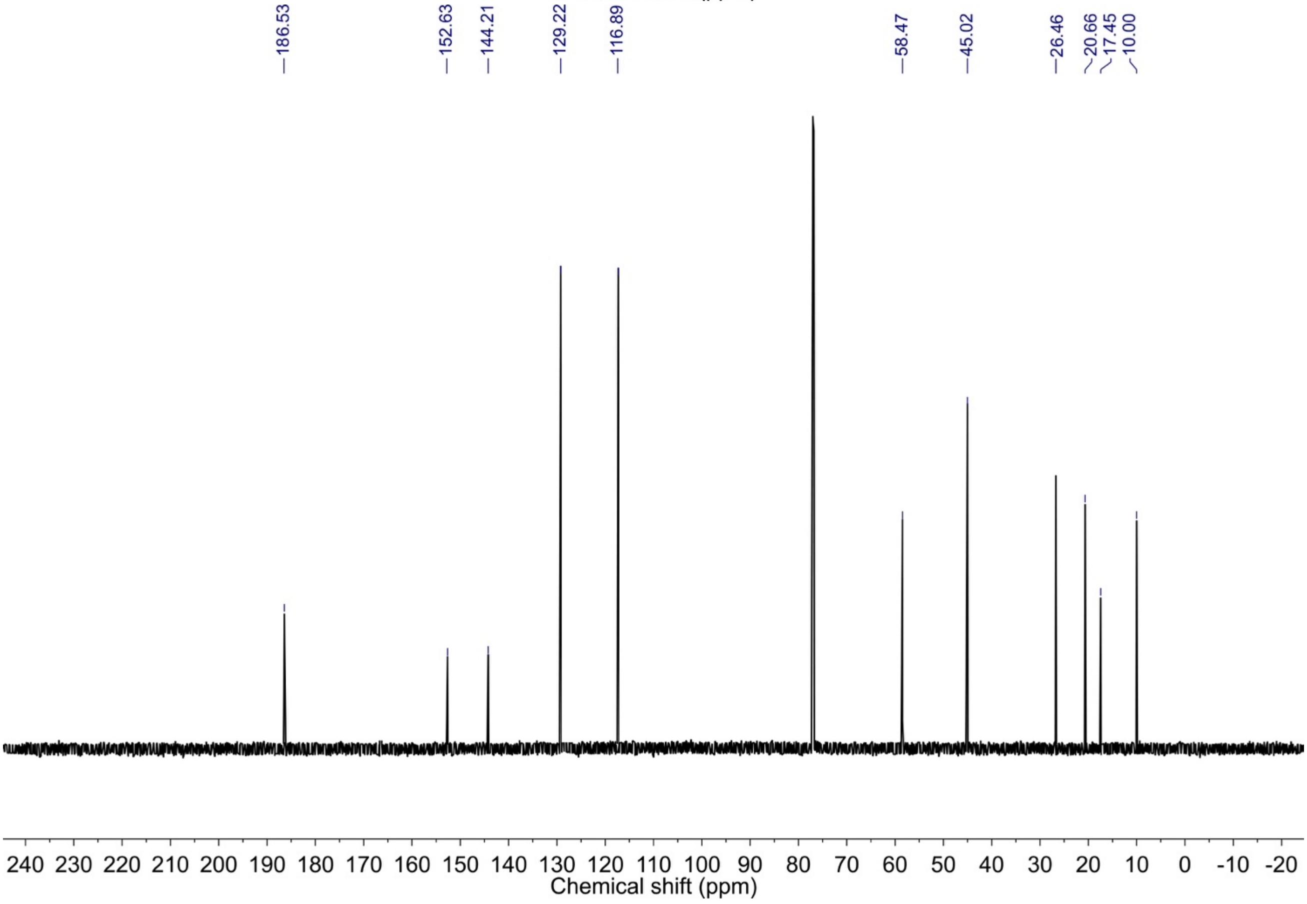
186.53
152.63
144.21
129.22
116.89
58.47
45.02
26.46
20.66
17.45
10.00
240 230 220 210 200 190 180 170 160 150 140 130 120 110 100 90 80 70 60 50 40 30 20 10 0 -10 -20
Chemical shift (ppm)

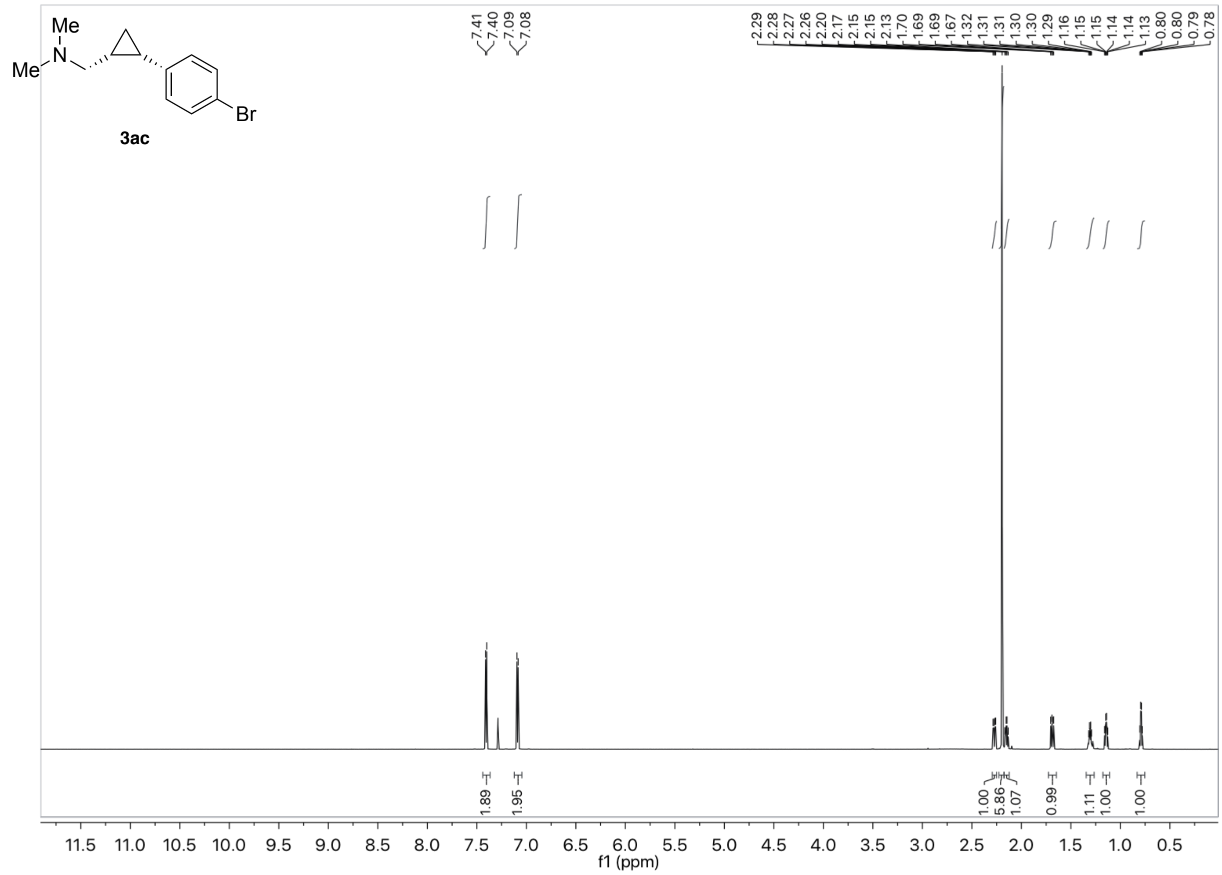
Me
N
Me
Br
3ac
7.41
7.40
7.09
7.08
2.29
2.28
2.27
2.26
2.20
2.17
2.15
2.15
2.13
1.70
1.69
1.69
1.67
1.32
1.31
1.31
1.30
1.30
1.29
1.16
1.15
1.15
1.14
1.14
1.13
0.80
0.80
0.79
0.78
1.89
1.95
1.00
5.86
1.07
0.99
1.11
1.00
1.00
11.5 11.0 10.5 10.0 9.5 9.0 8.5 8.0 7.5 7.0 6.5 6.0 5.5 5.0 4.5 4.0 3.5 3.0 2.5 2.0 1.5 1.0 0.5
f1 (ppm)

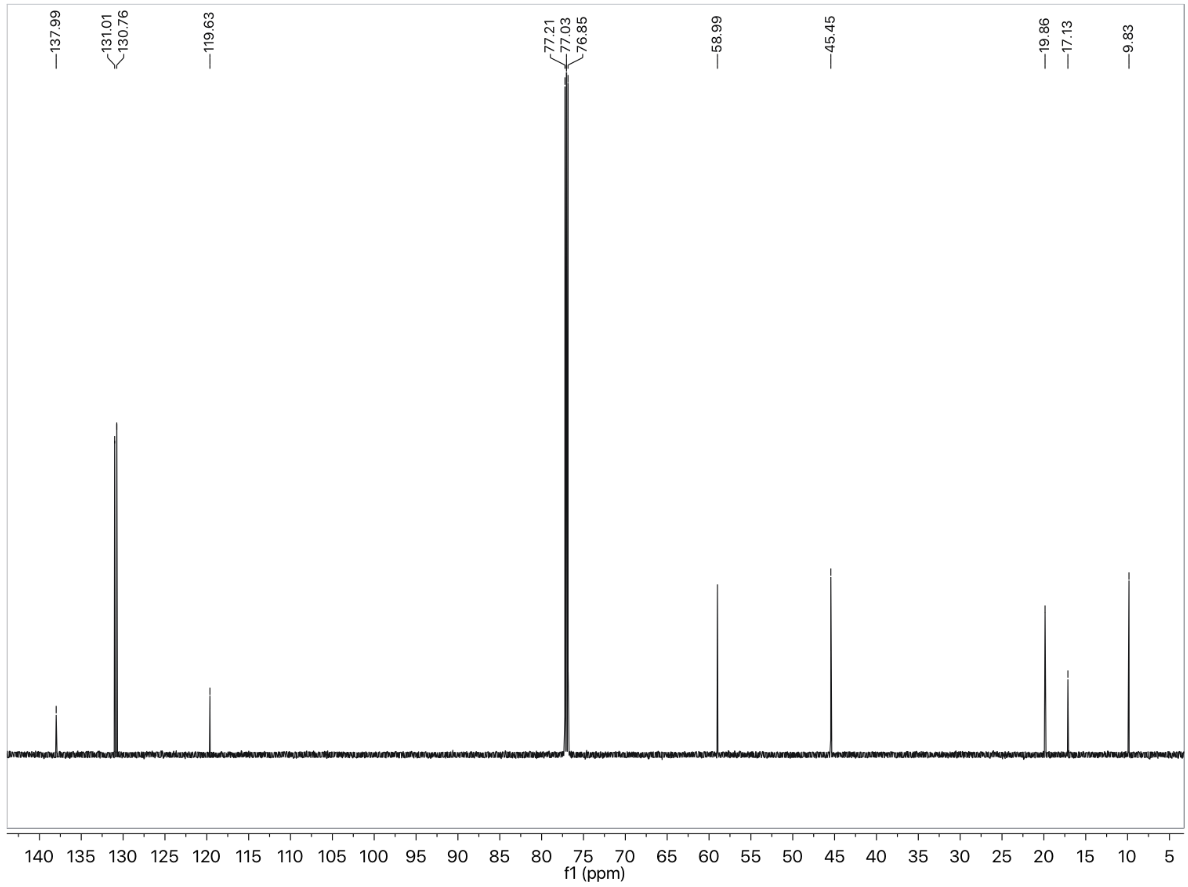
137.99
131.01
130.76
119.63
77.21
77.03
76.85
58.99
45.45
19.86
17.13
9.83
140 135 130 125 120 115 110 105 100 95 90 85 80 75 70 65 60 55 50 45 40 35 30 25 20 15 10 5
f1 (ppm)

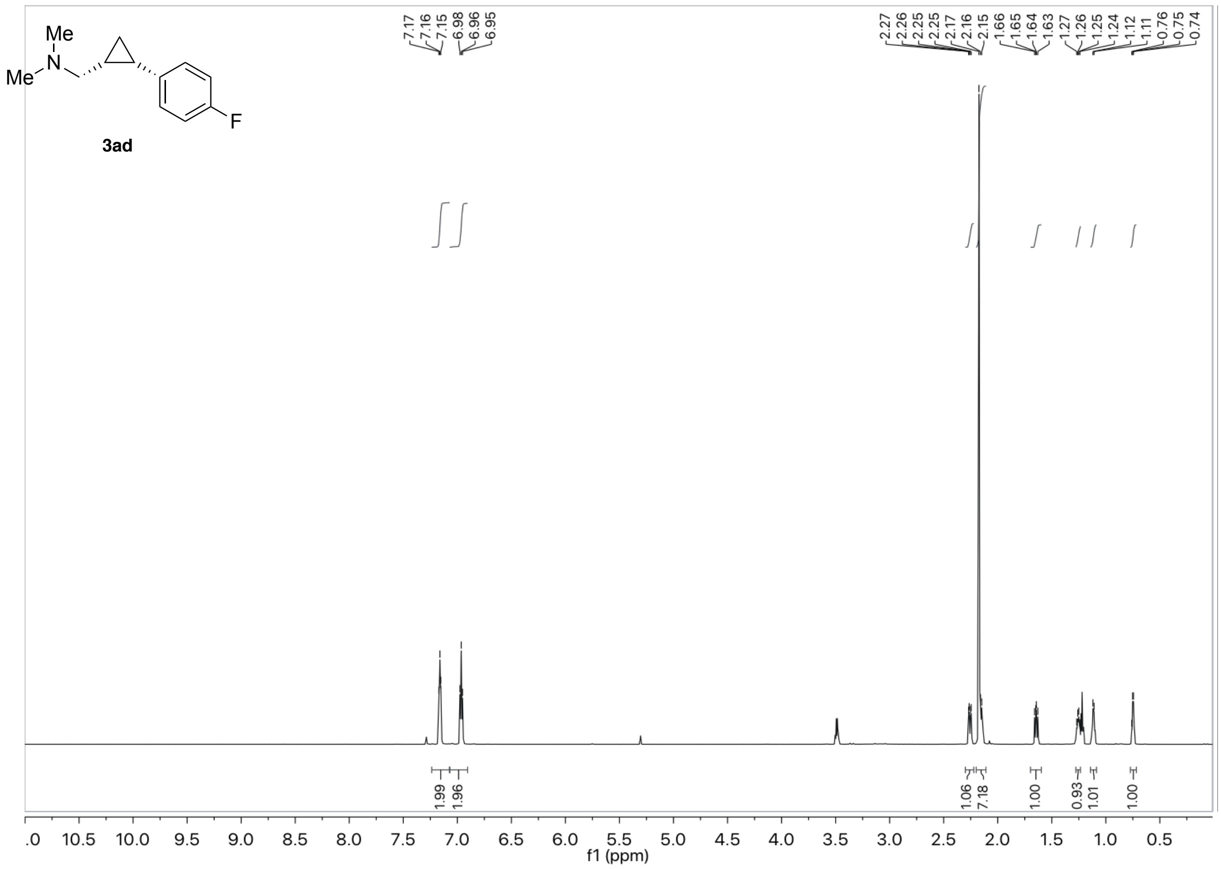

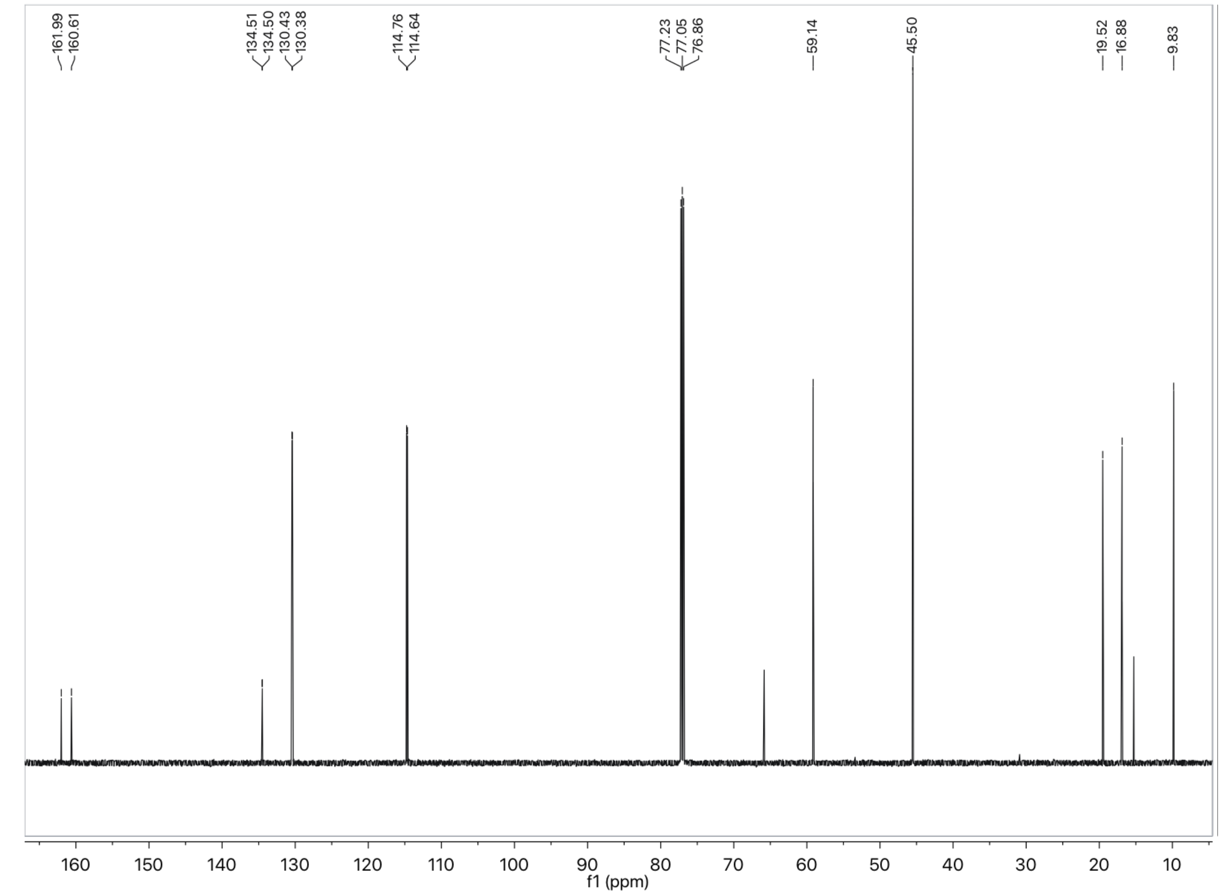

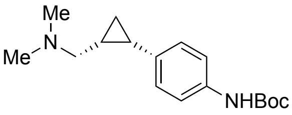

3ae

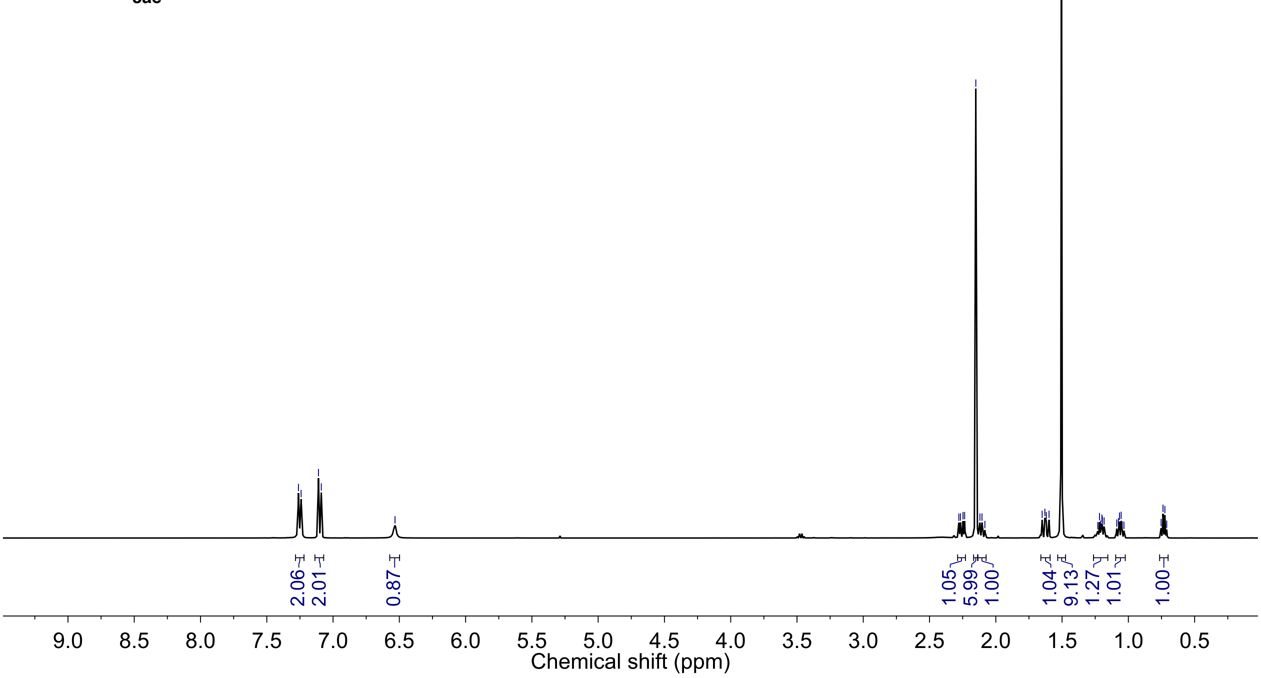
7.26
7.24
7.11
7.09
6.53
2.28
2.27
2.25
2.24
2.15
2.12
2.10
2.08
1.65
1.63
1.62
1.60
1.50
1.23
1.22
1.21
1.20
1.20
1.18
1.09
1.08
1.07
1.05
1.05
1.03
0.75
0.74
0.72
0.71
2.06
2.01
0.87
1.05
5.99
1.00
1.04
9.13
1.27
1.01
1.00
9.0 8.5 8.0 7.5 7.0 6.5 6.0 5.5 5.0 4.5 4.0 3.5 3.0 2.5 2.0 1.5 1.0 0.5
Chemical shift (ppm)

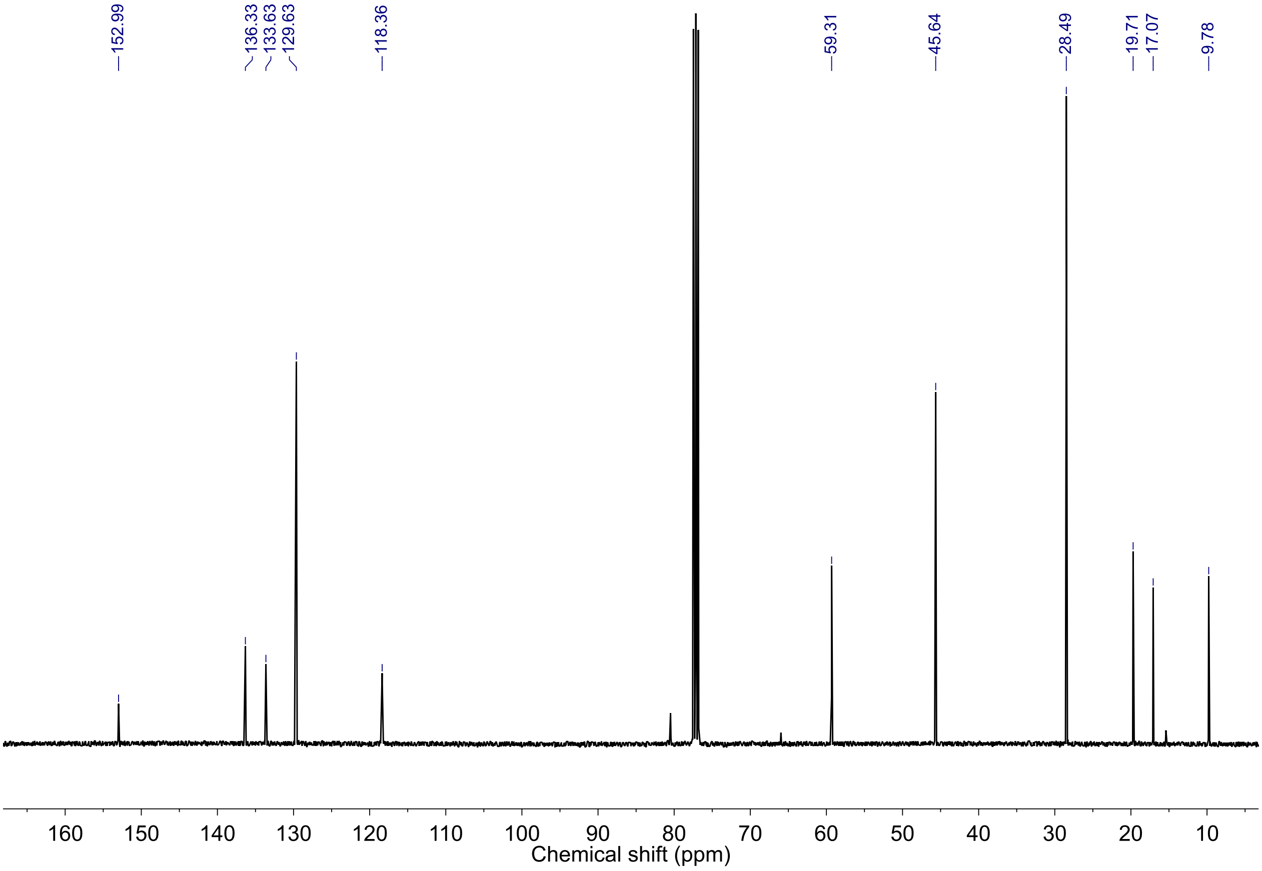
152.99
136.33
133.63
129.63
118.36
59.31
45.64
28.49
19.71
17.07
9.78
160 150 140 130 120 110 100 90 80 70 60 50 40 30 20 10
Chemical shift (ppm)

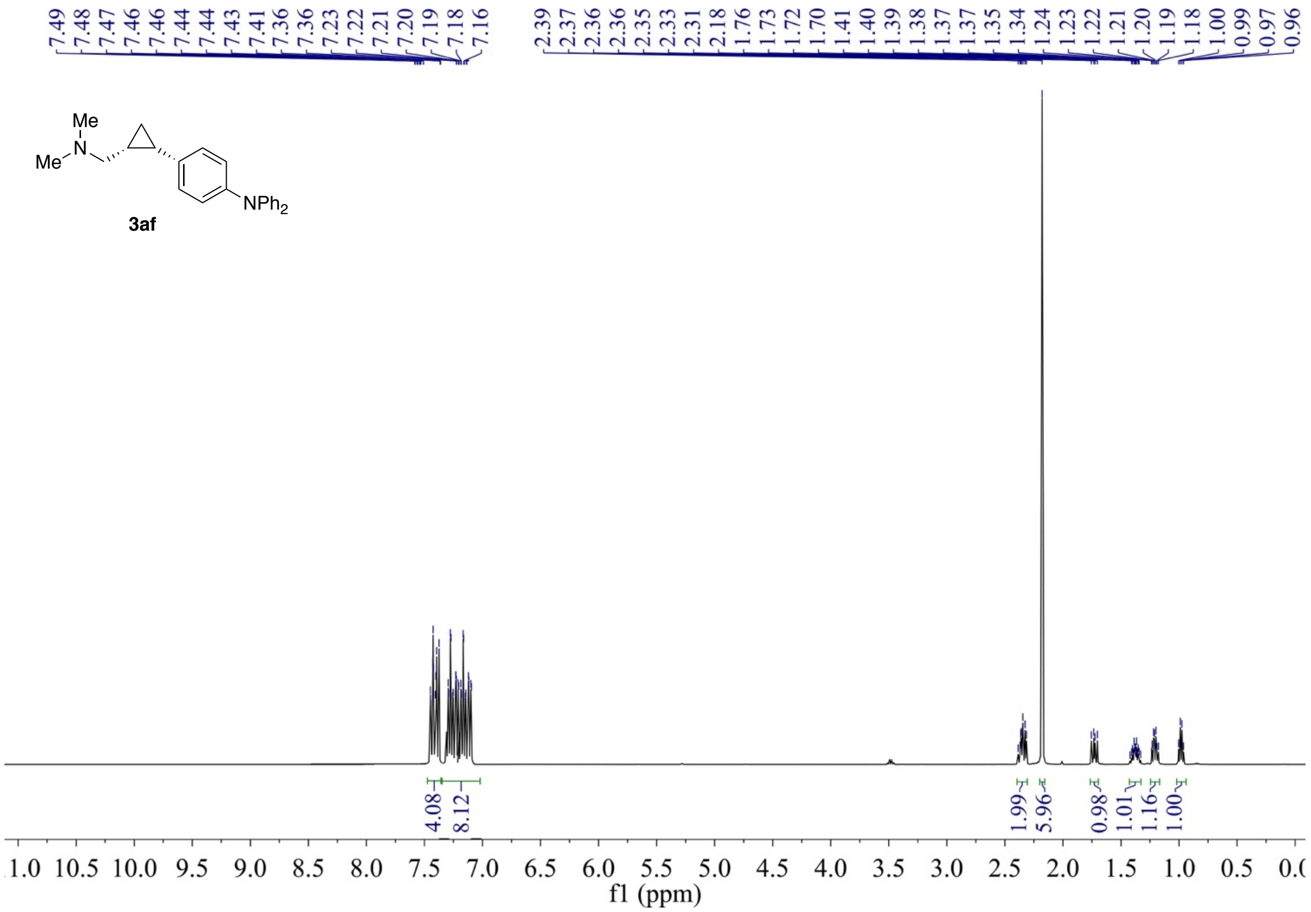
7.49 7.48 7.47 7.46 7.46 7.44 7.44 7.43 7.41 7.36 7.36 7.23 7.22 7.21 7.20 7.19 7.18 7.16
2.39 2.37 2.36 2.36 2.35 2.33 2.31 2.18 1.76 1.73 1.72 1.70 1.41 1.40 1.39 1.38 1.37 1.37 1.35 1.34 1.24 1.23 1.22 1.21 1.20 1.19 1.18 1.00 0.99 0.97 0.96
Me
Me
N
NPh2
3af
4.08
8.12
1.99
5.96
0.98
1.01
1.16
1.00
f1 (ppm)
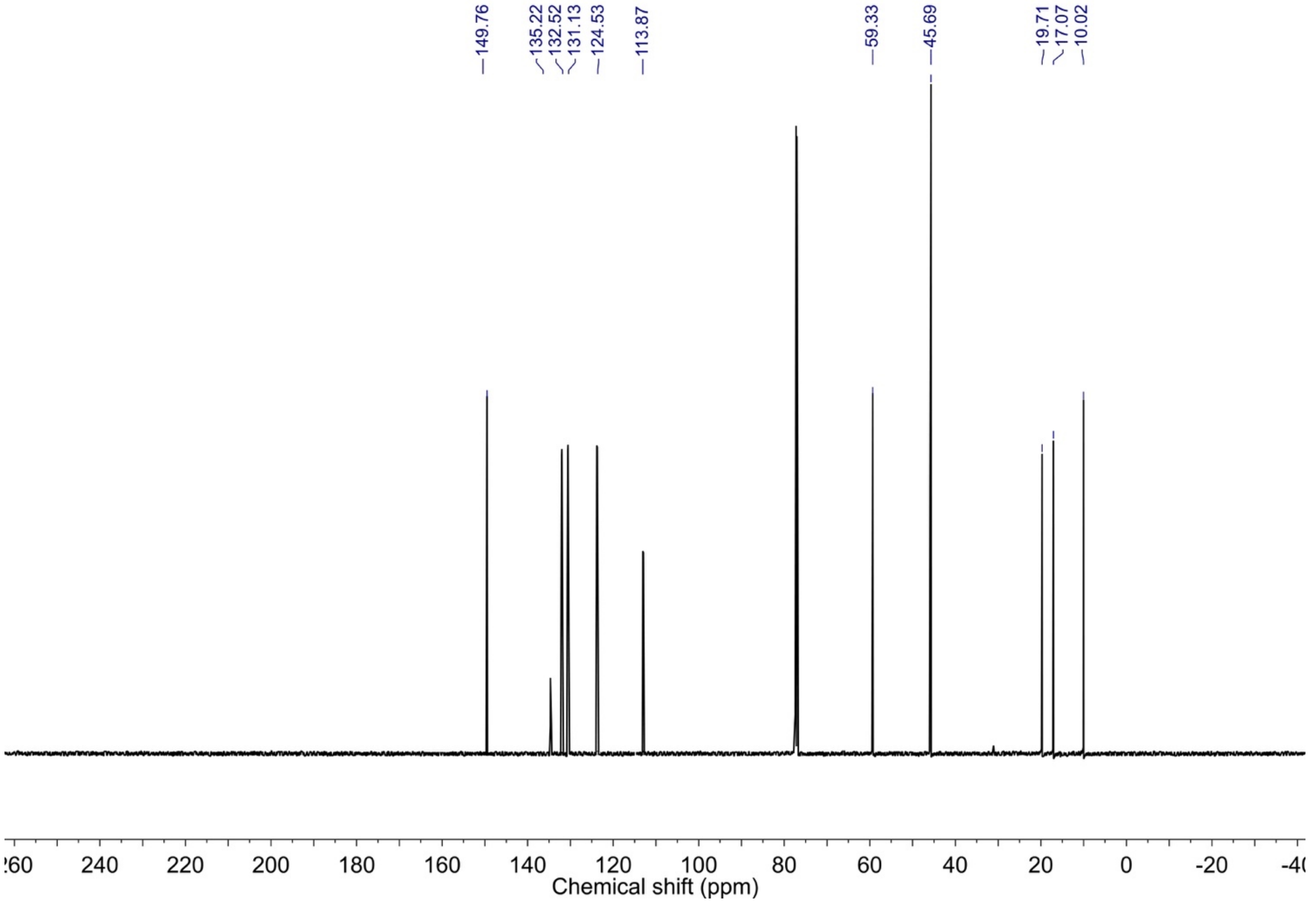
149.76
135.22
132.52
131.13
124.53
113.87
59.33
45.69
19.71
17.07
10.02
Chemical shift (ppm)

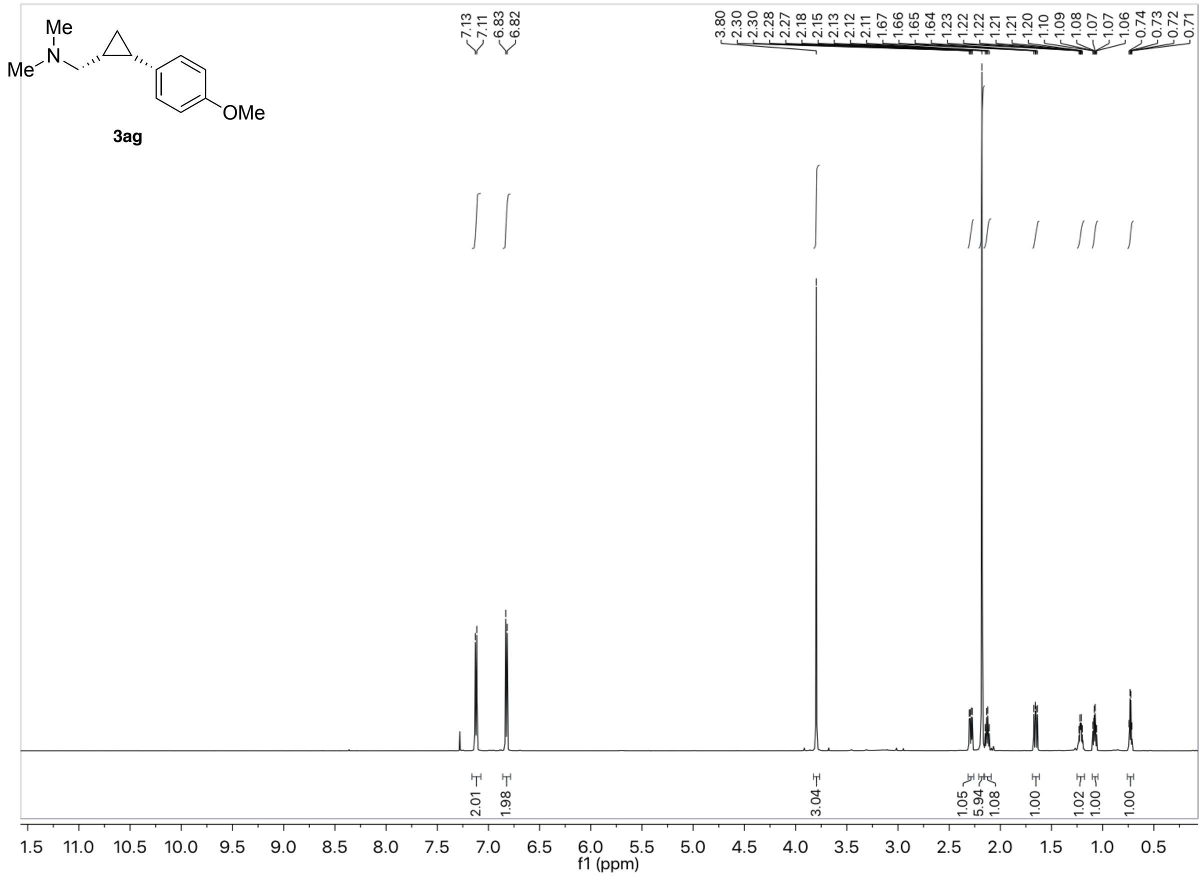
Me
N
Me
OMe
3ag
7.13
7.11
6.83
6.82
3.80
2.30
2.30
2.28
2.27
2.18
2.15
2.13
2.12
2.11
1.67
1.66
1.65
1.64
1.23
1.22
1.22
1.21
1.21
1.20
1.10
1.09
1.08
1.07
1.07
1.06
0.74
0.73
0.72
0.71
2.01
1.98
3.04
1.05
5.94
1.08
1.00
1.02
1.00
1.00
1.5 11.0 10.5 10.0 9.5 9.0 8.5 8.0 7.5 7.0 6.5 6.0 5.5 5.0 4.5 4.0 3.5 3.0 2.5 2.0 1.5 1.0 0.5
f1 (ppm)

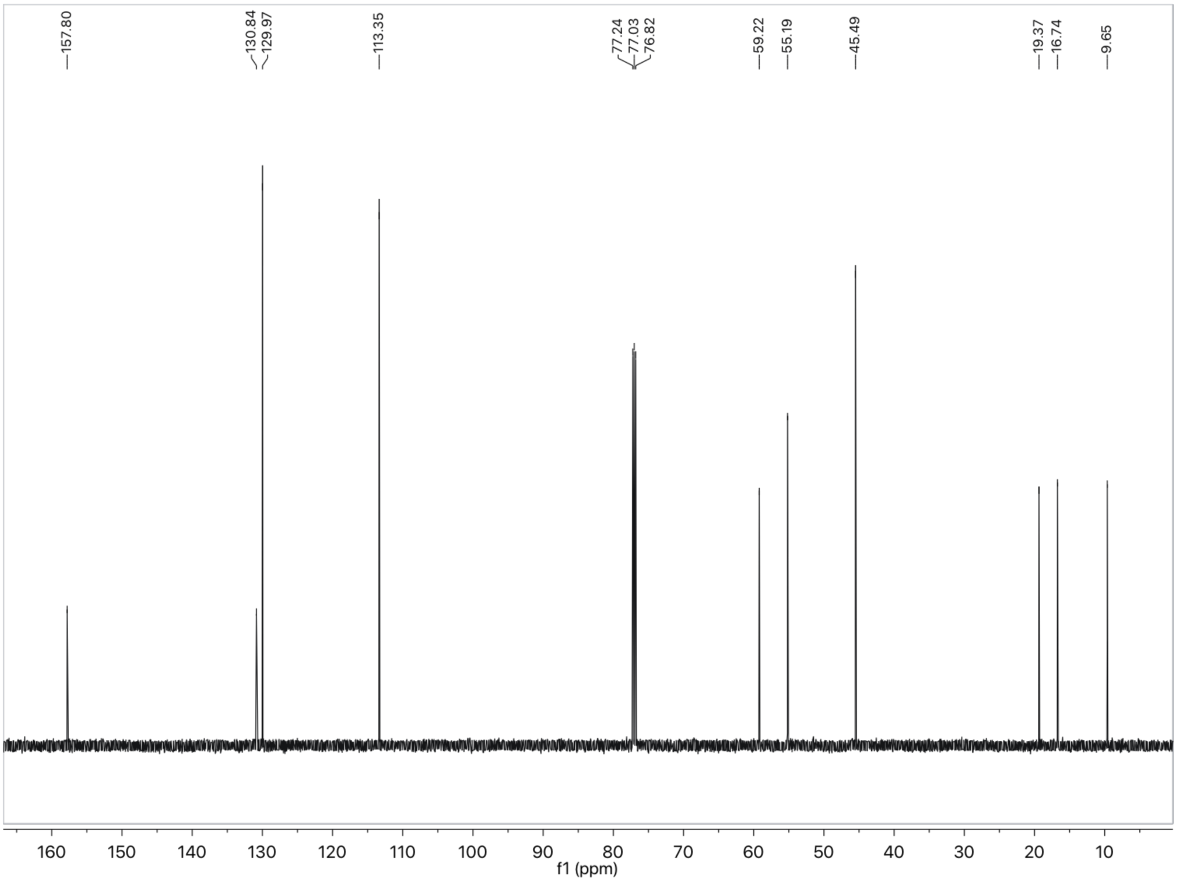
157.80
130.84
129.97
113.35
77.24
77.03
76.82
59.22
55.19
45.49
19.37
16.74
9.65
160 150 140 130 120 110 100 90 80 70 60 50 40 30 20 10
f1 (ppm)

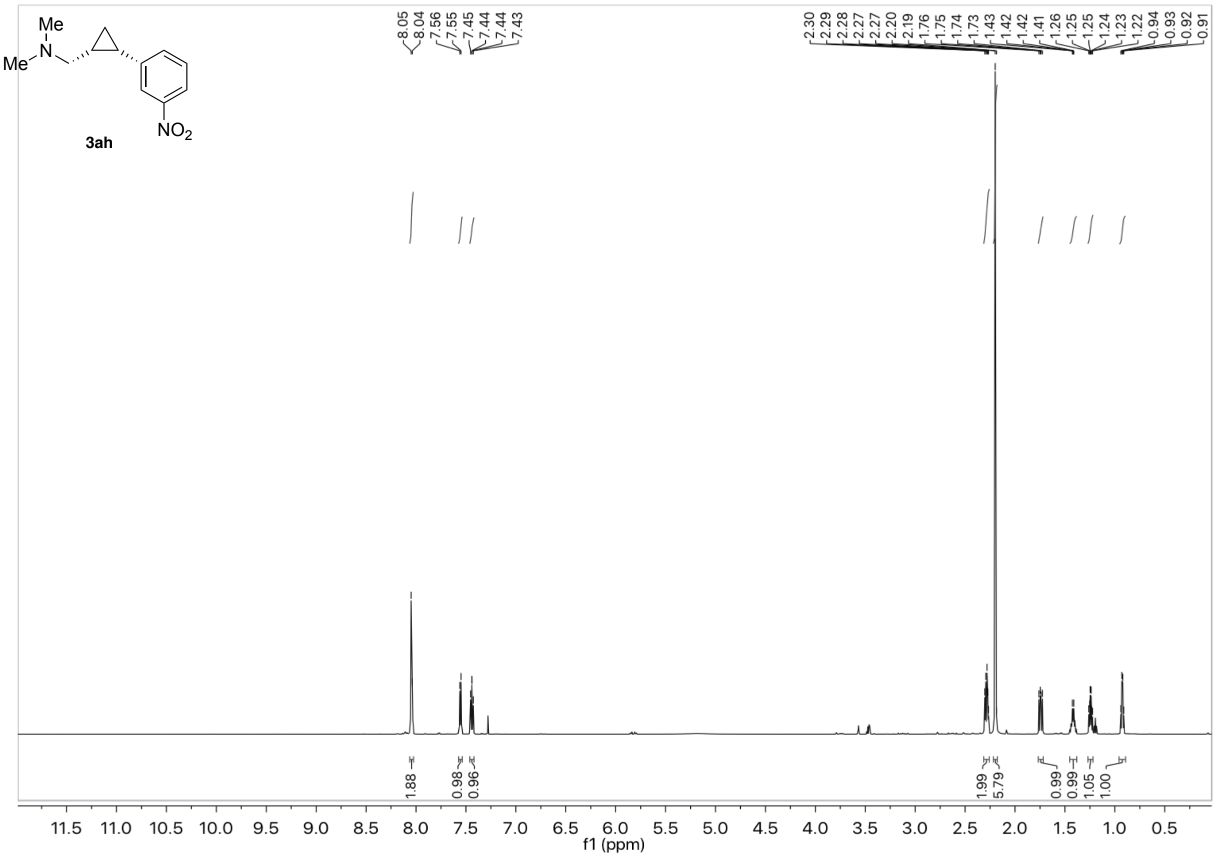
Me
N
Me
NO2
3ah
8.05
8.04
7.56
7.55
7.45
7.44
7.44
7.43
2.30
2.29
2.28
2.27
2.27
2.20
2.19
1.76
1.75
1.74
1.73
1.43
1.42
1.42
1.41
1.26
1.25
1.25
1.24
1.23
1.22
0.94
0.93
0.92
0.91
1.88
0.98
0.96
1.99
5.79
0.99
0.99
1.05
1.00
11.5 11.0 10.5 10.0 9.5 9.0 8.5 8.0 7.5 7.0 6.5 6.0 5.5 5.0 4.5 4.0 3.5 3.0 2.5 2.0 1.5 1.0 0.5
f1 (ppm)

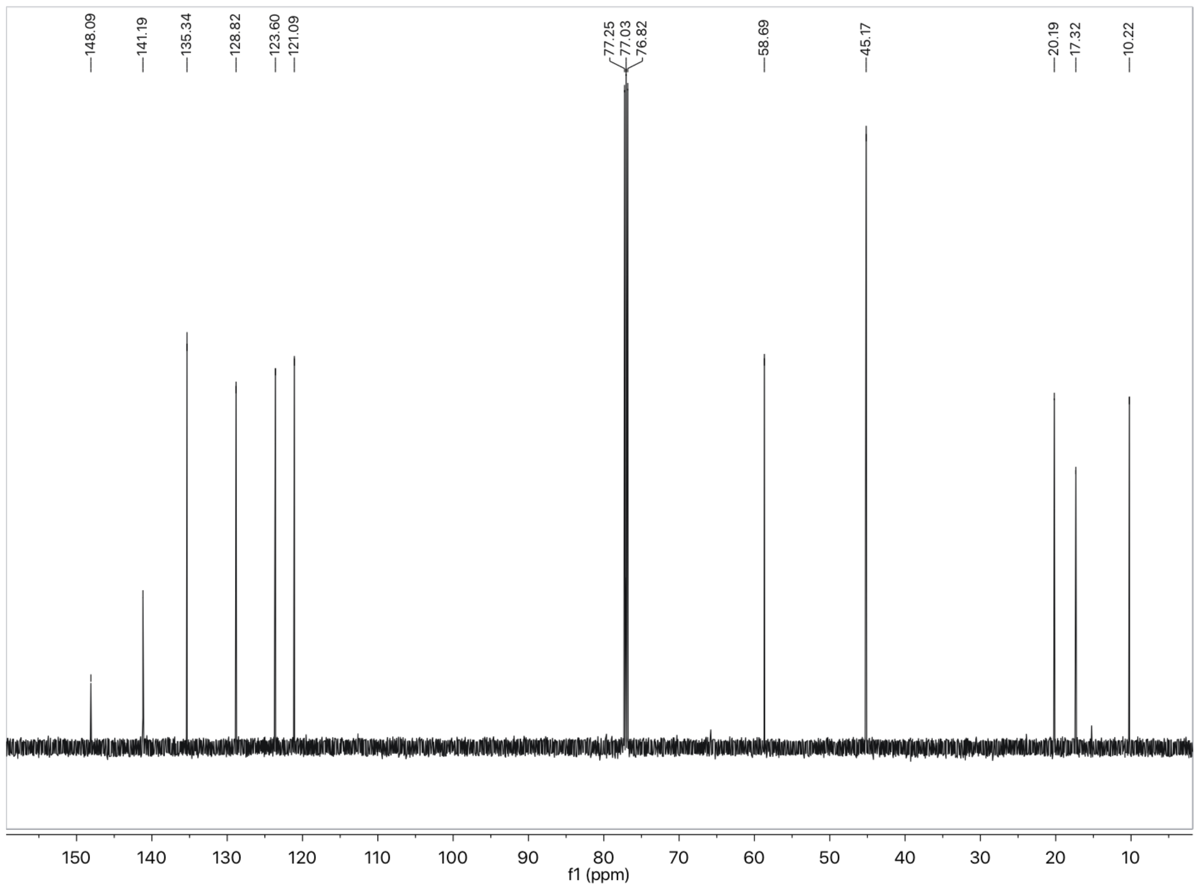
148.09
141.19
135.34
128.82
123.60
121.09
77.25
77.03
76.82
58.69
45.17
20.19
17.32
10.22
150 140 130 120 110 100 90 80 70 60 50 40 30 20 10
f1 (ppm)

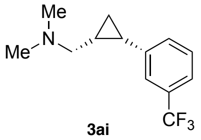
Me
Me
N
CF3

3ai

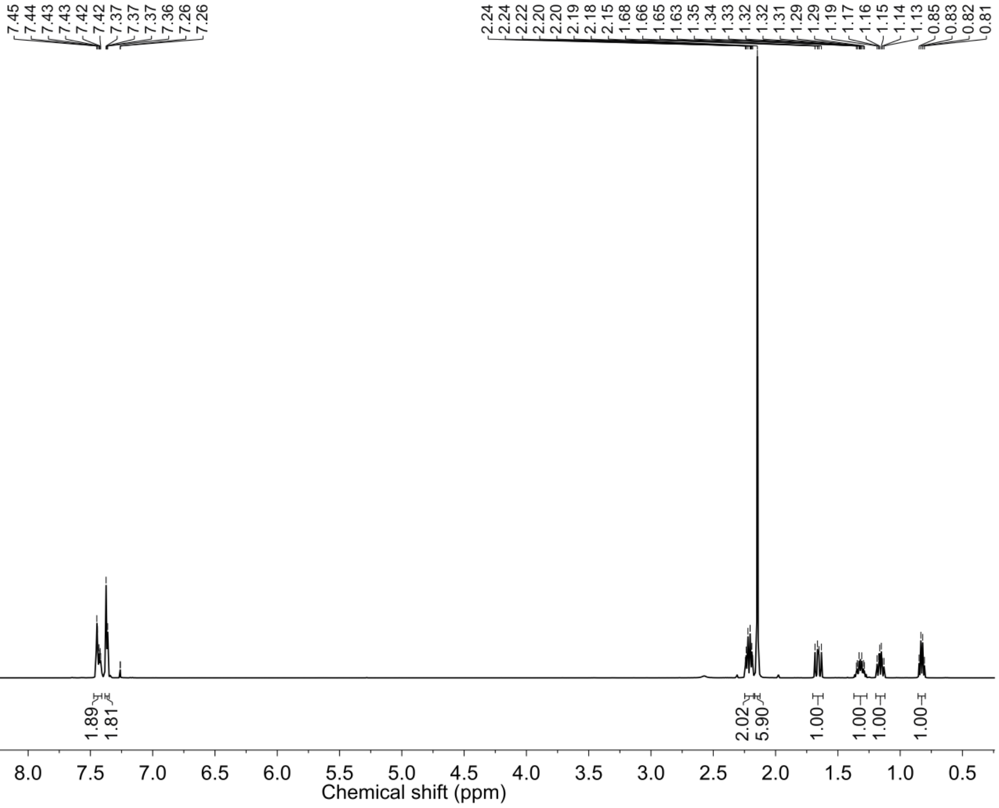
7.45
7.44
7.43
7.43
7.42
7.42
7.37
7.37
7.37
7.36
7.26
7.26
2.24
2.24
2.22
2.20
2.20
2.19
2.18
2.15
1.68
1.66
1.65
1.63
1.35
1.34
1.33
1.32
1.32
1.31
1.29
1.29
1.19
1.17
1.16
1.15
1.14
1.13
0.85
0.83
0.82
0.81
1.89
1.81
2.02
5.90
1.00
1.00
1.00
1.00
9.5
9.0
8.5
8.0
7.5
7.0
6.5
6.0
5.5
5.0
4.5
4.0
3.5
3.0
2.5
2.0
1.5
1.0
0.5
Chemical shift (ppm)

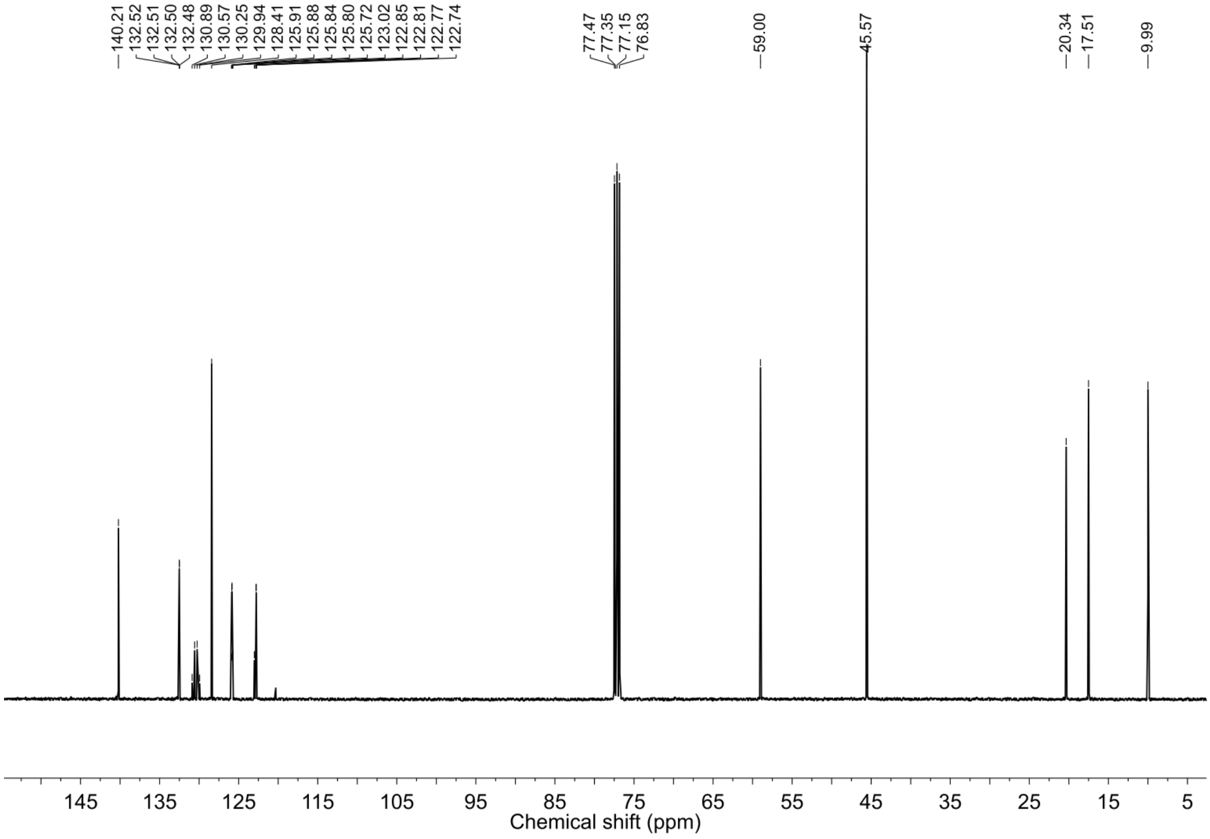
140.21
132.52
132.51
132.50
132.48
130.89
130.57
130.25
129.94
128.41
125.91
125.88
125.84
125.80
125.72
123.02
122.85
122.81
122.77
122.74
77.47
77.35
77.15
76.83
59.00
45.57
20.34
17.51
9.99
145
135
125
115
105
95
85
75
65
55
45
35
25
15
5
Chemical shift (ppm)

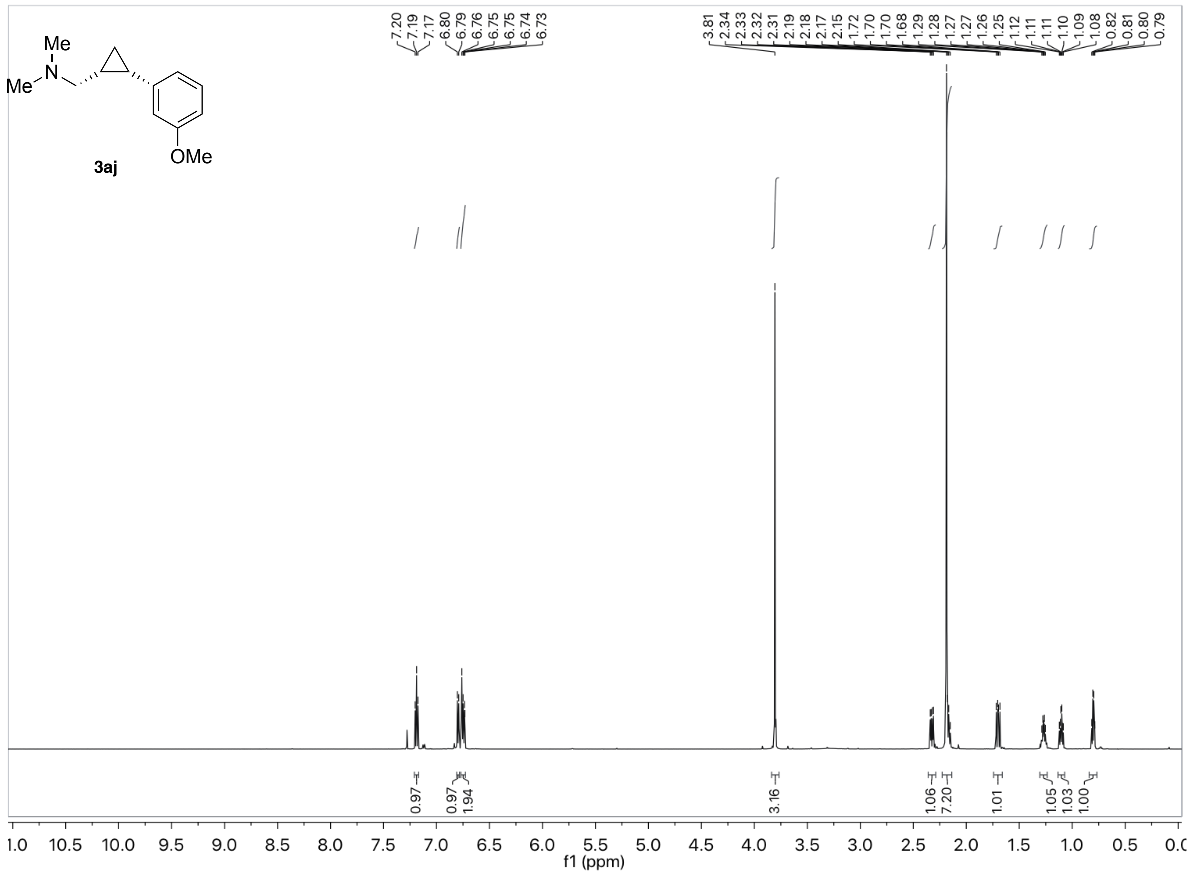
Me
N
Me
OMe
3aj
7.20
7.19
7.17
6.80
6.79
6.76
6.75
6.75
6.74
6.73
3.81
2.34
2.33
2.32
2.31
2.19
2.18
2.17
2.15
1.72
1.70
1.70
1.68
1.29
1.28
1.27
1.27
1.26
1.25
1.12
1.11
1.11
1.10
1.09
1.08
0.82
0.81
0.80
0.79
0.97
0.97
1.94
3.16
1.06
7.20
1.01
1.05
1.03
1.00
f1 (ppm)

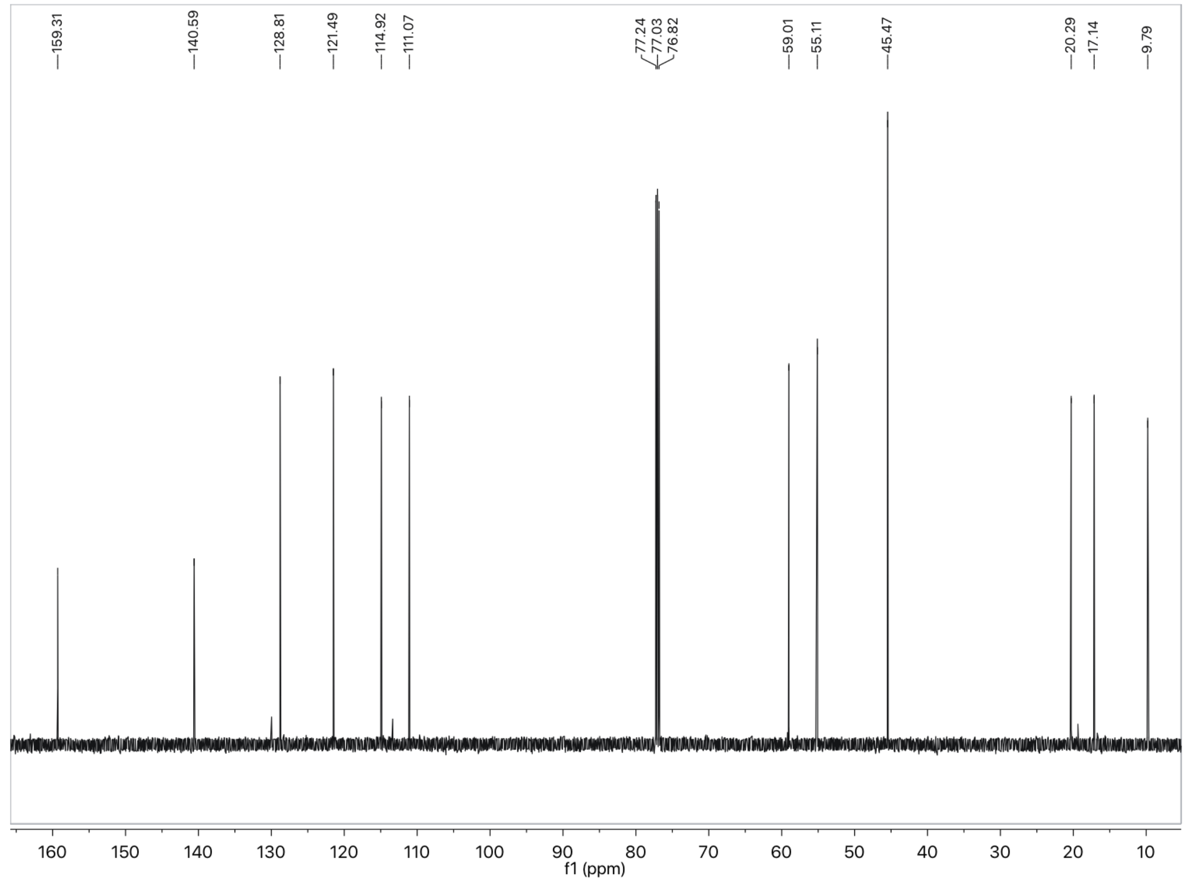
159.31
140.59
128.81
121.49
114.92
111.07
77.24
77.03
76.82
59.01
55.11
45.47
20.29
17.14
9.79
f1 (ppm)

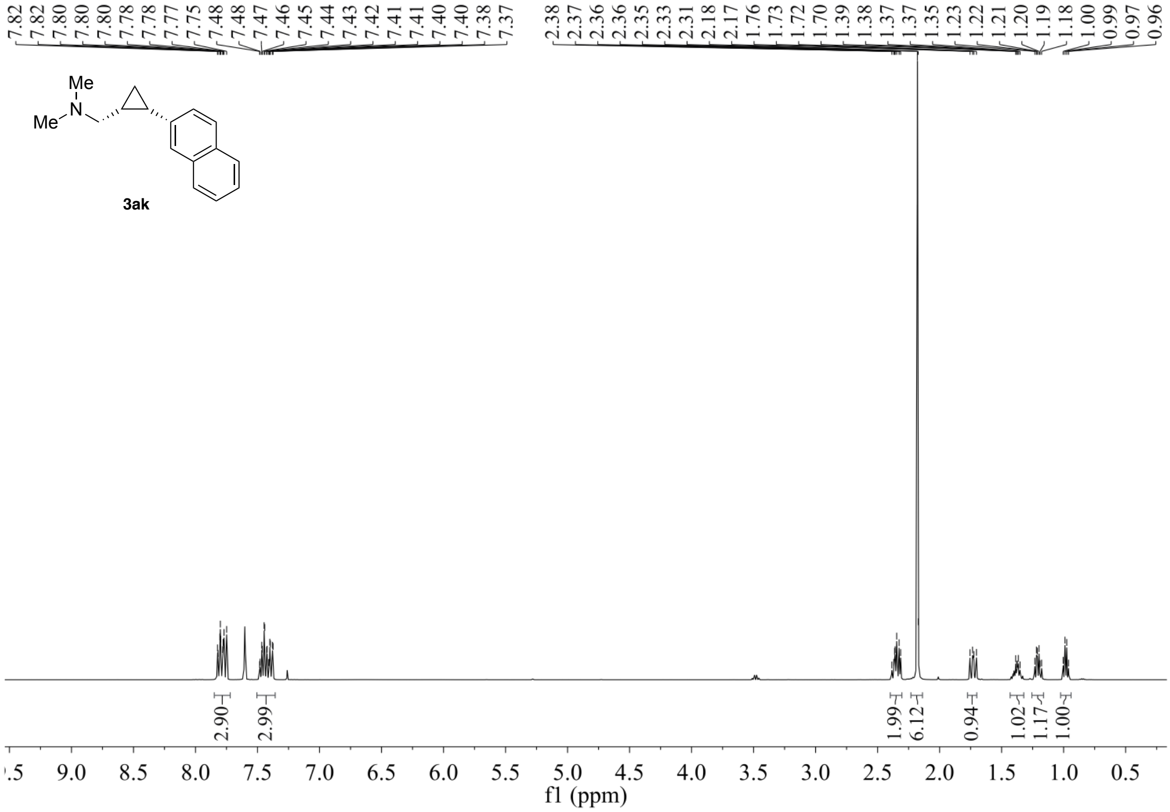
7.82 7.82 7.80 7.80 7.80 7.78 7.78 7.77 7.75 7.48 7.48 7.47 7.46 7.45 7.44 7.43 7.42 7.41 7.41 7.40 7.40 7.38 7.37
2.38 2.37 2.36 2.36 2.35 2.33 2.31 2.18 2.17 1.76 1.73 1.72 1.70 1.39 1.38 1.37 1.37 1.35 1.23 1.22 1.21 1.20 1.19 1.18 1.00 0.99 0.97 0.96
Me
N
Me
3ak
2.90
2.99
1.99
6.12
0.94
1.02
1.17
1.00
9.0 8.5 8.0 7.5 7.0 6.5 6.0 5.5 5.0 4.5 4.0 3.5 3.0 2.5 2.0 1.5 1.0 0.5
f1 (ppm)

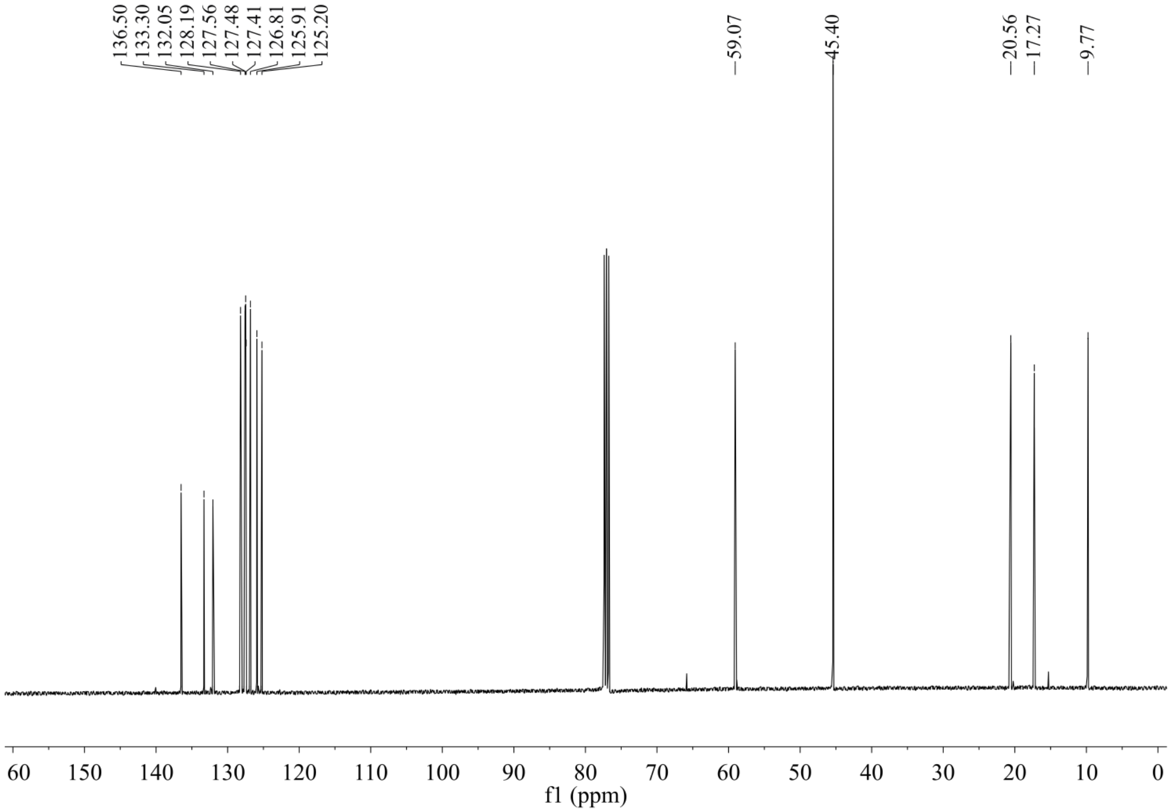
136.50 133.30 132.05 128.19 127.56 127.48 127.41 126.81 125.91 125.20
59.07
45.40
20.56
17.27
9.77
150 140 130 120 110 100 90 80 70 60 50 40 30 20 10 0
f1 (ppm)

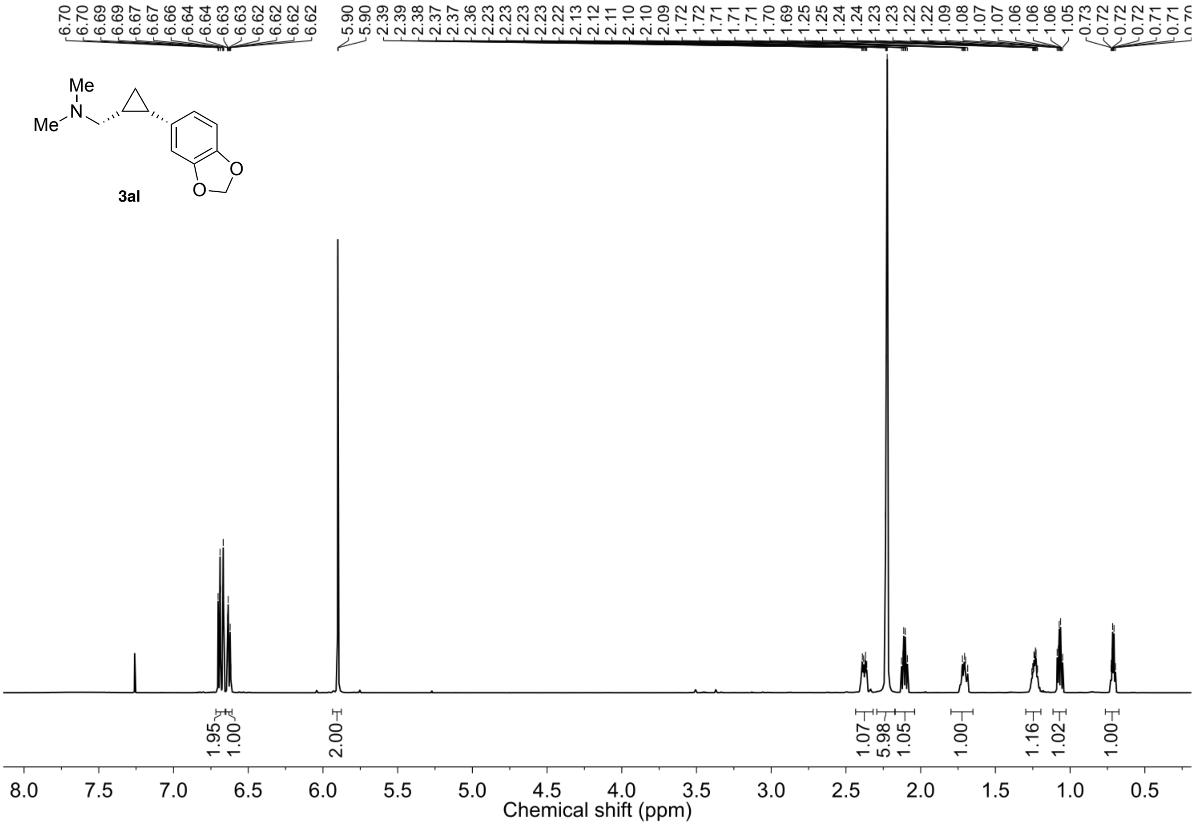
Me
N
Me
O
O
3al
Chemical shift (ppm)

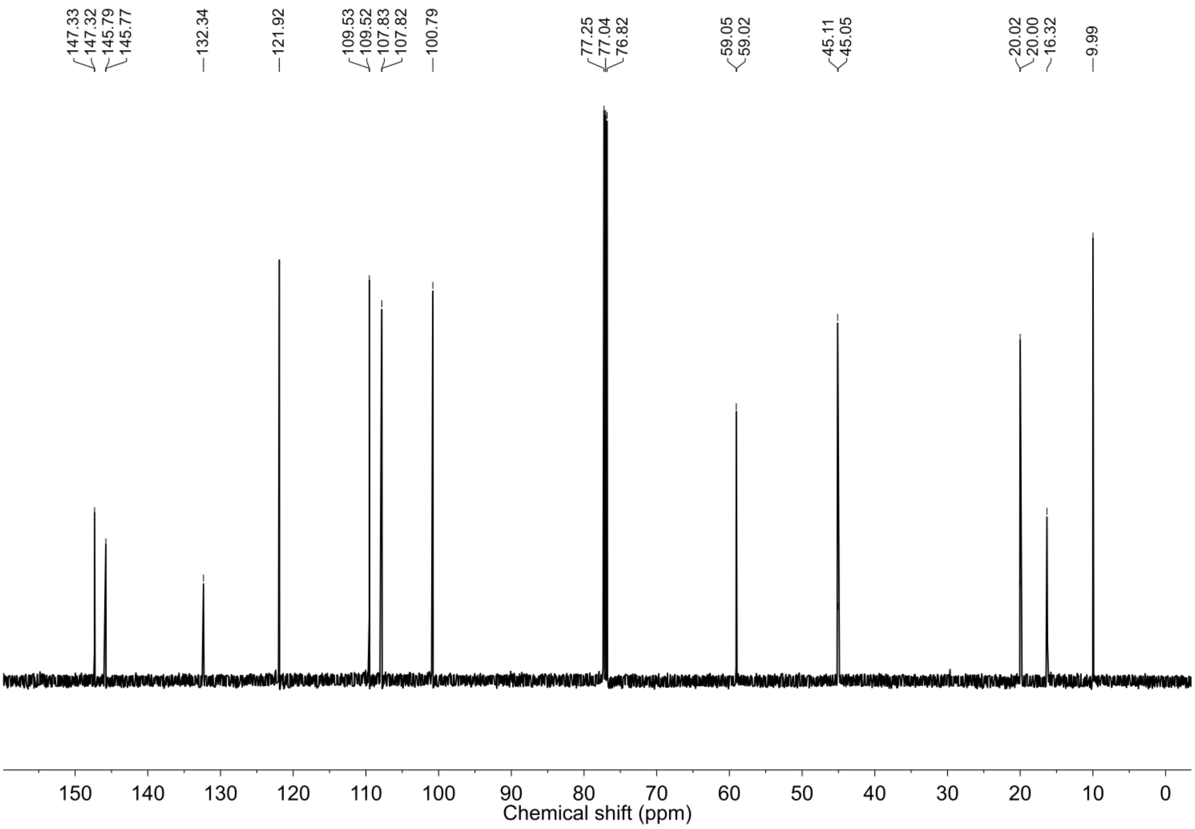
147.33
147.32
145.79
145.77
132.34
121.92
109.53
109.52
107.83
107.82
100.79
77.25
77.04
76.82
59.05
59.02
45.11
45.05
20.02
20.00
16.32
9.99
Chemical shift (ppm)

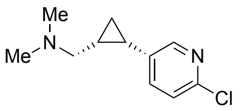
Me
Me
N
N
Cl

3am

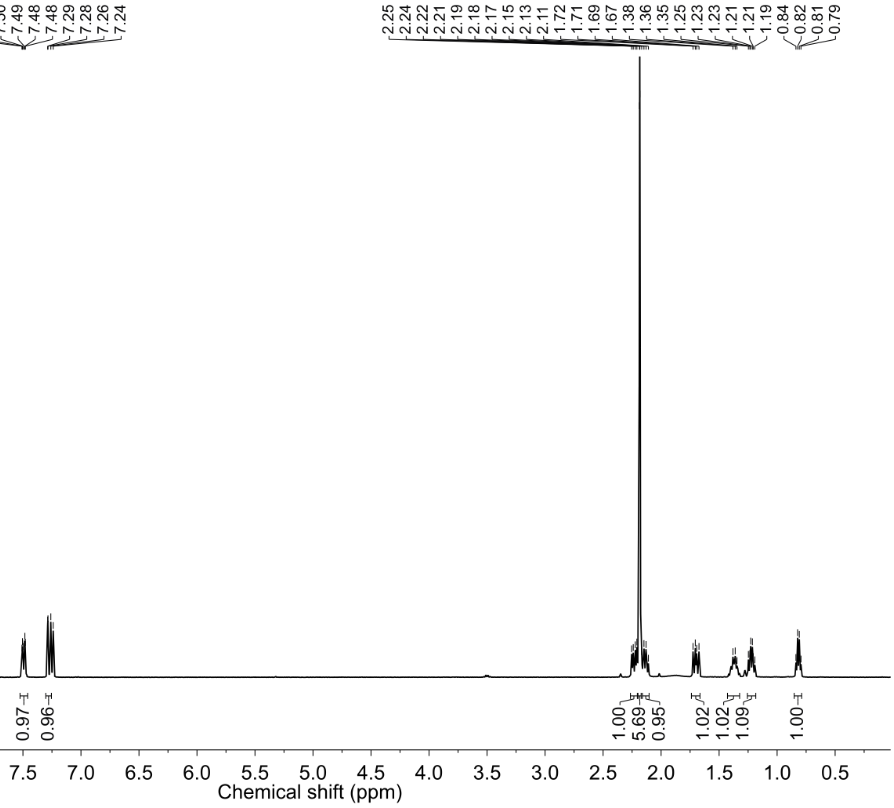
8.29 8.28 8.28 7.51 7.50 7.50 7.49 7.48 7.48 7.29 7.28 7.26 7.24
2.25 2.24 2.22 2.21 2.19 2.18 2.17 2.15 2.13 2.11 1.72 1.71 1.69 1.67 1.38 1.36 1.35 1.25 1.23 1.23 1.21 1.21 1.19 0.84 0.82 0.81 0.79
0.93 0.97 0.96 1.00 5.69 0.95 1.02 1.02 1.09 1.00
Chemical shift (ppm)

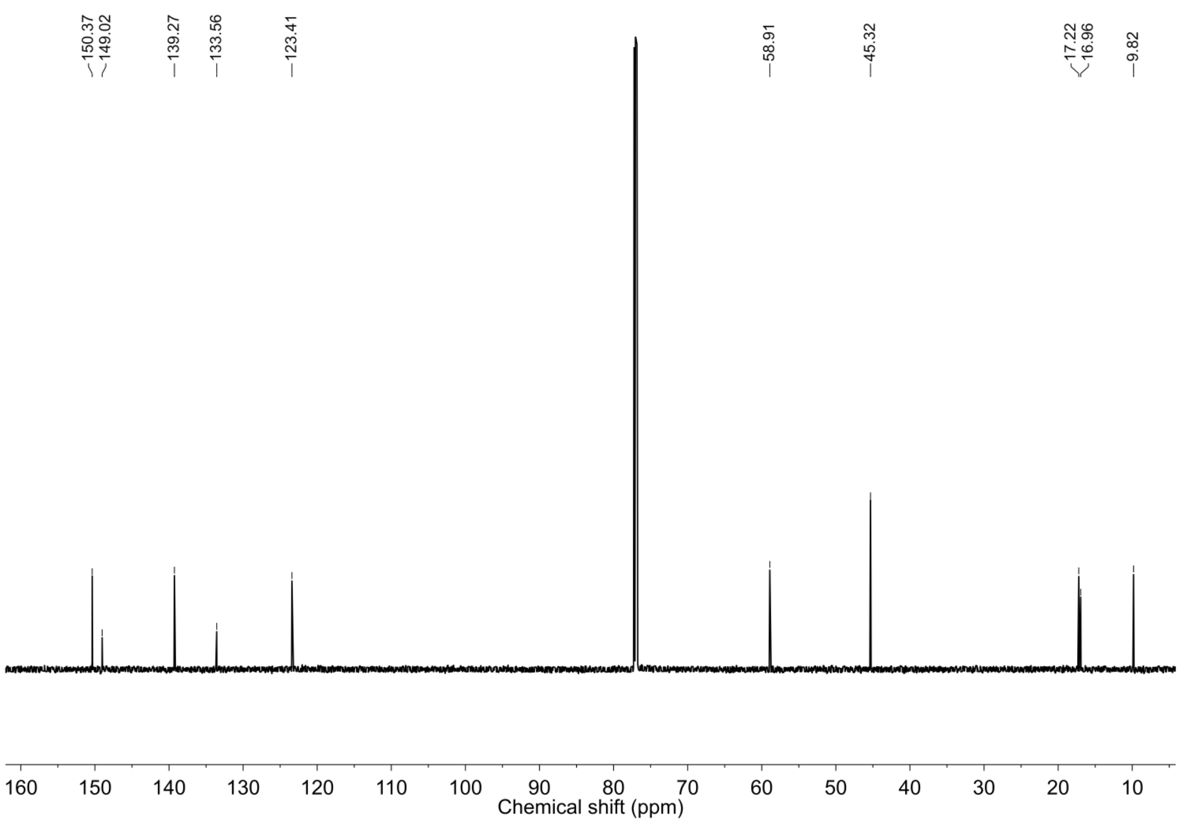
150.37 149.02 139.27 133.56 123.41 58.91 45.32 17.22 16.96 9.82
Chemical shift (ppm)

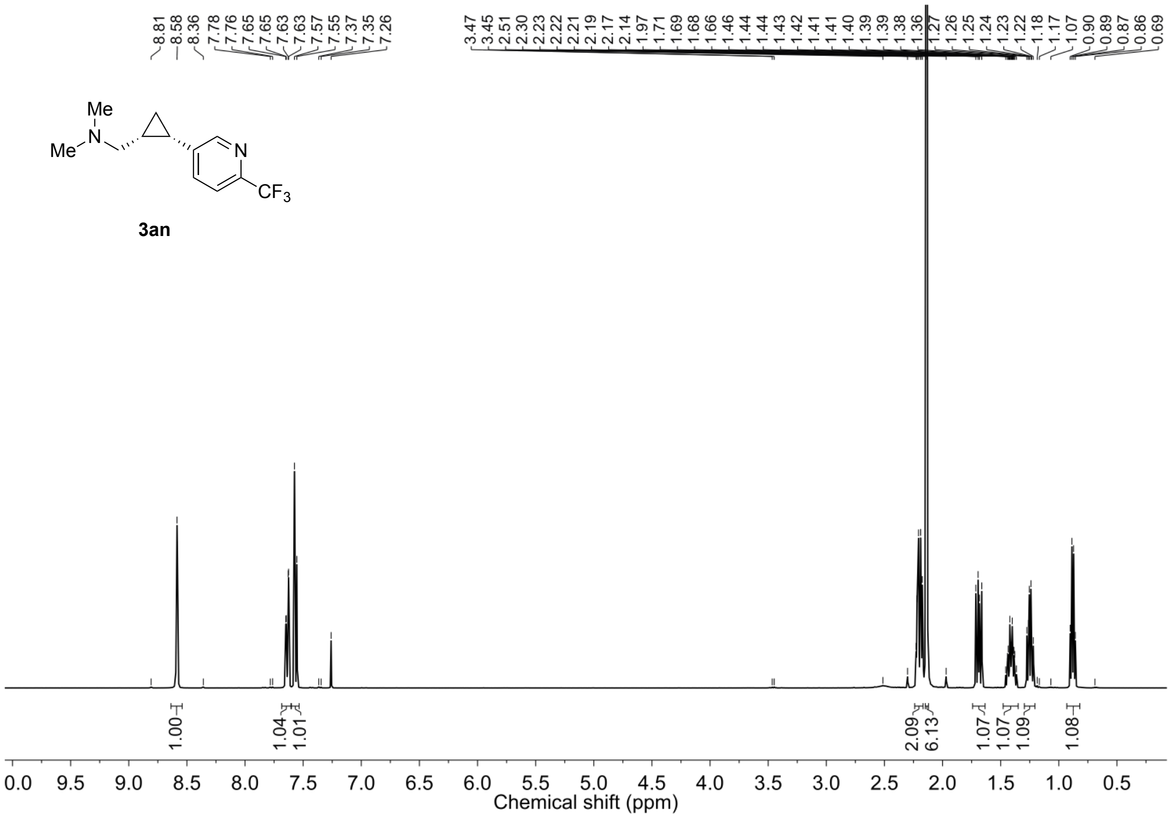

Me
N
Me
N
CF3
3an
8.81
8.58
8.36
7.78
7.76
7.65
7.65
7.63
7.63
7.57
7.55
7.37
7.35
7.26
3.47
3.45
2.51
2.30
2.23
2.22
2.21
2.19
2.17
2.14
1.97
1.71
1.69
1.68
1.66
1.46
1.44
1.44
1.43
1.42
1.41
1.41
1.40
1.39
1.39
1.38
1.36
1.27
1.26
1.25
1.24
1.23
1.22
1.18
1.17
1.07
0.90
0.89
0.87
0.86
0.69
1.00
1.04
1.01
2.09
6.13
1.07
1.07
1.09
1.08
Chemical shift (ppm)

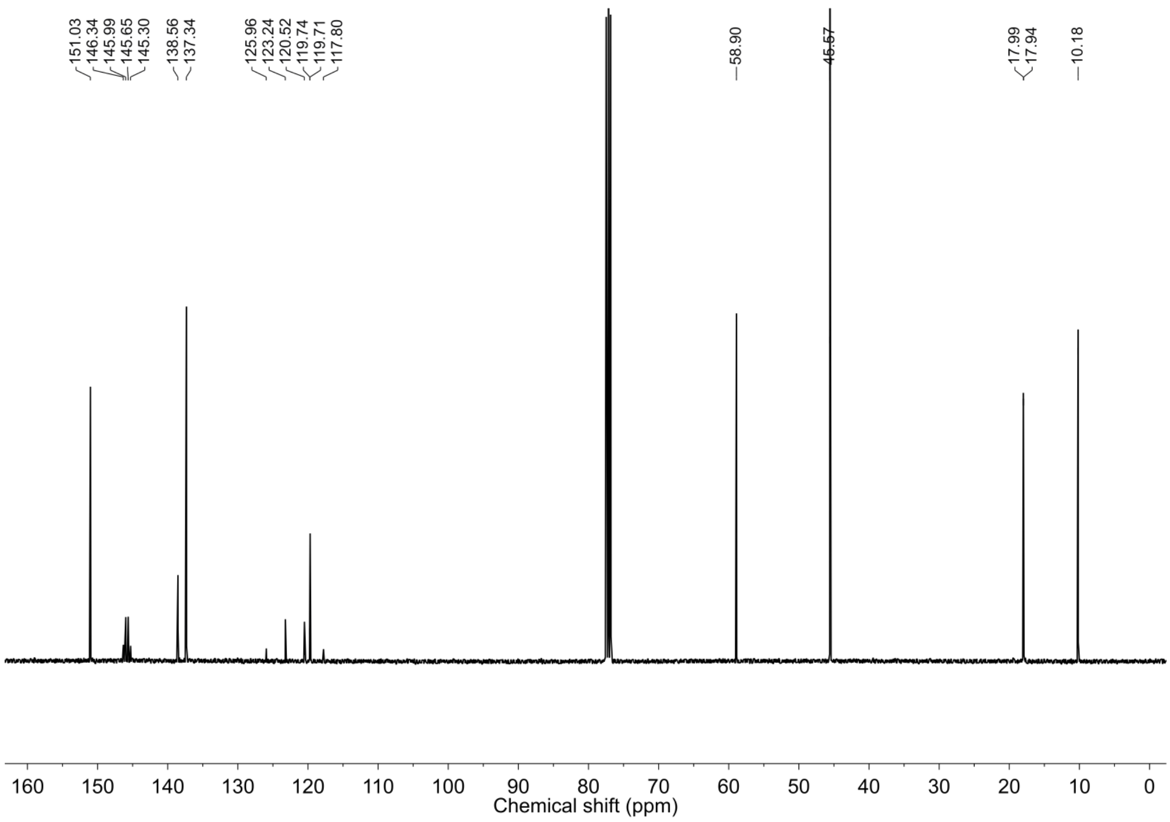

151.03
146.34
145.99
145.65
145.30
138.56
137.34
125.96
123.24
120.52
119.74
119.71
117.80
58.90
45.57
17.99
17.94
10.18
Chemical shift (ppm)

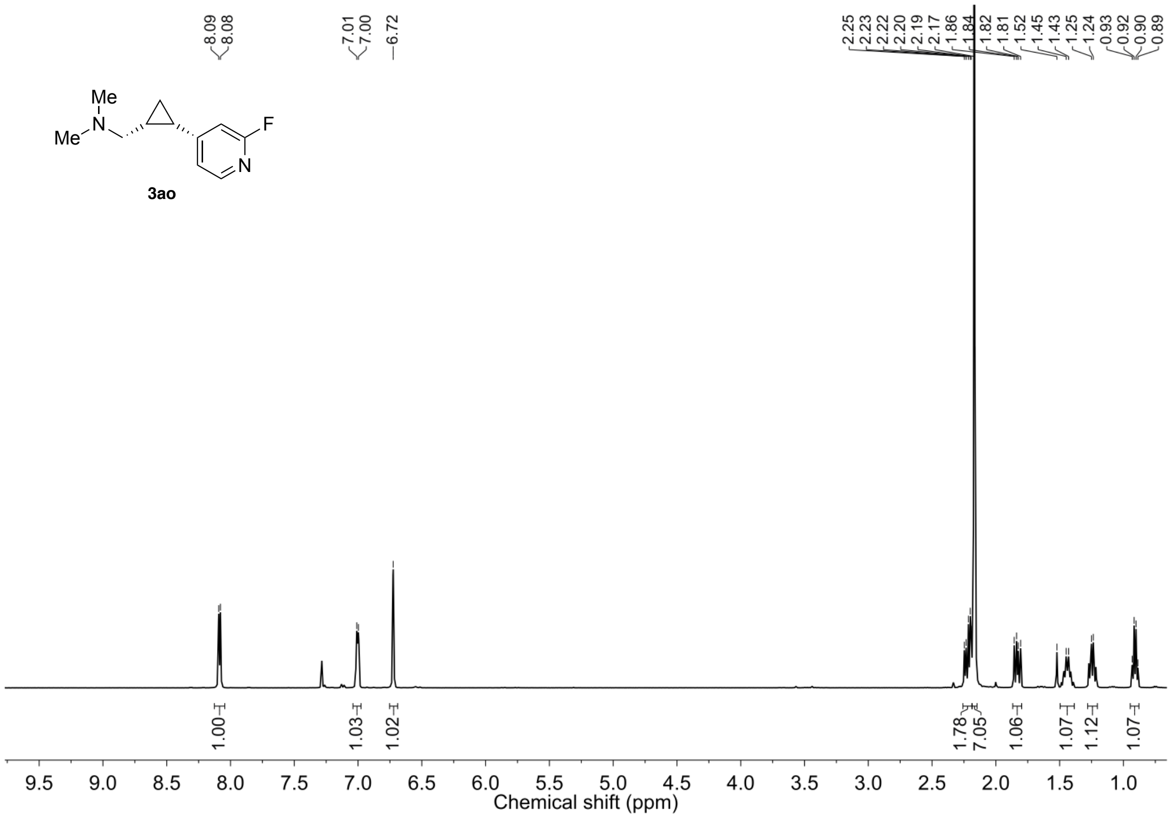
8.09
8.08
7.01
7.00
6.72
2.25
2.23
2.22
2.20
2.19
2.17
1.86
1.84
1.82
1.81
1.52
1.45
1.43
1.25
1.24
0.93
0.92
0.90
0.89
Me
Me
N
F
N
3ao
1.00
1.03
1.02
1.78
7.05
1.06
1.07
1.12
1.07
9.5
9.0
8.5
8.0
7.5
7.0
6.5
6.0
5.5
5.0
4.5
4.0
3.5
3.0
2.5
2.0
1.5
1.0
Chemical shift (ppm)

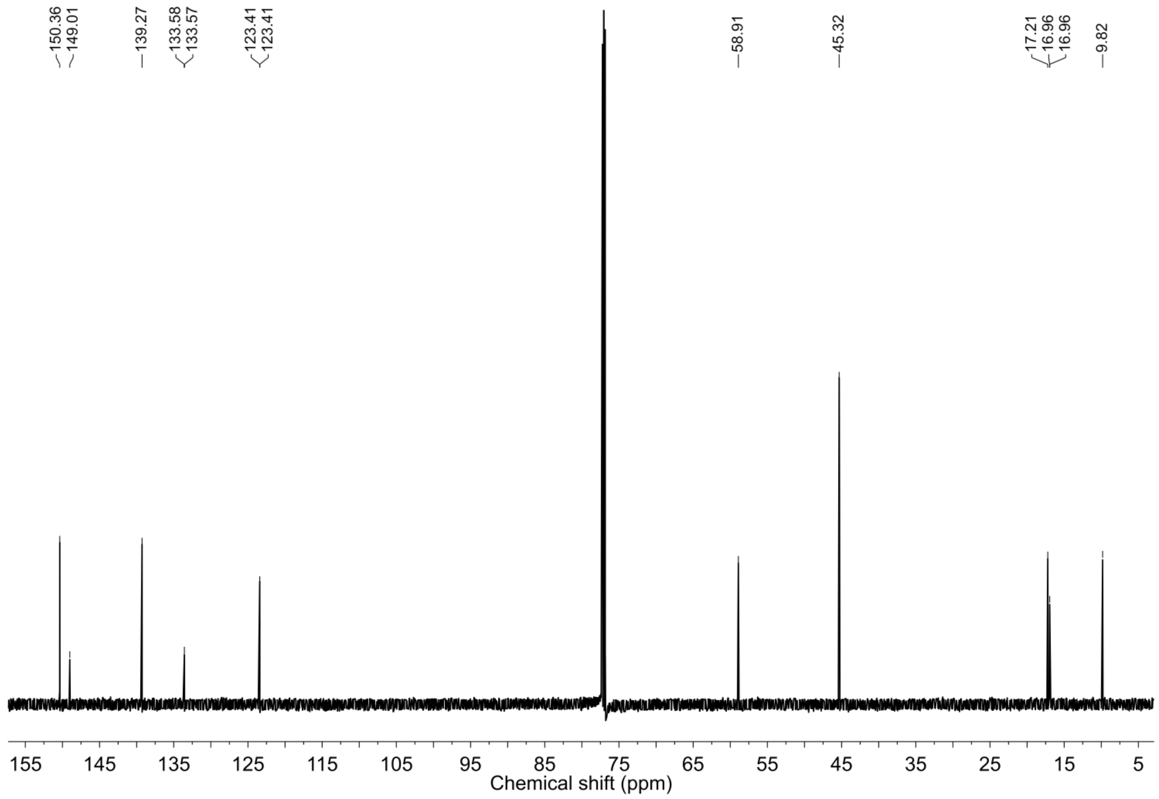
150.36
149.01
139.27
133.58
133.57
123.41
123.41
58.91
45.32
17.21
16.96
16.96
9.82
155
145
135
125
115
105
95
85
75
65
55
45
35
25
15
5
Chemical shift (ppm)

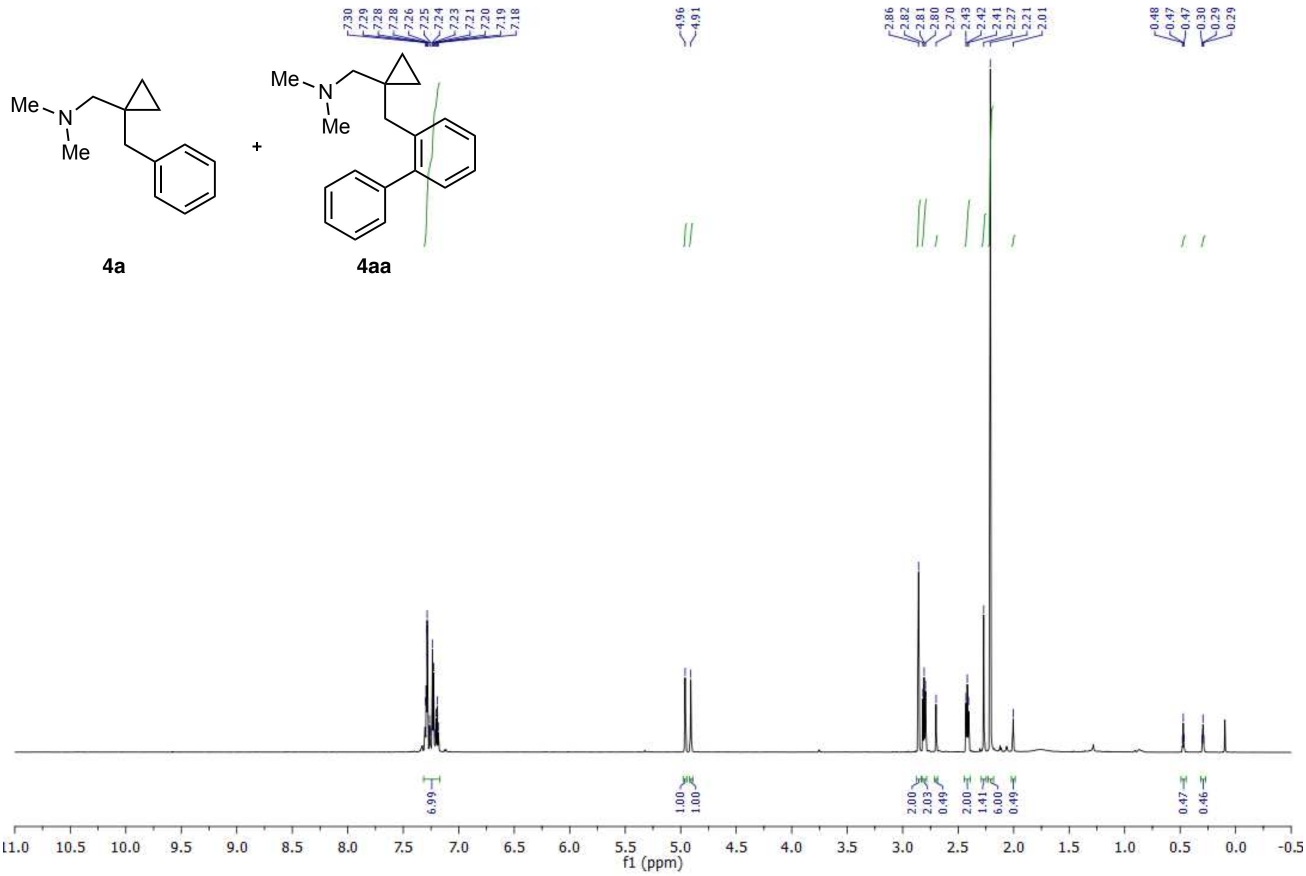
Me
N
Me
4a
+
Me
N
Me
4aa
7.30 7.29 7.28 7.28 7.26 7.25 7.24 7.23 7.21 7.20 7.19 7.18
4.96 4.91
2.86 2.82 2.81 2.80 2.70 2.43 2.42 2.41 2.27 2.21 2.01
0.48 0.47 0.47 0.30 0.29 0.29
6.99
1.00 1.00
2.00 2.03 0.49 2.00 1.41 6.00 0.49
0.47 0.46
f1 (ppm)

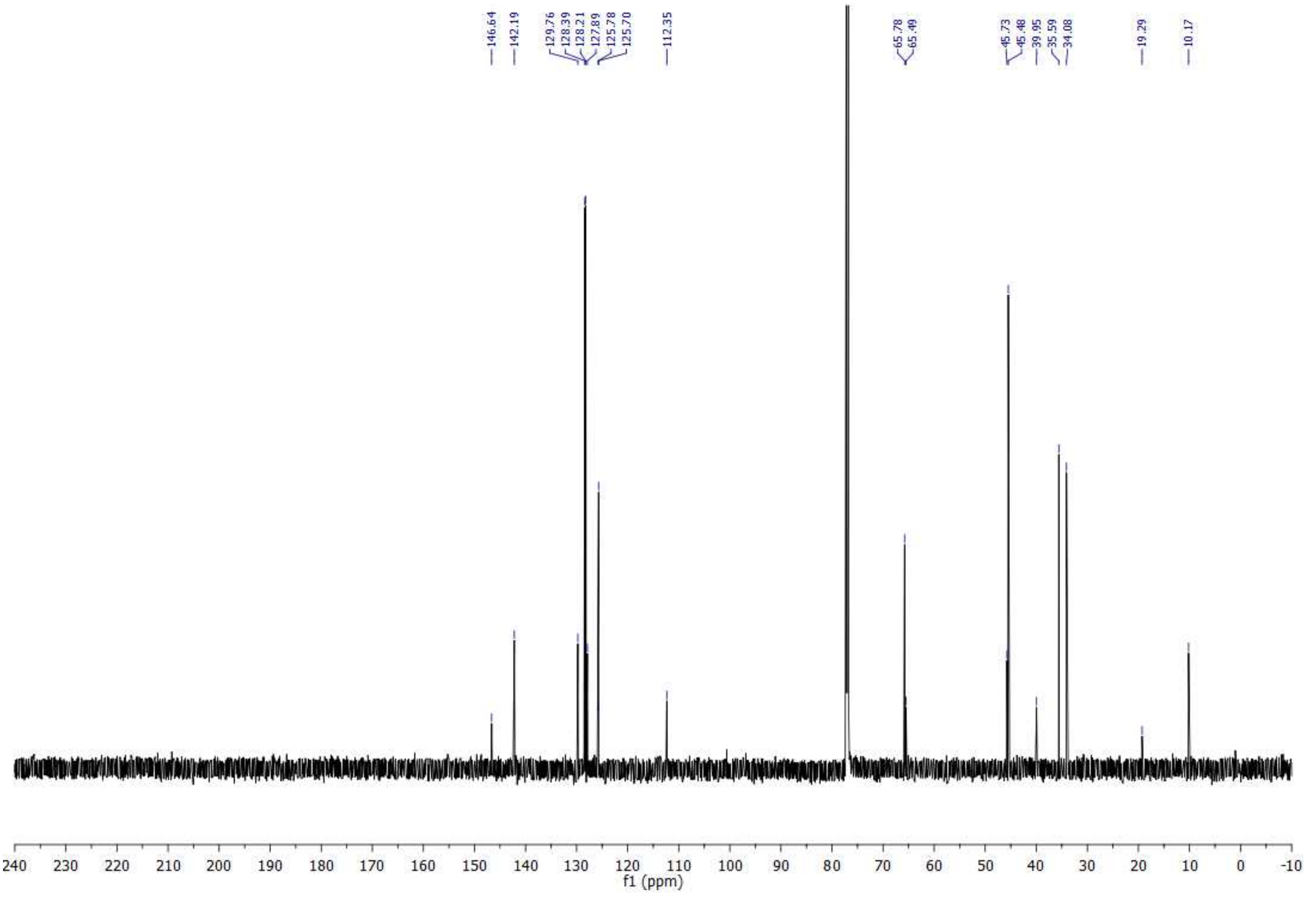
146.64
142.19
129.76 128.39 128.21 127.89 125.78 125.70
112.35
65.78 65.49
45.73 45.48 39.95 35.59 34.08
19.29
10.17
f1 (ppm)

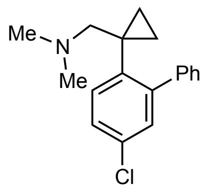
Me
N
Me
Ph
Cl

4b

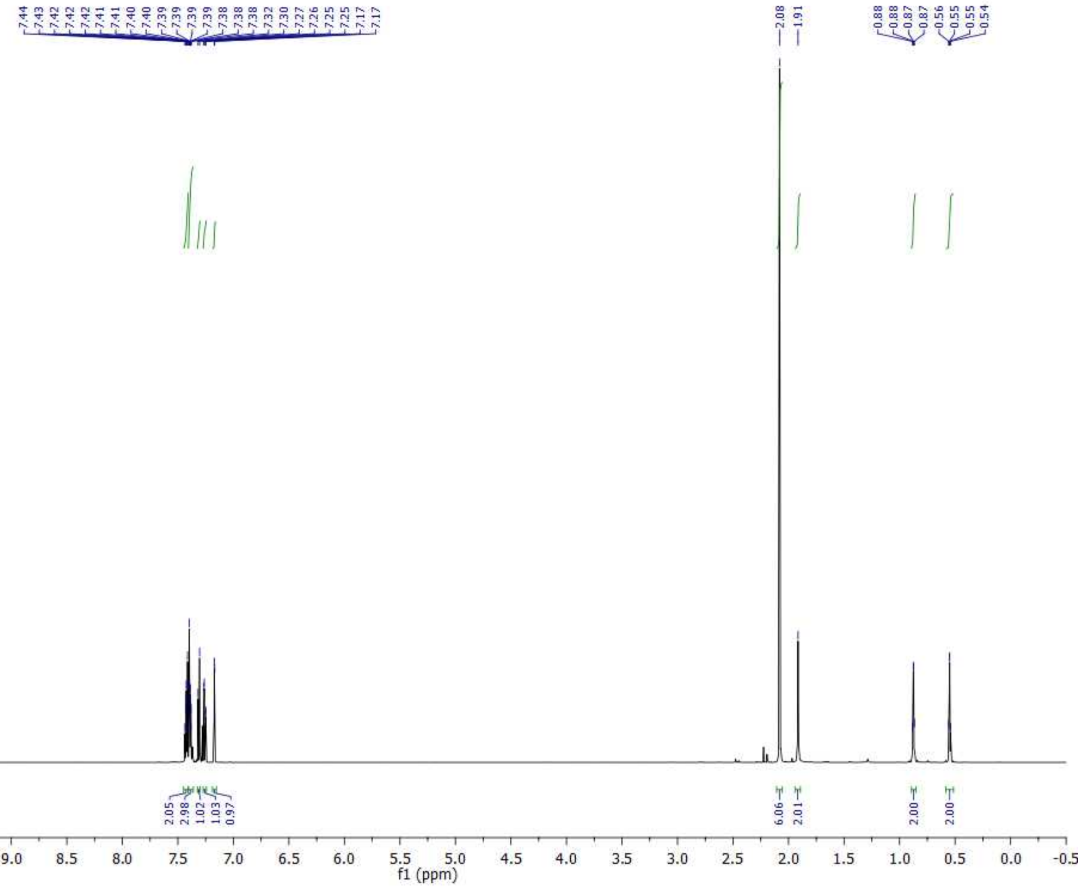
7.44
7.43
7.42
7.42
7.42
7.41
7.41
7.40
7.39
7.39
7.39
7.39
7.38
7.38
7.38
7.32
7.30
7.27
7.26
7.25
7.25
7.17
7.17
2.08
1.91
0.88
0.88
0.87
0.87
0.56
0.55
0.55
0.54
2.05
2.98
1.02
1.03
0.97
6.06
2.01
2.00
2.00
f1 (ppm)

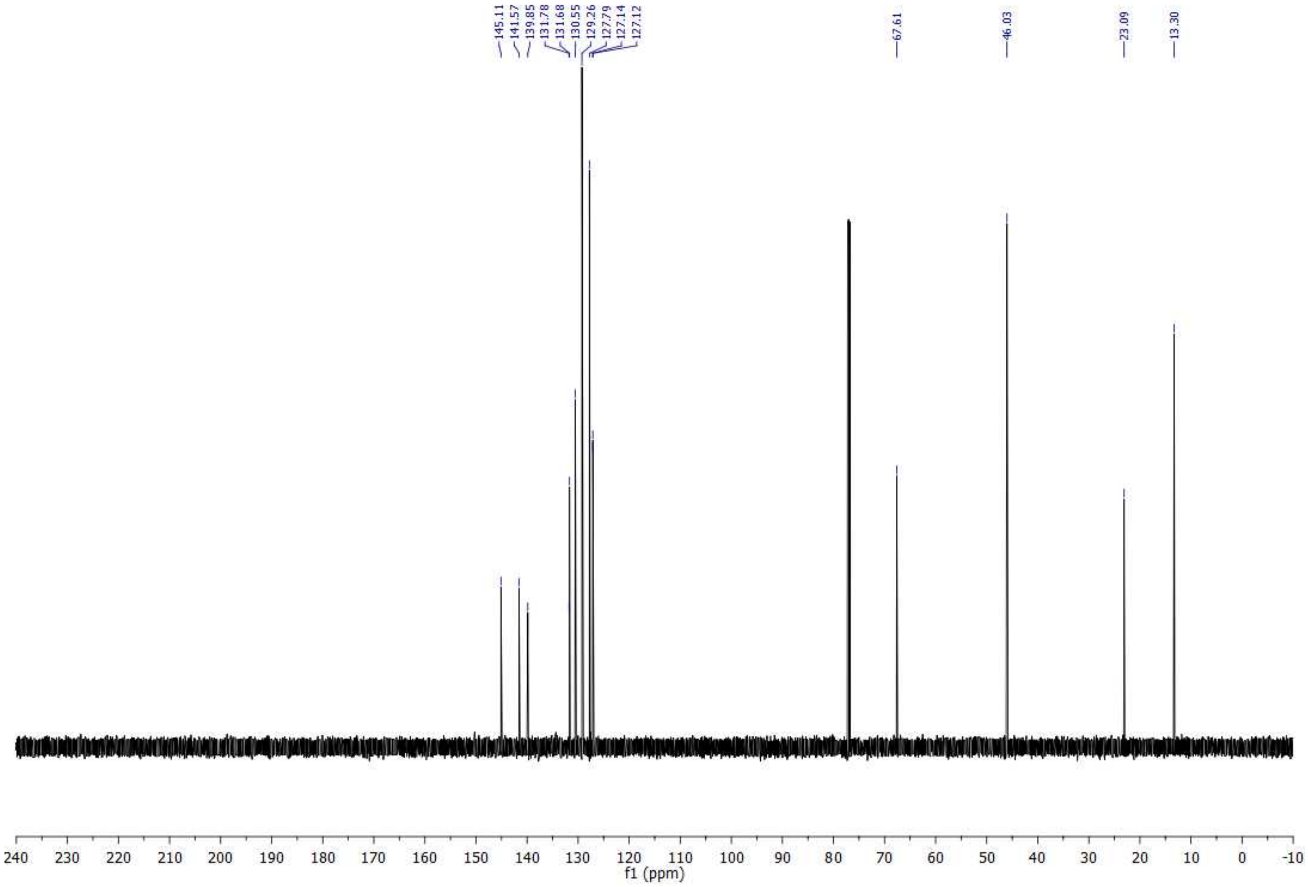
145.11
141.57
139.85
131.78
131.68
130.55
129.26
127.79
127.14
127.12
67.61
46.03
23.09
13.30
f1 (ppm)

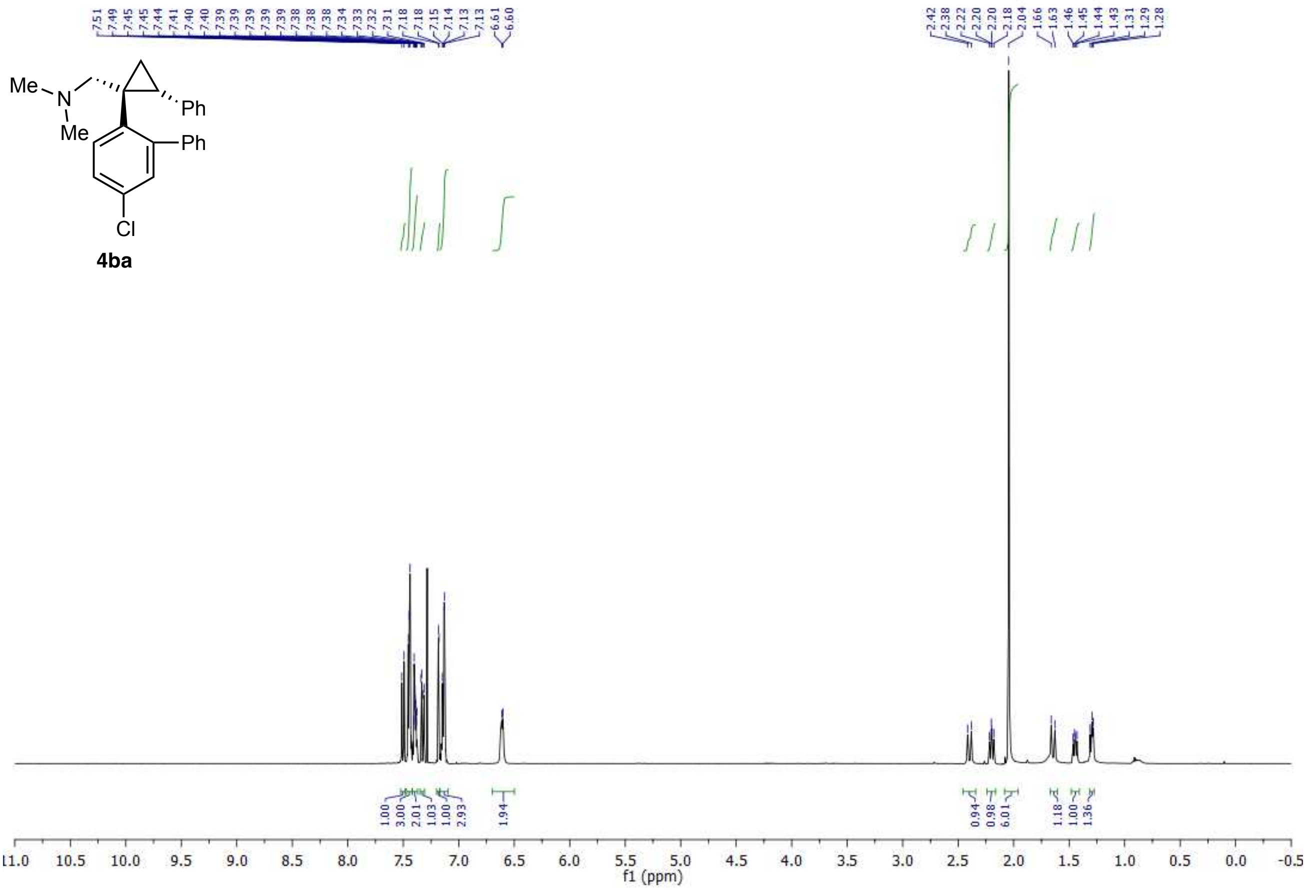

4ba

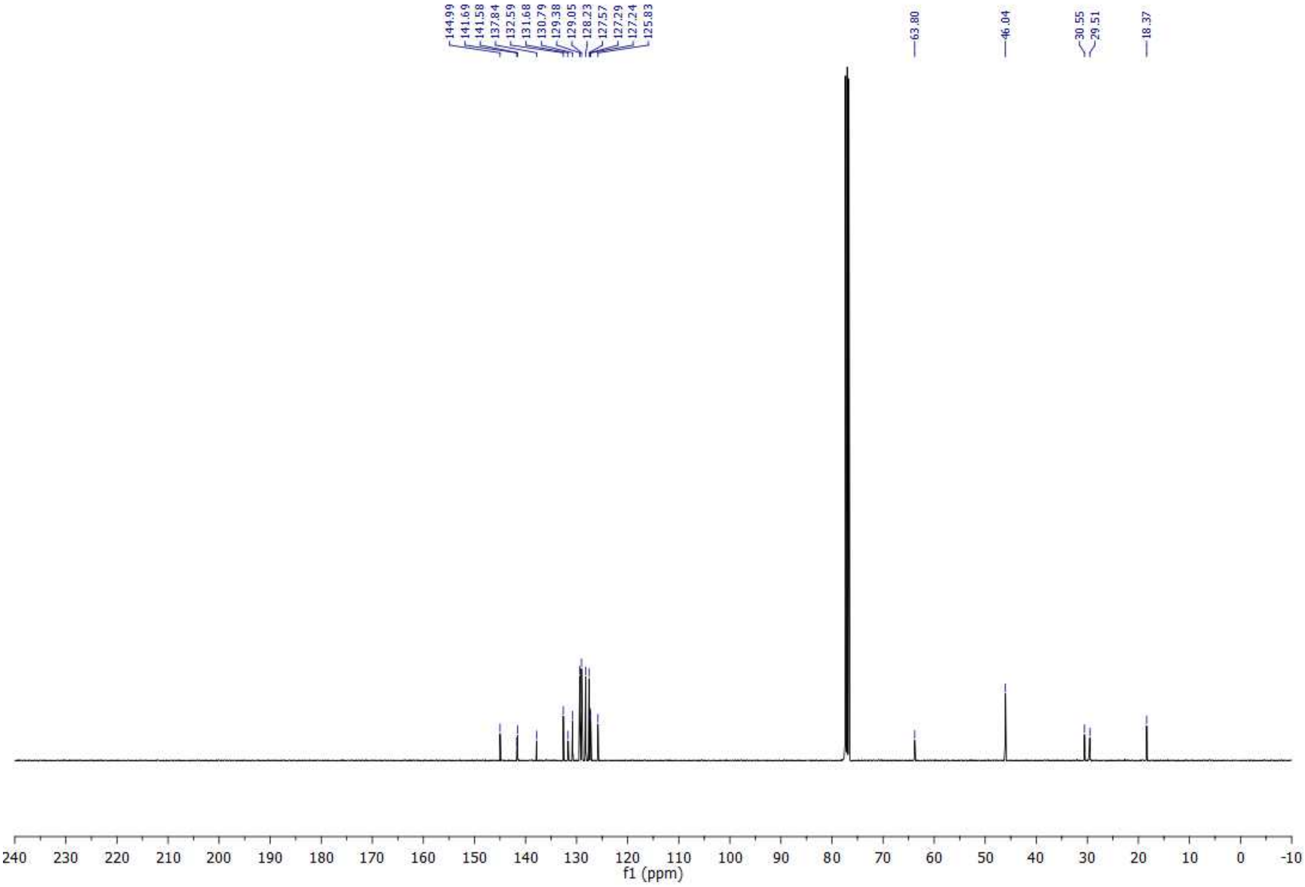

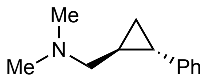

5

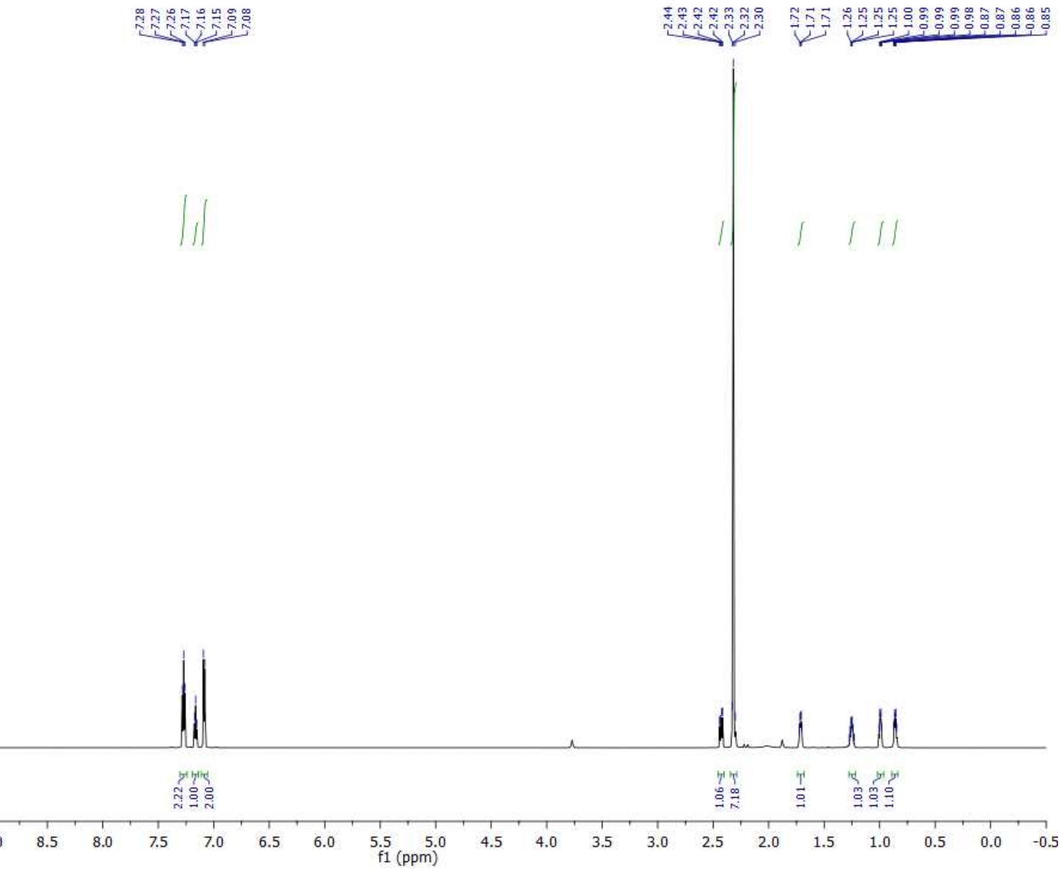

7.28
7.27
7.26
7.17
7.16
7.15
7.09
7.08
2.44
2.43
2.42
2.42
2.33
2.32
2.30
1.72
1.71
1.71
1.26
1.25
1.25
1.25
1.00
0.99
0.99
0.99
0.98
0.87
0.87
0.86
0.85
2.22
1.00
2.00
1.06
7.18
1.01
1.03
1.03
1.10
f1 (ppm)

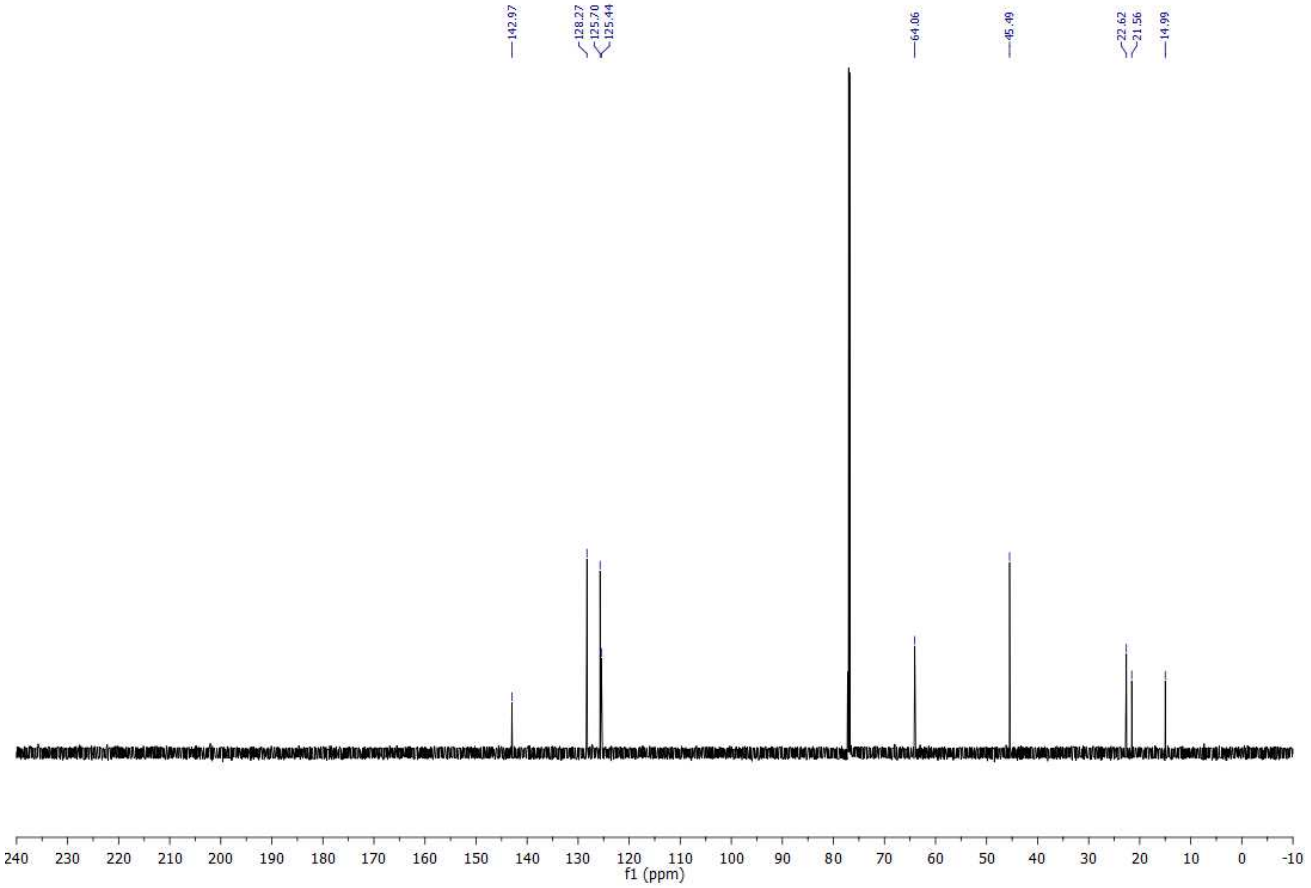

142.97
128.27
125.70
125.44
64.06
45.49
22.62
21.56
14.99
f1 (ppm)

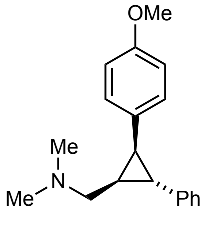

6

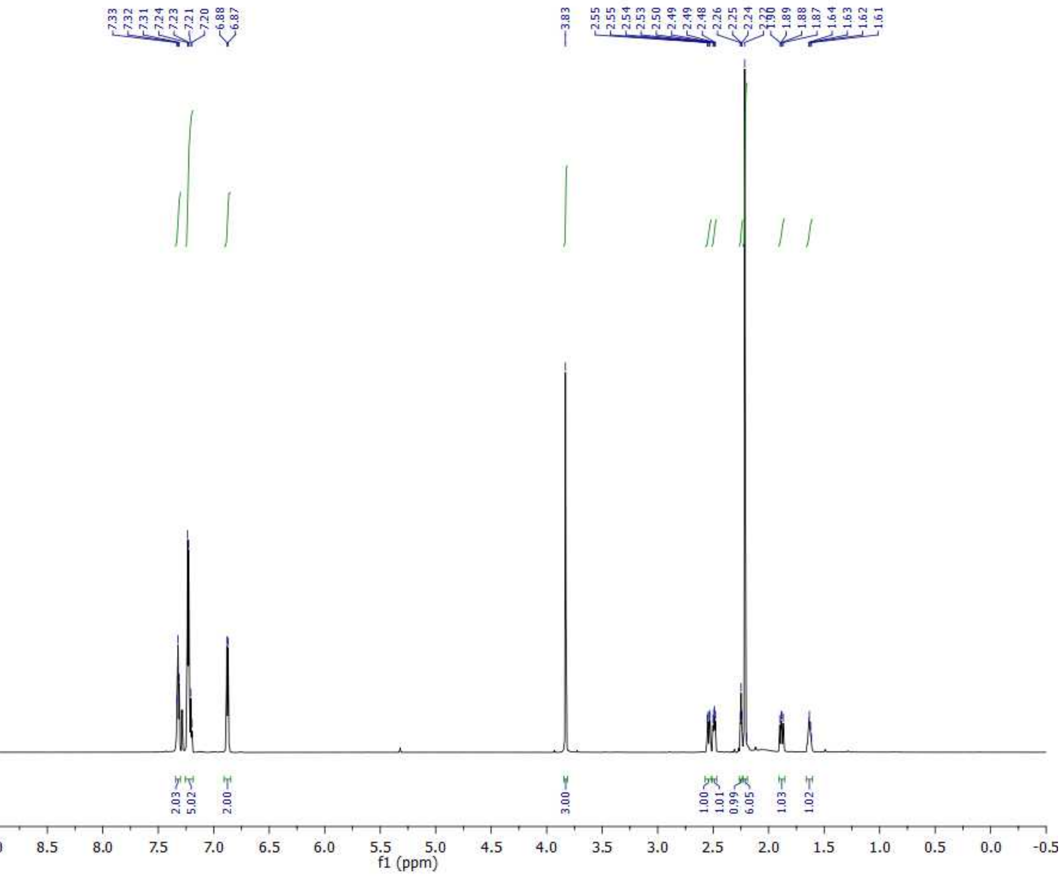

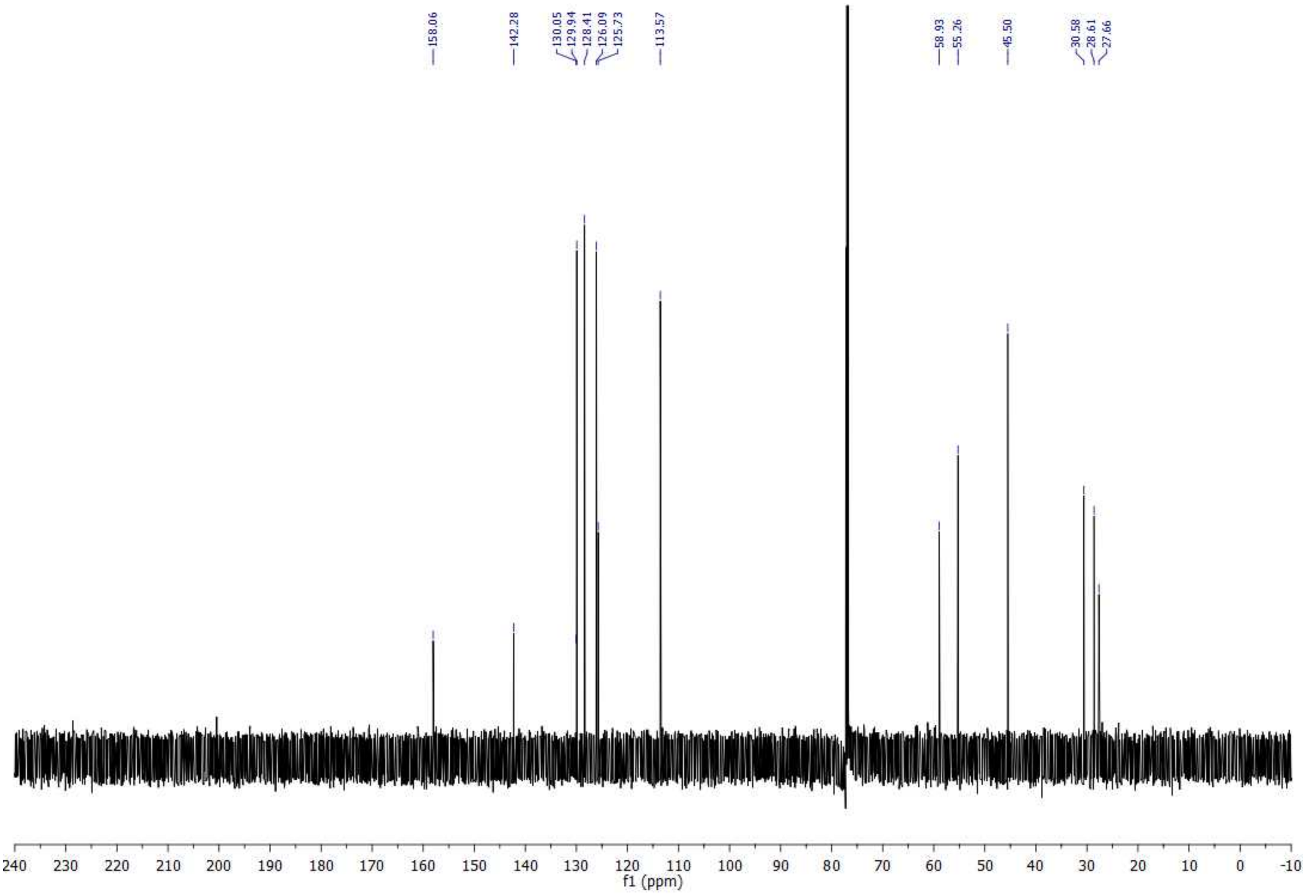

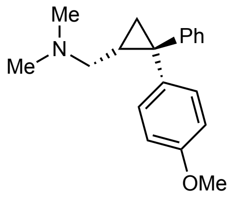

7

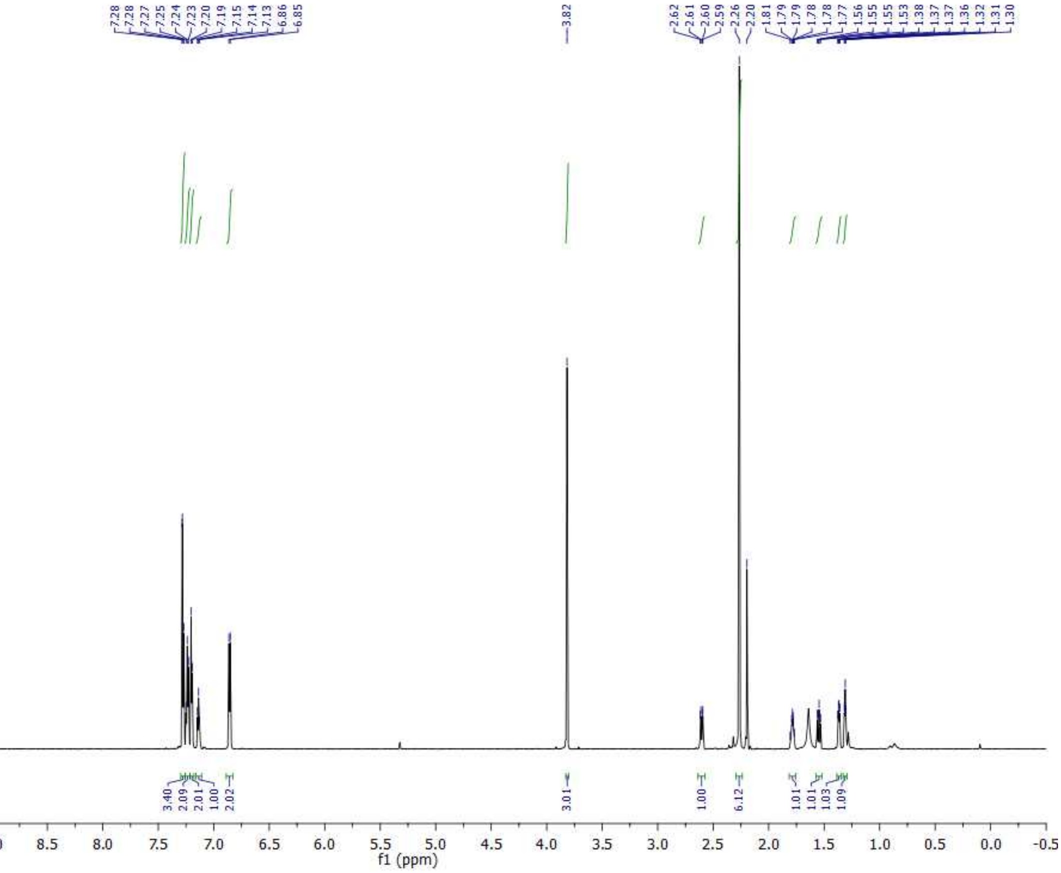

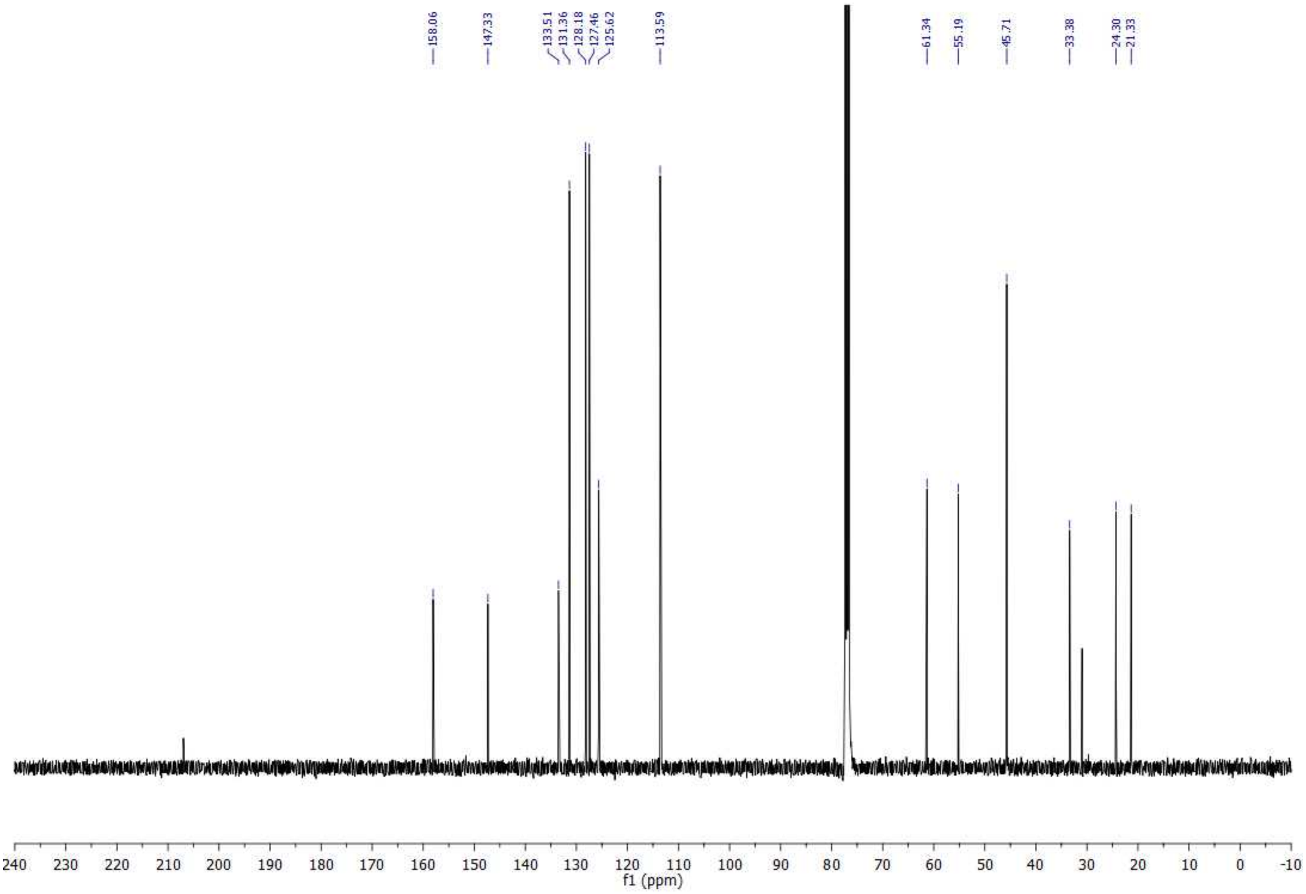

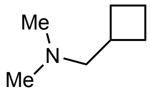

8a

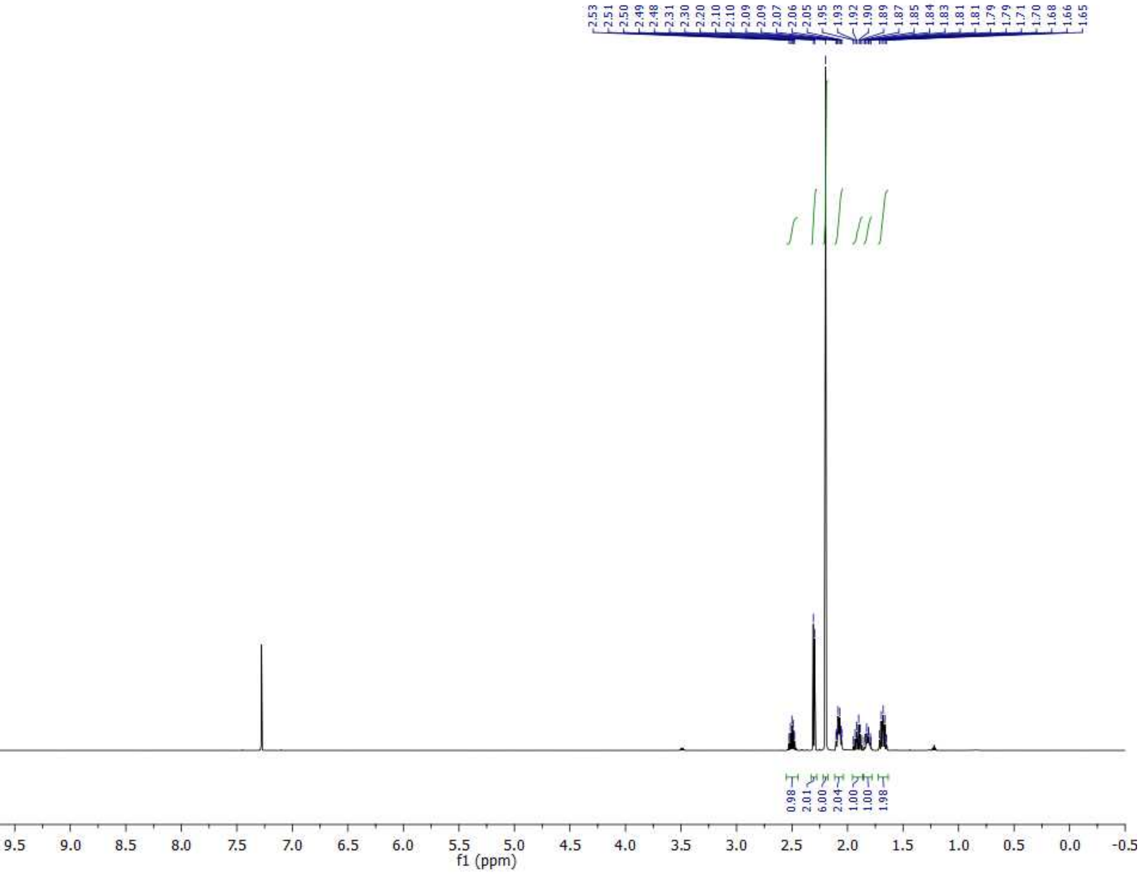

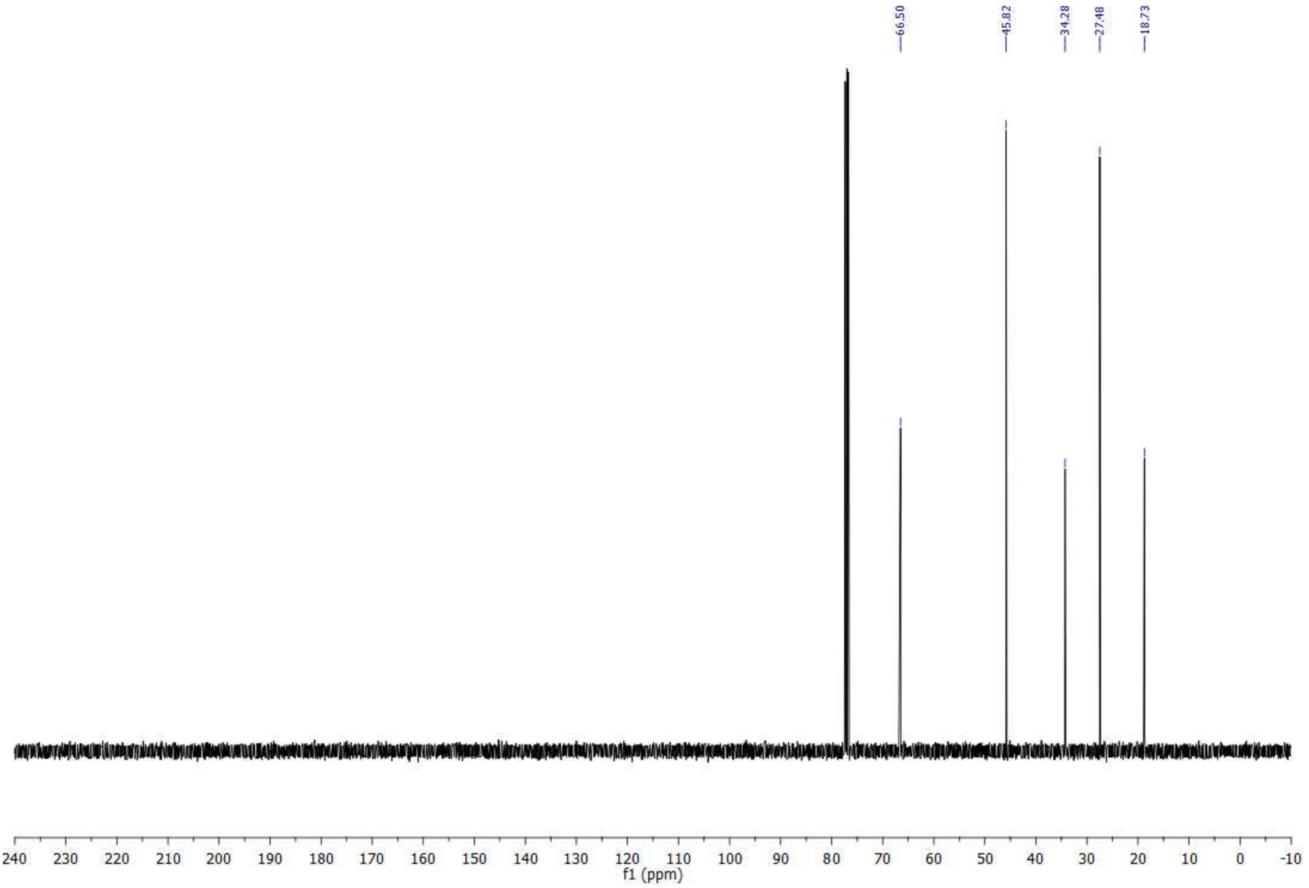

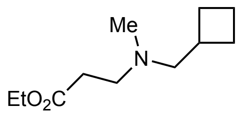

8b

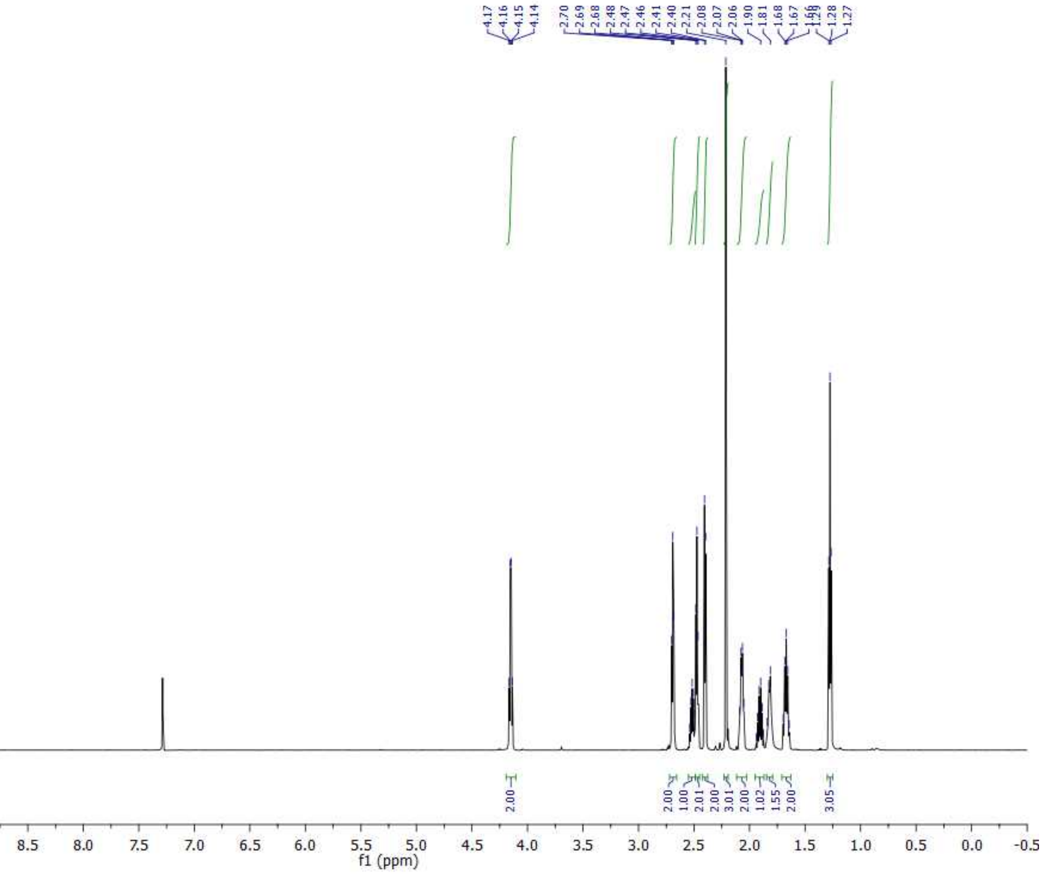

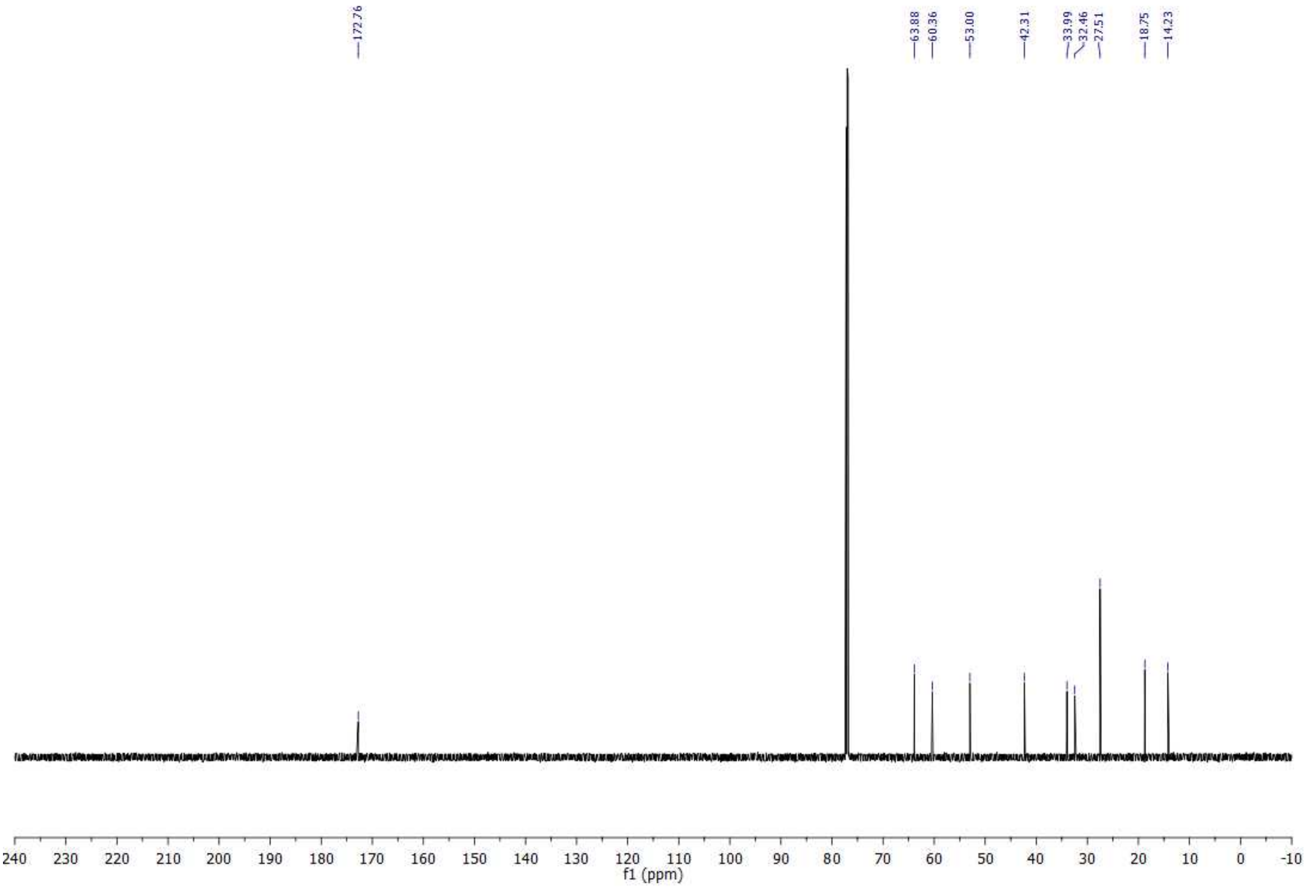

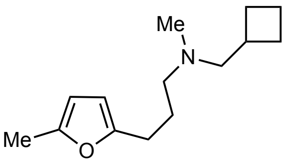
Me
N
Me
O

8c

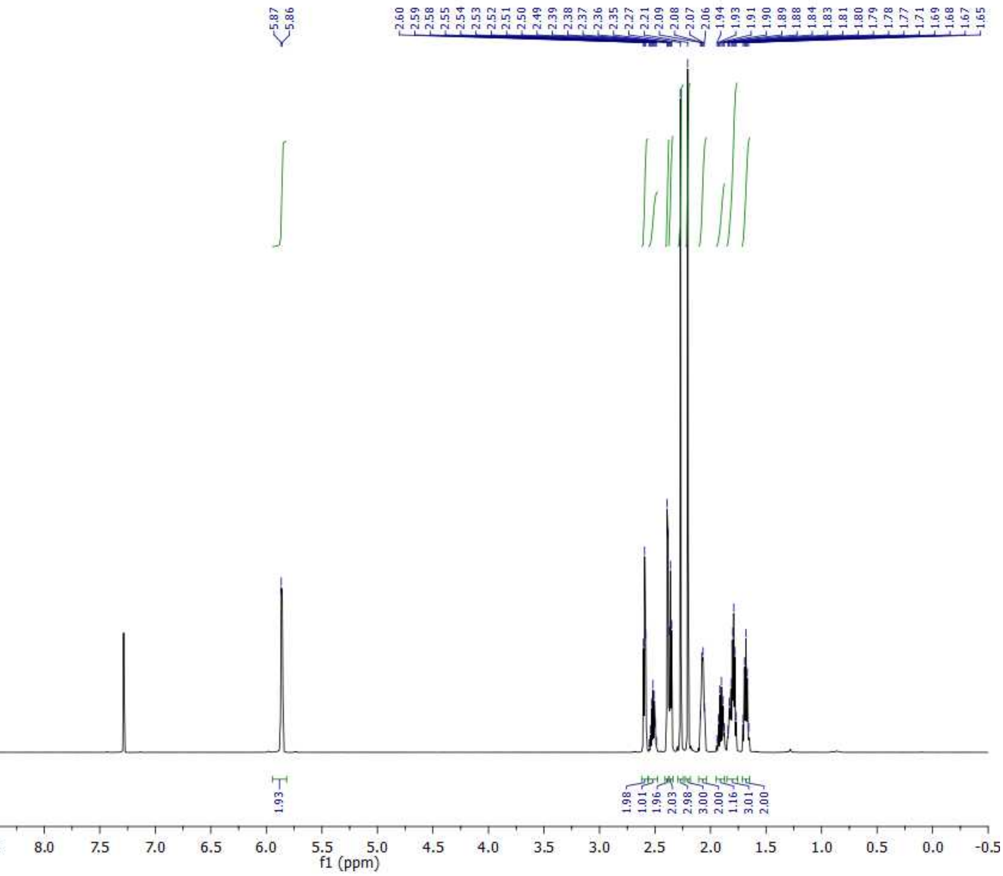
5.87
5.86
2.60
2.59
2.58
2.55
2.54
2.53
2.52
2.51
2.50
2.49
2.39
2.38
2.37
2.36
2.35
2.27
2.21
2.09
2.08
2.07
2.06
1.94
1.93
1.91
1.90
1.89
1.88
1.84
1.83
1.81
1.80
1.79
1.78
1.77
1.71
1.69
1.68
1.67
1.65
1.93
1.98
1.01
1.96
2.03
2.98
3.00
2.00
1.16
3.01
2.00
f1 (ppm)

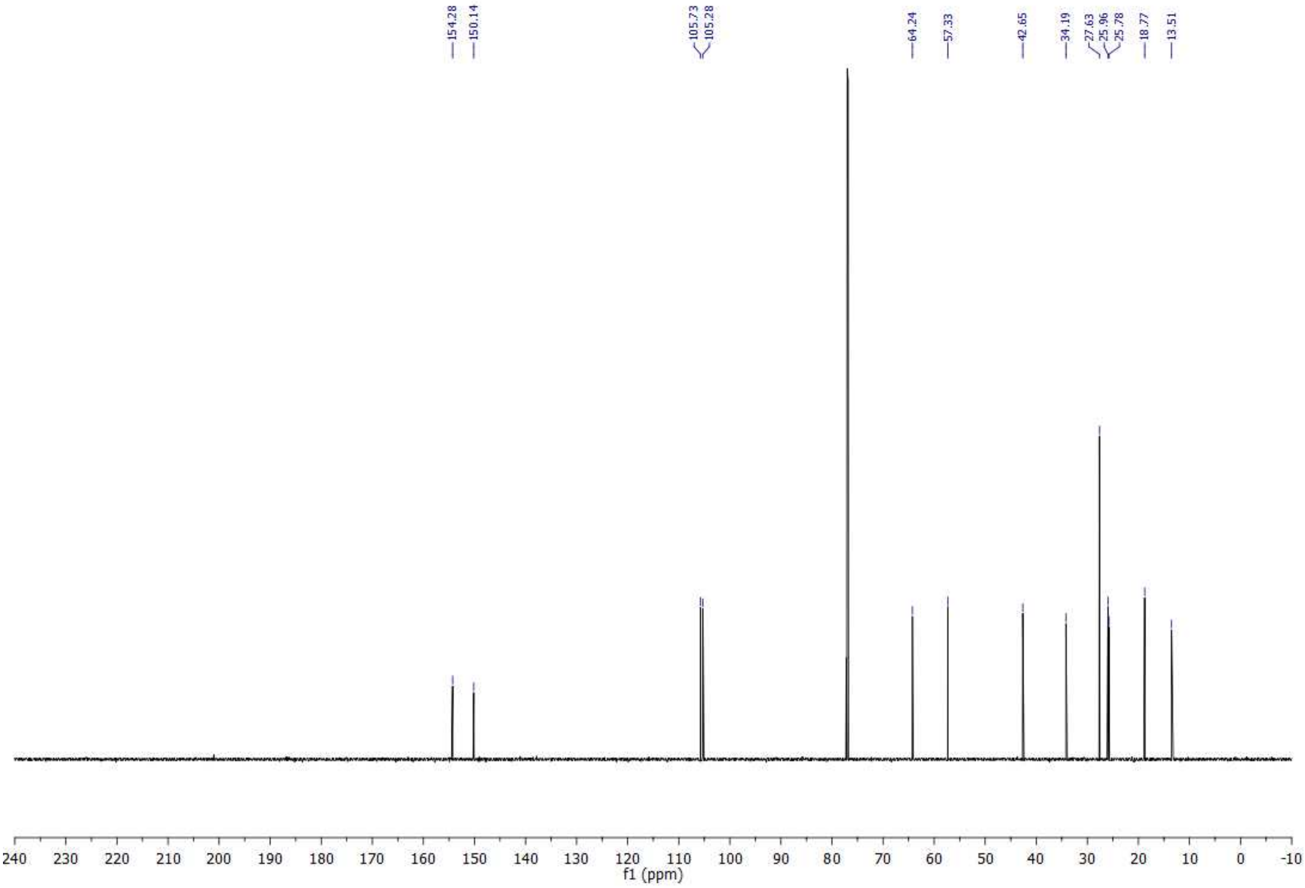
154.28
150.14
105.73
105.28
64.24
57.33
42.65
34.19
27.63
25.96
25.78
18.77
13.51
f1 (ppm)

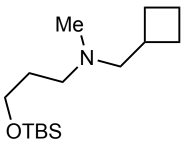

8d

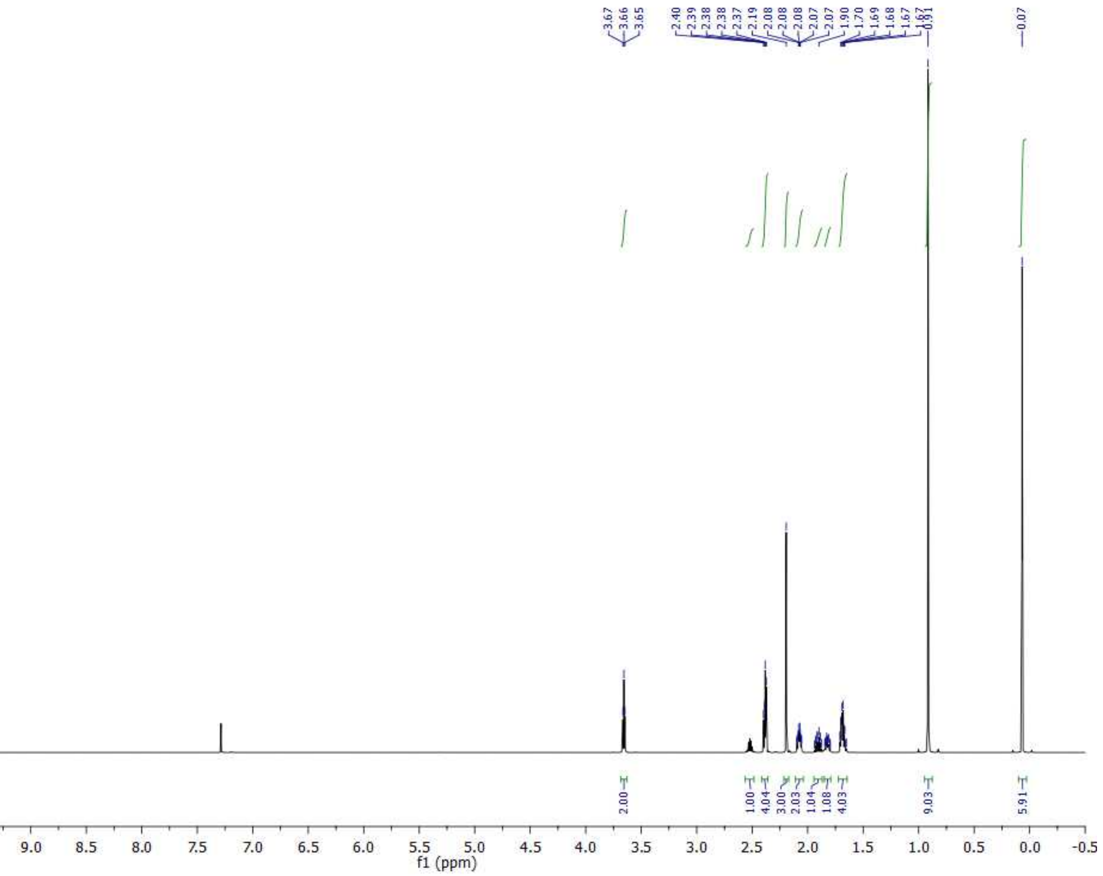

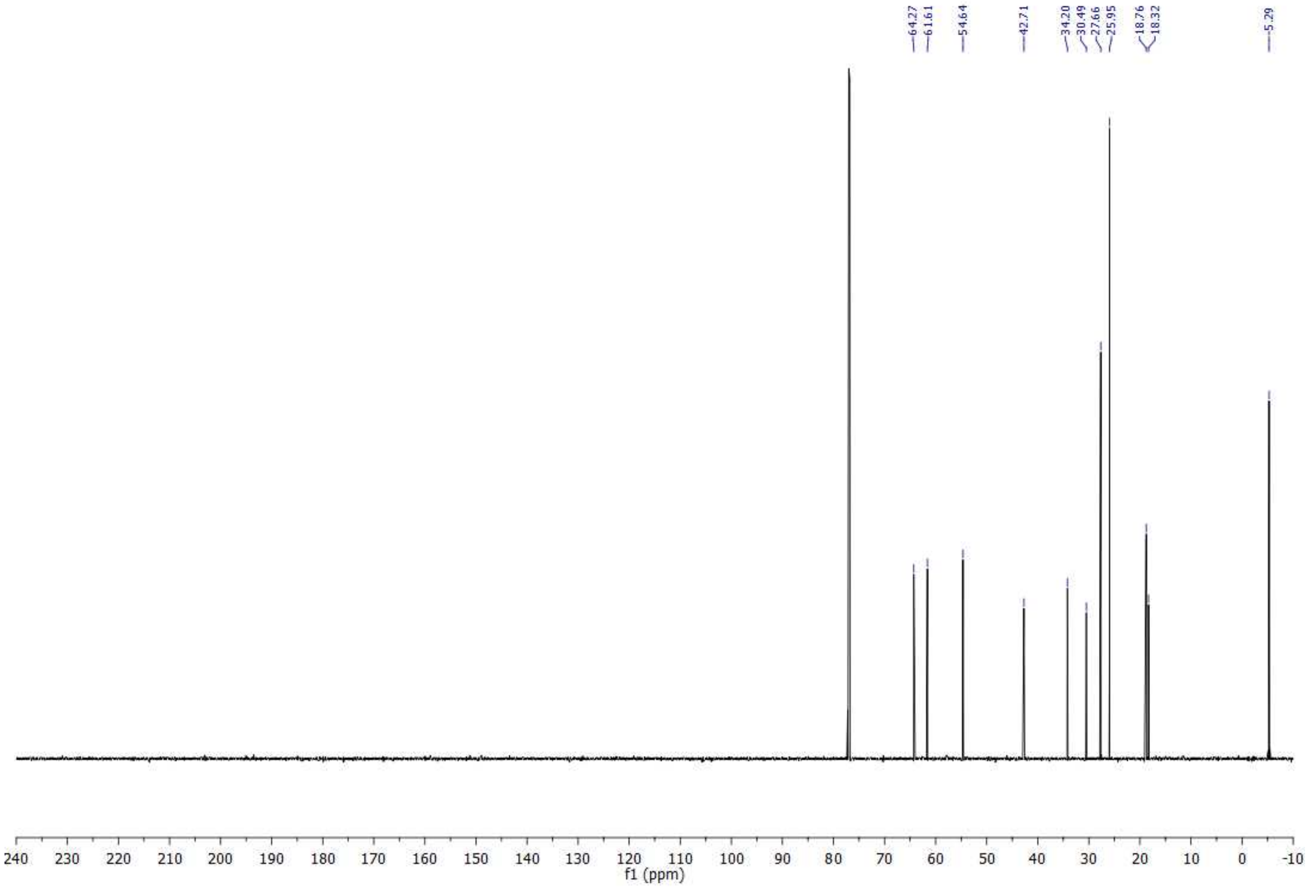

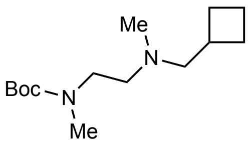

8e

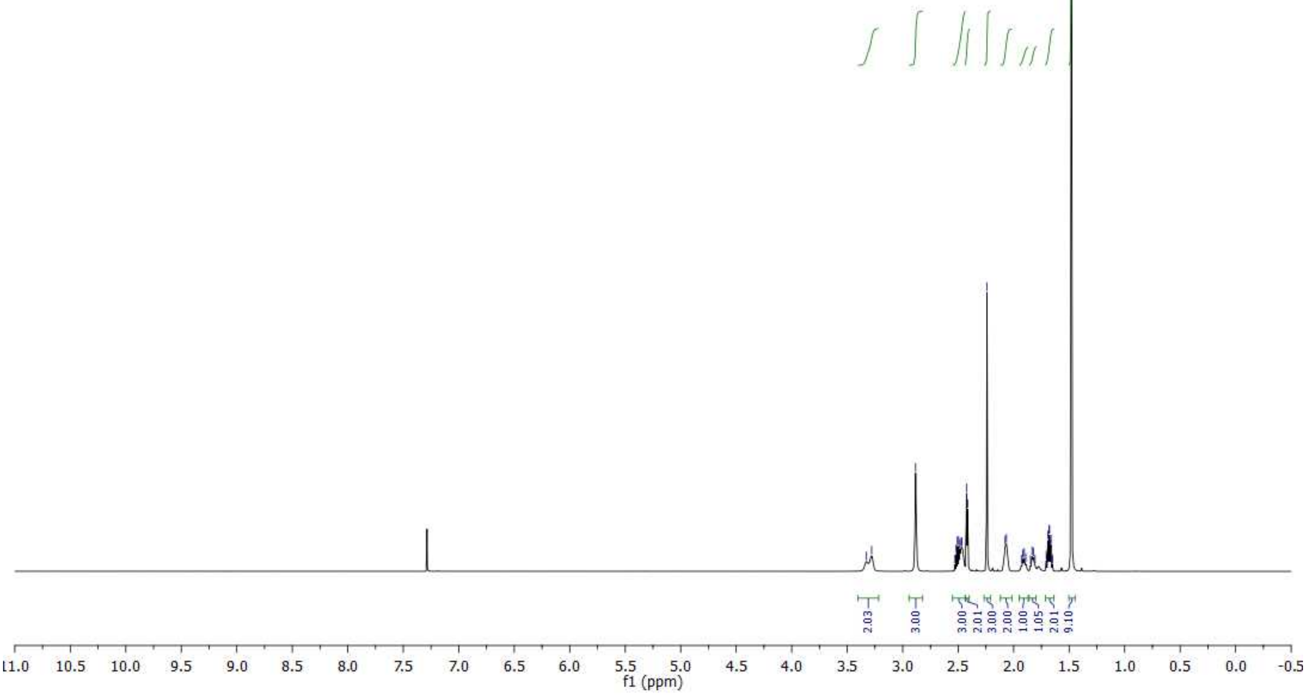

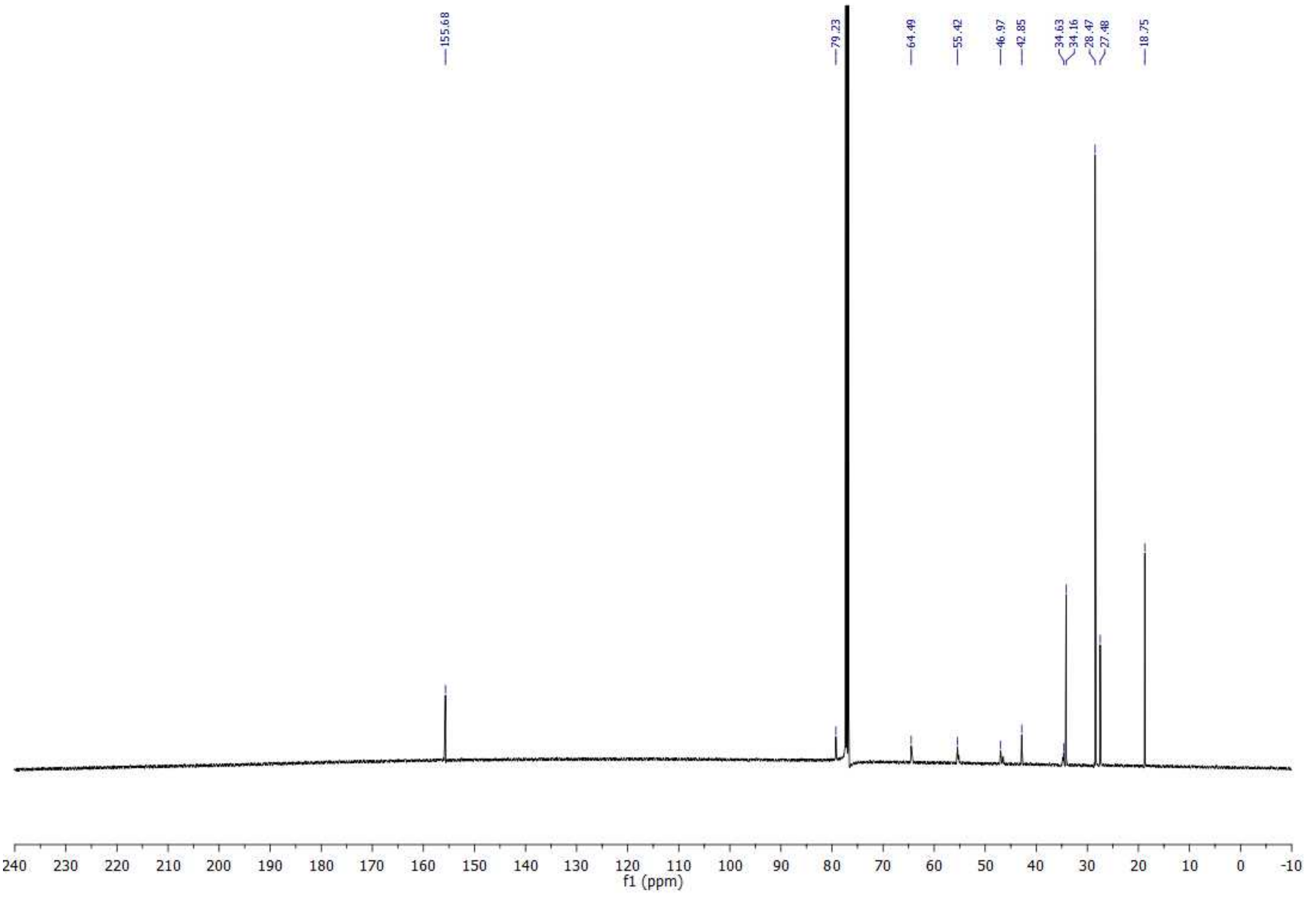

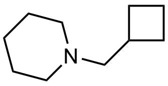

8f

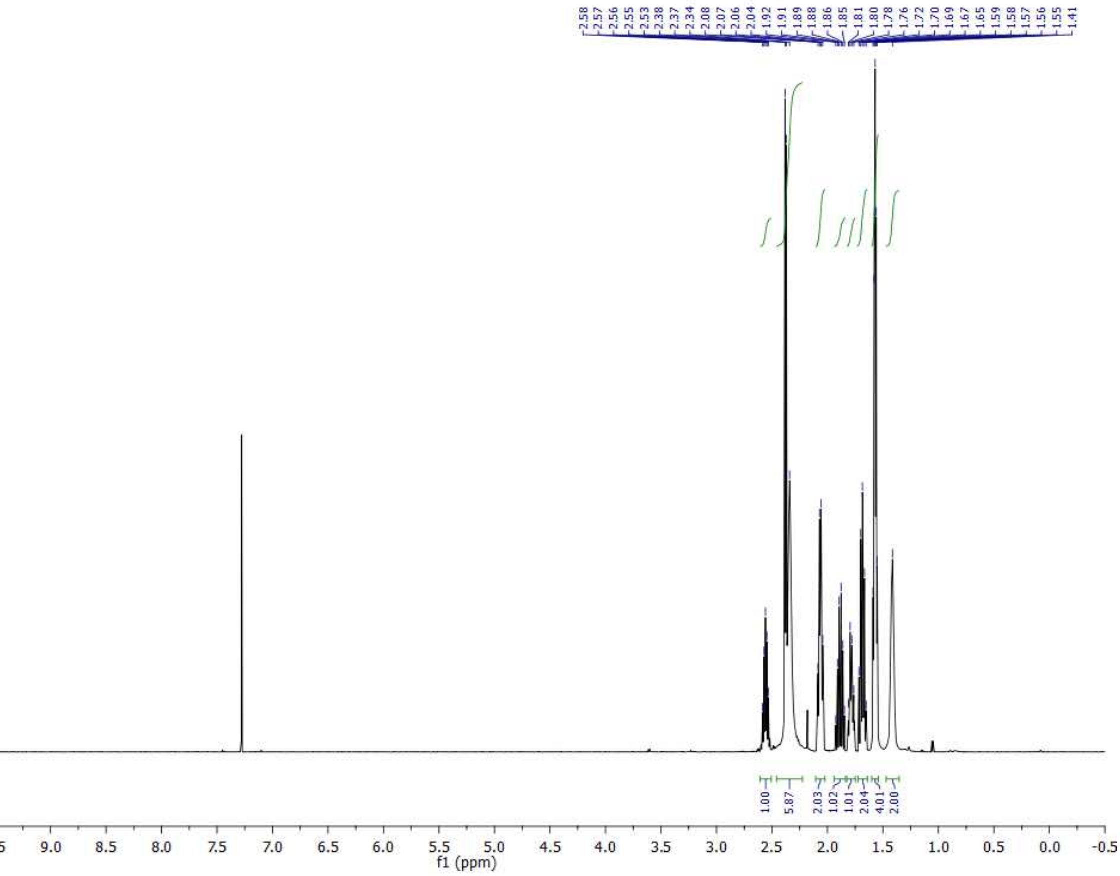
2.58 2.57 2.56 2.55 2.53 2.38 2.37 2.34 2.08 2.07 2.06 2.04 1.92 1.91 1.89 1.88 1.86 1.85 1.81 1.80 1.78 1.76 1.72 1.70 1.69 1.67 1.65 1.59 1.58 1.57 1.56 1.55 1.41
1.00 5.87 2.03 1.02 1.01 2.04 4.01 2.00
11.0 10.5 10.0 9.5 9.0 8.5 8.0 7.5 7.0 6.5 6.0 5.5 5.0 4.5 4.0 3.5 3.0 2.5 2.0 1.5 1.0 0.5 0.0 -0.5
f1 (ppm)

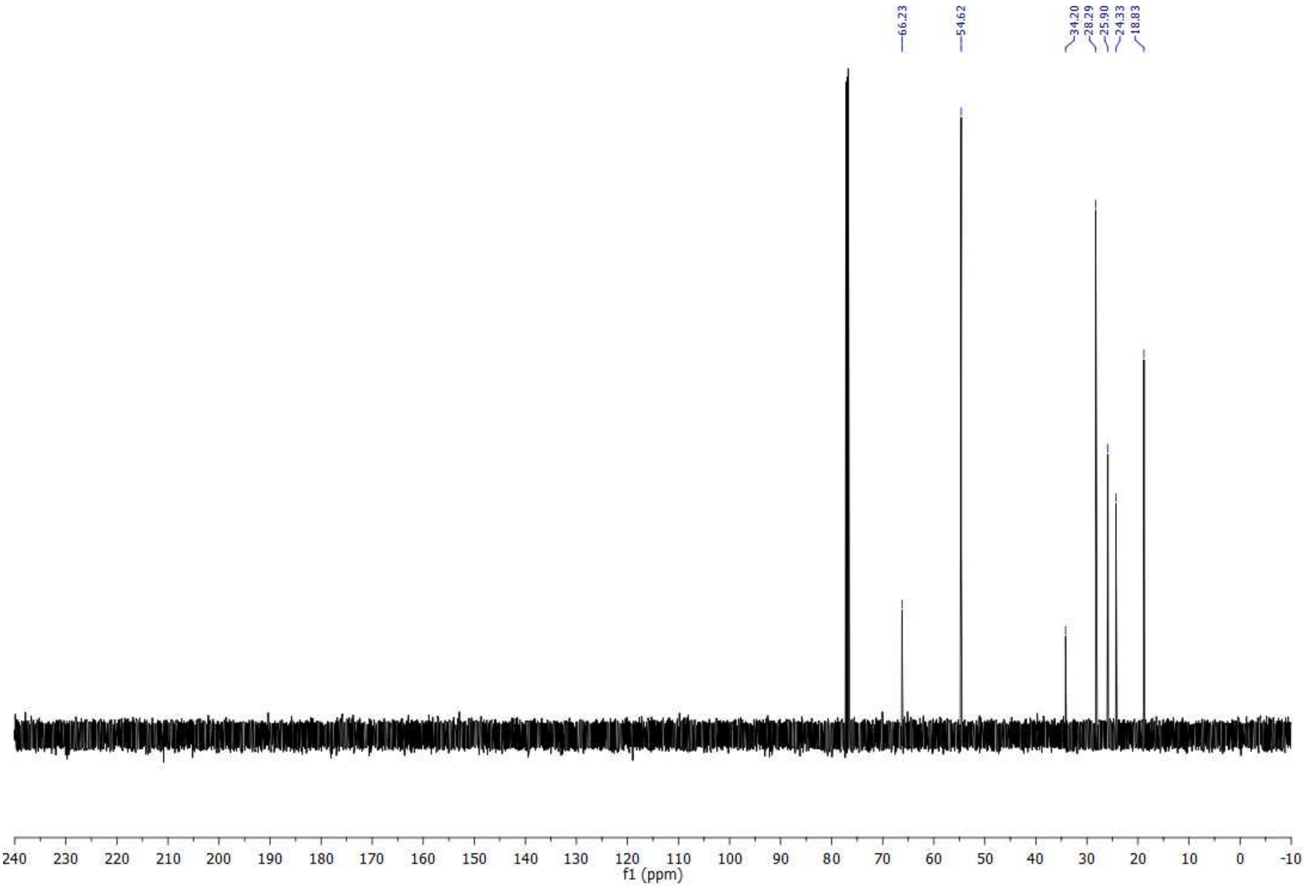
66.23
54.62
34.20 28.29 25.90 24.33 18.83
240 230 220 210 200 190 180 170 160 150 140 130 120 110 100 90 80 70 60 50 40 30 20 10 0 -10
f1 (ppm)

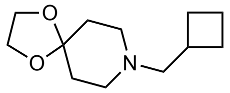

8g

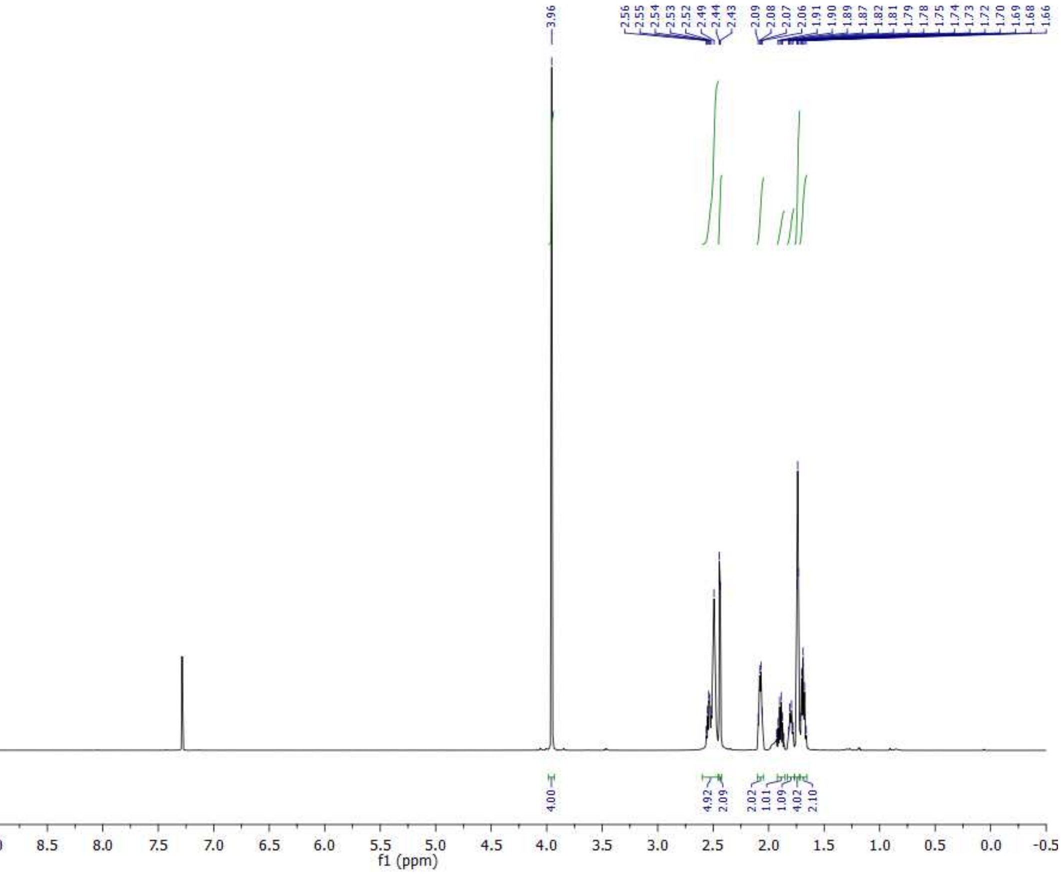

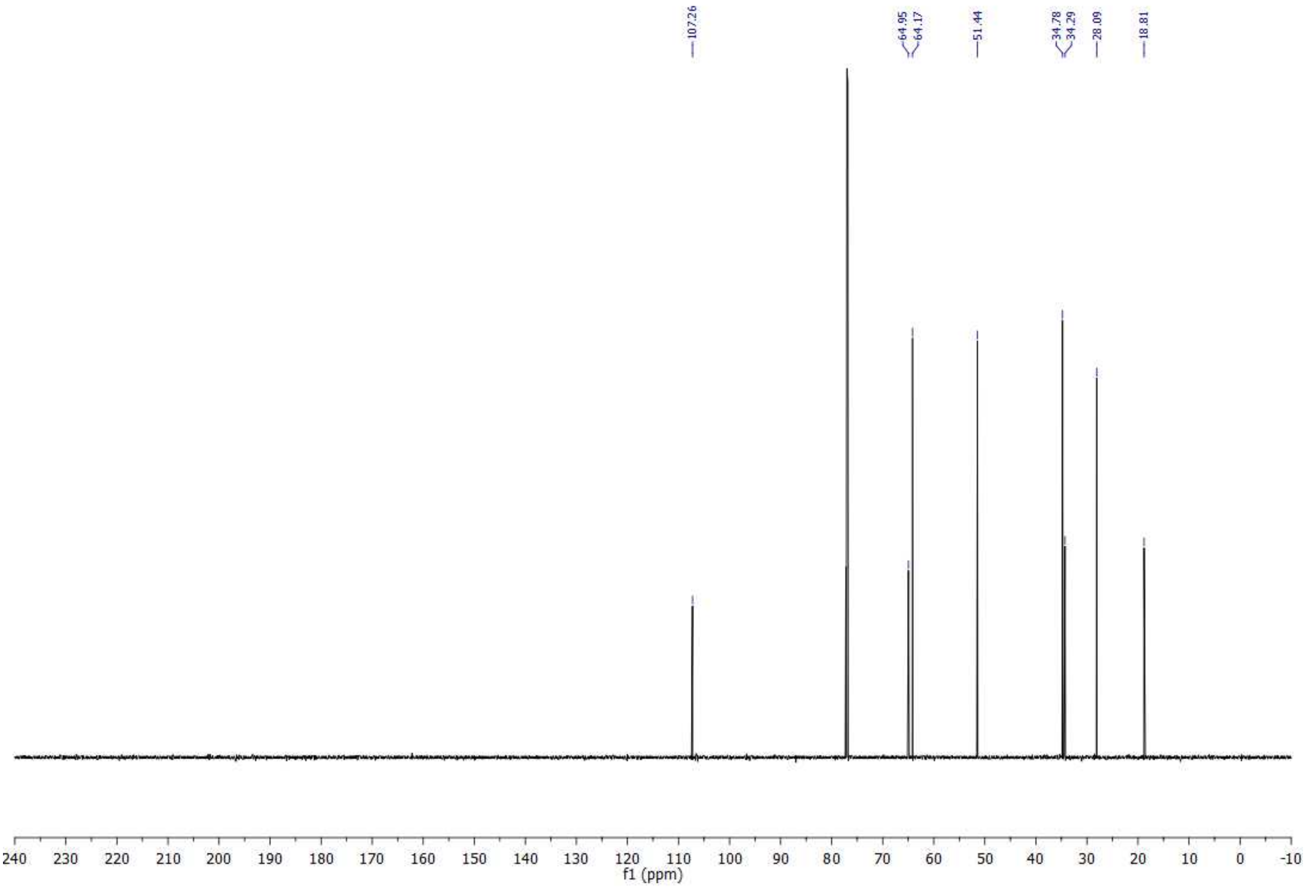

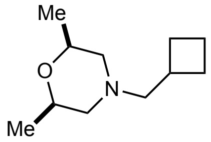
Me
O
N
Me

8h

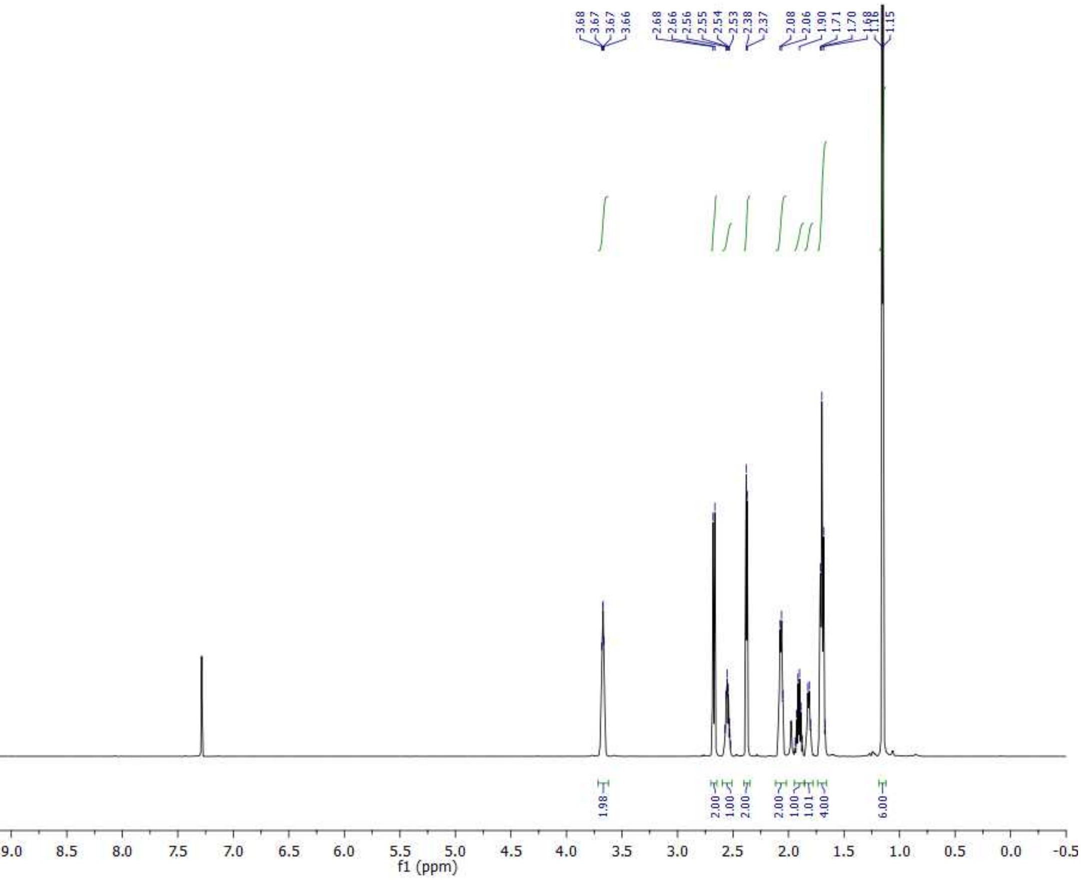
3.68
3.67
3.67
3.66
2.68
2.66
2.56
2.55
2.54
2.53
2.38
2.37
2.08
2.06
1.90
1.71
1.70
1.68
1.16
1.15
1.98
2.00
1.00
2.00
2.00
1.00
1.01
4.00
6.00
f1 (ppm)

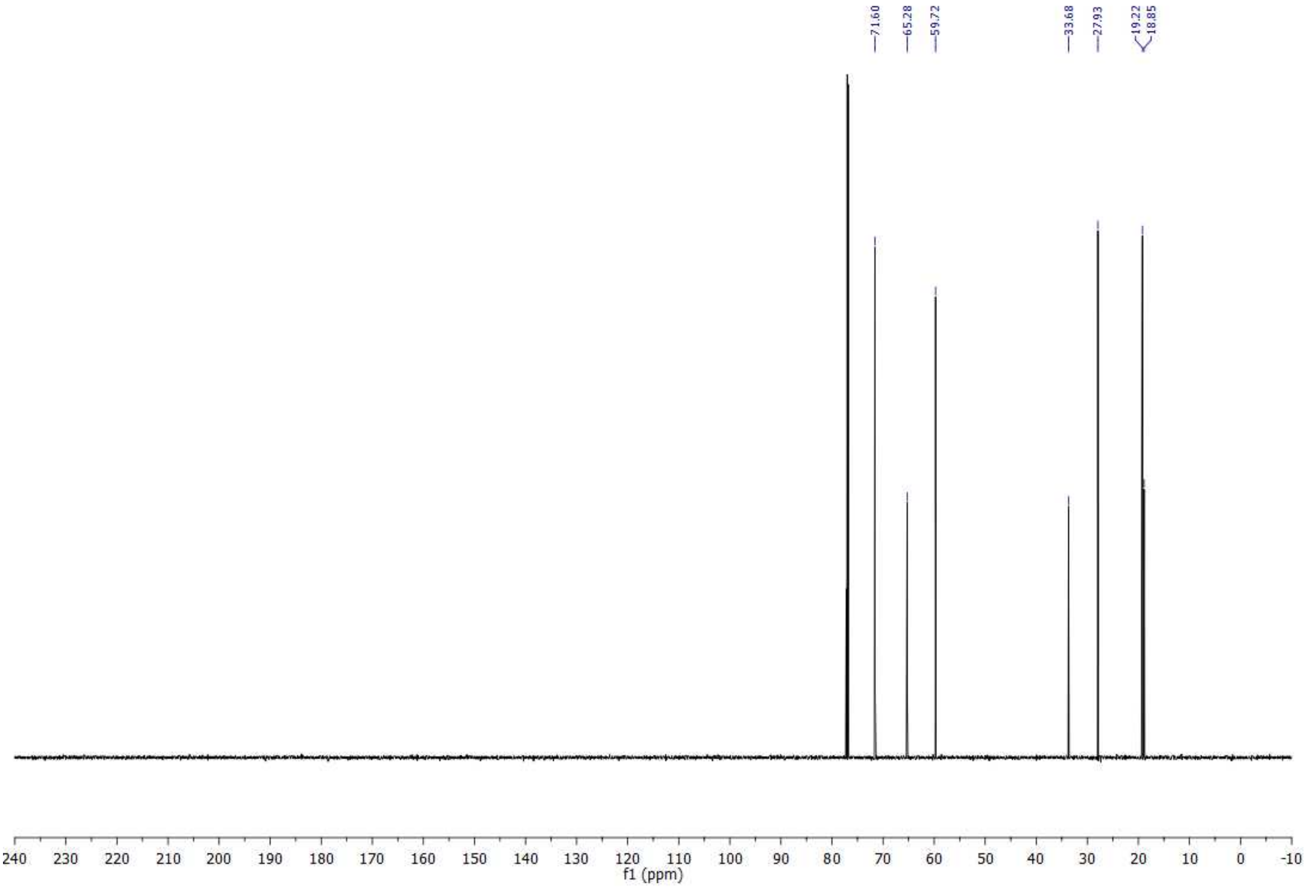
71.60
65.28
59.72
33.68
27.93
19.22
18.85
f1 (ppm)

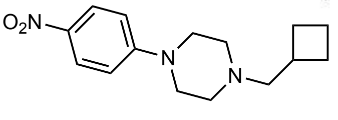
O2N
N
N

8i

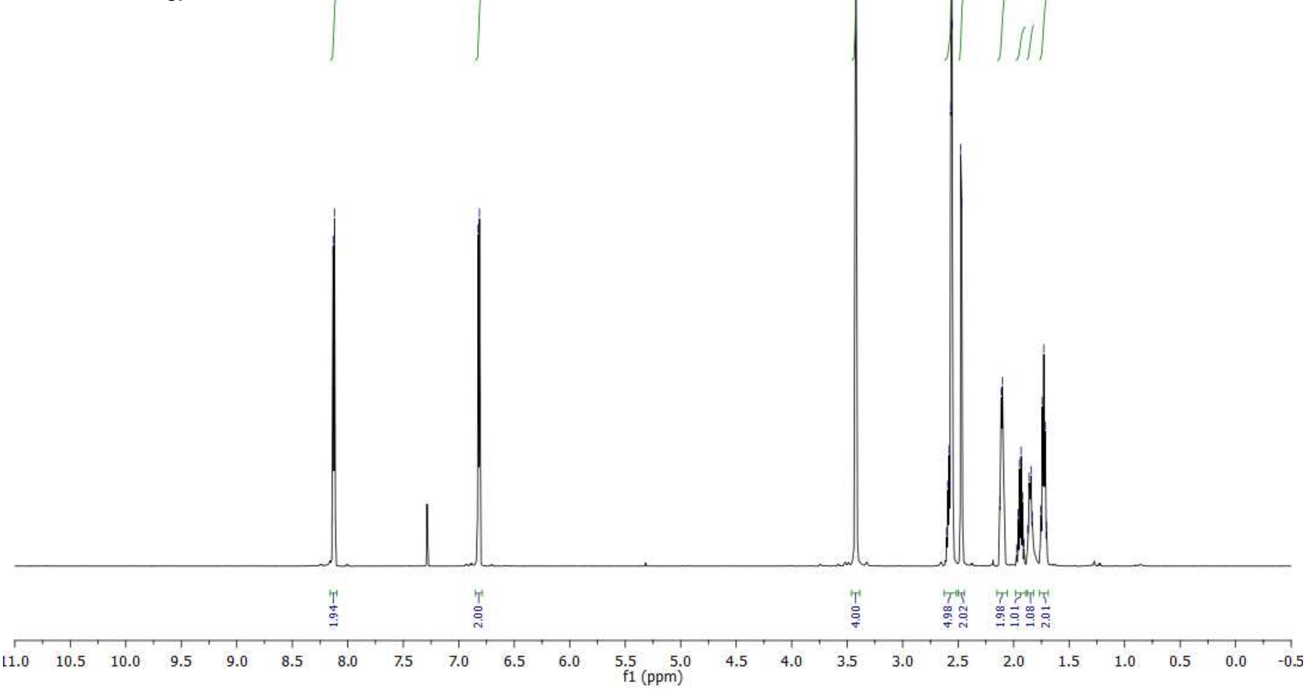
8.13
8.12
6.83
6.81
3.42
2.59
2.58
2.57
2.56
2.55
2.48
2.13
2.11
2.10
1.97
1.96
1.95
1.93
1.92
1.91
1.87
1.86
1.84
1.83
1.75
1.74
1.73
1.72
1.70
1.94
2.00
4.00
4.98
2.02
1.98
1.01
1.08
2.01
f1 (ppm)

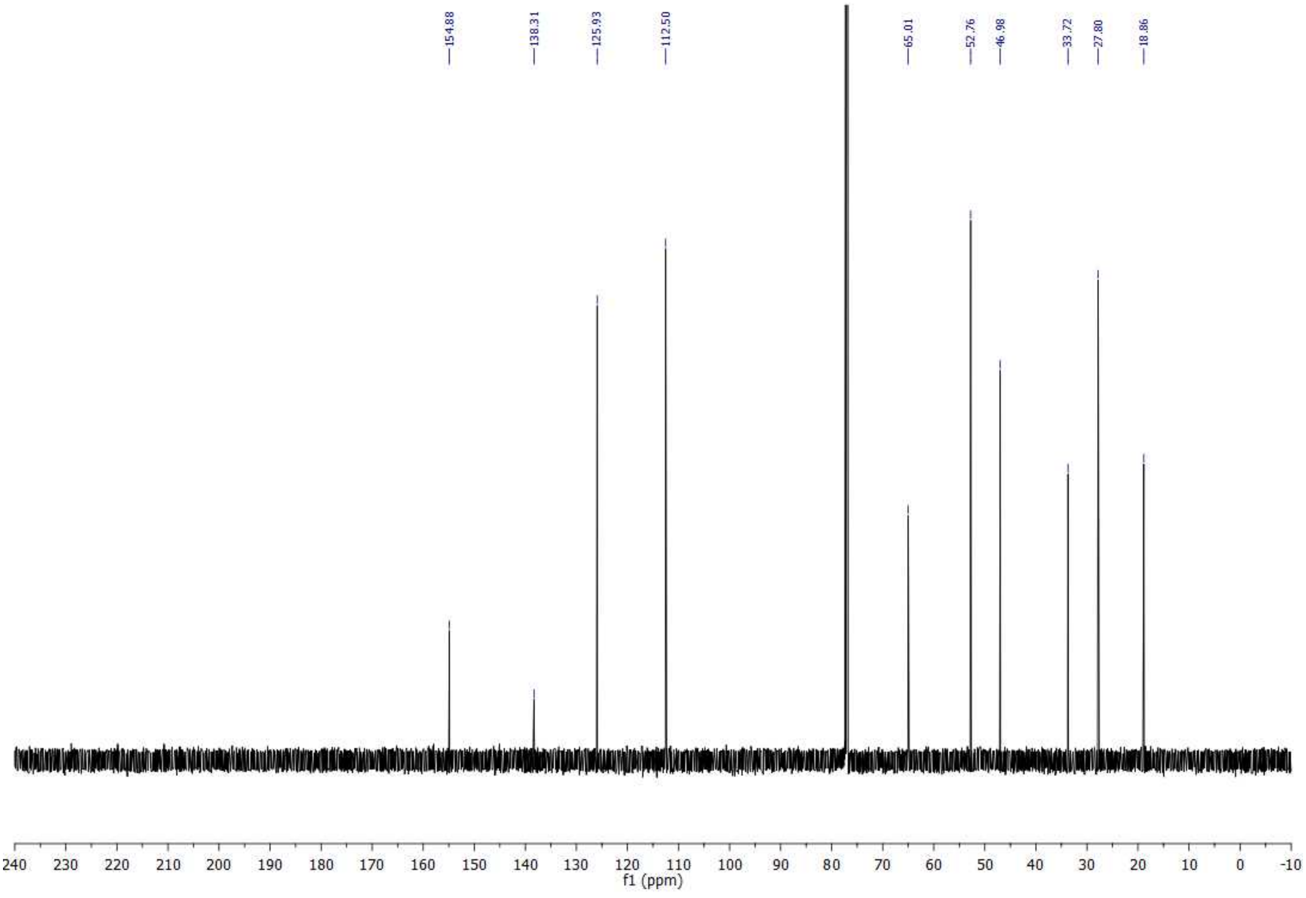
154.88
138.31
125.93
112.50
65.01
52.76
46.98
33.72
27.80
18.86
f1 (ppm)

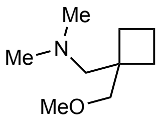
Me
Me
N
MeO

8j

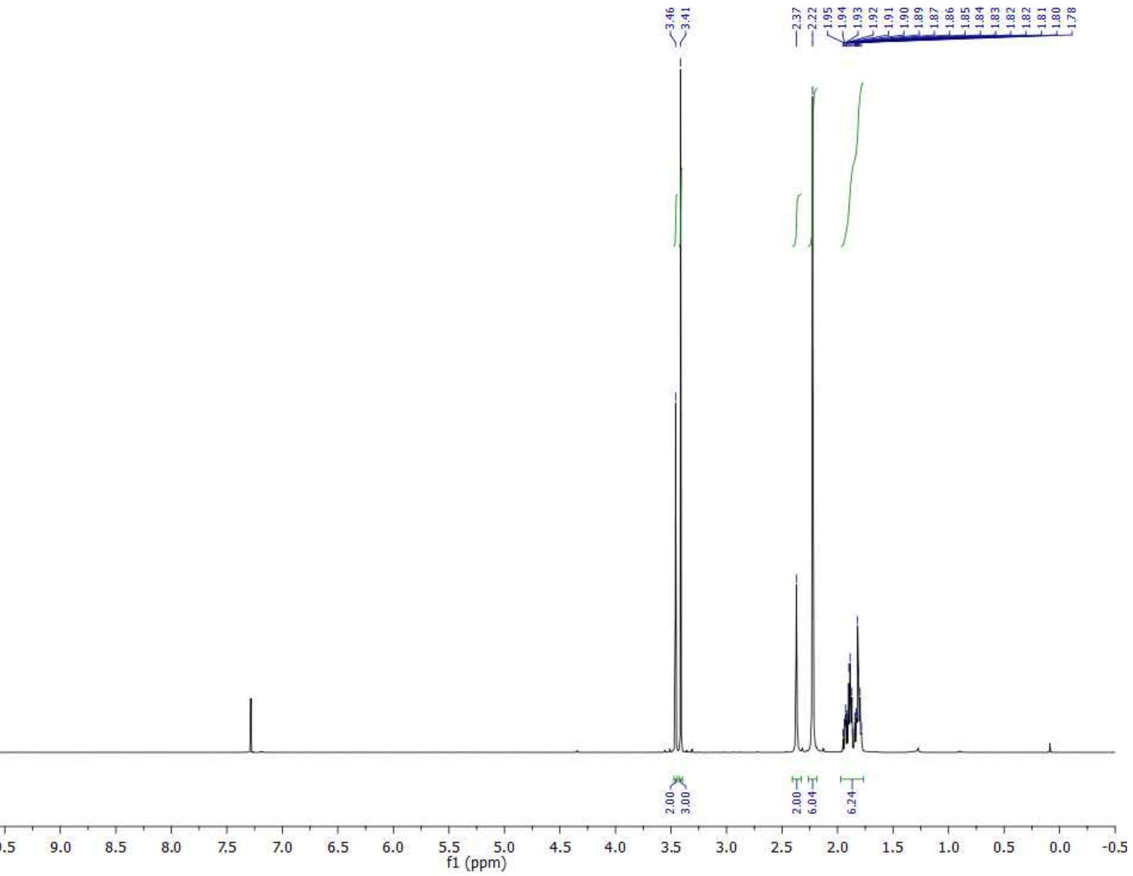
3.46
3.41
2.37
2.22
1.95
1.94
1.93
1.92
1.91
1.90
1.89
1.87
1.86
1.85
1.84
1.83
1.82
1.82
1.81
1.80
1.78
2.00
3.00
2.00
6.04
6.24
f1 (ppm)

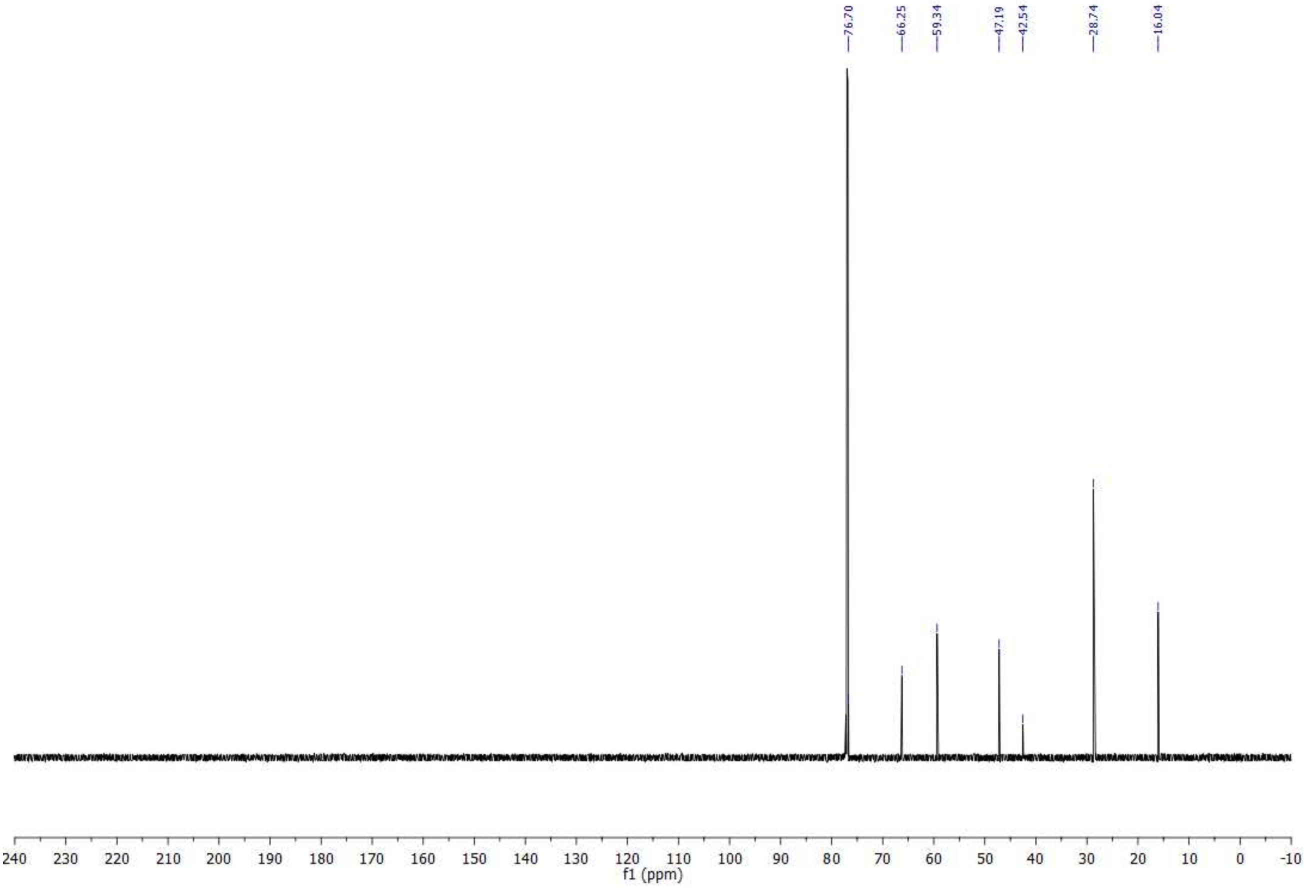
76.70
66.25
59.34
47.19
42.54
28.74
16.04
f1 (ppm)

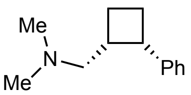

9a

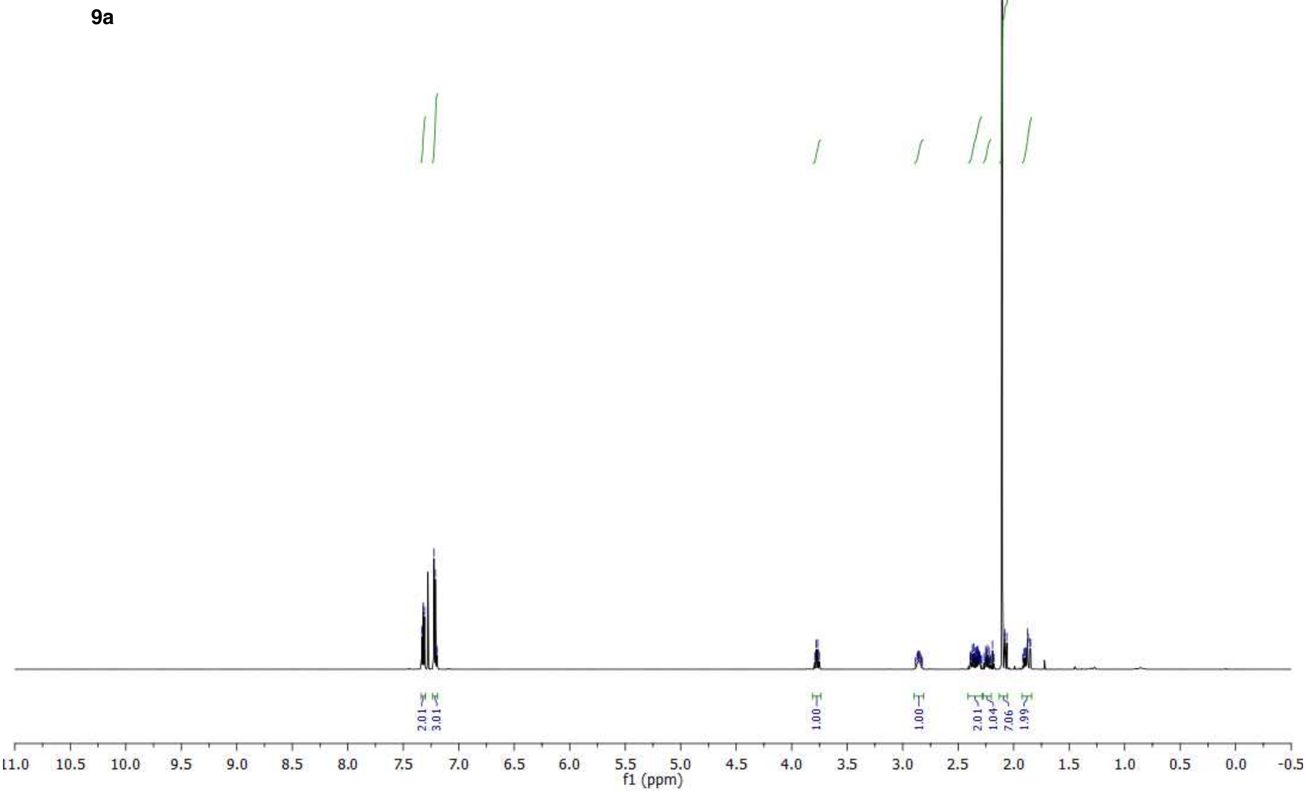

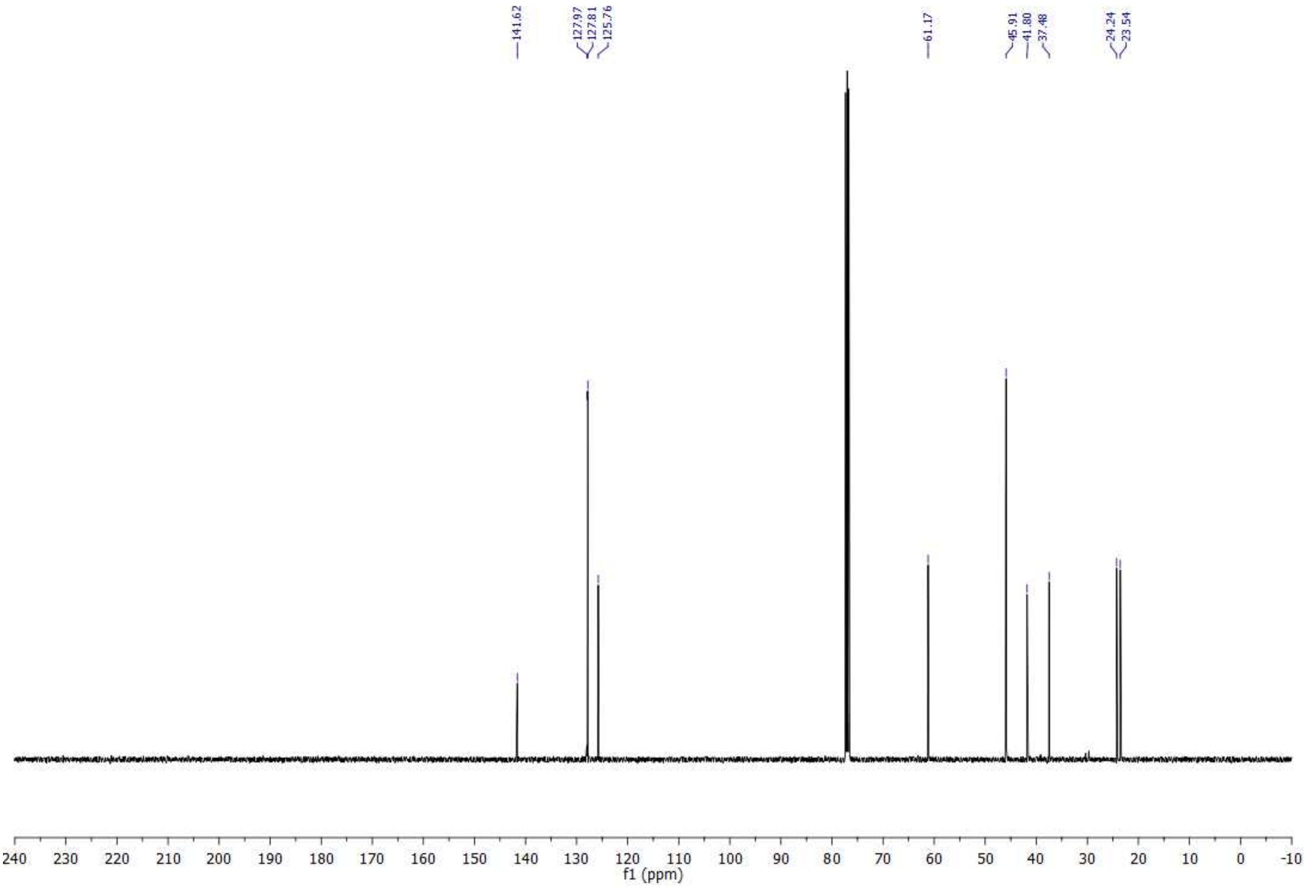

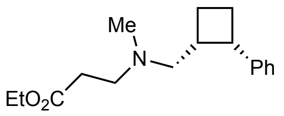

Me
N
Ph
EtO2C

9b

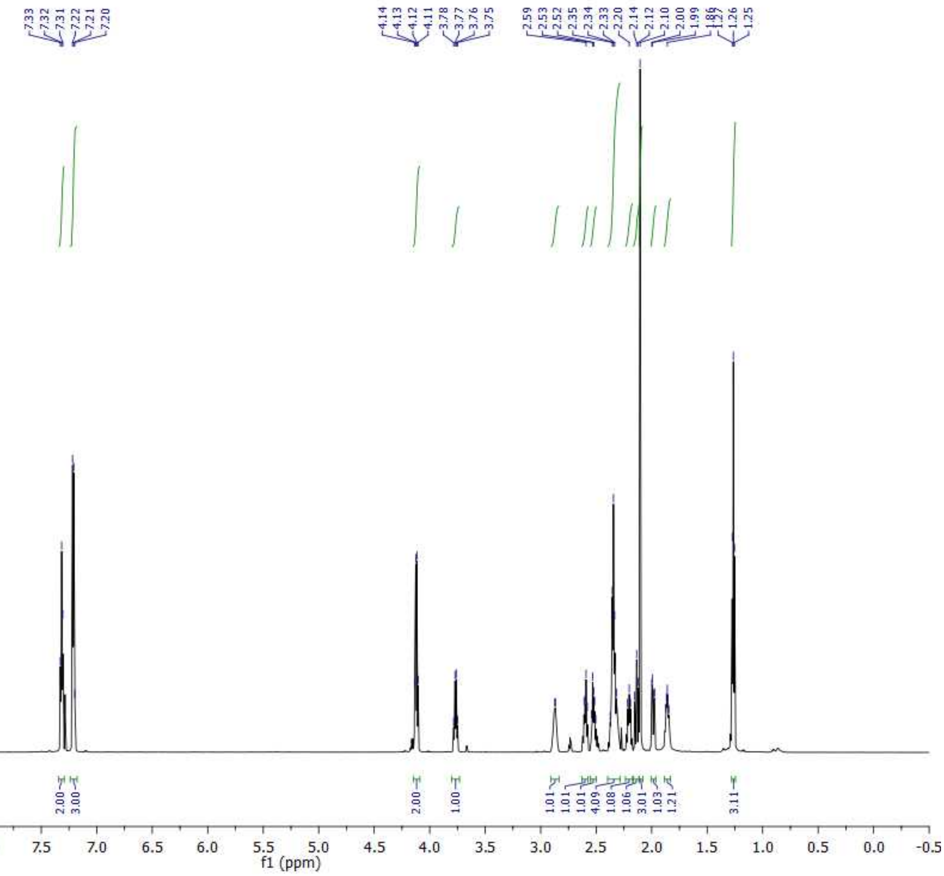

7.33 7.32 7.31 7.22 7.21 7.20
4.14 4.13 4.12 4.11 3.78 3.77 3.76 3.75
2.59 2.53 2.52 2.35 2.34 2.33 2.20 2.14 2.12 2.10 2.00 1.99 1.86 1.27 1.26 1.25
2.00 3.00 2.00 1.00 1.01 1.01 1.01 4.09 1.08 1.06 3.01 1.03 1.21 3.11
f1 (ppm)

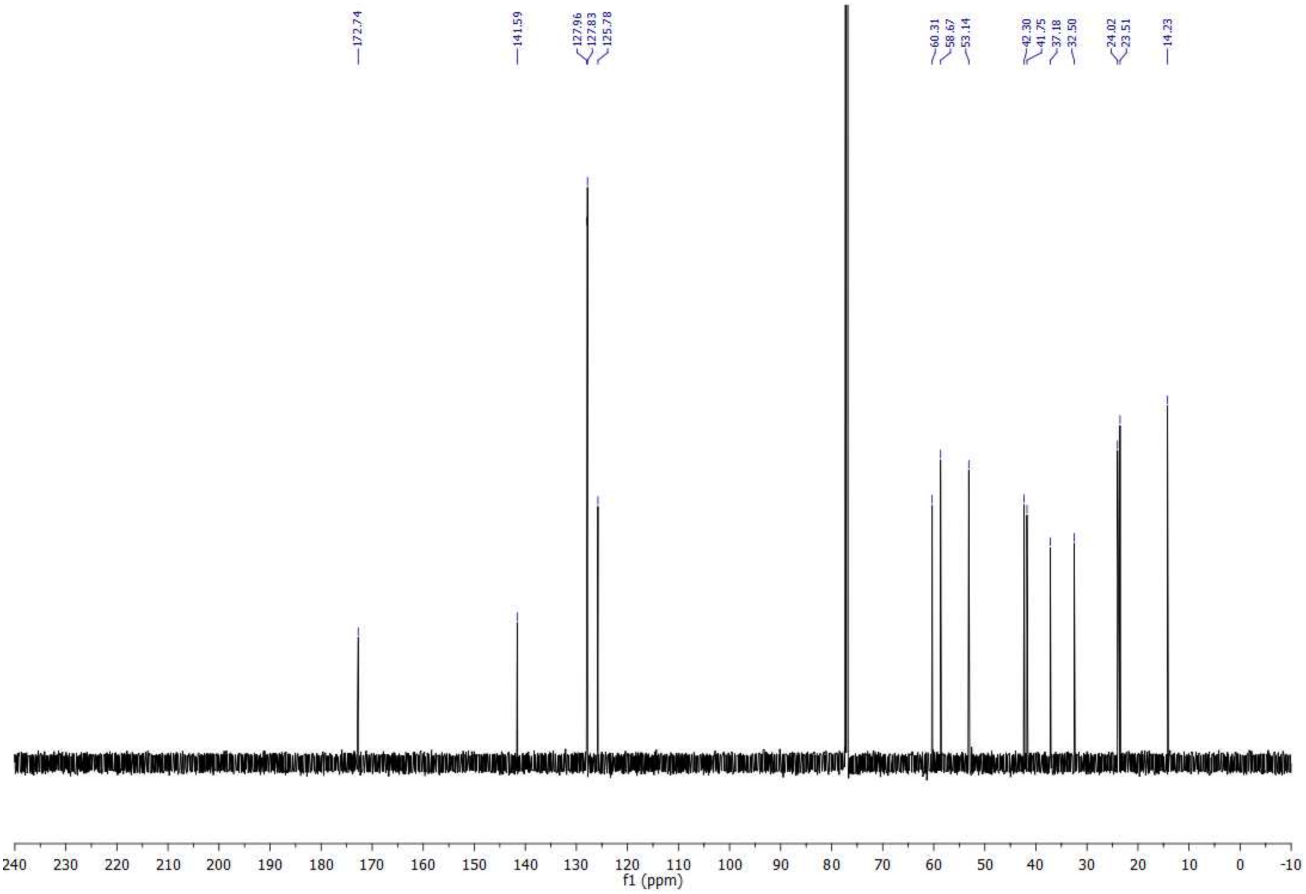

172.74
141.59
127.96 127.83 125.78
60.31 58.67 53.14
42.30 41.75 37.18 32.50
24.02 23.51
14.23
f1 (ppm)

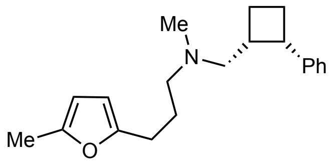
Me
N
Ph
Me
O

9c

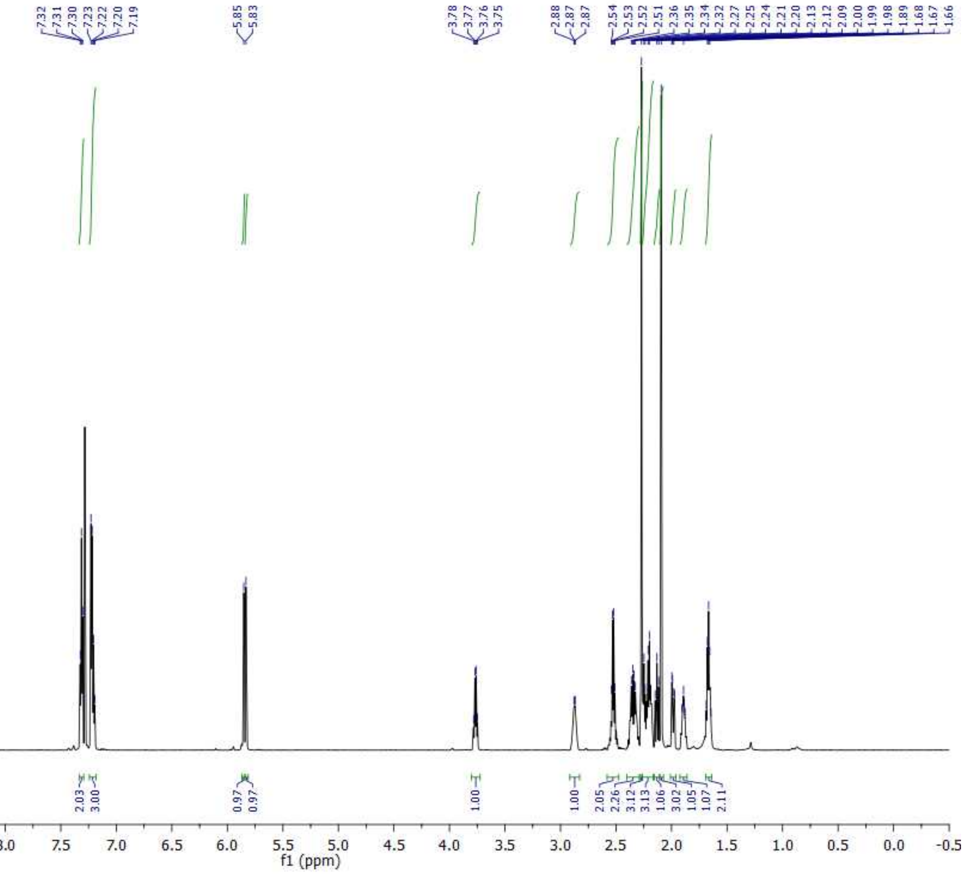
7.32
7.31
7.30
7.23
7.22
7.20
7.19
5.85
5.83
3.78
3.77
3.76
3.75
2.88
2.87
2.87
2.54
2.53
2.52
2.51
2.36
2.35
2.34
2.32
2.27
2.25
2.24
2.21
2.20
2.13
2.12
2.09
2.00
1.99
1.98
1.89
1.68
1.67
1.66
2.03
3.00
0.97
0.97
1.00
1.00
2.05
2.26
3.12
3.13
1.06
3.02
1.05
1.07
2.11
11.0 10.5 10.0 9.5 9.0 8.5 8.0 7.5 7.0 6.5 6.0 5.5 5.0 4.5 4.0 3.5 3.0 2.5 2.0 1.5 1.0 0.5 0.0 -0.5
f1 (ppm)

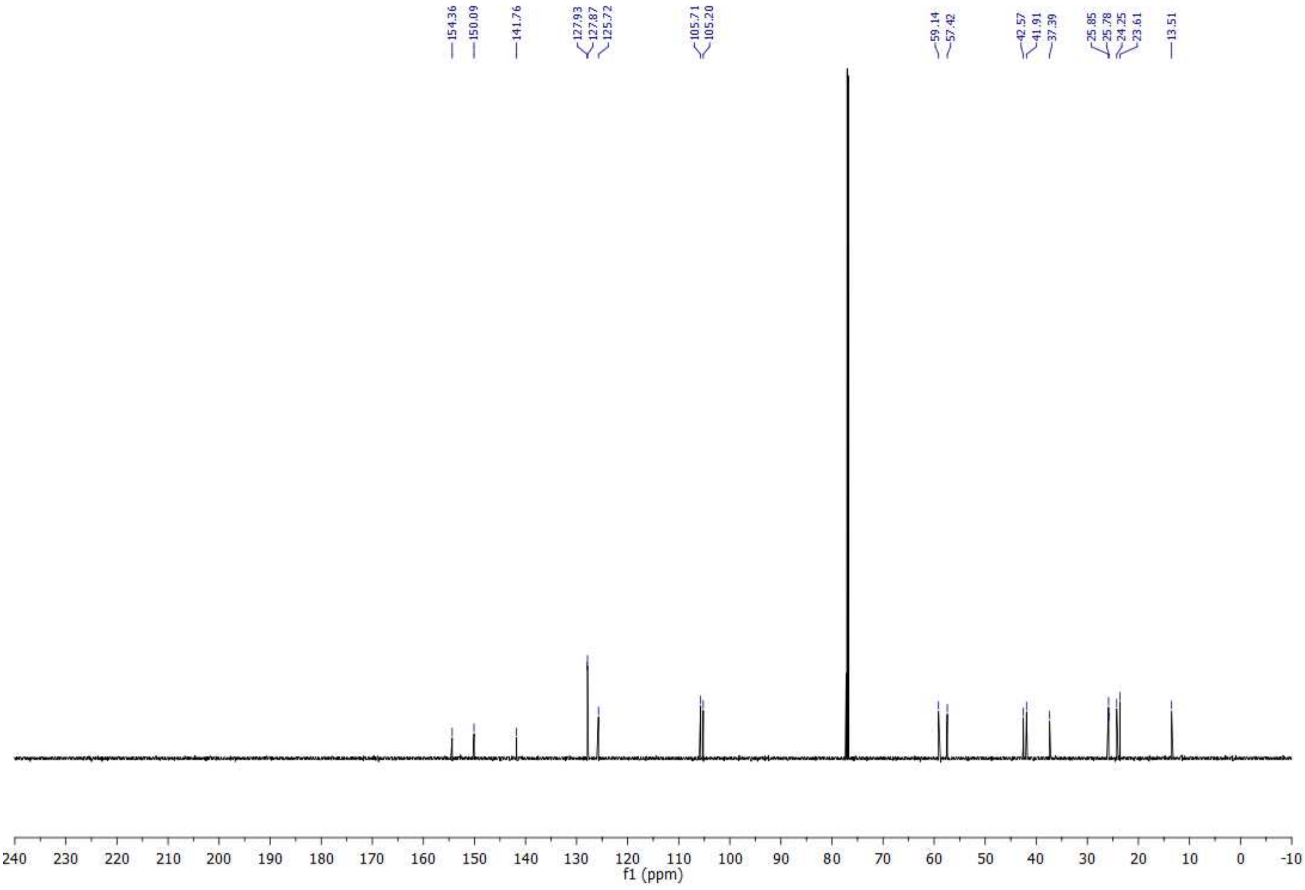
154.36
150.09
141.76
127.93
127.87
125.72
105.71
105.20
59.14
57.42
42.57
41.91
37.39
25.85
25.78
24.25
23.61
13.51
240 230 220 210 200 190 180 170 160 150 140 130 120 110 100 90 80 70 60 50 40 30 20 10 0 -10
f1 (ppm)

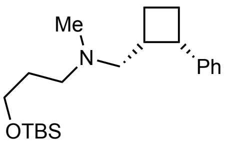

9d

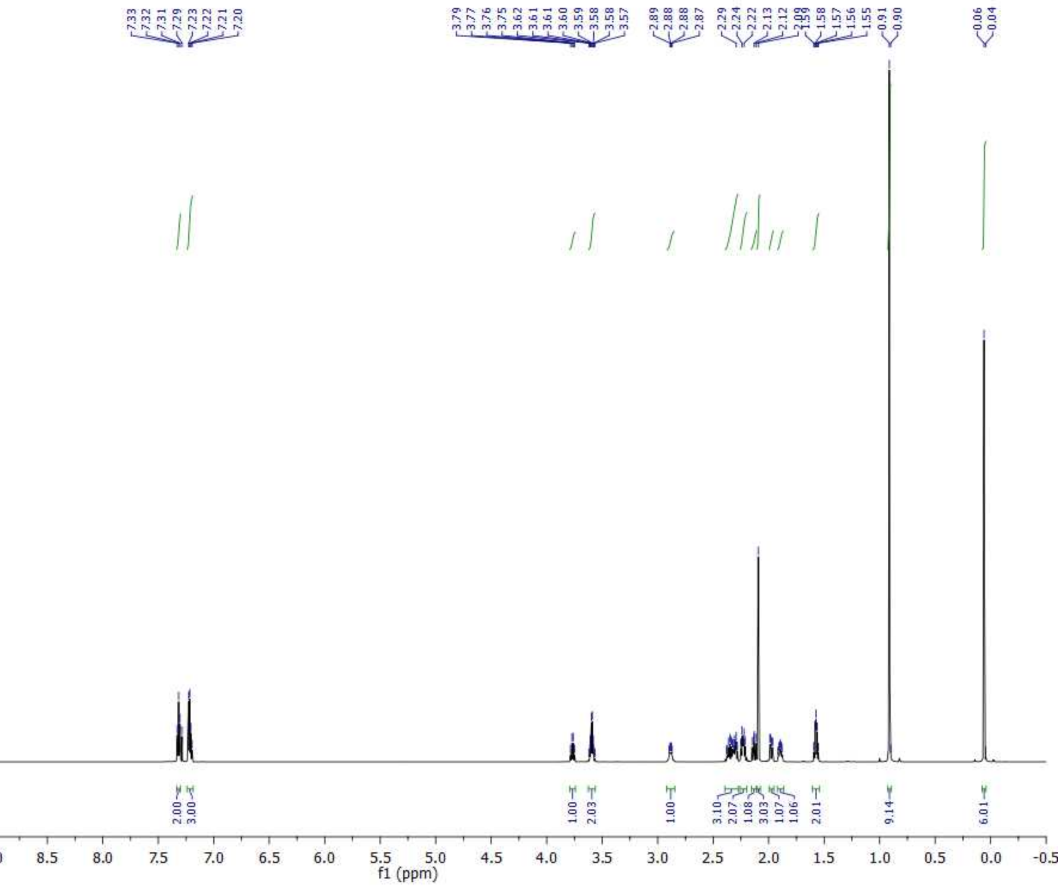
7.33
7.32
7.31
7.29
7.23
7.22
7.21
7.20
0.91
0.90
0.06
0.04
2.00
3.00
1.00
2.03
1.00
9.14
6.01
f1 (ppm)

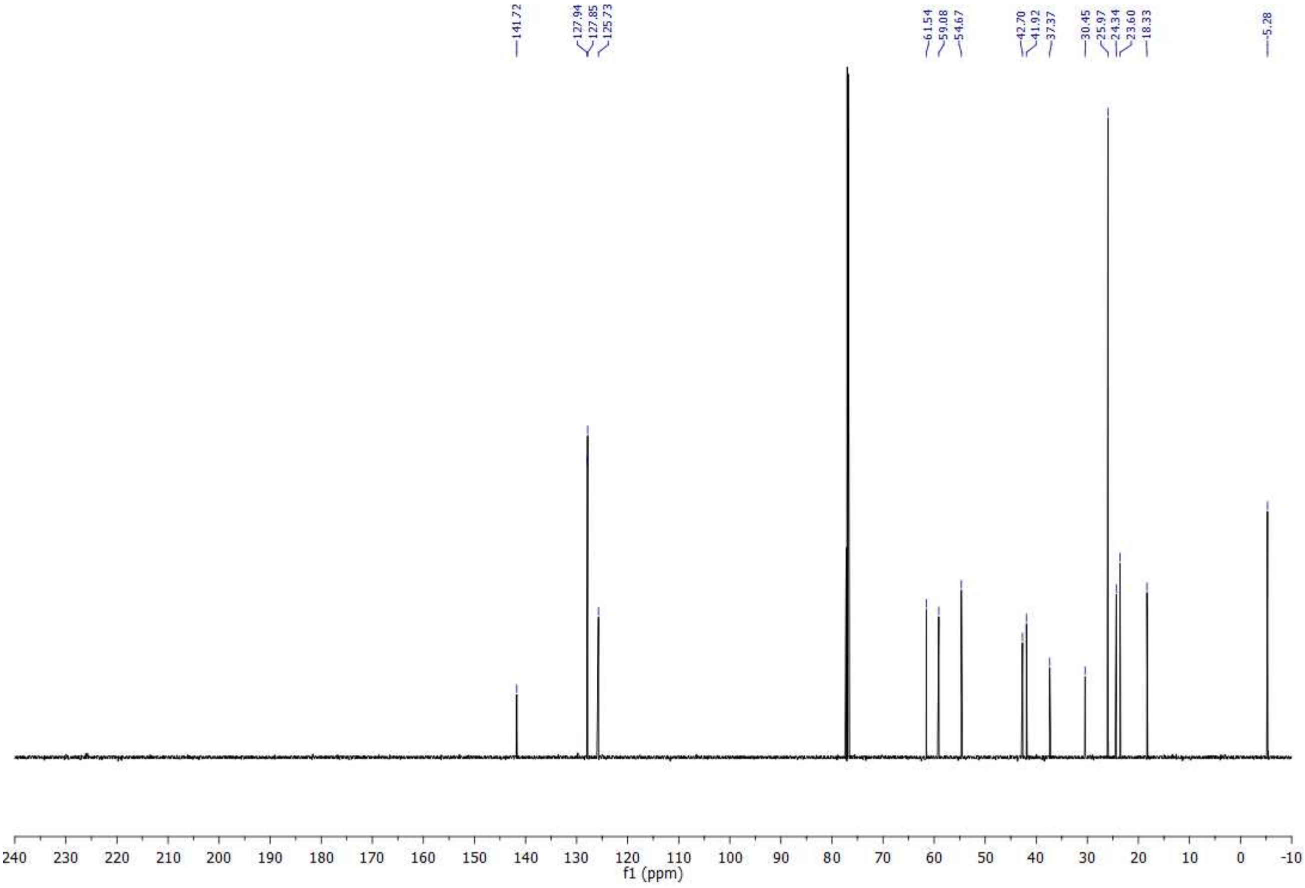
141.72
127.94
127.85
125.73
61.54
59.08
54.67
42.70
41.92
37.37
30.45
25.97
24.34
23.60
18.33
-5.28
f1 (ppm)

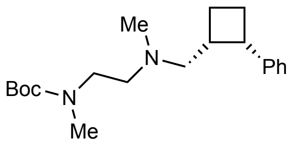
Me
N
Ph
Boc
N
Me

9e

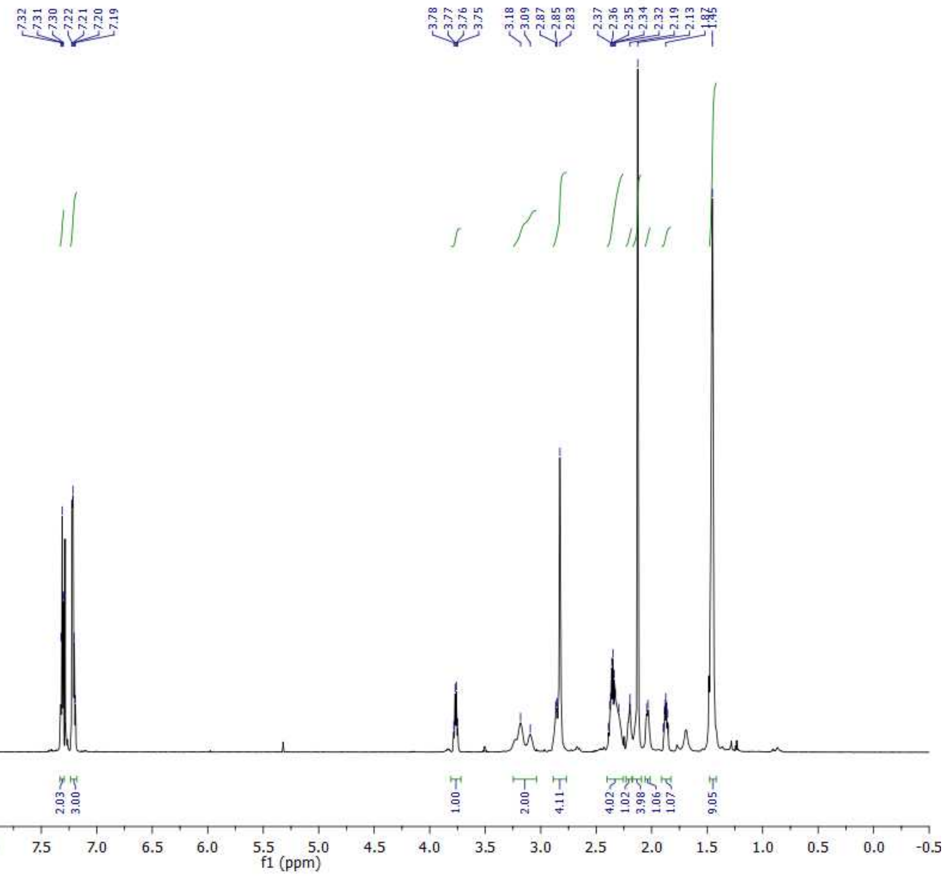
7.32
7.31
7.30
7.22
7.21
7.20
7.19
3.78
3.77
3.76
3.75
3.18
3.09
2.87
2.85
2.83
2.37
2.36
2.35
2.34
2.32
2.19
2.13
1.87
1.45
2.03
3.00
1.00
2.00
4.11
4.02
1.02
3.98
1.06
1.07
9.05
f1 (ppm)

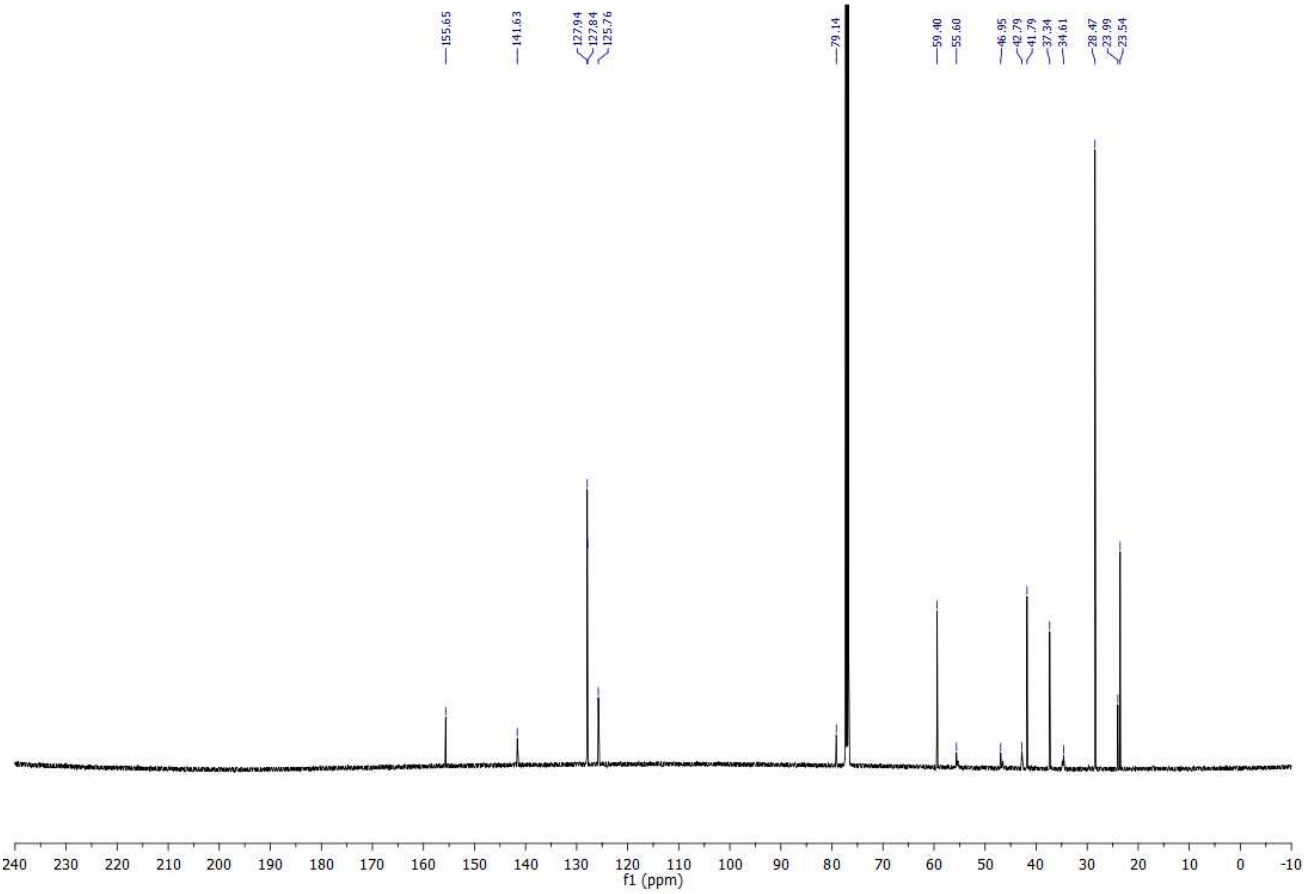
155.65
141.63
127.94
127.84
125.76
79.14
59.40
55.60
46.95
42.79
41.79
37.34
34.61
28.47
23.99
23.54
f1 (ppm)

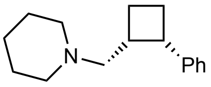

9f

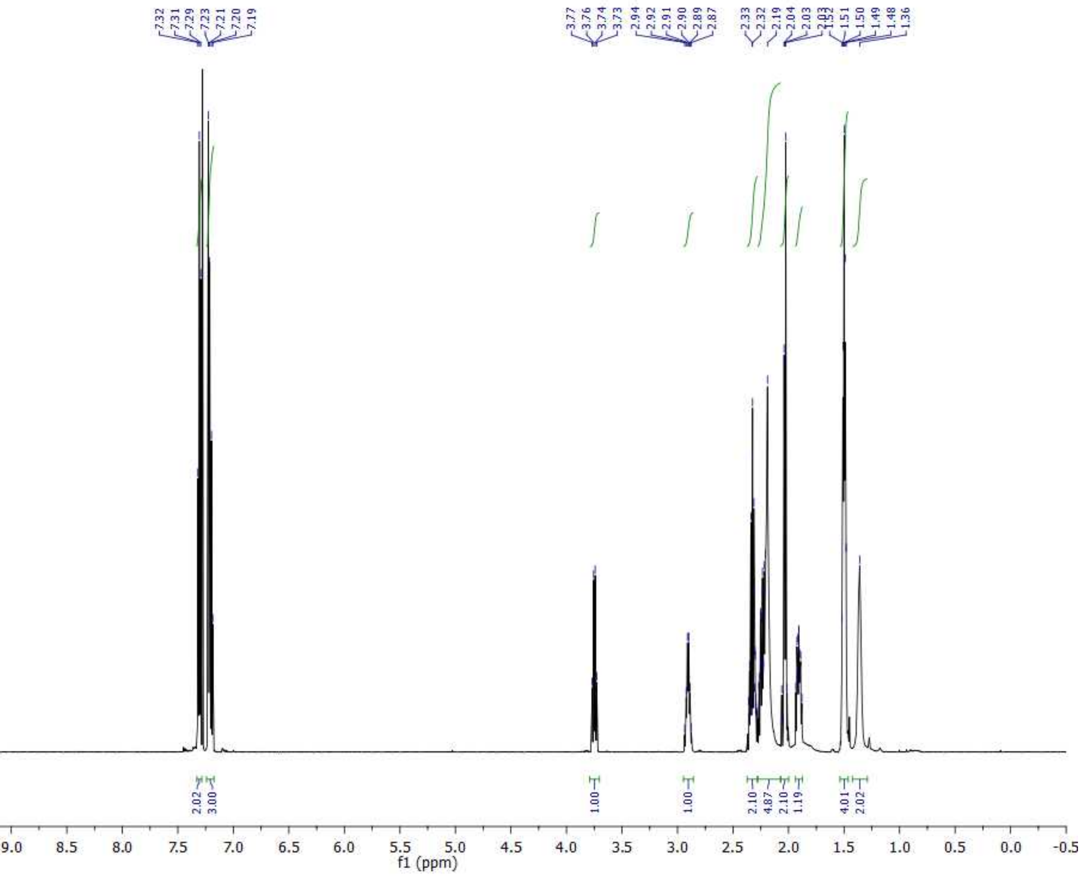

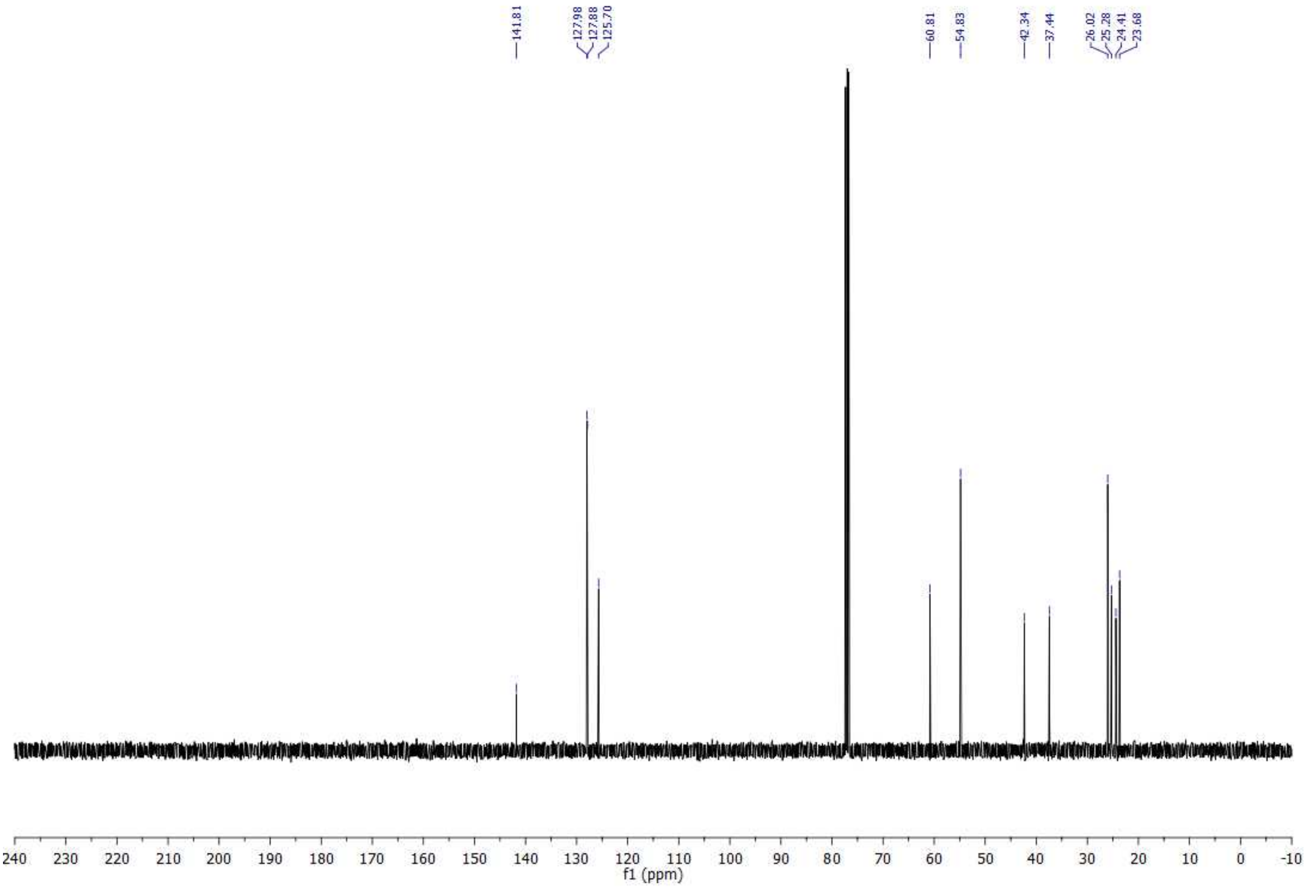

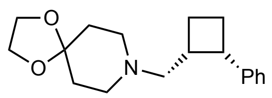
O
O
N
Ph

9g

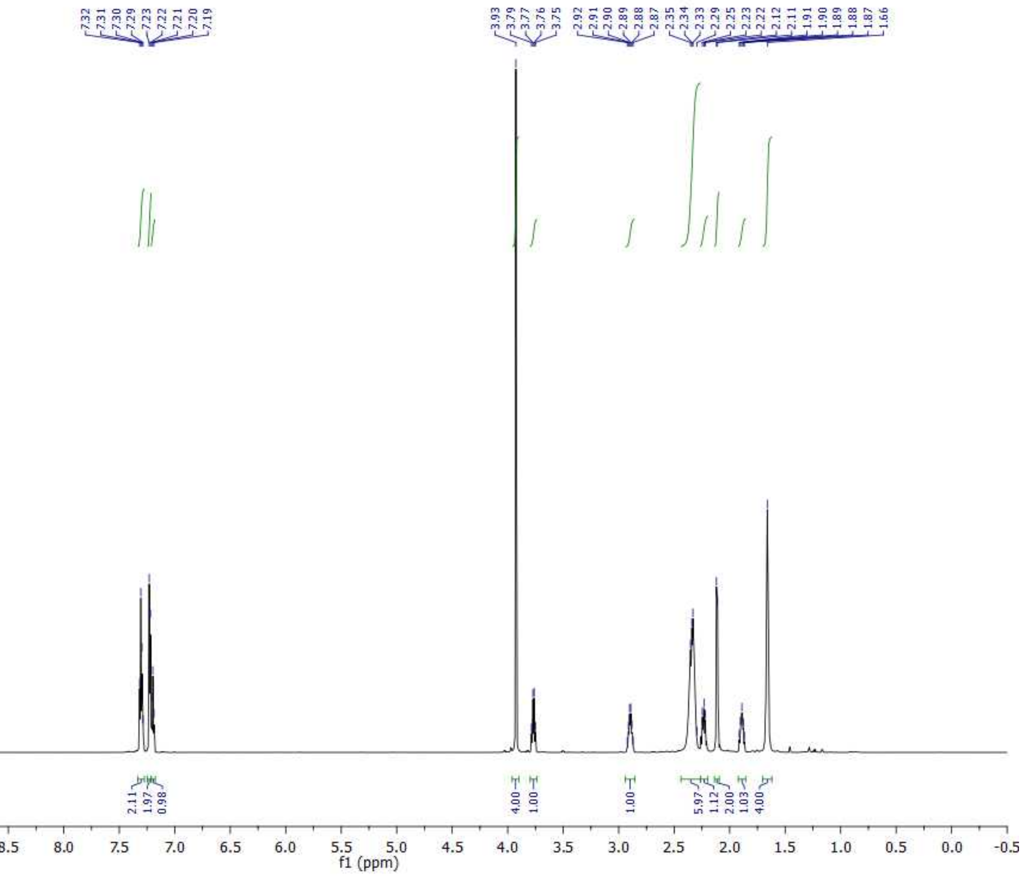
7.32 7.31 7.30 7.29 7.23 7.22 7.21 7.20 7.19
3.93 3.79 3.77 3.76 3.75
2.92 2.91 2.90 2.89 2.88 2.87 2.35 2.34 2.33 2.29 2.25 2.23 2.22 2.12 2.11 1.91 1.90 1.89 1.88 1.87 1.66
2.11 1.97 0.98
4.00 1.00
1.00
5.97 1.12 2.00 1.03 4.00
f1 (ppm)

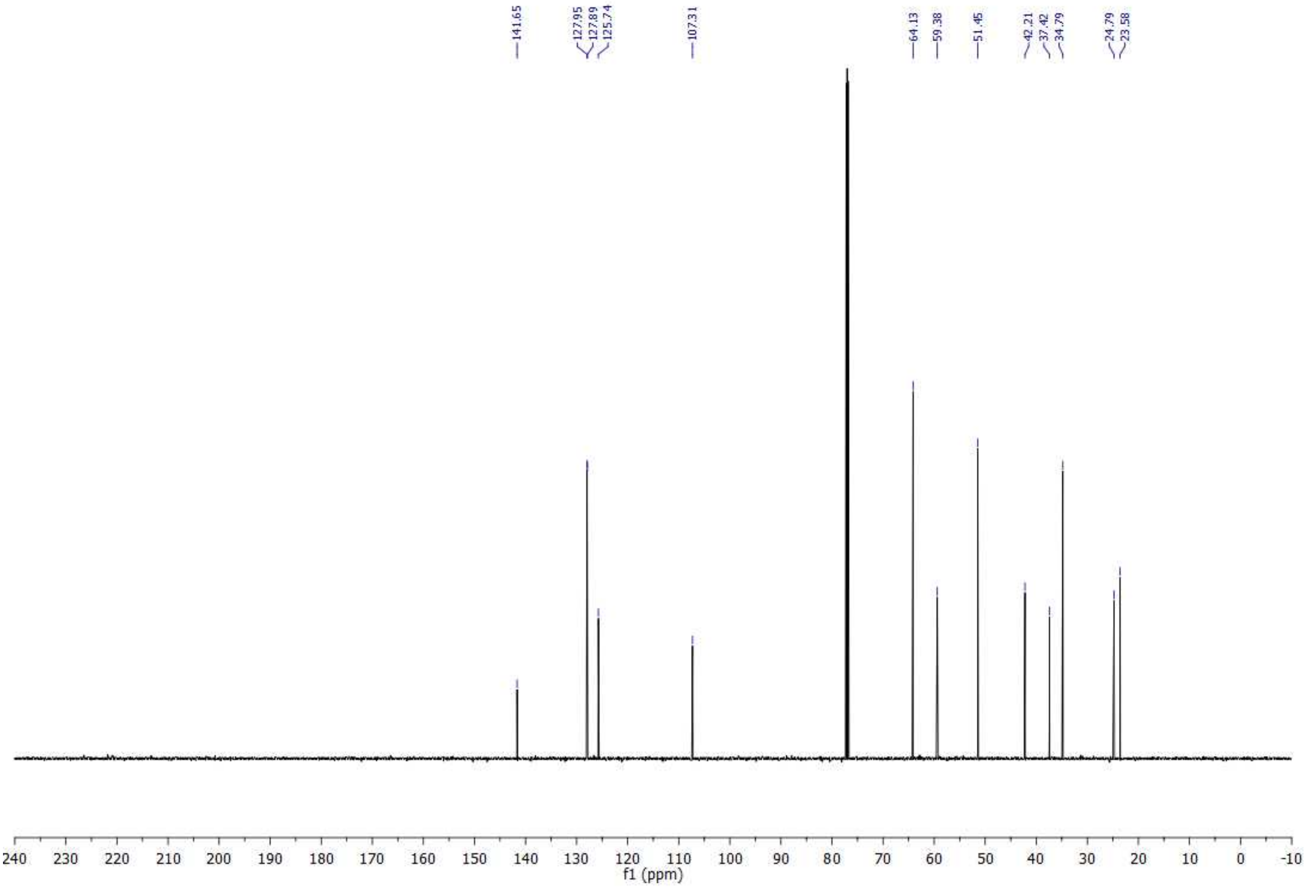
141.65
127.95 127.89 125.74
107.31
64.13
59.38
51.45
42.21 37.42 34.79
24.79 23.58
f1 (ppm)

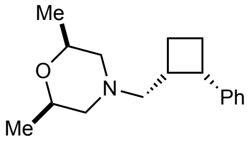
9h

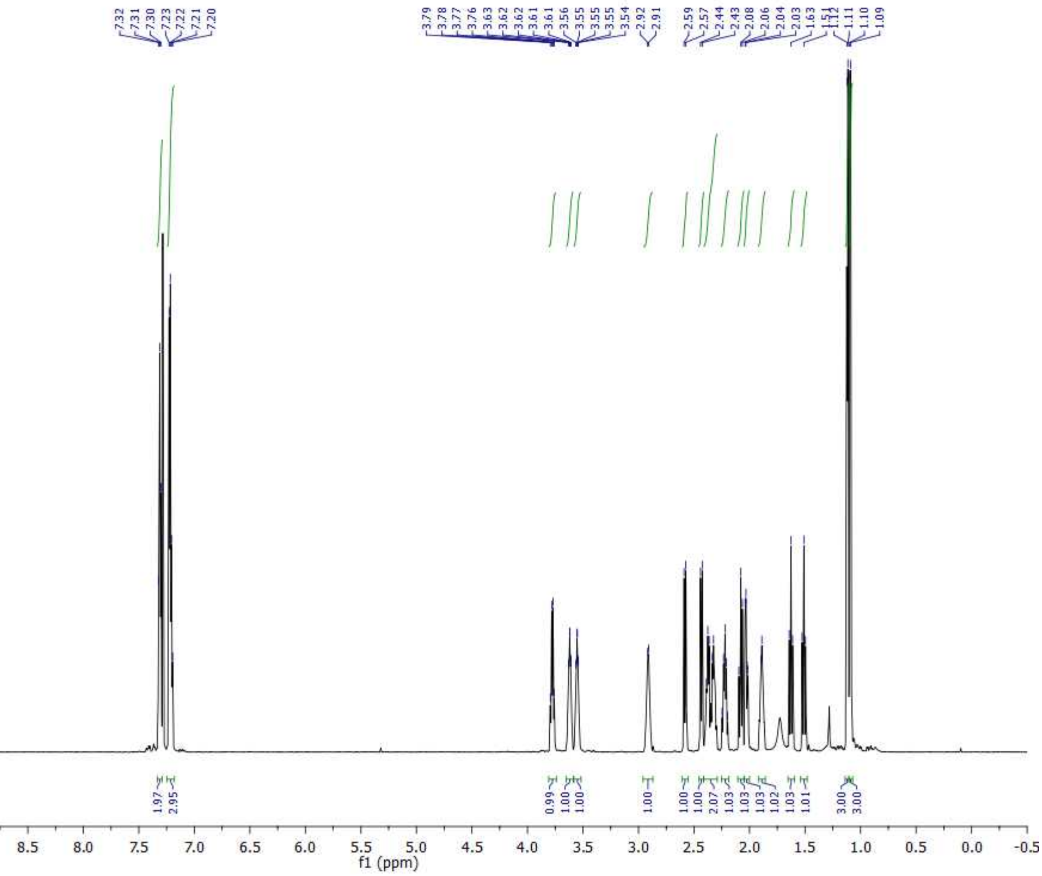

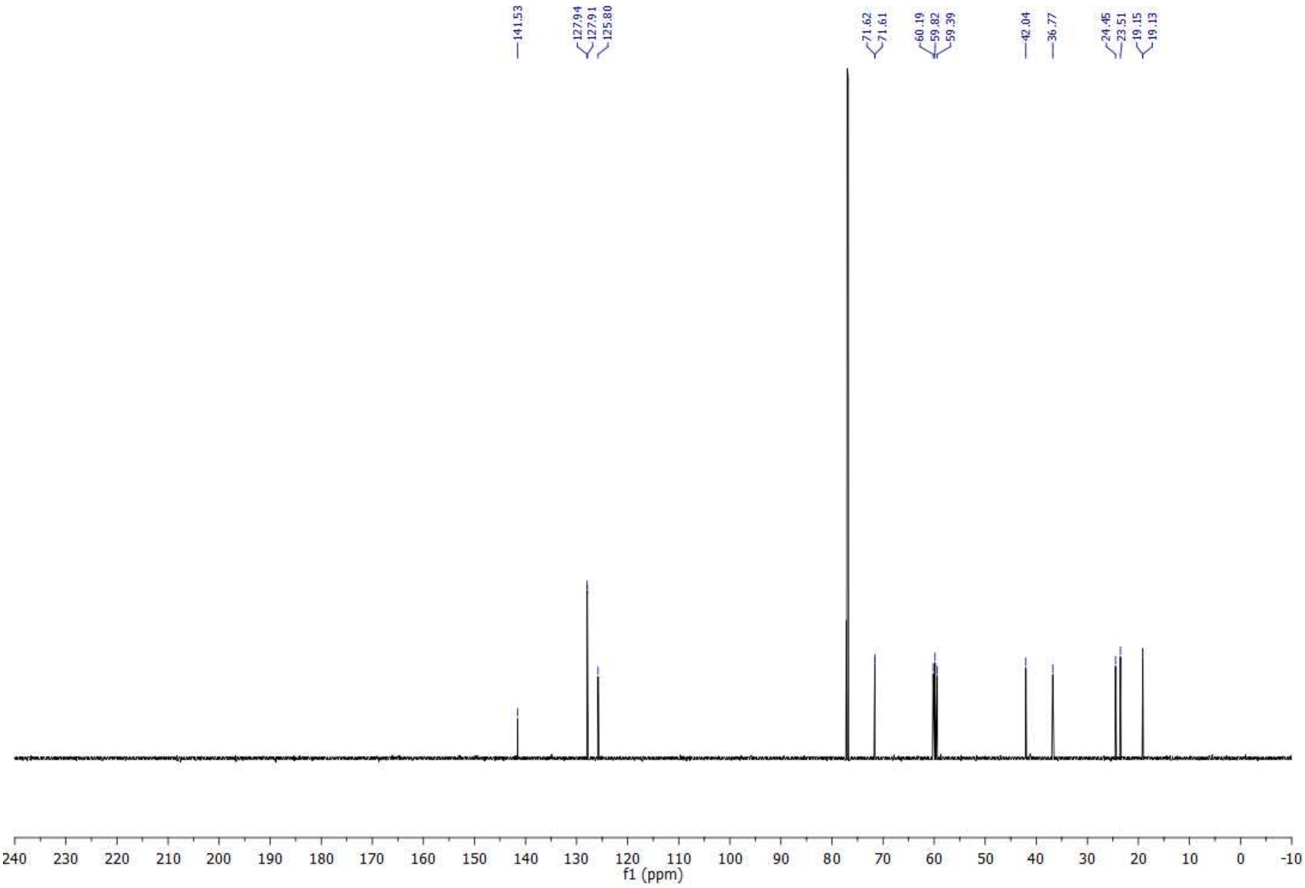

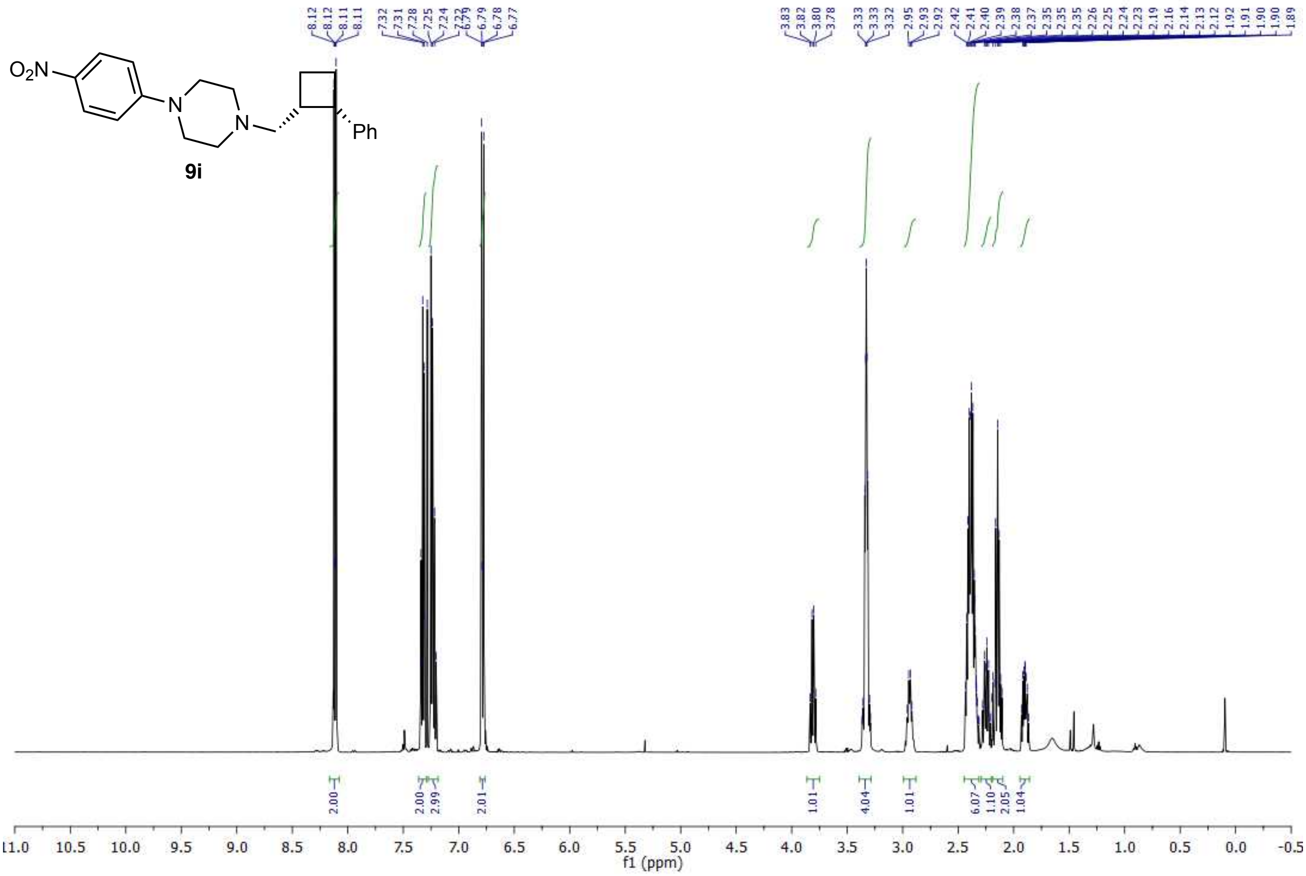
O_2N
Ph
9i
8.12 8.12 8.11 8.11 7.32 7.31 7.28 7.25 7.24 7.22 6.79 6.79 6.78 6.77
3.83 3.82 3.80 3.78 3.33 3.33 3.32 2.95 2.93 2.92 2.42 2.41 2.40 2.39 2.38 2.37 2.35 2.35 2.35 2.26 2.25 2.24 2.23 2.19 2.16 2.14 2.13 2.12 1.92 1.91 1.90 1.89
2.00 2.00 2.99 2.01 1.01 4.04 1.01 6.07 1.10 2.05 1.04
f1 (ppm)

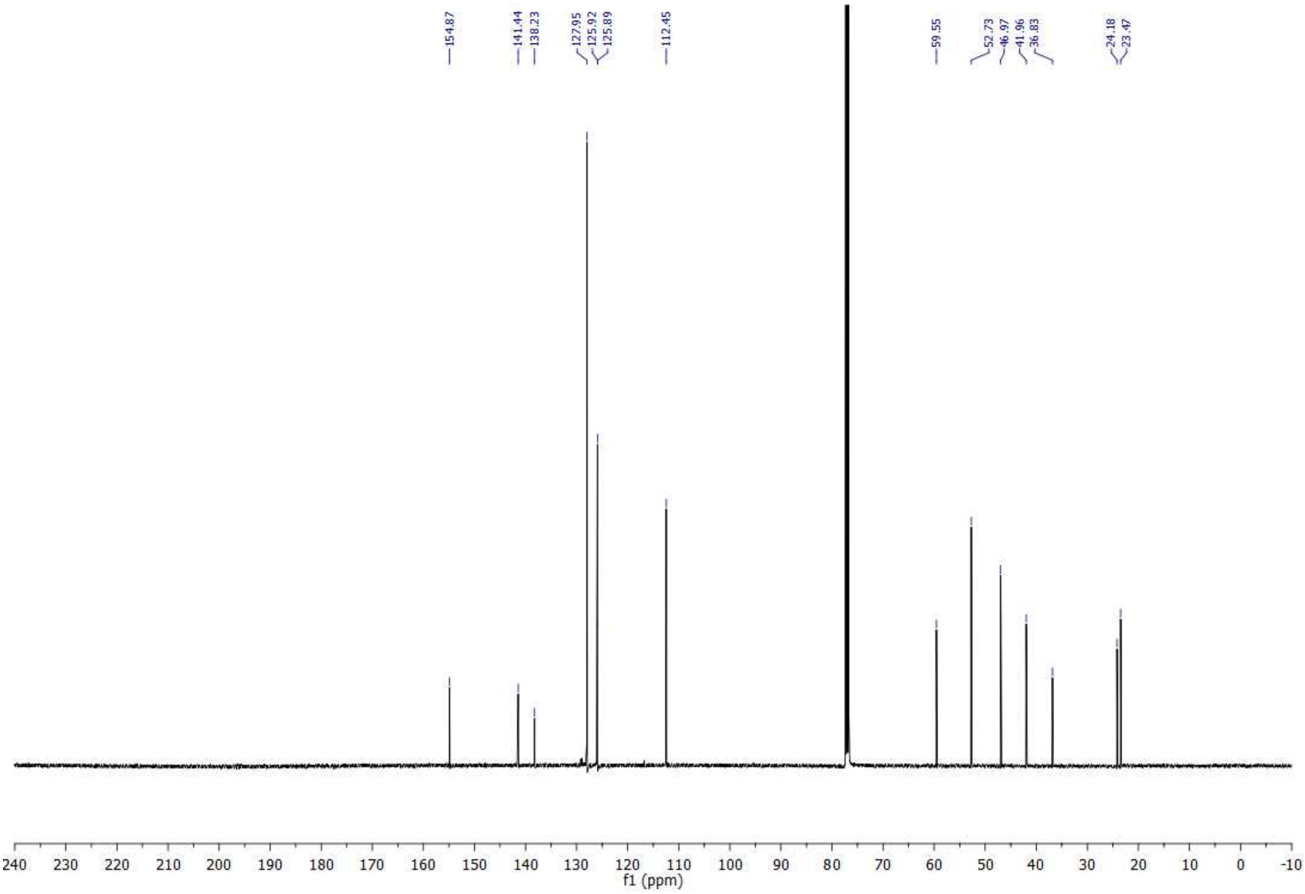
154.87
141.44 138.23
127.95 125.92 125.89
112.45
59.55
52.73 46.97 41.96 36.83
24.18 23.47
f1 (ppm)

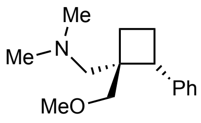

9j

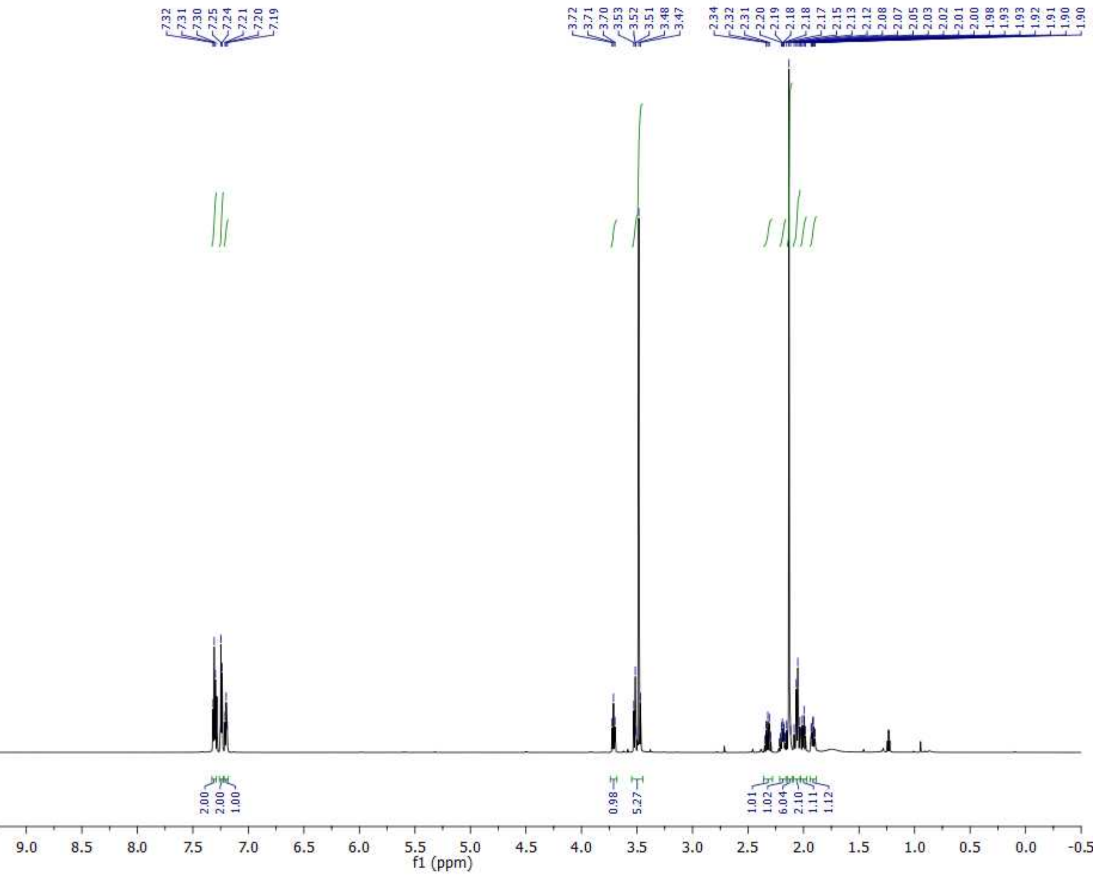

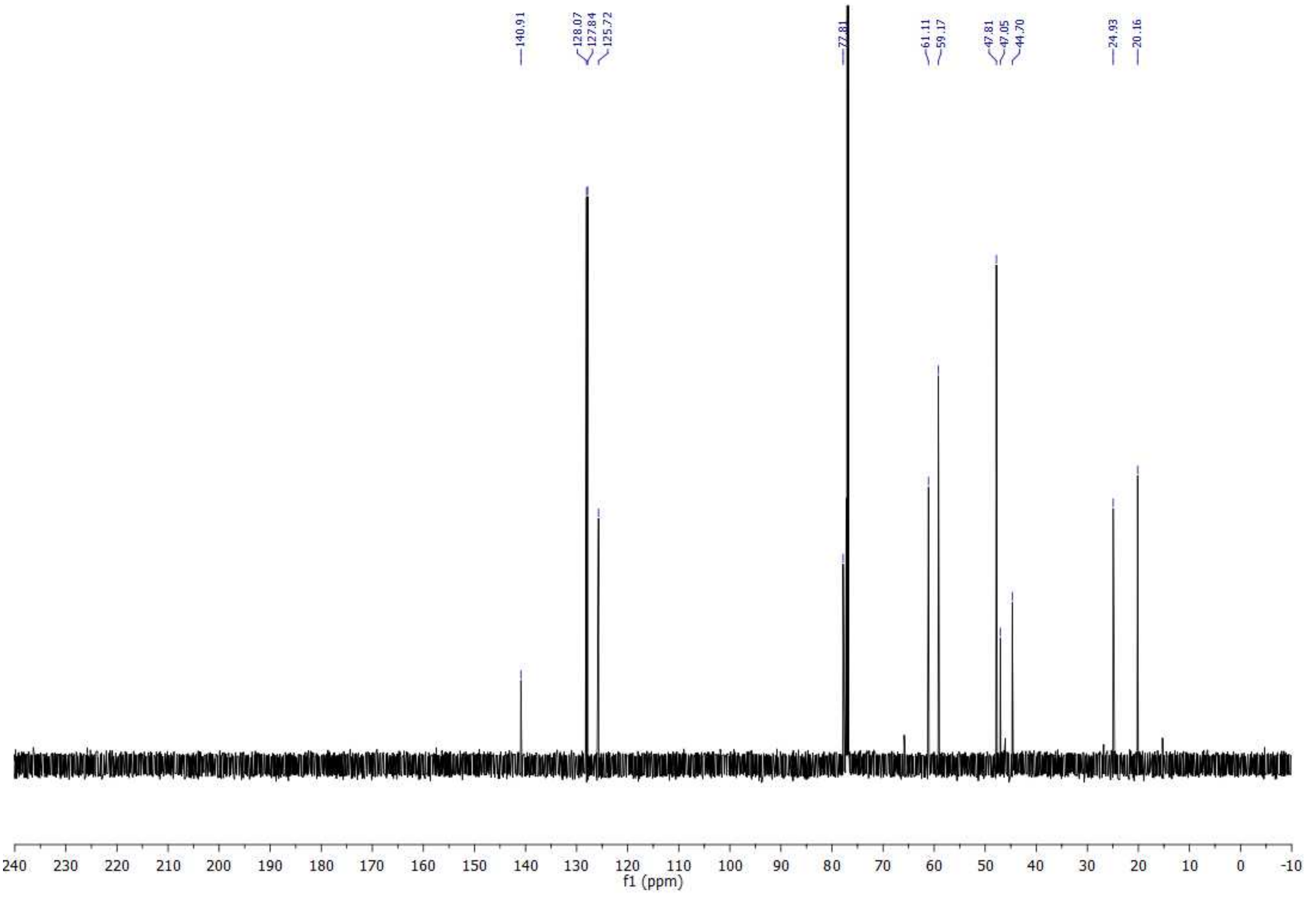

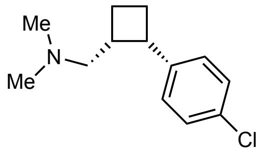
Me
N
Me
Cl

9aa

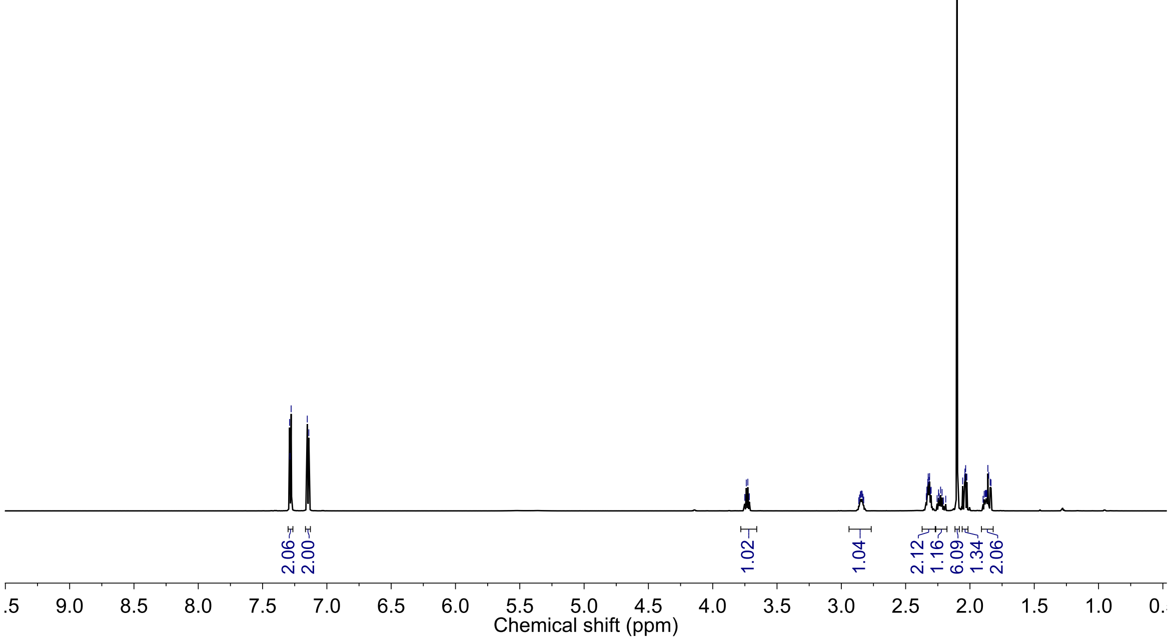
7.29
7.29
7.28
7.15
7.14
3.75
3.74
3.73
3.72
2.86
2.86
2.85
2.85
2.84
2.83
2.83
2.34
2.33
2.33
2.32
2.32
2.31
2.31
2.30
2.26
2.24
2.24
2.23
2.22
2.22
2.21
2.19
2.10
2.06
2.04
2.04
2.03
2.02
1.90
1.89
1.88
1.88
1.87
1.87
1.86
1.85
1.84
1.84
2.06
2.00
1.02
1.04
2.12
1.16
6.09
1.34
2.06
.5
9.0
8.5
8.0
7.5
7.0
6.5
6.0
5.5
5.0
4.5
4.0
3.5
3.0
2.5
2.0
1.5
1.0
0.
Chemical shift (ppm)

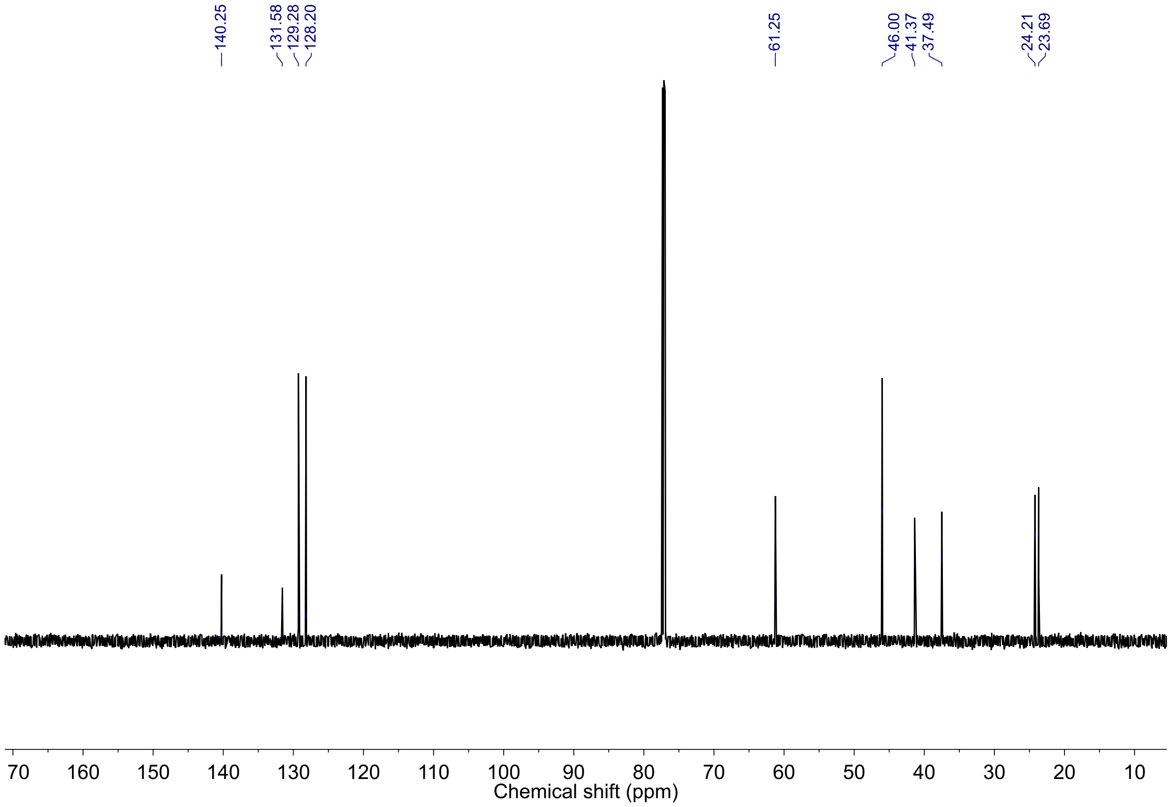
140.25
131.58
129.28
128.20
61.25
46.00
41.37
37.49
24.21
23.69
70
160
150
140
130
120
110
100
90
80
70
60
50
40
30
20
10
Chemical shift (ppm)

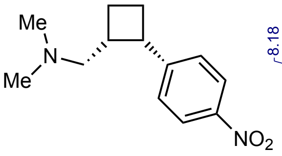
Me
N
Me
NO_2

9ab

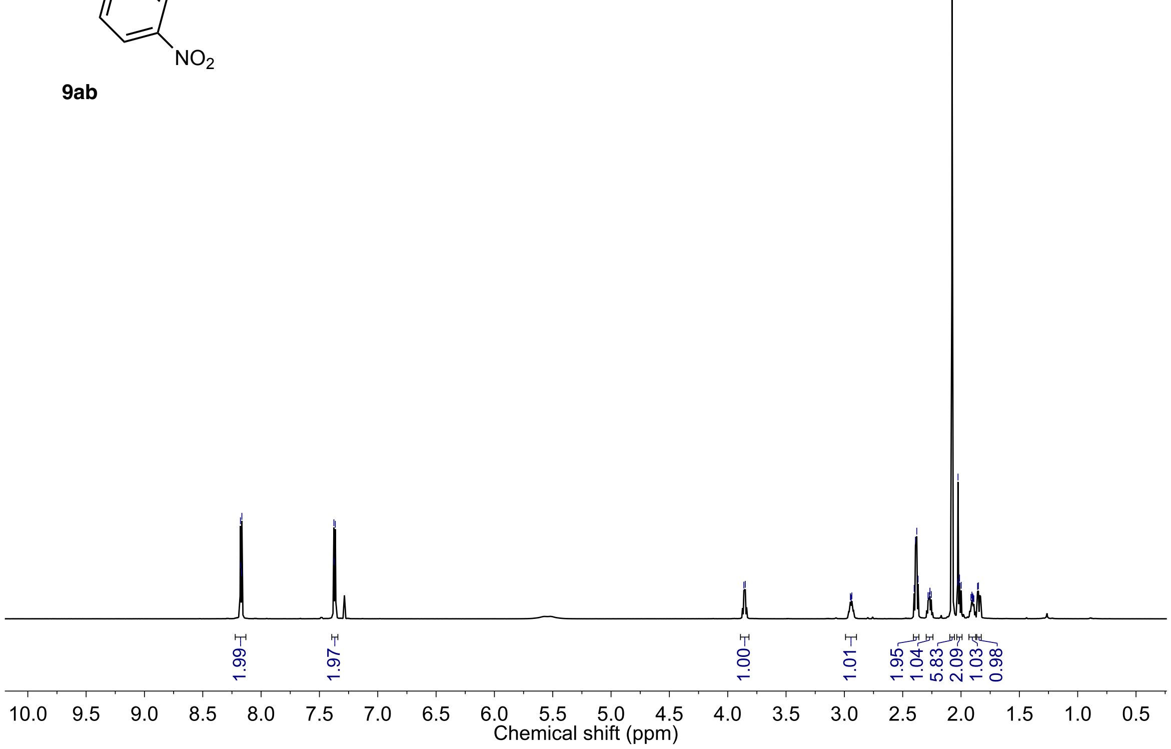
8.18
8.17
8.17
8.16
7.38
7.37
7.36
3.86
3.85
2.95
2.94
2.94
2.40
2.39
2.38
2.37
2.28
2.27
2.26
2.08
2.03
2.03
2.02
2.01
2.00
1.91
1.86
1.99
1.97
1.00
1.01
1.95
1.04
5.83
2.09
1.03
0.98
10.0 9.5 9.0 8.5 8.0 7.5 7.0 6.5 6.0 5.5 5.0 4.5 4.0 3.5 3.0 2.5 2.0 1.5 1.0 0.5
Chemical shift (ppm)

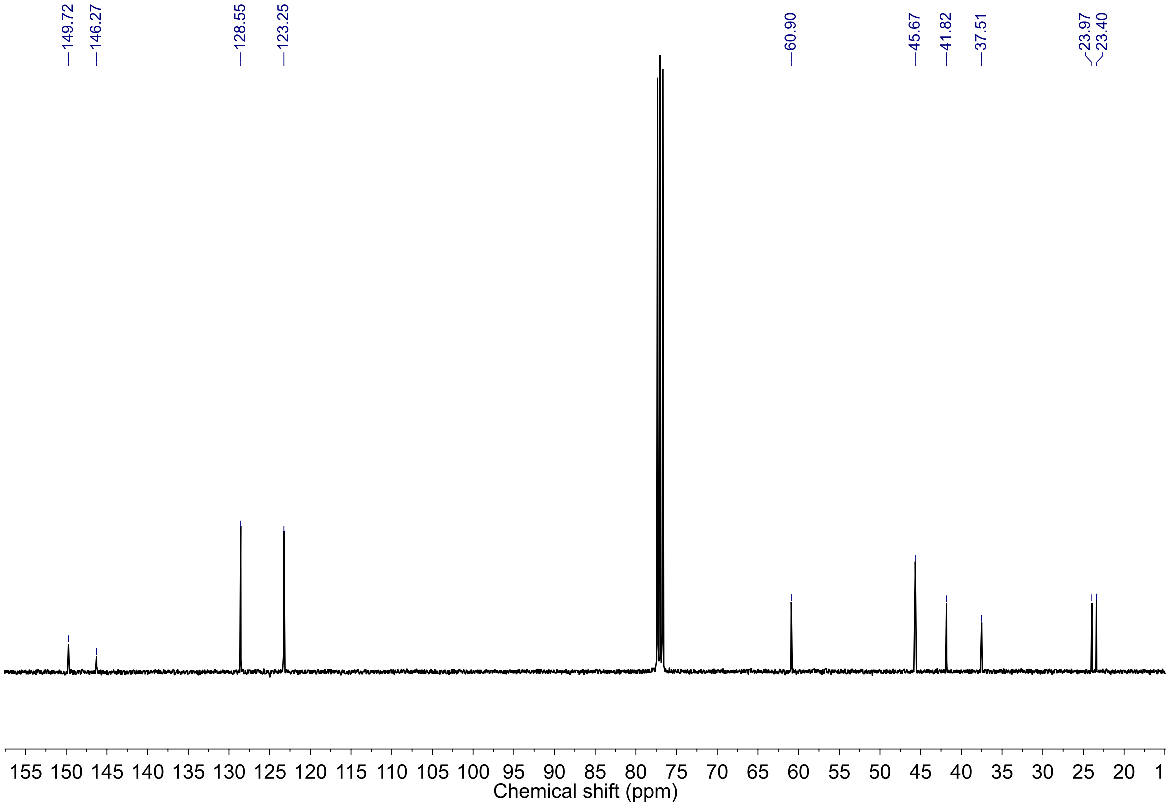
149.72
146.27
128.55
123.25
60.90
45.67
41.82
37.51
23.97
23.40
155 150 145 140 135 130 125 120 115 110 105 100 95 90 85 80 75 70 65 60 55 50 45 40 35 30 25 20
Chemical shift (ppm)

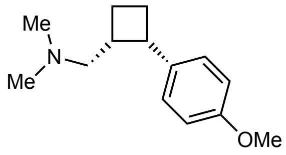
Me
N
Me
OMe

9ac

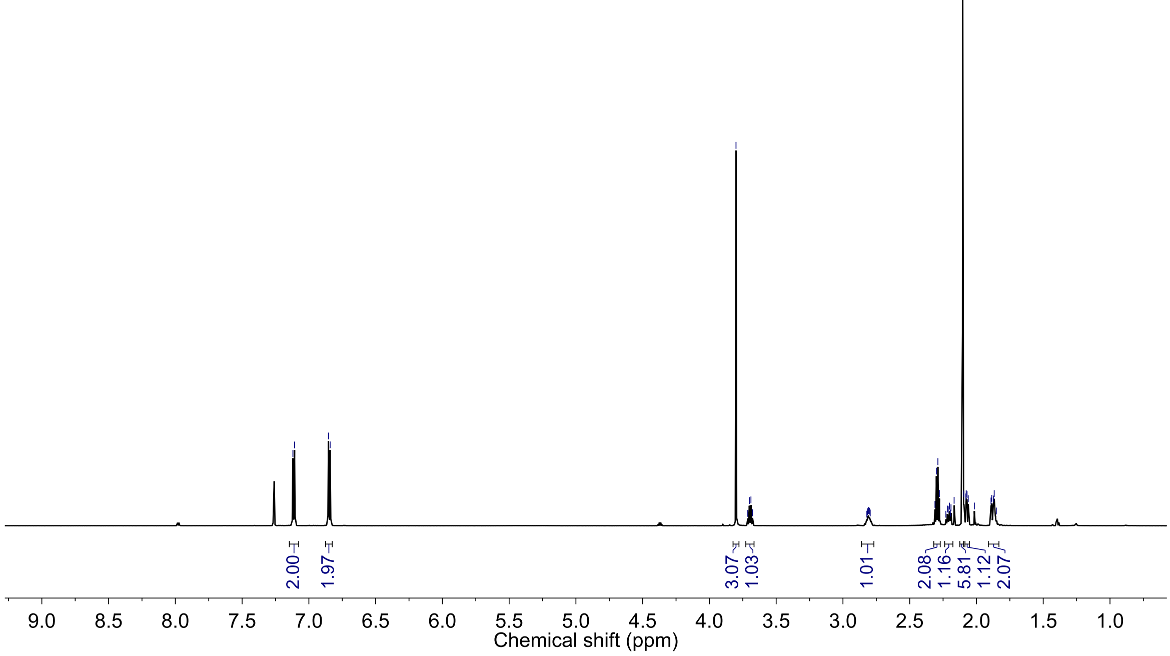
7.12
7.11
6.85
6.84
3.80
3.71
3.70
3.69
3.68
2.82
2.82
2.81
2.80
2.80
2.31
2.30
2.29
2.28
2.17
2.10
2.09
2.08
2.08
2.07
2.06
1.89
1.89
1.87
2.00
1.97
3.07
1.03
1.01
2.08
1.16
5.81
1.12
2.07
9.0
8.5
8.0
7.5
7.0
6.5
6.0
5.5
5.0
4.5
4.0
3.5
3.0
2.5
2.0
1.5
1.0
Chemical shift (ppm)

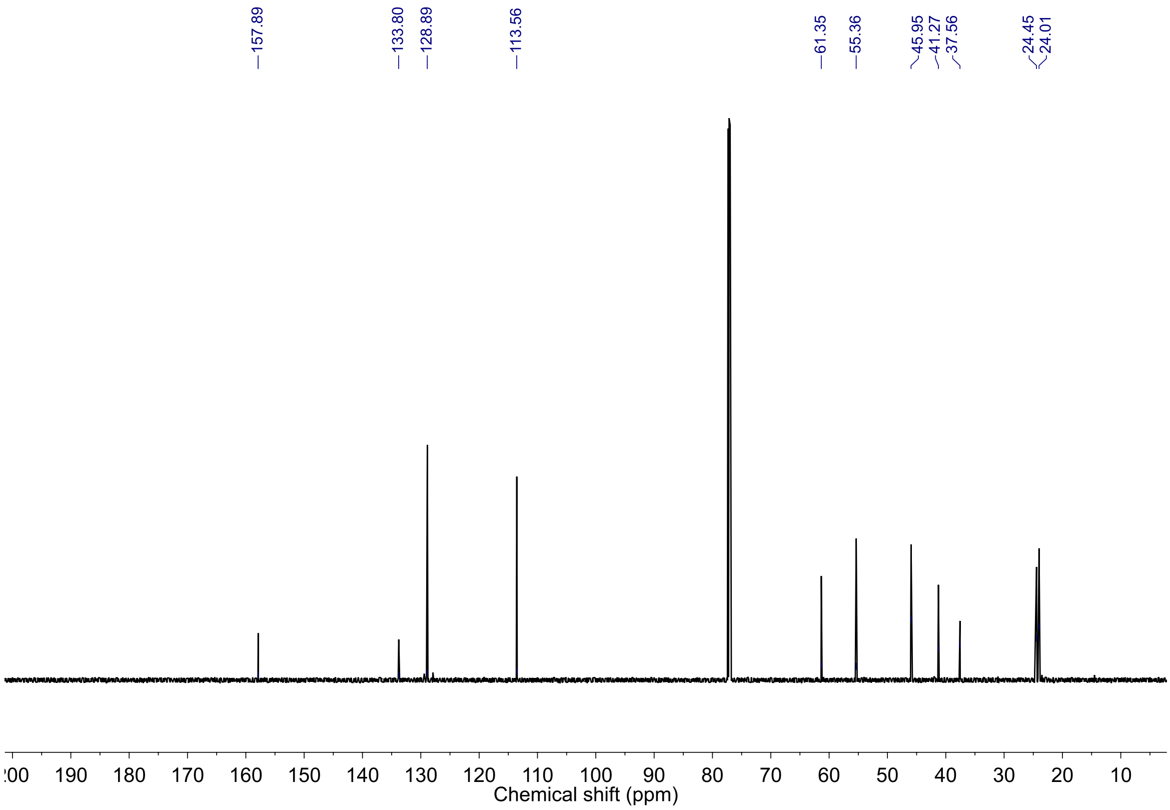
157.89
133.80
128.89
113.56
61.35
55.36
45.95
41.27
37.56
24.45
24.01
190
180
170
160
150
140
130
120
110
100
90
80
70
60
50
40
30
20
10
Chemical shift (ppm)

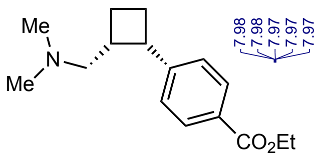
Me
N
Me
CO2Et

9ad

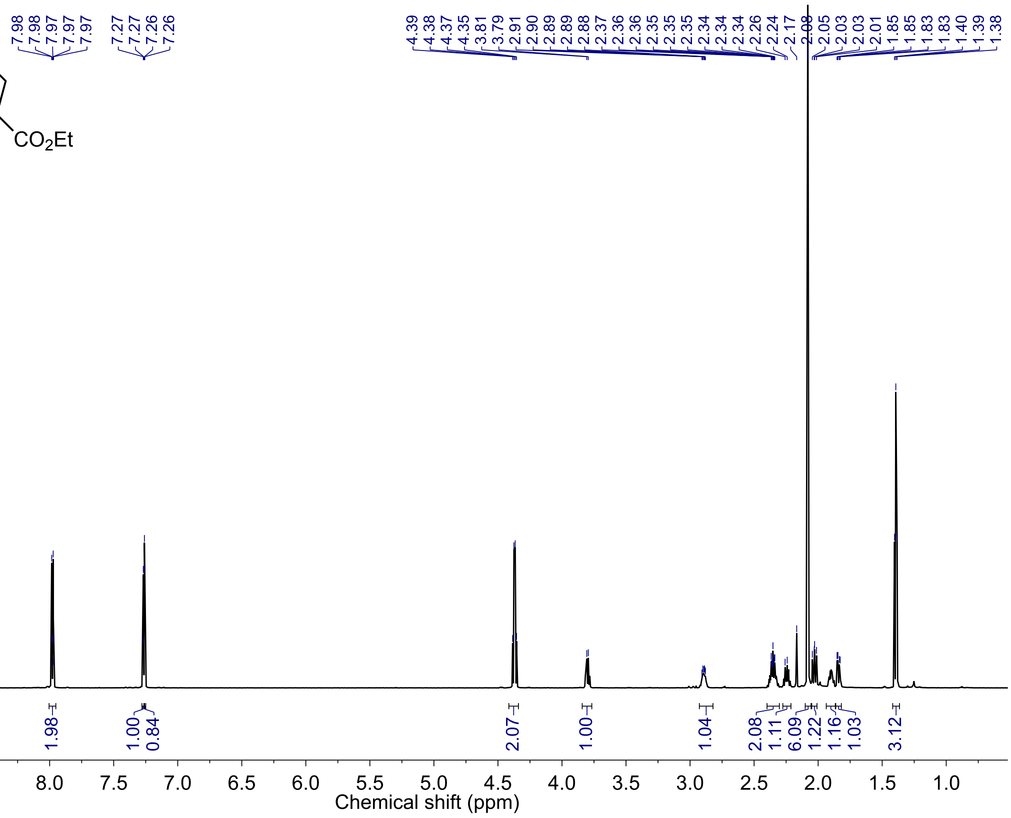
7.98
7.98
7.97
7.97
7.97
7.27
7.27
7.26
7.26
4.39
4.38
4.37
4.35
3.81
3.79
2.91
2.90
2.89
2.89
2.88
2.37
2.36
2.36
2.35
2.35
2.35
2.34
2.34
2.34
2.26
2.24
2.17
2.08
2.05
2.03
2.01
1.85
1.83
1.83
1.40
1.39
1.38
1.98
1.00
0.84
2.07
1.00
1.04
2.08
1.11
6.09
1.22
1.16
1.03
3.12
9.5
9.0
8.5
8.0
7.5
7.0
6.5
6.0
5.5
5.0
4.5
4.0
3.5
3.0
2.5
2.0
1.5
1.0
Chemical shift (ppm)

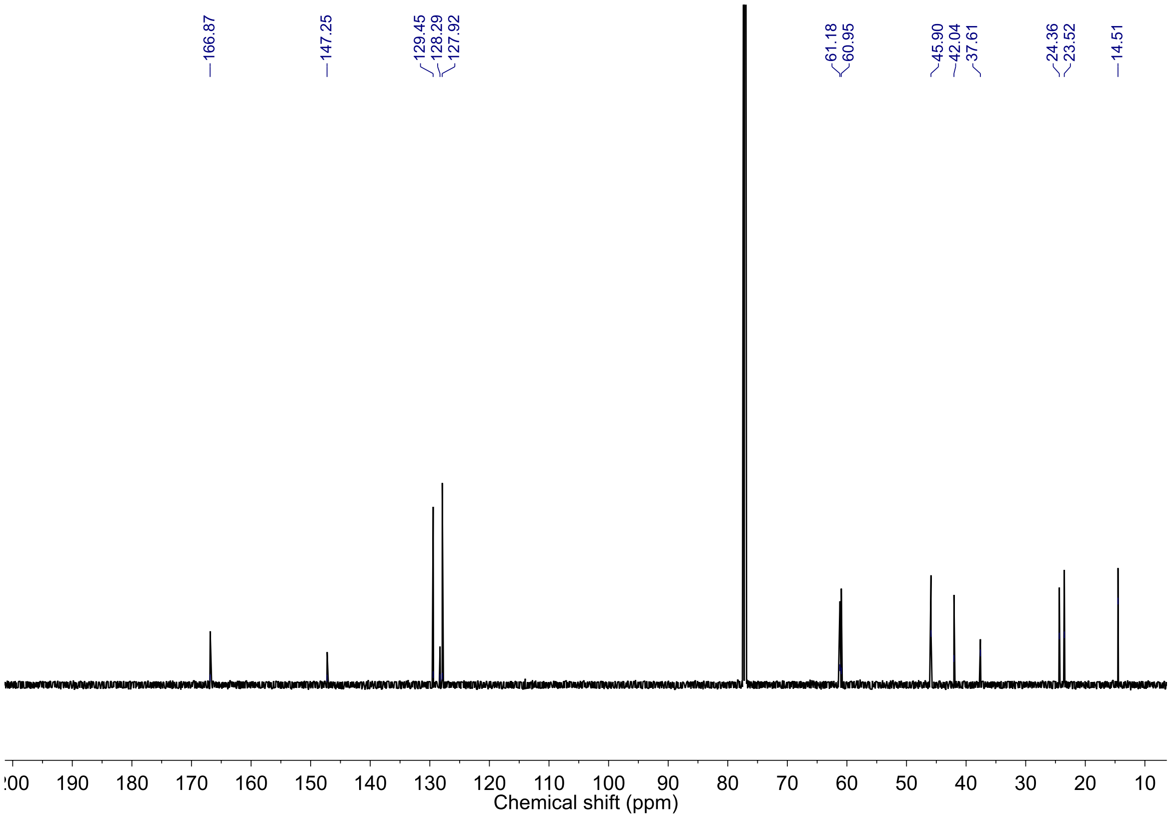
166.87
147.25
129.45
128.29
127.92
61.18
60.95
45.90
42.04
37.61
24.36
23.52
14.51
200
190
180
170
160
150
140
130
120
110
100
90
80
70
60
50
40
30
20
10
Chemical shift (ppm)

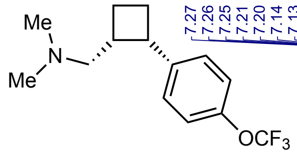
Me
N
Me
OCF$_3$

9ae

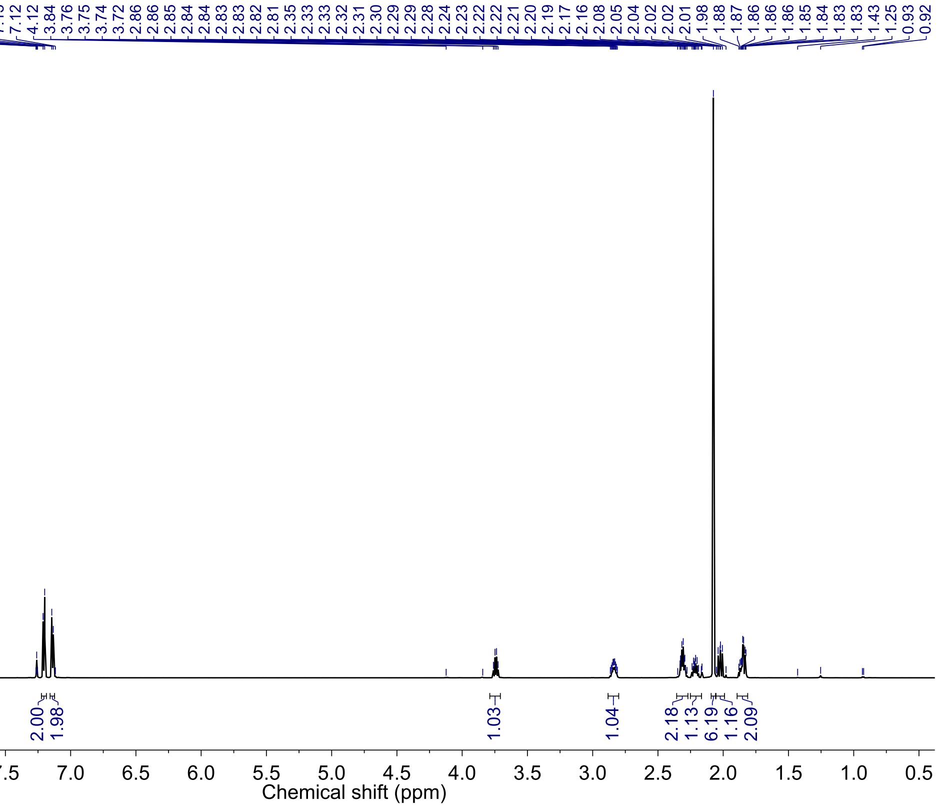
7.27 7.26 7.25 7.21 7.20 7.14 7.13 7.12 4.12 3.84 3.76 3.75 3.74 3.72 2.86 2.86 2.85 2.84 2.84 2.83 2.83 2.82 2.81 2.35 2.33 2.33 2.32 2.31 2.30 2.29 2.29 2.28 2.24 2.23 2.22 2.22 2.21 2.20 2.19 2.17 2.16 2.08 2.05 2.04 2.02 2.02 2.01 1.98 1.88 1.87 1.86 1.86 1.85 1.84 1.83 1.83 1.43 1.25 0.93 0.92
2.00 1.98 1.03 1.04 2.18 1.13 6.19 1.16 2.09
9.0 8.5 8.0 7.5 7.0 6.5 6.0 5.5 5.0 4.5 4.0 3.5 3.0 2.5 2.0 1.5 1.0 0.5
Chemical shift (ppm)

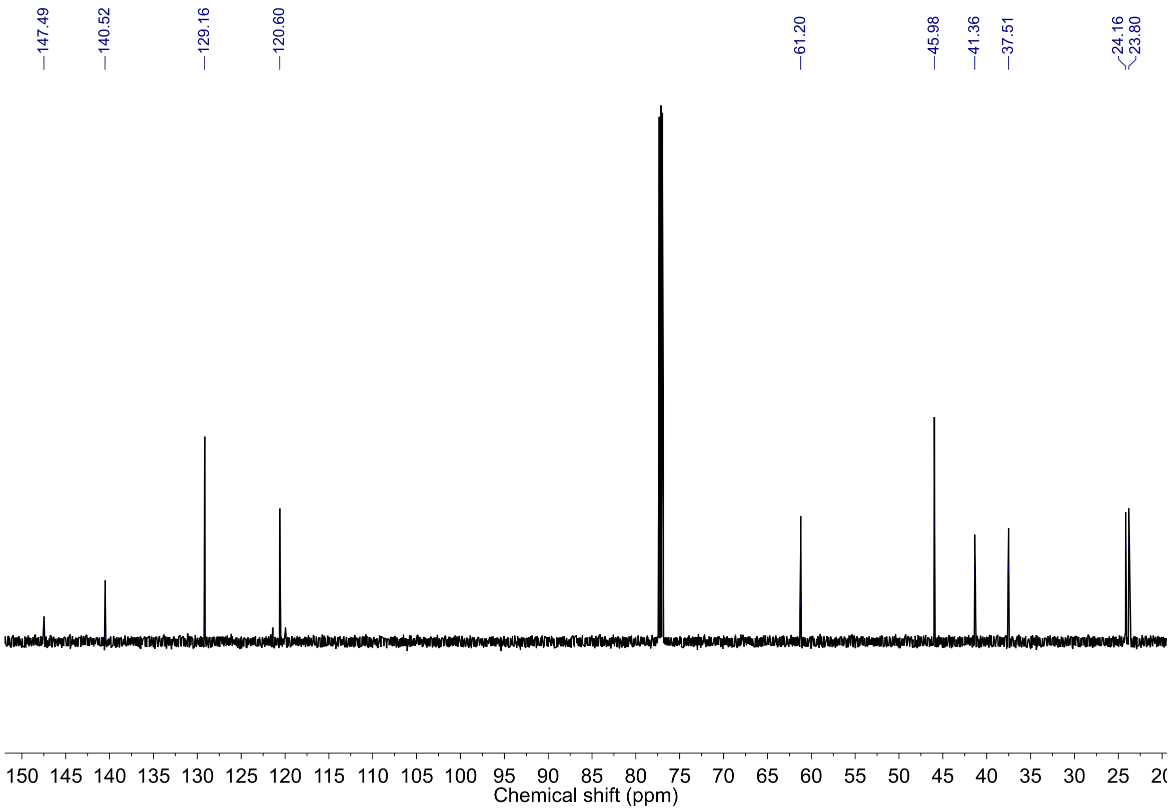
147.49 140.52 129.16 120.60 61.20 45.98 41.36 37.51 24.16 23.80
150 145 140 135 130 125 120 115 110 105 100 95 90 85 80 75 70 65 60 55 50 45 40 35 30 25
Chemical shift (ppm)

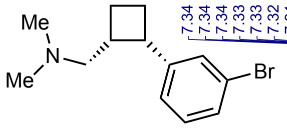
Me
N
Me
Br

9af

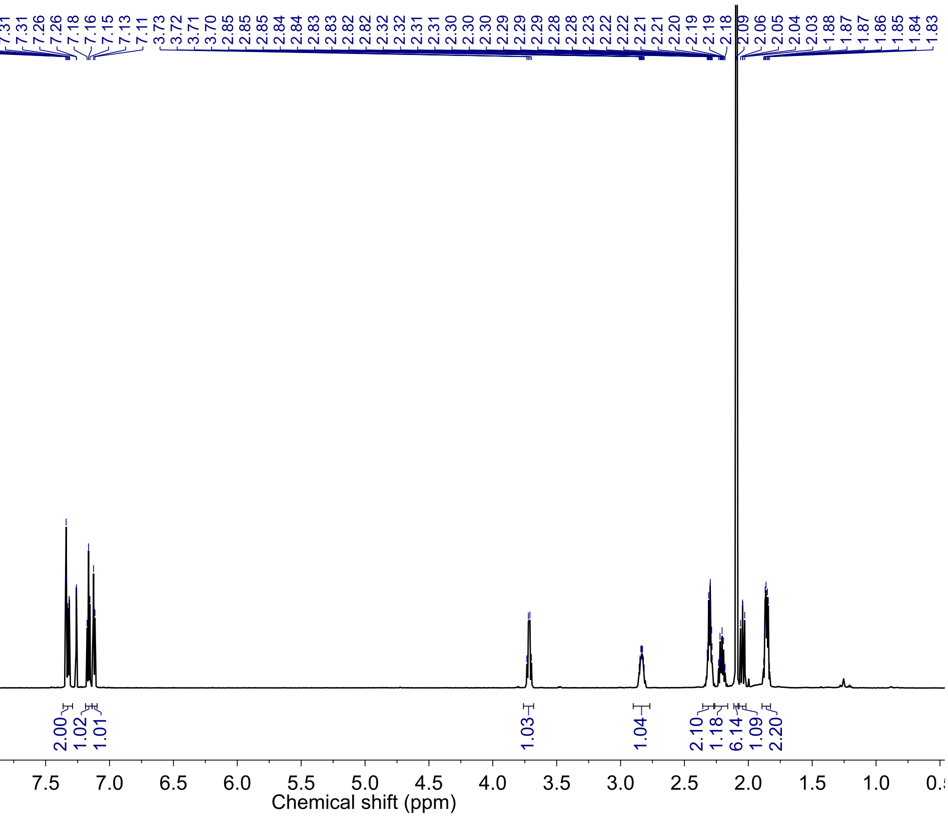
7.34
7.34
7.34
7.33
7.32
7.31
7.26
7.18
7.16
7.15
7.13
7.11
3.73
3.72
3.71
3.70
2.85
2.85
2.85
2.84
2.84
2.83
2.82
2.82
2.32
2.32
2.31
2.31
2.30
2.30
2.29
2.29
2.28
2.28
2.23
2.22
2.22
2.21
2.21
2.20
2.19
2.19
2.18
2.09
2.06
2.05
2.04
2.03
1.88
1.87
1.87
1.86
1.85
1.84
1.83
2.00
1.02
1.01
1.03
1.04
2.10
1.18
6.14
1.09
2.20
9.0
8.5
8.0
7.5
7.0
6.5
6.0
5.5
5.0
4.5
4.0
3.5
3.0
2.5
2.0
1.5
1.0
Chemical shift (ppm)

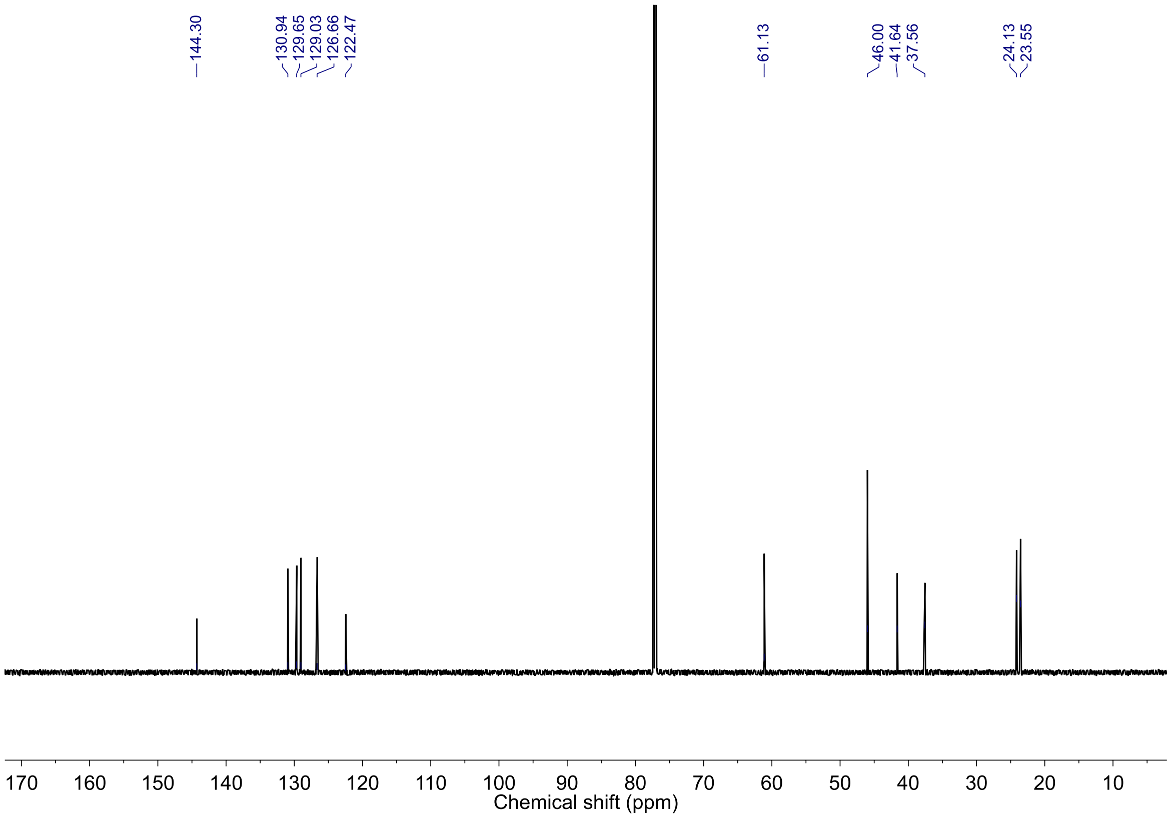
144.30
130.94
129.65
129.03
126.66
122.47
61.13
46.00
41.64
37.56
24.13
23.55
170
160
150
140
130
120
110
100
90
80
70
60
50
40
30
20
10
Chemical shift (ppm)

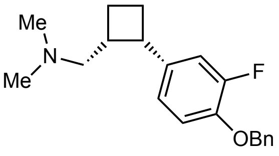
Me
N
Me
F
OBn

9ag

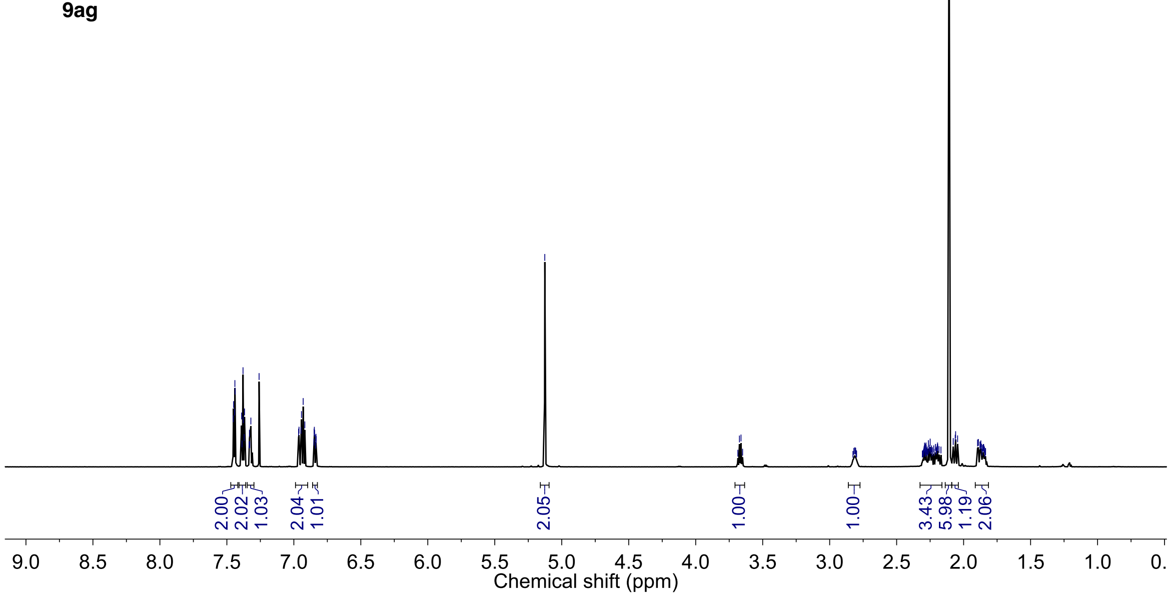
7.45 7.45 7.44 7.44 7.39 7.39 7.38 7.38 7.37 7.37 7.33 7.33 7.33 7.32 7.26 6.96 6.96 6.95 6.94 6.93 6.92 6.85 6.85 6.85 6.85 6.84 6.84 6.83 5.13 3.67 3.66 2.30 2.29 2.29 2.29 2.28 2.28 2.28 2.26 2.25 2.25 2.24 2.22 2.21 2.20 2.20 2.19 2.19 2.18 2.11 2.08 2.06 2.06 2.04 1.90 1.89 1.88 1.87 1.87 1.87 1.86 1.85 1.85 1.85 1.84
2.00 2.02 1.03 2.04 1.01 2.05 1.00 1.00 3.43 5.98 1.19 2.06
9.0 8.5 8.0 7.5 7.0 6.5 6.0 5.5 5.0 4.5 4.0 3.5 3.0 2.5 2.0 1.5 1.0 0.
Chemical shift (ppm)

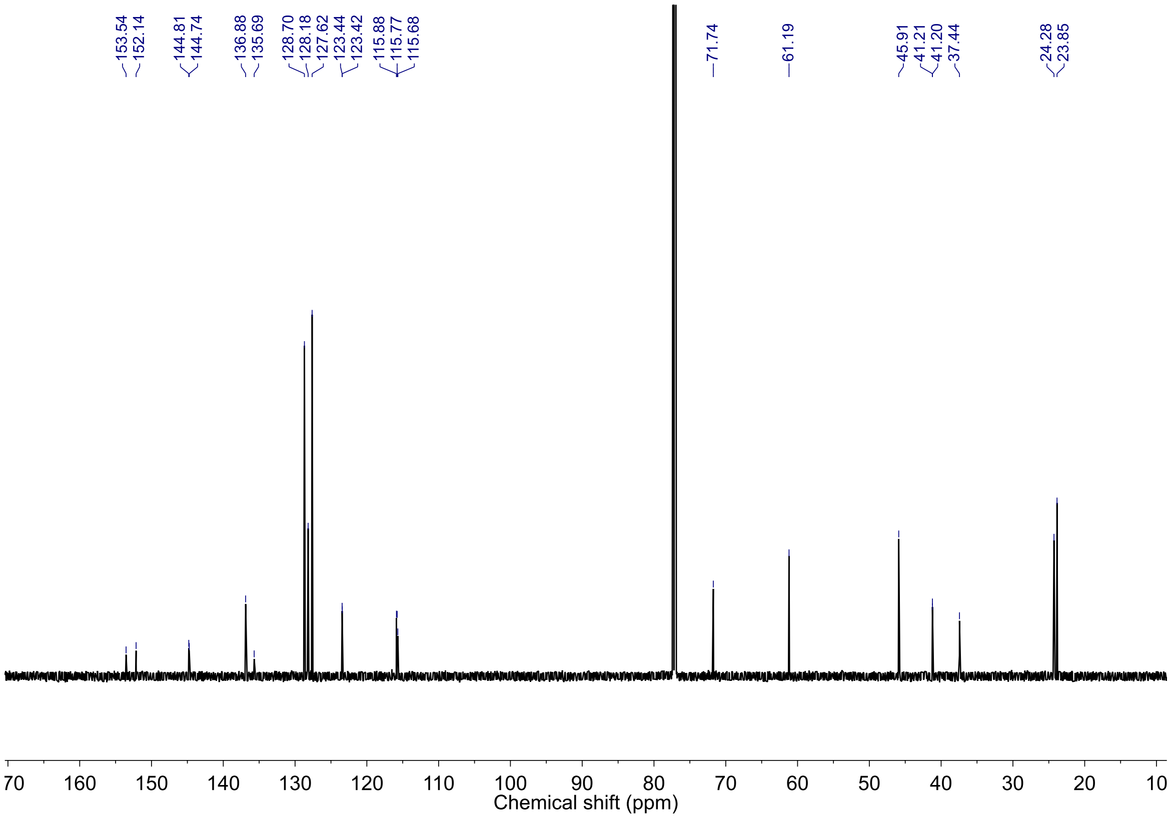
153.54 152.14 144.81 144.74 136.88 135.69 128.70 128.18 127.62 123.44 123.42 115.88 115.77 115.68 71.74 61.19 45.91 41.21 41.20 37.44 24.28 23.85
70 160 150 140 130 120 110 100 90 80 70 60 50 40 30 20 10
Chemical shift (ppm)

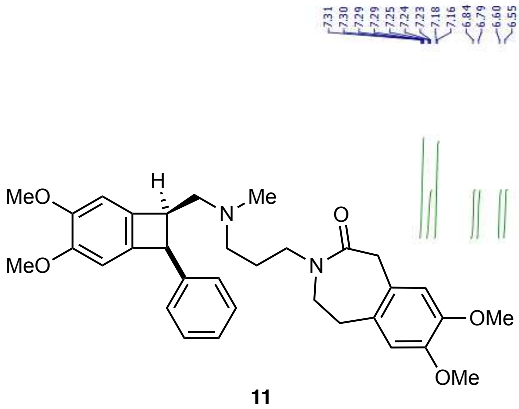

11

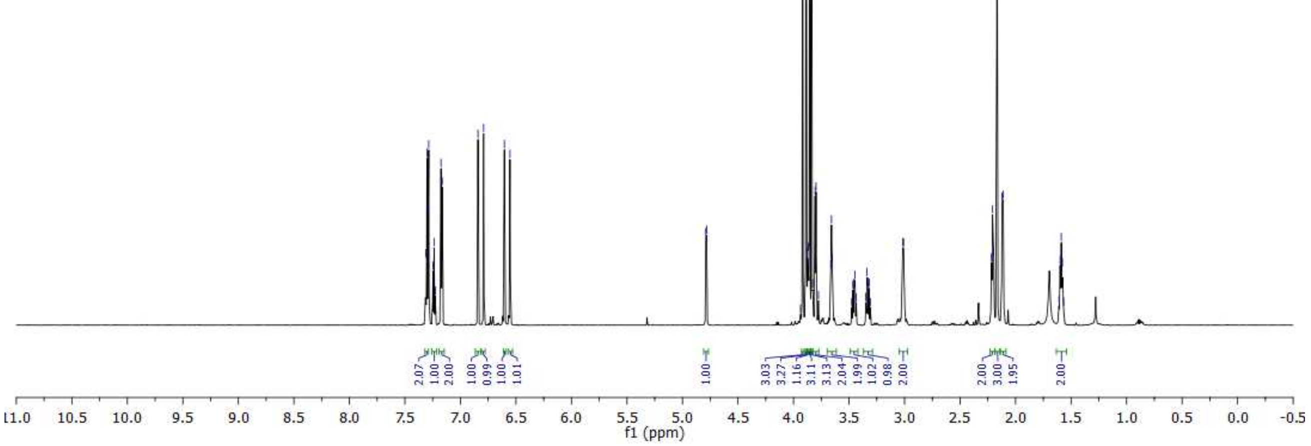

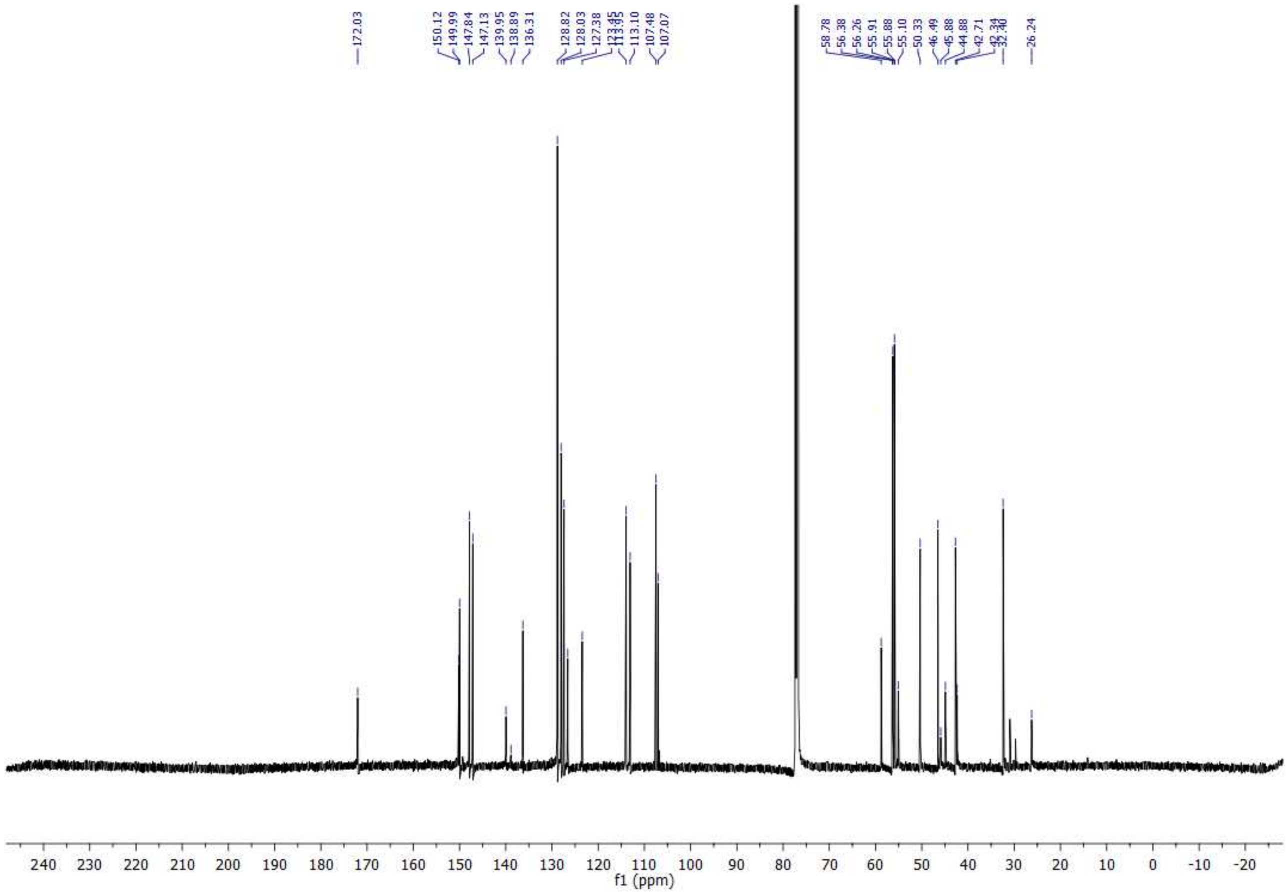

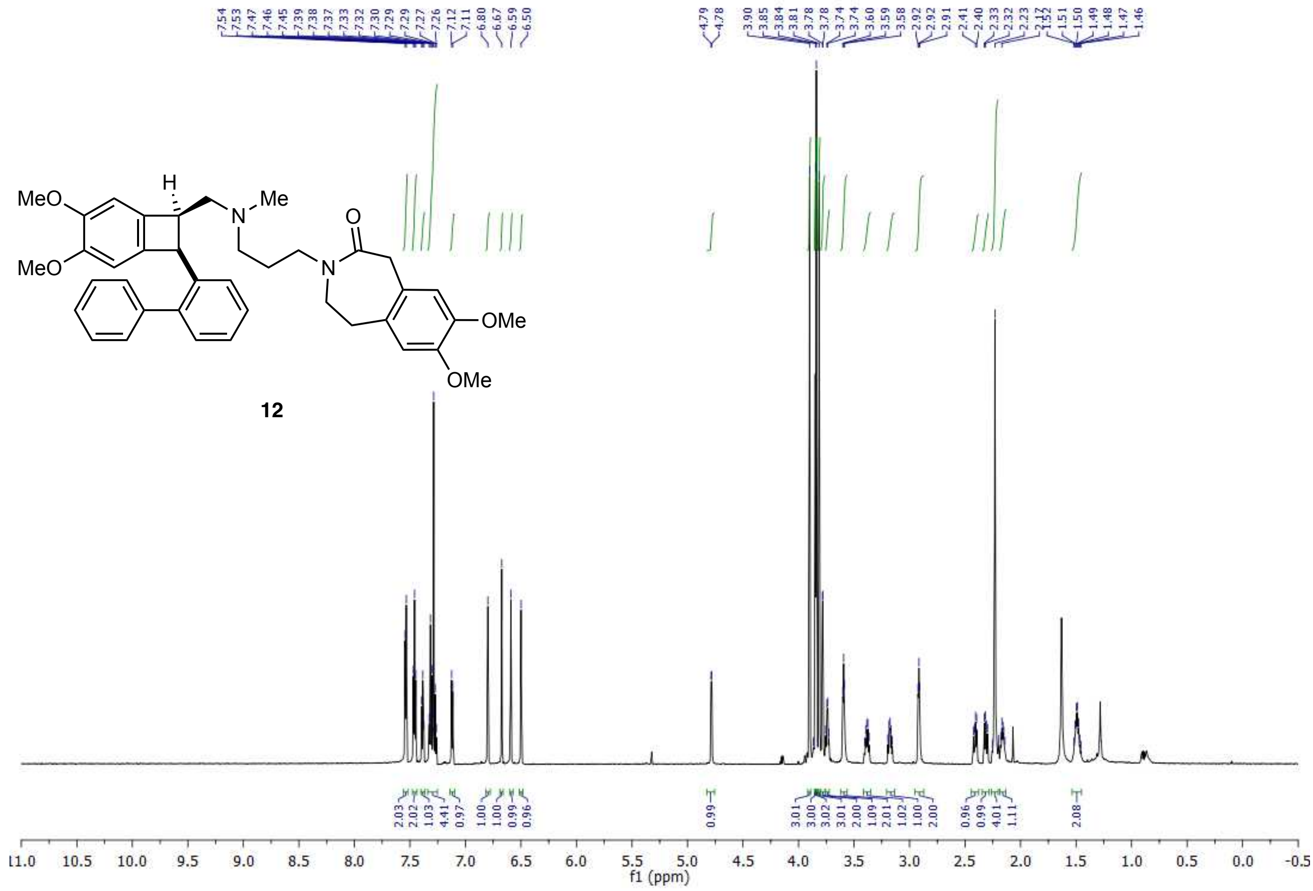
MeO
MeO
H
N
Me
O
N
OMe
OMe
12
f1 (ppm)
